# Study on the mechanism of Shenmai injection in the treatment of sepsis

**DOI:** 10.1111/jcmm.70201

**Published:** 2024-11-25

**Authors:** Mengxia Yang, Tengfei Chen, Yue Xu, Qingquan Liu, Xiaolong Xu

**Affiliations:** ^1^ Beijing Hospital of Traditional Chinese Medicine Capital Medical University Beijing China; ^2^ Beijing institute of Traditional Chinese Medicine Beijing China; ^3^ Graduate School of Beijing University of Chinese Medicine Beijing China; ^4^ China Science and Technology Development Center for Chinese Medicine Beijing China

**Keywords:** bioinformatics, mechanism, molecular docking, network pharmacology, sepsis, Shenmai injection

## Abstract

Shenmai injection (SMI) is widely used in the clinical treatment of sepsis, but its mechanism is not yet clear. This study aimed to explore the molecular mechanism through network pharmacology, bioinformatics, and molecular docking technologies. The active ingredients and targets of SMI were screened through traditional Chinese medicine databases and the Swiss Target Prediction database, respectively. The disease genes were searched using GEO and GeneCards databases, and Venn mapping was used to screen potential therapeutic targets. The key targets were selected using Cytoscape 3.9.1 software. The BioGPS database was used to evaluate the expression of these targets in tissues/cells. The DAVID database is used for enrichment analysis. Molecular docking technology was used to evaluate the interaction between these targets and core active ingredients. 122 potential therapeutic targets and 28 key targets were identified. Forty‐six potential therapeutic targets showed highly specific expression in 40 tissues/cells. The PI3K‐AKT, RAP1, and MAPK signalling pathways are highly enriched. The molecular docking results showed good interactions. This study systematically analysed the mechanism of SMI in treating sepsis, involving multiple targets and pathways, possibly related to anti‐inflammatory, anti‐oxidative stress, and immune regulation, providing reference value for future basic research of sepsis.

## 1 INTRODUCTION

Sepsis is a heterogeneous syndrome that can be divided into sepsis and septic shock based on the progression of the disease: sepsis is typically defined as a life‐threatening organ dysfunction syndrome caused by a dysregulated host response to infection, while compared to sepsis, septic shock is usually accompanied by particularly severe circulatory, cellular, and metabolic abnormalities, the severity of which can significantly increase the risk of death.^1^, ^2^ Both sepsis and septic shock share similar pathogenic mechanisms, including systemic inflammatory response syndrome, ischemia–reperfusion injury, oxidative and antioxidative imbalance, immune dysfunction, mitochondrial dysfunction, cell apoptosis, and autophagy disruption.^3^ Reportedly, the incidence of sepsis has significantly increased globally, affecting over 30 million patients annually and resulting in nearly 6 million deaths.^4^ Moreover, it has been found that 6%–30% of patients in intensive care units develop sepsis,^5^ with an in‐hospital mortality rate exceeding 10% for sepsis patients and over 40% for septic shock patients.^1^ Although with the continuous development and progress of modern medicine, there are various treatment options available for sepsis in clinical practice, such as early fluid resuscitation, antimicrobial therapy, hemodynamic management, mechanical ventilation, blood purification, nutritional support, and other therapies.^6^ However, the incidence and mortality rates remain high, and the high cost of treatment imposes a heavy burden on patients, their families, and the entire society.^7^

In recent years, with the continuous development of traditional Chinese medicine, we have discovered its unique advantages in the treatment of sepsis, which involves multiple targets and comprehensive regulation. It also has a certain clinical application basis and development space.^8^ Traditional Chinese medicine believes that the principle of ‘strengthening healthy energy’ should be consistently applied throughout the treatment of sepsis.^9^ As a traditional Chinese medicine injection, Shenmai Injection(SMI) is an important recommended medication for ‘strengthening healthy energy’ in the treatment of sepsis.^10^ Research has found that SMI not only reduces the APACHE II score of sepsis patients,^11^ but also decreases the ICU mortality rate, length of ICU stay, in‐hospital mortality rate, and total hospitalization time.^12^ However, its mechanism is still unclear.

Currently, as an emerging discipline, network pharmacology and bioinformatics are developing rapidly and have unique advantages in predicting the mechanism of traditional Chinese medicine. It is a promising approach.^13^, ^14^ Based on this, this study aims to preliminarily explore the molecular mechanism of SMI in the treatment of sepsis through network pharmacology and bioinformatics methods, in order to provide new insights for clinical and basic research. The technical roadmap of this study is shown in Figure S1.

## 2 MATERIALS AND METHODS

### 2.1 Prediction of potential therapeutic targets for SMI in the treatment of sepsis

#### 2.1.1 Acquisition of active ingredients of SMI

The Traditional Chinese Medicine Systems Pharmacology Database (TCMSP, http://lsp. Nwsuaf.edu.cn/tcmsp.php) is a unique platform that integrates systems biology and pharmacology. The platform collects 499 medicinal herbs and 12,144 chemical substances, providing their oral bioavailability, drug‐likeness, intestinal epithelial permeability, blood–brain barrier, and other pharmacokinetic properties.^15^ In this study, the TCMSP database was used to retrieve active ingredients according to the screening criteria: oral bioavailability (OB) ≥ 30% and drug‐likeness index (DL) ≥ 0.18. When the active ingredients of traditional Chinese medicine cannot be found in the TCMSP database, they are then searched in the Traditional Chinese Medicine Information Database(TCMID, http:// www.megabionet.org /tcmid/) and Traditional Chinese medicine@Taiwan (TCM Database@Taiwan, http://tcm.cmu.edu.tw/), and the retrieved active ingredients are queried for their OB and DL values in TCMSP. TCM‐ID is a key data resource center that promotes research on traditional Chinese medicine and clinical studies, containing 7443 high‐quality prescriptions and 27,631 human samples.^16^ The Traditional Chinese Medicine @ Taiwan Database is currently the world's largest non‐commercial Chinese medicine database, containing over 20,000 purified compounds isolated from 453 types of Chinese herbal medicine.^17^

#### 2.1.2 Screening of targets of SMI active ingredients

The PubChem database (https://pubchem.ncbi.nlm.nih.gov/) is an open chemical database of the National Institutes of Health (NIH), which primarily collects information on the chemical structures, identifiers, chemical and physical properties, as well as biological activities of small molecules.^18^ The Swiss Target Prediction database (http://www.swisstargetprediction.ch/) contains 370,000 known active substances of more than 3000 proteins from three different species. Based on a combination of 2D and 3D similarity, it can be used to predict the targeted actions of small molecules.^19^ We input the SMI active ingredients into the PubChem database to obtain typical Canonical SMILES structures, and then import them into the Swiss Target Prediction database for screening targets, retaining targets with a Probability >0, and finally constructing a network diagram of herbs‐active ingredients‐targets.

#### 2.1.3 Establishment of a sepsis disease gene library

The Gene Expression Omnibus database (GEO, https://www.ncbi.nlm.nih.gov/geo/) was established by the National Center for Biotechnology Information (NCBI) in 2000 as a high‐throughput gene expression database, which includes a large amount of data from microarray chips, next‐generation sequencing, and other forms of high‐throughput genomic data.^20^ In this database, we used ‘sepsis’ and ‘normal’ as search terms, selected ‘series’ as the entry type, ‘Homo sapiens’ as the species, and screened the dataset GSE137342 (provided by the GPL10558 platform, Illumina HumanHT‐12 V4.0 expression beadchip). We then used the online tool GEO2R to compare blood samples from septic patients with those from normal patients to screen for differentially expressed genes (DEGs).

The GeneCards database (https://www.genecards.org/) automatically integrates gene‐centred data from 150 network sources, including genomics, transcriptomics, proteomics, genetics, clinical, and functional information.^21^ We searched for genes related to sepsis in this database and established a sepsis gene library by merging and deduplicating them with the DEGs.

#### 2.1.4 Venn mapping

Utilizing the Venny 2.1 online tool (https://bioinfogp.cnb.csic.es/tools/venny/), we mapped the drug targets and disease genes to identify potential therapeutic targets of SMI in the treatment of sepsis.

### 2.2 Construction of potential therapeutic targets network

The STRING 12.0 database (https://string‐db.org/) is a protein–protein interaction(PPI) network database based on public databases and literature information, providing protein networks for over 2000 organisms.^22^ Cytoscape is an open‐source software project used for visualizing molecular interaction networks and integrating these networks with phenotype, gene expression profiles, and other molecular state data.^23^

We imported the obtained potential therapeutic targets into the STRING 12.0 database, selected the species as ‘Homo sapiens’, with a confidence score of 0.4, to obtain a PPI network. The PPI network data was then imported into the Cytoscape 3.9.1 software. We filtered key targets based on network topology metrics, including Degree Centrality (DC) ≥2 times the median, Closeness Centrality (CC) ≥ median, and Betweenness Centrality (BC) ≥ median. Additionally, we used the CytoHubba plugin in Cytoscape 3.9.1 to conduct MCC analysis and select the top 10 Hub genes.^24^ Furthermore, we used the MCODE plugin to filter important module genes, with parameters set as ‘Degree cutoff=2, Node score cutoff=0.2, K‐core=2, Max depth=100’ (Version 1.4.2, Bader Lab, University of Toronto).

### 2.3 Expression of potential therapeutic targets in Various Tissues/Cells

BioGPS (http://biogps.org) is a centralized gene annotation portal that can be used to query the expression of genes in tissues/cells.^25^ Genes meeting the following criteria are identified as tissue‐specifically expressed genes: (1) the expression level of the gene in a single tissue/cell is more than 10 times the median; (2) the expression level in the second most abundant tissue/cell does not exceed one‐third of the highest abundance tissue/cell expression level.^26^

### 2.4 Functional enrichment analysis of potential therapeutic targets

The DAVID database (http://david.ncifcrf.gov) is a network‐based, large‐scale integrated annotation knowledgebase primarily used for function and pathway enrichment analysis of genes.^27^ We imported potential therapeutic targets into the DAVID database using the following criteria: ‘Identifier: official_gene_symbol, Species: homo sapiens, List‐Species: homo sapiens, Background‐Species: homo sapiens’ for gene ontology (GO) and Kyoto Encyclopedia of Genes and Genomes (KEGG) enrichment analysis. Based on different functions, the GO analysis can be divided into biological process (BP), cellular component (CC), and molecular function (MF). The filtering criterion is that entries with a false discovery rate (FDR) < 0.05 are considered statistically significant.

### 2.5 Molecular Docking

Five active ingredients from SMI were selected to dock with five proteins in potential therapeutic targets. Initially, the 2D structures of the active ingredients were downloaded in sdf format from the PubChem database. These files were then converted to pdb format using Open Babel GUI 2.4.1 software (https://github.com/openbabel) and saved for later use. Next, the 3D structures of the proteins were queried and downloaded in pdb format from the RCSB Protein Data Bank (RCSB PDB, https://www.pdb.org/). Subsequently, the active ingredients were hydrogenated and designated as ligands using AutoDockTools 1.5.6 software (https://autodock.scripps.edu/), while the proteins were desolvated, hydrogenated, and set as receptors. The docking box was set, and Autogrid 4 and Autodock 4 were used to calculate the binding energy. Finally, PyMOL 2.4.1 software (https://pymol.org/2/) was utilized to visualize the docking results.

## 3 RESULTS

### 3.1 Active ingredients of SMI and their targets

Six active ingredients were screened from the TCMSP, TCMID, and TCM Database@Taiwan databases, including four active ingredients from Hongshen and two active ingredients from Maidong(Table 1). A total of 221 targets of the SMI active ingredients were retrieved from the Swiss Target Prediction database (Table S1). The active ingredients and their targets were imported into the Cytoscape 3.9.1 software to construct a herbs‐active ingredients‐targets network. This network consists of 229 nodes and 288 edges (Figure S2). The network analysis results show that 5,280,537‐n‐trans‐feruloyltyramine (Degree = 105), 8346‐DNOP (Degree = 64), 222,284‐beta‐sitosterol (Degree = 45), 5,280,794‐stigmasterol (Degree = 42), and 119,307‐ginsenoside rh2 (Degree = 26) are core active ingredients.

**TABLE 1:** Basic information of active ingredients in SMI.

Herbs	Active ingredients	Mol ID	PubChem ID	Oral Bioavailability(OB) (%)	Drug‐Likeness(DL)
Maidong	n‐trans‐feruloyltyramine	MOL008647	5,280,537	86.71	0.26
Maidong	Stigmasterol	MOL000449	5,280,794	43.83	0.76
Hongshen	DNOP	MOL002032	8346	40.59	0.4
Hongshen	Beta‐sitosterol	MOL000358	222,284	36.91	0.75
Hongshen	Ginsenoside rh2	MOL005344	119,307	36.32	0.56
Hongshen	(6Z,10E,14E,18E)‐2,6,10,15,19,23‐hexamethyltetracosa‐2,6,10,14,18,22‐hexaene	MOL002372	11,975,273	33.55	0.42

### 3.2 Sepsis disease genes

**FIGURE 1:**
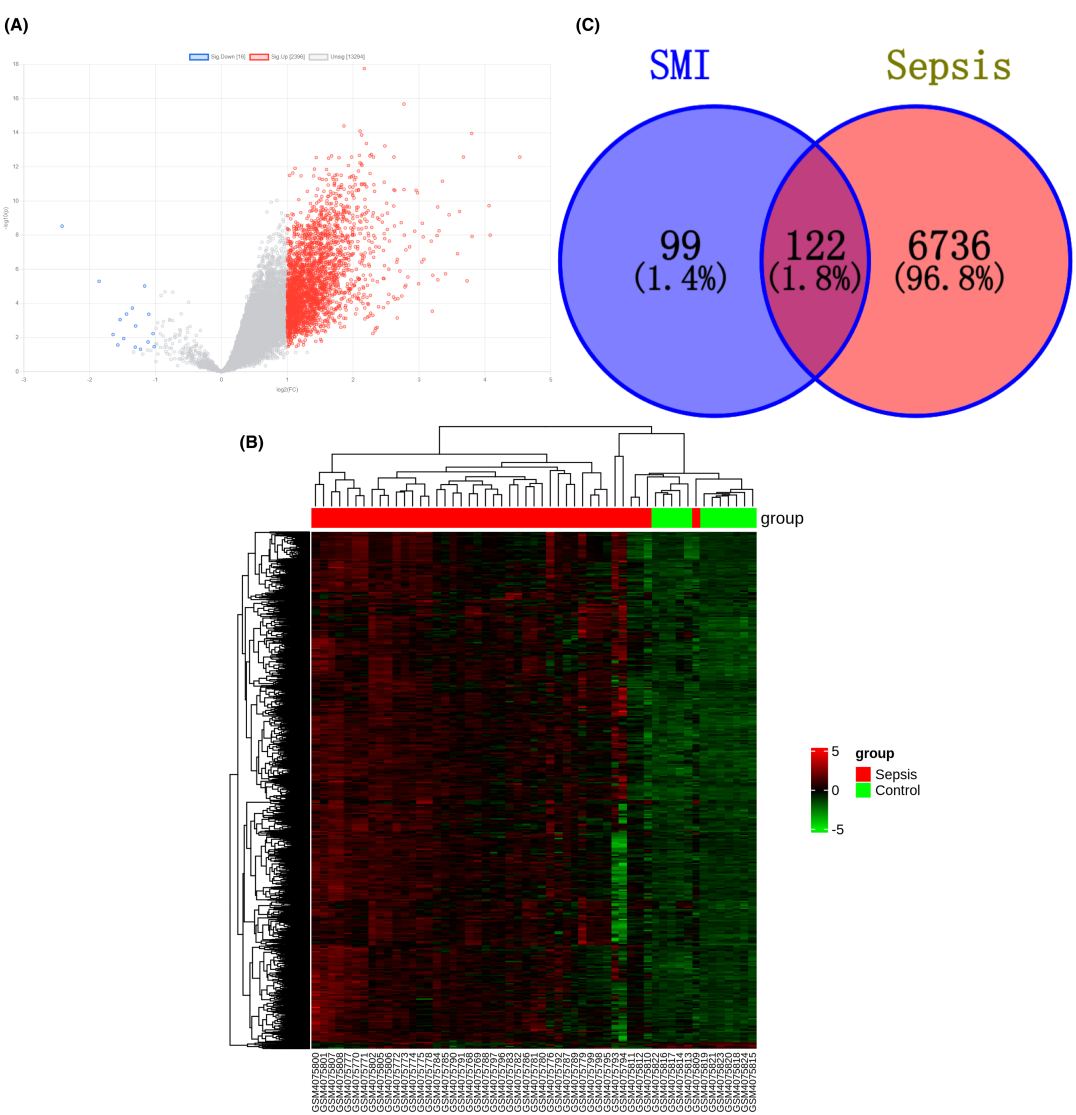
Screening of potential therapeutic targets of SMI in the treatment of sepsis. (A) Volcano map of differential genes in dataset GSE137342. Blue represents downregulated genes, red represents upregulated genes, and grey represents undifferentiated genes. (B) Heat map of DEGs with a multiple of change ≥2 and statistical significance. (C) Venn diagram of SMI targets and sepsis disease genes mapping.

The dataset GSE137342 was obtained by screening the GEO database and contains 55 samples, including 12 samples of normal blood specimens (4 female patients and 8 male patients) and 43 samples of septic blood specimens (14 female patients and 29 male patients), with an average age of 42 years. After analysis using GEO2R, 2396 up‐regulated genes, 16 down‐regulated genes, and 13,294 non‐differential genes were obtained (Figure 1A). We considered genes that meet the following conditions as DEGs: (1) adj. P. Val<0.05; (2) |log FC| ≥ 1 (Fold Change, FC). The results showed a total of 3416 DEGs, including 42 up‐regulated genes and 3374 down‐regulated genes (Figure 1B and Table S2). In addition, we retrieved 3996 sepsis genes from the GeneCards database (Table S3). After merging and deduplication with the 3416 DEGs, a total of 6858 disease genes were obtained.

### 3.3 Potential therapeutic targets

A Venn analysis was conducted on the 221 targets of active ingredients of SMI and the 6858 sepsis disease genes, resulting in 122 intersection genes. These intersection genes are considered as potential therapeutic targets of SMI in the treatment of sepsis(Figure 1C and Table S4).

### 3.4 Potential therapeutic targets interaction network

**FIGURE 2:**
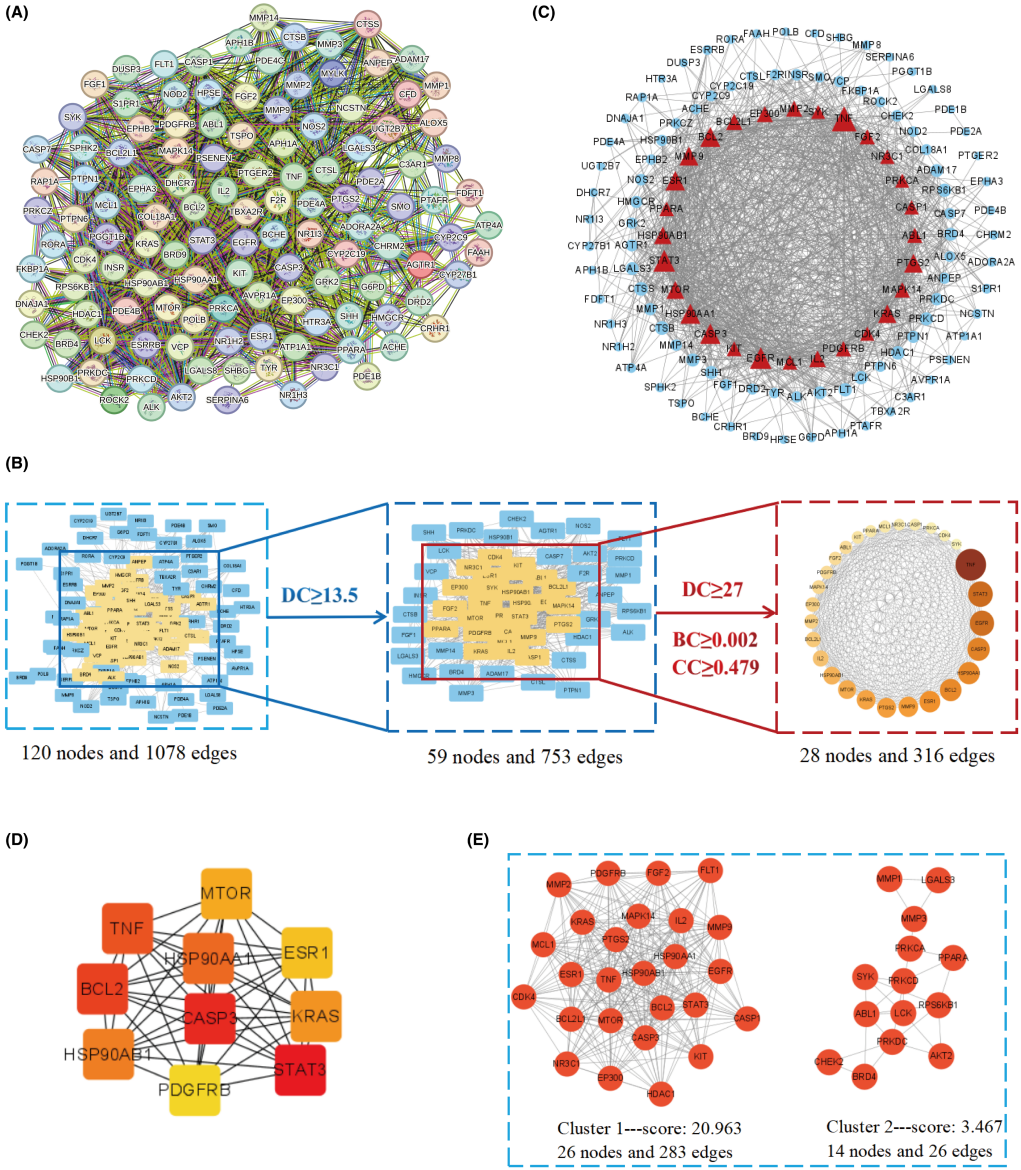
Interaction network of potential therapeutic targets of SMI in the treatment of sepsis. (A) PPI network of potential therapeutic targets. (B) Screening of key targets. After screening the key targets according to the DC≥median, a network consisting of 59 nodes and 753 edges is obtained. Further, according to the DC≥2‐fold median, BC≥median, CC≥median, a network diagram of key targets consisting of 28 edges and 316 nodes is obtained. (C) Network diagram of key targets and non key targets. Red represents key targets and light blue represents non key targets. The size of the node is related to the degree value. The larger the node, the greater the degree value, and the smaller the node, the smaller the degree value. (D) The CytoHubba plugin in Cytoscape 3.9.1 software was used to screen Hub genes of potential therapeutic targets. The colour of node changed from light yellow to red, and the corresponding degree value gradually increased. (E) The MCODE plugin in Cytoscape 3.9.1 software was used to screen important modules of potential therapeutic targets.

We imported the 122 potential therapeutic targets into the STRING 12.0 database to obtain a PPI network (Figure 2A), and combined with analysis using Cytoscape 3.9.1 software. The results showed that the network consisted of 120 nodes and 1078 edges, with median values of DC, BC, and CC being 13.5, 0.002, and 0.479, respectively. Using a criterion of DC ≥2 times the median, 28 key targets were selected (Table 2), and the visualization of key and non‐key targets was carried out (Figure 2B and Figure 2C). The CytoHubba plugin was used to screen the top 10 Hub genes (Figure 2D). Additionally, the MCODE plugin was employed to identify important modules among the potential therapeutic targets, revealing that these targets mainly resided in module 1 (with a score of 20.963, consisting of 26 nodes and 283 edges) and module 2 (with a score of 3.467, consisting of 14 nodes and 26 edges).

**TABLE 2:** Information of 28 core targets.

NO.	Uniprot ID	Gene Symbol	Description	Degree
1	P01375	TNF	Tumour Necrosis Factor	78
2	P40763	STAT3	Signal Transducer And Activator Of Transcription 3	63
3	P00533	EGFR	Epidermal Growth Factor Receptor	62
4	P42574	CASP3	Caspase 3	57
5	P07900	HSP90AA1	Heat Shock Protein 90 Alpha Family Class A Member 1	55
6	P10415	BCL2	BCL2 Apoptosis Regulator	55
7	P03372	ESR1	Oestrogen Receptor 1	53
8	P14780	MMP9	Matrix Metallopeptidase 9	50
9	P35354	PTGS2	Prostaglandin‐Endoperoxide Synthase 2	49
10	P01116	KRAS	KRAS Proto‐Oncogene, GTPase	48
11	P42345	MTOR	Mechanistic Target Of Rapamycin Kinase	46
12	P08238	HSP90AB1	Heat Shock Protein 90 Alpha Family Class B Member 1	44
13	P60568	IL2	Interleukin 2	40
14	Q07817	BCL2L1	BCL2 Like 1	38
15	P08253	MMP2	Matrix Metallopeptidase 2	36
16	Q09472	EP300	E1A Binding Protein P300	36
17	Q16539	MAPK14	Mitogen‐Activated Protein Kinase 14	34
18	P09619	PDGFRB	Platelet Derived Growth Factor Receptor Beta	33
19	P09038	FGF2	Fibroblast Growth Factor 2	33
20	P00519	ABL1	ABL Proto‐Oncogene 1, Non‐Receptor Tyrosine Kinase	33
21	P10721	KIT	KIT Proto‐Oncogene, Receptor Tyrosine Kinase	31
22	Q07869	PPARA	Peroxisome Proliferator Activated Receptor Alpha	30
23	Q07820	MCL1	MCL1 Apoptosis Regulator, BCL2 Family Member	30
24	P04150	NR3C1	Nuclear Receptor Subfamily 3 Group C Member 1	29
25	P29466	CASP1	Caspase 1	29
26	P17252	PRKCA	Protein Kinase C Alpha	28
27	P11802	CDK4	Cyclin Dependent Kinase 4	28
28	P43405	SYK	Spleen Associated Tyrosine Kinase	27

### 3.5 Tissues/cells‐specific expression of potential therapeutic targets

**FIGURE 3:**
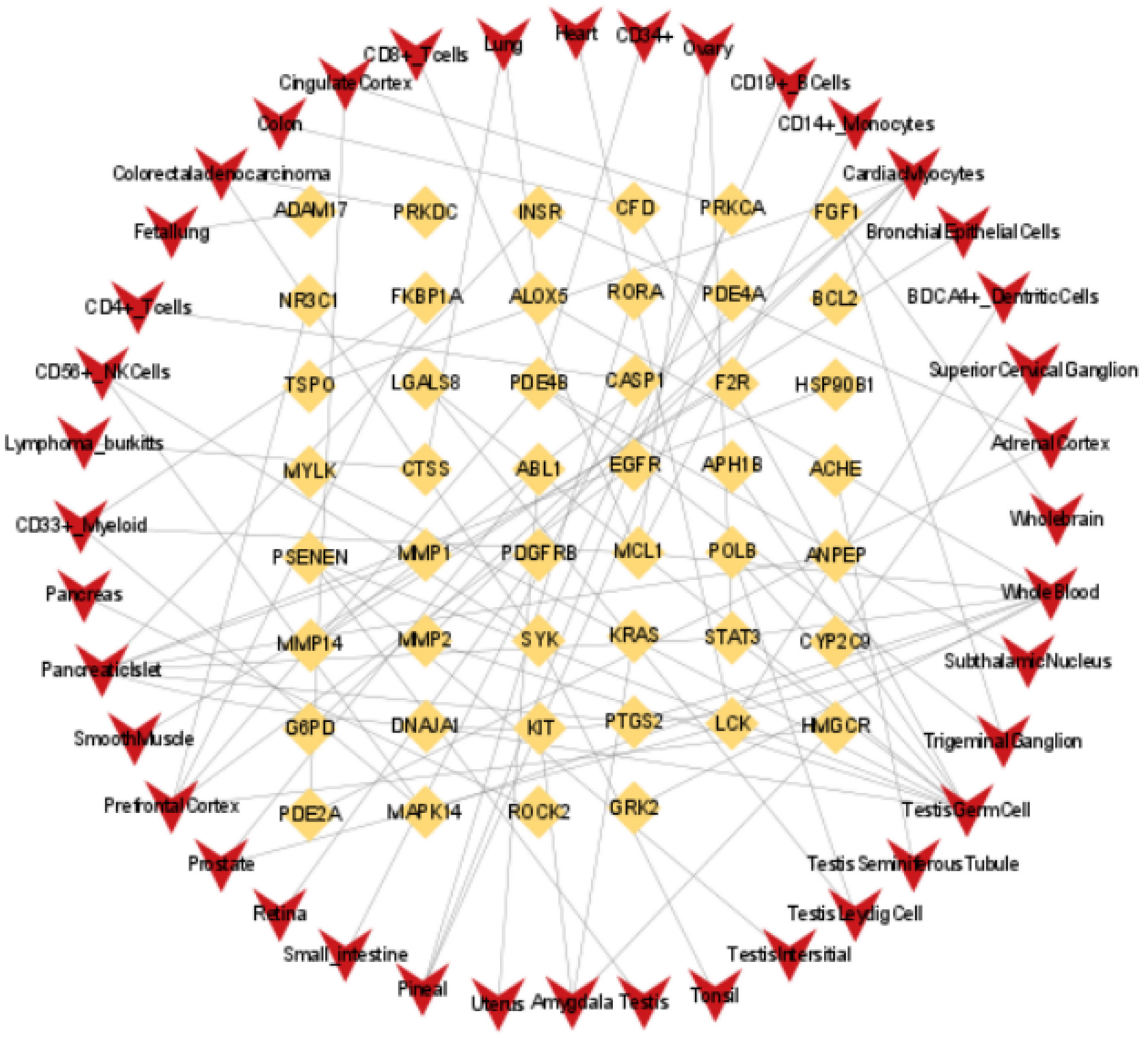
Network diagram of potential therapeutic targets‐tissues/cells. Yellow represents potential therapeutic targets and red represents specific tissues/cells.

By searching the BioGPS database, we found that most potential therapeutic targets exhibit high expression levels in multiple tissues/cells. We identified 46 potential therapeutic targets showing highly specific expression in 40 tissues/cells, mainly involving the heart, lungs, brain, pancreas, as well as immune cells such as CD4^+^ and CD8^+^ (Table S5). This indicates that SMI can not only treat sepsis but also address multi‐organ dysfunction associated with sepsis. Additionally, analysis using Cytoscape 3.9.1 software revealed that the potential therapeutic targets‐tissues/cells network consists of 86 nodes and 83 edges (Figure 3).

### 3.6 GO functional enrichment analysis

**FIGURE 4:**
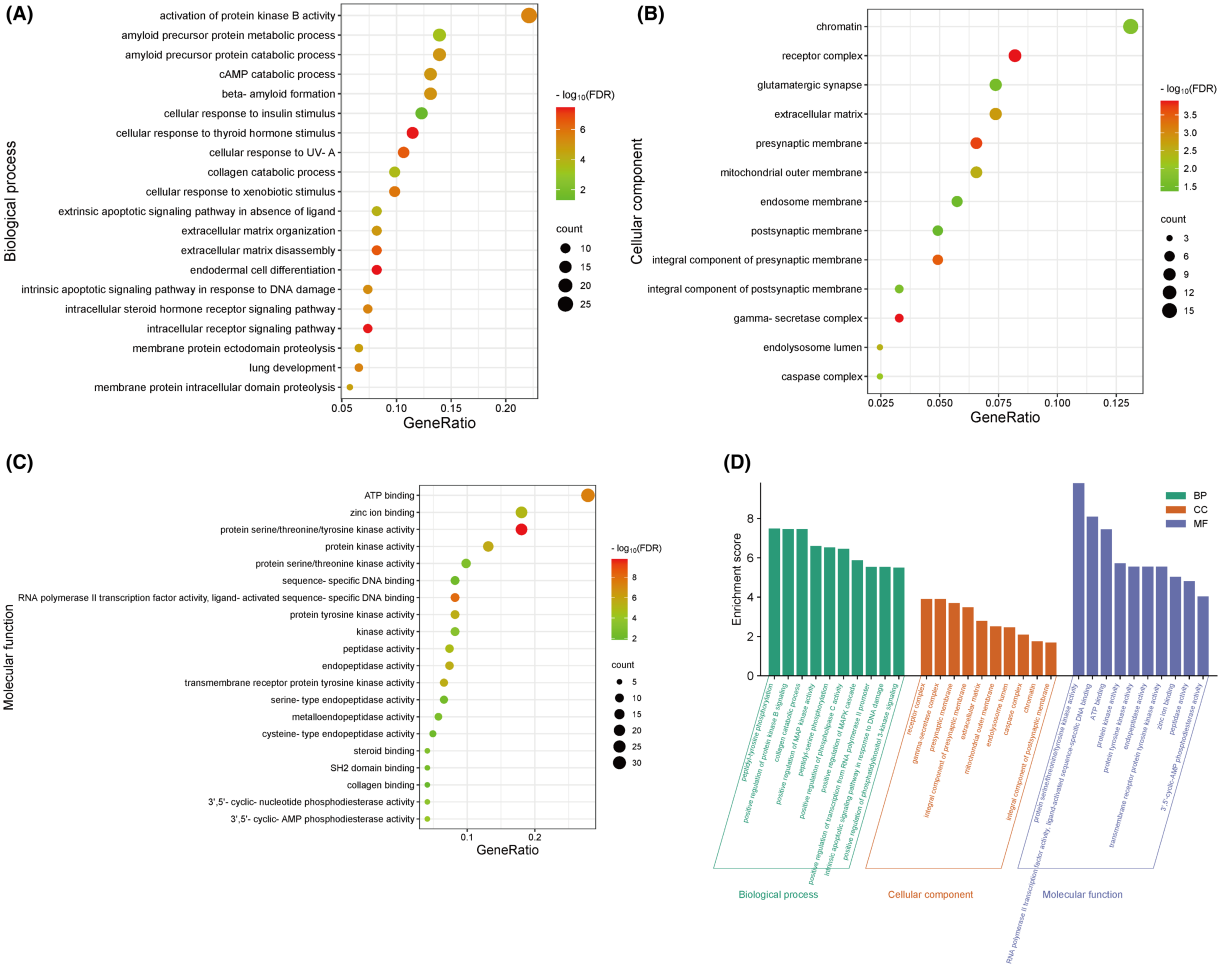
Go functional enrichment analysis of potential therapeutic targets of SMI in the treatment of sepsis. (A) Bubble chart of the top 20 BP based on gene counts. (B) Bubble chart of CC. (C) Bubble chart of the top 20 MF based on gene counts. (D) Three in one bar chart of the top 10 items in BP, CC, and MF based on FDR values.

We conducted GO functional enrichment analysis on 122 potential therapeutic targets using the DAVID database, obtaining a total of 111 entries, including 68 Biological Process (BP) terms, 13 Cellular Component (CC) terms, and 30 Molecular Function (MF) terms (Tables [Link], [Link], [Link]). Based on gene counts, we selected the top 20 entries for BP and MF, and 13 entries for CC to create bubble charts (Figure 4A–C). Additionally, based on the false discovery rate (FDR) values, we chose the top 10 entries for BP, CC, and MF to generate a combined bar chart (Figure 4D). In terms of BP, the analysis mainly involves positive regulation of RNA polymerase II promoter transcription, positive regulation of gene expression, positive regulation of DNA‐templated transcription, protein phosphorylation, and protein hydrolysis. Regarding CC, the analysis mainly covers chromatin, receptor complexes, extracellular matrix, glutamatergic synapse, and presynaptic membrane. For MF, the analysis primarily includes ATP binding, serine/threonine/tyrosine kinase activity, RNA polymerase II transcription factor activity, sequence‐specific DNA binding, and endopeptidase activity.

### 3.7 KEGG functional enrichment analysis

**FIGURE 5:**
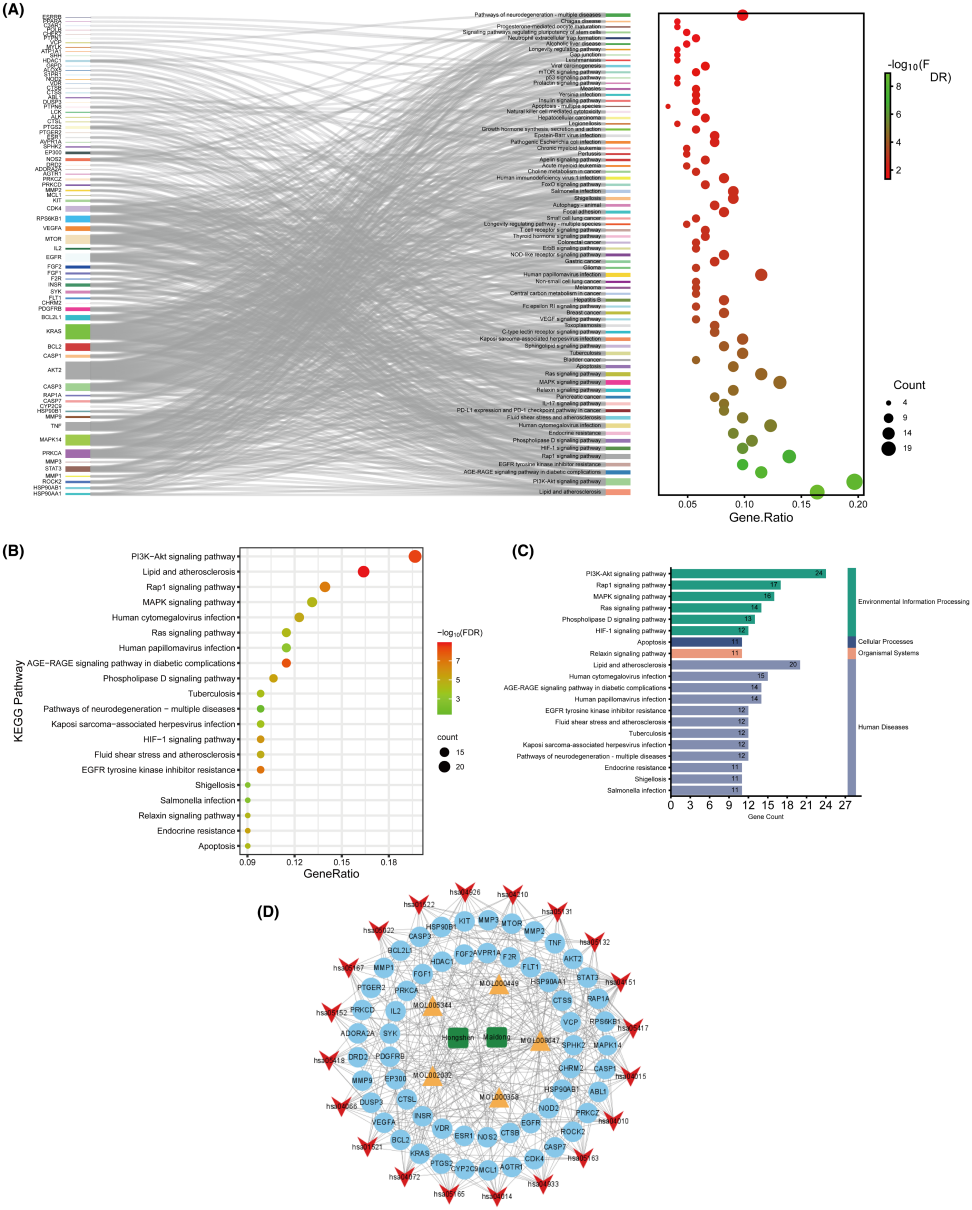
KEGG functional enrichment analysis of potential therapeutic targets of SMI in the treatment of sepsis and network construction of important pathways. (A) Network diagram of sankey dot pathway enrichment of SMI in the treatment of sepsis. (B) Bubble chart of the top 20 pathways based on gene counts. (C) Classification of the top 20 pathways according to gene counts. (D) Network diagram of SMI‐core active ingredients‐targets‐important pathways. Dark green represents herbs, yellow represents core active ingredients, light blue represents targets, and red represents important pathways.

A KEGG pathway analysis was conducted on 122 potential therapeutic targets, revealing that 74 pathways are associated with the mechanism of SMI in the treatment of sepsis (Figure 5A and Table S9). Based on gene counts, the top 20 pathways were selected to create bubble charts and were classified. Simultaneously, a herrbs‐active ingredientst‐targets‐pathways network diagram was constructed using Cytoscape 3.9.1 software, which consists of 85 nodes and 349 edges (Figure 5B–D and Table 3). The results indicated a high enrichment of the PI3K‐AKT signalling pathway, RAP1 signalling pathway, and MAPK signalling pathway, suggesting that SMI may exert its therapeutic effects on sepsis by regulating these pathways (Figure S3).

**TABLE 3:** KEGG enrichment results of potential targets.

ID	Term	Count	Gene Ratio(%)	Genes	FDR
hsa04151	PI3K‐Akt signalling pathway	24	19.67	PDGFRB, CHRM2, HSP90AA1, FLT1, HSP90AB1, SYK, INSR, F2R, PRKCA, FGF1, FGF2, EGFR, IL2, MTOR, HSP90B1, VEGFA, RPS6KB1, CDK4, AKT2, KIT, BCL2, KRAS, MCL1, BCL2L1	4.03E‐09
hsa05417	Lipid and atherosclerosis	20	16.39	HSP90AA1, HSP90AB1, ROCK2, MMP1, STAT3, MMP3, PRKCA, MAPK14, TNF, MMP9, HSP90B1, CYP2C9, CASP7, RAP1A, CASP3, AKT2, CASP1, BCL2, KRAS, BCL2L1	1.13E‐09
hsa04015	Rap1 signalling pathway	17	13.93	PDGFRB, FLT1, INSR, F2R, PRKCA, FGF1, MAPK14, FGF2, PRKCZ, EGFR, VEGFA, RAP1A, ADORA2A, AKT2, KIT, KRAS, DRD2	1.57E‐07
hsa04010	MAPK signalling pathway	16	13.11	PDGFRB, FLT1, DUSP3, INSR, PRKCA, FGF1, MAPK14, FGF2, TNF, EGFR, VEGFA, RAP1A, CASP3, AKT2, KIT, KRAS	3.79E‐05
hsa05163	Human cytomegalovirus infection	15	12.30	ROCK2, PTGER2, STAT3, PRKCA, PTGS2, MAPK14, TNF, EGFR, MTOR, VEGFA, RPS6KB1, CDK4, CASP3, AKT2, KRAS	8.00E‐06
hsa04933	AGE‐RAGE signalling pathway in diabetic complications	14	11.48	MMP2, PRKCD, STAT3, PRKCA, MAPK14, TNF, PRKCZ, VEGFA, CDK4, CASP3, AKT2, BCL2, AGTR1, KRAS	9.84E‐09
hsa04014	Ras signalling pathway	14	11.48	PDGFRB, FLT1, INSR, PRKCA, FGF1, FGF2, EGFR, VEGFA, RAP1A, AKT2, KIT, ABL1, KRAS, BCL2L1	4.78E‐05
hsa05165	Human papillomavirus infection	14	11.48	PDGFRB, HDAC1, PTGS2, TNF, PRKCZ, EGFR, MTOR, VEGFA, RPS6KB1, CDK4, CASP3, AKT2, EP300, KRAS	8.80E‐04
hsa04072	Phospholipase D signalling pathway	13	10.66	PDGFRB, SYK, SPHK2, INSR, F2R, PRKCA, AVPR1A, EGFR, MTOR, AKT2, KIT, AGTR1, KRAS	3.93E‐06
hsa01521	EGFR tyrosine kinase inhibitor resistance	12	9.84	PDGFRB, RPS6KB1, AKT2, STAT3, BCL2, KRAS, PRKCA, FGF2, EGFR, MTOR, BCL2L1, VEGFA	8.05E‐08
hsa04066	HIF‐1 signalling pathway	12	9.84	FLT1, RPS6KB1, NOS2, AKT2, INSR, STAT3, BCL2, EP300, PRKCA, EGFR, MTOR, VEGFA	1.44E‐06
hsa05418	Fluid shear stress and atherosclerosis	12	9.84	HSP90AA1, HSP90AB1, CTSL, AKT2, MMP2, BCL2, MAPK14, TNF, PRKCZ, MMP9, HSP90B1, VEGFA	1.13E‐05
hsa05152	Tuberculosis	12	9.84	SYK, NOS2, SPHK2, VDR, AKT2, CASP3, BCL2, EP300, NOD2, MAPK14, TNF, CTSS	8.22E‐05
hsa05167	Kaposi sarcoma‐associated herpesvirus infection	12	9.84	SYK, CDK4, AKT2, CASP3, STAT3, EP300, KRAS, MAPK14, PTGS2, FGF2, MTOR, VEGFA	1.54E‐04
hsa05022	Pathways of neurodegeneration—multiple diseases	12	9.84	VCP, CASP7, NOS2, CASP3, BCL2, KRAS, PRKCA, MAPK14, PTGS2, TNF, MTOR, BCL2L1	0.044268047
hsa01522	Endocrine resistance	11	9.02	RPS6KB1, CDK4, AKT2, MMP2, BCL2, KRAS, MAPK14, ESR1, MMP9, EGFR, MTOR	4.25E‐06
hsa04926	Relaxin signalling pathway	11	9.02	NOS2, MMP1, AKT2, MMP2, KRAS, PRKCA, MAPK14, PRKCZ, MMP9, EGFR, VEGFA	3.40E‐05
hsa04210	Apoptosis	11	9.02	CASP7, CTSL, AKT2, CASP3, BCL2, KRAS, TNF, CTSS, CTSB, BCL2L1, MCL1	4.78E‐05
hsa05131	Shigellosis	11	9.02	RPS6KB1, ROCK2, AKT2, PRKCD, CASP1, BCL2, MAPK14, TNF, EGFR, MTOR, BCL2L1	0.002405411
hsa05132	Salmonella infection	11	9.02	CASP7, HSP90AA1, HSP90AB1, ROCK2, AKT2, CASP3, CASP1, BCL2, MAPK14, TNF, HSP90B1	0.002499679

### 3.8 Molecular Docking Visualization

**FIGURE 6:**
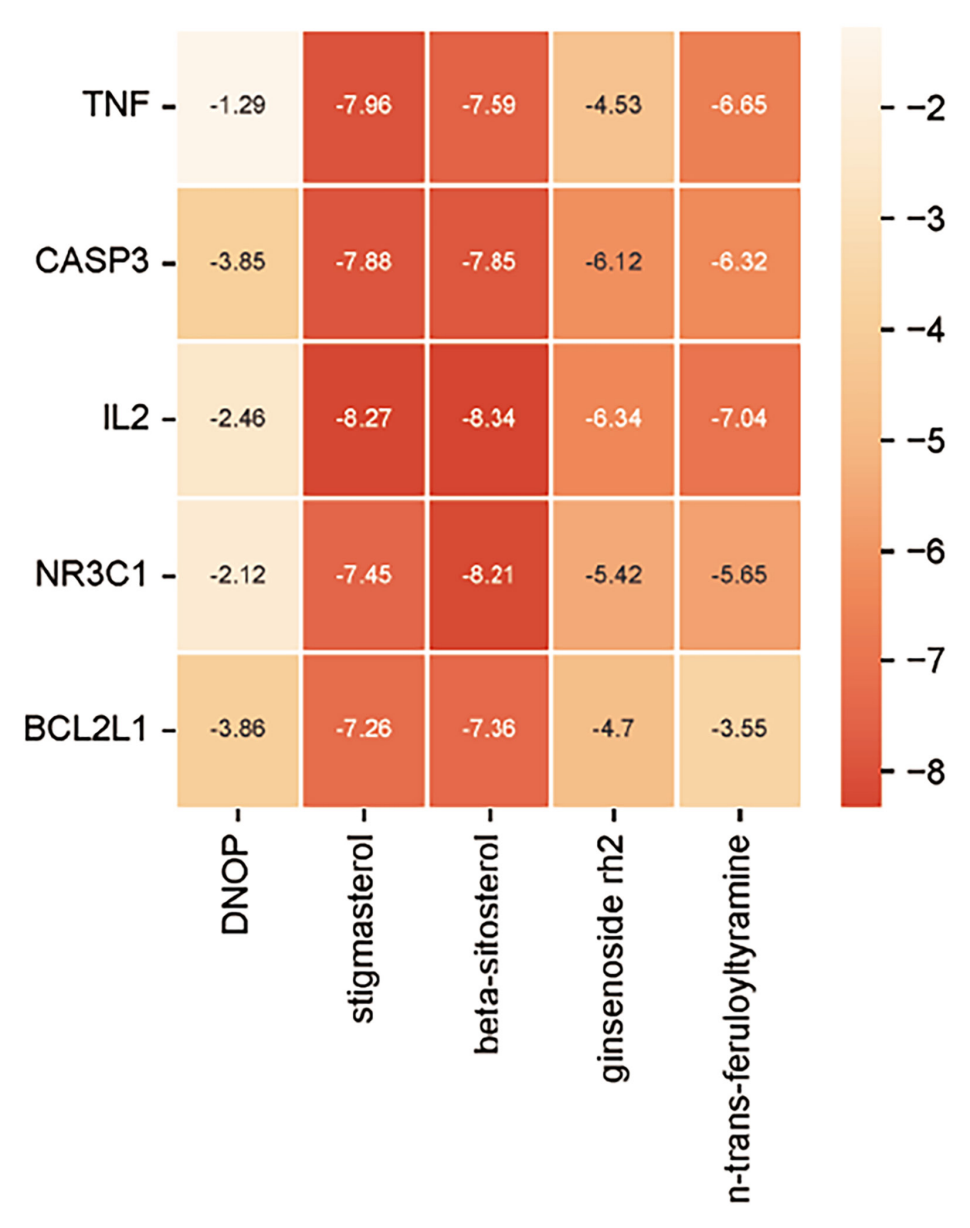
Heat map of molecular docking binding energy. Binding energy of 5 core active ingredients (n‐trans‐feruloyltyramine, DNOP, beta‐sitosterol, stigmasterol, and ginsenoside rh2) and 5 key targets (TNF, CASP3, IL2, NR3C1, and BCL2L1) docking separately.

**FIGURE 7:**
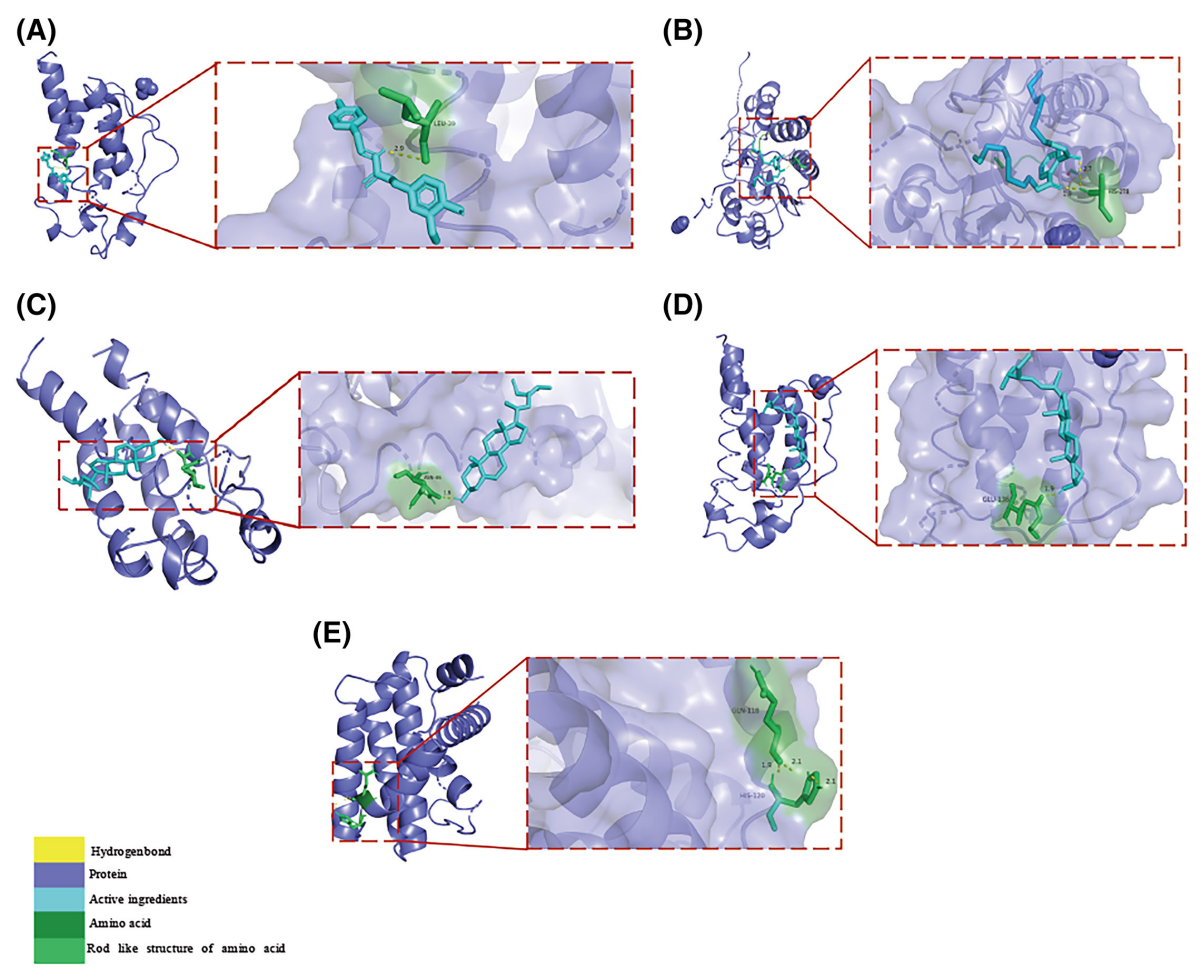
Docking diagram between core active ingredients and key targets. (A) n‐trans‐feruloyltyramine‐IL2. (B) DNOP‐CASP3. (C) beta‐sitosterol‐IL2. (D) stigmasterol‐IL2. (E) ginsenoside rh2‐BCL2L1.

Based on the network analysis of herbs‐active ingredients‐targets, the top 5 core active ingredients according to Degree value were selected to dock with key targets TNF, CASP3, IL2, NR3C1, and BCL2L1. The results showed that stigmasterol had the lowest binding energy with IL2 at −8.34 kcal/mol, while DNOP had the highest binding energy with TNF at −1.29 kcal/mol. However, both energies were lower than −1.2 kcal/mol, indicating satisfactory docking results (Figure 6 and Table 4). In addition, we utilized PyMOL 2.4.1 software to visualize the results with the lowest binding energy and hydrogen bonding (Figure 7).

**TABLE 4:** Details of targets and active ingredients for molecular docking.

Target	PDB ID	Center Coordinates	Compounds Mol ID	PubChem ID	Compounds Name
TNF	5UUI	45.263, 52.821, 13.638	MOL008647	5,280,537	n‐trans‐feruloyltyramine
CASP3	5IBP	27.53, 19.219, 107.059	MOL002032	8346	DNOP
IL2	7M2G	−24.657, −9.194, 0.783	MOL000358	222,284	Beta‐sitosterol
NR3C1	5G5W	6.114, −34.53, −2.831	MOL000449	5,280,794	Stigmasterol
BCL2L1	6GL8	4.919, 0.642, 10.413	MOL005344	119,307	Ginsenoside rh2

## 4 DISCUSSION

Sepsis is one of the main causes of death in the ICU, and its occurrence is related to various factors such as the patient's response to the infectious pathogen, host, and pathogen.^1^ As a major challenge in the field of critical care medicine, the mechanism of sepsis is complex and variable. The conventional treatments in Western medicine have limited effectiveness and inevitably lead to serious side effects.^28^ In recent years, traditional Chinese medicine has shown good efficacy in the treatment of sepsis, with fewer adverse reactions.^29^ In order to clarify the mechanism of SMI in the treatment of sepsis, in this study, we used network pharmacology methods to screen the targets of SMI and combined bioinformatics analysis methods to identify the disease genes of sepsis. Through Venn analysis, we clarified the potential therapeutic targets of SMI in the treatment of sepsis and conducted PPI network construction, functional enrichment analysis, and molecular docking on these targets.

By screening the active ingredients of SMI, constructing a network diagram of herbs‐active ingredients‐targets, it was determined that n‐trans‐feruloyltyramine, DNOP, beta‐sitosterol, stigmasterol, and ginsenoside rh2 are the core active ingredients. Among them, n‐trans‐feruloyltyramine (FLA) is an active phenylpropane compound with various pharmacological activities such as antioxidant, antibacterial, and anticancer properties.^30^ Studies have found that FLA can regulate the AP‐1 and JNK signalling pathways to reduce the mRNA expression levels of inducible nitric oxide synthase (iNOS) and cyclooxygenase‐2 (COX‐2) in vitro, thereby inhibiting the production of nitric oxide (NO) and prostaglandin E2 (PGE2), and alleviate reactive oxygen species(ROS)‐induced oxidative damage by improving cell viability, restoring cell morphology, and maintaining mitochondrial integrity.^31^, ^32^ DNOP is an ester compound derived from the esterification reaction of benzophenone and n‐octanol. Research has found that it can induce oxidative stress, block the NF‐E2‐related factor 2 (Nrf2) pathway, inhibit mitochondrial biogenesis, and cause excessive autophagy and death of liver cells.^33^ Beta‐sitosterol and stigmasterol are important bioorganic compounds that have been proven to be associated with inflammatory responses.^34^ In a study using a cecal ligation and puncture model of septic lung injury, beta‐sitosterol was found not only to inhibit the nuclear factor kappa‐B(NF‐κB) signalling cascade, reduce the production of inflammatory factors, lower lung tissue permeability, and alleviate lung tissue lesion severity, but also to enhance the function of the septic lung epithelial cell barrier by promoting the expression of claudin‐4 and claudin‐5.^35^ Animal experiments have shown that beta‐sitosterol can significantly suppress the inflammatory response by inhibiting the expression levels of tumour necrosis factor‐α(TNF‐α) and NF‐κBi through the inhibition of the NF‐κB pathway, as well as reduce the levels of AST and ALT in the serum while increasing the GSH content in the liver, thereby alleviating the liver oxidative stress response and preventing organ dysfunction associated with sepsis.^36^ Stigmasterol can counteract the inflammatory response induced by Aβ42 oligomers through the activation of the AMP‐activated protein kinase(AMPK)/NF‐κB and AMPK/NOD‐like receptor thermal protein domain associated protein 3(NLRP3) pathways, mediate the secretion of pro‐inflammatory cytokines, and inhibit the polarization of cells towards the M1 type.^37^ There are also studies suggesting that Stigmasterol can improve mitochondrial autophagic dysfunction (primarily achieved by reducing mitochondrial/lysosomal fusion and the ratio of LC3‐II/LC3‐I), decrease ROS production, restore mitochondrial membrane potential, thereby inhibiting cell death; in addition, it can promote CDK5 degradation and AKT phosphorylation, thereby suppressing the expression of CDK5, p35, and p25.^38^ Ginsenoside rh2, one of the bioactive components of ginsenosides, exhibits various pharmacological effects such as anti‐inflammatory, antioxidant, increasing non‐specific resistance and specific immune response.^39^ Studies have found that ginsenoside rh2 can regulate the expression of IL1B, ALOX5, and BCL2, exerting antibacterial effects and thereby improving the survival rate of septic patients.^40^ In vitro experiments have shown that ginsenoside rh2 can suppress the production of NO, TNF‐α, interleukin‐6 (IL‐6), IL‐1β, COX‐2, and iNOS by regulating the TGF‐β1/Smad pathway (increasing TGF‐β1 expression and reducing Smad expression), thereby alleviating LPS‐induced inflammation.^41^

Through the construction and analysis of the PPI network, 28 key targets have been identified, and several of these have been proven to be associated with the occurrence and development of sepsis. For example: in a septic mouse model created by cecal ligation and puncture(CLP), the levels of TNF‐α and IL‐2 sharply decreased after 12 h, leading to an increase in the survival rate of septic mice^42^; STAT3 in the mitochondria can induce NF‐κB nuclear localization, exacerbating LPS‐induced sepsis, while also increasing the dependence of metabolism on fatty acid oxidation^43^; the expression levels of cell surface EGFR increase in LPS‐induced macrophage inflammation, not only regulating the M1/M2 polarization phenotype transformation of macrophages but also impacting sepsis‐induced multi‐organ damage through metabolic reprogramming.^44^ Additionally, the roles of targets such as CASP1/3, BCL2, and MTOR in sepsis have been discussed.^45^, ^46^, ^47^, ^48^ Through the BioGPS database, we found that 40 tissues/cells can specifically overexpress potential therapeutic targets. Based on this, we speculate that SMI may alleviate late‐stage organ damage in sepsis through the potential therapeutic targets mentioned above.

Through enrichment analysis using the DAVID database, it was found that the mechanism of SMI in the treatment of sepsis is complex, mainly involving aspects such as anti‐inflammation, anti‐oxidative stress, and immune regulation. The PI3K/Akt pathway is a classical signalling pathway that plays an important role in regulating intracellular signal transduction, as well as cell growth, proliferation, autophagy, and apoptosis.^49^ When activated, it can exert anti‐inflammatory and anti‐oxidative functions.^50^ Studies have found that the activated PI3K/Akt signalling pathway can improve mitochondrial quality and alleviate LPS‐induced acute lung injury in rats.^51^ There is also research indicating that the activation of the PI3K/Akt pathway can enhance autophagy, inhibit cardiomyocyte apoptosis, and thereby improve myocardial dysfunction in septic rats.^52^ Additionally, because the PI3K/Akt pathway plays an important role in the normal immune function of neutrophils,^53^ the oxidative burst and phagocytic activity of neutrophils in septic mouse models can be significantly reduced when the PI3K/Akt pathway is inhibited.^54^ The RAP1 signalling pathway is closely related to inflammatory responses.^55^ Research has found that Rap1 can directly interact with PI3K through its effector domain 50, limiting Akt recruitment to TNFR2 signalling complexes, thereby inhibiting the transmission of pro‐inflammatory signals, while the loss of Rap1 promotes the transmission of pro‐inflammatory cytokine signals and NF‐κB activation.^56^ The MAPK signalling pathway can be activated by factors such as LPS and TNF‐A.^57^ The activated MAPK signalling pathway can promote the nuclear translocation of phosphorylated ERK, P38, and JNK, induce the expression of related inflammatory factors, and thus promote the inflammatory response.^58^ Research has also found that MAPK is an upstream activating factor of NF‐κB, playing an important role in regulating the activation of NF‐κB, which is associated with inflammatory responses and cell apoptosis during sepsis.^59^ Therefore, some scholars believe that p38 MAPK can promote the activation and nuclear translocation of NF‐κB, thereby promoting the expression of pro‐inflammatory factors such as IL‐1β and TNF‐α.^60^

Finally, we used molecular docking technology to validate the results of network pharmacology. We docked five core active ingredients (n‐trans‐feruloyltyramine, DNOP, beta‐sitosterol, stigmasterol, ginsenoside Rh2) with five key targets (TNF, CASP3, IL2, NR3C1, BCL2L1), respectively. The binding energies ranged from −8.34 to −1.29 kcal/mol, indicating a stable binding capability between the core active ingredients and the key targets. Among them, IL2 showed relatively good binding affinity to the core active ingredients, suggesting its crucial role in the treatment of sepsis with SMI, but further experimental validation is still needed.

It should be pointed out that there are still some limitations in this study. We only conducted a comprehensive analysis of the active ingredients of SMI, disease targets, and mechanisms from the perspective of online public databases. Therefore, the reliability of the results is closely related to the quality of the data uploaded in the database. Therefore, further basic research is still needed to verify the mechanism of SMI in the treatment of sepsis.

## 5 CONCLUSIONS

In conclusion, for the first time in this study, the mechanism of SMI in the treatment of sepsis was systematically explored using network pharmacology, bioinformatics, and molecular docking technologies. Our research results indicate that the core active ingredients of SMI are n‐trans‐feruloyltyramine, DNOP, beta‐sitosterol, stigmasterol, and ginsenoside rh2. These core active ingredients regulate key targets such as TNF, CASP3, and IL2 through signalling pathways such as PI3K‐AKT, RAP1, and MAPK, exerting anti‐inflammatory, anti‐oxidative stress, and immune‐regulating effects. This has certain reference value for the clinical treatment and basic research of sepsis.

## AUTHOR CONTRIBUTIONS

**Mengxia Yang:** Conceptualization (lead); writing – original draft (lead); writing – review and editing (lead). **Tengfei Chen:** Data curation (equal); formal analysis (equal); methodology (equal); software (equal). **Xiaolong Xu:** Investigation (equal); project administration (equal); supervision (equal). **Yue Xu:** Supervision (equal); validation (equal); visualization (equal). **Qingquan Liu:** Resources (equal); supervision (equal).

## FUNDING INFORMATION

The present study was supported by the National Natural Science Foundation of China (grant number 82474428), National Multidisciplinary Innovation Team Project of Traditional Chinese Medicine (grant no. ZYYCXTD‐D‐202201) and Key Discipline Construction Project of Traditional Chinese Medicine authorized by the State Administration of Traditional Chinese Medicine (grant no. zyyzdxk‐2023001).

## CONFLICT OF INTEREST STATEMENT

The authors confirm that there are no conflicts of interest.

## PATIENT CONSENT FOR PUBLICATION

Not applicable.

## Supporting information


Figure S1.

Figure S2.

Figure S3.

Table S1.

Table S2.

Table S3.

Table S4.

Table S5.

Table S6.

Table S7.

Table S8.

Table S9.

## Data Availability

The data that supports the findings of this study are available in the supplementary material of this article.
