# Supplementary figures and images for: Study on the mechanism of Shenmai injection in the treatment of sepsis
Source: J Cell Mol Med. 2024 Nov 25;28(22):e70201. doi: 10.1111/jcmm.70201 (PMC11586680; doi:10.1111/jcmm.70201)

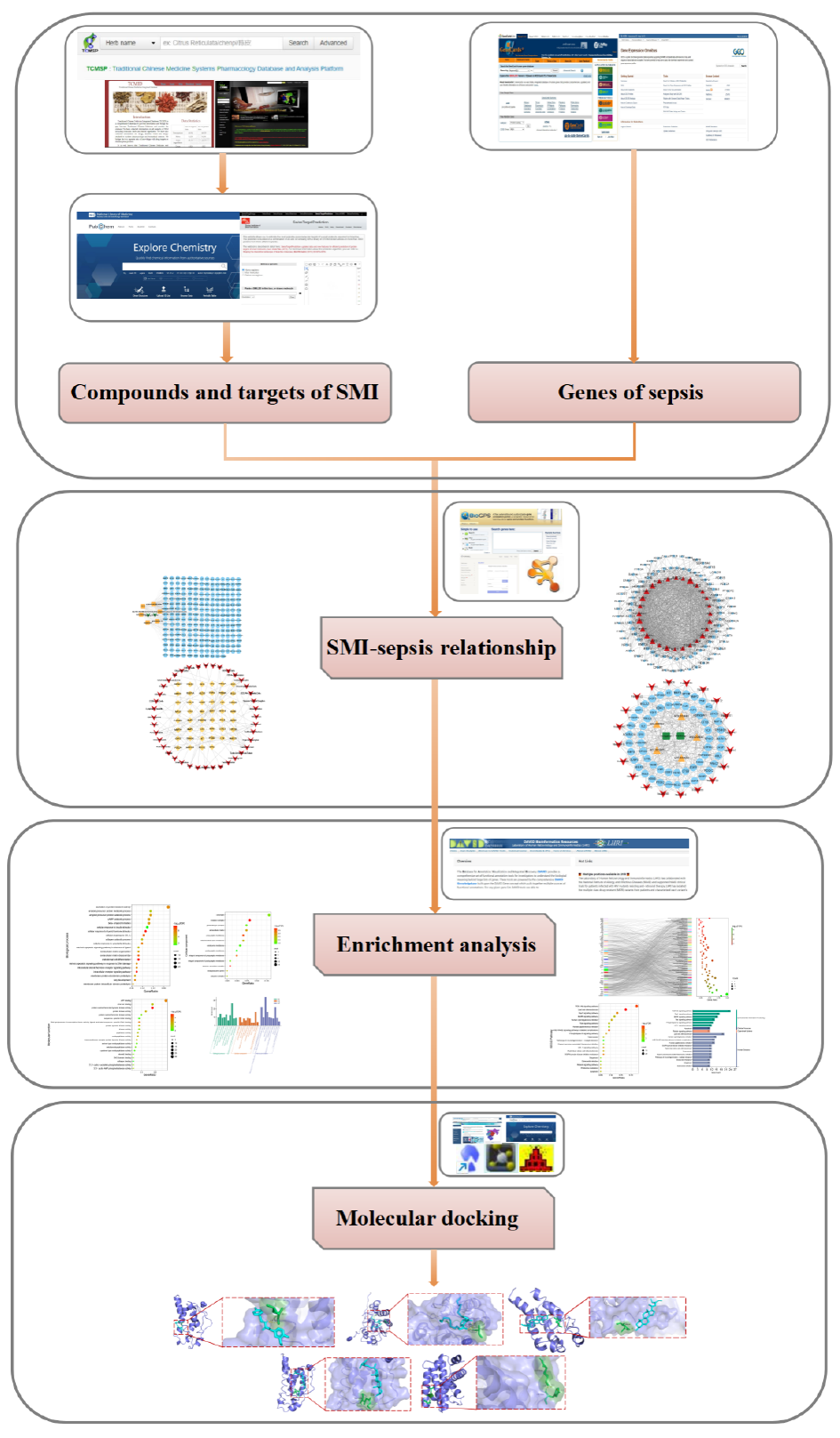

Supplement: Supplementary file 1 — Figure S1. [file JCMM-28-e70201-s012.tif]

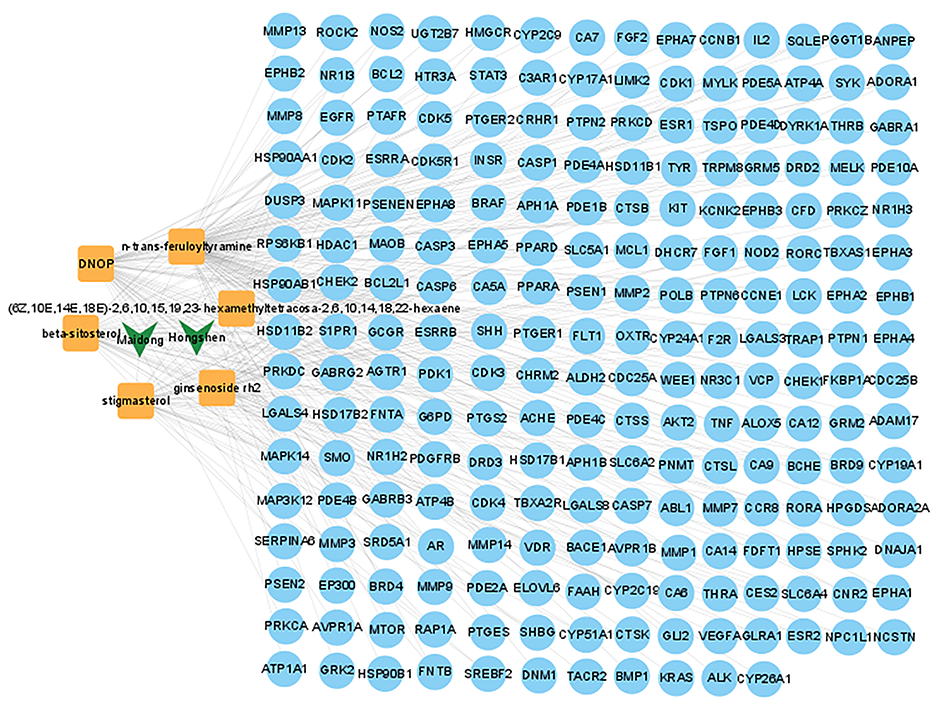

Supplement: Supplementary file 2 — Figure S2. [file JCMM-28-e70201-s011.tif]

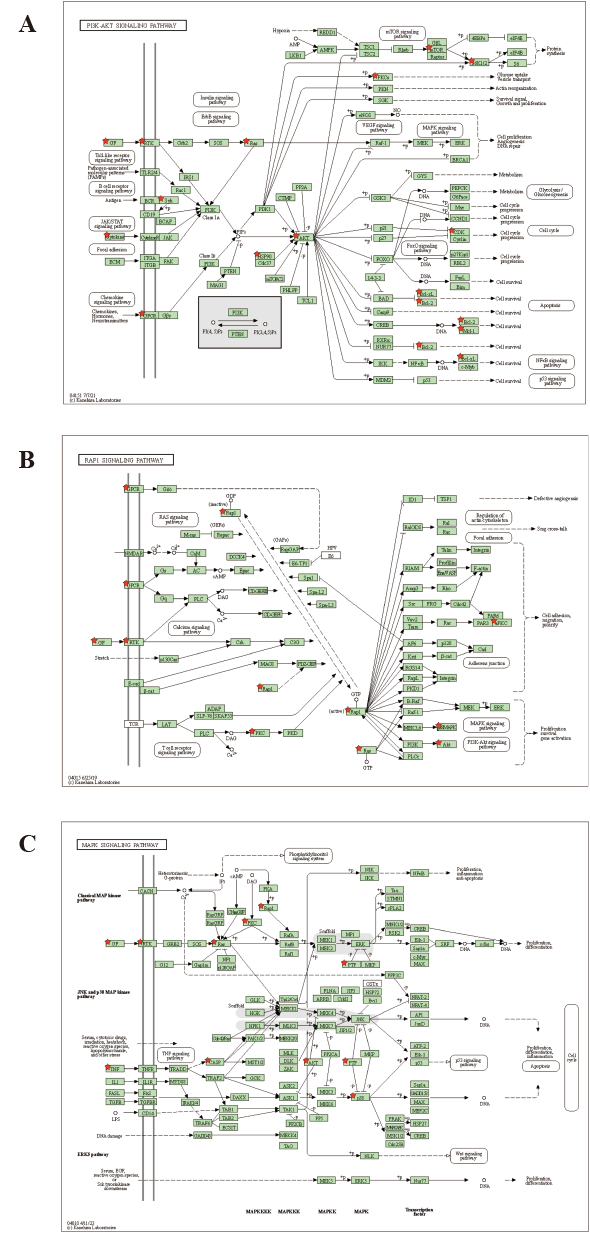

Supplement: Supplementary file 3 — Figure S3. [file JCMM-28-e70201-s005.tif]
