# Supplementary material for: Study on the mechanism of Shenmai injection in the treatment of sepsis
Source: J Cell Mol Med. 2024 Nov 25;28(22):e70201. doi: 10.1111/jcmm.70201 (PMC11586680; doi:10.1111/jcmm.70201)
Supplement: Supplementary file 4 — Table S1. [file JCMM-28-e70201-s001.docx]

**Supplementary Table 1 Basic information of 221 targets of SMI active ingredients**

| NO. | Target Symbol | Molecule Name | OB(%) | DL |
| --- | --- | --- | --- | --- |
| 1 | ABL1 | n-trans-feruloyltyramine | 86.71 | 0.26 |
| 1 | ABL1 | DNOP | 40.59 | 0.4 |
| 2 | ACHE | n-trans-feruloyltyramine | 86.71 | 0.26 |
| 2 | ACHE | stigmasterol | 43.83 | 0.76 |
| 2 | ACHE | beta-sitosterol | 36.91 | 0.75 |
| 3 | ADAM17 | n-trans-feruloyltyramine | 86.71 | 0.26 |
| 4 | ADORA1 | n-trans-feruloyltyramine | 86.71 | 0.26 |
| 4 | ADORA1 | DNOP | 40.59 | 0.4 |
| 4 | ADORA1 | ginsenoside rh2 | 36.32 | 0.56 |
| 5 | ADORA2A | n-trans-feruloyltyramine | 86.71 | 0.26 |
| 6 | AGTR1 | n-trans-feruloyltyramine | 86.71 | 0.26 |
| 7 | AKT2 | n-trans-feruloyltyramine | 86.71 | 0.26 |
| 8 | ALDH2 | n-trans-feruloyltyramine | 86.71 | 0.26 |
| 9 | ALK | n-trans-feruloyltyramine | 86.71 | 0.26 |
| 10 | ALOX5 | n-trans-feruloyltyramine | 86.71 | 0.26 |
| 11 | ANPEP | n-trans-feruloyltyramine | 86.71 | 0.26 |
| 12 | APH1A | ginsenoside rh2 | 36.32 | 0.56 |
| 13 | APH1B | ginsenoside rh2 | 36.32 | 0.56 |
| 14 | AR | stigmasterol | 43.83 | 0.76 |
| 14 | AR | beta-sitosterol | 36.91 | 0.75 |
| 15 | ATP1A1 | ginsenoside rh2 | 36.32 | 0.56 |
| 16 | ATP4A | n-trans-feruloyltyramine | 86.71 | 0.26 |
| 17 | ATP4B | n-trans-feruloyltyramine | 86.71 | 0.26 |
| 18 | AVPR1A | DNOP | 40.59 | 0.4 |
| 19 | AVPR1B | DNOP | 40.59 | 0.4 |
| 20 | BACE1 | (6Z,10E,14E,18E)-2,6,10,15,19,23-hexamethyltetracosa-2,6,10,14,18,22-hexaene | 33.55 | 0.42 |
| 21 | BCHE | n-trans-feruloyltyramine | 86.71 | 0.26 |
| 21 | BCHE | stigmasterol | 43.83 | 0.76 |
| 21 | BCHE | beta-sitosterol | 36.91 | 0.75 |
| 22 | BCL2 | n-trans-feruloyltyramine | 86.71 | 0.26 |
| 23 | BCL2L1 | n-trans-feruloyltyramine | 86.71 | 0.26 |
| 23 | BCL2L1 | ginsenoside rh2 | 36.32 | 0.56 |
| 24 | BMP1 | n-trans-feruloyltyramine | 86.71 | 0.26 |
| 25 | BRAF | n-trans-feruloyltyramine | 86.71 | 0.26 |
| 26 | BRD4 | n-trans-feruloyltyramine | 86.71 | 0.26 |
| 27 | BRD9 | n-trans-feruloyltyramine | 86.71 | 0.26 |
| 28 | C3AR1 | DNOP | 40.59 | 0.4 |
| 29 | CA12 | n-trans-feruloyltyramine | 86.71 | 0.26 |
| 30 | CA14 | n-trans-feruloyltyramine | 86.71 | 0.26 |
| 31 | CA5A | n-trans-feruloyltyramine | 86.71 | 0.26 |
| 32 | CA6 | n-trans-feruloyltyramine | 86.71 | 0.26 |
| 33 | CA7 | n-trans-feruloyltyramine | 86.71 | 0.26 |
| 34 | CA9 | n-trans-feruloyltyramine | 86.71 | 0.26 |
| 35 | CASP1 | DNOP | 40.59 | 0.4 |
| 36 | CASP3 | DNOP | 40.59 | 0.4 |
| 37 | CASP6 | DNOP | 40.59 | 0.4 |
| 38 | CASP7 | DNOP | 40.59 | 0.4 |
| 39 | CCNB1 | n-trans-feruloyltyramine | 86.71 | 0.26 |
| 40 | CCNE1 | n-trans-feruloyltyramine | 86.71 | 0.26 |
| 41 | CCR8 | DNOP | 40.59 | 0.4 |
| 42 | CDC25A | stigmasterol | 43.83 | 0.76 |
| 42 | CDC25A | beta-sitosterol | 36.91 | 0.75 |
| 43 | CDC25B | beta-sitosterol | 36.91 | 0.75 |
| 44 | CDK1 | n-trans-feruloyltyramine | 86.71 | 0.26 |
| 45 | CDK2 | n-trans-feruloyltyramine | 86.71 | 0.26 |
| 46 | CDK3 | n-trans-feruloyltyramine | 86.71 | 0.26 |
| 47 | CDK4 | n-trans-feruloyltyramine | 86.71 | 0.26 |
| 48 | CDK5 | n-trans-feruloyltyramine | 86.71 | 0.26 |
| 48 | CDK5 | DNOP | 40.59 | 0.4 |
| 49 | CDK5R1 | n-trans-feruloyltyramine | 86.71 | 0.26 |
| 49 | CDK5R1 | DNOP | 40.59 | 0.4 |
| 50 | CES2 | stigmasterol | 43.83 | 0.76 |
| 50 | CES2 | beta-sitosterol | 36.91 | 0.75 |
| 51 | CFD | n-trans-feruloyltyramine | 86.71 | 0.26 |
| 52 | CHEK1 | n-trans-feruloyltyramine | 86.71 | 0.26 |
| 53 | CHEK2 | n-trans-feruloyltyramine | 86.71 | 0.26 |
| 54 | CHRM2 | stigmasterol | 43.83 | 0.76 |
| 54 | CHRM2 | beta-sitosterol | 36.91 | 0.75 |
| 55 | CNR2 | n-trans-feruloyltyramine | 86.71 | 0.26 |
| 55 | CNR2 | (6Z,10E,14E,18E)-2,6,10,15,19,23-hexamethyltetracosa-2,6,10,14,18,22-hexaene | 33.55 | 0.42 |
| 56 | CRHR1 | DNOP | 40.59 | 0.4 |
| 57 | CTSB | DNOP | 40.59 | 0.4 |
| 58 | CTSK | DNOP | 40.59 | 0.4 |
| 59 | CTSL | n-trans-feruloyltyramine | 86.71 | 0.26 |
| 59 | CTSL | DNOP | 40.59 | 0.4 |
| 60 | CTSS | DNOP | 40.59 | 0.4 |
| 61 | CYP17A1 | stigmasterol | 43.83 | 0.76 |
| 61 | CYP17A1 | beta-sitosterol | 36.91 | 0.75 |
| 62 | CYP19A1 | stigmasterol | 43.83 | 0.76 |
| 62 | CYP19A1 | beta-sitosterol | 36.91 | 0.75 |
| 63 | CYP24A1 | DNOP | 40.59 | 0.4 |
| 64 | CYP26A1 | DNOP | 40.59 | 0.4 |
| 65 | CYP2C19 | stigmasterol | 43.83 | 0.76 |
| 65 | CYP2C19 | beta-sitosterol | 36.91 | 0.75 |
| 66 | CYP2C9 | DNOP | 40.59 | 0.4 |
| 67 | CYP51A1 | stigmasterol | 43.83 | 0.76 |
| 67 | CYP51A1 | beta-sitosterol | 36.91 | 0.75 |
| 68 | DHCR7 | stigmasterol | 43.83 | 0.76 |
| 68 | DHCR7 | beta-sitosterol | 36.91 | 0.75 |
| 69 | DNAJA1 | DNOP | 40.59 | 0.4 |
| 70 | DNM1 | n-trans-feruloyltyramine | 86.71 | 0.26 |
| 71 | DRD2 | n-trans-feruloyltyramine | 86.71 | 0.26 |
| 71 | DRD2 | beta-sitosterol | 36.91 | 0.75 |
| 72 | DRD3 | n-trans-feruloyltyramine | 86.71 | 0.26 |
| 73 | DUSP3 | n-trans-feruloyltyramine | 86.71 | 0.26 |
| 74 | DYRK1A | DNOP | 40.59 | 0.4 |
| 75 | EGFR | n-trans-feruloyltyramine | 86.71 | 0.26 |
| 76 | ELOVL6 | DNOP | 40.59 | 0.4 |
| 77 | EP300 | n-trans-feruloyltyramine | 86.71 | 0.26 |
| 78 | EPHA1 | n-trans-feruloyltyramine | 86.71 | 0.26 |
| 79 | EPHA2 | n-trans-feruloyltyramine | 86.71 | 0.26 |
| 80 | EPHA3 | n-trans-feruloyltyramine | 86.71 | 0.26 |
| 81 | EPHA4 | n-trans-feruloyltyramine | 86.71 | 0.26 |
| 82 | EPHA5 | n-trans-feruloyltyramine | 86.71 | 0.26 |
| 83 | EPHA7 | n-trans-feruloyltyramine | 86.71 | 0.26 |
| 84 | EPHA8 | n-trans-feruloyltyramine | 86.71 | 0.26 |
| 85 | EPHB1 | n-trans-feruloyltyramine | 86.71 | 0.26 |
| 86 | EPHB2 | n-trans-feruloyltyramine | 86.71 | 0.26 |
| 87 | EPHB3 | n-trans-feruloyltyramine | 86.71 | 0.26 |
| 88 | ESR1 | stigmasterol | 43.83 | 0.76 |
| 88 | ESR1 | beta-sitosterol | 36.91 | 0.75 |
| 89 | ESR2 | stigmasterol | 43.83 | 0.76 |
| 89 | ESR2 | beta-sitosterol | 36.91 | 0.75 |
| 90 | ESRRA | n-trans-feruloyltyramine | 86.71 | 0.26 |
| 91 | ESRRB | n-trans-feruloyltyramine | 86.71 | 0.26 |
| 92 | F2R | DNOP | 40.59 | 0.4 |
| 93 | FAAH | DNOP | 40.59 | 0.4 |
| 94 | FDFT1 | beta-sitosterol | 36.91 | 0.75 |
| 95 | FGF1 | ginsenoside rh2 | 36.32 | 0.56 |
| 96 | FGF2 | ginsenoside rh2 | 36.32 | 0.56 |
| 97 | FKBP1A | DNOP | 40.59 | 0.4 |
| 98 | FLT1 | DNOP | 40.59 | 0.4 |
| 99 | FNTA | n-trans-feruloyltyramine | 86.71 | 0.26 |
| 99 | FNTA | DNOP | 40.59 | 0.4 |
| 100 | FNTB | n-trans-feruloyltyramine | 86.71 | 0.26 |
| 100 | FNTB | DNOP | 40.59 | 0.4 |
| 101 | G6PD | stigmasterol | 43.83 | 0.76 |
| 101 | G6PD | beta-sitosterol | 36.91 | 0.75 |
| 102 | GABRA1 | DNOP | 40.59 | 0.4 |
| 103 | GABRB3 | DNOP | 40.59 | 0.4 |
| 104 | GABRG2 | DNOP | 40.59 | 0.4 |
| 105 | GCGR | stigmasterol | 43.83 | 0.76 |
| 106 | GLI2 | n-trans-feruloyltyramine | 86.71 | 0.26 |
| 107 | GLRA1 | stigmasterol | 43.83 | 0.76 |
| 107 | GLRA1 | beta-sitosterol | 36.91 | 0.75 |
| 108 | GRK2 | n-trans-feruloyltyramine | 86.71 | 0.26 |
| 109 | GRM2 | n-trans-feruloyltyramine | 86.71 | 0.26 |
| 110 | GRM5 | DNOP | 40.59 | 0.4 |
| 111 | HDAC1 | n-trans-feruloyltyramine | 86.71 | 0.26 |
| 112 | HMGCR | stigmasterol | 43.83 | 0.76 |
| 112 | HMGCR | beta-sitosterol | 36.91 | 0.75 |
| 113 | HPGDS | n-trans-feruloyltyramine | 86.71 | 0.26 |
| 114 | HPSE | ginsenoside rh2 | 36.32 | 0.56 |
| 115 | HSD11B1 | stigmasterol | 43.83 | 0.76 |
| 115 | HSD11B1 | beta-sitosterol | 36.91 | 0.75 |
| 116 | HSD11B2 | stigmasterol | 43.83 | 0.76 |
| 116 | HSD11B2 | beta-sitosterol | 36.91 | 0.75 |
| 116 | HSD11B2 | ginsenoside rh2 | 36.32 | 0.56 |
| 117 | HSD17B1 | n-trans-feruloyltyramine | 86.71 | 0.26 |
| 118 | HSD17B2 | n-trans-feruloyltyramine | 86.71 | 0.26 |
| 119 | HSP90AA1 | n-trans-feruloyltyramine | 86.71 | 0.26 |
| 119 | HSP90AA1 | ginsenoside rh2 | 36.32 | 0.56 |
| 120 | HSP90AB1 | n-trans-feruloyltyramine | 86.71 | 0.26 |
| 121 | HSP90B1 | n-trans-feruloyltyramine | 86.71 | 0.26 |
| 122 | HTR3A | n-trans-feruloyltyramine | 86.71 | 0.26 |
| 123 | IL2 | ginsenoside rh2 | 36.32 | 0.56 |
| 124 | INSR | n-trans-feruloyltyramine | 86.71 | 0.26 |
| 125 | KCNK2 | DNOP | 40.59 | 0.4 |
| 126 | KIT | DNOP | 40.59 | 0.4 |
| 127 | KRAS | DNOP | 40.59 | 0.4 |
| 128 | LCK | DNOP | 40.59 | 0.4 |
| 129 | LGALS3 | ginsenoside rh2 | 36.32 | 0.56 |
| 130 | LGALS4 | ginsenoside rh2 | 36.32 | 0.56 |
| 131 | LGALS8 | ginsenoside rh2 | 36.32 | 0.56 |
| 132 | LIMK2 | DNOP | 40.59 | 0.4 |
| 133 | MAOB | n-trans-feruloyltyramine | 86.71 | 0.26 |
| 134 | MAP3K12 | DNOP | 40.59 | 0.4 |
| 135 | MAPK11 | DNOP | 40.59 | 0.4 |
| 136 | MAPK14 | DNOP | 40.59 | 0.4 |
| 137 | MCL1 | n-trans-feruloyltyramine | 86.71 | 0.26 |
| 138 | MELK | n-trans-feruloyltyramine | 86.71 | 0.26 |
| 139 | MMP1 | n-trans-feruloyltyramine | 86.71 | 0.26 |
| 140 | MMP13 | n-trans-feruloyltyramine | 86.71 | 0.26 |
| 141 | MMP14 | n-trans-feruloyltyramine | 86.71 | 0.26 |
| 142 | MMP2 | n-trans-feruloyltyramine | 86.71 | 0.26 |
| 143 | MMP3 | n-trans-feruloyltyramine | 86.71 | 0.26 |
| 144 | MMP7 | n-trans-feruloyltyramine | 86.71 | 0.26 |
| 145 | MMP8 | n-trans-feruloyltyramine | 86.71 | 0.26 |
| 146 | MMP9 | n-trans-feruloyltyramine | 86.71 | 0.26 |
| 147 | MTOR | n-trans-feruloyltyramine | 86.71 | 0.26 |
| 148 | MYLK | n-trans-feruloyltyramine | 86.71 | 0.26 |
| 149 | NCSTN | ginsenoside rh2 | 36.32 | 0.56 |
| 150 | NOD2 | DNOP | 40.59 | 0.4 |
| 151 | NOS2 | stigmasterol | 43.83 | 0.76 |
| 151 | NOS2 | beta-sitosterol | 36.91 | 0.75 |
| 152 | NPC1L1 | stigmasterol | 43.83 | 0.76 |
| 152 | NPC1L1 | beta-sitosterol | 36.91 | 0.75 |
| 153 | NR1H2 | stigmasterol | 43.83 | 0.76 |
| 153 | NR1H2 | beta-sitosterol | 36.91 | 0.75 |
| 154 | NR1H3 | stigmasterol | 43.83 | 0.76 |
| 154 | NR1H3 | beta-sitosterol | 36.91 | 0.75 |
| 155 | NR1I3 | stigmasterol | 43.83 | 0.76 |
| 155 | NR1I3 | beta-sitosterol | 36.91 | 0.75 |
| 156 | NR3C1 | stigmasterol | 43.83 | 0.76 |
| 156 | NR3C1 | beta-sitosterol | 36.91 | 0.75 |
| 157 | OXTR | DNOP | 40.59 | 0.4 |
| 158 | PDE10A | DNOP | 40.59 | 0.4 |
| 159 | PDE1B | DNOP | 40.59 | 0.4 |
| 160 | PDE2A | DNOP | 40.59 | 0.4 |
| 161 | PDE4A | DNOP | 40.59 | 0.4 |
| 162 | PDE4B | DNOP | 40.59 | 0.4 |
| 163 | PDE4C | n-trans-feruloyltyramine | 86.71 | 0.26 |
| 164 | PDE4D | n-trans-feruloyltyramine | 86.71 | 0.26 |
| 165 | PDE5A | DNOP | 40.59 | 0.4 |
| 166 | PDGFRB | DNOP | 40.59 | 0.4 |
| 167 | PDK1 | n-trans-feruloyltyramine | 86.71 | 0.26 |
| 168 | PGGT1B | DNOP | 40.59 | 0.4 |
| 169 | PNMT | n-trans-feruloyltyramine | 86.71 | 0.26 |
| 170 | POLB | stigmasterol | 43.83 | 0.76 |
| 170 | POLB | beta-sitosterol | 36.91 | 0.75 |
| 171 | PPARA | (6Z,10E,14E,18E)-2,6,10,15,19,23-hexamethyltetracosa-2,6,10,14,18,22-hexaene | 33.55 | 0.42 |
| 172 | PPARD | stigmasterol | 43.83 | 0.76 |
| 172 | PPARD | beta-sitosterol | 36.91 | 0.75 |
| 173 | PRKCA | DNOP | 40.59 | 0.4 |
| 174 | PRKCD | DNOP | 40.59 | 0.4 |
| 175 | PRKCZ | n-trans-feruloyltyramine | 86.71 | 0.26 |
| 176 | PRKDC | DNOP | 40.59 | 0.4 |
| 177 | PSEN1 | ginsenoside rh2 | 36.32 | 0.56 |
| 178 | PSEN2 | ginsenoside rh2 | 36.32 | 0.56 |
| 179 | PSENEN | ginsenoside rh2 | 36.32 | 0.56 |
| 180 | PTAFR | DNOP | 40.59 | 0.4 |
| 180 | PTAFR | ginsenoside rh2 | 36.32 | 0.56 |
| 181 | PTGER1 | stigmasterol | 43.83 | 0.76 |
| 181 | PTGER1 | beta-sitosterol | 36.91 | 0.75 |
| 182 | PTGER2 | stigmasterol | 43.83 | 0.76 |
| 182 | PTGER2 | beta-sitosterol | 36.91 | 0.75 |
| 183 | PTGES | DNOP | 40.59 | 0.4 |
| 183 | PTGES | (6Z,10E,14E,18E)-2,6,10,15,19,23-hexamethyltetracosa-2,6,10,14,18,22-hexaene | 33.55 | 0.42 |
| 184 | PTGS2 | n-trans-feruloyltyramine | 86.71 | 0.26 |
| 184 | PTGS2 | DNOP | 40.59 | 0.4 |
| 185 | PTPN1 | stigmasterol | 43.83 | 0.76 |
| 185 | PTPN1 | DNOP | 40.59 | 0.4 |
| 185 | PTPN1 | beta-sitosterol | 36.91 | 0.75 |
| 186 | PTPN2 | DNOP | 40.59 | 0.4 |
| 187 | PTPN6 | stigmasterol | 43.83 | 0.76 |
| 187 | PTPN6 | beta-sitosterol | 36.91 | 0.75 |
| 188 | RAP1A | DNOP | 40.59 | 0.4 |
| 189 | ROCK2 | n-trans-feruloyltyramine | 86.71 | 0.26 |
| 190 | RORA | stigmasterol | 43.83 | 0.76 |
| 190 | RORA | beta-sitosterol | 36.91 | 0.75 |
| 191 | RORC | stigmasterol | 43.83 | 0.76 |
| 191 | RORC | beta-sitosterol | 36.91 | 0.75 |
| 191 | RORC | ginsenoside rh2 | 36.32 | 0.56 |
| 192 | RPS6KB1 | n-trans-feruloyltyramine | 86.71 | 0.26 |
| 193 | S1PR1 | ginsenoside rh2 | 36.32 | 0.56 |
| 194 | SERPINA6 | stigmasterol | 43.83 | 0.76 |
| 194 | SERPINA6 | beta-sitosterol | 36.91 | 0.75 |
| 195 | SHBG | stigmasterol | 43.83 | 0.76 |
| 195 | SHBG | beta-sitosterol | 36.91 | 0.75 |
| 196 | SHH | beta-sitosterol | 36.91 | 0.75 |
| 197 | SLC5A1 | n-trans-feruloyltyramine | 86.71 | 0.26 |
| 198 | SLC6A2 | stigmasterol | 43.83 | 0.76 |
| 198 | SLC6A2 | beta-sitosterol | 36.91 | 0.75 |
| 199 | SLC6A4 | stigmasterol | 43.83 | 0.76 |
| 199 | SLC6A4 | beta-sitosterol | 36.91 | 0.75 |
| 200 | SMO | DNOP | 40.59 | 0.4 |
| 201 | SPHK2 | n-trans-feruloyltyramine | 86.71 | 0.26 |
| 202 | SQLE | stigmasterol | 43.83 | 0.76 |
| 202 | SQLE | (6Z,10E,14E,18E)-2,6,10,15,19,23-hexamethyltetracosa-2,6,10,14,18,22-hexaene | 33.55 | 0.42 |
| 202 | SQLE | beta-sitosterol | 36.91 | 0.75 |
| 203 | SRD5A1 | DNOP | 40.59 | 0.4 |
| 204 | SREBF2 | stigmasterol | 43.83 | 0.76 |
| 204 | SREBF2 | beta-sitosterol | 36.91 | 0.75 |
| 205 | STAT3 | ginsenoside rh2 | 36.32 | 0.56 |
| 206 | SYK | n-trans-feruloyltyramine | 86.71 | 0.26 |
| 206 | SYK | ginsenoside rh2 | 36.32 | 0.56 |
| 207 | TACR2 | ginsenoside rh2 | 36.32 | 0.56 |
| 208 | TBXA2R | n-trans-feruloyltyramine | 86.71 | 0.26 |
| 209 | TBXAS1 | stigmasterol | 43.83 | 0.76 |
| 210 | THRA | n-trans-feruloyltyramine | 86.71 | 0.26 |
| 211 | THRB | n-trans-feruloyltyramine | 86.71 | 0.26 |
| 212 | TNF | n-trans-feruloyltyramine | 86.71 | 0.26 |
| 213 | TRAP1 | n-trans-feruloyltyramine | 86.71 | 0.26 |
| 214 | TRPM8 | n-trans-feruloyltyramine | 86.71 | 0.26 |
| 215 | TSPO | DNOP | 40.59 | 0.4 |
| 216 | TYR | n-trans-feruloyltyramine | 86.71 | 0.26 |
| 217 | UGT2B7 | beta-sitosterol | 36.91 | 0.75 |
| 218 | VCP | n-trans-feruloyltyramine | 86.71 | 0.26 |
| 219 | VDR | stigmasterol | 43.83 | 0.76 |
| 219 | VDR | beta-sitosterol | 36.91 | 0.75 |
| 220 | VEGFA | ginsenoside rh2 | 36.32 | 0.56 |
| 221 | WEE1 | n-trans-feruloyltyramine | 86.71 | 0.26 |
