# Supplementary material for: Study on the mechanism of Shenmai injection in the treatment of sepsis
Source: J Cell Mol Med. 2024 Nov 25;28(22):e70201. doi: 10.1111/jcmm.70201 (PMC11586680; doi:10.1111/jcmm.70201)
Supplement: Supplementary file 5 — Table S2. [file JCMM-28-e70201-s002.docx]

**Supplementary Table 2 Differential genes information analyzed by GEO datasets**

| Gene.symbol | adj.P.Val | P.Value | t | B | logFC | abs logFC | Change |
| --- | --- | --- | --- | --- | --- | --- | --- |
| MYL6 | 3.45E-13 | 2.45E-16 | -11.419583 | 26.85919671 | -4.531052 | 4.531052 | Down-regulated |
| TPD52L2 | 3.01E-10 | 1.28E-12 | -9.058311 | 18.52722249 | -4.063367 | 4.063367 | Down-regulated |
| S100A6 | 2.32E-08 | 2.77E-10 | -7.641626 | 13.27848992 | -3.805621 | 3.805621 | Down-regulated |
| GSTO1 | 1.49E-14 | 2.99E-18 | -12.713161 | 31.12228973 | -3.799648 | 3.799648 | Down-regulated |
| PPP2R2A | 9.84E-06 | 4.06E-07 | -5.726304 | 6.17662847 | -3.730069 | 3.730069 | Down-regulated |
| ATP6V0E1 | 3.34E-13 | 2.08E-16 | -11.466905 | 27.01915229 | -3.67466 | 3.67466 | Down-regulated |
| ROMO1 | 7.01E-10 | 3.71E-12 | -8.776141 | 17.49212921 | -3.616589 | 3.616589 | Down-regulated |
| ABCA1 | 2.38E-07 | 4.53E-09 | -6.912412 | 10.55251635 | -3.586179 | 3.586179 | Down-regulated |
| NOP10 | 8.95E-07 | 2.27E-08 | -6.490535 | 8.98210978 | -3.492879 | 3.492879 | Down-regulated |
| SPCS3 | 1.10E-09 | 6.64E-12 | -8.621805 | 16.92331005 | -3.456433 | 3.456433 | Down-regulated |
| POMP | 3.74E-06 | 1.26E-07 | -6.038738 | 7.31526223 | -3.402958 | 3.402958 | Down-regulated |
| UBE2F | 1.08E-11 | 1.93E-14 | -10.195016 | 22.61939319 | -3.355615 | 3.355615 | Down-regulated |
| HIST1H2BK | 3.57E-10 | 1.59E-12 | -9.00077 | 18.31668551 | -3.284301 | 3.284301 | Down-regulated |
| S100A8 | 2.02E-08 | 2.37E-10 | -7.682196 | 13.43009744 | -3.274383 | 3.274383 | Down-regulated |
| NEDD8 | 4.58E-06 | 1.59E-07 | -5.976125 | 7.08600357 | -3.210151 | 3.210151 | Down-regulated |
| LILRA5 | 5.40E-04 | 6.20E-05 | -4.326526 | 1.32831834 | -3.202448 | 3.202448 | Down-regulated |
| MRPL36 | 3.42E-11 | 9.12E-14 | -9.770069 | 21.10555746 | -3.189226 | 3.189226 | Down-regulated |
| TMUB2 | 4.33E-08 | 5.91E-10 | -7.444153 | 12.54016985 | -3.186344 | 3.186344 | Down-regulated |
| SDF2 | 7.01E-13 | 6.87E-16 | -11.12592 | 25.85993696 | -3.14139 | 3.14139 | Down-regulated |
| ATP6V1E1 | 1.37E-13 | 6.09E-17 | -11.821632 | 28.20862227 | -3.133778 | 3.133778 | Down-regulated |
| ANAPC11 | 2.32E-03 | 4.50E-04 | -3.725517 | -0.55472782 | -3.096333 | 3.096333 | Down-regulated |
| TMEM256 | 2.67E-07 | 5.28E-09 | -6.872209 | 10.4024899 | -3.085738 | 3.085738 | Down-regulated |
| PPP4C | 1.18E-06 | 3.17E-08 | -6.402473 | 8.65573141 | -3.065345 | 3.065345 | Down-regulated |
| FOLR3 | 2.44E-08 | 2.96E-10 | -7.624691 | 13.21519318 | -3.064208 | 3.064208 | Down-regulated |
| GNS | 3.64E-09 | 2.84E-11 | -8.238658 | 15.5042142 | -3.050295 | 3.050295 | Down-regulated |
| DCTN2 | 7.42E-07 | 1.80E-08 | -6.551938 | 9.21002825 | -3.042932 | 3.042932 | Down-regulated |
| TXNDC17 | 1.43E-05 | 6.39E-07 | -5.604556 | 5.73695804 | -2.994491 | 2.994491 | Down-regulated |
| MCEMP1 | 5.28E-11 | 1.57E-13 | -9.622539 | 20.57532935 | -2.978156 | 2.978156 | Down-regulated |
| CREB1 | 1.09E-10 | 3.83E-13 | -9.381847 | 19.7054196 | -2.97542 | 2.97542 | Down-regulated |
| ATP5I | 3.71E-11 | 1.00E-13 | -9.743865 | 21.01154947 | -2.956552 | 2.956552 | Down-regulated |
| SEC11A | 2.75E-10 | 1.13E-12 | -9.091232 | 18.64754821 | -2.950315 | 2.950315 | Down-regulated |
| PSMB1 | 1.09E-08 | 1.11E-10 | -7.88192 | 14.17585292 | -2.93484 | 2.93484 | Down-regulated |
| TMEM208 | 3.24E-05 | 1.75E-06 | -5.331176 | 4.75957131 | -2.931681 | 2.931681 | Down-regulated |
| TSPO | 9.49E-08 | 1.51E-09 | -7.199693 | 11.6259141 | -2.858198 | 2.858198 | Down-regulated |
| FAM96B | 1.42E-07 | 2.44E-09 | -7.073945 | 11.1558378 | -2.80175 | 2.80175 | Down-regulated |
| PRCP | 2.23E-05 | 1.11E-06 | -5.456501 | 5.20582446 | -2.786687 | 2.786687 | Down-regulated |
| EMC6 | 1.88E-08 | 2.17E-10 | -7.705941 | 13.51881556 | -2.777679 | 2.777679 | Down-regulated |
| ATOX1 | 3.33E-11 | 8.65E-14 | -9.784226 | 21.15631568 | -2.772011 | 2.772011 | Down-regulated |
| SMARCD3 | 2.37E-16 | 2.11E-20 | -14.248818 | 35.88357342 | -2.771504 | 2.771504 | Down-regulated |
| C20orf24 | 3.66E-09 | 2.88E-11 | -8.235143 | 15.49115306 | -2.739795 | 2.739795 | Down-regulated |
| ATP6V0B | 7.38E-05 | 4.86E-06 | -5.05101 | 3.77449701 | -2.738814 | 2.738814 | Down-regulated |
| SPSB3 | 4.25E-08 | 5.78E-10 | -7.449699 | 12.56091159 | -2.738172 | 2.738172 | Down-regulated |
| RNF7 | 3.27E-09 | 2.53E-11 | -8.268886 | 15.61649157 | -2.730879 | 2.730879 | Down-regulated |
| PFN1 | 5.96E-06 | 2.18E-07 | -5.892846 | 6.78186612 | -2.726116 | 2.726116 | Down-regulated |
| COX4I1 | 1.10E-06 | 2.92E-08 | -6.42408 | 8.73575558 | -2.696157 | 2.696157 | Down-regulated |
| RPN1 | 5.14E-08 | 7.30E-10 | -7.389092 | 12.33424262 | -2.692608 | 2.692608 | Down-regulated |
| POLE4 | 3.29E-06 | 1.07E-07 | -6.08153 | 7.47222846 | -2.685842 | 2.685842 | Down-regulated |
| ATP5EP2 | 1.47E-06 | 4.16E-08 | -6.330874 | 8.39084091 | -2.672915 | 2.672915 | Down-regulated |
| PLOD1 | 5.13E-07 | 1.14E-08 | -6.67151 | 9.65457876 | -2.671788 | 2.671788 | Down-regulated |
| OPRL1 | 4.10E-05 | 2.34E-06 | -5.252653 | 4.48165656 | -2.67089 | 2.67089 | Down-regulated |
| TPST2 | 1.27E-03 | 1.93E-04 | -3.987203 | 0.24608097 | -2.6701 | 2.6701 | Down-regulated |
| MYL12B | 3.10E-05 | 1.67E-06 | -5.344312 | 4.80619426 | -2.669312 | 2.669312 | Down-regulated |
| PSMB6 | 4.96E-14 | 1.76E-17 | -12.184732 | 29.40847869 | -2.668238 | 2.668238 | Down-regulated |
| ATP6V1D | 7.53E-07 | 1.84E-08 | -6.546203 | 9.18873054 | -2.661926 | 2.661926 | Down-regulated |
| MTIF3 | 2.33E-05 | 1.17E-06 | -5.441859 | 5.15352574 | -2.660268 | 2.660268 | Down-regulated |
| YWHAH | 3.98E-14 | 9.72E-18 | -12.360648 | 29.98328864 | -2.652179 | 2.652179 | Down-regulated |
| TCEB2 | 1.28E-11 | 2.48E-14 | -10.125801 | 22.3742272 | -2.641996 | 2.641996 | Down-regulated |
| TRABD | 3.53E-09 | 2.74E-11 | -8.247584 | 15.53737485 | -2.640001 | 2.640001 | Down-regulated |
| CHFR | 3.85E-11 | 1.05E-13 | -9.731092 | 20.9656964 | -2.633 | 2.633 | Down-regulated |
| TRAPPC8 | 2.63E-07 | 5.18E-09 | -6.877571 | 10.4224977 | -2.632839 | 2.632839 | Down-regulated |
| DOCK2 | 5.10E-09 | 4.36E-11 | -8.125787 | 15.08454586 | -2.628804 | 2.628804 | Down-regulated |
| TRAPPC2L | 3.34E-11 | 8.74E-14 | -9.781432 | 21.1462977 | -2.623915 | 2.623915 | Down-regulated |
| SEC61A1 | 3.45E-13 | 2.45E-16 | -11.420284 | 26.86156931 | -2.619874 | 2.619874 | Down-regulated |
| HSBP1 | 3.11E-10 | 1.34E-12 | -9.04767 | 18.48830971 | -2.617736 | 2.617736 | Down-regulated |
| CHCHD2 | 8.53E-05 | 5.78E-06 | -5.002775 | 3.60682426 | -2.613411 | 2.613411 | Down-regulated |
| SSU72 | 1.63E-08 | 1.79E-10 | -7.756108 | 13.70620786 | -2.612205 | 2.612205 | Down-regulated |
| ATP6AP1 | 1.44E-16 | 9.60E-21 | -14.501396 | 36.63566181 | -2.597706 | 2.597706 | Down-regulated |
| UBL5 | 9.44E-09 | 9.26E-11 | -7.928434 | 14.34935192 | -2.581384 | 2.581384 | Down-regulated |
| NOL12 | 2.84E-09 | 2.15E-11 | -8.312213 | 15.77733906 | -2.569254 | 2.569254 | Down-regulated |
| EXOSC3 | 1.76E-10 | 6.66E-13 | -9.233758 | 19.16734852 | -2.552398 | 2.552398 | Down-regulated |
| CA4 | 3.47E-06 | 1.15E-07 | -6.063262 | 7.40519376 | -2.545043 | 2.545043 | Down-regulated |
| SZRD1 | 1.93E-06 | 5.71E-08 | -6.247762 | 8.08393995 | -2.541287 | 2.541287 | Down-regulated |
| SRPK1 | 1.75E-07 | 3.16E-09 | -7.006757 | 10.90480313 | -2.541132 | 2.541132 | Down-regulated |
| ATP5J2 | 4.96E-14 | 1.68E-17 | -12.19918 | 29.4558473 | -2.537805 | 2.537805 | Down-regulated |
| TMED4 | 1.79E-04 | 1.51E-05 | -4.733816 | 2.68352232 | -2.528816 | 2.528816 | Down-regulated |
| C14orf2 | 4.09E-03 | 1.09E-03 | -3.442629 | -1.3830364 | -2.52244 | 2.52244 | Down-regulated |
| EIF4G3 | 1.14E-06 | 3.02E-08 | -6.415315 | 8.7032894 | -2.52211 | 2.52211 | Down-regulated |
| STOM | 8.68E-10 | 4.84E-12 | -8.70535 | 17.23144286 | -2.521978 | 2.521978 | Down-regulated |
| NSF | 3.69E-07 | 7.68E-09 | -6.774316 | 10.03744796 | -2.520237 | 2.520237 | Down-regulated |
| GALK1 | 1.39E-11 | 2.81E-14 | -10.09124 | 22.25159744 | -2.519704 | 2.519704 | Down-regulated |
| RAB7A | 8.41E-09 | 8.12E-11 | -7.962815 | 14.4775464 | -2.519454 | 2.519454 | Down-regulated |
| PSMA6 | 2.15E-10 | 8.40E-13 | -9.171505 | 18.94053663 | -2.509782 | 2.509782 | Down-regulated |
| LSM1 | 1.69E-05 | 7.85E-07 | -5.549057 | 5.53738407 | -2.508826 | 2.508826 | Down-regulated |
| ETF1 | 6.64E-14 | 2.51E-17 | -12.081152 | 29.06803911 | -2.482066 | 2.482066 | Down-regulated |
| SNX6 | 3.06E-08 | 3.95E-10 | -7.549437 | 12.93387234 | -2.479488 | 2.479488 | Down-regulated |
| HIST2H2AC | 1.19E-06 | 3.22E-08 | -6.398657 | 8.64160374 | -2.47564 | 2.47564 | Down-regulated |
| ZMPSTE24 | 1.19E-11 | 2.18E-14 | -10.160917 | 22.4986822 | -2.472 | 2.472 | Down-regulated |
| PSMB3 | 1.52E-08 | 1.64E-10 | -7.778695 | 13.79055516 | -2.471554 | 2.471554 | Down-regulated |
| TMEM205 | 7.18E-06 | 2.75E-07 | -5.831061 | 6.55684874 | -2.468361 | 2.468361 | Down-regulated |
| DUSP23 | 4.50E-08 | 6.19E-10 | -7.431912 | 12.49439042 | -2.464663 | 2.464663 | Down-regulated |
| TYK2 | 1.15E-07 | 1.91E-09 | -7.138053 | 11.39545642 | -2.463249 | 2.463249 | Down-regulated |
| NAPRT | 3.40E-04 | 3.43E-05 | -4.498682 | 1.89426547 | -2.461197 | 2.461197 | Down-regulated |
| SEC61B | 2.33E-08 | 2.80E-10 | -7.63917 | 13.26931132 | -2.461075 | 2.461075 | Down-regulated |
| FES | 1.53E-07 | 2.68E-09 | -7.049752 | 11.06543236 | -2.460665 | 2.460665 | Down-regulated |
| LPAR2 | 6.92E-06 | 2.62E-07 | -5.843907 | 6.6035868 | -2.458982 | 2.458982 | Down-regulated |
| LTA4H | 5.82E-04 | 6.87E-05 | -4.296495 | 1.23070329 | -2.457981 | 2.457981 | Down-regulated |
| SLC26A6 | 3.02E-09 | 2.31E-11 | -8.293419 | 15.70758023 | -2.442933 | 2.442933 | Down-regulated |
| POLR2F | 1.15E-09 | 7.09E-12 | -8.604125 | 16.8580382 | -2.44283 | 2.44283 | Down-regulated |
| TMEM183A | 1.64E-05 | 7.54E-07 | -5.559939 | 5.5764712 | -2.441696 | 2.441696 | Down-regulated |
| TANK | 3.00E-06 | 9.59E-08 | -6.110564 | 7.57885014 | -2.419379 | 2.419379 | Down-regulated |
| NOTCH1 | 8.00E-05 | 5.36E-06 | -5.023973 | 3.68043988 | -2.416038 | 2.416038 | Down-regulated |
| TMBIM4 | 6.56E-04 | 8.03E-05 | -4.250481 | 1.08179758 | -2.39964 | 2.39964 | Down-regulated |
| MRPL33 | 3.55E-06 | 1.18E-07 | -6.056067 | 7.37880165 | -2.396191 | 2.396191 | Down-regulated |
| IFI27L2 | 9.82E-08 | 1.57E-09 | -7.189038 | 11.58607292 | -2.39616 | 2.39616 | Down-regulated |
| CNIH4 | 4.29E-13 | 3.62E-16 | -11.308143 | 26.48133617 | -2.393394 | 2.393394 | Down-regulated |
| GPAT3 | 3.76E-05 | 2.10E-06 | -5.281384 | 4.5831871 | -2.389093 | 2.389093 | Down-regulated |
| H2AFJ | 3.07E-04 | 3.00E-05 | -4.537469 | 2.02320936 | -2.386156 | 2.386156 | Down-regulated |
| SLC9A1 | 2.53E-07 | 4.90E-09 | -6.891823 | 10.47567625 | -2.370889 | 2.370889 | Down-regulated |
| UPP1 | 1.70E-09 | 1.10E-11 | -8.487439 | 16.42670922 | -2.370338 | 2.370338 | Down-regulated |
| ANKRD33 | 8.37E-09 | 8.06E-11 | -7.964912 | 14.4853637 | -2.369127 | 2.369127 | Down-regulated |
| ARHGDIB | 3.32E-12 | 4.13E-15 | -10.621474 | 24.11732775 | -2.366622 | 2.366622 | Down-regulated |
| BUD31 | 3.93E-03 | 1.02E-03 | -3.464043 | -1.32178804 | -2.364614 | 2.364614 | Down-regulated |
| MAPKAPK3 | 9.21E-08 | 1.46E-09 | -7.20871 | 11.65963013 | -2.345203 | 2.345203 | Down-regulated |
| FAR1 | 4.33E-03 | 1.20E-03 | -3.411829 | -1.47069838 | -2.344972 | 2.344972 | Down-regulated |
| APBB3 | 2.83E-08 | 3.54E-10 | -7.578033 | 13.0407823 | -2.332612 | 2.332612 | Down-regulated |
| ZFAND2A | 5.68E-03 | 1.88E-03 | -3.260899 | -1.89269199 | -2.331137 | 2.331137 | Down-regulated |
| BCKDK | 1.85E-13 | 9.05E-17 | -11.70665 | 27.82492322 | -2.324723 | 2.324723 | Down-regulated |
| LAMTOR2 | 1.59E-11 | 3.61E-14 | -10.022446 | 22.00709743 | -2.315423 | 2.315423 | Down-regulated |
| UGCG | 1.86E-05 | 8.86E-07 | -5.516309 | 5.41988927 | -2.314167 | 2.314167 | Down-regulated |
| FBXL20 | 1.95E-08 | 2.27E-10 | -7.694116 | 13.47463507 | -2.313895 | 2.313895 | Down-regulated |
| RHOT1 | 7.08E-09 | 6.51E-11 | -8.020795 | 14.69362552 | -2.312767 | 2.312767 | Down-regulated |
| N4BP2L2 | 3.88E-07 | 8.15E-09 | -6.758876 | 9.97991399 | -2.31213 | 2.31213 | Down-regulated |
| SSNA1 | 3.45E-13 | 2.32E-16 | -11.434956 | 26.91119421 | -2.308116 | 2.308116 | Down-regulated |
| MRPS18C | 8.26E-10 | 4.53E-12 | -8.722643 | 17.29515789 | -2.307545 | 2.307545 | Down-regulated |
| OXLD1 | 1.78E-07 | 3.23E-09 | -7.000474 | 10.88133594 | -2.307252 | 2.307252 | Down-regulated |
| ATG7 | 2.59E-10 | 1.05E-12 | -9.113022 | 18.72713634 | -2.304407 | 2.304407 | Down-regulated |
| MCU | 9.07E-04 | 1.22E-04 | -4.124839 | 0.67942819 | -2.299245 | 2.299245 | Down-regulated |
| VAMP8 | 1.32E-09 | 8.31E-12 | -8.562447 | 16.70408309 | -2.296659 | 2.296659 | Down-regulated |
| TMEM258 | 2.78E-06 | 8.70E-08 | -6.136481 | 7.67410615 | -2.295253 | 2.295253 | Down-regulated |
| TAF10 | 5.02E-07 | 1.11E-08 | -6.678547 | 9.68076621 | -2.294758 | 2.294758 | Down-regulated |
| ARF1 | 9.52E-07 | 2.44E-08 | -6.471116 | 8.91008551 | -2.291692 | 2.291692 | Down-regulated |
| CCDC53 | 1.37E-07 | 2.34E-09 | -7.084463 | 11.19514529 | -2.290074 | 2.290074 | Down-regulated |
| FAM120B | 2.20E-09 | 1.58E-11 | -8.392814 | 16.07626951 | -2.289257 | 2.289257 | Down-regulated |
| SLC44A1 | 3.90E-08 | 5.23E-10 | -7.476176 | 12.65992909 | -2.288457 | 2.288457 | Down-regulated |
| RNF181 | 5.07E-08 | 7.15E-10 | -7.394575 | 12.35475086 | -2.287233 | 2.287233 | Down-regulated |
| GTF2E2 | 3.34E-12 | 4.23E-15 | -10.614928 | 24.09450285 | -2.283152 | 2.283152 | Down-regulated |
| PTTG1IP | 1.15E-09 | 7.00E-12 | -8.607556 | 16.8707078 | -2.272776 | 2.272776 | Down-regulated |
| FAM53C | 6.87E-04 | 8.57E-05 | -4.231103 | 1.01933227 | -2.265166 | 2.265166 | Down-regulated |
| GPR137 | 2.14E-07 | 4.01E-09 | -6.944559 | 10.67252434 | -2.263289 | 2.263289 | Down-regulated |
| WASH3P | 6.73E-05 | 4.32E-06 | -5.083366 | 3.88730656 | -2.262661 | 2.262661 | Down-regulated |
| AIM2 | 4.37E-06 | 1.51E-07 | -5.9903 | 7.13786123 | -2.261769 | 2.261769 | Down-regulated |
| UBE2M | 2.59E-04 | 2.40E-05 | -4.601883 | 2.23846371 | -2.258582 | 2.258582 | Down-regulated |
| LEPROT | 3.24E-12 | 3.96E-15 | -10.633342 | 24.15869717 | -2.258162 | 2.258162 | Down-regulated |
| PCNX1 | 4.39E-09 | 3.54E-11 | -8.180691 | 15.28876634 | -2.256229 | 2.256229 | Down-regulated |
| CCDC12 | 2.46E-13 | 1.42E-16 | -11.576526 | 27.38854195 | -2.255561 | 2.255561 | Down-regulated |
| STX5 | 1.23E-07 | 2.07E-09 | -7.116693 | 11.31560849 | -2.250697 | 2.250697 | Down-regulated |
| CD74 | 7.91E-12 | 1.25E-14 | -10.314662 | 23.04186467 | -2.246749 | 2.246749 | Down-regulated |
| WASH1 | 1.29E-06 | 3.53E-08 | -6.374618 | 8.55262706 | -2.245325 | 2.245325 | Down-regulated |
| COPS7A | 9.83E-12 | 1.70E-14 | -10.229612 | 22.74172736 | -2.243256 | 2.243256 | Down-regulated |
| NDUFV2 | 1.02E-03 | 1.45E-04 | -4.075065 | 0.52179637 | -2.241691 | 2.241691 | Down-regulated |
| FIBP | 4.07E-08 | 5.49E-10 | -7.463318 | 12.61184468 | -2.24045 | 2.24045 | Down-regulated |
| AUP1 | 1.00E-06 | 2.62E-08 | -6.453057 | 8.84313434 | -2.23447 | 2.23447 | Down-regulated |
| USP3 | 1.43E-08 | 1.52E-10 | -7.798586 | 13.86482492 | -2.231406 | 2.231406 | Down-regulated |
| WSB1 | 2.23E-04 | 2.00E-05 | -4.653572 | 2.41217651 | -2.230295 | 2.230295 | Down-regulated |
| CWC15 | 5.83E-10 | 2.93E-12 | -8.838826 | 17.72264087 | -2.228649 | 2.228649 | Down-regulated |
| ATP5L | 1.50E-05 | 6.80E-07 | -5.587993 | 5.67733978 | -2.225646 | 2.225646 | Down-regulated |
| TMEM91 | 2.39E-11 | 5.69E-14 | -9.898321 | 21.56458249 | -2.225337 | 2.225337 | Down-regulated |
| EMC7 | 7.48E-08 | 1.15E-09 | -7.271076 | 11.89285596 | -2.225049 | 2.225049 | Down-regulated |
| ZBTB48 | 1.97E-04 | 1.71E-05 | -4.698239 | 2.5629712 | -2.223585 | 2.223585 | Down-regulated |
| MTMR14 | 1.13E-03 | 1.66E-04 | -4.034117 | 0.39288985 | -2.220056 | 2.220056 | Down-regulated |
| VKORC1 | 1.33E-04 | 1.02E-05 | -4.842998 | 3.05585424 | -2.218419 | 2.218419 | Down-regulated |
| C5orf15 | 9.69E-08 | 1.54E-09 | -7.193697 | 11.6034961 | -2.215505 | 2.215505 | Down-regulated |
| MCRS1 | 1.93E-17 | 8.55E-22 | -15.294497 | 38.94136329 | -2.211165 | 2.211165 | Down-regulated |
| NDUFB3 | 8.87E-05 | 6.10E-06 | -4.987959 | 3.55544451 | -2.210675 | 2.210675 | Down-regulated |
| TNFAIP6 | 2.71E-09 | 2.02E-11 | -8.32751 | 15.83410239 | -2.209963 | 2.209963 | Down-regulated |
| ATP5H | 1.93E-09 | 1.33E-11 | -8.439157 | 16.24796786 | -2.206939 | 2.206939 | Down-regulated |
| DNAJA1 | 2.20E-11 | 5.10E-14 | -9.928391 | 21.67194347 | -2.205229 | 2.205229 | Down-regulated |
| EXOSC1 | 5.57E-07 | 1.26E-08 | -6.645629 | 9.55828429 | -2.202179 | 2.202179 | Down-regulated |
| ADAM15 | 4.96E-14 | 1.53E-17 | -12.226385 | 29.54496474 | -2.201834 | 2.201834 | Down-regulated |
| H2AFY | 7.87E-10 | 4.26E-12 | -8.738984 | 17.35534609 | -2.200931 | 2.200931 | Down-regulated |
| MSRB1 | 5.85E-07 | 1.33E-08 | -6.631043 | 9.50402995 | -2.200138 | 2.200138 | Down-regulated |
| S100A12 | 2.62E-05 | 1.36E-06 | -5.401218 | 5.00857919 | -2.199655 | 2.199655 | Down-regulated |
| GYG1 | 1.51E-07 | 2.62E-09 | -7.055264 | 11.0860315 | -2.199053 | 2.199053 | Down-regulated |
| TIMM17B | 6.24E-09 | 5.59E-11 | -8.060794 | 14.84261573 | -2.197672 | 2.197672 | Down-regulated |
| POLR2G | 5.14E-09 | 4.43E-11 | -8.121901 | 15.07008635 | -2.197501 | 2.197501 | Down-regulated |
| FAM50A | 4.87E-06 | 1.71E-07 | -5.956569 | 7.01449905 | -2.191583 | 2.191583 | Down-regulated |
| SDHAF2 | 2.19E-14 | 4.87E-18 | -12.566418 | 30.65023754 | -2.190364 | 2.190364 | Down-regulated |
| EMD | 8.06E-06 | 3.17E-07 | -5.792811 | 6.41782167 | -2.185445 | 2.185445 | Down-regulated |
| NARS | 3.59E-10 | 1.62E-12 | -8.995518 | 18.2974523 | -2.183926 | 2.183926 | Down-regulated |
| IRAK3 | 3.12E-08 | 4.03E-10 | -7.543808 | 12.91282663 | -2.183405 | 2.183405 | Down-regulated |
| AURKAIP1 | 2.10E-07 | 3.90E-09 | -6.951425 | 10.69815842 | -2.181219 | 2.181219 | Down-regulated |
| RAB24 | 1.51E-11 | 3.25E-14 | -10.051339 | 22.10985225 | -2.179048 | 2.179048 | Down-regulated |
| TIMP1 | 1.25E-10 | 4.50E-13 | -9.338514 | 19.54818885 | -2.179047 | 2.179047 | Down-regulated |
| COMMD3 | 9.54E-10 | 5.40E-12 | -8.676323 | 17.12444349 | -2.178642 | 2.178642 | Down-regulated |
| LAMP2 | 2.69E-10 | 1.10E-12 | -9.099996 | 18.6795635 | -2.178119 | 2.178119 | Down-regulated |
| IFNAR1 | 1.97E-09 | 1.39E-11 | -8.427374 | 16.20432823 | -2.177411 | 2.177411 | Down-regulated |
| RPS6KB2 | 1.42E-03 | 2.25E-04 | -3.941346 | 0.10350306 | -2.177125 | 2.177125 | Down-regulated |
| CDC26 | 3.55E-04 | 3.63E-05 | -4.482657 | 1.84114318 | -2.176205 | 2.176205 | Down-regulated |
| IFNGR2 | 1.21E-06 | 3.30E-08 | -6.392084 | 8.61727009 | -2.176034 | 2.176034 | Down-regulated |
| PPP6C | 2.84E-08 | 3.58E-10 | -7.575294 | 13.03054253 | -2.174853 | 2.174853 | Down-regulated |
| TMEM183B | 5.83E-10 | 2.86E-12 | -8.844901 | 17.74496299 | -2.174615 | 2.174615 | Down-regulated |
| HMGB2 | 1.39E-11 | 2.79E-14 | -10.093433 | 22.25938506 | -2.171249 | 2.171249 | Down-regulated |
| ADAP1 | 3.87E-06 | 1.31E-07 | -6.027828 | 7.27527956 | -2.170597 | 2.170597 | Down-regulated |
| TNFRSF1B | 8.52E-14 | 3.41E-17 | -11.991316 | 28.77158124 | -2.169247 | 2.169247 | Down-regulated |
| YIPF1 | 3.56E-07 | 7.35E-09 | -6.785791 | 10.08021908 | -2.169034 | 2.169034 | Down-regulated |
| RALA | 6.01E-05 | 3.75E-06 | -5.122806 | 4.02516313 | -2.165256 | 2.165256 | Down-regulated |
| ACADVL | 1.44E-07 | 2.48E-09 | -7.069651 | 11.13979191 | -2.164512 | 2.164512 | Down-regulated |
| PCMT1 | 8.19E-08 | 1.28E-09 | -7.243287 | 11.78893204 | -2.162583 | 2.162583 | Down-regulated |
| FEM1C | 4.90E-05 | 2.89E-06 | -5.194143 | 4.27547008 | -2.161981 | 2.161981 | Down-regulated |
| GRSF1 | 3.06E-11 | 7.69E-14 | -9.816337 | 21.27136163 | -2.161 | 2.161 | Down-regulated |
| SIL1 | 5.29E-06 | 1.90E-07 | -5.929547 | 6.91578255 | -2.160232 | 2.160232 | Down-regulated |
| TCEB1 | 2.54E-05 | 1.31E-06 | -5.411477 | 5.04513549 | -2.159251 | 2.159251 | Down-regulated |
| UQCRFS1 | 4.43E-06 | 1.53E-07 | -5.985965 | 7.12199976 | -2.156494 | 2.156494 | Down-regulated |
| OSBP | 7.20E-05 | 4.70E-06 | -5.060101 | 3.80616625 | -2.156149 | 2.156149 | Down-regulated |
| CSNK1G1 | 2.98E-11 | 7.34E-14 | -9.828893 | 21.31631817 | -2.154569 | 2.154569 | Down-regulated |
| TRAPPC1 | 4.20E-08 | 5.71E-10 | -7.453202 | 12.57401345 | -2.154166 | 2.154166 | Down-regulated |
| BSCL2 | 2.09E-07 | 3.88E-09 | -6.953103 | 10.70442639 | -2.152718 | 2.152718 | Down-regulated |
| SPATA2L | 2.22E-05 | 1.10E-06 | -5.457232 | 5.2084362 | -2.15232 | 2.15232 | Down-regulated |
| COMMD1 | 8.97E-05 | 6.19E-06 | -4.98366 | 3.54054374 | -2.149207 | 2.149207 | Down-regulated |
| GGH | 4.31E-07 | 9.20E-09 | -6.727168 | 9.86179124 | -2.14872 | 2.14872 | Down-regulated |
| GLG1 | 4.10E-04 | 4.35E-05 | -4.429818 | 1.66661022 | -2.147185 | 2.147185 | Down-regulated |
| ALOX5AP | 1.19E-11 | 2.20E-14 | -10.158604 | 22.49048664 | -2.14645 | 2.14645 | Down-regulated |
| PSMC1 | 4.61E-07 | 1.00E-08 | -6.704875 | 9.7787776 | -2.144303 | 2.144303 | Down-regulated |
| IMPDH1 | 5.87E-07 | 1.34E-08 | -6.629531 | 9.49840538 | -2.143555 | 2.143555 | Down-regulated |
| CCNT1 | 6.56E-10 | 3.42E-12 | -8.797092 | 17.56920515 | -2.142876 | 2.142876 | Down-regulated |
| MRPS11 | 3.00E-07 | 6.07E-09 | -6.8359 | 10.26704544 | -2.142705 | 2.142705 | Down-regulated |
| CAMP | 2.51E-05 | 1.28E-06 | -5.416048 | 5.06143016 | -2.142389 | 2.142389 | Down-regulated |
| VIMP | 1.01E-09 | 5.90E-12 | -8.652772 | 17.03758359 | -2.140023 | 2.140023 | Down-regulated |
| MANF | 1.32E-05 | 5.76E-07 | -5.632514 | 5.83770206 | -2.136095 | 2.136095 | Down-regulated |
| INSIG1 | 1.13E-03 | 1.65E-04 | -4.035513 | 0.39727246 | -2.13091 | 2.13091 | Down-regulated |
| PIN1 | 8.91E-05 | 6.13E-06 | -4.986587 | 3.55068947 | -2.130393 | 2.130393 | Down-regulated |
| C8orf76 | 7.67E-09 | 7.32E-11 | -7.990035 | 14.57900751 | -2.129749 | 2.129749 | Down-regulated |
| PLBD1 | 6.83E-19 | 1.52E-23 | -16.676729 | 42.76282379 | -2.129207 | 2.129207 | Down-regulated |
| CD99L2 | 1.55E-11 | 3.43E-14 | -10.036822 | 22.05823754 | -2.128926 | 2.128926 | Down-regulated |
| TDG | 1.43E-11 | 2.92E-14 | -10.080871 | 22.21477889 | -2.128923 | 2.128923 | Down-regulated |
| AGO2 | 2.40E-06 | 7.38E-08 | -6.179762 | 7.83334801 | -2.128211 | 2.128211 | Down-regulated |
| INTS6 | 9.27E-07 | 2.37E-08 | -6.479082 | 8.93962788 | -2.128081 | 2.128081 | Down-regulated |
| TPI1P2 | 7.67E-13 | 7.84E-16 | -11.088469 | 25.73168382 | -2.126061 | 2.126061 | Down-regulated |
| NDUFA4 | 3.21E-05 | 1.74E-06 | -5.333654 | 4.76836231 | -2.125763 | 2.125763 | Down-regulated |
| RPP21 | 1.26E-09 | 7.88E-12 | -8.576526 | 16.75610177 | -2.125608 | 2.125608 | Down-regulated |
| RHOQ | 2.53E-07 | 4.91E-09 | -6.891579 | 10.4747654 | -2.123082 | 2.123082 | Down-regulated |
| IGF2R | 1.45E-04 | 1.15E-05 | -4.811004 | 2.94638683 | -2.120111 | 2.120111 | Down-regulated |
| MYL6B | 8.45E-07 | 2.11E-08 | -6.509256 | 9.05157308 | -2.118243 | 2.118243 | Down-regulated |
| TIMM23 | 3.01E-09 | 2.29E-11 | -8.294774 | 15.71261165 | -2.117108 | 2.117108 | Down-regulated |
| TBC1D7 | 4.86E-11 | 1.41E-13 | -9.651213 | 20.67856359 | -2.11626 | 2.11626 | Down-regulated |
| RAB10 | 1.48E-11 | 3.06E-14 | -10.068225 | 22.16986039 | -2.11209 | 2.11209 | Down-regulated |
| CCPG1 | 3.89E-13 | 3.20E-16 | -11.343614 | 26.6017867 | -2.107751 | 2.107751 | Down-regulated |
| PPM1M | 1.47E-08 | 1.57E-10 | -7.790025 | 13.83286147 | -2.106585 | 2.106585 | Down-regulated |
| OSER1 | 5.10E-06 | 1.81E-07 | -5.942301 | 6.9623638 | -2.105821 | 2.105821 | Down-regulated |
| NDUFA8 | 6.71E-13 | 6.41E-16 | -11.14555 | 25.92708634 | -2.10512 | 2.10512 | Down-regulated |
| RPS6KA1 | 3.53E-13 | 2.74E-16 | -11.387598 | 26.75091511 | -2.104587 | 2.104587 | Down-regulated |
| SLC16A3 | 1.80E-07 | 3.28E-09 | -6.996645 | 10.86703277 | -2.103994 | 2.103994 | Down-regulated |
| NDUFB8 | 2.59E-09 | 1.92E-11 | -8.340969 | 15.88403332 | -2.102887 | 2.102887 | Down-regulated |
| COA3 | 5.02E-10 | 2.38E-12 | -8.893984 | 17.92521435 | -2.101869 | 2.101869 | Down-regulated |
| SNRPD2 | 7.44E-07 | 1.80E-08 | -6.551072 | 9.20681219 | -2.099178 | 2.099178 | Down-regulated |
| R3HCC1 | 2.14E-13 | 1.09E-16 | -11.652765 | 27.64449365 | -2.096184 | 2.096184 | Down-regulated |
| GLA | 2.91E-05 | 1.54E-06 | -5.366527 | 4.88511998 | -2.095577 | 2.095577 | Down-regulated |
| DERL2 | 7.39E-06 | 2.84E-07 | -5.821626 | 6.52253559 | -2.095508 | 2.095508 | Down-regulated |
| RNASE2 | 3.24E-15 | 3.59E-19 | -13.358277 | 33.16215016 | -2.094902 | 2.094902 | Down-regulated |
| VPS16 | 5.48E-12 | 7.91E-15 | -10.44068 | 23.48498252 | -2.094681 | 2.094681 | Down-regulated |
| STX16 | 4.02E-07 | 8.49E-09 | -6.748137 | 9.93990057 | -2.093013 | 2.093013 | Down-regulated |
| C19orf53 | 7.93E-06 | 3.10E-07 | -5.798489 | 6.43844458 | -2.089497 | 2.089497 | Down-regulated |
| FAM160B1 | 4.25E-04 | 4.56E-05 | -4.4166 | 1.62310552 | -2.087765 | 2.087765 | Down-regulated |
| GLTP | 7.20E-06 | 2.76E-07 | -5.829857 | 6.55246723 | -2.087757 | 2.087757 | Down-regulated |
| ANAPC13 | 7.57E-06 | 2.93E-07 | -5.813479 | 6.49291461 | -2.087663 | 2.087663 | Down-regulated |
| PIK3AP1 | 5.21E-05 | 3.14E-06 | -5.171935 | 4.19741663 | -2.087478 | 2.087478 | Down-regulated |
| GATAD2A | 1.07E-06 | 2.81E-08 | -6.434358 | 8.77383443 | -2.084719 | 2.084719 | Down-regulated |
| NDUFS7 | 1.10E-04 | 8.09E-06 | -4.908983 | 3.28254457 | -2.082701 | 2.082701 | Down-regulated |
| MAPK13 | 3.23E-11 | 8.32E-14 | -9.794842 | 21.19436234 | -2.081134 | 2.081134 | Down-regulated |
| DPH3 | 2.25E-12 | 2.59E-15 | -10.751488 | 24.56955641 | -2.08095 | 2.08095 | Down-regulated |
| NFKBIA | 3.64E-11 | 9.77E-14 | -9.751187 | 21.03782301 | -2.080648 | 2.080648 | Down-regulated |
| SNX3 | 2.07E-06 | 6.18E-08 | -6.22679 | 8.00660501 | -2.080109 | 2.080109 | Down-regulated |
| EWSR1 | 7.65E-09 | 7.23E-11 | -7.993185 | 14.59074804 | -2.080011 | 2.080011 | Down-regulated |
| TMCO1 | 1.02E-04 | 7.30E-06 | -4.937898 | 3.38226455 | -2.079854 | 2.079854 | Down-regulated |
| NCSTN | 7.60E-05 | 5.03E-06 | -5.041505 | 3.7414101 | -2.079597 | 2.079597 | Down-regulated |
| PHF23 | 1.40E-02 | 8.91E-03 | -2.708925 | -3.31953453 | -2.078793 | 2.078793 | Down-regulated |
| SCARB2 | 6.05E-09 | 5.33E-11 | -8.07316 | 14.8886611 | -2.076843 | 2.076843 | Down-regulated |
| MRPS22 | 4.14E-05 | 2.37E-06 | -5.249188 | 4.46942398 | -2.072735 | 2.072735 | Down-regulated |
| PDK3 | 4.48E-08 | 6.14E-10 | -7.434005 | 12.50221761 | -2.072273 | 2.072273 | Down-regulated |
| CLTB | 4.04E-04 | 4.27E-05 | -4.435352 | 1.68484456 | -2.070483 | 2.070483 | Down-regulated |
| BATF | 6.73E-07 | 1.58E-08 | -6.585307 | 9.33399927 | -2.070348 | 2.070348 | Down-regulated |
| CYC1 | 6.49E-06 | 2.42E-07 | -5.864246 | 6.67763919 | -2.069854 | 2.069854 | Down-regulated |
| TMEM87A | 4.96E-14 | 1.76E-17 | -12.185075 | 29.40960318 | -2.069375 | 2.069375 | Down-regulated |
| PRR13 | 4.39E-05 | 2.53E-06 | -5.230653 | 4.40403961 | -2.069362 | 2.069362 | Down-regulated |
| MRPS10 | 1.23E-07 | 2.07E-09 | -7.117464 | 11.31849316 | -2.068487 | 2.068487 | Down-regulated |
| KRTCAP2 | 1.05E-05 | 4.37E-07 | -5.706847 | 6.10619793 | -2.068343 | 2.068343 | Down-regulated |
| CASP1 | 4.24E-09 | 3.37E-11 | -8.193267 | 15.33552366 | -2.066356 | 2.066356 | Down-regulated |
| CHIC2 | 7.02E-07 | 1.67E-08 | -6.570194 | 9.27784467 | -2.065984 | 2.065984 | Down-regulated |
| PMM1 | 1.58E-07 | 2.76E-09 | -7.041688 | 11.03530367 | -2.06588 | 2.06588 | Down-regulated |
| PDXK | 3.64E-09 | 2.85E-11 | -8.238004 | 15.50178476 | -2.065673 | 2.065673 | Down-regulated |
| UQCRHL | 1.40E-09 | 8.87E-12 | -8.545159 | 16.64018391 | -2.062013 | 2.062013 | Down-regulated |
| PRDX5 | 1.28E-04 | 9.73E-06 | -4.857475 | 3.10548404 | -2.06169 | 2.06169 | Down-regulated |
| SEC22B | 6.68E-11 | 2.06E-13 | -9.548993 | 20.31014704 | -2.059337 | 2.059337 | Down-regulated |
| ASAP1 | 6.33E-08 | 9.43E-10 | -7.322112 | 12.08373098 | -2.057543 | 2.057543 | Down-regulated |
| CRIPT | 1.76E-08 | 1.98E-10 | -7.729663 | 13.60743236 | -2.056525 | 2.056525 | Down-regulated |
| TOM1 | 7.12E-09 | 6.58E-11 | -8.018008 | 14.68324451 | -2.055901 | 2.055901 | Down-regulated |
| RXRB | 9.55E-05 | 6.73E-06 | -4.960498 | 3.46036332 | -2.055376 | 2.055376 | Down-regulated |
| RPN2 | 3.75E-10 | 1.72E-12 | -8.980667 | 18.24306204 | -2.054974 | 2.054974 | Down-regulated |
| YIF1B | 4.09E-04 | 4.33E-05 | -4.431616 | 1.67253542 | -2.054834 | 2.054834 | Down-regulated |
| SAT1 | 5.69E-10 | 2.77E-12 | -8.853814 | 17.77771039 | -2.054191 | 2.054191 | Down-regulated |
| SARNP | 9.95E-05 | 7.09E-06 | -4.945998 | 3.41023892 | -2.052842 | 2.052842 | Down-regulated |
| TOR1A | 1.46E-04 | 1.15E-05 | -4.809393 | 2.9408822 | -2.052706 | 2.052706 | Down-regulated |
| SNF8 | 1.94E-06 | 5.74E-08 | -6.246091 | 8.07777632 | -2.049457 | 2.049457 | Down-regulated |
| GSK3B | 7.01E-13 | 7.01E-16 | -11.12022 | 25.84042709 | -2.047708 | 2.047708 | Down-regulated |
| RTFDC1 | 2.80E-05 | 1.47E-06 | -5.379433 | 4.93102055 | -2.047018 | 2.047018 | Down-regulated |
| RPL39L | 4.52E-07 | 9.77E-09 | -6.711397 | 9.80306068 | -2.0466 | 2.0466 | Down-regulated |
| PPFIA1 | 8.94E-12 | 1.51E-14 | -10.262291 | 22.85714938 | -2.044561 | 2.044561 | Down-regulated |
| GRN | 1.23E-14 | 2.18E-18 | -12.807931 | 31.42556474 | -2.043394 | 2.043394 | Down-regulated |
| PTRHD1 | 1.55E-04 | 1.25E-05 | -4.787128 | 2.86488697 | -2.042977 | 2.042977 | Down-regulated |
| LAMTOR1 | 6.35E-12 | 9.45E-15 | -10.391597 | 23.31261735 | -2.040762 | 2.040762 | Down-regulated |
| GLE1 | 1.07E-05 | 4.48E-07 | -5.699806 | 6.08072323 | -2.038893 | 2.038893 | Down-regulated |
| CYB5R1 | 7.58E-04 | 9.70E-05 | -4.194449 | 0.9015785 | -2.038742 | 2.038742 | Down-regulated |
| LRCH4 | 9.44E-08 | 1.50E-09 | -7.201515 | 11.63272933 | -2.03572 | 2.03572 | Down-regulated |
| PRR14 | 1.55E-11 | 3.45E-14 | -10.034981 | 22.05168817 | -2.033197 | 2.033197 | Down-regulated |
| SYF2 | 1.96E-09 | 1.37E-11 | -8.431288 | 16.21882298 | -2.02989 | 2.02989 | Down-regulated |
| INTS1 | 1.07E-07 | 1.75E-09 | -7.160571 | 11.47964136 | -2.027503 | 2.027503 | Down-regulated |
| SLC2A3 | 1.84E-05 | 8.73E-07 | -5.52053 | 5.4350228 | -2.022498 | 2.022498 | Down-regulated |
| TRPT1 | 6.50E-05 | 4.14E-06 | -5.095329 | 3.92907937 | -2.022326 | 2.022326 | Down-regulated |
| PSMB7 | 8.47E-12 | 1.41E-14 | -10.280779 | 22.92239621 | -2.018601 | 2.018601 | Down-regulated |
| NINJ1 | 1.66E-05 | 7.70E-07 | -5.554199 | 5.55585338 | -2.018048 | 2.018048 | Down-regulated |
| SEC22A | 1.79E-08 | 2.01E-10 | -7.725239 | 13.59090752 | -2.017096 | 2.017096 | Down-regulated |
| TINF2 | 3.90E-09 | 3.08E-11 | -8.217136 | 15.42424275 | -2.015065 | 2.015065 | Down-regulated |
| SLC11A1 | 1.10E-09 | 6.63E-12 | -8.622181 | 16.92469972 | -2.014296 | 2.014296 | Down-regulated |
| CFL1 | 1.02E-10 | 3.55E-13 | -9.402715 | 19.78107321 | -2.013677 | 2.013677 | Down-regulated |
| NBEAL2 | 3.54E-05 | 1.95E-06 | -5.30247 | 4.65781706 | -2.012898 | 2.012898 | Down-regulated |
| ABTB1 | 6.28E-09 | 5.64E-11 | -8.058339 | 14.83347298 | -2.012548 | 2.012548 | Down-regulated |
| CST7 | 1.28E-05 | 5.58E-07 | -5.641166 | 5.86890235 | -2.011466 | 2.011466 | Down-regulated |
| GPN3 | 2.88E-10 | 1.21E-12 | -9.07445 | 18.58622408 | -2.010697 | 2.010697 | Down-regulated |
| ARPC1A | 6.86E-05 | 4.44E-06 | -5.076245 | 3.86245528 | -2.008706 | 2.008706 | Down-regulated |
| NFKB1 | 3.02E-04 | 2.93E-05 | -4.544299 | 2.04596855 | -2.007975 | 2.007975 | Down-regulated |
| SCO2 | 3.56E-06 | 1.18E-07 | -6.054795 | 7.37413483 | -2.005948 | 2.005948 | Down-regulated |
| SLC26A8 | 2.46E-13 | 1.42E-16 | -11.577008 | 27.39016358 | -2.004527 | 2.004527 | Down-regulated |
| RGL4 | 1.20E-05 | 5.15E-07 | -5.662686 | 5.94657062 | -2.002647 | 2.002647 | Down-regulated |
| SAR1B | 1.15E-05 | 4.89E-07 | -5.676342 | 5.9958995 | -2.00174 | 2.00174 | Down-regulated |
| LILRA3 | 2.58E-12 | 3.04E-15 | -10.706934 | 24.41482167 | -2.001599 | 2.001599 | Down-regulated |
| C5orf51 | 1.92E-05 | 9.24E-07 | -5.505109 | 5.37975055 | -2.000393 | 2.000393 | Down-regulated |
| NPC2 | 8.68E-05 | 5.91E-06 | -4.99655 | 3.58523097 | -1.999954 | 1.999954 | Down-regulated |
| SLC15A3 | 6.77E-05 | 4.36E-06 | -5.081013 | 3.87909384 | -1.998947 | 1.998947 | Down-regulated |
| FAM172A | 1.84E-09 | 1.24E-11 | -8.457728 | 16.31673793 | -1.99643 | 1.99643 | Down-regulated |
| YY1 | 7.31E-11 | 2.35E-13 | -9.51335 | 20.18142687 | -1.995572 | 1.995572 | Down-regulated |
| ZFAND2B | 1.15E-09 | 6.99E-12 | -8.608165 | 16.87295552 | -1.994757 | 1.994757 | Down-regulated |
| FLOT2 | 7.03E-11 | 2.20E-13 | -9.531109 | 20.24557783 | -1.993973 | 1.993973 | Down-regulated |
| MRPL22 | 1.25E-11 | 2.33E-14 | -10.14282 | 22.43456109 | -1.993405 | 1.993405 | Down-regulated |
| CRADD | 8.47E-09 | 8.22E-11 | -7.959661 | 14.46578888 | -1.992933 | 1.992933 | Down-regulated |
| PSMC3 | 1.18E-08 | 1.21E-10 | -7.857957 | 14.08643765 | -1.992179 | 1.992179 | Down-regulated |
| DUS2 | 3.19E-11 | 8.15E-14 | -9.800451 | 21.21445888 | -1.991452 | 1.991452 | Down-regulated |
| CARS2 | 7.95E-05 | 5.31E-06 | -5.026572 | 3.68947129 | -1.990822 | 1.990822 | Down-regulated |
| ZNF622 | 1.11E-03 | 1.62E-04 | -4.041261 | 0.41532677 | -1.990546 | 1.990546 | Down-regulated |
| GUSB | 4.62E-08 | 6.40E-10 | -7.423508 | 12.46295917 | -1.988727 | 1.988727 | Down-regulated |
| DYNLL1 | 2.51E-07 | 4.86E-09 | -6.894149 | 10.48435829 | -1.988692 | 1.988692 | Down-regulated |
| TYMP | 1.72E-08 | 1.91E-10 | -7.739027 | 13.64241224 | -1.988321 | 1.988321 | Down-regulated |
| CD14 | 1.80E-08 | 2.05E-10 | -7.720037 | 13.57147542 | -1.986876 | 1.986876 | Down-regulated |
| GLIPR2 | 4.63E-11 | 1.34E-13 | -9.666472 | 20.73346669 | -1.986082 | 1.986082 | Down-regulated |
| RPL26L1 | 1.92E-08 | 2.23E-10 | -7.698718 | 13.4918287 | -1.985994 | 1.985994 | Down-regulated |
| MRPS15 | 2.52E-07 | 4.88E-09 | -6.892785 | 10.47926602 | -1.985273 | 1.985273 | Down-regulated |
| TNFRSF14 | 1.00E-07 | 1.61E-09 | -7.183041 | 11.56364981 | -1.981546 | 1.981546 | Down-regulated |
| CCNY | 1.07E-07 | 1.76E-09 | -7.158672 | 11.47253991 | -1.980932 | 1.980932 | Down-regulated |
| SLC25A28 | 4.76E-07 | 1.04E-08 | -6.695146 | 9.74255374 | -1.977587 | 1.977587 | Down-regulated |
| THOC7 | 3.34E-13 | 2.06E-16 | -11.47005 | 27.0297734 | -1.977431 | 1.977431 | Down-regulated |
| SQSTM1 | 1.76E-05 | 8.26E-07 | -5.535212 | 5.48768583 | -1.976093 | 1.976093 | Down-regulated |
| VPS26A | 5.45E-12 | 7.75E-15 | -10.446696 | 23.5060881 | -1.972731 | 1.972731 | Down-regulated |
| ALPL | 5.21E-09 | 4.53E-11 | -8.11619 | 15.04883459 | -1.970162 | 1.970162 | Down-regulated |
| FGD3 | 3.41E-05 | 1.86E-06 | -5.314833 | 4.70161671 | -1.968695 | 1.968695 | Down-regulated |
| ACER3 | 5.21E-04 | 5.91E-05 | -4.340595 | 1.3741668 | -1.966842 | 1.966842 | Down-regulated |
| SETD3 | 2.84E-05 | 1.49E-06 | -5.375085 | 4.91555483 | -1.965271 | 1.965271 | Down-regulated |
| CD3E | 1.07E-07 | 1.76E-09 | -7.158636 | 11.47240537 | -1.96323 | 1.96323 | Down-regulated |
| UBASH3B | 9.63E-07 | 2.48E-08 | -6.467128 | 8.89529888 | -1.962021 | 1.962021 | Down-regulated |
| UBAC1 | 1.25E-04 | 9.51E-06 | -4.863889 | 3.12749221 | -1.960004 | 1.960004 | Down-regulated |
| SNX27 | 6.01E-04 | 7.17E-05 | -4.283808 | 1.18956512 | -1.959429 | 1.959429 | Down-regulated |
| SLC35B1 | 2.74E-05 | 1.43E-06 | -5.385951 | 4.95421547 | -1.956744 | 1.956744 | Down-regulated |
| ACSS2 | 2.72E-07 | 5.43E-09 | -6.865086 | 10.37591585 | -1.955481 | 1.955481 | Down-regulated |
| SDHB | 7.84E-08 | 1.21E-09 | -7.257278 | 11.84125324 | -1.954821 | 1.954821 | Down-regulated |
| PFDN5 | 8.02E-05 | 5.38E-06 | -5.02298 | 3.67698847 | -1.954816 | 1.954816 | Down-regulated |
| CTNS | 5.20E-06 | 1.85E-07 | -5.935666 | 6.93812681 | -1.953773 | 1.953773 | Down-regulated |
| RECQL | 3.44E-04 | 3.48E-05 | -4.494655 | 1.88090798 | -1.952867 | 1.952867 | Down-regulated |
| TSSC4 | 3.23E-05 | 1.75E-06 | -5.332319 | 4.76362706 | -1.952813 | 1.952813 | Down-regulated |
| KCNH6 | 3.45E-08 | 4.56E-10 | -7.512025 | 12.79398446 | -1.952724 | 1.952724 | Down-regulated |
| CXCL16 | 1.48E-07 | 2.56E-09 | -7.061036 | 11.10759907 | -1.952706 | 1.952706 | Down-regulated |
| PADI4 | 3.01E-09 | 2.29E-11 | -8.295256 | 15.71439877 | -1.952483 | 1.952483 | Down-regulated |
| PPM1F | 1.65E-06 | 4.73E-08 | -6.297276 | 8.26669558 | -1.950063 | 1.950063 | Down-regulated |
| RFWD2 | 4.48E-08 | 6.14E-10 | -7.434332 | 12.50344266 | -1.949595 | 1.949595 | Down-regulated |
| TCF25 | 2.20E-11 | 5.15E-14 | -9.925724 | 21.66242493 | -1.949316 | 1.949316 | Down-regulated |
| RAPGEF1 | 1.17E-04 | 8.70E-06 | -4.888891 | 3.21338992 | -1.947651 | 1.947651 | Down-regulated |
| YWHAG | 1.40E-05 | 6.21E-07 | -5.612281 | 5.76477958 | -1.946662 | 1.946662 | Down-regulated |
| DYNC1LI2 | 9.53E-05 | 6.71E-06 | -4.961326 | 3.46322625 | -1.945015 | 1.945015 | Down-regulated |
| ITGB2 | 8.68E-10 | 4.84E-12 | -8.705478 | 17.23191441 | -1.944912 | 1.944912 | Down-regulated |
| HEBP2 | 3.02E-05 | 1.61E-06 | -5.353739 | 4.83967516 | -1.943999 | 1.943999 | Down-regulated |
| ATP6V1F | 3.57E-10 | 1.59E-12 | -9.000513 | 18.31574312 | -1.943647 | 1.943647 | Down-regulated |
| RAB31 | 4.45E-14 | 1.19E-17 | -12.301494 | 29.79047766 | -1.941485 | 1.941485 | Down-regulated |
| HIST1H4C | 3.88E-07 | 8.12E-09 | -6.759841 | 9.98351082 | -1.94014 | 1.94014 | Down-regulated |
| MALSU1 | 1.98E-06 | 5.88E-08 | -6.239879 | 8.05486509 | -1.939127 | 1.939127 | Down-regulated |
| GALC | 1.39E-06 | 3.88E-08 | -6.349511 | 8.45974859 | -1.939056 | 1.939056 | Down-regulated |
| TUG1 | 1.01E-12 | 1.07E-15 | -11.00001 | 25.42803509 | -1.938655 | 1.938655 | Down-regulated |
| VMP1 | 2.60E-05 | 1.34E-06 | -5.403902 | 5.01814144 | -1.938633 | 1.938633 | Down-regulated |
| RBM18 | 5.28E-07 | 1.18E-08 | -6.662168 | 9.6198165 | -1.938202 | 1.938202 | Down-regulated |
| MMGT1 | 2.07E-06 | 6.20E-08 | -6.225822 | 8.00303648 | -1.935736 | 1.935736 | Down-regulated |
| P4HB | 1.67E-06 | 4.83E-08 | -6.292006 | 8.24723294 | -1.935571 | 1.935571 | Down-regulated |
| FOXJ2 | 1.10E-06 | 2.90E-08 | -6.426166 | 8.74348519 | -1.935172 | 1.935172 | Down-regulated |
| ITGAM | 5.57E-07 | 1.26E-08 | -6.645719 | 9.55861589 | -1.935139 | 1.935139 | Down-regulated |
| CERS6 | 5.31E-08 | 7.61E-10 | -7.378236 | 12.29364061 | -1.935026 | 1.935026 | Down-regulated |
| TGOLN2 | 1.14E-05 | 4.84E-07 | -5.67908 | 6.00579364 | -1.934901 | 1.934901 | Down-regulated |
| CGGBP1 | 2.97E-05 | 1.58E-06 | -5.359054 | 4.85855906 | -1.934428 | 1.934428 | Down-regulated |
| WDR83OS | 7.33E-10 | 3.91E-12 | -8.762086 | 17.44040247 | -1.93384 | 1.93384 | Down-regulated |
| IFITM1 | 1.35E-05 | 5.94E-07 | -5.624175 | 5.80763872 | -1.933725 | 1.933725 | Down-regulated |
| RPS10 | 2.93E-05 | 1.55E-06 | -5.363842 | 4.87557564 | -1.933467 | 1.933467 | Down-regulated |
| ANKRD13A | 6.69E-05 | 4.28E-06 | -5.086029 | 3.89660102 | -1.932919 | 1.932919 | Down-regulated |
| BOLA2 | 5.00E-11 | 1.47E-13 | -9.641014 | 20.64185323 | -1.931094 | 1.931094 | Down-regulated |
| EMILIN2 | 1.23E-04 | 9.25E-06 | -4.871578 | 3.15389228 | -1.92935 | 1.92935 | Down-regulated |
| HARS | 3.59E-05 | 1.98E-06 | -5.297527 | 4.64031468 | -1.929302 | 1.929302 | Down-regulated |
| ENY2 | 2.77E-05 | 1.45E-06 | -5.382709 | 4.94268011 | -1.928499 | 1.928499 | Down-regulated |
| LSM2 | 9.95E-13 | 1.04E-15 | -11.008839 | 25.45838769 | -1.928216 | 1.928216 | Down-regulated |
| CMTM7 | 5.14E-08 | 7.31E-10 | -7.388743 | 12.33293705 | -1.926917 | 1.926917 | Down-regulated |
| KLHL8 | 6.43E-06 | 2.39E-07 | -5.867933 | 6.69106596 | -1.92643 | 1.92643 | Down-regulated |
| LSM14A | 2.32E-08 | 2.77E-10 | -7.641714 | 13.27881869 | -1.925413 | 1.925413 | Down-regulated |
| NT5C3A | 6.40E-13 | 5.97E-16 | -11.165862 | 25.99651621 | -1.924307 | 1.924307 | Down-regulated |
| GRK2 | 3.65E-10 | 1.66E-12 | -8.989415 | 18.27510358 | -1.922708 | 1.922708 | Down-regulated |
| H2AFZ | 5.42E-08 | 7.77E-10 | -7.372682 | 12.27286594 | -1.920869 | 1.920869 | Down-regulated |
| PPP1R11 | 3.13E-06 | 1.01E-07 | -6.096682 | 7.52785793 | -1.919758 | 1.919758 | Down-regulated |
| DECR1 | 5.31E-05 | 3.22E-06 | -5.164601 | 4.17166907 | -1.91776 | 1.91776 | Down-regulated |
| EIF3M | 1.43E-04 | 1.13E-05 | -4.815978 | 2.96338497 | -1.916569 | 1.916569 | Down-regulated |
| PAPOLA | 1.46E-05 | 6.56E-07 | -5.597446 | 5.71135898 | -1.913816 | 1.913816 | Down-regulated |
| ANXA3 | 3.20E-03 | 7.34E-04 | -3.570342 | -1.01416115 | -1.913317 | 1.913317 | Down-regulated |
| NR1H3 | 3.27E-08 | 4.28E-10 | -7.528229 | 12.85457534 | -1.912805 | 1.912805 | Down-regulated |
| RPA3 | 2.16E-06 | 6.52E-08 | -6.212462 | 7.95379439 | -1.91231 | 1.91231 | Down-regulated |
| HINT3 | 1.90E-09 | 1.30E-11 | -8.445086 | 16.26992719 | -1.912293 | 1.912293 | Down-regulated |
| DAGLB | 7.97E-05 | 5.32E-06 | -5.025662 | 3.68631012 | -1.912106 | 1.912106 | Down-regulated |
| RAB4B | 2.42E-09 | 1.76E-11 | -8.364206 | 15.97021505 | -1.909814 | 1.909814 | Down-regulated |
| TLR5 | 3.11E-10 | 1.34E-12 | -9.046858 | 18.48534056 | -1.909772 | 1.909772 | Down-regulated |
| CAMLG | 1.26E-09 | 7.90E-12 | -8.575625 | 16.75277311 | -1.909657 | 1.909657 | Down-regulated |
| PARL | 2.12E-05 | 1.04E-06 | -5.472562 | 5.26324308 | -1.909268 | 1.909268 | Down-regulated |
| SRP19 | 6.38E-08 | 9.54E-10 | -7.319162 | 12.07269424 | -1.908863 | 1.908863 | Down-regulated |
| DNAJB11 | 1.92E-08 | 2.22E-10 | -7.700018 | 13.49668836 | -1.908831 | 1.908831 | Down-regulated |
| FOXN2 | 1.61E-07 | 2.84E-09 | -7.034793 | 11.00954018 | -1.905918 | 1.905918 | Down-regulated |
| PLEKHA2 | 7.21E-15 | 1.12E-18 | -13.009628 | 32.06688121 | -1.904954 | 1.904954 | Down-regulated |
| RAB3IP | 2.33E-05 | 1.17E-06 | -5.44077 | 5.14963691 | -1.901832 | 1.901832 | Down-regulated |
| TBCA | 1.79E-09 | 1.19E-11 | -8.467609 | 16.35331783 | -1.901398 | 1.901398 | Down-regulated |
| AP1M1 | 3.59E-10 | 1.63E-12 | -8.994779 | 18.29474816 | -1.900381 | 1.900381 | Down-regulated |
| PEX19 | 2.57E-08 | 3.15E-10 | -7.608013 | 13.15285628 | -1.898547 | 1.898547 | Down-regulated |
| ZNF148 | 1.92E-08 | 2.23E-10 | -7.698309 | 13.49030335 | -1.898003 | 1.898003 | Down-regulated |
| RPL35 | 1.58E-07 | 2.76E-09 | -7.041796 | 11.03570563 | -1.897639 | 1.897639 | Down-regulated |
| ADSL | 1.24E-10 | 4.44E-13 | -9.342523 | 19.56274344 | -1.89748 | 1.89748 | Down-regulated |
| TST | 7.55E-08 | 1.16E-09 | -7.268311 | 11.88251385 | -1.897424 | 1.897424 | Down-regulated |
| LNPEP | 1.47E-06 | 4.15E-08 | -6.3318 | 8.3942649 | -1.897322 | 1.897322 | Down-regulated |
| VEZF1 | 4.52E-07 | 9.77E-09 | -6.711484 | 9.80338578 | -1.896524 | 1.896524 | Down-regulated |
| ERO1A | 1.21E-06 | 3.30E-08 | -6.391989 | 8.61691794 | -1.895367 | 1.895367 | Down-regulated |
| LCP1 | 3.93E-05 | 2.21E-06 | -5.268169 | 4.53646528 | -1.894749 | 1.894749 | Down-regulated |
| LASP1 | 1.14E-06 | 3.03E-08 | -6.41484 | 8.7015307 | -1.89469 | 1.89469 | Down-regulated |
| ZDHHC6 | 1.58E-06 | 4.53E-08 | -6.308679 | 8.30881937 | -1.894566 | 1.894566 | Down-regulated |
| TUBA4A | 2.10E-09 | 1.49E-11 | -8.408371 | 16.13392449 | -1.894249 | 1.894249 | Down-regulated |
| NCLN | 4.65E-04 | 5.11E-05 | -4.383043 | 1.51293722 | -1.893968 | 1.893968 | Down-regulated |
| POC1A | 2.78E-04 | 2.62E-05 | -4.576148 | 2.15229997 | -1.893956 | 1.893956 | Down-regulated |
| TOP3A | 6.28E-09 | 5.67E-11 | -8.057204 | 14.82924681 | -1.893003 | 1.893003 | Down-regulated |
| SNRPG | 2.45E-08 | 2.97E-10 | -7.623421 | 13.21044943 | -1.892489 | 1.892489 | Down-regulated |
| STRA13 | 2.26E-04 | 2.04E-05 | -4.648382 | 2.3946944 | -1.89244 | 1.89244 | Down-regulated |
| CLIP1 | 6.06E-10 | 3.07E-12 | -8.826024 | 17.67559138 | -1.891357 | 1.891357 | Down-regulated |
| MAP3K1 | 1.11E-04 | 8.16E-06 | -4.906575 | 3.27425291 | -1.89039 | 1.89039 | Down-regulated |
| LAMP1 | 2.51E-05 | 1.29E-06 | -5.414468 | 5.05579965 | -1.888678 | 1.888678 | Down-regulated |
| COPS3 | 1.15E-05 | 4.92E-07 | -5.675111 | 5.99144935 | -1.888488 | 1.888488 | Down-regulated |
| MPPE1 | 1.34E-03 | 2.08E-04 | -3.965318 | 0.17792089 | -1.888037 | 1.888037 | Down-regulated |
| ZNF586 | 2.48E-09 | 1.81E-11 | -8.356842 | 15.94290405 | -1.887372 | 1.887372 | Down-regulated |
| CEACAM1 | 4.56E-11 | 1.31E-13 | -9.672149 | 20.75388538 | -1.885858 | 1.885858 | Down-regulated |
| FBXO18 | 2.64E-04 | 2.45E-05 | -4.59511 | 2.21576773 | -1.885542 | 1.885542 | Down-regulated |
| GNPDA1 | 1.44E-06 | 4.05E-08 | -6.338072 | 8.41744947 | -1.885395 | 1.885395 | Down-regulated |
| NDUFS3 | 2.18E-07 | 4.09E-09 | -6.93939 | 10.65322735 | -1.884039 | 1.884039 | Down-regulated |
| GALE | 3.03E-08 | 3.88E-10 | -7.553646 | 12.94960849 | -1.883859 | 1.883859 | Down-regulated |
| ARPC5 | 2.38E-04 | 2.17E-05 | -4.630621 | 2.33493846 | -1.883778 | 1.883778 | Down-regulated |
| RAB11A | 3.80E-08 | 5.09E-10 | -7.482873 | 12.68497413 | -1.883507 | 1.883507 | Down-regulated |
| GSDMD | 2.91E-08 | 3.67E-10 | -7.568387 | 13.00472259 | -1.882983 | 1.882983 | Down-regulated |
| TMEM14C | 2.48E-07 | 4.76E-09 | -6.899738 | 10.50521531 | -1.882425 | 1.882425 | Down-regulated |
| MRGBP | 1.26E-06 | 3.43E-08 | -6.381803 | 8.57921539 | -1.882156 | 1.882156 | Down-regulated |
| ZDHHC16 | 4.76E-06 | 1.67E-07 | -5.963351 | 7.03929008 | -1.881926 | 1.881926 | Down-regulated |
| GAPDH | 4.63E-04 | 5.09E-05 | -4.384279 | 1.51698569 | -1.881777 | 1.881777 | Down-regulated |
| MTX1 | 4.54E-07 | 9.85E-09 | -6.709333 | 9.79537491 | -1.881487 | 1.881487 | Down-regulated |
| PAPD5 | 6.65E-09 | 6.09E-11 | -8.038457 | 14.75942151 | -1.881344 | 1.881344 | Down-regulated |
| NPEPL1 | 1.67E-06 | 4.81E-08 | -6.293043 | 8.25106268 | -1.88127 | 1.88127 | Down-regulated |
| BRK1 | 5.20E-03 | 1.61E-03 | -3.312651 | -1.74943664 | -1.880598 | 1.880598 | Down-regulated |
| RRAGD | 4.65E-05 | 2.72E-06 | -5.211146 | 4.33530643 | -1.880424 | 1.880424 | Down-regulated |
| GHITM | 3.31E-04 | 3.31E-05 | -4.509407 | 1.92986999 | -1.880303 | 1.880303 | Down-regulated |
| LILRA2 | 3.28E-06 | 1.06E-07 | -6.082863 | 7.47711901 | -1.880066 | 1.880066 | Down-regulated |
| TMEM127 | 4.53E-05 | 2.63E-06 | -5.219927 | 4.36623689 | -1.879919 | 1.879919 | Down-regulated |
| ARAF | 3.64E-08 | 4.83E-10 | -7.496849 | 12.73723747 | -1.879869 | 1.879869 | Down-regulated |
| COASY | 1.15E-07 | 1.91E-09 | -7.137934 | 11.39501131 | -1.879467 | 1.879467 | Down-regulated |
| NT5C | 4.71E-06 | 1.65E-07 | -5.966968 | 7.05251589 | -1.878354 | 1.878354 | Down-regulated |
| UQCRQ | 1.99E-04 | 1.72E-05 | -4.696057 | 2.55559266 | -1.877249 | 1.877249 | Down-regulated |
| CCND3 | 1.17E-08 | 1.20E-10 | -7.860951 | 14.09760922 | -1.875581 | 1.875581 | Down-regulated |
| S100P | 2.48E-03 | 4.96E-04 | -3.695196 | -0.64543907 | -1.874848 | 1.874848 | Down-regulated |
| SLC12A9 | 2.53E-08 | 3.10E-10 | -7.612928 | 13.17122668 | -1.87451 | 1.87451 | Down-regulated |
| AKIRIN2 | 7.08E-06 | 2.69E-07 | -5.836556 | 6.57683693 | -1.872169 | 1.872169 | Down-regulated |
| ETFB | 4.80E-06 | 1.69E-07 | -5.961066 | 7.0309376 | -1.871171 | 1.871171 | Down-regulated |
| VPS9D1 | 1.99E-07 | 3.67E-09 | -6.967293 | 10.75741074 | -1.87008 | 1.87008 | Down-regulated |
| RBM22 | 1.98E-08 | 2.32E-10 | -7.688357 | 13.45311814 | -1.869121 | 1.869121 | Down-regulated |
| SYT11 | 1.41E-05 | 6.26E-07 | -5.610084 | 5.75686457 | -1.869021 | 1.869021 | Down-regulated |
| LRSAM1 | 3.62E-03 | 8.92E-04 | -3.507441 | -1.1969118 | -1.86827 | 1.86827 | Down-regulated |
| NUDT3 | 5.21E-05 | 3.14E-06 | -5.171628 | 4.19633918 | -1.866712 | 1.866712 | Down-regulated |
| VNN1 | 7.87E-10 | 4.28E-12 | -8.737639 | 17.35039263 | -1.865916 | 1.865916 | Down-regulated |
| WDR48 | 6.93E-11 | 2.16E-13 | -9.537013 | 20.26689762 | -1.865631 | 1.865631 | Down-regulated |
| VPS53 | 5.56E-10 | 2.69E-12 | -8.861072 | 17.8043722 | -1.864738 | 1.864738 | Down-regulated |
| MRPL35 | 3.65E-07 | 7.57E-09 | -6.778162 | 10.05178365 | -1.864578 | 1.864578 | Down-regulated |
| ZFP91 | 1.22E-08 | 1.27E-10 | -7.846354 | 14.04313586 | -1.864344 | 1.864344 | Down-regulated |
| MVB12A | 1.02E-09 | 6.05E-12 | -8.646462 | 17.01430497 | -1.863337 | 1.863337 | Down-regulated |
| PLEKHM2 | 7.90E-11 | 2.62E-13 | -9.484797 | 20.07821856 | -1.86309 | 1.86309 | Down-regulated |
| SLC44A2 | 2.99E-11 | 7.44E-14 | -9.825441 | 21.30396155 | -1.862957 | 1.862957 | Down-regulated |
| TBC1D2 | 8.76E-05 | 6.00E-06 | -4.992611 | 3.57157195 | -1.862793 | 1.862793 | Down-regulated |
| HP | 3.30E-06 | 1.08E-07 | -6.080283 | 7.46765038 | -1.862122 | 1.862122 | Down-regulated |
| BTBD2 | 4.19E-15 | 5.58E-19 | -13.222923 | 32.73893896 | -1.861233 | 1.861233 | Down-regulated |
| LYRM1 | 1.97E-10 | 7.56E-13 | -9.199556 | 19.04278229 | -1.860049 | 1.860049 | Down-regulated |
| FCGR1A | 6.60E-07 | 1.54E-08 | -6.591419 | 9.35671176 | -1.85802 | 1.85802 | Down-regulated |
| TRAPPC3 | 2.15E-05 | 1.06E-06 | -5.467888 | 5.24652685 | -1.857816 | 1.857816 | Down-regulated |
| PPP3CB | 5.83E-06 | 2.13E-07 | -5.899072 | 6.80456945 | -1.857568 | 1.857568 | Down-regulated |
| BAZ1A | 8.63E-07 | 2.17E-08 | -6.502585 | 9.02681692 | -1.85756 | 1.85756 | Down-regulated |
| PACSIN2 | 5.45E-12 | 7.65E-15 | -10.450042 | 23.51782408 | -1.85699 | 1.85699 | Down-regulated |
| LST1 | 2.27E-06 | 6.93E-08 | -6.196683 | 7.89565948 | -1.855923 | 1.855923 | Down-regulated |
| SAP30L | 5.78E-06 | 2.10E-07 | -5.902056 | 6.81545501 | -1.855637 | 1.855637 | Down-regulated |
| OSTF1 | 8.96E-06 | 3.63E-07 | -5.756753 | 6.28696825 | -1.854658 | 1.854658 | Down-regulated |
| C1orf50 | 6.52E-10 | 3.37E-12 | -8.801186 | 17.58426349 | -1.854658 | 1.854658 | Down-regulated |
| CNPY2 | 2.02E-06 | 6.03E-08 | -6.233443 | 8.03113164 | -1.854503 | 1.854503 | Down-regulated |
| ULK1 | 1.56E-08 | 1.69E-10 | -7.771323 | 13.76302701 | -1.854231 | 1.854231 | Down-regulated |
| ARHGEF2 | 2.21E-06 | 6.73E-08 | -6.204168 | 7.92323301 | -1.852227 | 1.852227 | Down-regulated |
| ATP5B | 7.99E-05 | 5.34E-06 | -5.024849 | 3.6834852 | -1.851574 | 1.851574 | Down-regulated |
| SRF | 2.42E-06 | 7.47E-08 | -6.176582 | 7.82163913 | -1.850953 | 1.850953 | Down-regulated |
| GRAMD1A | 4.13E-06 | 1.42E-07 | -6.007311 | 7.20012825 | -1.850914 | 1.850914 | Down-regulated |
| MED16 | 1.52E-09 | 9.68E-12 | -8.52194 | 16.55433541 | -1.850605 | 1.850605 | Down-regulated |
| HSD17B12 | 3.13E-07 | 6.38E-09 | -6.823102 | 10.21932005 | -1.849686 | 1.849686 | Down-regulated |
| ATP8B4 | 7.58E-11 | 2.48E-13 | -9.499703 | 20.13211067 | -1.849443 | 1.849443 | Down-regulated |
| PRDX1 | 1.56E-04 | 1.26E-05 | -4.785606 | 2.85969895 | -1.847433 | 1.847433 | Down-regulated |
| C1orf162 | 4.51E-08 | 6.23E-10 | -7.43036 | 12.48858453 | -1.847208 | 1.847208 | Down-regulated |
| COX5A | 3.01E-10 | 1.28E-12 | -9.059286 | 18.53078823 | -1.846063 | 1.846063 | Down-regulated |
| NSL1 | 1.55E-03 | 2.53E-04 | -3.904395 | -0.01070524 | -1.84557 | 1.84557 | Down-regulated |
| CHCHD1 | 1.98E-08 | 2.32E-10 | -7.688421 | 13.45335875 | -1.841967 | 1.841967 | Down-regulated |
| TMEM59 | 2.10E-04 | 1.85E-05 | -4.675451 | 2.48596292 | -1.840947 | 1.840947 | Down-regulated |
| UBXN1 | 4.13E-06 | 1.41E-07 | -6.008109 | 7.20305232 | -1.840871 | 1.840871 | Down-regulated |
| NAA10 | 9.41E-10 | 5.31E-12 | -8.680755 | 17.1407825 | -1.840281 | 1.840281 | Down-regulated |
| PAQR4 | 2.63E-07 | 5.20E-09 | -6.876569 | 10.41875915 | -1.840105 | 1.840105 | Down-regulated |
| DCUN1D3 | 8.75E-08 | 1.37E-09 | -7.223788 | 11.71601562 | -1.838309 | 1.838309 | Down-regulated |
| CEACAM3 | 1.07E-04 | 7.72E-06 | -4.922094 | 3.32773137 | -1.835541 | 1.835541 | Down-regulated |
| SLC35A5 | 4.75E-05 | 2.79E-06 | -5.20436 | 4.31141999 | -1.833285 | 1.833285 | Down-regulated |
| FAM63A | 3.13E-06 | 1.01E-07 | -6.096724 | 7.5280105 | -1.831833 | 1.831833 | Down-regulated |
| ATF4 | 1.90E-04 | 1.64E-05 | -4.710918 | 2.60589132 | -1.831792 | 1.831792 | Down-regulated |
| ZNF672 | 1.97E-09 | 1.38E-11 | -8.42789 | 16.20623915 | -1.831447 | 1.831447 | Down-regulated |
| TMEM141 | 2.14E-08 | 2.54E-10 | -7.664223 | 13.36293632 | -1.830986 | 1.830986 | Down-regulated |
| DIABLO | 4.28E-06 | 1.47E-07 | -5.99677 | 7.16153969 | -1.830064 | 1.830064 | Down-regulated |
| CCDC28A | 6.23E-08 | 9.23E-10 | -7.327893 | 12.10535034 | -1.829388 | 1.829388 | Down-regulated |
| THOC5 | 4.00E-08 | 5.38E-10 | -7.468696 | 12.63195514 | -1.829377 | 1.829377 | Down-regulated |
| SNX11 | 4.63E-05 | 2.70E-06 | -5.212775 | 4.34104257 | -1.828947 | 1.828947 | Down-regulated |
| HIKESHI | 2.21E-03 | 4.18E-04 | -3.749125 | -0.48379034 | -1.828404 | 1.828404 | Down-regulated |
| NCF4 | 2.79E-08 | 3.47E-10 | -7.583464 | 13.06108629 | -1.828084 | 1.828084 | Down-regulated |
| CNN2 | 5.88E-09 | 5.16E-11 | -8.08198 | 14.92149766 | -1.82807 | 1.82807 | Down-regulated |
| AQP9 | 3.80E-10 | 1.75E-12 | -8.976087 | 18.2262825 | -1.827309 | 1.827309 | Down-regulated |
| CCM2 | 1.55E-11 | 3.39E-14 | -10.039642 | 22.0682629 | -1.826044 | 1.826044 | Down-regulated |
| FAM207A | 3.49E-06 | 1.16E-07 | -6.061126 | 7.3973548 | -1.822921 | 1.822921 | Down-regulated |
| ATP2A3 | 1.66E-07 | 2.96E-09 | -7.023787 | 10.96842112 | -1.822706 | 1.822706 | Down-regulated |
| IFNGR1 | 9.56E-07 | 2.46E-08 | -6.469737 | 8.90497304 | -1.822486 | 1.822486 | Down-regulated |
| SP1 | 3.98E-12 | 5.39E-15 | -10.547184 | 23.85797955 | -1.820268 | 1.820268 | Down-regulated |
| ITPRIP | 1.30E-05 | 5.67E-07 | -5.63679 | 5.85311949 | -1.81949 | 1.81949 | Down-regulated |
| CCDC189 | 1.10E-04 | 8.08E-06 | -4.909306 | 3.283658 | -1.818603 | 1.818603 | Down-regulated |
| PARP10 | 2.09E-06 | 6.25E-08 | -6.223665 | 7.99508258 | -1.817877 | 1.817877 | Down-regulated |
| NCOA3 | 2.59E-10 | 1.05E-12 | -9.112249 | 18.72431261 | -1.817671 | 1.817671 | Down-regulated |
| IFITM2 | 2.20E-11 | 5.19E-14 | -9.923417 | 21.65419371 | -1.816779 | 1.816779 | Down-regulated |
| PSMA4 | 1.53E-05 | 6.99E-07 | -5.580456 | 5.65022663 | -1.815552 | 1.815552 | Down-regulated |
| HIST1H1C | 2.42E-11 | 5.80E-14 | -9.89338 | 21.54693194 | -1.815507 | 1.815507 | Down-regulated |
| CARHSP1 | 2.16E-06 | 6.53E-08 | -6.212297 | 7.95318501 | -1.815191 | 1.815191 | Down-regulated |
| LY96 | 1.70E-05 | 7.92E-07 | -5.546739 | 5.52906232 | -1.815091 | 1.815091 | Down-regulated |
| SCAP | 5.36E-06 | 1.93E-07 | -5.925112 | 6.89959191 | -1.81439 | 1.81439 | Down-regulated |
| CMC2 | 3.86E-03 | 9.88E-04 | -3.474194 | -1.29266932 | -1.813562 | 1.813562 | Down-regulated |
| PET100 | 1.21E-08 | 1.26E-10 | -7.848832 | 14.05238543 | -1.813497 | 1.813497 | Down-regulated |
| CCDC43 | 1.09E-08 | 1.11E-10 | -7.881975 | 14.17605531 | -1.811567 | 1.811567 | Down-regulated |
| USB1 | 5.29E-04 | 6.04E-05 | -4.334345 | 1.35378955 | -1.811444 | 1.811444 | Down-regulated |
| ZNF467 | 1.04E-07 | 1.69E-09 | -7.170162 | 11.51549671 | -1.81139 | 1.81139 | Down-regulated |
| PLCG2 | 6.61E-05 | 4.22E-06 | -5.089845 | 3.90992602 | -1.811135 | 1.811135 | Down-regulated |
| UBE2J1 | 4.75E-07 | 1.03E-08 | -6.696572 | 9.74786251 | -1.810283 | 1.810283 | Down-regulated |
| ZNF511 | 3.77E-08 | 5.05E-10 | -7.48538 | 12.69434831 | -1.810111 | 1.810111 | Down-regulated |
| REPIN1 | 1.47E-07 | 2.54E-09 | -7.063311 | 11.11609855 | -1.809693 | 1.809693 | Down-regulated |
| RAB40C | 1.43E-05 | 6.40E-07 | -5.604073 | 5.7352202 | -1.808442 | 1.808442 | Down-regulated |
| TLN1 | 2.21E-07 | 4.17E-09 | -6.93386 | 10.63258051 | -1.80768 | 1.80768 | Down-regulated |
| UNC50 | 5.82E-13 | 5.30E-16 | -11.199846 | 26.11255556 | -1.807328 | 1.807328 | Down-regulated |
| HMGCR | 5.22E-05 | 3.15E-06 | -5.170614 | 4.19277973 | -1.806466 | 1.806466 | Down-regulated |
| KLHDC3 | 4.39E-11 | 1.25E-13 | -9.684822 | 20.79945802 | -1.806284 | 1.806284 | Down-regulated |
| KCNJ2 | 8.85E-08 | 1.39E-09 | -7.22068 | 11.70439239 | -1.805703 | 1.805703 | Down-regulated |
| PPP1R21 | 4.71E-09 | 3.93E-11 | -8.153486 | 15.18759248 | -1.805688 | 1.805688 | Down-regulated |
| CNST | 3.88E-04 | 4.06E-05 | -4.449886 | 1.73278044 | -1.804177 | 1.804177 | Down-regulated |
| FBXO11 | 7.02E-05 | 4.56E-06 | -5.068523 | 3.83552372 | -1.803773 | 1.803773 | Down-regulated |
| HIST1H2BD | 3.53E-08 | 4.68E-10 | -7.505093 | 12.76806656 | -1.802819 | 1.802819 | Down-regulated |
| CAPZA2 | 1.12E-06 | 2.97E-08 | -6.420008 | 8.72067046 | -1.802325 | 1.802325 | Down-regulated |
| MOSPD2 | 1.16E-06 | 3.12E-08 | -6.406751 | 8.67157406 | -1.801419 | 1.801419 | Down-regulated |
| AKAP13 | 2.46E-03 | 4.92E-04 | -3.697441 | -0.63873766 | -1.800268 | 1.800268 | Down-regulated |
| INAFM1 | 4.76E-07 | 1.04E-08 | -6.694479 | 9.74007206 | -1.800036 | 1.800036 | Down-regulated |
| SPCS2 | 2.88E-07 | 5.77E-09 | -6.849084 | 10.31622004 | -1.798185 | 1.798185 | Down-regulated |
| DHDDS | 7.18E-06 | 2.74E-07 | -5.831394 | 6.55805875 | -1.79752 | 1.79752 | Down-regulated |
| NACC2 | 2.67E-07 | 5.30E-09 | -6.871274 | 10.3989997 | -1.796673 | 1.796673 | Down-regulated |
| TMED1 | 8.28E-11 | 2.76E-13 | -9.47059 | 20.02683663 | -1.796294 | 1.796294 | Down-regulated |
| HPCAL1 | 7.11E-09 | 6.56E-11 | -8.019012 | 14.68698588 | -1.794444 | 1.794444 | Down-regulated |
| LZIC | 2.64E-04 | 2.46E-05 | -4.594639 | 2.21418973 | -1.79435 | 1.79435 | Down-regulated |
| CSF2RA | 6.45E-07 | 1.51E-08 | -6.597946 | 9.38097197 | -1.794166 | 1.794166 | Down-regulated |
| PIAS4 | 5.89E-03 | 2.00E-03 | -3.240154 | -1.94968809 | -1.792018 | 1.792018 | Down-regulated |
| CANX | 3.92E-07 | 8.29E-09 | -6.754572 | 9.96387598 | -1.791293 | 1.791293 | Down-regulated |
| MRPS33 | 1.21E-04 | 9.10E-06 | -4.876003 | 3.16908842 | -1.790359 | 1.790359 | Down-regulated |
| GPR65 | 5.16E-05 | 3.10E-06 | -5.175246 | 4.20904905 | -1.789721 | 1.789721 | Down-regulated |
| MPLKIP | 9.57E-06 | 3.92E-07 | -5.735737 | 6.21079316 | -1.788712 | 1.788712 | Down-regulated |
| DNAJC8 | 2.39E-08 | 2.89E-10 | -7.631073 | 13.23904849 | -1.788145 | 1.788145 | Down-regulated |
| ZBED1 | 5.21E-05 | 3.14E-06 | -5.171524 | 4.19597401 | -1.787721 | 1.787721 | Down-regulated |
| FRMD8 | 2.00E-04 | 1.73E-05 | -4.694506 | 2.550345 | -1.787135 | 1.787135 | Down-regulated |
| TMEM219 | 5.99E-03 | 2.05E-03 | -3.230987 | -1.97479504 | -1.786607 | 1.786607 | Down-regulated |
| RBM14 | 2.58E-12 | 3.10E-15 | -10.701758 | 24.39683157 | -1.786529 | 1.786529 | Down-regulated |
| COQ10A | 3.53E-13 | 2.75E-16 | -11.387131 | 26.74933294 | -1.786481 | 1.786481 | Down-regulated |
| MEA1 | 2.84E-04 | 2.71E-05 | -4.566973 | 2.12163624 | -1.785882 | 1.785882 | Down-regulated |
| TAX1BP1 | 7.10E-06 | 2.70E-07 | -5.835703 | 6.57373447 | -1.785789 | 1.785789 | Down-regulated |
| PHF19 | 4.37E-04 | 4.74E-05 | -4.405184 | 1.58558115 | -1.785689 | 1.785689 | Down-regulated |
| ZNF20 | 1.13E-03 | 1.64E-04 | -4.036547 | 0.40051878 | -1.785384 | 1.785384 | Down-regulated |
| PPT1 | 7.14E-11 | 2.25E-13 | -9.525301 | 20.2246019 | -1.782574 | 1.782574 | Down-regulated |
| CHD9 | 6.82E-07 | 1.61E-08 | -6.580808 | 9.31727839 | -1.782538 | 1.782538 | Down-regulated |
| ZYX | 5.24E-13 | 4.65E-16 | -11.236702 | 26.23823419 | -1.781765 | 1.781765 | Down-regulated |
| MAP7D1 | 2.91E-05 | 1.55E-06 | -5.365348 | 4.8809294 | -1.781389 | 1.781389 | Down-regulated |
| ENO1 | 2.36E-08 | 2.84E-10 | -7.635846 | 13.25688878 | -1.780458 | 1.780458 | Down-regulated |
| TPRG1L | 7.54E-11 | 2.45E-13 | -9.502952 | 20.14385338 | -1.777002 | 1.777002 | Down-regulated |
| CALM3 | 6.19E-04 | 7.45E-05 | -4.272748 | 1.15375235 | -1.776708 | 1.776708 | Down-regulated |
| CMTM3 | 6.27E-07 | 1.45E-08 | -6.607706 | 9.41725412 | -1.77635 | 1.77635 | Down-regulated |
| TOMM6 | 3.28E-06 | 1.07E-07 | -6.082322 | 7.47513383 | -1.775754 | 1.775754 | Down-regulated |
| BRPF3 | 7.53E-10 | 4.05E-12 | -8.75256 | 17.40533307 | -1.775585 | 1.775585 | Down-regulated |
| PHF21A | 2.96E-04 | 2.85E-05 | -4.552514 | 2.07336216 | -1.775002 | 1.775002 | Down-regulated |
| IP6K1 | 2.25E-05 | 1.12E-06 | -5.453601 | 5.19546298 | -1.774941 | 1.774941 | Down-regulated |
| KIAA0556 | 1.65E-06 | 4.73E-08 | -6.29736 | 8.26700839 | -1.774788 | 1.774788 | Down-regulated |
| DBNL | 1.14E-04 | 8.41E-06 | -4.898176 | 3.24533454 | -1.774513 | 1.774513 | Down-regulated |
| APAF1 | 5.53E-08 | 7.95E-10 | -7.366713 | 12.25054267 | -1.773112 | 1.773112 | Down-regulated |
| VPS41 | 1.67E-09 | 1.08E-11 | -8.493957 | 16.45082698 | -1.77261 | 1.77261 | Down-regulated |
| SSBP3 | 1.47E-07 | 2.55E-09 | -7.062836 | 11.11432587 | -1.772485 | 1.772485 | Down-regulated |
| CAPZA1 | 1.21E-06 | 3.28E-08 | -6.393739 | 8.62339605 | -1.772454 | 1.772454 | Down-regulated |
| CPEB3 | 3.19E-11 | 8.12E-14 | -9.801407 | 21.21788545 | -1.771389 | 1.771389 | Down-regulated |
| C16orf72 | 1.80E-04 | 1.52E-05 | -4.731473 | 2.67557295 | -1.770859 | 1.770859 | Down-regulated |
| MIER1 | 6.61E-09 | 6.02E-11 | -8.041485 | 14.77069963 | -1.770833 | 1.770833 | Down-regulated |
| GLMP | 4.05E-05 | 2.30E-06 | -5.257336 | 4.49819273 | -1.769961 | 1.769961 | Down-regulated |
| TMEM179B | 1.93E-09 | 1.32E-11 | -8.439735 | 16.2501092 | -1.769618 | 1.769618 | Down-regulated |
| TRMT1 | 1.29E-05 | 5.60E-07 | -5.640096 | 5.86504572 | -1.769401 | 1.769401 | Down-regulated |
| STAU1 | 1.45E-06 | 4.08E-08 | -6.336302 | 8.41090683 | -1.769109 | 1.769109 | Down-regulated |
| RRBP1 | 3.35E-07 | 6.88E-09 | -6.803024 | 10.14445822 | -1.769097 | 1.769097 | Down-regulated |
| CCDC174 | 5.59E-06 | 2.02E-07 | -5.91313 | 6.85585791 | -1.767985 | 1.767985 | Down-regulated |
| DHX34 | 4.57E-09 | 3.72E-11 | -8.167517 | 15.2397783 | -1.767358 | 1.767358 | Down-regulated |
| USF1 | 8.26E-10 | 4.55E-12 | -8.721828 | 17.29215767 | -1.767129 | 1.767129 | Down-regulated |
| PPP2R5E | 2.89E-06 | 9.14E-08 | -6.123405 | 7.62603606 | -1.766852 | 1.766852 | Down-regulated |
| MFN1 | 6.61E-09 | 6.00E-11 | -8.042189 | 14.77332318 | -1.766775 | 1.766775 | Down-regulated |
| LRRC28 | 7.59E-06 | 2.95E-07 | -5.811919 | 6.48724532 | -1.762778 | 1.762778 | Down-regulated |
| PUF60 | 4.57E-09 | 3.73E-11 | -8.16715 | 15.23841242 | -1.762173 | 1.762173 | Down-regulated |
| SIRPA | 7.85E-05 | 5.23E-06 | -5.030836 | 3.70429585 | -1.761725 | 1.761725 | Down-regulated |
| IFI27L1 | 2.51E-07 | 4.84E-09 | -6.895275 | 10.48856013 | -1.761231 | 1.761231 | Down-regulated |
| DHRS7B | 2.49E-05 | 1.27E-06 | -5.418435 | 5.06994317 | -1.761081 | 1.761081 | Down-regulated |
| RABEPK | 2.90E-07 | 5.84E-09 | -6.84612 | 10.30516524 | -1.761046 | 1.761046 | Down-regulated |
| MED8 | 1.94E-06 | 5.76E-08 | -6.245451 | 8.07541542 | -1.760028 | 1.760028 | Down-regulated |
| CPD | 1.04E-05 | 4.32E-07 | -5.710026 | 6.11769986 | -1.758442 | 1.758442 | Down-regulated |
| ANKRA2 | 7.15E-09 | 6.66E-11 | -8.014771 | 14.67118343 | -1.757087 | 1.757087 | Down-regulated |
| SIRPB1 | 9.49E-05 | 6.67E-06 | -4.963131 | 3.46947182 | -1.756916 | 1.756916 | Down-regulated |
| OPLAH | 1.24E-09 | 7.69E-12 | -8.582961 | 16.77987402 | -1.75649 | 1.75649 | Down-regulated |
| PSMD8 | 5.02E-10 | 2.37E-12 | -8.894768 | 17.92809177 | -1.756172 | 1.756172 | Down-regulated |
| UNC119 | 9.97E-08 | 1.60E-09 | -7.184352 | 11.56855458 | -1.755928 | 1.755928 | Down-regulated |
| ZNHIT3 | 4.40E-04 | 4.77E-05 | -4.402949 | 1.57823919 | -1.755286 | 1.755286 | Down-regulated |
| BLVRA | 1.37E-06 | 3.83E-08 | -6.352917 | 8.47234331 | -1.754742 | 1.754742 | Down-regulated |
| IER3 | 1.81E-05 | 8.57E-07 | -5.525285 | 5.45207183 | -1.754675 | 1.754675 | Down-regulated |
| DNAJA2 | 5.40E-05 | 3.29E-06 | -5.158671 | 4.15085602 | -1.754507 | 1.754507 | Down-regulated |
| LILRB3 | 7.28E-05 | 4.78E-06 | -5.055535 | 3.79025865 | -1.754452 | 1.754452 | Down-regulated |
| KIF1B | 1.09E-06 | 2.87E-08 | -6.428601 | 8.75250357 | -1.754407 | 1.754407 | Down-regulated |
| FAM107B | 3.95E-06 | 1.34E-07 | -6.021258 | 7.25121019 | -1.754277 | 1.754277 | Down-regulated |
| MANBAL | 5.23E-04 | 5.95E-05 | -4.338859 | 1.36850484 | -1.752362 | 1.752362 | Down-regulated |
| SIVA1 | 1.81E-09 | 1.21E-11 | -8.462814 | 16.33556755 | -1.752097 | 1.752097 | Down-regulated |
| EGLN2 | 1.73E-07 | 3.11E-09 | -7.010404 | 10.91842683 | -1.751911 | 1.751911 | Down-regulated |
| FGR | 1.12E-09 | 6.75E-12 | -8.617324 | 16.90676888 | -1.751786 | 1.751786 | Down-regulated |
| RGS19 | 2.07E-09 | 1.46E-11 | -8.414022 | 16.15486077 | -1.750864 | 1.750864 | Down-regulated |
| TMEM41B | 2.41E-08 | 2.91E-10 | -7.6288 | 13.23055428 | -1.75081 | 1.75081 | Down-regulated |
| STOML2 | 1.69E-04 | 1.40E-05 | -4.755256 | 2.75636029 | -1.749527 | 1.749527 | Down-regulated |
| TMX1 | 5.37E-09 | 4.69E-11 | -8.107089 | 15.01496478 | -1.747727 | 1.747727 | Down-regulated |
| GLIPR1 | 1.86E-10 | 7.09E-13 | -9.216715 | 19.10528945 | -1.747117 | 1.747117 | Down-regulated |
| FAM111A | 8.47E-12 | 1.40E-14 | -10.283146 | 22.93074419 | -1.747088 | 1.747088 | Down-regulated |
| NFKBIZ | 1.51E-05 | 6.83E-07 | -5.586825 | 5.67313726 | -1.746737 | 1.746737 | Down-regulated |
| TMEM216 | 7.76E-04 | 1.00E-04 | -4.185128 | 0.87171987 | -1.74592 | 1.74592 | Down-regulated |
| CTRC | 8.37E-09 | 8.07E-11 | -7.96462 | 14.48427539 | -1.745672 | 1.745672 | Down-regulated |
| C1orf122 | 4.24E-05 | 2.43E-06 | -5.241357 | 4.4417898 | -1.745491 | 1.745491 | Down-regulated |
| FADD | 4.95E-13 | 4.29E-16 | -11.25993 | 26.3173491 | -1.745051 | 1.745051 | Down-regulated |
| ZDHHC7 | 3.85E-07 | 8.02E-09 | -6.762945 | 9.99507586 | -1.745048 | 1.745048 | Down-regulated |
| LDLR | 3.29E-08 | 4.32E-10 | -7.525988 | 12.84619526 | -1.742699 | 1.742699 | Down-regulated |
| ACTN1 | 1.01E-10 | 3.44E-13 | -9.411257 | 19.81202692 | -1.742327 | 1.742327 | Down-regulated |
| SUCLG1 | 1.31E-06 | 3.59E-08 | -6.369671 | 8.53432142 | -1.740684 | 1.740684 | Down-regulated |
| DENND5A | 1.26E-06 | 3.44E-08 | -6.380873 | 8.57577434 | -1.74064 | 1.74064 | Down-regulated |
| GMPR2 | 8.67E-07 | 2.18E-08 | -6.500825 | 9.02028792 | -1.740358 | 1.740358 | Down-regulated |
| EBLN2 | 1.21E-05 | 5.22E-07 | -5.65919 | 5.93394949 | -1.74035 | 1.74035 | Down-regulated |
| SLC39A3 | 2.06E-05 | 1.00E-06 | -5.482689 | 5.29947063 | -1.740058 | 1.740058 | Down-regulated |
| S100A11 | 3.85E-04 | 4.02E-05 | -4.453038 | 1.74318723 | -1.740024 | 1.740024 | Down-regulated |
| ACTR1A | 4.49E-08 | 6.17E-10 | -7.432979 | 12.4983828 | -1.739277 | 1.739277 | Down-regulated |
| RAB11FIP1 | 4.07E-06 | 1.39E-07 | -6.012369 | 7.21865321 | -1.739166 | 1.739166 | Down-regulated |
| CTSH | 1.07E-09 | 6.40E-12 | -8.631366 | 16.95859953 | -1.738745 | 1.738745 | Down-regulated |
| NUMB | 2.82E-10 | 1.17E-12 | -9.082681 | 18.61630335 | -1.738573 | 1.738573 | Down-regulated |
| RRP7A | 3.54E-05 | 1.95E-06 | -5.302158 | 4.65671033 | -1.738237 | 1.738237 | Down-regulated |
| COX15 | 4.44E-09 | 3.60E-11 | -8.176177 | 15.27198276 | -1.737886 | 1.737886 | Down-regulated |
| UBAC2 | 3.09E-08 | 3.99E-10 | -7.546813 | 12.92406255 | -1.737849 | 1.737849 | Down-regulated |
| PQLC1 | 6.46E-05 | 4.11E-06 | -5.097602 | 3.93702204 | -1.737735 | 1.737735 | Down-regulated |
| MGEA5 | 1.06E-06 | 2.80E-08 | -6.435628 | 8.77853852 | -1.736987 | 1.736987 | Down-regulated |
| CETN2 | 1.09E-10 | 3.89E-13 | -9.378178 | 19.69211487 | -1.735489 | 1.735489 | Down-regulated |
| FKBP5 | 1.52E-08 | 1.65E-10 | -7.777928 | 13.78769226 | -1.735377 | 1.735377 | Down-regulated |
| HGS | 4.48E-04 | 4.88E-05 | -4.396313 | 1.55645504 | -1.735163 | 1.735163 | Down-regulated |
| SRA1 | 5.09E-10 | 2.43E-12 | -8.8879 | 17.90288441 | -1.735083 | 1.735083 | Down-regulated |
| MMP25 | 2.35E-05 | 1.18E-06 | -5.438292 | 5.14078812 | -1.734937 | 1.734937 | Down-regulated |
| G6PD | 9.90E-10 | 5.78E-12 | -8.65836 | 17.05819413 | -1.734738 | 1.734738 | Down-regulated |
| HAUS4 | 4.46E-07 | 9.57E-09 | -6.716802 | 9.82318806 | -1.733137 | 1.733137 | Down-regulated |
| COQ5 | 4.65E-07 | 1.01E-08 | -6.70215 | 9.76862828 | -1.731281 | 1.731281 | Down-regulated |
| ZMAT2 | 1.07E-06 | 2.83E-08 | -6.432608 | 8.76734918 | -1.730129 | 1.730129 | Down-regulated |
| LLPH | 1.88E-08 | 2.16E-10 | -7.706429 | 13.52063848 | -1.729971 | 1.729971 | Down-regulated |
| DGUOK | 2.97E-04 | 2.86E-05 | -4.550883 | 2.06792265 | -1.729445 | 1.729445 | Down-regulated |
| SMAP2 | 1.76E-06 | 5.11E-08 | -6.276956 | 8.19166524 | -1.728609 | 1.728609 | Down-regulated |
| C3AR1 | 4.82E-08 | 6.69E-10 | -7.411722 | 12.41887915 | -1.728167 | 1.728167 | Down-regulated |
| CLEC4A | 2.64E-05 | 1.37E-06 | -5.398743 | 4.99976185 | -1.727757 | 1.727757 | Down-regulated |
| NCKAP1L | 1.19E-06 | 3.22E-08 | -6.398605 | 8.64141194 | -1.727211 | 1.727211 | Down-regulated |
| CSF3R | 1.79E-09 | 1.20E-11 | -8.466355 | 16.34867303 | -1.727026 | 1.727026 | Down-regulated |
| SLC16A5 | 1.41E-08 | 1.49E-10 | -7.804273 | 13.88605986 | -1.72685 | 1.72685 | Down-regulated |
| SELO | 2.21E-06 | 6.72E-08 | -6.204755 | 7.92539481 | -1.726178 | 1.726178 | Down-regulated |
| SNRPB2 | 2.33E-06 | 7.16E-08 | -6.18788 | 7.86323936 | -1.724852 | 1.724852 | Down-regulated |
| ATP6V0A1 | 2.04E-03 | 3.74E-04 | -3.784078 | -0.37827722 | -1.724829 | 1.724829 | Down-regulated |
| DCAF10 | 6.57E-08 | 9.86E-10 | -7.310433 | 12.04004895 | -1.724759 | 1.724759 | Down-regulated |
| TNNI2 | 3.72E-07 | 7.76E-09 | -6.771795 | 10.02805576 | -1.724051 | 1.724051 | Down-regulated |
| RYBP | 9.83E-12 | 1.70E-14 | -10.228986 | 22.73951497 | -1.723954 | 1.723954 | Down-regulated |
| SLC35A1 | 5.76E-06 | 2.09E-07 | -5.903526 | 6.82081679 | -1.723728 | 1.723728 | Down-regulated |
| VNN2 | 3.67E-04 | 3.78E-05 | -4.470546 | 1.80105189 | -1.723355 | 1.723355 | Down-regulated |
| ARF4 | 1.02E-08 | 1.02E-10 | -7.903009 | 14.25452518 | -1.723229 | 1.723229 | Down-regulated |
| MRPL21 | 1.25E-05 | 5.39E-07 | -5.650559 | 5.90279436 | -1.72267 | 1.72267 | Down-regulated |
| RARS | 1.03E-10 | 3.58E-13 | -9.399935 | 19.77099706 | -1.722237 | 1.722237 | Down-regulated |
| SRSF6 | 5.67E-07 | 1.28E-08 | -6.639758 | 9.53644392 | -1.721928 | 1.721928 | Down-regulated |
| SEPHS2 | 4.47E-05 | 2.59E-06 | -5.22436 | 4.3818571 | -1.72178 | 1.72178 | Down-regulated |
| CTSB | 8.47E-09 | 8.22E-11 | -7.959785 | 14.46625089 | -1.721463 | 1.721463 | Down-regulated |
| VDAC3 | 9.42E-06 | 3.84E-07 | -5.741052 | 6.23005104 | -1.720407 | 1.720407 | Down-regulated |
| C8orf33 | 1.29E-07 | 2.18E-09 | -7.10321 | 11.26521415 | -1.71979 | 1.71979 | Down-regulated |
| WDR33 | 6.35E-10 | 3.24E-12 | -8.811595 | 17.62254172 | -1.719718 | 1.719718 | Down-regulated |
| RSPRY1 | 1.51E-11 | 3.19E-14 | -10.056407 | 22.12786451 | -1.719062 | 1.719062 | Down-regulated |
| RPS29 | 1.26E-11 | 2.41E-14 | -10.133398 | 22.40116203 | -1.718923 | 1.718923 | Down-regulated |
| COX7B | 1.20E-06 | 3.27E-08 | -6.394631 | 8.62669811 | -1.718558 | 1.718558 | Down-regulated |
| AP5M1 | 4.08E-07 | 8.65E-09 | -6.743439 | 9.92239925 | -1.718308 | 1.718308 | Down-regulated |
| ARPP19 | 1.19E-06 | 3.23E-08 | -6.397897 | 8.63878893 | -1.717915 | 1.717915 | Down-regulated |
| SNRPB | 1.04E-08 | 1.05E-10 | -7.895097 | 14.22501221 | -1.717812 | 1.717812 | Down-regulated |
| MTPN | 1.71E-03 | 2.91E-04 | -3.86163 | -0.14211516 | -1.717736 | 1.717736 | Down-regulated |
| FCN1 | 8.64E-10 | 4.78E-12 | -8.708596 | 17.2434053 | -1.7174 | 1.7174 | Down-regulated |
| LOC105274304 | 1.19E-05 | 5.12E-07 | -5.664086 | 5.95162776 | -1.717046 | 1.717046 | Down-regulated |
| MED31 | 1.71E-05 | 7.98E-07 | -5.544708 | 5.52177135 | -1.715845 | 1.715845 | Down-regulated |
| RSBN1 | 2.77E-08 | 3.44E-10 | -7.585772 | 13.06971645 | -1.715833 | 1.715833 | Down-regulated |
| MRPL28 | 4.57E-06 | 1.59E-07 | -5.976433 | 7.08713 | -1.714824 | 1.714824 | Down-regulated |
| MED10 | 1.39E-11 | 2.80E-14 | -10.092479 | 22.25599649 | -1.714779 | 1.714779 | Down-regulated |
| NIPA2 | 3.37E-06 | 1.11E-07 | -6.072335 | 7.43847892 | -1.714542 | 1.714542 | Down-regulated |
| MRPL37 | 2.21E-06 | 6.73E-08 | -6.204253 | 7.92354752 | -1.714473 | 1.714473 | Down-regulated |
| VPS37A | 8.29E-06 | 3.28E-07 | -5.783282 | 6.3832231 | -1.714306 | 1.714306 | Down-regulated |
| FLJ20021 | 1.56E-06 | 4.47E-08 | -6.312073 | 8.32136043 | -1.714131 | 1.714131 | Down-regulated |
| CEPT1 | 5.28E-11 | 1.57E-13 | -9.622568 | 20.57543284 | -1.713986 | 1.713986 | Down-regulated |
| COL4A3BP | 3.18E-05 | 1.72E-06 | -5.336762 | 4.77939299 | -1.711903 | 1.711903 | Down-regulated |
| MYBPC3 | 2.10E-06 | 6.30E-08 | -6.22168 | 7.98776829 | -1.710966 | 1.710966 | Down-regulated |
| MYL12A | 9.83E-04 | 1.37E-04 | -4.091157 | 0.57264538 | -1.708917 | 1.708917 | Down-regulated |
| PEX16 | 1.80E-08 | 2.06E-10 | -7.719959 | 13.57118396 | -1.708567 | 1.708567 | Down-regulated |
| VAMP5 | 6.50E-10 | 3.35E-12 | -8.802963 | 17.59079824 | -1.708336 | 1.708336 | Down-regulated |
| DGAT1 | 4.30E-09 | 3.44E-11 | -8.188529 | 15.31790919 | -1.708246 | 1.708246 | Down-regulated |
| AVL9 | 9.17E-04 | 1.25E-04 | -4.119281 | 0.66177563 | -1.708216 | 1.708216 | Down-regulated |
| MEPCE | 1.08E-08 | 1.10E-10 | -7.884272 | 14.18462594 | -1.708081 | 1.708081 | Down-regulated |
| NT5C2 | 1.16E-06 | 3.11E-08 | -6.407573 | 8.67461751 | -1.706117 | 1.706117 | Down-regulated |
| E2F6 | 1.76E-10 | 6.64E-13 | -9.234547 | 19.17022012 | -1.706029 | 1.706029 | Down-regulated |
| RNU6-1 | 2.50E-07 | 4.82E-09 | -6.896035 | 10.49139388 | -1.70557 | 1.70557 | Down-regulated |
| ANXA1 | 4.00E-05 | 2.26E-06 | -5.261897 | 4.51430271 | -1.705198 | 1.705198 | Down-regulated |
| PIK3CG | 4.70E-06 | 1.64E-07 | -5.967576 | 7.05473969 | -1.704729 | 1.704729 | Down-regulated |
| CHUK | 1.08E-06 | 2.85E-08 | -6.430246 | 8.75859783 | -1.704473 | 1.704473 | Down-regulated |
| DUSP22 | 6.77E-04 | 8.38E-05 | -4.237657 | 1.0404419 | -1.704381 | 1.704381 | Down-regulated |
| TMEM38B | 3.90E-08 | 5.24E-10 | -7.475581 | 12.65770389 | -1.703457 | 1.703457 | Down-regulated |
| ZNF593 | 5.22E-06 | 1.86E-07 | -5.934475 | 6.9337774 | -1.703313 | 1.703313 | Down-regulated |
| ABHD8 | 1.69E-03 | 2.87E-04 | -3.866479 | -0.12725823 | -1.703022 | 1.703022 | Down-regulated |
| KATNB1 | 7.35E-07 | 1.77E-08 | -6.556158 | 9.22570408 | -1.701896 | 1.701896 | Down-regulated |
| ARID3B | 9.37E-05 | 6.56E-06 | -4.967657 | 3.48513032 | -1.701809 | 1.701809 | Down-regulated |
| ARG1 | 1.64E-05 | 7.58E-07 | -5.558426 | 5.57103604 | -1.701288 | 1.701288 | Down-regulated |
| UROS | 1.00E-04 | 7.16E-06 | -4.943179 | 3.40050062 | -1.700529 | 1.700529 | Down-regulated |
| SLC8B1 | 2.12E-05 | 1.04E-06 | -5.474087 | 5.268697 | -1.700441 | 1.700441 | Down-regulated |
| SMIM4 | 3.93E-04 | 4.14E-05 | -4.444764 | 1.71588061 | -1.700244 | 1.700244 | Down-regulated |
| UIMC1 | 7.16E-05 | 4.68E-06 | -5.061687 | 3.81169379 | -1.700235 | 1.700235 | Down-regulated |
| MLKL | 4.92E-08 | 6.89E-10 | -7.404116 | 12.39043323 | -1.699721 | 1.699721 | Down-regulated |
| FBXO22 | 7.92E-12 | 1.27E-14 | -10.310556 | 23.02739515 | -1.699175 | 1.699175 | Down-regulated |
| MTHFD2 | 1.64E-05 | 7.59E-07 | -5.558165 | 5.57009918 | -1.699136 | 1.699136 | Down-regulated |
| HSPBAP1 | 8.70E-07 | 2.19E-08 | -6.499406 | 9.01502068 | -1.697753 | 1.697753 | Down-regulated |
| AGPS | 2.86E-03 | 6.15E-04 | -3.627191 | -0.84724124 | -1.696806 | 1.696806 | Down-regulated |
| NFE2 | 2.62E-07 | 5.15E-09 | -6.878842 | 10.42723713 | -1.696774 | 1.696774 | Down-regulated |
| CASP3 | 3.20E-08 | 4.15E-10 | -7.536251 | 12.88457213 | -1.696112 | 1.696112 | Down-regulated |
| MT1F | 1.77E-08 | 1.99E-10 | -7.728601 | 13.60346735 | -1.694931 | 1.694931 | Down-regulated |
| CLEC5A | 4.45E-10 | 2.07E-12 | -8.931087 | 18.06133886 | -1.694482 | 1.694482 | Down-regulated |
| NELFE | 7.73E-05 | 5.14E-06 | -5.03552 | 3.72058561 | -1.694445 | 1.694445 | Down-regulated |
| NIPBL | 2.36E-05 | 1.19E-06 | -5.436593 | 5.13472582 | -1.693225 | 1.693225 | Down-regulated |
| PTEN | 2.71E-07 | 5.41E-09 | -6.866172 | 10.37996657 | -1.692155 | 1.692155 | Down-regulated |
| RPS7 | 2.08E-03 | 3.83E-04 | -3.776337 | -0.40169515 | -1.692021 | 1.692021 | Down-regulated |
| MGST2 | 1.47E-04 | 1.16E-05 | -4.808382 | 2.93742768 | -1.691444 | 1.691444 | Down-regulated |
| BAG5 | 3.19E-06 | 1.03E-07 | -6.091124 | 7.5074484 | -1.690898 | 1.690898 | Down-regulated |
| SNHG9 | 6.17E-12 | 9.04E-15 | -10.403742 | 23.35529538 | -1.690568 | 1.690568 | Down-regulated |
| ARSB | 2.63E-05 | 1.36E-06 | -5.400314 | 5.00535984 | -1.690385 | 1.690385 | Down-regulated |
| EFR3A | 1.63E-03 | 2.72E-04 | -3.882616 | -0.07773439 | -1.690141 | 1.690141 | Down-regulated |
| MKLN1 | 6.94E-08 | 1.05E-09 | -7.294077 | 11.97887547 | -1.690058 | 1.690058 | Down-regulated |
| CDK5RAP2 | 2.96E-06 | 9.40E-08 | -6.115962 | 7.5986813 | -1.690031 | 1.690031 | Down-regulated |
| HDDC3 | 6.04E-03 | 2.08E-03 | -3.226133 | -1.98806814 | -1.689242 | 1.689242 | Down-regulated |
| NDUFAF1 | 3.20E-05 | 1.73E-06 | -5.335333 | 4.77431993 | -1.688762 | 1.688762 | Down-regulated |
| ST3GAL6 | 9.00E-10 | 5.06E-12 | -8.693589 | 17.18809484 | -1.688618 | 1.688618 | Down-regulated |
| MAP4K2 | 4.16E-08 | 5.65E-10 | -7.45585 | 12.58391456 | -1.688501 | 1.688501 | Down-regulated |
| KAT8 | 1.17E-11 | 2.10E-14 | -10.170957 | 22.53423615 | -1.688434 | 1.688434 | Down-regulated |
| ANKRD22 | 1.03E-03 | 1.45E-04 | -4.073904 | 0.51813029 | -1.688352 | 1.688352 | Down-regulated |
| PLAUR | 1.30E-03 | 2.01E-04 | -3.975899 | 0.21085058 | -1.686857 | 1.686857 | Down-regulated |
| CYB5R3 | 6.80E-04 | 8.45E-05 | -4.235197 | 1.03251487 | -1.684827 | 1.684827 | Down-regulated |
| TMEM50A | 7.72E-06 | 3.01E-07 | -5.806849 | 6.46882033 | -1.684473 | 1.684473 | Down-regulated |
| SSR4 | 2.73E-08 | 3.38E-10 | -7.589854 | 13.08497543 | -1.684447 | 1.684447 | Down-regulated |
| C4orf3 | 2.12E-10 | 8.23E-13 | -9.176815 | 18.95989591 | -1.684305 | 1.684305 | Down-regulated |
| HTATIP2 | 3.59E-10 | 1.62E-12 | -8.996521 | 18.30112628 | -1.68371 | 1.68371 | Down-regulated |
| ABR | 6.16E-05 | 3.88E-06 | -5.11334 | 3.99204197 | -1.683151 | 1.683151 | Down-regulated |
| SMUG1 | 5.07E-09 | 4.31E-11 | -8.128758 | 15.09560229 | -1.682856 | 1.682856 | Down-regulated |
| TACC1 | 7.11E-05 | 4.63E-06 | -5.064378 | 3.8210739 | -1.682485 | 1.682485 | Down-regulated |
| DDA1 | 7.88E-07 | 1.94E-08 | -6.532029 | 9.13610033 | -1.681888 | 1.681888 | Down-regulated |
| AP2S1 | 7.04E-12 | 1.08E-14 | -10.355093 | 23.18424076 | -1.681168 | 1.681168 | Down-regulated |
| PHKA2 | 2.98E-06 | 9.51E-08 | -6.112738 | 7.58683785 | -1.680708 | 1.680708 | Down-regulated |
| C9orf106 | 5.92E-05 | 3.67E-06 | -5.128661 | 4.04566206 | -1.680506 | 1.680506 | Down-regulated |
| BRAT1 | 2.16E-08 | 2.58E-10 | -7.660352 | 13.34847275 | -1.680093 | 1.680093 | Down-regulated |
| SIK3 | 1.41E-12 | 1.54E-15 | -10.898504 | 25.07835305 | -1.679964 | 1.679964 | Down-regulated |
| DIP2B | 4.27E-07 | 9.12E-09 | -6.729501 | 9.87048093 | -1.679268 | 1.679268 | Down-regulated |
| LILRB1 | 1.09E-07 | 1.80E-09 | -7.153896 | 11.45468535 | -1.678982 | 1.678982 | Down-regulated |
| BST1 | 8.26E-10 | 4.51E-12 | -8.723859 | 17.29963974 | -1.676678 | 1.676678 | Down-regulated |
| COPA | 2.45E-07 | 4.70E-09 | -6.90305 | 10.51757733 | -1.676442 | 1.676442 | Down-regulated |
| SPDYE8P | 6.04E-06 | 2.21E-07 | -5.888751 | 6.766934 | -1.675885 | 1.675885 | Down-regulated |
| DEF8 | 5.64E-04 | 6.57E-05 | -4.309375 | 1.27252539 | -1.675821 | 1.675821 | Down-regulated |
| PEX5 | 9.73E-07 | 2.52E-08 | -6.463205 | 8.88075442 | -1.675675 | 1.675675 | Down-regulated |
| HAVCR2 | 6.77E-07 | 1.59E-08 | -6.583072 | 9.32569219 | -1.67498 | 1.67498 | Down-regulated |
| ARPC2 | 5.92E-04 | 7.03E-05 | -4.289723 | 1.20873506 | -1.672915 | 1.672915 | Down-regulated |
| ALG13 | 2.62E-07 | 5.14E-09 | -6.879534 | 10.42982008 | -1.67286 | 1.67286 | Down-regulated |
| MAPK1 | 2.29E-13 | 1.22E-16 | -11.620838 | 27.53740231 | -1.672727 | 1.672727 | Down-regulated |
| EFHD2 | 8.74E-06 | 3.51E-07 | -5.765197 | 6.31759498 | -1.672348 | 1.672348 | Down-regulated |
| ATP6V1A | 4.95E-08 | 6.95E-10 | -7.401753 | 12.38159674 | -1.671823 | 1.671823 | Down-regulated |
| ZDHHC5 | 2.12E-08 | 2.51E-10 | -7.668024 | 13.37714347 | -1.671136 | 1.671136 | Down-regulated |
| NUFIP2 | 1.26E-06 | 3.44E-08 | -6.381324 | 8.57744097 | -1.671046 | 1.671046 | Down-regulated |
| USP9X | 3.68E-04 | 3.79E-05 | -4.469713 | 1.79829597 | -1.670717 | 1.670717 | Down-regulated |
| PARK7 | 1.90E-09 | 1.29E-11 | -8.446639 | 16.27567783 | -1.669887 | 1.669887 | Down-regulated |
| LAMTOR5 | 1.29E-04 | 9.86E-06 | -4.853531 | 3.0919594 | -1.669237 | 1.669237 | Down-regulated |
| EZR | 6.63E-07 | 1.55E-08 | -6.590232 | 9.35230039 | -1.668994 | 1.668994 | Down-regulated |
| HIST1H4K | 2.79E-07 | 5.56E-09 | -6.858798 | 10.3524554 | -1.668783 | 1.668783 | Down-regulated |
| CDCA4 | 1.04E-11 | 1.82E-14 | -10.209981 | 22.67232918 | -1.667063 | 1.667063 | Down-regulated |
| RPL18A | 4.29E-10 | 1.98E-12 | -8.94261 | 18.10359413 | -1.666707 | 1.666707 | Down-regulated |
| RPRD1A | 3.46E-08 | 4.57E-10 | -7.511226 | 12.79099733 | -1.666599 | 1.666599 | Down-regulated |
| DPM3 | 6.70E-06 | 2.52E-07 | -5.853984 | 6.64026596 | -1.665982 | 1.665982 | Down-regulated |
| UVSSA | 1.73E-04 | 1.45E-05 | -4.744929 | 2.72125855 | -1.663684 | 1.663684 | Down-regulated |
| FIG4 | 1.73E-06 | 5.03E-08 | -6.281091 | 8.20693031 | -1.663499 | 1.663499 | Down-regulated |
| CPSF1 | 3.30E-06 | 1.08E-07 | -6.080129 | 7.46708471 | -1.663469 | 1.663469 | Down-regulated |
| ARL6IP5 | 1.66E-07 | 2.96E-09 | -7.023557 | 10.96756216 | -1.662589 | 1.662589 | Down-regulated |
| DDIAS | 1.65E-08 | 1.82E-10 | -7.752277 | 13.69189914 | -1.66224 | 1.66224 | Down-regulated |
| P2RX1 | 2.52E-09 | 1.85E-11 | -8.350706 | 15.92015045 | -1.661765 | 1.661765 | Down-regulated |
| SPDYE2B | 8.50E-05 | 5.76E-06 | -5.003789 | 3.61034404 | -1.661639 | 1.661639 | Down-regulated |
| PEX11B | 1.72E-06 | 4.98E-08 | -6.283791 | 8.21690137 | -1.660516 | 1.660516 | Down-regulated |
| C10orf54 | 7.77E-08 | 1.19E-09 | -7.260519 | 11.85337328 | -1.660146 | 1.660146 | Down-regulated |
| SHCBP1 | 1.96E-09 | 1.36E-11 | -8.432641 | 16.22383439 | -1.659956 | 1.659956 | Down-regulated |
| CEBPB | 2.20E-05 | 1.09E-06 | -5.460658 | 5.22068008 | -1.659145 | 1.659145 | Down-regulated |
| MEI1 | 6.57E-11 | 2.01E-13 | -9.555263 | 20.33277391 | -1.658648 | 1.658648 | Down-regulated |
| NDUFAF3 | 6.38E-05 | 4.05E-06 | -5.101713 | 3.95138866 | -1.658173 | 1.658173 | Down-regulated |
| HEXIM2 | 1.05E-04 | 7.56E-06 | -4.928039 | 3.3482379 | -1.657919 | 1.657919 | Down-regulated |
| MRRF | 3.53E-06 | 1.17E-07 | -6.05752 | 7.38412984 | -1.657818 | 1.657818 | Down-regulated |
| CCNG1 | 1.88E-09 | 1.27E-11 | -8.45116 | 16.29241725 | -1.657731 | 1.657731 | Down-regulated |
| PDCD7 | 6.76E-07 | 1.59E-08 | -6.584241 | 9.33003868 | -1.65736 | 1.65736 | Down-regulated |
| EXO5 | 4.13E-11 | 1.16E-13 | -9.70541 | 20.87345678 | -1.657189 | 1.657189 | Down-regulated |
| PDLIM7 | 1.03E-07 | 1.67E-09 | -7.173551 | 11.52816839 | -1.657162 | 1.657162 | Down-regulated |
| STRADA | 6.51E-04 | 7.94E-05 | -4.253599 | 1.09185977 | -1.656979 | 1.656979 | Down-regulated |
| LOC146880 | 1.35E-08 | 1.41E-10 | -7.817898 | 13.93692379 | -1.656041 | 1.656041 | Down-regulated |
| CYTH4 | 1.26E-07 | 2.13E-09 | -7.10928 | 11.28790231 | -1.655922 | 1.655922 | Down-regulated |
| IDH1 | 2.64E-05 | 1.37E-06 | -5.399083 | 5.00097382 | -1.654321 | 1.654321 | Down-regulated |
| PGAP2 | 1.11E-07 | 1.85E-09 | -7.146361 | 11.42651619 | -1.652823 | 1.652823 | Down-regulated |
| MOB3A | 2.80E-07 | 5.59E-09 | -6.857401 | 10.34724428 | -1.652206 | 1.652206 | Down-regulated |
| HECTD3 | 7.44E-04 | 9.49E-05 | -4.201046 | 0.92273401 | -1.650987 | 1.650987 | Down-regulated |
| GAK | 3.35E-06 | 1.10E-07 | -6.074397 | 7.44604761 | -1.650797 | 1.650797 | Down-regulated |
| BAZ2B | 2.25E-04 | 2.02E-05 | -4.650268 | 2.40104553 | -1.650426 | 1.650426 | Down-regulated |
| C7orf25 | 5.05E-03 | 1.53E-03 | -3.329062 | -1.70369075 | -1.649238 | 1.649238 | Down-regulated |
| BCL6 | 1.74E-04 | 1.46E-05 | -4.742871 | 2.71426798 | -1.649188 | 1.649188 | Down-regulated |
| RNF167 | 2.48E-03 | 4.97E-04 | -3.694534 | -0.64741282 | -1.649101 | 1.649101 | Down-regulated |
| SMG5 | 5.06E-05 | 3.01E-06 | -5.182827 | 4.23568659 | -1.648419 | 1.648419 | Down-regulated |
| ADM | 8.70E-10 | 4.87E-12 | -8.70366 | 17.22521635 | -1.648296 | 1.648296 | Down-regulated |
| TICAM2 | 9.42E-08 | 1.49E-09 | -7.202357 | 11.63587495 | -1.647604 | 1.647604 | Down-regulated |
| CTBS | 1.30E-07 | 2.20E-09 | -7.100915 | 11.25663406 | -1.647293 | 1.647293 | Down-regulated |
| HBP1 | 2.08E-07 | 3.86E-09 | -6.954114 | 10.70819876 | -1.647152 | 1.647152 | Down-regulated |
| PMPCA | 5.77E-04 | 6.77E-05 | -4.300534 | 1.24381147 | -1.646935 | 1.646935 | Down-regulated |
| PTTG1 | 5.97E-04 | 7.12E-05 | -4.286089 | 1.19695576 | -1.646066 | 1.646066 | Down-regulated |
| ZNF845 | 4.39E-09 | 3.52E-11 | -8.181997 | 15.29362369 | -1.645442 | 1.645442 | Down-regulated |
| DTX2 | 2.98E-04 | 2.88E-05 | -4.548749 | 2.06080427 | -1.645176 | 1.645176 | Down-regulated |
| CRELD2 | 6.89E-05 | 4.46E-06 | -5.074871 | 3.85766169 | -1.645016 | 1.645016 | Down-regulated |
| LRP5L | 6.63E-06 | 2.49E-07 | -5.856864 | 6.65075535 | -1.644959 | 1.644959 | Down-regulated |
| CSNK1G2 | 3.01E-05 | 1.61E-06 | -5.354611 | 4.84277112 | -1.644676 | 1.644676 | Down-regulated |
| WTAP | 5.83E-10 | 2.91E-12 | -8.839993 | 17.72693038 | -1.644429 | 1.644429 | Down-regulated |
| QSOX1 | 8.32E-07 | 2.07E-08 | -6.514104 | 9.06956331 | -1.64323 | 1.64323 | Down-regulated |
| DCXR | 6.77E-07 | 1.59E-08 | -6.583409 | 9.32694654 | -1.642081 | 1.642081 | Down-regulated |
| COQ10B | 5.33E-06 | 1.91E-07 | -5.927093 | 6.9068223 | -1.642014 | 1.642014 | Down-regulated |
| FAM120AOS | 7.11E-05 | 4.62E-06 | -5.064899 | 3.82288811 | -1.6418 | 1.6418 | Down-regulated |
| LRG1 | 1.57E-08 | 1.71E-10 | -7.768542 | 13.75264173 | -1.641741 | 1.641741 | Down-regulated |
| IL10RB | 5.15E-05 | 3.08E-06 | -5.176558 | 4.21365563 | -1.641223 | 1.641223 | Down-regulated |
| WDR83 | 1.02E-03 | 1.43E-04 | -4.078125 | 0.53145625 | -1.640547 | 1.640547 | Down-regulated |
| GLUD1 | 7.79E-08 | 1.20E-09 | -7.259638 | 11.85008055 | -1.640518 | 1.640518 | Down-regulated |
| NUDT2 | 9.00E-07 | 2.29E-08 | -6.487701 | 8.97159684 | -1.640393 | 1.640393 | Down-regulated |
| STX2 | 1.38E-04 | 1.08E-05 | -4.829037 | 3.00805076 | -1.639667 | 1.639667 | Down-regulated |
| SMS | 6.89E-10 | 3.63E-12 | -8.781877 | 17.51323449 | -1.639469 | 1.639469 | Down-regulated |
| PGK1 | 1.29E-07 | 2.19E-09 | -7.102772 | 11.26357432 | -1.639108 | 1.639108 | Down-regulated |
| CAPS | 7.24E-04 | 9.16E-05 | -4.21133 | 0.95574502 | -1.638324 | 1.638324 | Down-regulated |
| STX6 | 9.70E-09 | 9.63E-11 | -7.918293 | 14.3115344 | -1.638165 | 1.638165 | Down-regulated |
| FAM131A | 1.53E-07 | 2.67E-09 | -7.050332 | 11.06760085 | -1.637693 | 1.637693 | Down-regulated |
| CUL4B | 6.87E-05 | 4.45E-06 | -5.075588 | 3.86016418 | -1.637416 | 1.637416 | Down-regulated |
| ADSS | 6.51E-04 | 7.95E-05 | -4.253218 | 1.09063161 | -1.63706 | 1.63706 | Down-regulated |
| EIF6 | 3.43E-06 | 1.13E-07 | -6.066938 | 7.41867721 | -1.636375 | 1.636375 | Down-regulated |
| TMEM170B | 3.12E-06 | 1.00E-07 | -6.098732 | 7.53538583 | -1.636034 | 1.636034 | Down-regulated |
| DEF6 | 7.74E-04 | 9.97E-05 | -4.186149 | 0.87498763 | -1.635262 | 1.635262 | Down-regulated |
| UBE2J2 | 2.96E-05 | 1.58E-06 | -5.360174 | 4.86253887 | -1.635017 | 1.635017 | Down-regulated |
| KIF5B | 1.13E-07 | 1.87E-09 | -7.143328 | 11.41517686 | -1.634737 | 1.634737 | Down-regulated |
| CSRNP1 | 9.73E-05 | 6.90E-06 | -4.953657 | 3.43670936 | -1.634426 | 1.634426 | Down-regulated |
| ALKBH5 | 1.15E-09 | 7.04E-12 | -8.606294 | 16.86604595 | -1.634208 | 1.634208 | Down-regulated |
| NDUFS8 | 5.98E-05 | 3.72E-06 | -5.124938 | 4.0326276 | -1.633435 | 1.633435 | Down-regulated |
| SLC43A2 | 1.44E-04 | 1.14E-05 | -4.813186 | 2.95384206 | -1.633032 | 1.633032 | Down-regulated |
| CD2BP2 | 6.25E-04 | 7.55E-05 | -4.268765 | 1.14086746 | -1.632439 | 1.632439 | Down-regulated |
| EML3 | 4.41E-03 | 1.23E-03 | -3.403157 | -1.49528697 | -1.632328 | 1.632328 | Down-regulated |
| NAA38 | 5.65E-04 | 6.59E-05 | -4.308893 | 1.27096087 | -1.632251 | 1.632251 | Down-regulated |
| FAM189B | 3.07E-04 | 3.00E-05 | -4.537277 | 2.02256975 | -1.631657 | 1.631657 | Down-regulated |
| LRR1 | 7.64E-09 | 7.20E-11 | -7.994575 | 14.59592748 | -1.631267 | 1.631267 | Down-regulated |
| ING4 | 9.55E-09 | 9.42E-11 | -7.924139 | 14.33333683 | -1.630932 | 1.630932 | Down-regulated |
| MUL1 | 3.09E-05 | 1.66E-06 | -5.345834 | 4.81159828 | -1.630812 | 1.630812 | Down-regulated |
| SERTAD1 | 1.25E-03 | 1.90E-04 | -3.992845 | 0.26368482 | -1.630353 | 1.630353 | Down-regulated |
| MFSD13A | 2.46E-03 | 4.93E-04 | -3.697227 | -0.63937549 | -1.630247 | 1.630247 | Down-regulated |
| FAM58A | 3.06E-05 | 1.64E-06 | -5.348815 | 4.82218493 | -1.629905 | 1.629905 | Down-regulated |
| VPS26B | 3.61E-07 | 7.47E-09 | -6.781742 | 10.06512492 | -1.629326 | 1.629326 | Down-regulated |
| SUZ12 | 6.04E-08 | 8.89E-10 | -7.337457 | 12.14112199 | -1.628529 | 1.628529 | Down-regulated |
| TNFSF14 | 1.69E-09 | 1.10E-11 | -8.48863 | 16.43111549 | -1.628304 | 1.628304 | Down-regulated |
| RNF115 | 3.41E-08 | 4.49E-10 | -7.515881 | 12.80840663 | -1.628196 | 1.628196 | Down-regulated |
| XPR1 | 3.79E-13 | 3.03E-16 | -11.35894 | 26.65377806 | -1.627518 | 1.627518 | Down-regulated |
| DAPP1 | 1.55E-04 | 1.25E-05 | -4.786759 | 2.86363097 | -1.626089 | 1.626089 | Down-regulated |
| ANKRD39 | 1.55E-04 | 1.24E-05 | -4.78839 | 2.86919167 | -1.626071 | 1.626071 | Down-regulated |
| MED22 | 2.97E-09 | 2.25E-11 | -8.300247 | 15.73292721 | -1.625485 | 1.625485 | Down-regulated |
| CENPB | 2.09E-04 | 1.83E-05 | -4.678193 | 2.49522053 | -1.625125 | 1.625125 | Down-regulated |
| GPS1 | 6.32E-04 | 7.65E-05 | -4.264783 | 1.12799369 | -1.624771 | 1.624771 | Down-regulated |
| PRDX4 | 5.21E-05 | 3.14E-06 | -5.171384 | 4.19548321 | -1.623527 | 1.623527 | Down-regulated |
| ZBTB3 | 2.15E-06 | 6.50E-08 | -6.213618 | 7.9580517 | -1.623457 | 1.623457 | Down-regulated |
| PHF11 | 7.24E-06 | 2.78E-07 | -5.82814 | 6.54622345 | -1.623306 | 1.623306 | Down-regulated |
| PLEKHO2 | 9.00E-06 | 3.64E-07 | -5.755462 | 6.28228698 | -1.622486 | 1.622486 | Down-regulated |
| MAZ | 2.30E-04 | 2.08E-05 | -4.642581 | 2.37516883 | -1.622255 | 1.622255 | Down-regulated |
| CEBPD | 1.26E-13 | 5.30E-17 | -11.862044 | 28.34305076 | -1.621745 | 1.621745 | Down-regulated |
| ARFGAP2 | 1.65E-07 | 2.92E-09 | -7.02717 | 10.98105995 | -1.621308 | 1.621308 | Down-regulated |
| ETV6 | 7.76E-11 | 2.55E-13 | -9.491582 | 20.10275455 | -1.620786 | 1.620786 | Down-regulated |
| UQCR10 | 1.09E-04 | 7.96E-06 | -4.913586 | 3.2984033 | -1.620642 | 1.620642 | Down-regulated |
| UPF2 | 2.66E-03 | 5.53E-04 | -3.660949 | -0.74735136 | -1.620583 | 1.620583 | Down-regulated |
| SLC30A1 | 1.59E-11 | 3.57E-14 | -10.02556 | 22.01817836 | -1.620523 | 1.620523 | Down-regulated |
| ZNF746 | 2.37E-04 | 2.16E-05 | -4.631795 | 2.33888704 | -1.620397 | 1.620397 | Down-regulated |
| SRRT | 2.46E-08 | 3.00E-10 | -7.621549 | 13.20345106 | -1.619765 | 1.619765 | Down-regulated |
| WDR13 | 5.30E-06 | 1.90E-07 | -5.928989 | 6.91374549 | -1.619084 | 1.619084 | Down-regulated |
| NDUFAF7 | 5.99E-10 | 3.02E-12 | -8.830522 | 17.6921223 | -1.618911 | 1.618911 | Down-regulated |
| IRF7 | 1.28E-05 | 5.57E-07 | -5.641583 | 5.87040676 | -1.617099 | 1.617099 | Down-regulated |
| WDR1 | 2.60E-07 | 5.07E-09 | -6.882994 | 10.44273189 | -1.616618 | 1.616618 | Down-regulated |
| DDOST | 2.45E-07 | 4.69E-09 | -6.903177 | 10.51805142 | -1.615451 | 1.615451 | Down-regulated |
| TMEM33 | 2.22E-09 | 1.61E-11 | -8.387909 | 16.0580915 | -1.615212 | 1.615212 | Down-regulated |
| WDR41 | 4.87E-08 | 6.77E-10 | -7.408493 | 12.40680481 | -1.615167 | 1.615167 | Down-regulated |
| BANP | 9.75E-07 | 2.53E-08 | -6.461972 | 8.87618182 | -1.61512 | 1.61512 | Down-regulated |
| C1orf112 | 8.99E-09 | 8.75E-11 | -7.943374 | 14.40506233 | -1.614989 | 1.614989 | Down-regulated |
| DPM1 | 7.96E-07 | 1.96E-08 | -6.5284 | 9.12262843 | -1.614523 | 1.614523 | Down-regulated |
| RMI1 | 5.88E-08 | 8.54E-10 | -7.348042 | 12.18071202 | -1.614348 | 1.614348 | Down-regulated |
| ZNF576 | 1.38E-04 | 1.07E-05 | -4.829973 | 3.0112541 | -1.614122 | 1.614122 | Down-regulated |
| ELMOD2 | 1.14E-08 | 1.16E-10 | -7.868476 | 14.12568846 | -1.614078 | 1.614078 | Down-regulated |
| TCN1 | 1.79E-06 | 5.20E-08 | -6.272337 | 8.1746184 | -1.613821 | 1.613821 | Down-regulated |
| RBX1 | 6.03E-08 | 8.84E-10 | -7.339094 | 12.14724402 | -1.613353 | 1.613353 | Down-regulated |
| DDX5 | 6.83E-07 | 1.61E-08 | -6.580024 | 9.31436778 | -1.612975 | 1.612975 | Down-regulated |
| SELPLG | 2.51E-08 | 3.06E-10 | -7.615911 | 13.18237668 | -1.611817 | 1.611817 | Down-regulated |
| CDK4 | 6.12E-05 | 3.84E-06 | -5.115933 | 4.0011127 | -1.611747 | 1.611747 | Down-regulated |
| ARHGAP30 | 3.99E-04 | 4.20E-05 | -4.44013 | 1.70059438 | -1.611611 | 1.611611 | Down-regulated |
| DTX3L | 1.64E-07 | 2.89E-09 | -7.029728 | 10.9906177 | -1.611527 | 1.611527 | Down-regulated |
| HENMT1 | 9.24E-07 | 2.36E-08 | -6.480297 | 8.94413495 | -1.611425 | 1.611425 | Down-regulated |
| IL18RAP | 8.99E-07 | 2.29E-08 | -6.48857 | 8.97482159 | -1.610737 | 1.610737 | Down-regulated |
| AIRE | 1.26E-04 | 9.63E-06 | -4.860357 | 3.11537292 | -1.610283 | 1.610283 | Down-regulated |
| FAM173B | 4.53E-03 | 1.29E-03 | -3.387543 | -1.53945717 | -1.610195 | 1.610195 | Down-regulated |
| LINC00936 | 2.97E-08 | 3.79E-10 | -7.560224 | 12.97420467 | -1.60992 | 1.60992 | Down-regulated |
| DBNDD2 | 1.36E-05 | 6.01E-07 | -5.620979 | 5.7961187 | -1.60901 | 1.60901 | Down-regulated |
| CD48 | 4.93E-05 | 2.92E-06 | -5.191462 | 4.26604112 | -1.60849 | 1.60849 | Down-regulated |
| KDM7A | 1.08E-05 | 4.50E-07 | -5.69856 | 6.07621809 | -1.608061 | 1.608061 | Down-regulated |
| PMVK | 1.83E-06 | 5.35E-08 | -6.264929 | 8.14727708 | -1.607288 | 1.607288 | Down-regulated |
| RNF149 | 9.47E-09 | 9.31E-11 | -7.927018 | 14.34407141 | -1.606778 | 1.606778 | Down-regulated |
| VPS8 | 6.53E-06 | 2.45E-07 | -5.861836 | 6.66886006 | -1.606703 | 1.606703 | Down-regulated |
| TMEM189 | 5.11E-04 | 5.77E-05 | -4.347796 | 1.39766055 | -1.606274 | 1.606274 | Down-regulated |
| MED29 | 6.11E-05 | 3.83E-06 | -5.116797 | 4.00413503 | -1.606174 | 1.606174 | Down-regulated |
| PATL1 | 1.53E-04 | 1.23E-05 | -4.792418 | 2.8829302 | -1.605443 | 1.605443 | Down-regulated |
| WRB | 1.51E-08 | 1.63E-10 | -7.781318 | 13.80035161 | -1.605389 | 1.605389 | Down-regulated |
| ABCF1 | 1.50E-03 | 2.42E-04 | -3.918082 | 0.03152717 | -1.605131 | 1.605131 | Down-regulated |
| FAM213A | 6.73E-08 | 1.01E-09 | -7.303402 | 12.01375418 | -1.604541 | 1.604541 | Down-regulated |
| SRP14 | 1.35E-05 | 5.92E-07 | -5.625227 | 5.81142901 | -1.604268 | 1.604268 | Down-regulated |
| CPEB2-AS1 | 2.40E-04 | 2.19E-05 | -4.627804 | 2.32546914 | -1.60346 | 1.60346 | Down-regulated |
| NAGK | 1.45E-10 | 5.39E-13 | -9.290402 | 19.37340647 | -1.602964 | 1.602964 | Down-regulated |
| CPSF2 | 8.23E-07 | 2.05E-08 | -6.517396 | 9.0817818 | -1.602644 | 1.602644 | Down-regulated |
| SF3B6 | 2.15E-10 | 8.44E-13 | -9.170152 | 18.93560145 | -1.601092 | 1.601092 | Down-regulated |
| SAFB | 9.90E-10 | 5.76E-12 | -8.659314 | 17.06171611 | -1.600892 | 1.600892 | Down-regulated |
| ATG3 | 7.54E-06 | 2.92E-07 | -5.8148 | 6.49771806 | -1.599774 | 1.599774 | Down-regulated |
| CALM2 | 4.55E-05 | 2.64E-06 | -5.218807 | 4.3622918 | -1.59975 | 1.59975 | Down-regulated |
| MED28 | 3.79E-06 | 1.28E-07 | -6.03403 | 7.2980091 | -1.599742 | 1.599742 | Down-regulated |
| ATP5J | 1.03E-07 | 1.68E-09 | -7.171118 | 11.5190736 | -1.599703 | 1.599703 | Down-regulated |
| CIB1 | 7.19E-08 | 1.10E-09 | -7.282805 | 11.93671869 | -1.599315 | 1.599315 | Down-regulated |
| ARL5A | 2.64E-04 | 2.46E-05 | -4.594632 | 2.21416589 | -1.598934 | 1.598934 | Down-regulated |
| BRI3BP | 7.16E-06 | 2.73E-07 | -5.832759 | 6.56302235 | -1.59851 | 1.59851 | Down-regulated |
| RNPEP | 1.77E-07 | 3.20E-09 | -7.003447 | 10.89244208 | -1.598359 | 1.598359 | Down-regulated |
| MAT2A | 3.14E-06 | 1.01E-07 | -6.095725 | 7.52434142 | -1.598342 | 1.598342 | Down-regulated |
| EDEM2 | 1.46E-05 | 6.56E-07 | -5.597691 | 5.71224332 | -1.598024 | 1.598024 | Down-regulated |
| ATPIF1 | 5.20E-07 | 1.15E-08 | -6.66804 | 9.64166435 | -1.597982 | 1.597982 | Down-regulated |
| IDH3A | 9.15E-05 | 6.35E-06 | -4.976486 | 3.5156934 | -1.597691 | 1.597691 | Down-regulated |
| DBN1 | 1.01E-08 | 1.01E-10 | -7.905061 | 14.26217944 | -1.597447 | 1.597447 | Down-regulated |
| CASP4 | 3.32E-04 | 3.33E-05 | -4.507706 | 1.92421909 | -1.597082 | 1.597082 | Down-regulated |
| TMEM115 | 2.05E-07 | 3.79E-09 | -6.959131 | 10.726933 | -1.59704 | 1.59704 | Down-regulated |
| MRPL17 | 1.68E-12 | 1.90E-15 | -10.838977 | 24.87267047 | -1.596549 | 1.596549 | Down-regulated |
| MGAT5 | 9.56E-06 | 3.91E-07 | -5.736492 | 6.21352977 | -1.595817 | 1.595817 | Down-regulated |
| SSH3 | 6.84E-04 | 8.51E-05 | -4.233318 | 1.02646477 | -1.595724 | 1.595724 | Down-regulated |
| FKBP11 | 4.05E-05 | 2.30E-06 | -5.257357 | 4.49826778 | -1.595016 | 1.595016 | Down-regulated |
| FBN2 | 4.23E-05 | 2.42E-06 | -5.242686 | 4.44648071 | -1.594306 | 1.594306 | Down-regulated |
| HSPA6 | 2.46E-03 | 4.92E-04 | -3.697664 | -0.63807142 | -1.592533 | 1.592533 | Down-regulated |
| OPA3 | 8.11E-08 | 1.26E-09 | -7.246319 | 11.80026953 | -1.592232 | 1.592232 | Down-regulated |
| PGS1 | 3.86E-07 | 8.07E-09 | -6.761607 | 9.99008824 | -1.591672 | 1.591672 | Down-regulated |
| SLC25A44 | 8.09E-12 | 1.31E-14 | -10.301095 | 22.99404347 | -1.591566 | 1.591566 | Down-regulated |
| PSD4 | 1.63E-04 | 1.34E-05 | -4.767756 | 2.79888865 | -1.59134 | 1.59134 | Down-regulated |
| RPS16 | 6.06E-09 | 5.38E-11 | -8.070901 | 14.88025088 | -1.591278 | 1.591278 | Down-regulated |
| B9D2 | 1.88E-08 | 2.16E-10 | -7.707078 | 13.5230616 | -1.59036 | 1.59036 | Down-regulated |
| TACO1 | 5.14E-09 | 4.42E-11 | -8.122193 | 15.07117125 | -1.589997 | 1.589997 | Down-regulated |
| SPG7 | 4.66E-05 | 2.72E-06 | -5.210605 | 4.33340124 | -1.589491 | 1.589491 | Down-regulated |
| TMEM2 | 3.57E-06 | 1.19E-07 | -6.054002 | 7.3712262 | -1.589462 | 1.589462 | Down-regulated |
| ZNF571 | 2.59E-04 | 2.41E-05 | -4.600928 | 2.23526248 | -1.58905 | 1.58905 | Down-regulated |
| CBX3 | 8.34E-04 | 1.10E-04 | -4.156973 | 0.7817371 | -1.588902 | 1.588902 | Down-regulated |
| PEX13 | 1.39E-05 | 6.18E-07 | -5.613517 | 5.7692341 | -1.588764 | 1.588764 | Down-regulated |
| C15orf57 | 7.55E-06 | 2.92E-07 | -5.814173 | 6.49543792 | -1.588655 | 1.588655 | Down-regulated |
| THBS3 | 2.35E-05 | 1.19E-06 | -5.437037 | 5.13630855 | -1.588303 | 1.588303 | Down-regulated |
| RAD21 | 2.98E-06 | 9.50E-08 | -6.11304 | 7.58794479 | -1.588219 | 1.588219 | Down-regulated |
| USP24 | 3.62E-06 | 1.21E-07 | -6.04967 | 7.35534183 | -1.587884 | 1.587884 | Down-regulated |
| COQ3 | 6.78E-05 | 4.37E-06 | -5.080628 | 3.87774922 | -1.587375 | 1.587375 | Down-regulated |
| SWI5 | 1.98E-05 | 9.58E-07 | -5.495301 | 5.34461898 | -1.586732 | 1.586732 | Down-regulated |
| PSME3 | 1.15E-09 | 7.09E-12 | -8.604415 | 16.85910896 | -1.586208 | 1.586208 | Down-regulated |
| MED19 | 5.09E-05 | 3.04E-06 | -5.180128 | 4.22619944 | -1.586042 | 1.586042 | Down-regulated |
| RPS27L | 4.50E-10 | 2.10E-12 | -8.927366 | 18.04769454 | -1.585986 | 1.585986 | Down-regulated |
| SLC44A4 | 4.27E-04 | 4.59E-05 | -4.414405 | 1.61588558 | -1.585952 | 1.585952 | Down-regulated |
| ATP5G1 | 5.30E-08 | 7.56E-10 | -7.379838 | 12.29963218 | -1.585753 | 1.585753 | Down-regulated |
| SLC25A46 | 5.32E-05 | 3.23E-06 | -5.164114 | 4.16995905 | -1.585488 | 1.585488 | Down-regulated |
| FASTK | 1.68E-03 | 2.83E-04 | -3.869882 | -0.1168249 | -1.585233 | 1.585233 | Down-regulated |
| ARHGEF6 | 3.20E-06 | 1.04E-07 | -6.089981 | 7.5032507 | -1.584805 | 1.584805 | Down-regulated |
| HCFC1R1 | 4.92E-07 | 1.08E-08 | -6.684904 | 9.70442892 | -1.584548 | 1.584548 | Down-regulated |
| DNAJC17 | 4.24E-04 | 4.54E-05 | -4.417427 | 1.62582512 | -1.583446 | 1.583446 | Down-regulated |
| C17orf49 | 1.67E-04 | 1.38E-05 | -4.758741 | 2.76821065 | -1.58344 | 1.58344 | Down-regulated |
| PGM1 | 7.22E-04 | 9.13E-05 | -4.212322 | 0.95893105 | -1.582915 | 1.582915 | Down-regulated |
| C9orf64 | 6.34E-03 | 2.27E-03 | -3.19718 | -2.06696325 | -1.582383 | 1.582383 | Down-regulated |
| LSM3 | 1.54E-09 | 9.82E-12 | -8.518274 | 16.5407786 | -1.581589 | 1.581589 | Down-regulated |
| DOPEY2 | 3.15E-03 | 7.16E-04 | -3.578182 | -0.99123947 | -1.580831 | 1.580831 | Down-regulated |
| C6orf47 | 7.14E-10 | 3.79E-12 | -8.770179 | 17.47018808 | -1.580804 | 1.580804 | Down-regulated |
| NME8 | 4.59E-06 | 1.60E-07 | -5.974847 | 7.08132872 | -1.580483 | 1.580483 | Down-regulated |
| TIPARP | 8.95E-05 | 6.17E-06 | -4.984562 | 3.5436701 | -1.579148 | 1.579148 | Down-regulated |
| TXN | 3.65E-06 | 1.22E-07 | -6.04695 | 7.34536827 | -1.578784 | 1.578784 | Down-regulated |
| ENKD1 | 1.34E-04 | 1.03E-05 | -4.840793 | 3.04830251 | -1.578327 | 1.578327 | Down-regulated |
| ZNF787 | 1.89E-05 | 9.05E-07 | -5.510715 | 5.39983917 | -1.578206 | 1.578206 | Down-regulated |
| TM9SF2 | 4.38E-05 | 2.52E-06 | -5.231641 | 4.40752355 | -1.578115 | 1.578115 | Down-regulated |
| ARL15 | 7.15E-09 | 6.67E-11 | -8.014354 | 14.66962944 | -1.578061 | 1.578061 | Down-regulated |
| SLC38A10 | 3.88E-07 | 8.14E-09 | -6.759241 | 9.98127239 | -1.578002 | 1.578002 | Down-regulated |
| FMC1 | 9.10E-07 | 2.32E-08 | -6.484788 | 8.96079276 | -1.57786 | 1.57786 | Down-regulated |
| MRPS23 | 1.89E-08 | 2.18E-10 | -7.704302 | 13.5126936 | -1.577407 | 1.577407 | Down-regulated |
| UBXN2A | 2.72E-11 | 6.63E-14 | -9.856576 | 21.41537199 | -1.576757 | 1.576757 | Down-regulated |
| STAG1 | 1.05E-07 | 1.72E-09 | -7.165915 | 11.49961883 | -1.57652 | 1.57652 | Down-regulated |
| RNGTT | 1.36E-07 | 2.32E-09 | -7.087634 | 11.20699555 | -1.576171 | 1.576171 | Down-regulated |
| PPP2CA | 2.15E-04 | 1.90E-05 | -4.668095 | 2.46113734 | -1.575855 | 1.575855 | Down-regulated |
| CCDC90B | 3.79E-04 | 3.95E-05 | -4.458356 | 1.76075235 | -1.575749 | 1.575749 | Down-regulated |
| KCND1 | 8.01E-08 | 1.24E-09 | -7.2503 | 11.81515627 | -1.575419 | 1.575419 | Down-regulated |
| TIGAR | 3.56E-04 | 3.64E-05 | -4.481753 | 1.83814856 | -1.575212 | 1.575212 | Down-regulated |
| MSL3 | 2.79E-06 | 8.76E-08 | -6.134722 | 7.66763821 | -1.575127 | 1.575127 | Down-regulated |
| STK19 | 2.47E-04 | 2.27E-05 | -4.617453 | 2.29069965 | -1.575095 | 1.575095 | Down-regulated |
| NENF | 3.63E-04 | 3.74E-05 | -4.473637 | 1.81128058 | -1.574715 | 1.574715 | Down-regulated |
| UBL4A | 1.93E-09 | 1.33E-11 | -8.437824 | 16.24303126 | -1.574485 | 1.574485 | Down-regulated |
| DERA | 4.04E-05 | 2.29E-06 | -5.258432 | 4.50206433 | -1.574233 | 1.574233 | Down-regulated |
| PPM1D | 1.01E-07 | 1.64E-09 | -7.178496 | 11.54665933 | -1.57386 | 1.57386 | Down-regulated |
| E2F4 | 2.56E-10 | 1.02E-12 | -9.118798 | 18.74822522 | -1.573653 | 1.573653 | Down-regulated |
| B2M | 9.90E-10 | 5.78E-12 | -8.658167 | 17.05748512 | -1.573172 | 1.573172 | Down-regulated |
| PSTPIP2 | 1.86E-03 | 3.27E-04 | -3.825125 | -0.25362939 | -1.57303 | 1.57303 | Down-regulated |
| P4HA1 | 7.93E-06 | 3.10E-07 | -5.798223 | 6.43747942 | -1.572322 | 1.572322 | Down-regulated |
| UBIAD1 | 1.75E-08 | 1.96E-10 | -7.73187 | 13.6156783 | -1.572288 | 1.572288 | Down-regulated |
| AMY1B | 5.04E-03 | 1.53E-03 | -3.329483 | -1.70251453 | -1.572176 | 1.572176 | Down-regulated |
| AKR1D1 | 1.19E-05 | 5.10E-07 | -5.665168 | 5.95553626 | -1.572146 | 1.572146 | Down-regulated |
| RAB27A | 8.37E-06 | 3.32E-07 | -5.779924 | 6.37103105 | -1.571323 | 1.571323 | Down-regulated |
| GNA15 | 2.99E-05 | 1.60E-06 | -5.356109 | 4.84809529 | -1.571234 | 1.571234 | Down-regulated |
| LOC100129550 | 6.00E-05 | 3.74E-06 | -5.123265 | 4.02676932 | -1.57094 | 1.57094 | Down-regulated |
| ZNF524 | 1.31E-06 | 3.62E-08 | -6.367667 | 8.52690564 | -1.570025 | 1.570025 | Down-regulated |
| STAU2 | 5.81E-10 | 2.84E-12 | -8.846916 | 17.75236818 | -1.5699 | 1.5699 | Down-regulated |
| DRAM1 | 1.75E-05 | 8.23E-07 | -5.536285 | 5.49153768 | -1.569112 | 1.569112 | Down-regulated |
| SLC24A4 | 1.87E-03 | 3.30E-04 | -3.822798 | -0.26071798 | -1.569094 | 1.569094 | Down-regulated |
| TRMO | 1.53E-06 | 4.34E-08 | -6.319945 | 8.35044785 | -1.569056 | 1.569056 | Down-regulated |
| CTSL | 4.88E-09 | 4.08E-11 | -8.143117 | 15.14902433 | -1.568978 | 1.568978 | Down-regulated |
| TTC32 | 3.49E-04 | 3.55E-05 | -4.489092 | 1.86246287 | -1.568631 | 1.568631 | Down-regulated |
| TNFSF13B | 9.53E-05 | 6.71E-06 | -4.961447 | 3.46364533 | -1.568481 | 1.568481 | Down-regulated |
| SLIRP | 4.32E-05 | 2.49E-06 | -5.235584 | 4.42142781 | -1.568404 | 1.568404 | Down-regulated |
| FKTN | 2.07E-04 | 1.82E-05 | -4.681198 | 2.50536905 | -1.568269 | 1.568269 | Down-regulated |
| DEGS1 | 1.98E-06 | 5.88E-08 | -6.239698 | 8.05419847 | -1.567794 | 1.567794 | Down-regulated |
| FASTKD5 | 2.71E-09 | 2.02E-11 | -8.328008 | 15.83594799 | -1.567754 | 1.567754 | Down-regulated |
| CMIP | 4.94E-09 | 4.17E-11 | -8.137627 | 15.12859891 | -1.564924 | 1.564924 | Down-regulated |
| NUDC | 8.14E-04 | 1.07E-04 | -4.166294 | 0.81149099 | -1.564544 | 1.564544 | Down-regulated |
| MTRF1L | 1.04E-04 | 7.45E-06 | -4.931946 | 3.36171763 | -1.564268 | 1.564268 | Down-regulated |
| IFFO1 | 1.02E-07 | 1.66E-09 | -7.174623 | 11.53217636 | -1.563829 | 1.563829 | Down-regulated |
| CDS2 | 1.76E-05 | 8.25E-07 | -5.535596 | 5.48906408 | -1.56381 | 1.56381 | Down-regulated |
| RARS2 | 6.53E-06 | 2.44E-07 | -5.862281 | 6.67047885 | -1.563518 | 1.563518 | Down-regulated |
| ALG14 | 3.07E-05 | 1.65E-06 | -5.347223 | 4.81653097 | -1.563244 | 1.563244 | Down-regulated |
| ZNF613 | 1.07E-04 | 7.75E-06 | -4.921026 | 3.32405174 | -1.562751 | 1.562751 | Down-regulated |
| GABARAP | 4.07E-07 | 8.62E-09 | -6.744108 | 9.92489043 | -1.562718 | 1.562718 | Down-regulated |
| EFCAB11 | 9.70E-09 | 9.61E-11 | -7.91885 | 14.31361101 | -1.562559 | 1.562559 | Down-regulated |
| CD151 | 1.63E-05 | 7.49E-07 | -5.561745 | 5.58296228 | -1.56197 | 1.56197 | Down-regulated |
| CEP85L | 2.56E-10 | 1.02E-12 | -9.119074 | 18.74923497 | -1.56193 | 1.56193 | Down-regulated |
| MCMBP | 1.70E-06 | 4.93E-08 | -6.286562 | 8.22713301 | -1.561809 | 1.561809 | Down-regulated |
| PNKP | 2.90E-08 | 3.65E-10 | -7.569803 | 13.01001545 | -1.561282 | 1.561282 | Down-regulated |
| HIST1H3F | 9.64E-09 | 9.53E-11 | -7.92111 | 14.32204055 | -1.561195 | 1.561195 | Down-regulated |
| UQCRH | 6.91E-07 | 1.63E-08 | -6.576952 | 9.30295034 | -1.560831 | 1.560831 | Down-regulated |
| C8orf59 | 1.00E-10 | 3.39E-13 | -9.415203 | 19.8263223 | -1.559923 | 1.559923 | Down-regulated |
| TRIB3 | 8.25E-06 | 3.26E-07 | -5.784865 | 6.38896952 | -1.559693 | 1.559693 | Down-regulated |
| NOMO2 | 1.72E-09 | 1.13E-11 | -8.480557 | 16.40123986 | -1.559602 | 1.559602 | Down-regulated |
| MRPL42 | 1.19E-05 | 5.10E-07 | -5.665502 | 5.95674163 | -1.558623 | 1.558623 | Down-regulated |
| UBN2 | 1.29E-11 | 2.52E-14 | -10.121354 | 22.3584547 | -1.558118 | 1.558118 | Down-regulated |
| ANKRD17 | 6.22E-07 | 1.43E-08 | -6.611222 | 9.43032464 | -1.557738 | 1.557738 | Down-regulated |
| GBA | 2.22E-04 | 1.99E-05 | -4.655131 | 2.4174279 | -1.55765 | 1.55765 | Down-regulated |
| TRAM1 | 1.87E-05 | 8.92E-07 | -5.514494 | 5.41338269 | -1.556989 | 1.556989 | Down-regulated |
| RAC1 | 3.93E-11 | 1.09E-13 | -9.721019 | 20.92952829 | -1.55691 | 1.55691 | Down-regulated |
| LSM12 | 1.96E-04 | 1.70E-05 | -4.700411 | 2.57032147 | -1.556516 | 1.556516 | Down-regulated |
| C1GALT1C1 | 1.08E-07 | 1.77E-09 | -7.15748 | 11.46808376 | -1.556462 | 1.556462 | Down-regulated |
| MAP3K6 | 4.18E-04 | 4.47E-05 | -4.42238 | 1.6421203 | -1.556288 | 1.556288 | Down-regulated |
| TDRD9 | 1.66E-07 | 2.95E-09 | -7.02436 | 10.97056502 | -1.555983 | 1.555983 | Down-regulated |
| TRIM69 | 2.66E-07 | 5.27E-09 | -6.872878 | 10.40498464 | -1.555885 | 1.555885 | Down-regulated |
| CXXC1 | 1.71E-05 | 7.98E-07 | -5.54474 | 5.52188394 | -1.555713 | 1.555713 | Down-regulated |
| RNASEL | 3.37E-04 | 3.39E-05 | -4.502202 | 1.90594522 | -1.555083 | 1.555083 | Down-regulated |
| RNASET2 | 3.88E-07 | 8.17E-09 | -6.758144 | 9.97718763 | -1.554898 | 1.554898 | Down-regulated |
| DNAH1 | 3.46E-03 | 8.28E-04 | -3.531595 | -1.12697752 | -1.554886 | 1.554886 | Down-regulated |
| RAB8B | 2.68E-08 | 3.31E-10 | -7.595385 | 13.10565274 | -1.554348 | 1.554348 | Down-regulated |
| RBM12B | 1.05E-06 | 2.76E-08 | -6.438636 | 8.78968718 | -1.55422 | 1.55422 | Down-regulated |
| RPS15A | 3.87E-06 | 1.31E-07 | -6.028385 | 7.27732014 | -1.554186 | 1.554186 | Down-regulated |
| RWDD1 | 7.17E-04 | 9.06E-05 | -4.214623 | 0.96632243 | -1.553941 | 1.553941 | Down-regulated |
| PAM16 | 2.97E-04 | 2.87E-05 | -4.550508 | 2.06667207 | -1.552707 | 1.552707 | Down-regulated |
| TBPL1 | 1.41E-08 | 1.50E-10 | -7.803127 | 13.88178037 | -1.552551 | 1.552551 | Down-regulated |
| ZC3HAV1 | 2.28E-04 | 2.06E-05 | -4.645832 | 2.3861115 | -1.552313 | 1.552313 | Down-regulated |
| MRPL44 | 8.71E-05 | 5.95E-06 | -4.994707 | 3.57883899 | -1.551861 | 1.551861 | Down-regulated |
| RNF34 | 3.49E-07 | 7.20E-09 | -6.791253 | 10.10057827 | -1.549697 | 1.549697 | Down-regulated |
| ARHGAP9 | 1.90E-09 | 1.29E-11 | -8.445639 | 16.27197403 | -1.548212 | 1.548212 | Down-regulated |
| CLDN15 | 1.00E-07 | 1.61E-09 | -7.182585 | 11.56194554 | -1.548049 | 1.548049 | Down-regulated |
| IPO11 | 9.64E-06 | 3.96E-07 | -5.733185 | 6.20154918 | -1.54786 | 1.54786 | Down-regulated |
| ATP6V1B2 | 1.33E-06 | 3.70E-08 | -6.361766 | 8.50507656 | -1.547832 | 1.547832 | Down-regulated |
| PSTPIP1 | 3.25E-05 | 1.76E-06 | -5.329997 | 4.75538929 | -1.547715 | 1.547715 | Down-regulated |
| C7orf49 | 3.47E-07 | 7.15E-09 | -6.79328 | 10.10813248 | -1.547626 | 1.547626 | Down-regulated |
| PTPRJ | 5.51E-05 | 3.37E-06 | -5.152315 | 4.12855737 | -1.547273 | 1.547273 | Down-regulated |
| TOP1P2 | 1.10E-04 | 8.08E-06 | -4.90942 | 3.28405169 | -1.546361 | 1.546361 | Down-regulated |
| RPL11 | 5.02E-10 | 2.39E-12 | -8.893098 | 17.92196429 | -1.546032 | 1.546032 | Down-regulated |
| NECTIN2 | 7.51E-06 | 2.89E-07 | -5.816927 | 6.50544811 | -1.545917 | 1.545917 | Down-regulated |
| LRRC8B | 1.85E-05 | 8.81E-07 | -5.518094 | 5.42628714 | -1.545087 | 1.545087 | Down-regulated |
| ATXN2 | 1.90E-04 | 1.63E-05 | -4.712202 | 2.61024058 | -1.544518 | 1.544518 | Down-regulated |
| UBLCP1 | 4.15E-04 | 4.42E-05 | -4.425183 | 1.65134681 | -1.544449 | 1.544449 | Down-regulated |
| APH1B | 8.72E-05 | 5.96E-06 | -4.99428 | 3.57735644 | -1.54431 | 1.54431 | Down-regulated |
| CDYL2 | 2.96E-05 | 1.57E-06 | -5.360699 | 4.86440528 | -1.543505 | 1.543505 | Down-regulated |
| RFC1 | 1.42E-04 | 1.12E-05 | -4.81894 | 2.97351323 | -1.543325 | 1.543325 | Down-regulated |
| COX6C | 2.34E-05 | 1.18E-06 | -5.439271 | 5.14428257 | -1.543054 | 1.543054 | Down-regulated |
| C1orf123 | 1.18E-04 | 8.78E-06 | -4.886243 | 3.20428473 | -1.543 | 1.543 | Down-regulated |
| UBE2E1 | 1.22E-08 | 1.27E-10 | -7.846469 | 14.04356714 | -1.542625 | 1.542625 | Down-regulated |
| PNPT1 | 2.30E-04 | 2.08E-05 | -4.64279 | 2.37587004 | -1.542029 | 1.542029 | Down-regulated |
| PER2 | 2.90E-04 | 2.78E-05 | -4.559774 | 2.09759287 | -1.541931 | 1.541931 | Down-regulated |
| RPF1 | 2.75E-06 | 8.60E-08 | -6.139552 | 7.68539853 | -1.541531 | 1.541531 | Down-regulated |
| SVBP | 4.49E-05 | 2.60E-06 | -5.223263 | 4.37799199 | -1.541241 | 1.541241 | Down-regulated |
| HADHA | 1.48E-08 | 1.59E-10 | -7.786629 | 13.8201818 | -1.541185 | 1.541185 | Down-regulated |
| C2orf49 | 1.66E-04 | 1.37E-05 | -4.760529 | 2.77429255 | -1.540883 | 1.540883 | Down-regulated |
| SLX4 | 2.12E-04 | 1.87E-05 | -4.672704 | 2.47668867 | -1.540471 | 1.540471 | Down-regulated |
| EDEM3 | 6.25E-05 | 3.95E-06 | -5.108253 | 3.97425127 | -1.540315 | 1.540315 | Down-regulated |
| JDP2 | 1.15E-05 | 4.88E-07 | -5.676857 | 5.99775955 | -1.539522 | 1.539522 | Down-regulated |
| RNF113A | 1.08E-05 | 4.52E-07 | -5.697528 | 6.07248486 | -1.539233 | 1.539233 | Down-regulated |
| MRPS16 | 1.68E-08 | 1.85E-10 | -7.747056 | 13.67239895 | -1.539064 | 1.539064 | Down-regulated |
| TIMM22 | 1.18E-07 | 1.98E-09 | -7.12872 | 11.3605665 | -1.538696 | 1.538696 | Down-regulated |
| CRKL | 7.00E-08 | 1.06E-09 | -7.291211 | 11.96815979 | -1.537666 | 1.537666 | Down-regulated |
| CYSTM1 | 4.79E-04 | 5.30E-05 | -4.372436 | 1.47819873 | -1.537316 | 1.537316 | Down-regulated |
| PNPLA6 | 6.46E-05 | 4.11E-06 | -5.097387 | 3.93627265 | -1.537097 | 1.537097 | Down-regulated |
| IFITM3 | 2.60E-07 | 5.06E-09 | -6.883422 | 10.44432915 | -1.536545 | 1.536545 | Down-regulated |
| CLK3 | 1.42E-05 | 6.31E-07 | -5.608157 | 5.74992516 | -1.536445 | 1.536445 | Down-regulated |
| CSNK1D | 2.59E-06 | 8.04E-08 | -6.157299 | 7.75067333 | -1.536003 | 1.536003 | Down-regulated |
| RABL3 | 3.47E-06 | 1.15E-07 | -6.063569 | 7.40631797 | -1.53581 | 1.53581 | Down-regulated |
| SS18L2 | 2.20E-05 | 1.09E-06 | -5.460119 | 5.21875577 | -1.535549 | 1.535549 | Down-regulated |
| NUP37 | 4.82E-05 | 2.84E-06 | -5.198996 | 4.29254234 | -1.535225 | 1.535225 | Down-regulated |
| TATDN3 | 9.08E-04 | 1.23E-04 | -4.123939 | 0.67657069 | -1.535198 | 1.535198 | Down-regulated |
| TXLNA | 3.49E-04 | 3.55E-05 | -4.488714 | 1.86121054 | -1.535042 | 1.535042 | Down-regulated |
| STK24 | 9.99E-06 | 4.14E-07 | -5.721394 | 6.15884752 | -1.534263 | 1.534263 | Down-regulated |
| ZNF839 | 6.63E-05 | 4.23E-06 | -5.089183 | 3.90761453 | -1.534217 | 1.534217 | Down-regulated |
| MGST1 | 4.41E-05 | 2.55E-06 | -5.228382 | 4.39603273 | -1.533736 | 1.533736 | Down-regulated |
| PIWIL4 | 2.64E-04 | 2.46E-05 | -4.594577 | 2.21398343 | -1.53357 | 1.53357 | Down-regulated |
| FAM46A | 8.10E-03 | 3.52E-03 | -3.045467 | -2.47221271 | -1.533078 | 1.533078 | Down-regulated |
| ATG9A | 7.79E-12 | 1.21E-14 | -10.3228 | 23.07053643 | -1.533044 | 1.533044 | Down-regulated |
| C20orf27 | 5.68E-04 | 6.64E-05 | -4.306349 | 1.26269549 | -1.532456 | 1.532456 | Down-regulated |
| DPY30 | 2.33E-05 | 1.17E-06 | -5.442029 | 5.15413276 | -1.531728 | 1.531728 | Down-regulated |
| RIPK3 | 1.04E-04 | 7.46E-06 | -4.931895 | 3.36154406 | -1.531583 | 1.531583 | Down-regulated |
| GABPB1 | 3.88E-07 | 8.15E-09 | -6.758806 | 9.97965255 | -1.531253 | 1.531253 | Down-regulated |
| RCN3 | 9.11E-06 | 3.70E-07 | -5.751641 | 6.26843271 | -1.531072 | 1.531072 | Down-regulated |
| TUBA1C | 1.53E-06 | 4.34E-08 | -6.319813 | 8.34995938 | -1.530352 | 1.530352 | Down-regulated |
| CIAO1 | 9.56E-05 | 6.75E-06 | -4.959616 | 3.45731066 | -1.530017 | 1.530017 | Down-regulated |
| PPA2 | 2.97E-04 | 2.87E-05 | -4.550414 | 2.06635715 | -1.529351 | 1.529351 | Down-regulated |
| PRKAA1 | 1.74E-04 | 1.46E-05 | -4.743129 | 2.71514611 | -1.529235 | 1.529235 | Down-regulated |
| LINC00493 | 1.63E-04 | 1.33E-05 | -4.768893 | 2.80275855 | -1.528485 | 1.528485 | Down-regulated |
| DKFZP564C152 | 1.70E-09 | 1.11E-11 | -8.485908 | 16.42104417 | -1.528296 | 1.528296 | Down-regulated |
| NDUFA1 | 1.01E-05 | 4.17E-07 | -5.719264 | 6.15113649 | -1.527924 | 1.527924 | Down-regulated |
| NDUFB6 | 4.49E-07 | 9.67E-09 | -6.71409 | 9.8130884 | -1.527339 | 1.527339 | Down-regulated |
| APH1A | 3.66E-09 | 2.88E-11 | -8.234961 | 15.4904784 | -1.52715 | 1.52715 | Down-regulated |
| CLEC3B | 6.03E-04 | 7.20E-05 | -4.28254 | 1.18545692 | -1.526476 | 1.526476 | Down-regulated |
| TSPAN31 | 2.67E-10 | 1.09E-12 | -9.102578 | 18.68899373 | -1.526377 | 1.526377 | Down-regulated |
| ISCU | 2.91E-08 | 3.68E-10 | -7.567524 | 13.00149553 | -1.526191 | 1.526191 | Down-regulated |
| BICD2 | 3.45E-07 | 7.09E-09 | -6.795284 | 10.11560416 | -1.52596 | 1.52596 | Down-regulated |
| CDC37 | 3.96E-06 | 1.35E-07 | -6.02069 | 7.24912888 | -1.525761 | 1.525761 | Down-regulated |
| AHR | 2.58E-05 | 1.33E-06 | -5.405999 | 5.02561277 | -1.524687 | 1.524687 | Down-regulated |
| ATP11B | 9.15E-09 | 8.92E-11 | -7.938291 | 14.38611162 | -1.524576 | 1.524576 | Down-regulated |
| SMIM14 | 4.98E-04 | 5.57E-05 | -4.357801 | 1.43033434 | -1.524195 | 1.524195 | Down-regulated |
| MYOM1 | 5.12E-08 | 7.26E-10 | -7.390575 | 12.33978964 | -1.524087 | 1.524087 | Down-regulated |
| GIN1 | 2.97E-08 | 3.79E-10 | -7.560256 | 12.97432167 | -1.523925 | 1.523925 | Down-regulated |
| CSTA | 5.87E-07 | 1.34E-08 | -6.629656 | 9.4988733 | -1.523809 | 1.523809 | Down-regulated |
| GNB1 | 2.66E-05 | 1.38E-06 | -5.39608 | 4.99027781 | -1.523175 | 1.523175 | Down-regulated |
| BCCIP | 5.09E-04 | 5.73E-05 | -4.349703 | 1.40388576 | -1.523161 | 1.523161 | Down-regulated |
| ISG20 | 2.55E-07 | 4.96E-09 | -6.888546 | 10.46344964 | -1.52304 | 1.52304 | Down-regulated |
| LRP10 | 7.71E-04 | 9.92E-05 | -4.187592 | 0.8796099 | -1.522243 | 1.522243 | Down-regulated |
| MRPL53 | 4.94E-05 | 2.93E-06 | -5.190125 | 4.26133917 | -1.521963 | 1.521963 | Down-regulated |
| TSHZ3 | 5.57E-03 | 1.82E-03 | -3.272416 | -1.86094465 | -1.521858 | 1.521858 | Down-regulated |
| NME1 | 3.76E-04 | 3.91E-05 | -4.460823 | 1.76890324 | -1.521254 | 1.521254 | Down-regulated |
| FCRLB | 1.07E-05 | 4.47E-07 | -5.70056 | 6.0834534 | -1.521225 | 1.521225 | Down-regulated |
| DCTN4 | 1.01E-09 | 5.92E-12 | -8.652121 | 17.03518063 | -1.521148 | 1.521148 | Down-regulated |
| ASXL1 | 1.18E-09 | 7.33E-12 | -8.595423 | 16.82590483 | -1.521137 | 1.521137 | Down-regulated |
| KDELR2 | 3.78E-04 | 3.93E-05 | -4.459286 | 1.76382527 | -1.520623 | 1.520623 | Down-regulated |
| ETFA | 5.56E-05 | 3.41E-06 | -5.149206 | 4.11765377 | -1.520542 | 1.520542 | Down-regulated |
| RBKS | 1.57E-06 | 4.51E-08 | -6.309814 | 8.31301409 | -1.519951 | 1.519951 | Down-regulated |
| RBM27 | 9.28E-03 | 4.48E-03 | -2.959829 | -2.69474417 | -1.519949 | 1.519949 | Down-regulated |
| PCID2 | 2.66E-07 | 5.25E-09 | -6.874131 | 10.40966031 | -1.519118 | 1.519118 | Down-regulated |
| GABARAPL2 | 3.88E-07 | 8.14E-09 | -6.759357 | 9.98170422 | -1.519103 | 1.519103 | Down-regulated |
| FKBP1A | 1.21E-07 | 2.03E-09 | -7.121996 | 11.33543095 | -1.518788 | 1.518788 | Down-regulated |
| INPPL1 | 2.47E-11 | 5.99E-14 | -9.884454 | 21.51503892 | -1.518688 | 1.518688 | Down-regulated |
| PPP2R2B | 8.37E-06 | 3.33E-07 | -5.779627 | 6.36995587 | -1.518522 | 1.518522 | Down-regulated |
| AP3S2 | 1.19E-04 | 8.94E-06 | -4.881248 | 3.18711286 | -1.518011 | 1.518011 | Down-regulated |
| SKP1 | 5.49E-04 | 6.34E-05 | -4.320059 | 1.30726868 | -1.517865 | 1.517865 | Down-regulated |
| NAA60 | 5.16E-05 | 3.10E-06 | -5.175265 | 4.20911307 | -1.517798 | 1.517798 | Down-regulated |
| HSP90B1 | 9.34E-07 | 2.39E-08 | -6.476757 | 8.93100814 | -1.516659 | 1.516659 | Down-regulated |
| NDST2 | 4.77E-04 | 5.28E-05 | -4.373678 | 1.48226331 | -1.516605 | 1.516605 | Down-regulated |
| DNAJB12 | 1.39E-05 | 6.19E-07 | -5.613331 | 5.76856353 | -1.516506 | 1.516506 | Down-regulated |
| DBI | 3.37E-10 | 1.47E-12 | -9.021211 | 18.39151103 | -1.516467 | 1.516467 | Down-regulated |
| SDHAP1 | 4.72E-04 | 5.21E-05 | -4.37758 | 1.49504001 | -1.516388 | 1.516388 | Down-regulated |
| SLC25A3 | 7.19E-08 | 1.10E-09 | -7.28271 | 11.93636612 | -1.51552 | 1.51552 | Down-regulated |
| TFPT | 2.51E-06 | 7.77E-08 | -6.166289 | 7.78375663 | -1.515128 | 1.515128 | Down-regulated |
| CEBPG | 3.23E-06 | 1.05E-07 | -6.086964 | 7.49217344 | -1.514955 | 1.514955 | Down-regulated |
| MIF4GD | 2.71E-07 | 5.41E-09 | -6.866229 | 10.38017873 | -1.514931 | 1.514931 | Down-regulated |
| NSMCE2 | 1.09E-04 | 7.93E-06 | -4.9147 | 3.30224245 | -1.514326 | 1.514326 | Down-regulated |
| LOC148413 | 7.46E-07 | 1.81E-08 | -6.550061 | 9.20305686 | -1.513911 | 1.513911 | Down-regulated |
| NUDT18 | 5.87E-04 | 6.94E-05 | -4.293491 | 1.22095493 | -1.513468 | 1.513468 | Down-regulated |
| INPP1 | 3.13E-08 | 4.05E-10 | -7.542579 | 12.90823333 | -1.513466 | 1.513466 | Down-regulated |
| FAM200A | 2.82E-06 | 8.91E-08 | -6.130198 | 7.65100655 | -1.512947 | 1.512947 | Down-regulated |
| ETFDH | 2.37E-05 | 1.20E-06 | -5.434093 | 5.12580007 | -1.512933 | 1.512933 | Down-regulated |
| CHMP5 | 2.34E-07 | 4.43E-09 | -6.918244 | 10.57428742 | -1.512479 | 1.512479 | Down-regulated |
| LRWD1 | 6.04E-08 | 8.89E-10 | -7.337558 | 12.14149736 | -1.511777 | 1.511777 | Down-regulated |
| BRD2 | 9.70E-10 | 5.60E-12 | -8.66694 | 17.08984078 | -1.51111 | 1.51111 | Down-regulated |
| DMAP1 | 2.67E-04 | 2.49E-05 | -4.590842 | 2.20147243 | -1.511106 | 1.511106 | Down-regulated |
| URM1 | 1.01E-06 | 2.63E-08 | -6.45205 | 8.83940197 | -1.510843 | 1.510843 | Down-regulated |
| HPRT1 | 1.28E-10 | 4.62E-13 | -9.331771 | 19.52370798 | -1.510679 | 1.510679 | Down-regulated |
| CCDC93 | 2.00E-03 | 3.62E-04 | -3.794163 | -0.34772649 | -1.510091 | 1.510091 | Down-regulated |
| APBA3 | 4.04E-04 | 4.26E-05 | -4.436187 | 1.68759584 | -1.51009 | 1.51009 | Down-regulated |
| MPC2 | 2.75E-06 | 8.59E-08 | -6.139635 | 7.68570374 | -1.509356 | 1.509356 | Down-regulated |
| RHBDF2 | 1.94E-09 | 1.34E-11 | -8.435976 | 16.23618877 | -1.509083 | 1.509083 | Down-regulated |
| PSMG3 | 1.15E-02 | 6.48E-03 | -2.826918 | -3.03087619 | -1.508893 | 1.508893 | Down-regulated |
| NDUFS1 | 6.46E-06 | 2.41E-07 | -5.866013 | 6.68407216 | -1.508752 | 1.508752 | Down-regulated |
| ARMC7 | 3.48E-03 | 8.38E-04 | -3.527526 | -1.13877972 | -1.508541 | 1.508541 | Down-regulated |
| LPCAT1 | 6.33E-08 | 9.43E-10 | -7.322301 | 12.08443431 | -1.508488 | 1.508488 | Down-regulated |
| IFI6 | 6.99E-06 | 2.65E-07 | -5.840134 | 6.58985677 | -1.508436 | 1.508436 | Down-regulated |
| RRP36 | 1.64E-03 | 2.75E-04 | -3.87952 | -0.08724267 | -1.508432 | 1.508432 | Down-regulated |
| NUCB2 | 4.71E-09 | 3.93E-11 | -8.153475 | 15.18755308 | -1.507659 | 1.507659 | Down-regulated |
| LARP4 | 3.65E-06 | 1.22E-07 | -6.0467 | 7.34445007 | -1.507549 | 1.507549 | Down-regulated |
| GBGT1 | 6.82E-07 | 1.61E-08 | -6.580623 | 9.31659242 | -1.5074 | 1.5074 | Down-regulated |
| H3F3B | 3.86E-07 | 8.09E-09 | -6.760953 | 9.98765176 | -1.506735 | 1.506735 | Down-regulated |
| PRMT5 | 6.76E-10 | 3.55E-12 | -8.787861 | 17.53525148 | -1.506181 | 1.506181 | Down-regulated |
| UQCR11 | 1.45E-08 | 1.55E-10 | -7.793646 | 13.84638267 | -1.505807 | 1.505807 | Down-regulated |
| CCDC51 | 2.11E-06 | 6.35E-08 | -6.219689 | 7.98042672 | -1.504983 | 1.504983 | Down-regulated |
| TMEM230 | 1.13E-04 | 8.38E-06 | -4.899178 | 3.24878461 | -1.504951 | 1.504951 | Down-regulated |
| UPK3BL | 1.21E-05 | 5.21E-07 | -5.65953 | 5.93517711 | -1.504716 | 1.504716 | Down-regulated |
| DARS | 2.99E-05 | 1.59E-06 | -5.357238 | 4.85210393 | -1.503958 | 1.503958 | Down-regulated |
| FTH1 | 3.41E-04 | 3.44E-05 | -4.498346 | 1.8931504 | -1.503912 | 1.503912 | Down-regulated |
| UQCC2 | 3.62E-03 | 8.94E-04 | -3.506799 | -1.1987642 | -1.503847 | 1.503847 | Down-regulated |
| SETMAR | 1.13E-05 | 4.78E-07 | -5.682689 | 6.01883429 | -1.503725 | 1.503725 | Down-regulated |
| RING1 | 1.47E-07 | 2.56E-09 | -7.061696 | 11.11006258 | -1.503723 | 1.503723 | Down-regulated |
| TMEM97 | 2.02E-03 | 3.67E-04 | -3.789647 | -0.36141327 | -1.503495 | 1.503495 | Down-regulated |
| TP53I3 | 5.33E-04 | 6.10E-05 | -4.331594 | 1.34482402 | -1.503454 | 1.503454 | Down-regulated |
| IRF1 | 3.25E-08 | 4.24E-10 | -7.531093 | 12.86528411 | -1.503448 | 1.503448 | Down-regulated |
| MCM6 | 7.41E-07 | 1.79E-08 | -6.553251 | 9.21490699 | -1.503396 | 1.503396 | Down-regulated |
| FCGR1B | 1.34E-04 | 1.03E-05 | -4.84038 | 3.04688659 | -1.503346 | 1.503346 | Down-regulated |
| TFE3 | 5.83E-10 | 2.90E-12 | -8.841019 | 17.73070121 | -1.50283 | 1.50283 | Down-regulated |
| TBCB | 6.93E-08 | 1.05E-09 | -7.294782 | 11.98151559 | -1.502609 | 1.502609 | Down-regulated |
| HEBP1 | 8.91E-06 | 3.59E-07 | -5.75921 | 6.2958774 | -1.502346 | 1.502346 | Down-regulated |
| NKIRAS1 | 2.25E-05 | 1.12E-06 | -5.452363 | 5.19103901 | -1.502016 | 1.502016 | Down-regulated |
| NCBP1 | 3.41E-03 | 8.09E-04 | -3.539058 | -1.10530746 | -1.501692 | 1.501692 | Down-regulated |
| NARF | 1.36E-03 | 2.12E-04 | -3.958742 | 0.15748334 | -1.501451 | 1.501451 | Down-regulated |
| ZNF318 | 3.85E-07 | 8.04E-09 | -6.762334 | 9.99279731 | -1.500297 | 1.500297 | Down-regulated |
| ATXN1 | 4.20E-06 | 1.44E-07 | -6.002755 | 7.18344927 | -1.500065 | 1.500065 | Down-regulated |
| MRPL15 | 4.76E-07 | 1.04E-08 | -6.694702 | 9.7409001 | -1.499965 | 1.499965 | Down-regulated |
| MT1A | 3.02E-08 | 3.87E-10 | -7.554686 | 12.9534968 | -1.499744 | 1.499744 | Down-regulated |
| CHSY1 | 1.53E-04 | 1.22E-05 | -4.79295 | 2.88474375 | -1.499536 | 1.499536 | Down-regulated |
| MRPS12 | 3.46E-06 | 1.14E-07 | -6.064486 | 7.40968256 | -1.499483 | 1.499483 | Down-regulated |
| NUCB1 | 1.47E-08 | 1.58E-10 | -7.788435 | 13.82692589 | -1.499294 | 1.499294 | Down-regulated |
| RBM39 | 2.99E-05 | 1.59E-06 | -5.357079 | 4.85153955 | -1.498503 | 1.498503 | Down-regulated |
| MMADHC | 1.86E-05 | 8.85E-07 | -5.516721 | 5.42136427 | -1.498444 | 1.498444 | Down-regulated |
| DSC2 | 1.87E-05 | 8.93E-07 | -5.51439 | 5.41301051 | -1.498102 | 1.498102 | Down-regulated |
| FAR2 | 5.05E-07 | 1.12E-08 | -6.67643 | 9.67288762 | -1.49797 | 1.49797 | Down-regulated |
| PPM1G | 1.62E-04 | 1.33E-05 | -4.770173 | 2.80711667 | -1.497911 | 1.497911 | Down-regulated |
| CFLAR | 7.80E-04 | 1.01E-04 | -4.182704 | 0.86396074 | -1.497103 | 1.497103 | Down-regulated |
| AGTRAP | 2.08E-04 | 1.83E-05 | -4.679208 | 2.4986466 | -1.497101 | 1.497101 | Down-regulated |
| ELF4 | 1.92E-04 | 1.65E-05 | -4.707946 | 2.59582715 | -1.496409 | 1.496409 | Down-regulated |
| TAPBPL | 6.35E-10 | 3.23E-12 | -8.812261 | 17.62499151 | -1.496395 | 1.496395 | Down-regulated |
| PDCL | 5.99E-07 | 1.37E-08 | -6.623691 | 9.47668983 | -1.495845 | 1.495845 | Down-regulated |
| DDRGK1 | 1.90E-06 | 5.57E-08 | -6.254115 | 8.10737607 | -1.495562 | 1.495562 | Down-regulated |
| NPL | 1.93E-07 | 3.54E-09 | -6.976881 | 10.79321614 | -1.495296 | 1.495296 | Down-regulated |
| STK3 | 1.33E-07 | 2.26E-09 | -7.094037 | 11.23092643 | -1.49527 | 1.49527 | Down-regulated |
| DIAPH2 | 6.03E-05 | 3.76E-06 | -5.12173 | 4.02139897 | -1.494898 | 1.494898 | Down-regulated |
| PTBP1 | 1.42E-04 | 1.11E-05 | -4.820162 | 2.97768877 | -1.494539 | 1.494539 | Down-regulated |
| MRPL11 | 3.10E-04 | 3.05E-05 | -4.533104 | 2.00867356 | -1.4944 | 1.4944 | Down-regulated |
| GADD45G | 3.33E-04 | 3.34E-05 | -4.506852 | 1.92138464 | -1.494392 | 1.494392 | Down-regulated |
| TOPORS | 2.48E-04 | 2.29E-05 | -4.615293 | 2.28344867 | -1.494076 | 1.494076 | Down-regulated |
| NANS | 3.20E-09 | 2.47E-11 | -8.275411 | 15.64072101 | -1.493941 | 1.493941 | Down-regulated |
| MDP1 | 1.17E-07 | 1.95E-09 | -7.13236 | 11.37417524 | -1.493289 | 1.493289 | Down-regulated |
| RETN | 7.11E-03 | 2.79E-03 | -3.126009 | -2.25879458 | -1.492315 | 1.492315 | Down-regulated |
| IAH1 | 6.25E-07 | 1.44E-08 | -6.609397 | 9.4235419 | -1.492256 | 1.492256 | Down-regulated |
| PLEK | 2.36E-05 | 1.19E-06 | -5.435594 | 5.13115717 | -1.492061 | 1.492061 | Down-regulated |
| SMARCC2 | 2.55E-07 | 4.95E-09 | -6.889011 | 10.46518546 | -1.492022 | 1.492022 | Down-regulated |
| ATPAF1 | 4.93E-05 | 2.92E-06 | -5.19119 | 4.26508412 | -1.49146 | 1.49146 | Down-regulated |
| SNTB2 | 6.96E-08 | 1.05E-09 | -7.293053 | 11.97504808 | -1.491241 | 1.491241 | Down-regulated |
| TMEM79 | 3.55E-06 | 1.18E-07 | -6.05552 | 7.37679357 | -1.491129 | 1.491129 | Down-regulated |
| UBA7 | 1.68E-05 | 7.82E-07 | -5.550188 | 5.54144594 | -1.491051 | 1.491051 | Down-regulated |
| PCBP2 | 1.33E-07 | 2.26E-09 | -7.094579 | 11.23295381 | -1.490677 | 1.490677 | Down-regulated |
| SEC61G | 4.67E-09 | 3.87E-11 | -8.157264 | 15.20164657 | -1.490186 | 1.490186 | Down-regulated |
| SGF29 | 1.36E-05 | 5.99E-07 | -5.621944 | 5.79959661 | -1.489642 | 1.489642 | Down-regulated |
| IST1 | 1.84E-05 | 8.71E-07 | -5.521148 | 5.43723822 | -1.489274 | 1.489274 | Down-regulated |
| CKS2 | 1.83E-06 | 5.34E-08 | -6.265135 | 8.14803653 | -1.487997 | 1.487997 | Down-regulated |
| LHFPL2 | 1.24E-05 | 5.35E-07 | -5.652385 | 5.90938357 | -1.487663 | 1.487663 | Down-regulated |
| C18orf21 | 5.58E-08 | 8.05E-10 | -7.363355 | 12.23798152 | -1.48763 | 1.48763 | Down-regulated |
| CENPBD1 | 2.55E-07 | 4.96E-09 | -6.888953 | 10.46496568 | -1.487621 | 1.487621 | Down-regulated |
| UBALD2 | 4.00E-05 | 2.25E-06 | -5.262474 | 4.51634342 | -1.48757 | 1.48757 | Down-regulated |
| AKR1A1 | 8.66E-07 | 2.18E-08 | -6.501367 | 9.02229727 | -1.486919 | 1.486919 | Down-regulated |
| STX12 | 8.87E-05 | 6.10E-06 | -4.987923 | 3.55531808 | -1.486327 | 1.486327 | Down-regulated |
| MBTD1 | 7.92E-07 | 1.95E-08 | -6.53057 | 9.13068285 | -1.485721 | 1.485721 | Down-regulated |
| ADCY4 | 6.84E-04 | 8.52E-05 | -4.233062 | 1.02563967 | -1.485547 | 1.485547 | Down-regulated |
| TRIP12 | 6.10E-07 | 1.40E-08 | -6.616625 | 9.45041378 | -1.485055 | 1.485055 | Down-regulated |
| CCDC120 | 6.99E-06 | 2.65E-07 | -5.840295 | 6.59044263 | -1.484416 | 1.484416 | Down-regulated |
| SNRNP25 | 9.60E-10 | 5.46E-12 | -8.673511 | 17.1140719 | -1.484035 | 1.484035 | Down-regulated |
| OTULIN | 2.98E-07 | 6.03E-09 | -6.837687 | 10.273713 | -1.483734 | 1.483734 | Down-regulated |
| LCOR | 2.75E-10 | 1.14E-12 | -9.090343 | 18.64429977 | -1.483482 | 1.483482 | Down-regulated |
| SH3KBP1 | 9.17E-04 | 1.25E-04 | -4.119453 | 0.66232176 | -1.48313 | 1.48313 | Down-regulated |
| KRCC1 | 7.99E-06 | 3.13E-07 | -5.795827 | 6.42877714 | -1.482979 | 1.482979 | Down-regulated |
| ZNF438 | 1.66E-04 | 1.37E-05 | -4.760915 | 2.7756079 | -1.482706 | 1.482706 | Down-regulated |
| GSTP1 | 1.18E-03 | 1.74E-04 | -4.018963 | 0.34536541 | -1.482295 | 1.482295 | Down-regulated |
| NDUFA6 | 1.64E-05 | 7.59E-07 | -5.558207 | 5.57025143 | -1.482098 | 1.482098 | Down-regulated |
| PSMC5 | 1.30E-03 | 2.00E-04 | -3.976586 | 0.21298917 | -1.481754 | 1.481754 | Down-regulated |
| CRISPLD2 | 3.46E-04 | 3.51E-05 | -4.492179 | 1.87269644 | -1.481695 | 1.481695 | Down-regulated |
| DDX42 | 3.53E-10 | 1.56E-12 | -9.005762 | 18.33496026 | -1.481652 | 1.481652 | Down-regulated |
| HERPUD2 | 9.88E-06 | 4.08E-07 | -5.724911 | 6.1715838 | -1.481526 | 1.481526 | Down-regulated |
| RPS3A | 7.19E-03 | 2.85E-03 | -3.118699 | -2.2783268 | -1.481007 | 1.481007 | Down-regulated |
| LRPAP1 | 1.57E-04 | 1.27E-05 | -4.782624 | 2.84953159 | -1.480938 | 1.480938 | Down-regulated |
| MASTL | 7.86E-07 | 1.93E-08 | -6.532872 | 9.13923021 | -1.48082 | 1.48082 | Down-regulated |
| KDELR1 | 1.15E-05 | 4.86E-07 | -5.678215 | 6.00266758 | -1.480735 | 1.480735 | Down-regulated |
| CPSF4 | 1.30E-06 | 3.57E-08 | -6.371064 | 8.5394755 | -1.479883 | 1.479883 | Down-regulated |
| HELZ2 | 8.50E-08 | 1.33E-09 | -7.232634 | 11.74909184 | -1.478728 | 1.478728 | Down-regulated |
| LCN2 | 5.31E-08 | 7.61E-10 | -7.378004 | 12.29277093 | -1.477974 | 1.477974 | Down-regulated |
| GCHFR | 5.60E-08 | 8.10E-10 | -7.361943 | 12.23270085 | -1.477792 | 1.477792 | Down-regulated |
| RAB1A | 5.15E-06 | 1.83E-07 | -5.93903 | 6.95041635 | -1.477687 | 1.477687 | Down-regulated |
| ITGAE | 8.84E-07 | 2.23E-08 | -6.494654 | 8.99739085 | -1.477457 | 1.477457 | Down-regulated |
| TSEN34 | 1.20E-08 | 1.24E-10 | -7.852627 | 14.06654871 | -1.47716 | 1.47716 | Down-regulated |
| TMEM138 | 5.67E-08 | 8.21E-10 | -7.358188 | 12.21865938 | -1.476977 | 1.476977 | Down-regulated |
| LRRC41 | 2.31E-03 | 4.49E-04 | -3.726507 | -0.5517573 | -1.476765 | 1.476765 | Down-regulated |
| NDUFS4 | 4.39E-05 | 2.53E-06 | -5.230592 | 4.40382201 | -1.475945 | 1.475945 | Down-regulated |
| ANG | 1.62E-08 | 1.77E-10 | -7.758422 | 13.71485009 | -1.475632 | 1.475632 | Down-regulated |
| SPEN | 8.97E-07 | 2.28E-08 | -6.489687 | 8.97896454 | -1.475513 | 1.475513 | Down-regulated |
| FAM35A | 1.42E-06 | 3.97E-08 | -6.343583 | 8.43782597 | -1.474555 | 1.474555 | Down-regulated |
| IDNK | 3.98E-03 | 1.04E-03 | -3.458037 | -1.33899209 | -1.47426 | 1.47426 | Down-regulated |
| DYNLL2 | 8.32E-07 | 2.07E-08 | -6.514344 | 9.07045333 | -1.474129 | 1.474129 | Down-regulated |
| EXOC6 | 1.63E-04 | 1.33E-05 | -4.76919 | 2.80376832 | -1.474081 | 1.474081 | Down-regulated |
| GOSR2 | 5.28E-07 | 1.18E-08 | -6.66215 | 9.61974984 | -1.473883 | 1.473883 | Down-regulated |
| TRIP11 | 4.91E-08 | 6.85E-10 | -7.405602 | 12.39599057 | -1.473754 | 1.473754 | Down-regulated |
| DSCC1 | 2.49E-09 | 1.83E-11 | -8.354546 | 15.93438926 | -1.473699 | 1.473699 | Down-regulated |
| WSB2 | 2.21E-04 | 1.97E-05 | -4.657285 | 2.4246861 | -1.473 | 1.473 | Down-regulated |
| MPDU1 | 2.25E-07 | 4.24E-09 | -6.929748 | 10.61722808 | -1.472621 | 1.472621 | Down-regulated |
| MTCH1 | 4.94E-09 | 4.17E-11 | -8.137532 | 15.1282449 | -1.472512 | 1.472512 | Down-regulated |
| DICER1 | 2.12E-06 | 6.39E-08 | -6.21798 | 7.9741306 | -1.472463 | 1.472463 | Down-regulated |
| EMC9 | 5.01E-03 | 1.52E-03 | -3.332986 | -1.69273137 | -1.472359 | 1.472359 | Down-regulated |
| TMEM9B | 1.41E-05 | 6.25E-07 | -5.610552 | 5.75855034 | -1.472098 | 1.472098 | Down-regulated |
| BRCA1 | 6.00E-07 | 1.37E-08 | -6.62223 | 9.47125425 | -1.471985 | 1.471985 | Down-regulated |
| FAM209B | 2.45E-03 | 4.89E-04 | -3.699532 | -0.63249263 | -1.471888 | 1.471888 | Down-regulated |
| FERMT3 | 4.45E-04 | 4.85E-05 | -4.398198 | 1.56264171 | -1.471846 | 1.471846 | Down-regulated |
| PIK3CA | 7.24E-06 | 2.78E-07 | -5.827892 | 6.54532117 | -1.471833 | 1.471833 | Down-regulated |
| GEMIN6 | 3.10E-09 | 2.38E-11 | -8.285367 | 15.67768736 | -1.471234 | 1.471234 | Down-regulated |
| KHSRP | 7.15E-03 | 2.83E-03 | -3.121699 | -2.2703144 | -1.470732 | 1.470732 | Down-regulated |
| TEX261 | 6.13E-05 | 3.86E-06 | -5.114823 | 3.99723152 | -1.470452 | 1.470452 | Down-regulated |
| BRD9 | 1.85E-04 | 1.57E-05 | -4.721571 | 2.64198852 | -1.470288 | 1.470288 | Down-regulated |
| ARAP3 | 7.41E-10 | 3.97E-12 | -8.75798 | 17.42528932 | -1.470161 | 1.470161 | Down-regulated |
| DPRXP4 | 7.23E-04 | 9.15E-05 | -4.211633 | 0.9567177 | -1.469757 | 1.469757 | Down-regulated |
| RPL7L1 | 1.89E-04 | 1.62E-05 | -4.712938 | 2.61273091 | -1.469587 | 1.469587 | Down-regulated |
| CIDEB | 3.36E-06 | 1.10E-07 | -6.073627 | 7.44322014 | -1.469081 | 1.469081 | Down-regulated |
| MBP | 4.17E-07 | 8.87E-09 | -6.736735 | 9.89742516 | -1.468931 | 1.468931 | Down-regulated |
| POLB | 1.53E-04 | 1.23E-05 | -4.792278 | 2.88245401 | -1.468707 | 1.468707 | Down-regulated |
| GSR | 2.31E-03 | 4.48E-04 | -3.727118 | -0.54992421 | -1.468505 | 1.468505 | Down-regulated |
| CYFIP2 | 3.31E-05 | 1.80E-06 | -5.324193 | 4.73480342 | -1.468046 | 1.468046 | Down-regulated |
| ERCC1 | 2.63E-08 | 3.24E-10 | -7.601255 | 13.12759406 | -1.467768 | 1.467768 | Down-regulated |
| C1orf216 | 1.61E-04 | 1.31E-05 | -4.773615 | 2.81883869 | -1.467598 | 1.467598 | Down-regulated |
| ZC3H3 | 1.44E-04 | 1.14E-05 | -4.813203 | 2.95390085 | -1.466119 | 1.466119 | Down-regulated |
| CLEC16A | 1.13E-04 | 8.37E-06 | -4.899663 | 3.25045304 | -1.465998 | 1.465998 | Down-regulated |
| RAX2 | 1.76E-03 | 3.02E-04 | -3.850454 | -0.17632221 | -1.465178 | 1.465178 | Down-regulated |
| CDC16 | 1.58E-08 | 1.72E-10 | -7.766757 | 13.74597563 | -1.464725 | 1.464725 | Down-regulated |
| MRPS7 | 2.12E-06 | 6.40E-08 | -6.217564 | 7.97259753 | -1.464653 | 1.464653 | Down-regulated |
| TIMP2 | 5.12E-04 | 5.79E-05 | -4.346632 | 1.39386292 | -1.464435 | 1.464435 | Down-regulated |
| CD164 | 1.43E-03 | 2.28E-04 | -3.937184 | 0.09061011 | -1.463624 | 1.463624 | Down-regulated |
| GADD45B | 2.78E-06 | 8.71E-08 | -6.136221 | 7.67314879 | -1.462158 | 1.462158 | Down-regulated |
| GOLGA7 | 6.86E-04 | 8.55E-05 | -4.231888 | 1.02185901 | -1.46208 | 1.46208 | Down-regulated |
| PMPCB | 1.77E-09 | 1.17E-11 | -8.471786 | 16.36877752 | -1.461899 | 1.461899 | Down-regulated |
| CCBE1 | 2.13E-04 | 1.88E-05 | -4.671807 | 2.47366277 | -1.461894 | 1.461894 | Down-regulated |
| POLR2C | 2.91E-06 | 9.23E-08 | -6.120807 | 7.6164887 | -1.461882 | 1.461882 | Down-regulated |
| BCL2L1 | 1.94E-03 | 3.47E-04 | -3.807221 | -0.30809721 | -1.461752 | 1.461752 | Down-regulated |
| PIP4K2C | 6.56E-05 | 4.19E-06 | -5.092049 | 3.91762375 | -1.460746 | 1.460746 | Down-regulated |
| PI4KB | 1.45E-03 | 2.31E-04 | -3.932502 | 0.07611349 | -1.460651 | 1.460651 | Down-regulated |
| ACLY | 9.67E-05 | 6.85E-06 | -4.9556 | 3.44342435 | -1.460579 | 1.460579 | Down-regulated |
| TMEM120A | 6.27E-07 | 1.45E-08 | -6.607859 | 9.41782221 | -1.460506 | 1.460506 | Down-regulated |
| ZBTB8OS | 8.71E-03 | 3.99E-03 | -3.000935 | -2.58850226 | -1.459858 | 1.459858 | Down-regulated |
| PGLYRP1 | 4.09E-04 | 4.34E-05 | -4.430618 | 1.66924746 | -1.459792 | 1.459792 | Down-regulated |
| ERI1 | 1.19E-05 | 5.08E-07 | -5.666141 | 5.95904943 | -1.459665 | 1.459665 | Down-regulated |
| C7orf50 | 3.13E-09 | 2.41E-11 | -8.282012 | 15.66523332 | -1.459538 | 1.459538 | Down-regulated |
| TRMT1L | 5.46E-07 | 1.22E-08 | -6.652782 | 9.58489188 | -1.459472 | 1.459472 | Down-regulated |
| TUBG1 | 2.22E-04 | 1.99E-05 | -4.655741 | 2.41948308 | -1.459148 | 1.459148 | Down-regulated |
| LYZ | 6.07E-03 | 2.11E-03 | -3.222569 | -1.99780578 | -1.458953 | 1.458953 | Down-regulated |
| RAB35 | 5.12E-08 | 7.23E-10 | -7.391337 | 12.34263976 | -1.456521 | 1.456521 | Down-regulated |
| NUB1 | 9.41E-05 | 6.59E-06 | -4.966256 | 3.48028123 | -1.456123 | 1.456123 | Down-regulated |
| SDF2L1 | 2.67E-05 | 1.39E-06 | -5.394843 | 4.98587229 | -1.455508 | 1.455508 | Down-regulated |
| DYRK4 | 1.10E-02 | 6.01E-03 | -2.854061 | -2.96316122 | -1.454641 | 1.454641 | Down-regulated |
| RAB2A | 6.50E-10 | 3.33E-12 | -8.804253 | 17.59554303 | -1.454359 | 1.454359 | Down-regulated |
| ANKIB1 | 5.65E-03 | 1.86E-03 | -3.264413 | -1.88301493 | -1.454245 | 1.454245 | Down-regulated |
| CUTA | 1.79E-06 | 5.22E-08 | -6.27121 | 8.17045586 | -1.454038 | 1.454038 | Down-regulated |
| GDI2 | 4.20E-04 | 4.49E-05 | -4.420872 | 1.6371583 | -1.453688 | 1.453688 | Down-regulated |
| DCTPP1 | 6.37E-06 | 2.36E-07 | -5.871217 | 6.70303112 | -1.453483 | 1.453483 | Down-regulated |
| SCCPDH | 1.67E-05 | 7.77E-07 | -5.551723 | 5.5469598 | -1.45307 | 1.45307 | Down-regulated |
| POLR1D | 5.17E-04 | 5.85E-05 | -4.343648 | 1.38412452 | -1.452878 | 1.452878 | Down-regulated |
| TRIM35 | 3.84E-04 | 4.01E-05 | -4.454001 | 1.74636778 | -1.452557 | 1.452557 | Down-regulated |
| SNRPC | 1.13E-04 | 8.36E-06 | -4.9 | 3.2516144 | -1.452228 | 1.452228 | Down-regulated |
| DUSP28 | 5.60E-05 | 3.44E-06 | -5.14661 | 4.10855292 | -1.452178 | 1.452178 | Down-regulated |
| C12orf45 | 8.27E-06 | 3.27E-07 | -5.784212 | 6.38659676 | -1.451579 | 1.451579 | Down-regulated |
| RPL34 | 9.44E-09 | 9.27E-11 | -7.928226 | 14.34857794 | -1.451311 | 1.451311 | Down-regulated |
| ASXL2 | 7.43E-05 | 4.90E-06 | -5.048748 | 3.76662039 | -1.450906 | 1.450906 | Down-regulated |
| SLFN11 | 3.40E-03 | 8.06E-04 | -3.540164 | -1.10209552 | -1.450598 | 1.450598 | Down-regulated |
| BAP1 | 2.68E-04 | 2.51E-05 | -4.588796 | 2.19462175 | -1.450243 | 1.450243 | Down-regulated |
| HIST1H4H | 9.91E-05 | 7.05E-06 | -4.947468 | 3.41531652 | -1.450062 | 1.450062 | Down-regulated |
| RRAGC | 8.86E-07 | 2.24E-08 | -6.493878 | 8.99451376 | -1.449783 | 1.449783 | Down-regulated |
| ZSWIM1 | 4.10E-06 | 1.40E-07 | -6.010291 | 7.21104059 | -1.449257 | 1.449257 | Down-regulated |
| LIN54 | 3.76E-05 | 2.10E-06 | -5.281296 | 4.58287724 | -1.448739 | 1.448739 | Down-regulated |
| TMEM165 | 3.67E-06 | 1.23E-07 | -6.045161 | 7.3388091 | -1.448702 | 1.448702 | Down-regulated |
| SPC24 | 1.39E-04 | 1.08E-05 | -4.827758 | 3.00367478 | -1.447977 | 1.447977 | Down-regulated |
| PI4K2B | 3.36E-03 | 7.93E-04 | -3.545433 | -1.0867757 | -1.447664 | 1.447664 | Down-regulated |
| RAMP2-AS1 | 5.09E-09 | 4.34E-11 | -8.127006 | 15.08908373 | -1.447594 | 1.447594 | Down-regulated |
| GMFB | 8.74E-05 | 5.98E-06 | -4.993445 | 3.5744635 | -1.446673 | 1.446673 | Down-regulated |
| YIPF3 | 1.14E-04 | 8.40E-06 | -4.898413 | 3.24614887 | -1.446603 | 1.446603 | Down-regulated |
| LDHA | 2.89E-05 | 1.52E-06 | -5.369231 | 4.89473443 | -1.446592 | 1.446592 | Down-regulated |
| BCYRN1 | 7.85E-08 | 1.21E-09 | -7.256212 | 11.83726699 | -1.446485 | 1.446485 | Down-regulated |
| FAM206A | 1.20E-06 | 3.27E-08 | -6.394761 | 8.62717989 | -1.446463 | 1.446463 | Down-regulated |
| R3HDM1 | 2.12E-06 | 6.36E-08 | -6.219026 | 7.97798352 | -1.445435 | 1.445435 | Down-regulated |
| ACOX1 | 4.73E-09 | 3.95E-11 | -8.15168 | 15.1808755 | -1.445375 | 1.445375 | Down-regulated |
| CCT2 | 6.14E-06 | 2.26E-07 | -5.882983 | 6.74590884 | -1.445009 | 1.445009 | Down-regulated |
| STAT6 | 2.54E-09 | 1.88E-11 | -8.346856 | 15.90586754 | -1.444675 | 1.444675 | Down-regulated |
| SNORA3B | 5.21E-05 | 3.14E-06 | -5.171941 | 4.19743926 | -1.444668 | 1.444668 | Down-regulated |
| LMAN2L | 1.47E-04 | 1.17E-05 | -4.806183 | 2.92991711 | -1.444274 | 1.444274 | Down-regulated |
| UBE2D3 | 3.03E-05 | 1.62E-06 | -5.35231 | 4.83459709 | -1.443897 | 1.443897 | Down-regulated |
| WRAP53 | 1.10E-04 | 8.03E-06 | -4.911024 | 3.28957826 | -1.442802 | 1.442802 | Down-regulated |
| RPA4 | 5.02E-10 | 2.37E-12 | -8.895161 | 17.92953352 | -1.442365 | 1.442365 | Down-regulated |
| RAB32 | 1.27E-04 | 9.70E-06 | -4.858347 | 3.10847704 | -1.44224 | 1.44224 | Down-regulated |
| PPP1R35 | 2.16E-03 | 4.06E-04 | -3.758026 | -0.45697733 | -1.442164 | 1.442164 | Down-regulated |
| IL18 | 1.63E-05 | 7.46E-07 | -5.562999 | 5.58746949 | -1.441945 | 1.441945 | Down-regulated |
| EFEMP2 | 8.07E-09 | 7.74E-11 | -7.975387 | 14.52441123 | -1.441747 | 1.441747 | Down-regulated |
| PPP1CB | 7.15E-09 | 6.63E-11 | -8.016017 | 14.67582498 | -1.441565 | 1.441565 | Down-regulated |
| RNF213 | 6.67E-04 | 8.21E-05 | -4.243839 | 1.06036963 | -1.440849 | 1.440849 | Down-regulated |
| CISD1 | 7.14E-06 | 2.72E-07 | -5.833513 | 6.56576552 | -1.440555 | 1.440555 | Down-regulated |
| TOMM40L | 2.39E-04 | 2.18E-05 | -4.629459 | 2.33103258 | -1.440355 | 1.440355 | Down-regulated |
| FRG1 | 7.25E-11 | 2.32E-13 | -9.517283 | 20.19563763 | -1.440272 | 1.440272 | Down-regulated |
| TRIP6 | 2.83E-04 | 2.69E-05 | -4.569176 | 2.12899584 | -1.439909 | 1.439909 | Down-regulated |
| XRN1 | 3.45E-13 | 2.36E-16 | -11.431012 | 26.89785502 | -1.439828 | 1.439828 | Down-regulated |
| TROVE2 | 2.45E-05 | 1.25E-06 | -5.424234 | 5.09062585 | -1.439278 | 1.439278 | Down-regulated |
| MCM3 | 3.44E-06 | 1.13E-07 | -6.066315 | 7.41639329 | -1.439126 | 1.439126 | Down-regulated |
| GPR84 | 2.39E-08 | 2.89E-10 | -7.631331 | 13.24001153 | -1.438366 | 1.438366 | Down-regulated |
| WDFY1 | 2.98E-06 | 9.50E-08 | -6.113158 | 7.58837946 | -1.43827 | 1.43827 | Down-regulated |
| HPSE | 1.34E-05 | 5.90E-07 | -5.626025 | 5.8143078 | -1.438179 | 1.438179 | Down-regulated |
| UCN | 8.24E-05 | 5.54E-06 | -5.014503 | 3.64753722 | -1.438119 | 1.438119 | Down-regulated |
| POC5 | 3.89E-06 | 1.32E-07 | -6.026546 | 7.27058294 | -1.437936 | 1.437936 | Down-regulated |
| TMEM167A | 1.92E-08 | 2.23E-10 | -7.698896 | 13.49249396 | -1.43785 | 1.43785 | Down-regulated |
| GOLGA6L6 | 2.39E-07 | 4.57E-09 | -6.909943 | 10.54330388 | -1.437421 | 1.437421 | Down-regulated |
| AEN | 1.07E-10 | 3.77E-13 | -9.386632 | 19.72277186 | -1.437297 | 1.437297 | Down-regulated |
| FUT4 | 1.30E-04 | 9.95E-06 | -4.851111 | 3.08365916 | -1.437246 | 1.437246 | Down-regulated |
| HK2 | 2.54E-05 | 1.31E-06 | -5.41129 | 5.04446887 | -1.437089 | 1.437089 | Down-regulated |
| KBTBD2 | 9.92E-04 | 1.39E-04 | -4.087447 | 0.56091318 | -1.437001 | 1.437001 | Down-regulated |
| ZMYND15 | 2.77E-03 | 5.87E-04 | -3.641584 | -0.80472448 | -1.436412 | 1.436412 | Down-regulated |
| ARHGAP4 | 7.21E-07 | 1.73E-08 | -6.561809 | 9.24669191 | -1.436113 | 1.436113 | Down-regulated |
| TCP11L1 | 1.10E-04 | 8.06E-06 | -4.910273 | 3.28699009 | -1.435766 | 1.435766 | Down-regulated |
| PILRA | 2.05E-07 | 3.79E-09 | -6.959176 | 10.72709939 | -1.435748 | 1.435748 | Down-regulated |
| MGC72080 | 1.31E-08 | 1.37E-10 | -7.825867 | 13.96667192 | -1.435073 | 1.435073 | Down-regulated |
| SON | 1.29E-05 | 5.60E-07 | -5.640262 | 5.86564383 | -1.435071 | 1.435071 | Down-regulated |
| PAPOLG | 5.99E-05 | 3.74E-06 | -5.123731 | 4.02840314 | -1.435038 | 1.435038 | Down-regulated |
| SOD2 | 1.27E-04 | 9.64E-06 | -4.859937 | 3.11393147 | -1.435033 | 1.435033 | Down-regulated |
| NDUFB10 | 2.98E-06 | 9.47E-08 | -6.113856 | 7.59094424 | -1.434301 | 1.434301 | Down-regulated |
| C2orf47 | 3.29E-07 | 6.74E-09 | -6.808632 | 10.1653666 | -1.433985 | 1.433985 | Down-regulated |
| B3GNT8 | 1.19E-04 | 8.91E-06 | -4.882079 | 3.18997034 | -1.433399 | 1.433399 | Down-regulated |
| PSMA5 | 2.48E-04 | 2.29E-05 | -4.615529 | 2.28424315 | -1.433307 | 1.433307 | Down-regulated |
| C19orf60 | 6.94E-04 | 8.67E-05 | -4.227745 | 1.0085212 | -1.433046 | 1.433046 | Down-regulated |
| COPRS | 3.04E-07 | 6.17E-09 | -6.831496 | 10.25062314 | -1.432836 | 1.432836 | Down-regulated |
| ZNF600 | 2.18E-07 | 4.09E-09 | -6.939259 | 10.6527378 | -1.432733 | 1.432733 | Down-regulated |
| ARHGAP21 | 1.54E-06 | 4.39E-08 | -6.31719 | 8.34026457 | -1.432573 | 1.432573 | Down-regulated |
| ZNF668 | 1.37E-07 | 2.34E-09 | -7.085147 | 11.19770104 | -1.432471 | 1.432471 | Down-regulated |
| DCP2 | 1.81E-05 | 8.54E-07 | -5.526332 | 5.4558297 | -1.432442 | 1.432442 | Down-regulated |
| ALKBH6 | 1.43E-04 | 1.13E-05 | -4.816226 | 2.9642344 | -1.432421 | 1.432421 | Down-regulated |
| TMEM60 | 9.82E-05 | 6.97E-06 | -4.95054 | 3.42593467 | -1.432364 | 1.432364 | Down-regulated |
| MCM7 | 5.17E-05 | 3.11E-06 | -5.174528 | 4.20652607 | -1.431515 | 1.431515 | Down-regulated |
| DDX49 | 6.95E-09 | 6.37E-11 | -8.026497 | 14.71487131 | -1.431322 | 1.431322 | Down-regulated |
| SKIV2L | 6.43E-07 | 1.50E-08 | -6.599361 | 9.38623376 | -1.431278 | 1.431278 | Down-regulated |
| CTSS | 1.92E-04 | 1.65E-05 | -4.708178 | 2.59661188 | -1.431027 | 1.431027 | Down-regulated |
| CHST13 | 6.98E-07 | 1.66E-08 | -6.572378 | 9.28595888 | -1.430696 | 1.430696 | Down-regulated |
| MRPL40 | 1.84E-04 | 1.57E-05 | -4.722469 | 2.64503309 | -1.430579 | 1.430579 | Down-regulated |
| SUCLA2 | 1.34E-03 | 2.08E-04 | -3.964791 | 0.1762823 | -1.43049 | 1.43049 | Down-regulated |
| PSMC2 | 1.86E-07 | 3.38E-09 | -6.988765 | 10.83760233 | -1.430393 | 1.430393 | Down-regulated |
| RAB8A | 1.73E-03 | 2.95E-04 | -3.857731 | -0.15405756 | -1.430126 | 1.430126 | Down-regulated |
| FAM136A | 9.18E-08 | 1.45E-09 | -7.20994 | 11.66423014 | -1.429858 | 1.429858 | Down-regulated |
| NLRC4 | 3.29E-06 | 1.07E-07 | -6.081708 | 7.47288002 | -1.429856 | 1.429856 | Down-regulated |
| SLC30A7 | 1.42E-05 | 6.31E-07 | -5.607797 | 5.74862991 | -1.429688 | 1.429688 | Down-regulated |
| RPS27 | 2.87E-04 | 2.74E-05 | -4.56393 | 2.11146921 | -1.429503 | 1.429503 | Down-regulated |
| FAM127A | 1.22E-04 | 9.22E-06 | -4.872425 | 3.15680064 | -1.429174 | 1.429174 | Down-regulated |
| MFSD3 | 5.40E-04 | 6.21E-05 | -4.326188 | 1.32721801 | -1.428842 | 1.428842 | Down-regulated |
| TPCN2 | 1.77E-07 | 3.20E-09 | -7.003298 | 10.89188398 | -1.428537 | 1.428537 | Down-regulated |
| ZNF784 | 1.78E-07 | 3.23E-09 | -7.00117 | 10.88393562 | -1.428478 | 1.428478 | Down-regulated |
| C3orf14 | 7.84E-04 | 1.02E-04 | -4.180874 | 0.85810484 | -1.428308 | 1.428308 | Down-regulated |
| NAPG | 1.84E-05 | 8.72E-07 | -5.520797 | 5.43598012 | -1.428037 | 1.428037 | Down-regulated |
| TSPAN17 | 2.12E-06 | 6.38E-08 | -6.218196 | 7.97492576 | -1.427316 | 1.427316 | Down-regulated |
| RPS19BP1 | 9.49E-05 | 6.67E-06 | -4.962963 | 3.46888947 | -1.427181 | 1.427181 | Down-regulated |
| PECR | 3.88E-05 | 2.18E-06 | -5.272098 | 4.55035198 | -1.425987 | 1.425987 | Down-regulated |
| ATP2B4 | 5.01E-06 | 1.77E-07 | -5.947889 | 6.98277915 | -1.425335 | 1.425335 | Down-regulated |
| CD27-AS1 | 1.29E-07 | 2.18E-09 | -7.103882 | 11.26772325 | -1.42376 | 1.42376 | Down-regulated |
| RIT1 | 6.56E-10 | 3.41E-12 | -8.797991 | 17.57251151 | -1.423736 | 1.423736 | Down-regulated |
| CACNA1E | 1.31E-04 | 1.00E-05 | -4.848653 | 3.07523458 | -1.423601 | 1.423601 | Down-regulated |
| MS4A6A | 1.02E-08 | 1.03E-10 | -7.900365 | 14.24466248 | -1.423272 | 1.423272 | Down-regulated |
| SOD1 | 7.76E-04 | 1.00E-04 | -4.185076 | 0.87155295 | -1.423168 | 1.423168 | Down-regulated |
| CALML4 | 1.07E-06 | 2.83E-08 | -6.432883 | 8.76837071 | -1.422719 | 1.422719 | Down-regulated |
| ATF2 | 4.97E-08 | 6.99E-10 | -7.400397 | 12.37652465 | -1.42264 | 1.42264 | Down-regulated |
| ELANE | 1.01E-06 | 2.66E-08 | -6.449022 | 8.82817626 | -1.422239 | 1.422239 | Down-regulated |
| HIST2H4A | 3.49E-09 | 2.71E-11 | -8.251227 | 15.55090579 | -1.421883 | 1.421883 | Down-regulated |
| LOC729603 | 1.71E-09 | 1.13E-11 | -8.482287 | 16.40764353 | -1.421464 | 1.421464 | Down-regulated |
| SV2A | 5.44E-06 | 1.96E-07 | -5.921089 | 6.88490532 | -1.421446 | 1.421446 | Down-regulated |
| KDM3A | 3.06E-05 | 1.65E-06 | -5.348389 | 4.82067201 | -1.421323 | 1.421323 | Down-regulated |
| PLD1 | 1.69E-04 | 1.41E-05 | -4.753743 | 2.7512129 | -1.421264 | 1.421264 | Down-regulated |
| PROK1 | 6.53E-09 | 5.90E-11 | -8.04652 | 14.78945621 | -1.421241 | 1.421241 | Down-regulated |
| EBPL | 1.31E-06 | 3.60E-08 | -6.368899 | 8.53146422 | -1.420339 | 1.420339 | Down-regulated |
| PNP | 1.46E-03 | 2.32E-04 | -3.930985 | 0.07141963 | -1.420207 | 1.420207 | Down-regulated |
| TTPAL | 5.02E-04 | 5.62E-05 | -4.355567 | 1.42303796 | -1.419996 | 1.419996 | Down-regulated |
| AP5B1 | 4.60E-03 | 1.32E-03 | -3.378621 | -1.56463447 | -1.419872 | 1.419872 | Down-regulated |
| RBCK1 | 5.48E-07 | 1.23E-08 | -6.651138 | 9.578778 | -1.419812 | 1.419812 | Down-regulated |
| ITFG1 | 1.15E-05 | 4.88E-07 | -5.6771 | 5.99863623 | -1.419704 | 1.419704 | Down-regulated |
| TMEM42 | 3.00E-07 | 6.09E-09 | -6.834972 | 10.26358533 | -1.419352 | 1.419352 | Down-regulated |
| ZNF12 | 1.87E-07 | 3.41E-09 | -6.986421 | 10.8288454 | -1.41904 | 1.41904 | Down-regulated |
| IL15 | 1.89E-06 | 5.54E-08 | -6.255682 | 8.11315502 | -1.419019 | 1.419019 | Down-regulated |
| OAT | 3.18E-03 | 7.27E-04 | -3.573577 | -1.00470533 | -1.418243 | 1.418243 | Down-regulated |
| GMDS | 3.46E-04 | 3.50E-05 | -4.492863 | 1.87496586 | -1.418216 | 1.418216 | Down-regulated |
| USP37 | 1.49E-05 | 6.75E-07 | -5.589847 | 5.68401042 | -1.418121 | 1.418121 | Down-regulated |
| DIRC2 | 1.17E-03 | 1.74E-04 | -4.019681 | 0.3476153 | -1.418044 | 1.418044 | Down-regulated |
| ZRANB2 | 1.15E-06 | 3.06E-08 | -6.4117 | 8.68990197 | -1.417968 | 1.417968 | Down-regulated |
| SNAR-A1 | 1.63E-04 | 1.33E-05 | -4.768422 | 2.80115472 | -1.417767 | 1.417767 | Down-regulated |
| SRSF1 | 2.95E-08 | 3.75E-10 | -7.562539 | 12.9828598 | -1.417576 | 1.417576 | Down-regulated |
| IDS | 1.74E-04 | 1.46E-05 | -4.743005 | 2.71472458 | -1.417128 | 1.417128 | Down-regulated |
| ZNF160 | 2.82E-04 | 2.67E-05 | -4.571386 | 2.1363806 | -1.416652 | 1.416652 | Down-regulated |
| VCL | 2.73E-03 | 5.76E-04 | -3.648046 | -0.78559931 | -1.416603 | 1.416603 | Down-regulated |
| NRM | 4.02E-04 | 4.24E-05 | -4.4373 | 1.69126407 | -1.416333 | 1.416333 | Down-regulated |
| TRIM39 | 2.69E-06 | 8.37E-08 | -6.14656 | 7.71116958 | -1.41605 | 1.41605 | Down-regulated |
| TARBP2 | 3.82E-04 | 3.98E-05 | -4.455876 | 1.75256132 | -1.416014 | 1.416014 | Down-regulated |
| CMC1 | 4.34E-05 | 2.49E-06 | -5.234789 | 4.41862107 | -1.416007 | 1.416007 | Down-regulated |
| ACAT1 | 1.71E-05 | 7.98E-07 | -5.54453 | 5.52113157 | -1.415573 | 1.415573 | Down-regulated |
| CASP2 | 4.81E-08 | 6.67E-10 | -7.412656 | 12.42237193 | -1.415508 | 1.415508 | Down-regulated |
| DGCR6L | 3.76E-04 | 3.91E-05 | -4.461093 | 1.76979777 | -1.415365 | 1.415365 | Down-regulated |
| MRPL54 | 1.20E-02 | 6.92E-03 | -2.8026 | -3.09112818 | -1.414851 | 1.414851 | Down-regulated |
| AIDA | 1.72E-08 | 1.92E-10 | -7.737573 | 13.63697883 | -1.414431 | 1.414431 | Down-regulated |
| C5orf24 | 4.99E-05 | 2.97E-06 | -5.187225 | 4.25114601 | -1.414274 | 1.414274 | Down-regulated |
| CCS | 1.49E-12 | 1.66E-15 | -10.87701 | 25.00413688 | -1.413774 | 1.413774 | Down-regulated |
| EPB41L5 | 8.26E-04 | 1.09E-04 | -4.160572 | 0.79322173 | -1.413601 | 1.413601 | Down-regulated |
| HIST1H2AM | 7.97E-03 | 3.42E-03 | -3.05516 | -2.44673758 | -1.412457 | 1.412457 | Down-regulated |
| TPMT | 5.48E-07 | 1.23E-08 | -6.650763 | 9.57738069 | -1.412296 | 1.412296 | Down-regulated |
| CDAN1 | 2.92E-08 | 3.71E-10 | -7.565944 | 12.99558829 | -1.412127 | 1.412127 | Down-regulated |
| CHTOP | 1.93E-06 | 5.68E-08 | -6.249179 | 8.08916671 | -1.411991 | 1.411991 | Down-regulated |
| RPL41 | 6.83E-05 | 4.41E-06 | -5.077932 | 3.86834275 | -1.411712 | 1.411712 | Down-regulated |
| FAM220A | 3.49E-04 | 3.55E-05 | -4.489306 | 1.86317441 | -1.411162 | 1.411162 | Down-regulated |
| FRAT2 | 2.19E-06 | 6.65E-08 | -6.207284 | 7.93471277 | -1.410989 | 1.410989 | Down-regulated |
| C14orf93 | 1.79E-04 | 1.51E-05 | -4.732558 | 2.67925468 | -1.410769 | 1.410769 | Down-regulated |
| APOBEC3F | 6.95E-07 | 1.65E-08 | -6.574192 | 9.2926955 | -1.410633 | 1.410633 | Down-regulated |
| POLR2H | 2.93E-03 | 6.40E-04 | -3.614082 | -0.88587812 | -1.410471 | 1.410471 | Down-regulated |
| UCHL3 | 1.07E-04 | 7.78E-06 | -4.91993 | 3.32027282 | -1.410328 | 1.410328 | Down-regulated |
| MAX | 3.31E-04 | 3.32E-05 | -4.508591 | 1.92715984 | -1.409909 | 1.409909 | Down-regulated |
| UBQLN1 | 4.34E-04 | 4.69E-05 | -4.408472 | 1.59638511 | -1.409642 | 1.409642 | Down-regulated |
| ZDHHC19 | 6.63E-06 | 2.49E-07 | -5.857152 | 6.65180088 | -1.409158 | 1.409158 | Down-regulated |
| COX19 | 5.66E-07 | 1.28E-08 | -6.640959 | 9.54091082 | -1.408587 | 1.408587 | Down-regulated |
| TAOK3 | 2.13E-04 | 1.88E-05 | -4.670863 | 2.47047759 | -1.408578 | 1.408578 | Down-regulated |
| CNOT1 | 3.75E-06 | 1.26E-07 | -6.038152 | 7.31311402 | -1.408249 | 1.408249 | Down-regulated |
| LPP | 3.39E-05 | 1.84E-06 | -5.317296 | 4.71034764 | -1.408054 | 1.408054 | Down-regulated |
| RPUSD1 | 3.13E-04 | 3.07E-05 | -4.530433 | 1.99978112 | -1.40767 | 1.40767 | Down-regulated |
| TOMM7 | 5.25E-06 | 1.88E-07 | -5.932258 | 6.92568317 | -1.407661 | 1.407661 | Down-regulated |
| IMP4 | 8.80E-04 | 1.18E-04 | -4.136143 | 0.71537203 | -1.407562 | 1.407562 | Down-regulated |
| PCF11 | 3.66E-06 | 1.22E-07 | -6.046384 | 7.34329375 | -1.407338 | 1.407338 | Down-regulated |
| LINC00921 | 1.71E-07 | 3.07E-09 | -7.013981 | 10.93178969 | -1.407158 | 1.407158 | Down-regulated |
| TLR2 | 2.29E-04 | 2.07E-05 | -4.643236 | 2.37737133 | -1.407018 | 1.407018 | Down-regulated |
| SERPINB6 | 6.11E-05 | 3.83E-06 | -5.116951 | 4.00467473 | -1.406672 | 1.406672 | Down-regulated |
| HOXC6 | 1.43E-05 | 6.41E-07 | -5.603596 | 5.73350256 | -1.406594 | 1.406594 | Down-regulated |
| CNOT4 | 3.69E-08 | 4.91E-10 | -7.492695 | 12.72170207 | -1.406507 | 1.406507 | Down-regulated |
| FBXL19-AS1 | 4.42E-03 | 1.23E-03 | -3.40149 | -1.50000976 | -1.40648 | 1.40648 | Down-regulated |
| SCYL1 | 6.17E-06 | 2.27E-07 | -5.881348 | 6.73994727 | -1.406349 | 1.406349 | Down-regulated |
| IGFBP7 | 3.75E-06 | 1.26E-07 | -6.037413 | 7.31040696 | -1.405668 | 1.405668 | Down-regulated |
| ADNP2 | 1.65E-03 | 2.76E-04 | -3.877716 | -0.09278368 | -1.405232 | 1.405232 | Down-regulated |
| ROPN1L | 1.15E-04 | 8.54E-06 | -4.894098 | 3.23130258 | -1.404924 | 1.404924 | Down-regulated |
| ANKRD13D | 4.96E-04 | 5.54E-05 | -4.359544 | 1.4360319 | -1.404741 | 1.404741 | Down-regulated |
| GLRX2 | 7.65E-09 | 7.29E-11 | -7.991217 | 14.58341323 | -1.404333 | 1.404333 | Down-regulated |
| TMSB15B | 2.77E-04 | 2.61E-05 | -4.577665 | 2.15737514 | -1.404184 | 1.404184 | Down-regulated |
| P4HTM | 3.41E-08 | 4.50E-10 | -7.515419 | 12.80667713 | -1.403426 | 1.403426 | Down-regulated |
| TCN2 | 8.44E-07 | 2.11E-08 | -6.509635 | 9.0529776 | -1.402758 | 1.402758 | Down-regulated |
| TMEM186 | 1.52E-04 | 1.22E-05 | -4.793529 | 2.88672135 | -1.402673 | 1.402673 | Down-regulated |
| LOC100288893 | 3.74E-04 | 3.88E-05 | -4.463508 | 1.77777732 | -1.402275 | 1.402275 | Down-regulated |
| GIGYF1 | 3.05E-05 | 1.63E-06 | -5.350515 | 4.8282219 | -1.401501 | 1.401501 | Down-regulated |
| NDN | 4.50E-12 | 6.20E-15 | -10.50848 | 23.7225896 | -1.400873 | 1.400873 | Down-regulated |
| BLOC1S6 | 1.74E-03 | 2.98E-04 | -3.854362 | -0.16436769 | -1.400716 | 1.400716 | Down-regulated |
| PRCC | 1.52E-04 | 1.22E-05 | -4.794134 | 2.88878401 | -1.400538 | 1.400538 | Down-regulated |
| ARHGAP27 | 5.46E-09 | 4.78E-11 | -8.101993 | 14.99599668 | -1.400424 | 1.400424 | Down-regulated |
| FAM118B | 3.85E-04 | 4.03E-05 | -4.452546 | 1.74156182 | -1.400265 | 1.400265 | Down-regulated |
| SPHK2 | 7.92E-07 | 1.95E-08 | -6.529871 | 9.1280895 | -1.400077 | 1.400077 | Down-regulated |
| POLA2 | 1.80E-06 | 5.25E-08 | -6.269595 | 8.16449766 | -1.400072 | 1.400072 | Down-regulated |
| ALKBH1 | 8.61E-03 | 3.92E-03 | -3.007722 | -2.57085884 | -1.399825 | 1.399825 | Down-regulated |
| KDM5B | 1.17E-03 | 1.74E-04 | -4.01928 | 0.34635795 | -1.399736 | 1.399736 | Down-regulated |
| SNORD14A | 8.07E-04 | 1.06E-04 | -4.169388 | 0.82137485 | -1.399621 | 1.399621 | Down-regulated |
| PUS3 | 2.17E-07 | 4.06E-09 | -6.94121 | 10.66001927 | -1.399584 | 1.399584 | Down-regulated |
| C2orf76 | 1.80E-05 | 8.51E-07 | -5.527207 | 5.45896557 | -1.399372 | 1.399372 | Down-regulated |
| CDKN1A | 1.44E-04 | 1.13E-05 | -4.81432 | 2.95771795 | -1.398956 | 1.398956 | Down-regulated |
| RBM10 | 1.09E-04 | 7.99E-06 | -4.912474 | 3.29457108 | -1.398531 | 1.398531 | Down-regulated |
| DNAJC3 | 6.60E-06 | 2.47E-07 | -5.858832 | 6.65791905 | -1.398304 | 1.398304 | Down-regulated |
| YIF1A | 1.40E-05 | 6.23E-07 | -5.611281 | 5.76117617 | -1.397675 | 1.397675 | Down-regulated |
| NRADDP | 6.34E-05 | 4.01E-06 | -5.104284 | 3.96037522 | -1.39692 | 1.39692 | Down-regulated |
| TOMM70 | 3.43E-05 | 1.87E-06 | -5.312839 | 4.69455138 | -1.396556 | 1.396556 | Down-regulated |
| ZNF407 | 4.59E-09 | 3.77E-11 | -8.164107 | 15.22709774 | -1.395937 | 1.395937 | Down-regulated |
| ISCA2 | 2.17E-04 | 1.93E-05 | -4.663726 | 2.44640231 | -1.395722 | 1.395722 | Down-regulated |
| NIPSNAP3A | 2.61E-07 | 5.12E-09 | -6.880601 | 10.43380007 | -1.395576 | 1.395576 | Down-regulated |
| BAG4 | 3.67E-04 | 3.79E-05 | -4.469791 | 1.79855588 | -1.395406 | 1.395406 | Down-regulated |
| MAN1A2 | 3.26E-07 | 6.65E-09 | -6.812161 | 10.1785247 | -1.395334 | 1.395334 | Down-regulated |
| IL10 | 1.67E-05 | 7.77E-07 | -5.551993 | 5.54792981 | -1.395063 | 1.395063 | Down-regulated |
| CD300LF | 2.82E-09 | 2.12E-11 | -8.315028 | 15.78778462 | -1.394345 | 1.394345 | Down-regulated |
| LEMD2 | 2.10E-05 | 1.03E-06 | -5.475896 | 5.27516735 | -1.393905 | 1.393905 | Down-regulated |
| HSPA7 | 9.15E-05 | 6.36E-06 | -4.976287 | 3.51500756 | -1.393654 | 1.393654 | Down-regulated |
| PSME4 | 1.99E-06 | 5.91E-08 | -6.238479 | 8.04970253 | -1.39348 | 1.39348 | Down-regulated |
| MT1E | 5.96E-08 | 8.68E-10 | -7.343835 | 12.1649749 | -1.393015 | 1.393015 | Down-regulated |
| MIR503HG | 4.69E-04 | 5.17E-05 | -4.38001 | 1.50299799 | -1.392317 | 1.392317 | Down-regulated |
| TNK2 | 1.17E-06 | 3.15E-08 | -6.403944 | 8.66117959 | -1.392222 | 1.392222 | Down-regulated |
| FAM49B | 1.98E-03 | 3.58E-04 | -3.797522 | -0.3375392 | -1.392133 | 1.392133 | Down-regulated |
| ALDH8A1 | 5.34E-05 | 3.25E-06 | -5.16225 | 4.16341656 | -1.391459 | 1.391459 | Down-regulated |
| DCUN1D4 | 2.92E-08 | 3.71E-10 | -7.565557 | 12.99414237 | -1.390886 | 1.390886 | Down-regulated |
| MFAP3 | 1.26E-07 | 2.13E-09 | -7.109221 | 11.28768107 | -1.388966 | 1.388966 | Down-regulated |
| RNF135 | 3.58E-03 | 8.73E-04 | -3.51444 | -1.17667716 | -1.388596 | 1.388596 | Down-regulated |
| NTN3 | 1.55E-04 | 1.25E-05 | -4.787143 | 2.86494018 | -1.388579 | 1.388579 | Down-regulated |
| PSMG4 | 1.11E-03 | 1.61E-04 | -4.042929 | 0.42056908 | -1.388253 | 1.388253 | Down-regulated |
| DUT | 1.68E-04 | 1.39E-05 | -4.757644 | 2.76447908 | -1.387903 | 1.387903 | Down-regulated |
| SLC37A4 | 1.21E-03 | 1.82E-04 | -4.00595 | 0.30463301 | -1.387844 | 1.387844 | Down-regulated |
| TFIP11 | 3.23E-08 | 4.20E-10 | -7.533521 | 12.87436236 | -1.387029 | 1.387029 | Down-regulated |
| SLMAP | 5.35E-10 | 2.57E-12 | -8.873696 | 17.85073493 | -1.386996 | 1.386996 | Down-regulated |
| MRPS17 | 1.00E-04 | 7.17E-06 | -4.942821 | 3.39926344 | -1.386708 | 1.386708 | Down-regulated |
| ZNRD1 | 2.53E-05 | 1.30E-06 | -5.412653 | 5.04932741 | -1.38627 | 1.38627 | Down-regulated |
| UPB1 | 1.13E-08 | 1.15E-10 | -7.871733 | 14.13784359 | -1.385449 | 1.385449 | Down-regulated |
| MRPL10 | 8.26E-05 | 5.56E-06 | -5.013701 | 3.64475275 | -1.385283 | 1.385283 | Down-regulated |
| FBXO38 | 5.12E-08 | 7.24E-10 | -7.391141 | 12.34190681 | -1.385263 | 1.385263 | Down-regulated |
| HOOK2 | 1.17E-03 | 1.73E-04 | -4.020082 | 0.34886951 | -1.384848 | 1.384848 | Down-regulated |
| AKR7A2 | 6.72E-08 | 1.01E-09 | -7.304256 | 12.01694613 | -1.383003 | 1.383003 | Down-regulated |
| TPT1 | 1.50E-03 | 2.42E-04 | -3.918422 | 0.03257859 | -1.382778 | 1.382778 | Down-regulated |
| TNFRSF10B | 2.58E-03 | 5.26E-04 | -3.676457 | -0.70127453 | -1.382223 | 1.382223 | Down-regulated |
| HLA-E | 2.39E-03 | 4.73E-04 | -3.710167 | -0.60070668 | -1.38199 | 1.38199 | Down-regulated |
| LZTR1 | 4.77E-04 | 5.28E-05 | -4.373718 | 1.48239568 | -1.38197 | 1.38197 | Down-regulated |
| ZFYVE16 | 5.21E-03 | 1.62E-03 | -3.311591 | -1.75238453 | -1.381926 | 1.381926 | Down-regulated |
| MBD6 | 4.68E-04 | 5.16E-05 | -4.38057 | 1.50483485 | -1.381858 | 1.381858 | Down-regulated |
| FYTTD1 | 4.42E-07 | 9.49E-09 | -6.719141 | 9.83189749 | -1.381735 | 1.381735 | Down-regulated |
| PIGX | 3.27E-08 | 4.29E-10 | -7.527959 | 12.85356618 | -1.381368 | 1.381368 | Down-regulated |
| FAM200B | 3.29E-10 | 1.43E-12 | -9.029142 | 18.420532 | -1.381153 | 1.381153 | Down-regulated |
| SLC22A18 | 5.97E-07 | 1.36E-08 | -6.624776 | 9.48072444 | -1.380996 | 1.380996 | Down-regulated |
| PCCB | 1.11E-03 | 1.61E-04 | -4.043218 | 0.42147728 | -1.380454 | 1.380454 | Down-regulated |
| UHMK1 | 1.85E-05 | 8.77E-07 | -5.519267 | 5.43049168 | -1.380334 | 1.380334 | Down-regulated |
| BLZF1 | 4.36E-07 | 9.32E-09 | -6.723872 | 9.84951609 | -1.379872 | 1.379872 | Down-regulated |
| PIGF | 2.47E-05 | 1.26E-06 | -5.420594 | 5.07764232 | -1.379828 | 1.379828 | Down-regulated |
| VPS13B | 1.09E-04 | 7.95E-06 | -4.91399 | 3.29979761 | -1.379419 | 1.379419 | Down-regulated |
| ZNF821 | 6.32E-04 | 7.65E-05 | -4.264772 | 1.12795797 | -1.379325 | 1.379325 | Down-regulated |
| RER1 | 2.93E-07 | 5.92E-09 | -6.842665 | 10.29227936 | -1.379125 | 1.379125 | Down-regulated |
| CSNK1A1 | 6.03E-08 | 8.84E-10 | -7.338975 | 12.14679858 | -1.379067 | 1.379067 | Down-regulated |
| C20orf196 | 8.95E-06 | 3.62E-07 | -5.75741 | 6.28934912 | -1.378714 | 1.378714 | Down-regulated |
| C9orf66 | 6.11E-04 | 7.33E-05 | -4.277451 | 1.16897644 | -1.378683 | 1.378683 | Down-regulated |
| BARD1 | 2.39E-03 | 4.71E-04 | -3.71129 | -0.59734448 | -1.378586 | 1.378586 | Down-regulated |
| C19orf54 | 4.57E-09 | 3.73E-11 | -8.166822 | 15.23719347 | -1.378427 | 1.378427 | Down-regulated |
| KIAA2013 | 1.01E-06 | 2.64E-08 | -6.451127 | 8.83598011 | -1.378381 | 1.378381 | Down-regulated |
| POR | 4.82E-05 | 2.84E-06 | -5.199021 | 4.29263175 | -1.37823 | 1.37823 | Down-regulated |
| FUT8-AS1 | 3.06E-04 | 2.98E-05 | -4.538941 | 2.02811367 | -1.37811 | 1.37811 | Down-regulated |
| TMED3 | 6.75E-06 | 2.54E-07 | -5.851618 | 6.63165307 | -1.377635 | 1.377635 | Down-regulated |
| RPS17 | 1.12E-04 | 8.27E-06 | -4.902866 | 3.26148063 | -1.377284 | 1.377284 | Down-regulated |
| CHD1 | 4.85E-04 | 5.39E-05 | -4.367669 | 1.46259979 | -1.377201 | 1.377201 | Down-regulated |
| RNF175 | 4.35E-06 | 1.50E-07 | -5.992135 | 7.1445751 | -1.377172 | 1.377172 | Down-regulated |
| RGL1 | 2.24E-04 | 2.01E-05 | -4.652881 | 2.40984969 | -1.376275 | 1.376275 | Down-regulated |
| FRS3 | 2.73E-05 | 1.43E-06 | -5.387493 | 4.95970577 | -1.37584 | 1.37584 | Down-regulated |
| CEACAM8 | 1.12E-03 | 1.62E-04 | -4.040699 | 0.41356194 | -1.375521 | 1.375521 | Down-regulated |
| PUS7L | 3.24E-05 | 1.76E-06 | -5.330737 | 4.75801172 | -1.375066 | 1.375066 | Down-regulated |
| SEPT5 | 7.65E-09 | 7.26E-11 | -7.992416 | 14.58788093 | -1.374902 | 1.374902 | Down-regulated |
| LGALS8 | 1.02E-07 | 1.65E-09 | -7.175803 | 11.53658782 | -1.374325 | 1.374325 | Down-regulated |
| CD58 | 4.19E-05 | 2.40E-06 | -5.245377 | 4.45597527 | -1.373421 | 1.373421 | Down-regulated |
| NMD3 | 5.03E-05 | 2.99E-06 | -5.184708 | 4.24229467 | -1.373305 | 1.373305 | Down-regulated |
| SRGAP2 | 2.47E-05 | 1.26E-06 | -5.421465 | 5.08074719 | -1.373284 | 1.373284 | Down-regulated |
| VPS29 | 2.32E-06 | 7.12E-08 | -6.189484 | 7.86914619 | -1.372437 | 1.372437 | Down-regulated |
| RTCA | 1.42E-05 | 6.32E-07 | -5.607399 | 5.74719461 | -1.372304 | 1.372304 | Down-regulated |
| EVA1B | 3.25E-04 | 3.23E-05 | -4.516539 | 1.95356721 | -1.372156 | 1.372156 | Down-regulated |
| IL16 | 3.33E-07 | 6.83E-09 | -6.804939 | 10.15159589 | -1.372104 | 1.372104 | Down-regulated |
| PRPS2 | 9.56E-08 | 1.52E-09 | -7.197542 | 11.61787264 | -1.371582 | 1.371582 | Down-regulated |
| MRPL13 | 2.29E-04 | 2.07E-05 | -4.644267 | 2.38084361 | -1.371324 | 1.371324 | Down-regulated |
| PRPSAP1 | 1.14E-03 | 1.66E-04 | -4.032629 | 0.38821956 | -1.371319 | 1.371319 | Down-regulated |
| KIF14 | 1.25E-03 | 1.90E-04 | -3.991915 | 0.26078294 | -1.371157 | 1.371157 | Down-regulated |
| LTB4R | 9.39E-05 | 6.57E-06 | -4.967094 | 3.48318313 | -1.370738 | 1.370738 | Down-regulated |
| GNA13 | 1.35E-05 | 5.96E-07 | -5.623167 | 5.80400444 | -1.370708 | 1.370708 | Down-regulated |
| RBBP8 | 3.99E-06 | 1.36E-07 | -6.018619 | 7.24154405 | -1.370409 | 1.370409 | Down-regulated |
| SP3 | 7.91E-05 | 5.27E-06 | -5.028579 | 3.69644939 | -1.37027 | 1.37027 | Down-regulated |
| CEP85 | 1.59E-05 | 7.26E-07 | -5.570352 | 5.61389523 | -1.370164 | 1.370164 | Down-regulated |
| ZKSCAN4 | 2.47E-06 | 7.63E-08 | -6.170984 | 7.80103464 | -1.369935 | 1.369935 | Down-regulated |
| UBA6 | 3.35E-06 | 1.10E-07 | -6.07425 | 7.44550722 | -1.36964 | 1.36964 | Down-regulated |
| TRIM41 | 4.99E-06 | 1.76E-07 | -5.94939 | 6.98826381 | -1.369566 | 1.369566 | Down-regulated |
| FIZ1 | 6.50E-04 | 7.93E-05 | -4.25403 | 1.09325237 | -1.369385 | 1.369385 | Down-regulated |
| VNN3 | 2.36E-04 | 2.15E-05 | -4.633565 | 2.34483875 | -1.369039 | 1.369039 | Down-regulated |
| PLPP4 | 1.00E-04 | 7.17E-06 | -4.942797 | 3.39918109 | -1.368632 | 1.368632 | Down-regulated |
| GALNT1 | 2.26E-06 | 6.91E-08 | -6.197338 | 7.89807087 | -1.368252 | 1.368252 | Down-regulated |
| HNRNPCL2 | 6.28E-09 | 5.65E-11 | -8.058056 | 14.83241651 | -1.368193 | 1.368193 | Down-regulated |
| C11orf54 | 1.13E-09 | 6.85E-12 | -8.613519 | 16.89272318 | -1.367733 | 1.367733 | Down-regulated |
| HIST1H3D | 2.87E-05 | 1.51E-06 | -5.371743 | 4.90366912 | -1.367487 | 1.367487 | Down-regulated |
| CRHR1 | 2.22E-09 | 1.61E-11 | -8.388473 | 16.06018239 | -1.367155 | 1.367155 | Down-regulated |
| YY1AP1 | 2.84E-04 | 2.71E-05 | -4.567046 | 2.12187892 | -1.366534 | 1.366534 | Down-regulated |
| LINC00339 | 1.29E-05 | 5.64E-07 | -5.63824 | 5.85834966 | -1.366453 | 1.366453 | Down-regulated |
| ZBTB47 | 5.46E-07 | 1.22E-08 | -6.652672 | 9.58448387 | -1.365892 | 1.365892 | Down-regulated |
| CENPL | 6.10E-03 | 2.12E-03 | -3.219899 | -2.00509689 | -1.365829 | 1.365829 | Down-regulated |
| RMI2 | 1.29E-03 | 1.98E-04 | -3.980434 | 0.22497796 | -1.365732 | 1.365732 | Down-regulated |
| PPP6R2 | 1.32E-06 | 3.64E-08 | -6.366203 | 8.5214889 | -1.365507 | 1.365507 | Down-regulated |
| CTNNA1 | 1.21E-03 | 1.82E-04 | -4.005387 | 0.30287218 | -1.365384 | 1.365384 | Down-regulated |
| IDE | 6.83E-05 | 4.41E-06 | -5.07799 | 3.86854408 | -1.365254 | 1.365254 | Down-regulated |
| HSPE1 | 2.43E-04 | 2.23E-05 | -4.622662 | 2.30819377 | -1.365039 | 1.365039 | Down-regulated |
| SESTD1 | 6.40E-07 | 1.49E-08 | -6.60063 | 9.39095017 | -1.364774 | 1.364774 | Down-regulated |
| LRRFIP2 | 1.70E-03 | 2.88E-04 | -3.864709 | -0.13268176 | -1.364378 | 1.364378 | Down-regulated |
| LBR | 5.90E-03 | 2.00E-03 | -3.239301 | -1.95202647 | -1.363986 | 1.363986 | Down-regulated |
| CRACR2B | 1.60E-04 | 1.30E-05 | -4.774774 | 2.8227848 | -1.36397 | 1.36397 | Down-regulated |
| PHF5A | 7.44E-05 | 4.91E-06 | -5.048031 | 3.76412633 | -1.363967 | 1.363967 | Down-regulated |
| PTAR1 | 1.12E-04 | 8.25E-06 | -4.903622 | 3.26408297 | -1.363401 | 1.363401 | Down-regulated |
| ZNF772 | 1.60E-07 | 2.81E-09 | -7.036756 | 11.01687534 | -1.363362 | 1.363362 | Down-regulated |
| UBR5 | 7.02E-07 | 1.67E-08 | -6.57024 | 9.2780141 | -1.363296 | 1.363296 | Down-regulated |
| ITPRIPL2 | 8.75E-04 | 1.17E-04 | -4.138731 | 0.72360687 | -1.363214 | 1.363214 | Down-regulated |
| MYNN | 1.95E-05 | 9.44E-07 | -5.499251 | 5.3587657 | -1.362984 | 1.362984 | Down-regulated |
| ZNF2 | 1.96E-04 | 1.70E-05 | -4.700635 | 2.57108054 | -1.362559 | 1.362559 | Down-regulated |
| GPR141 | 2.15E-05 | 1.06E-06 | -5.468973 | 5.25040645 | -1.362456 | 1.362456 | Down-regulated |
| NOL8 | 5.00E-05 | 2.97E-06 | -5.186667 | 4.2491823 | -1.362158 | 1.362158 | Down-regulated |
| GABPB2 | 4.21E-03 | 1.14E-03 | -3.426803 | -1.42814472 | -1.362019 | 1.362019 | Down-regulated |
| DUSP3 | 1.75E-04 | 1.47E-05 | -4.740376 | 2.7057932 | -1.361571 | 1.361571 | Down-regulated |
| ZRSR2 | 1.70E-04 | 1.42E-05 | -4.751682 | 2.74420884 | -1.36108 | 1.36108 | Down-regulated |
| IFNAR2 | 6.18E-08 | 9.15E-10 | -7.330067 | 12.11348333 | -1.360914 | 1.360914 | Down-regulated |
| GTF2IRD2B | 1.18E-03 | 1.76E-04 | -4.016261 | 0.33690006 | -1.360881 | 1.360881 | Down-regulated |
| DHRS1 | 3.93E-03 | 1.02E-03 | -3.465221 | -1.31841121 | -1.36066 | 1.36066 | Down-regulated |
| ERP29 | 6.05E-09 | 5.35E-11 | -8.072218 | 14.88515359 | -1.360625 | 1.360625 | Down-regulated |
| GLRX | 9.05E-05 | 6.27E-06 | -4.980172 | 3.52846139 | -1.360351 | 1.360351 | Down-regulated |
| ATRIP | 3.04E-04 | 2.95E-05 | -4.542002 | 2.03831432 | -1.360205 | 1.360205 | Down-regulated |
| METTL22 | 2.65E-03 | 5.51E-04 | -3.662109 | -0.74391129 | -1.359759 | 1.359759 | Down-regulated |
| NPM3 | 1.44E-07 | 2.48E-09 | -7.070195 | 11.14182335 | -1.359512 | 1.359512 | Down-regulated |
| NEDD4 | 2.66E-04 | 2.48E-05 | -4.591915 | 2.20506395 | -1.359475 | 1.359475 | Down-regulated |
| BST2 | 5.83E-06 | 2.13E-07 | -5.899318 | 6.80546594 | -1.359372 | 1.359372 | Down-regulated |
| ICAM1 | 5.55E-06 | 2.00E-07 | -5.915101 | 6.86305021 | -1.359157 | 1.359157 | Down-regulated |
| KYNU | 1.53E-05 | 6.96E-07 | -5.581472 | 5.65387977 | -1.358257 | 1.358257 | Down-regulated |
| MDH2 | 2.70E-04 | 2.53E-05 | -4.585933 | 2.18503528 | -1.358232 | 1.358232 | Down-regulated |
| HIRIP3 | 2.62E-07 | 5.16E-09 | -6.87848 | 10.42588882 | -1.357623 | 1.357623 | Down-regulated |
| RAE1 | 6.04E-09 | 5.32E-11 | -8.073995 | 14.89177136 | -1.357608 | 1.357608 | Down-regulated |
| PGM2 | 1.87E-04 | 1.60E-05 | -4.717328 | 2.62760768 | -1.357284 | 1.357284 | Down-regulated |
| SUMO2 | 1.29E-04 | 9.83E-06 | -4.854631 | 3.0957302 | -1.357262 | 1.357262 | Down-regulated |
| RPL6 | 5.12E-04 | 5.78E-05 | -4.347338 | 1.39616562 | -1.357225 | 1.357225 | Down-regulated |
| SCYL2 | 7.33E-05 | 4.82E-06 | -5.052961 | 3.78129277 | -1.357114 | 1.357114 | Down-regulated |
| ATG4C | 1.88E-05 | 9.00E-07 | -5.512295 | 5.40550127 | -1.357049 | 1.357049 | Down-regulated |
| MAPKAP1 | 1.79E-05 | 8.45E-07 | -5.529322 | 5.46655366 | -1.356903 | 1.356903 | Down-regulated |
| TXNRD2 | 8.63E-06 | 3.45E-07 | -5.769803 | 6.33430461 | -1.356838 | 1.356838 | Down-regulated |
| HIST1H4J | 2.67E-04 | 2.50E-05 | -4.590416 | 2.20004573 | -1.356835 | 1.356835 | Down-regulated |
| LOC153577 | 6.95E-07 | 1.65E-08 | -6.573734 | 9.29099539 | -1.356613 | 1.356613 | Down-regulated |
| SLC25A16 | 2.07E-04 | 1.81E-05 | -4.681591 | 2.50669635 | -1.356593 | 1.356593 | Down-regulated |
| E4F1 | 3.26E-08 | 4.25E-10 | -7.530165 | 12.86181619 | -1.356513 | 1.356513 | Down-regulated |
| PDZD8 | 2.62E-07 | 5.13E-09 | -6.879824 | 10.43090438 | -1.356424 | 1.356424 | Down-regulated |
| RNF114 | 1.32E-03 | 2.03E-04 | -3.971816 | 0.19813864 | -1.356235 | 1.356235 | Down-regulated |
| PSMB5 | 2.20E-03 | 4.17E-04 | -3.750024 | -0.48108639 | -1.355741 | 1.355741 | Down-regulated |
| ACVR1B | 1.65E-05 | 7.61E-07 | -5.557374 | 5.56725628 | -1.355649 | 1.355649 | Down-regulated |
| FUCA2 | 9.04E-05 | 6.26E-06 | -4.980704 | 3.53030527 | -1.355509 | 1.355509 | Down-regulated |
| PSMB10 | 9.73E-06 | 4.01E-07 | -5.729746 | 6.18909266 | -1.355451 | 1.355451 | Down-regulated |
| PARVG | 8.79E-04 | 1.18E-04 | -4.13653 | 0.71660252 | -1.355377 | 1.355377 | Down-regulated |
| GAS6 | 3.00E-07 | 6.08E-09 | -6.835527 | 10.26565523 | -1.355217 | 1.355217 | Down-regulated |
| ZFYVE21 | 1.28E-04 | 9.77E-06 | -4.856363 | 3.10167107 | -1.355008 | 1.355008 | Down-regulated |
| RAB33A | 7.89E-03 | 3.36E-03 | -3.061634 | -2.42969046 | -1.354792 | 1.354792 | Down-regulated |
| TRAPPC5 | 2.47E-05 | 1.26E-06 | -5.421214 | 5.0798532 | -1.354671 | 1.354671 | Down-regulated |
| SEL1L | 1.76E-04 | 1.48E-05 | -4.73849 | 2.69938946 | -1.354487 | 1.354487 | Down-regulated |
| ARL8B | 1.88E-04 | 1.61E-05 | -4.714503 | 2.61803286 | -1.354112 | 1.354112 | Down-regulated |
| SLA | 2.42E-04 | 2.21E-05 | -4.624576 | 2.31462357 | -1.353857 | 1.353857 | Down-regulated |
| CDK13 | 2.81E-06 | 8.83E-08 | -6.132545 | 7.65963357 | -1.35378 | 1.35378 | Down-regulated |
| BIRC2 | 7.06E-05 | 4.59E-06 | -5.06697 | 3.83011006 | -1.353443 | 1.353443 | Down-regulated |
| MAD2L2 | 5.16E-05 | 3.09E-06 | -5.175515 | 4.20999131 | -1.353424 | 1.353424 | Down-regulated |
| CHRNA5 | 1.31E-03 | 2.01E-04 | -3.975285 | 0.20893591 | -1.353406 | 1.353406 | Down-regulated |
| MFSD5 | 7.54E-09 | 7.07E-11 | -7.999128 | 14.61289569 | -1.352948 | 1.352948 | Down-regulated |
| PPP1CC | 9.97E-06 | 4.12E-07 | -5.722222 | 6.16184496 | -1.352769 | 1.352769 | Down-regulated |
| COIL | 7.61E-04 | 9.77E-05 | -4.192288 | 0.89465264 | -1.35191 | 1.35191 | Down-regulated |
| AGTPBP1 | 1.77E-06 | 5.14E-08 | -6.275284 | 8.18549513 | -1.351634 | 1.351634 | Down-regulated |
| TP53I13 | 1.18E-04 | 8.79E-06 | -4.88588 | 3.20303779 | -1.351616 | 1.351616 | Down-regulated |
| CDA | 1.34E-03 | 2.08E-04 | -3.964719 | 0.17605905 | -1.351525 | 1.351525 | Down-regulated |
| ELF2 | 1.18E-04 | 8.78E-06 | -4.88634 | 3.20461692 | -1.351491 | 1.351491 | Down-regulated |
| OSM | 2.80E-04 | 2.65E-05 | -4.572715 | 2.14082169 | -1.350999 | 1.350999 | Down-regulated |
| ST13 | 3.79E-06 | 1.28E-07 | -6.033998 | 7.29789182 | -1.35093 | 1.35093 | Down-regulated |
| NME2 | 1.33E-04 | 1.02E-05 | -4.843249 | 3.05671498 | -1.350751 | 1.350751 | Down-regulated |
| NOTCH2 | 4.94E-04 | 5.50E-05 | -4.361676 | 1.44300132 | -1.350673 | 1.350673 | Down-regulated |
| C12orf10 | 1.62E-08 | 1.77E-10 | -7.759125 | 13.7174766 | -1.350668 | 1.350668 | Down-regulated |
| TMEM184C | 1.61E-05 | 7.35E-07 | -5.56683 | 5.60123597 | -1.350586 | 1.350586 | Down-regulated |
| FAM91A1 | 6.11E-06 | 2.24E-07 | -5.884934 | 6.75302158 | -1.350504 | 1.350504 | Down-regulated |
| LIN7A | 6.45E-04 | 7.85E-05 | -4.256984 | 1.10279005 | -1.35013 | 1.35013 | Down-regulated |
| FLAD1 | 4.35E-04 | 4.70E-05 | -4.407319 | 1.59259533 | -1.349313 | 1.349313 | Down-regulated |
| ZNF652 | 1.39E-02 | 8.81E-03 | -2.713152 | -3.30935503 | -1.348837 | 1.348837 | Down-regulated |
| ZBTB34 | 2.66E-05 | 1.38E-06 | -5.396456 | 4.99161683 | -1.348339 | 1.348339 | Down-regulated |
| USP15 | 2.24E-05 | 1.12E-06 | -5.454181 | 5.19753473 | -1.34804 | 1.34804 | Down-regulated |
| ADAM10 | 2.05E-04 | 1.80E-05 | -4.684064 | 2.5150515 | -1.347919 | 1.347919 | Down-regulated |
| UHRF1BP1L | 1.98E-03 | 3.57E-04 | -3.798198 | -0.33548949 | -1.347804 | 1.347804 | Down-regulated |
| BCKDHA | 7.32E-04 | 9.29E-05 | -4.207169 | 0.94238153 | -1.346802 | 1.346802 | Down-regulated |
| TEN1 | 2.35E-04 | 2.14E-05 | -4.634845 | 2.34914309 | -1.346309 | 1.346309 | Down-regulated |
| GNB4 | 9.04E-08 | 1.42E-09 | -7.214759 | 11.68224893 | -1.346163 | 1.346163 | Down-regulated |
| MILR1 | 1.03E-05 | 4.27E-07 | -5.712922 | 6.1281808 | -1.345592 | 1.345592 | Down-regulated |
| SPOP | 6.71E-07 | 1.57E-08 | -6.586398 | 9.33805323 | -1.345334 | 1.345334 | Down-regulated |
| CD4 | 1.27E-05 | 5.48E-07 | -5.645902 | 5.88599029 | -1.34528 | 1.34528 | Down-regulated |
| UBE3C | 6.39E-04 | 7.76E-05 | -4.260591 | 1.11444331 | -1.344948 | 1.344948 | Down-regulated |
| ECM1 | 1.09E-07 | 1.79E-09 | -7.154258 | 11.45603789 | -1.34485 | 1.34485 | Down-regulated |
| ERLEC1 | 1.72E-02 | 1.21E-02 | -2.592238 | -3.59566123 | -1.344519 | 1.344519 | Down-regulated |
| DDAH2 | 3.07E-05 | 1.65E-06 | -5.347296 | 4.81678783 | -1.344355 | 1.344355 | Down-regulated |
| ZNF627 | 1.74E-04 | 1.46E-05 | -4.74295 | 2.7145351 | -1.344185 | 1.344185 | Down-regulated |
| LOC285147 | 1.92E-04 | 1.65E-05 | -4.707722 | 2.59506717 | -1.344174 | 1.344174 | Down-regulated |
| MRPL55 | 1.10E-08 | 1.13E-10 | -7.877575 | 14.15964083 | -1.343835 | 1.343835 | Down-regulated |
| FBXO28 | 8.69E-05 | 5.93E-06 | -4.995648 | 3.5821002 | -1.343756 | 1.343756 | Down-regulated |
| PRKCD | 5.70E-04 | 6.66E-05 | -4.305369 | 1.25951018 | -1.343211 | 1.343211 | Down-regulated |
| WBP1 | 2.38E-07 | 4.54E-09 | -6.911967 | 10.55085598 | -1.342723 | 1.342723 | Down-regulated |
| ZFC3H1 | 3.51E-06 | 1.16E-07 | -6.059316 | 7.39071651 | -1.342554 | 1.342554 | Down-regulated |
| FEZ2 | 2.13E-03 | 3.96E-04 | -3.765947 | -0.43308178 | -1.342549 | 1.342549 | Down-regulated |
| BRE | 1.99E-06 | 5.93E-08 | -6.23789 | 8.04752913 | -1.342443 | 1.342443 | Down-regulated |
| SNORA5C | 7.24E-03 | 2.88E-03 | -3.115081 | -2.28798088 | -1.341729 | 1.341729 | Down-regulated |
| ZFP36 | 1.04E-07 | 1.70E-09 | -7.168242 | 11.50831835 | -1.341591 | 1.341591 | Down-regulated |
| CREB3L2 | 5.70E-04 | 6.67E-05 | -4.305046 | 1.25846141 | -1.3413 | 1.3413 | Down-regulated |
| TRIQK | 3.39E-05 | 1.85E-06 | -5.316724 | 4.7083202 | -1.341164 | 1.341164 | Down-regulated |
| PLEKHJ1 | 1.65E-04 | 1.36E-05 | -4.763804 | 2.78543794 | -1.341098 | 1.341098 | Down-regulated |
| METTL9 | 1.46E-03 | 2.32E-04 | -3.930975 | 0.07138833 | -1.340839 | 1.340839 | Down-regulated |
| AFMID | 3.02E-08 | 3.87E-10 | -7.554969 | 12.95455543 | -1.340474 | 1.340474 | Down-regulated |
| CERS2 | 6.22E-07 | 1.43E-08 | -6.610979 | 9.42942206 | -1.340437 | 1.340437 | Down-regulated |
| ACVRL1 | 3.37E-04 | 3.39E-05 | -4.501978 | 1.90520312 | -1.34039 | 1.34039 | Down-regulated |
| RHOU | 1.12E-06 | 2.97E-08 | -6.419943 | 8.7204297 | -1.339562 | 1.339562 | Down-regulated |
| DDX59 | 1.32E-03 | 2.04E-04 | -3.97034 | 0.19354351 | -1.339546 | 1.339546 | Down-regulated |
| FAM65B | 1.55E-03 | 2.54E-04 | -3.904165 | -0.01141365 | -1.339378 | 1.339378 | Down-regulated |
| NXT2 | 2.07E-05 | 1.01E-06 | -5.481696 | 5.29591927 | -1.339343 | 1.339343 | Down-regulated |
| OMG | 6.76E-05 | 4.34E-06 | -5.082018 | 3.88260116 | -1.339285 | 1.339285 | Down-regulated |
| CREB5 | 1.50E-06 | 4.24E-08 | -6.326184 | 8.37350382 | -1.338939 | 1.338939 | Down-regulated |
| ZSWIM3 | 9.48E-05 | 6.65E-06 | -4.963875 | 3.47204486 | -1.33854 | 1.33854 | Down-regulated |
| HNRNPCL3 | 2.92E-08 | 3.71E-10 | -7.565693 | 12.99464895 | -1.338124 | 1.338124 | Down-regulated |
| TMED10P1 | 1.55E-03 | 2.55E-04 | -3.902297 | -0.01717078 | -1.338113 | 1.338113 | Down-regulated |
| IKBKG | 2.73E-06 | 8.51E-08 | -6.142322 | 7.6955848 | -1.337909 | 1.337909 | Down-regulated |
| OST4 | 5.41E-04 | 6.22E-05 | -4.325755 | 1.32580619 | -1.337684 | 1.337684 | Down-regulated |
| SRRM1 | 3.75E-06 | 1.26E-07 | -6.038293 | 7.31363292 | -1.33745 | 1.33745 | Down-regulated |
| DCLRE1C | 9.41E-04 | 1.29E-04 | -4.108761 | 0.62839723 | -1.337354 | 1.337354 | Down-regulated |
| SPAG7 | 1.29E-06 | 3.55E-08 | -6.373105 | 8.54702806 | -1.337344 | 1.337344 | Down-regulated |
| CPT1A | 8.12E-07 | 2.01E-08 | -6.521816 | 9.09818616 | -1.337207 | 1.337207 | Down-regulated |
| TP53BP2 | 8.68E-06 | 3.48E-07 | -5.767468 | 6.32583287 | -1.337124 | 1.337124 | Down-regulated |
| ELL2 | 2.74E-03 | 5.79E-04 | -3.646019 | -0.79160036 | -1.337001 | 1.337001 | Down-regulated |
| ADAR | 2.84E-08 | 3.57E-10 | -7.575377 | 13.03085414 | -1.336824 | 1.336824 | Down-regulated |
| NFIL3 | 2.76E-04 | 2.60E-05 | -4.578996 | 2.161825 | -1.336613 | 1.336613 | Down-regulated |
| KYAT1 | 2.07E-06 | 6.20E-08 | -6.225995 | 8.0036723 | -1.336439 | 1.336439 | Down-regulated |
| COG5 | 1.27E-03 | 1.94E-04 | -3.986517 | 0.24394003 | -1.335808 | 1.335808 | Down-regulated |
| AGO3 | 2.17E-07 | 4.07E-09 | -6.940557 | 10.65758099 | -1.334673 | 1.334673 | Down-regulated |
| GSTO2 | 7.84E-08 | 1.21E-09 | -7.257125 | 11.8406823 | -1.334584 | 1.334584 | Down-regulated |
| CAB39L | 3.35E-05 | 1.82E-06 | -5.321052 | 4.72366409 | -1.334162 | 1.334162 | Down-regulated |
| SEC24A | 3.86E-03 | 9.88E-04 | -3.474121 | -1.29287857 | -1.334031 | 1.334031 | Down-regulated |
| PKP4 | 2.35E-03 | 4.59E-04 | -3.719319 | -0.57330767 | -1.333228 | 1.333228 | Down-regulated |
| MTHFS | 8.04E-06 | 3.16E-07 | -5.793674 | 6.42095488 | -1.332437 | 1.332437 | Down-regulated |
| SSH2 | 1.36E-04 | 1.06E-05 | -4.833743 | 3.02415933 | -1.332424 | 1.332424 | Down-regulated |
| ZBTB45 | 1.29E-07 | 2.17E-09 | -7.104158 | 11.26875799 | -1.332407 | 1.332407 | Down-regulated |
| RAB13 | 3.31E-06 | 1.08E-07 | -6.078922 | 7.46265465 | -1.3324 | 1.3324 | Down-regulated |
| ANO9 | 9.61E-04 | 1.33E-04 | -4.100381 | 0.60184214 | -1.332137 | 1.332137 | Down-regulated |
| RPL23 | 1.00E-06 | 2.61E-08 | -6.453435 | 8.8445353 | -1.332073 | 1.332073 | Down-regulated |
| BPI | 6.06E-09 | 5.38E-11 | -8.070658 | 14.87934324 | -1.331789 | 1.331789 | Down-regulated |
| PDSS2 | 7.19E-09 | 6.73E-11 | -8.012326 | 14.66207427 | -1.331763 | 1.331763 | Down-regulated |
| OTUD6B | 1.00E-03 | 1.40E-04 | -4.084036 | 0.55013078 | -1.331698 | 1.331698 | Down-regulated |
| FAM173A | 1.16E-06 | 3.11E-08 | -6.407276 | 8.67351631 | -1.331513 | 1.331513 | Down-regulated |
| SLC40A1 | 4.57E-07 | 9.92E-09 | -6.707338 | 9.78794574 | -1.331405 | 1.331405 | Down-regulated |
| RRAGB | 8.00E-05 | 5.35E-06 | -5.024151 | 3.68105855 | -1.331294 | 1.331294 | Down-regulated |
| NFYB | 9.12E-08 | 1.44E-09 | -7.212089 | 11.67226844 | -1.330726 | 1.330726 | Down-regulated |
| FBXL18 | 2.59E-03 | 5.30E-04 | -3.674075 | -0.70835898 | -1.330664 | 1.330664 | Down-regulated |
| SGSM2 | 8.34E-08 | 1.30E-09 | -7.238298 | 11.77027615 | -1.330169 | 1.330169 | Down-regulated |
| ZNF773 | 5.05E-03 | 1.53E-03 | -3.328978 | -1.70392533 | -1.32967 | 1.32967 | Down-regulated |
| ERI3 | 6.70E-04 | 8.26E-05 | -4.242167 | 1.05497727 | -1.329634 | 1.329634 | Down-regulated |
| FRS2 | 7.63E-03 | 3.18E-03 | -3.081307 | -2.37773225 | -1.329184 | 1.329184 | Down-regulated |
| CTRL | 1.73E-04 | 1.44E-05 | -4.747011 | 2.72833324 | -1.329145 | 1.329145 | Down-regulated |
| ITGB1 | 3.75E-05 | 2.10E-06 | -5.282267 | 4.58631107 | -1.328463 | 1.328463 | Down-regulated |
| TM2D3 | 4.63E-04 | 5.08E-05 | -4.384958 | 1.51921387 | -1.328282 | 1.328282 | Down-regulated |
| FAAP100 | 5.16E-05 | 3.10E-06 | -5.175243 | 4.20903653 | -1.328244 | 1.328244 | Down-regulated |
| LINC01018 | 1.26E-04 | 9.58E-06 | -4.861626 | 3.11972712 | -1.328097 | 1.328097 | Down-regulated |
| ZNF14 | 2.29E-05 | 1.14E-06 | -5.447574 | 5.17393438 | -1.327411 | 1.327411 | Down-regulated |
| ZBTB22 | 2.35E-07 | 4.46E-09 | -6.91644 | 10.56755211 | -1.327366 | 1.327366 | Down-regulated |
| MED23 | 4.27E-09 | 3.41E-11 | -8.190634 | 15.32573474 | -1.327346 | 1.327346 | Down-regulated |
| DHX9 | 9.15E-09 | 8.95E-11 | -7.937591 | 14.38349933 | -1.326761 | 1.326761 | Down-regulated |
| UROD | 1.47E-03 | 2.35E-04 | -3.927814 | 0.06160963 | -1.326314 | 1.326314 | Down-regulated |
| PAPSS2 | 7.59E-07 | 1.85E-08 | -6.543997 | 9.18053641 | -1.326282 | 1.326282 | Down-regulated |
| H3F3AP4 | 1.60E-07 | 2.82E-09 | -7.036369 | 11.01542958 | -1.325455 | 1.325455 | Down-regulated |
| ST14 | 6.27E-08 | 9.31E-10 | -7.32563 | 12.09688614 | -1.325165 | 1.325165 | Down-regulated |
| LINC01567 | 8.69E-06 | 3.49E-07 | -5.76716 | 6.32471586 | -1.324868 | 1.324868 | Down-regulated |
| UBTD2 | 1.88E-06 | 5.50E-08 | -6.257637 | 8.1203697 | -1.32471 | 1.32471 | Down-regulated |
| HSPA1L | 2.32E-06 | 7.11E-08 | -6.189767 | 7.87018917 | -1.324637 | 1.324637 | Down-regulated |
| RANBP1 | 6.36E-08 | 9.49E-10 | -7.320632 | 12.07819202 | -1.324432 | 1.324432 | Down-regulated |
| SPTLC1 | 6.76E-05 | 4.35E-06 | -5.081637 | 3.88127106 | -1.324112 | 1.324112 | Down-regulated |
| SCMH1 | 3.02E-06 | 9.68E-08 | -6.108041 | 7.56957809 | -1.323605 | 1.323605 | Down-regulated |
| SFT2D2 | 4.48E-06 | 1.56E-07 | -5.982131 | 7.10797174 | -1.323439 | 1.323439 | Down-regulated |
| RABGEF1 | 1.50E-02 | 9.89E-03 | -2.669553 | -3.41375639 | -1.323288 | 1.323288 | Down-regulated |
| AGPAT2 | 2.46E-03 | 4.93E-04 | -3.696999 | -0.64005829 | -1.323162 | 1.323162 | Down-regulated |
| ZNF516 | 5.37E-05 | 3.27E-06 | -5.160557 | 4.1574745 | -1.323022 | 1.323022 | Down-regulated |
| CKS1B | 6.51E-04 | 7.95E-05 | -4.253422 | 1.09128872 | -1.323008 | 1.323008 | Down-regulated |
| SLC33A1 | 9.77E-05 | 6.93E-06 | -4.952384 | 3.43230804 | -1.322644 | 1.322644 | Down-regulated |
| SLC25A51 | 2.94E-05 | 1.56E-06 | -5.362606 | 4.87118281 | -1.321862 | 1.321862 | Down-regulated |
| SBF2 | 1.01E-04 | 7.20E-06 | -4.941718 | 3.39545586 | -1.321642 | 1.321642 | Down-regulated |
| ANKMY1 | 1.16E-03 | 1.70E-04 | -4.025324 | 0.3653026 | -1.321418 | 1.321418 | Down-regulated |
| ZNF319 | 3.74E-06 | 1.26E-07 | -6.039083 | 7.31652897 | -1.321412 | 1.321412 | Down-regulated |
| CLEC2B | 1.44E-08 | 1.53E-10 | -7.797351 | 13.86021418 | -1.321385 | 1.321385 | Down-regulated |
| COMMD5 | 3.75E-03 | 9.44E-04 | -3.489003 | -1.25008841 | -1.32106 | 1.32106 | Down-regulated |
| SNTB1 | 1.28E-04 | 9.79E-06 | -4.855749 | 3.09956375 | -1.320432 | 1.320432 | Down-regulated |
| MAN2B2 | 1.18E-03 | 1.76E-04 | -4.015947 | 0.33591889 | -1.320402 | 1.320402 | Down-regulated |
| FAAP20 | 1.65E-05 | 7.60E-07 | -5.557813 | 5.56883399 | -1.320278 | 1.320278 | Down-regulated |
| DCAF5 | 1.41E-04 | 1.10E-05 | -4.822907 | 2.9870788 | -1.320044 | 1.320044 | Down-regulated |
| THBD | 3.07E-06 | 9.87E-08 | -6.103074 | 7.55133484 | -1.319852 | 1.319852 | Down-regulated |
| MRPS24 | 3.67E-05 | 2.03E-06 | -5.290458 | 4.61528986 | -1.31968 | 1.31968 | Down-regulated |
| NUDT22 | 8.72E-04 | 1.16E-04 | -4.140174 | 0.72819998 | -1.319532 | 1.319532 | Down-regulated |
| SPNS1 | 3.42E-05 | 1.87E-06 | -5.313761 | 4.69781945 | -1.319247 | 1.319247 | Down-regulated |
| SLC27A3 | 2.66E-05 | 1.38E-06 | -5.395451 | 4.98803865 | -1.31915 | 1.31915 | Down-regulated |
| CETP | 2.15E-09 | 1.53E-11 | -8.401318 | 16.10778573 | -1.318361 | 1.318361 | Down-regulated |
| SDHC | 1.68E-04 | 1.39E-05 | -4.757265 | 2.76319035 | -1.31827 | 1.31827 | Down-regulated |
| THEMIS2 | 1.79E-03 | 3.10E-04 | -3.842079 | -0.20191751 | -1.31826 | 1.31826 | Down-regulated |
| PIGN | 4.26E-06 | 1.46E-07 | -5.998318 | 7.16720728 | -1.318196 | 1.318196 | Down-regulated |
| PAFAH1B3 | 2.54E-03 | 5.16E-04 | -3.682749 | -0.68254599 | -1.317945 | 1.317945 | Down-regulated |
| ATP9A | 1.50E-07 | 2.61E-09 | -7.056919 | 11.09221494 | -1.317765 | 1.317765 | Down-regulated |
| GALNS | 1.61E-03 | 2.68E-04 | -3.886712 | -0.065143 | -1.317666 | 1.317666 | Down-regulated |
| USP8 | 7.16E-06 | 2.73E-07 | -5.832486 | 6.56203006 | -1.317335 | 1.317335 | Down-regulated |
| RASSF7 | 2.98E-06 | 9.53E-08 | -6.112257 | 7.58506947 | -1.317136 | 1.317136 | Down-regulated |
| SNAPC4 | 2.83E-06 | 8.95E-08 | -6.128813 | 7.64591545 | -1.317092 | 1.317092 | Down-regulated |
| CCL3L1 | 8.72E-04 | 1.16E-04 | -4.140386 | 0.72887459 | -1.316304 | 1.316304 | Down-regulated |
| HCFC1 | 1.56E-05 | 7.12E-07 | -5.575294 | 5.6316636 | -1.315702 | 1.315702 | Down-regulated |
| HK3 | 5.85E-05 | 3.62E-06 | -5.132369 | 4.05864806 | -1.314424 | 1.314424 | Down-regulated |
| GNPTG | 5.26E-06 | 1.88E-07 | -5.931505 | 6.9229326 | -1.314334 | 1.314334 | Down-regulated |
| SLC38A9 | 7.70E-03 | 3.22E-03 | -3.076663 | -2.39002017 | -1.314176 | 1.314176 | Down-regulated |
| MTMR2 | 7.33E-05 | 4.82E-06 | -5.053167 | 3.78201001 | -1.314121 | 1.314121 | Down-regulated |
| SYNCRIP | 3.86E-05 | 2.16E-06 | -5.273764 | 4.55624003 | -1.314027 | 1.314027 | Down-regulated |
| SVIL | 1.40E-05 | 6.20E-07 | -5.612693 | 5.76626539 | -1.313872 | 1.313872 | Down-regulated |
| KCTD17 | 2.43E-03 | 4.84E-04 | -3.702906 | -0.62241389 | -1.313648 | 1.313648 | Down-regulated |
| TDP2 | 8.12E-06 | 3.20E-07 | -5.789941 | 6.40740034 | -1.312721 | 1.312721 | Down-regulated |
| KCNJ15 | 2.31E-03 | 4.47E-04 | -3.728251 | -0.54652597 | -1.312692 | 1.312692 | Down-regulated |
| DUSP2 | 6.03E-08 | 8.85E-10 | -7.338619 | 12.14546527 | -1.3126 | 1.3126 | Down-regulated |
| SYT15 | 1.18E-03 | 1.75E-04 | -4.017154 | 0.33969903 | -1.312565 | 1.312565 | Down-regulated |
| SCNM1 | 4.22E-03 | 1.14E-03 | -3.426313 | -1.42953959 | -1.312284 | 1.312284 | Down-regulated |
| SH3GL1 | 4.74E-05 | 2.78E-06 | -5.205217 | 4.31443354 | -1.312279 | 1.312279 | Down-regulated |
| RTN3 | 1.94E-06 | 5.73E-08 | -6.246758 | 8.08023516 | -1.311807 | 1.311807 | Down-regulated |
| DHX37 | 4.44E-04 | 4.83E-05 | -4.399614 | 1.56728787 | -1.311718 | 1.311718 | Down-regulated |
| TRIM8 | 4.14E-04 | 4.40E-05 | -4.426581 | 1.65595189 | -1.31142 | 1.31142 | Down-regulated |
| FAM160A2 | 1.24E-08 | 1.29E-10 | -7.841251 | 14.02409193 | -1.310605 | 1.310605 | Down-regulated |
| MRPL51 | 3.12E-05 | 1.68E-06 | -5.342474 | 4.79966714 | -1.310417 | 1.310417 | Down-regulated |
| FBXO30 | 4.72E-04 | 5.20E-05 | -4.377801 | 1.49576465 | -1.310341 | 1.310341 | Down-regulated |
| RNASEK | 2.17E-03 | 4.09E-04 | -3.756012 | -0.46304662 | -1.309799 | 1.309799 | Down-regulated |
| TMLHE | 1.09E-04 | 7.94E-06 | -4.914493 | 3.30153053 | -1.30959 | 1.30959 | Down-regulated |
| EIF2B4 | 2.69E-07 | 5.33E-09 | -6.869864 | 10.39374003 | -1.309447 | 1.309447 | Down-regulated |
| VDR | 9.00E-07 | 2.29E-08 | -6.487646 | 8.97139526 | -1.309177 | 1.309177 | Down-regulated |
| FAM13B | 2.24E-03 | 4.25E-04 | -3.743719 | -0.50005818 | -1.308955 | 1.308955 | Down-regulated |
| ABCD3 | 5.06E-04 | 5.68E-05 | -4.352256 | 1.41222139 | -1.30892 | 1.30892 | Down-regulated |
| MYH9 | 1.39E-03 | 2.18E-04 | -3.950315 | 0.13131821 | -1.308713 | 1.308713 | Down-regulated |
| NUBPL | 6.85E-05 | 4.43E-06 | -5.076491 | 3.86331347 | -1.308303 | 1.308303 | Down-regulated |
| TMEM50B | 6.52E-08 | 9.78E-10 | -7.312692 | 12.04849649 | -1.308013 | 1.308013 | Down-regulated |
| CYBB | 2.00E-07 | 3.68E-09 | -6.966711 | 10.75523662 | -1.307948 | 1.307948 | Down-regulated |
| TMEM164 | 1.33E-05 | 5.83E-07 | -5.629366 | 5.82634923 | -1.307792 | 1.307792 | Down-regulated |
| CWC25 | 7.08E-07 | 1.69E-08 | -6.567344 | 9.26725651 | -1.307117 | 1.307117 | Down-regulated |
| NDUFB7 | 9.50E-05 | 6.68E-06 | -4.962718 | 3.46804268 | -1.30674 | 1.30674 | Down-regulated |
| CAPN1 | 5.40E-10 | 2.60E-12 | -8.870116 | 17.83758792 | -1.306533 | 1.306533 | Down-regulated |
| NDRG1 | 1.54E-05 | 7.03E-07 | -5.578961 | 5.6448497 | -1.305937 | 1.305937 | Down-regulated |
| CENPW | 2.82E-06 | 8.90E-08 | -6.130474 | 7.65201883 | -1.305914 | 1.305914 | Down-regulated |
| HSPA1A | 2.74E-06 | 8.56E-08 | -6.140535 | 7.68901151 | -1.30589 | 1.30589 | Down-regulated |
| PHGDH | 1.04E-03 | 1.47E-04 | -4.070208 | 0.50646893 | -1.305531 | 1.305531 | Down-regulated |
| DCTN6 | 3.91E-11 | 1.08E-13 | -9.725059 | 20.94403606 | -1.305328 | 1.305328 | Down-regulated |
| SUCNR1 | 4.58E-09 | 3.75E-11 | -8.165417 | 15.23196852 | -1.305009 | 1.305009 | Down-regulated |
| PLD2 | 1.14E-05 | 4.84E-07 | -5.679448 | 6.00712145 | -1.304388 | 1.304388 | Down-regulated |
| DHRS7 | 1.01E-08 | 1.00E-10 | -7.907472 | 14.27117351 | -1.304317 | 1.304317 | Down-regulated |
| MIDN | 6.69E-04 | 8.25E-05 | -4.242346 | 1.05555636 | -1.30431 | 1.30431 | Down-regulated |
| UTP11 | 6.17E-03 | 2.17E-03 | -3.212501 | -2.02527521 | -1.304279 | 1.304279 | Down-regulated |
| ELMOD3 | 4.42E-04 | 4.81E-05 | -4.400905 | 1.57152818 | -1.303651 | 1.303651 | Down-regulated |
| FGGY | 1.61E-03 | 2.68E-04 | -3.886799 | -0.06487511 | -1.303416 | 1.303416 | Down-regulated |
| CENPE | 4.81E-05 | 2.83E-06 | -5.200277 | 4.29704829 | -1.303268 | 1.303268 | Down-regulated |
| CLIC4 | 2.72E-03 | 5.71E-04 | -3.650769 | -0.77753564 | -1.303024 | 1.303024 | Down-regulated |
| UBE2I | 3.80E-06 | 1.28E-07 | -6.033633 | 7.29655422 | -1.30213 | 1.30213 | Down-regulated |
| PDCD4 | 5.70E-05 | 3.51E-06 | -5.14102 | 4.08895766 | -1.302085 | 1.302085 | Down-regulated |
| SAMSN1 | 7.73E-05 | 5.13E-06 | -5.035781 | 3.72149371 | -1.302084 | 1.302084 | Down-regulated |
| TMEM218 | 9.75E-07 | 2.52E-08 | -6.462458 | 8.87798505 | -1.30208 | 1.30208 | Down-regulated |
| USMG5 | 7.14E-06 | 2.72E-07 | -5.833723 | 6.56652993 | -1.301918 | 1.301918 | Down-regulated |
| MIR635 | 1.45E-06 | 4.07E-08 | -6.337008 | 8.41351818 | -1.301747 | 1.301747 | Down-regulated |
| CEP250 | 1.31E-06 | 3.60E-08 | -6.369335 | 8.533077 | -1.301407 | 1.301407 | Down-regulated |
| PPP4R1 | 9.33E-03 | 4.52E-03 | -2.956676 | -2.70284941 | -1.300817 | 1.300817 | Down-regulated |
| CCNH | 2.62E-06 | 8.13E-08 | -6.154239 | 7.73941763 | -1.300054 | 1.300054 | Down-regulated |
| SPACA6 | 4.71E-04 | 5.20E-05 | -4.37813 | 1.49684255 | -1.299962 | 1.299962 | Down-regulated |
| SLC3A2 | 1.71E-09 | 1.12E-11 | -8.484194 | 16.4146998 | -1.299802 | 1.299802 | Down-regulated |
| HIST1H2BJ | 4.90E-05 | 2.90E-06 | -5.193456 | 4.27305624 | -1.29959 | 1.29959 | Down-regulated |
| SNRNP40 | 2.33E-05 | 1.17E-06 | -5.441986 | 5.15397926 | -1.298854 | 1.298854 | Down-regulated |
| HDAC1 | 8.49E-03 | 3.83E-03 | -3.016029 | -2.549223 | -1.297628 | 1.297628 | Down-regulated |
| DOCK5 | 1.95E-05 | 9.46E-07 | -5.498811 | 5.35719082 | -1.297558 | 1.297558 | Down-regulated |
| ABHD2 | 2.41E-07 | 4.61E-09 | -6.908039 | 10.53619642 | -1.297455 | 1.297455 | Down-regulated |
| ZNF788 | 1.17E-06 | 3.15E-08 | -6.403905 | 8.66103272 | -1.297056 | 1.297056 | Down-regulated |
| UBQLN2 | 7.96E-07 | 1.97E-08 | -6.528051 | 9.1213335 | -1.296657 | 1.296657 | Down-regulated |
| RBMS1 | 1.20E-02 | 6.91E-03 | -2.803433 | -3.0890708 | -1.296655 | 1.296655 | Down-regulated |
| FARSB | 1.06E-04 | 7.68E-06 | -4.923735 | 3.33339325 | -1.296134 | 1.296134 | Down-regulated |
| DHRS13 | 4.01E-05 | 2.26E-06 | -5.2614 | 4.51254924 | -1.295253 | 1.295253 | Down-regulated |
| SNU13 | 9.67E-06 | 3.98E-07 | -5.732082 | 6.19755449 | -1.295173 | 1.295173 | Down-regulated |
| CENPM | 4.69E-06 | 1.64E-07 | -5.968263 | 7.05725034 | -1.294956 | 1.294956 | Down-regulated |
| VCP | 5.58E-05 | 3.42E-06 | -5.147728 | 4.11247097 | -1.294777 | 1.294777 | Down-regulated |
| RASA1 | 2.44E-05 | 1.24E-06 | -5.42494 | 5.09314366 | -1.294396 | 1.294396 | Down-regulated |
| PLCL2 | 4.34E-04 | 4.68E-05 | -4.408645 | 1.5969514 | -1.293942 | 1.293942 | Down-regulated |
| TBK1 | 1.44E-07 | 2.47E-09 | -7.070805 | 11.14410509 | -1.292923 | 1.292923 | Down-regulated |
| NABP1 | 1.64E-05 | 7.54E-07 | -5.559953 | 5.57652353 | -1.292693 | 1.292693 | Down-regulated |
| ZNF235 | 1.01E-03 | 1.42E-04 | -4.080743 | 0.53972679 | -1.292657 | 1.292657 | Down-regulated |
| NHSL2 | 6.37E-05 | 4.03E-06 | -5.102421 | 3.95386286 | -1.292152 | 1.292152 | Down-regulated |
| CDCA5 | 8.87E-05 | 6.10E-06 | -4.987814 | 3.55494105 | -1.292044 | 1.292044 | Down-regulated |
| TBL1X | 1.55E-03 | 2.55E-04 | -3.901833 | -0.01860065 | -1.291445 | 1.291445 | Down-regulated |
| CARD8 | 1.02E-06 | 2.68E-08 | -6.446712 | 8.81961547 | -1.290845 | 1.290845 | Down-regulated |
| BBS7 | 4.55E-05 | 2.65E-06 | -5.21825 | 4.36032937 | -1.290276 | 1.290276 | Down-regulated |
| CD177 | 2.85E-05 | 1.50E-06 | -5.374185 | 4.91235165 | -1.290031 | 1.290031 | Down-regulated |
| MTMR3 | 1.80E-02 | 1.30E-02 | -2.564221 | -3.66054098 | -1.289504 | 1.289504 | Down-regulated |
| NINJ2 | 8.21E-05 | 5.51E-06 | -5.015973 | 3.65264558 | -1.289126 | 1.289126 | Down-regulated |
| CEBPZ | 1.58E-04 | 1.28E-05 | -4.779689 | 2.83953127 | -1.288837 | 1.288837 | Down-regulated |
| SIGLEC9 | 1.45E-08 | 1.55E-10 | -7.793891 | 13.84729701 | -1.288578 | 1.288578 | Down-regulated |
| FAM133CP | 2.15E-08 | 2.56E-10 | -7.66308 | 13.358668 | -1.288567 | 1.288567 | Down-regulated |
| KIAA1429 | 2.50E-06 | 7.73E-08 | -6.167639 | 7.78872519 | -1.28778 | 1.28778 | Down-regulated |
| ZMYM6 | 6.00E-07 | 1.37E-08 | -6.622481 | 9.47219064 | -1.287663 | 1.287663 | Down-regulated |
| COX6A1 | 5.08E-04 | 5.72E-05 | -4.350241 | 1.40564375 | -1.287536 | 1.287536 | Down-regulated |
| EPN1 | 4.76E-05 | 2.80E-06 | -5.203458 | 4.30824512 | -1.287418 | 1.287418 | Down-regulated |
| IL4R | 2.10E-07 | 3.91E-09 | -6.951139 | 10.69709191 | -1.286647 | 1.286647 | Down-regulated |
| ROCK2 | 7.63E-04 | 9.81E-05 | -4.191151 | 0.89100984 | -1.286525 | 1.286525 | Down-regulated |
| DDT | 1.45E-03 | 2.32E-04 | -3.93177 | 0.07384662 | -1.286298 | 1.286298 | Down-regulated |
| RIPK1 | 1.48E-05 | 6.67E-07 | -5.592983 | 5.69529552 | -1.286204 | 1.286204 | Down-regulated |
| RPL29 | 9.47E-03 | 4.64E-03 | -2.947469 | -2.72648246 | -1.286015 | 1.286015 | Down-regulated |
| PXK | 5.56E-05 | 3.41E-06 | -5.148669 | 4.11577309 | -1.285879 | 1.285879 | Down-regulated |
| HSPA5 | 2.50E-04 | 2.30E-05 | -4.613326 | 2.27684639 | -1.285878 | 1.285878 | Down-regulated |
| HNMT | 4.36E-04 | 4.72E-05 | -4.40602 | 1.58832735 | -1.285272 | 1.285272 | Down-regulated |
| ERLIN1 | 8.58E-08 | 1.34E-09 | -7.22982 | 11.73856909 | -1.285207 | 1.285207 | Down-regulated |
| BTF3L4 | 2.23E-04 | 2.00E-05 | -4.653927 | 2.41337265 | -1.284918 | 1.284918 | Down-regulated |
| TRIM33 | 6.21E-04 | 7.48E-05 | -4.271554 | 1.14988877 | -1.284731 | 1.284731 | Down-regulated |
| ITGA5 | 4.68E-06 | 1.63E-07 | -5.969685 | 7.06244942 | -1.284562 | 1.284562 | Down-regulated |
| SCARNA16 | 1.28E-03 | 1.95E-04 | -3.984087 | 0.23636381 | -1.284138 | 1.284138 | Down-regulated |
| HIST1H2AE | 1.28E-05 | 5.58E-07 | -5.641117 | 5.86872778 | -1.283979 | 1.283979 | Down-regulated |
| PRKCSH | 5.54E-05 | 3.39E-06 | -5.150321 | 4.1215632 | -1.283844 | 1.283844 | Down-regulated |
| SPOUT1 | 2.16E-03 | 4.04E-04 | -3.759794 | -0.45164764 | -1.283602 | 1.283602 | Down-regulated |
| PRELID1 | 5.14E-09 | 4.44E-11 | -8.121246 | 15.06764781 | -1.283459 | 1.283459 | Down-regulated |
| CLDN14 | 1.22E-03 | 1.83E-04 | -4.003466 | 0.29686659 | -1.282755 | 1.282755 | Down-regulated |
| HEXDC | 1.46E-04 | 1.16E-05 | -4.808975 | 2.93945256 | -1.28248 | 1.28248 | Down-regulated |
| ZNF615 | 4.55E-05 | 2.64E-06 | -5.218779 | 4.36219297 | -1.281928 | 1.281928 | Down-regulated |
| TMEM170A | 1.67E-04 | 1.38E-05 | -4.758594 | 2.76771064 | -1.281752 | 1.281752 | Down-regulated |
| EIF4G2 | 7.12E-04 | 8.98E-05 | -4.217404 | 0.97526189 | -1.281689 | 1.281689 | Down-regulated |
| EXOC7 | 4.39E-05 | 2.54E-06 | -5.230064 | 4.40196268 | -1.281632 | 1.281632 | Down-regulated |
| MPC1 | 2.16E-03 | 4.04E-04 | -3.759524 | -0.45246032 | -1.281317 | 1.281317 | Down-regulated |
| FAM49A | 1.46E-04 | 1.16E-05 | -4.809157 | 2.94007695 | -1.281065 | 1.281065 | Down-regulated |
| SCAMP1 | 2.47E-05 | 1.25E-06 | -5.422189 | 5.08333089 | -1.28106 | 1.28106 | Down-regulated |
| AP1B1 | 1.11E-07 | 1.84E-09 | -7.147469 | 11.43065843 | -1.280646 | 1.280646 | Down-regulated |
| ACOT13 | 6.37E-07 | 1.48E-08 | -6.602337 | 9.39729664 | -1.280524 | 1.280524 | Down-regulated |
| ANKRD9 | 6.13E-05 | 3.85E-06 | -5.115035 | 3.9979721 | -1.280501 | 1.280501 | Down-regulated |
| ELK1 | 5.76E-04 | 6.77E-05 | -4.300966 | 1.24521481 | -1.28041 | 1.28041 | Down-regulated |
| PPP2R5A | 3.67E-07 | 7.64E-09 | -6.775784 | 10.04292099 | -1.280086 | 1.280086 | Down-regulated |
| FTSJ1 | 7.21E-04 | 9.11E-05 | -4.212971 | 0.96101434 | -1.280082 | 1.280082 | Down-regulated |
| SNAI3-AS1 | 8.73E-08 | 1.37E-09 | -7.224975 | 11.72045314 | -1.279996 | 1.279996 | Down-regulated |
| KLHL22 | 5.25E-03 | 1.64E-03 | -3.30693 | -1.76534638 | -1.279479 | 1.279479 | Down-regulated |
| AKR7A3 | 6.46E-06 | 2.41E-07 | -5.866137 | 6.68452424 | -1.279412 | 1.279412 | Down-regulated |
| PRKCI | 3.48E-05 | 1.91E-06 | -5.308092 | 4.67773019 | -1.278998 | 1.278998 | Down-regulated |
| SNRPD1 | 1.88E-05 | 8.98E-07 | -5.512903 | 5.40767936 | -1.278464 | 1.278464 | Down-regulated |
| MRPS28 | 1.98E-03 | 3.57E-04 | -3.798447 | -0.33473388 | -1.278367 | 1.278367 | Down-regulated |
| GOLPH3L | 9.14E-05 | 6.35E-06 | -4.976837 | 3.51690982 | -1.278264 | 1.278264 | Down-regulated |
| FTH1P3 | 1.60E-04 | 1.30E-05 | -4.775643 | 2.82574435 | -1.277991 | 1.277991 | Down-regulated |
| ALPP | 3.63E-05 | 2.01E-06 | -5.293592 | 4.62638337 | -1.276903 | 1.276903 | Down-regulated |
| MIB2 | 4.29E-04 | 4.62E-05 | -4.412556 | 1.60980733 | -1.275627 | 1.275627 | Down-regulated |
| DGCR6 | 6.74E-04 | 8.33E-05 | -4.239415 | 1.04610626 | -1.275468 | 1.275468 | Down-regulated |
| SCAF11 | 2.63E-04 | 2.44E-05 | -4.596325 | 2.21983952 | -1.275213 | 1.275213 | Down-regulated |
| AP3M1 | 7.63E-05 | 5.05E-06 | -5.040156 | 3.73671751 | -1.275126 | 1.275126 | Down-regulated |
| LYG1 | 9.02E-03 | 4.26E-03 | -2.978292 | -2.64715674 | -1.27498 | 1.27498 | Down-regulated |
| FAM175A | 9.17E-03 | 4.39E-03 | -2.967497 | -2.67500568 | -1.274768 | 1.274768 | Down-regulated |
| NKAP | 1.39E-02 | 8.84E-03 | -2.71157 | -3.31316665 | -1.27374 | 1.27374 | Down-regulated |
| RPL32 | 6.10E-07 | 1.40E-08 | -6.616839 | 9.45120883 | -1.273472 | 1.273472 | Down-regulated |
| CFB | 2.13E-04 | 1.89E-05 | -4.670241 | 2.46837876 | -1.273444 | 1.273444 | Down-regulated |
| PHKB | 4.01E-03 | 1.05E-03 | -3.453664 | -1.35150454 | -1.273032 | 1.273032 | Down-regulated |
| SPIN3 | 5.42E-04 | 6.24E-05 | -4.324922 | 1.32309618 | -1.272845 | 1.272845 | Down-regulated |
| SP140 | 5.74E-05 | 3.54E-06 | -5.138522 | 4.0802043 | -1.272649 | 1.272649 | Down-regulated |
| AMOTL2 | 2.08E-03 | 3.82E-04 | -3.77717 | -0.39917834 | -1.272604 | 1.272604 | Down-regulated |
| SLC35F2 | 6.16E-05 | 3.88E-06 | -5.11304 | 3.99099413 | -1.272582 | 1.272582 | Down-regulated |
| C8orf37 | 1.51E-02 | 1.00E-02 | -2.664334 | -3.42616651 | -1.27252 | 1.27252 | Down-regulated |
| TTC13 | 5.05E-03 | 1.54E-03 | -3.328278 | -1.70588065 | -1.272364 | 1.272364 | Down-regulated |
| CARD17 | 4.75E-07 | 1.04E-08 | -6.695886 | 9.74530987 | -1.272245 | 1.272245 | Down-regulated |
| MR1 | 8.80E-07 | 2.22E-08 | -6.496094 | 9.00273303 | -1.271612 | 1.271612 | Down-regulated |
| ERP27 | 6.03E-08 | 8.86E-10 | -7.338545 | 12.14519152 | -1.271193 | 1.271193 | Down-regulated |
| CCDC125 | 1.94E-05 | 9.39E-07 | -5.500805 | 5.36433013 | -1.270519 | 1.270519 | Down-regulated |
| HIST1H2BC | 2.12E-05 | 1.04E-06 | -5.472602 | 5.2633857 | -1.270042 | 1.270042 | Down-regulated |
| SMIM3 | 1.34E-03 | 2.08E-04 | -3.965367 | 0.17807561 | -1.26994 | 1.26994 | Down-regulated |
| OGFOD3 | 2.50E-03 | 5.02E-04 | -3.691148 | -0.65751355 | -1.269711 | 1.269711 | Down-regulated |
| LSM7 | 9.74E-04 | 1.35E-04 | -4.0945 | 0.58322277 | -1.269213 | 1.269213 | Down-regulated |
| NSDHL | 6.51E-05 | 4.15E-06 | -5.094471 | 3.92608379 | -1.269076 | 1.269076 | Down-regulated |
| TNFRSF19 | 9.10E-06 | 3.69E-07 | -5.751913 | 6.26941815 | -1.268524 | 1.268524 | Down-regulated |
| ZNF174 | 3.63E-04 | 3.74E-05 | -4.47414 | 1.8129436 | -1.268396 | 1.268396 | Down-regulated |
| TMEM80 | 1.55E-04 | 1.24E-05 | -4.788063 | 2.86807482 | -1.267769 | 1.267769 | Down-regulated |
| HAL | 2.00E-04 | 1.74E-05 | -4.693389 | 2.54656831 | -1.267205 | 1.267205 | Down-regulated |
| YPEL5 | 1.21E-06 | 3.28E-08 | -6.393434 | 8.6222673 | -1.267014 | 1.267014 | Down-regulated |
| SAYSD1 | 1.19E-08 | 1.23E-10 | -7.85483 | 14.07476676 | -1.26695 | 1.26695 | Down-regulated |
| GP6 | 3.40E-04 | 3.43E-05 | -4.499074 | 1.89556679 | -1.266598 | 1.266598 | Down-regulated |
| C4orf48 | 1.39E-05 | 6.15E-07 | -5.614755 | 5.77369307 | -1.266522 | 1.266522 | Down-regulated |
| CCT5 | 2.87E-04 | 2.74E-05 | -4.563678 | 2.11062972 | -1.266251 | 1.266251 | Down-regulated |
| TOX2 | 4.52E-07 | 9.76E-09 | -6.711831 | 9.80467553 | -1.266175 | 1.266175 | Down-regulated |
| EIF4EBP3 | 2.36E-05 | 1.19E-06 | -5.436248 | 5.13349476 | -1.265858 | 1.265858 | Down-regulated |
| INTS12 | 9.87E-06 | 4.08E-07 | -5.725194 | 6.17260711 | -1.265776 | 1.265776 | Down-regulated |
| TMEM217 | 3.97E-03 | 1.04E-03 | -3.458974 | -1.33630821 | -1.265529 | 1.265529 | Down-regulated |
| SLC16A6 | 6.44E-03 | 2.34E-03 | -3.186786 | -2.09516536 | -1.265314 | 1.265314 | Down-regulated |
| ST6GALNAC2 | 2.36E-05 | 1.19E-06 | -5.435964 | 5.13247811 | -1.265163 | 1.265163 | Down-regulated |
| SLC4A5 | 8.06E-06 | 3.17E-07 | -5.79253 | 6.41680123 | -1.26503 | 1.26503 | Down-regulated |
| SIGIRR | 1.44E-04 | 1.13E-05 | -4.815209 | 2.96075632 | -1.264712 | 1.264712 | Down-regulated |
| SIRPD | 3.42E-05 | 1.87E-06 | -5.313496 | 4.69687865 | -1.264543 | 1.264543 | Down-regulated |
| RPL13AP3 | 3.42E-07 | 7.03E-09 | -6.797657 | 10.12444757 | -1.264479 | 1.264479 | Down-regulated |
| HERC4 | 1.95E-05 | 9.45E-07 | -5.499002 | 5.35787177 | -1.264143 | 1.264143 | Down-regulated |
| INTS7 | 1.04E-03 | 1.48E-04 | -4.067296 | 0.49728353 | -1.26409 | 1.26409 | Down-regulated |
| VCX3A | 2.51E-05 | 1.29E-06 | -5.415427 | 5.0592188 | -1.264087 | 1.264087 | Down-regulated |
| TUBB | 1.21E-03 | 1.80E-04 | -4.007923 | 0.31080199 | -1.26357 | 1.26357 | Down-regulated |
| PLXDC2 | 2.02E-04 | 1.76E-05 | -4.689526 | 2.53350758 | -1.263457 | 1.263457 | Down-regulated |
| ICA1 | 2.91E-06 | 9.24E-08 | -6.120323 | 7.61470856 | -1.263362 | 1.263362 | Down-regulated |
| NUP98 | 4.16E-05 | 2.38E-06 | -5.247954 | 4.46506962 | -1.263278 | 1.263278 | Down-regulated |
| PARD6A | 1.71E-07 | 3.06E-09 | -7.015026 | 10.93569372 | -1.262966 | 1.262966 | Down-regulated |
| HIST2H2AA4 | 6.26E-06 | 2.31E-07 | -5.877331 | 6.72531025 | -1.26292 | 1.26292 | Down-regulated |
| VPS51 | 4.85E-03 | 1.44E-03 | -3.349892 | -1.64541064 | -1.262865 | 1.262865 | Down-regulated |
| USP48 | 6.57E-04 | 8.04E-05 | -4.250065 | 1.08045413 | -1.262776 | 1.262776 | Down-regulated |
| SECTM1 | 6.46E-06 | 2.41E-07 | -5.865886 | 6.6836099 | -1.26189 | 1.26189 | Down-regulated |
| PRKAR1A | 7.54E-06 | 2.92E-07 | -5.81455 | 6.49680741 | -1.261469 | 1.261469 | Down-regulated |
| SLC9A6 | 1.76E-05 | 8.30E-07 | -5.534184 | 5.48399739 | -1.261402 | 1.261402 | Down-regulated |
| BCL9L | 2.42E-03 | 4.81E-04 | -3.70462 | -0.61729234 | -1.261226 | 1.261226 | Down-regulated |
| SH3BGR | 2.31E-04 | 2.09E-05 | -4.641015 | 2.36989741 | -1.26067 | 1.26067 | Down-regulated |
| ARID4A | 1.74E-07 | 3.14E-09 | -7.00807 | 10.90971062 | -1.260436 | 1.260436 | Down-regulated |
| HCN4 | 1.82E-05 | 8.60E-07 | -5.524494 | 5.44923665 | -1.260238 | 1.260238 | Down-regulated |
| PES1 | 1.66E-07 | 2.96E-09 | -7.024019 | 10.96928919 | -1.260165 | 1.260165 | Down-regulated |
| CATSPER1 | 1.37E-04 | 1.06E-05 | -4.832554 | 3.02008733 | -1.260114 | 1.260114 | Down-regulated |
| RPS21 | 1.08E-04 | 7.81E-06 | -4.918822 | 3.31645278 | -1.259926 | 1.259926 | Down-regulated |
| DNAJC28 | 2.23E-03 | 4.24E-04 | -3.74479 | -0.49683658 | -1.258931 | 1.258931 | Down-regulated |
| TRIB1 | 1.80E-08 | 2.05E-10 | -7.720512 | 13.57324996 | -1.258621 | 1.258621 | Down-regulated |
| GOLGA5 | 1.48E-04 | 1.18E-05 | -4.802871 | 2.91860593 | -1.258075 | 1.258075 | Down-regulated |
| TMX4 | 7.17E-05 | 4.68E-06 | -5.061382 | 3.81063209 | -1.257862 | 1.257862 | Down-regulated |
| HSPB11 | 5.82E-04 | 6.86E-05 | -4.297076 | 1.23258789 | -1.257849 | 1.257849 | Down-regulated |
| MSH5 | 1.38E-07 | 2.37E-09 | -7.081442 | 11.18385722 | -1.257211 | 1.257211 | Down-regulated |
| N4BP1 | 2.16E-03 | 4.05E-04 | -3.758684 | -0.45499348 | -1.256882 | 1.256882 | Down-regulated |
| FRMD3 | 3.81E-03 | 9.69E-04 | -3.480571 | -1.27434601 | -1.256038 | 1.256038 | Down-regulated |
| HEMK1 | 9.06E-04 | 1.22E-04 | -4.12492 | 0.67968629 | -1.255722 | 1.255722 | Down-regulated |
| PAM | 8.12E-07 | 2.02E-08 | -6.521658 | 9.09759943 | -1.255129 | 1.255129 | Down-regulated |
| ACYP1 | 7.80E-06 | 3.05E-07 | -5.803147 | 6.45536904 | -1.254813 | 1.254813 | Down-regulated |
| SMA4 | 2.69E-04 | 2.52E-05 | -4.587288 | 2.18957372 | -1.254472 | 1.254472 | Down-regulated |
| ZDHHC12 | 6.34E-07 | 1.47E-08 | -6.604295 | 9.4045743 | -1.254328 | 1.254328 | Down-regulated |
| HOOK3 | 1.87E-07 | 3.41E-09 | -6.986273 | 10.82829228 | -1.254278 | 1.254278 | Down-regulated |
| RAB3GAP2 | 2.14E-07 | 4.00E-09 | -6.94465 | 10.6728638 | -1.254262 | 1.254262 | Down-regulated |
| SWT1 | 9.57E-05 | 6.77E-06 | -4.959029 | 3.45528124 | -1.254093 | 1.254093 | Down-regulated |
| HSPA1B | 8.95E-07 | 2.27E-08 | -6.490311 | 8.98128006 | -1.254022 | 1.254022 | Down-regulated |
| ZNF815P | 7.60E-06 | 2.96E-07 | -5.81138 | 6.48528833 | -1.253953 | 1.253953 | Down-regulated |
| ZNF322 | 1.91E-05 | 9.21E-07 | -5.505894 | 5.3825633 | -1.2535 | 1.2535 | Down-regulated |
| HSCB | 8.04E-09 | 7.69E-11 | -7.977132 | 14.53091742 | -1.253147 | 1.253147 | Down-regulated |
| STAMBP | 2.95E-04 | 2.83E-05 | -4.553773 | 2.07756335 | -1.253101 | 1.253101 | Down-regulated |
| TXNRD1 | 1.04E-05 | 4.31E-07 | -5.710469 | 6.11930378 | -1.252818 | 1.252818 | Down-regulated |
| PRDX6 | 1.20E-02 | 6.96E-03 | -2.800378 | -3.09661451 | -1.252736 | 1.252736 | Down-regulated |
| PRKRIP1 | 1.56E-05 | 7.11E-07 | -5.575669 | 5.63301326 | -1.252495 | 1.252495 | Down-regulated |
| TRRAP | 2.92E-03 | 6.36E-04 | -3.616033 | -0.88013242 | -1.252321 | 1.252321 | Down-regulated |
| ELMO2 | 2.07E-05 | 1.01E-06 | -5.481482 | 5.29515102 | -1.252196 | 1.252196 | Down-regulated |
| BCAT1 | 2.48E-05 | 1.26E-06 | -5.420055 | 5.07571988 | -1.252108 | 1.252108 | Down-regulated |
| SPTSSA | 6.71E-04 | 8.29E-05 | -4.241172 | 1.0517694 | -1.251467 | 1.251467 | Down-regulated |
| MYL5 | 9.02E-05 | 6.24E-06 | -4.981581 | 3.53334301 | -1.251262 | 1.251262 | Down-regulated |
| ZNF26 | 2.59E-06 | 8.02E-08 | -6.158003 | 7.7532645 | -1.251129 | 1.251129 | Down-regulated |
| AAED1 | 2.30E-04 | 2.08E-05 | -4.641866 | 2.3727609 | -1.251107 | 1.251107 | Down-regulated |
| CSK | 1.68E-07 | 3.00E-09 | -7.020206 | 10.95504454 | -1.250959 | 1.250959 | Down-regulated |
| PLA2G16 | 6.77E-04 | 8.38E-05 | -4.237897 | 1.04121396 | -1.250876 | 1.250876 | Down-regulated |
| FAM175B | 2.55E-03 | 5.20E-04 | -3.680243 | -0.69000676 | -1.250327 | 1.250327 | Down-regulated |
| GALNT4 | 2.71E-07 | 5.40E-09 | -6.866579 | 10.38148537 | -1.250138 | 1.250138 | Down-regulated |
| TOP1 | 8.06E-04 | 1.05E-04 | -4.170068 | 0.82354955 | -1.249278 | 1.249278 | Down-regulated |
| SUOX | 1.15E-03 | 1.68E-04 | -4.029083 | 0.37709073 | -1.249239 | 1.249239 | Down-regulated |
| PLP2 | 6.94E-07 | 1.64E-08 | -6.575246 | 9.29661395 | -1.249179 | 1.249179 | Down-regulated |
| NDUFA7 | 9.01E-03 | 4.25E-03 | -2.97858 | -2.64641253 | -1.248823 | 1.248823 | Down-regulated |
| BRMS1L | 1.28E-03 | 1.95E-04 | -3.984673 | 0.23819041 | -1.248233 | 1.248233 | Down-regulated |
| PDS5B | 2.91E-03 | 6.33E-04 | -3.617643 | -0.87539013 | -1.24805 | 1.24805 | Down-regulated |
| BOLA3 | 1.15E-06 | 3.07E-08 | -6.411311 | 8.68845833 | -1.247978 | 1.247978 | Down-regulated |
| QARS | 1.31E-06 | 3.62E-08 | -6.367853 | 8.52759609 | -1.247705 | 1.247705 | Down-regulated |
| CAPRIN1 | 1.82E-05 | 8.61E-07 | -5.524042 | 5.44761671 | -1.247624 | 1.247624 | Down-regulated |
| POGK | 2.87E-03 | 6.20E-04 | -3.624579 | -0.8549486 | -1.247598 | 1.247598 | Down-regulated |
| PHTF1 | 1.01E-03 | 1.41E-04 | -4.082118 | 0.54407047 | -1.247532 | 1.247532 | Down-regulated |
| SGPL1 | 2.79E-06 | 8.76E-08 | -6.134666 | 7.66743095 | -1.247166 | 1.247166 | Down-regulated |
| CHEK2 | 2.63E-02 | 2.15E-02 | -2.365306 | -4.10492219 | -1.246987 | 1.246987 | Down-regulated |
| NPTN | 1.13E-03 | 1.65E-04 | -4.034287 | 0.3934228 | -1.246628 | 1.246628 | Down-regulated |
| POMC | 2.86E-04 | 2.73E-05 | -4.56451 | 2.11340816 | -1.246273 | 1.246273 | Down-regulated |
| CYSLTR1 | 2.19E-04 | 1.96E-05 | -4.660049 | 2.43400506 | -1.24586 | 1.24586 | Down-regulated |
| TPI1 | 8.08E-06 | 3.18E-07 | -5.791687 | 6.41374158 | -1.245781 | 1.245781 | Down-regulated |
| INPP4A | 9.20E-04 | 1.25E-04 | -4.118162 | 0.65822215 | -1.245444 | 1.245444 | Down-regulated |
| DDX51 | 1.19E-05 | 5.11E-07 | -5.664607 | 5.95350884 | -1.245374 | 1.245374 | Down-regulated |
| LOC286254 | 4.29E-03 | 1.18E-03 | -3.416238 | -1.4581808 | -1.24483 | 1.24483 | Down-regulated |
| FKBP3 | 1.36E-05 | 5.99E-07 | -5.621925 | 5.79952859 | -1.2447 | 1.2447 | Down-regulated |
| PPP1R8 | 1.75E-05 | 8.23E-07 | -5.536362 | 5.49181259 | -1.244649 | 1.244649 | Down-regulated |
| CNOT9 | 1.66E-05 | 7.68E-07 | -5.554876 | 5.55828347 | -1.244613 | 1.244613 | Down-regulated |
| SIGLEC5 | 8.65E-06 | 3.46E-07 | -5.768915 | 6.3310831 | -1.244397 | 1.244397 | Down-regulated |
| HELLS | 1.87E-05 | 8.92E-07 | -5.514597 | 5.41375148 | -1.244291 | 1.244291 | Down-regulated |
| GPN1 | 2.49E-05 | 1.27E-06 | -5.418277 | 5.06937965 | -1.244267 | 1.244267 | Down-regulated |
| ZNF486 | 1.90E-06 | 5.60E-08 | -6.252941 | 8.10304461 | -1.244088 | 1.244088 | Down-regulated |
| C16orf87 | 3.84E-04 | 4.01E-05 | -4.453538 | 1.74483866 | -1.244001 | 1.244001 | Down-regulated |
| IRAK2 | 2.76E-03 | 5.84E-04 | -3.643253 | -0.79978479 | -1.243875 | 1.243875 | Down-regulated |
| NUSAP1 | 5.34E-03 | 1.69E-03 | -3.297326 | -1.79201648 | -1.243834 | 1.243834 | Down-regulated |
| FCAR | 3.06E-04 | 2.99E-05 | -4.538189 | 2.02560756 | -1.243474 | 1.243474 | Down-regulated |
| LMNB2 | 4.51E-04 | 4.93E-05 | -4.393655 | 1.54773078 | -1.243128 | 1.243128 | Down-regulated |
| PPP2R3C | 2.70E-05 | 1.41E-06 | -5.390485 | 4.97035675 | -1.242875 | 1.242875 | Down-regulated |
| CDK7 | 1.78E-03 | 3.07E-04 | -3.845362 | -0.1918855 | -1.242578 | 1.242578 | Down-regulated |
| STYXL1 | 1.19E-04 | 8.95E-06 | -4.880816 | 3.18562763 | -1.242497 | 1.242497 | Down-regulated |
| LOC646214 | 1.82E-03 | 3.18E-04 | -3.834637 | -0.22463261 | -1.242347 | 1.242347 | Down-regulated |
| SNX14 | 2.23E-06 | 6.80E-08 | -6.201558 | 7.91361611 | -1.24214 | 1.24214 | Down-regulated |
| VGLL4 | 5.24E-07 | 1.17E-08 | -6.665263 | 9.63133126 | -1.241249 | 1.241249 | Down-regulated |
| PPP4R3B | 6.57E-09 | 5.95E-11 | -8.044303 | 14.78119866 | -1.241161 | 1.241161 | Down-regulated |
| GBP2 | 3.50E-05 | 1.92E-06 | -5.306443 | 4.67188922 | -1.241144 | 1.241144 | Down-regulated |
| ARRDC4 | 1.17E-04 | 8.68E-06 | -4.889467 | 3.21537207 | -1.240997 | 1.240997 | Down-regulated |
| SGK3 | 1.39E-08 | 1.46E-10 | -7.808861 | 13.90318889 | -1.240965 | 1.240965 | Down-regulated |
| ZNF282 | 5.38E-04 | 6.16E-05 | -4.328333 | 1.33420274 | -1.240337 | 1.240337 | Down-regulated |
| GYPE | 4.10E-04 | 4.35E-05 | -4.429781 | 1.66648854 | -1.240317 | 1.240317 | Down-regulated |
| ALKBH7 | 8.79E-04 | 1.18E-04 | -4.136794 | 0.71744243 | -1.240146 | 1.240146 | Down-regulated |
| MBNL2 | 4.56E-03 | 1.30E-03 | -3.38313 | -1.551914 | -1.240085 | 1.240085 | Down-regulated |
| SNRNP70 | 1.13E-05 | 4.76E-07 | -5.683539 | 6.02190796 | -1.24008 | 1.24008 | Down-regulated |
| HAUS2 | 2.83E-04 | 2.69E-05 | -4.568922 | 2.12814504 | -1.239531 | 1.239531 | Down-regulated |
| KBTBD4 | 3.61E-03 | 8.90E-04 | -3.50826 | -1.19454583 | -1.238512 | 1.238512 | Down-regulated |
| C2orf74 | 1.64E-05 | 7.51E-07 | -5.560897 | 5.57991361 | -1.238408 | 1.238408 | Down-regulated |
| AMFR | 3.11E-03 | 7.00E-04 | -3.585469 | -0.96990578 | -1.238304 | 1.238304 | Down-regulated |
| ASB8 | 1.65E-05 | 7.63E-07 | -5.556925 | 5.56564463 | -1.238255 | 1.238255 | Down-regulated |
| B4GALT5 | 4.60E-04 | 5.04E-05 | -4.387007 | 1.52593086 | -1.238137 | 1.238137 | Down-regulated |
| FLVCR2 | 1.05E-06 | 2.76E-08 | -6.439011 | 8.79107508 | -1.238073 | 1.238073 | Down-regulated |
| SNRNP35 | 6.76E-03 | 2.55E-03 | -3.157523 | -2.17422391 | -1.237988 | 1.237988 | Down-regulated |
| PLIN3 | 1.89E-03 | 3.35E-04 | -3.818118 | -0.27496464 | -1.237623 | 1.237623 | Down-regulated |
| AIM1 | 6.51E-05 | 4.15E-06 | -5.094783 | 3.92717449 | -1.237364 | 1.237364 | Down-regulated |
| SLC39A8 | 3.02E-03 | 6.71E-04 | -3.599282 | -0.92939328 | -1.237298 | 1.237298 | Down-regulated |
| RPRD1B | 1.76E-03 | 3.03E-04 | -3.849091 | -0.18048878 | -1.237191 | 1.237191 | Down-regulated |
| KPTN | 1.12E-02 | 6.23E-03 | -2.841278 | -2.9951124 | -1.237015 | 1.237015 | Down-regulated |
| DNASE2 | 6.20E-04 | 7.46E-05 | -4.271971 | 1.15123807 | -1.237001 | 1.237001 | Down-regulated |
| SORT1 | 6.49E-05 | 4.13E-06 | -5.096182 | 3.93206003 | -1.236918 | 1.236918 | Down-regulated |
| TCAIM | 7.23E-04 | 9.15E-05 | -4.21166 | 0.95680329 | -1.236601 | 1.236601 | Down-regulated |
| SEMA4A | 2.48E-06 | 7.67E-08 | -6.169781 | 7.79660546 | -1.236593 | 1.236593 | Down-regulated |
| SH3GLB1 | 9.73E-06 | 4.01E-07 | -5.729702 | 6.18893279 | -1.236339 | 1.236339 | Down-regulated |
| PDE4C | 3.72E-05 | 2.07E-06 | -5.28561 | 4.59813573 | -1.235064 | 1.235064 | Down-regulated |
| AARS | 1.93E-06 | 5.67E-08 | -6.249569 | 8.09060422 | -1.235025 | 1.235025 | Down-regulated |
| PAXBP1 | 4.99E-06 | 1.76E-07 | -5.949344 | 6.98809758 | -1.234767 | 1.234767 | Down-regulated |
| IKBIP | 9.62E-07 | 2.47E-08 | -6.467787 | 8.89774378 | -1.234301 | 1.234301 | Down-regulated |
| URB2 | 6.67E-04 | 8.21E-05 | -4.243845 | 1.06038777 | -1.234183 | 1.234183 | Down-regulated |
| TMEM131 | 8.40E-04 | 1.11E-04 | -4.153896 | 0.77192358 | -1.23375 | 1.23375 | Down-regulated |
| LIX1L | 3.20E-06 | 1.04E-07 | -6.089317 | 7.50081289 | -1.233693 | 1.233693 | Down-regulated |
| DDIT3 | 1.72E-05 | 8.02E-07 | -5.543352 | 5.51690147 | -1.233402 | 1.233402 | Down-regulated |
| FAM216A | 6.34E-05 | 4.02E-06 | -5.103711 | 3.95837197 | -1.23313 | 1.23313 | Down-regulated |
| ICE1 | 1.19E-06 | 3.22E-08 | -6.398243 | 8.64007175 | -1.232882 | 1.232882 | Down-regulated |
| SLF1 | 5.78E-05 | 3.57E-06 | -5.136328 | 4.07251637 | -1.232501 | 1.232501 | Down-regulated |
| ARHGEF37 | 1.53E-05 | 6.95E-07 | -5.581947 | 5.65559101 | -1.232426 | 1.232426 | Down-regulated |
| RDX | 1.15E-03 | 1.69E-04 | -4.028593 | 0.37555372 | -1.231336 | 1.231336 | Down-regulated |
| RIN1 | 6.25E-04 | 7.54E-05 | -4.268823 | 1.14105434 | -1.231249 | 1.231249 | Down-regulated |
| TMBIM1 | 2.30E-05 | 1.15E-06 | -5.445557 | 5.16672874 | -1.231216 | 1.231216 | Down-regulated |
| TRAF3 | 7.91E-03 | 3.38E-03 | -3.059444 | -2.43546079 | -1.23089 | 1.23089 | Down-regulated |
| COPB1 | 8.68E-03 | 3.98E-03 | -3.00252 | -2.58438321 | -1.230093 | 1.230093 | Down-regulated |
| LMBR1 | 3.60E-05 | 1.99E-06 | -5.296239 | 4.63575165 | -1.229818 | 1.229818 | Down-regulated |
| POLE3 | 8.74E-06 | 3.51E-07 | -5.765329 | 6.31807082 | -1.229739 | 1.229739 | Down-regulated |
| HDAC4 | 6.61E-04 | 8.11E-05 | -4.247505 | 1.07219454 | -1.229395 | 1.229395 | Down-regulated |
| PMM2 | 2.87E-02 | 2.39E-02 | -2.321018 | -4.1998881 | -1.229251 | 1.229251 | Down-regulated |
| ATG10 | 1.05E-03 | 1.51E-04 | -4.062732 | 0.48289558 | -1.229209 | 1.229209 | Down-regulated |
| F12 | 1.05E-03 | 1.50E-04 | -4.063827 | 0.48634571 | -1.229004 | 1.229004 | Down-regulated |
| IFT122 | 3.19E-05 | 1.72E-06 | -5.335931 | 4.77644134 | -1.228711 | 1.228711 | Down-regulated |
| SRI | 9.80E-05 | 6.95E-06 | -4.951346 | 3.42871979 | -1.2283 | 1.2283 | Down-regulated |
| CSTF3 | 1.38E-02 | 8.70E-03 | -2.717686 | -3.29842553 | -1.227974 | 1.227974 | Down-regulated |
| C19orf38 | 2.12E-05 | 1.04E-06 | -5.473199 | 5.26551959 | -1.227015 | 1.227015 | Down-regulated |
| ATG12 | 8.96E-06 | 3.63E-07 | -5.756713 | 6.28682302 | -1.226875 | 1.226875 | Down-regulated |
| SLC6A12 | 2.35E-05 | 1.19E-06 | -5.437379 | 5.13753042 | -1.226782 | 1.226782 | Down-regulated |
| ZYG11B | 2.87E-03 | 6.21E-04 | -3.623821 | -0.85718152 | -1.226606 | 1.226606 | Down-regulated |
| DCTN1 | 1.43E-04 | 1.12E-05 | -4.817382 | 2.96818495 | -1.22654 | 1.22654 | Down-regulated |
| TLR1 | 3.62E-06 | 1.21E-07 | -6.049418 | 7.35441708 | -1.226407 | 1.226407 | Down-regulated |
| KDM3B | 6.35E-06 | 2.35E-07 | -5.872546 | 6.70787203 | -1.2261 | 1.2261 | Down-regulated |
| HIST1H2BH | 3.62E-04 | 3.72E-05 | -4.475592 | 1.81775092 | -1.225888 | 1.225888 | Down-regulated |
| STARD8 | 1.21E-04 | 9.08E-06 | -4.876787 | 3.17178475 | -1.224775 | 1.224775 | Down-regulated |
| LYPLA1 | 8.37E-06 | 3.33E-07 | -5.779386 | 6.3690801 | -1.224711 | 1.224711 | Down-regulated |
| AVIL | 1.07E-03 | 1.54E-04 | -4.056576 | 0.4635048 | -1.224164 | 1.224164 | Down-regulated |
| TAF6L | 9.07E-05 | 6.29E-06 | -4.979279 | 3.52536731 | -1.224026 | 1.224026 | Down-regulated |
| CCDC149 | 1.31E-06 | 3.60E-08 | -6.368969 | 8.53172404 | -1.223315 | 1.223315 | Down-regulated |
| RAF1 | 3.48E-03 | 8.37E-04 | -3.528107 | -1.13709412 | -1.223107 | 1.223107 | Down-regulated |
| MRPL19 | 1.01E-03 | 1.43E-04 | -4.07891 | 0.53393496 | -1.223083 | 1.223083 | Down-regulated |
| MGST3 | 1.76E-03 | 3.03E-04 | -3.849316 | -0.17980187 | -1.222959 | 1.222959 | Down-regulated |
| CMTM4 | 3.44E-06 | 1.14E-07 | -6.06573 | 7.41424781 | -1.222678 | 1.222678 | Down-regulated |
| ITPA | 2.62E-06 | 8.12E-08 | -6.154603 | 7.74075644 | -1.222512 | 1.222512 | Down-regulated |
| SNAPC2 | 5.06E-05 | 3.02E-06 | -5.182667 | 4.23512269 | -1.222357 | 1.222357 | Down-regulated |
| FAM13A | 2.70E-06 | 8.40E-08 | -6.145635 | 7.7077691 | -1.222053 | 1.222053 | Down-regulated |
| EXT2 | 3.68E-05 | 2.04E-06 | -5.289384 | 4.6114883 | -1.221826 | 1.221826 | Down-regulated |
| MUC1 | 2.36E-05 | 1.19E-06 | -5.435676 | 5.13145178 | -1.221717 | 1.221717 | Down-regulated |
| HNRNPUL1 | 3.94E-03 | 1.02E-03 | -3.463206 | -1.324187 | -1.221587 | 1.221587 | Down-regulated |
| GPKOW | 3.38E-05 | 1.84E-06 | -5.317503 | 4.71108377 | -1.220886 | 1.220886 | Down-regulated |
| SLK | 3.63E-05 | 2.01E-06 | -5.29361 | 4.62644679 | -1.220858 | 1.220858 | Down-regulated |
| TRMT10A | 1.13E-04 | 8.29E-06 | -4.902124 | 3.25892338 | -1.220857 | 1.220857 | Down-regulated |
| COA1 | 2.98E-04 | 2.88E-05 | -4.548763 | 2.06085272 | -1.220474 | 1.220474 | Down-regulated |
| RAB6A | 6.49E-03 | 2.37E-03 | -3.181839 | -2.10856639 | -1.220437 | 1.220437 | Down-regulated |
| PMS2 | 3.56E-07 | 7.35E-09 | -6.786061 | 10.08122475 | -1.220378 | 1.220378 | Down-regulated |
| ZNF223 | 1.71E-03 | 2.90E-04 | -3.862554 | -0.13928496 | -1.22034 | 1.22034 | Down-regulated |
| GTF3A | 5.67E-04 | 6.63E-05 | -4.307077 | 1.26506055 | -1.219948 | 1.219948 | Down-regulated |
| C8orf44 | 2.78E-04 | 2.63E-05 | -4.575378 | 2.14972507 | -1.21989 | 1.21989 | Down-regulated |
| ARL5B | 1.93E-03 | 3.43E-04 | -3.81079 | -0.29725053 | -1.219333 | 1.219333 | Down-regulated |
| SLAIN2 | 5.26E-04 | 5.98E-05 | -4.337253 | 1.36326821 | -1.219092 | 1.219092 | Down-regulated |
| SMIM19 | 1.23E-03 | 1.85E-04 | -4.000617 | 0.28796059 | -1.218564 | 1.218564 | Down-regulated |
| SNHG11 | 4.49E-05 | 2.60E-06 | -5.223289 | 4.37808264 | -1.218306 | 1.218306 | Down-regulated |
| GCN1 | 4.41E-09 | 3.56E-11 | -8.179048 | 15.28265728 | -1.218186 | 1.218186 | Down-regulated |
| CPSF3 | 4.08E-08 | 5.51E-10 | -7.462225 | 12.6077553 | -1.218133 | 1.218133 | Down-regulated |
| TPP2 | 4.40E-04 | 4.77E-05 | -4.402943 | 1.578219 | -1.218058 | 1.218058 | Down-regulated |
| RBMX2 | 4.80E-06 | 1.69E-07 | -5.960728 | 7.02970107 | -1.217937 | 1.217937 | Down-regulated |
| OAZ1 | 1.19E-04 | 8.91E-06 | -4.882002 | 3.18970506 | -1.217721 | 1.217721 | Down-regulated |
| SUMO1P3 | 1.28E-05 | 5.57E-07 | -5.641762 | 5.8710553 | -1.217504 | 1.217504 | Down-regulated |
| ZNF581 | 1.12E-03 | 1.64E-04 | -4.037341 | 0.40301424 | -1.217422 | 1.217422 | Down-regulated |
| RAP1A | 1.44E-02 | 9.31E-03 | -2.692238 | -3.35959844 | -1.217245 | 1.217245 | Down-regulated |
| YTHDF3 | 6.40E-06 | 2.38E-07 | -5.869648 | 6.69731574 | -1.216974 | 1.216974 | Down-regulated |
| EVI5 | 7.56E-03 | 3.13E-03 | -3.086933 | -2.36282976 | -1.216621 | 1.216621 | Down-regulated |
| PPM1E | 8.63E-06 | 3.45E-07 | -5.769714 | 6.33398208 | -1.216479 | 1.216479 | Down-regulated |
| FUT7 | 4.33E-03 | 1.20E-03 | -3.411835 | -1.47067999 | -1.216183 | 1.216183 | Down-regulated |
| RNASE1 | 1.33E-06 | 3.68E-08 | -6.363329 | 8.51085861 | -1.21603 | 1.21603 | Down-regulated |
| ABCG1 | 1.94E-06 | 5.75E-08 | -6.245768 | 8.07658582 | -1.215815 | 1.215815 | Down-regulated |
| VPS13A | 2.97E-05 | 1.58E-06 | -5.358947 | 4.85817871 | -1.21536 | 1.21536 | Down-regulated |
| TAF12 | 3.99E-05 | 2.25E-06 | -5.26359 | 4.52028462 | -1.215093 | 1.215093 | Down-regulated |
| NDUFA12 | 6.88E-06 | 2.60E-07 | -5.845726 | 6.61020608 | -1.214892 | 1.214892 | Down-regulated |
| HLTF | 5.09E-05 | 3.04E-06 | -5.180522 | 4.22758607 | -1.214344 | 1.214344 | Down-regulated |
| KCNH3 | 5.33E-09 | 4.64E-11 | -8.109658 | 15.02452446 | -1.214135 | 1.214135 | Down-regulated |
| KPNA4 | 2.28E-04 | 2.06E-05 | -4.645589 | 2.38529346 | -1.21397 | 1.21397 | Down-regulated |
| PRTFDC1 | 4.52E-03 | 1.28E-03 | -3.388909 | -1.5355963 | -1.213697 | 1.213697 | Down-regulated |
| RPS6KC1 | 4.28E-06 | 1.48E-07 | -5.996407 | 7.16021283 | -1.213316 | 1.213316 | Down-regulated |
| PAPD4 | 9.27E-07 | 2.37E-08 | -6.478988 | 8.9392794 | -1.21329 | 1.21329 | Down-regulated |
| SERAC1 | 2.59E-03 | 5.31E-04 | -3.673382 | -0.71042197 | -1.213153 | 1.213153 | Down-regulated |
| SSX2IP | 1.70E-06 | 4.93E-08 | -6.286421 | 8.22661021 | -1.213063 | 1.213063 | Down-regulated |
| FRAT1 | 4.94E-07 | 1.09E-08 | -6.683419 | 9.69890246 | -1.213007 | 1.213007 | Down-regulated |
| FBXO33 | 1.79E-07 | 3.26E-09 | -6.998597 | 10.87432518 | -1.21245 | 1.21245 | Down-regulated |
| PHF20L1 | 6.52E-07 | 1.52E-08 | -6.594862 | 9.36951173 | -1.212443 | 1.212443 | Down-regulated |
| DNA2 | 2.89E-03 | 6.26E-04 | -3.621512 | -0.86399043 | -1.212337 | 1.212337 | Down-regulated |
| TERF1 | 1.68E-04 | 1.39E-05 | -4.757421 | 2.7637219 | -1.210866 | 1.210866 | Down-regulated |
| WNK1 | 1.63E-05 | 7.50E-07 | -5.561488 | 5.58203796 | -1.210331 | 1.210331 | Down-regulated |
| PYCARD | 2.10E-05 | 1.03E-06 | -5.475866 | 5.27506203 | -1.210236 | 1.210236 | Down-regulated |
| TAF6 | 2.71E-04 | 2.54E-05 | -4.585206 | 2.18260254 | -1.210212 | 1.210212 | Down-regulated |
| FGFR1OP2 | 1.53E-06 | 4.37E-08 | -6.318201 | 8.34400057 | -1.210208 | 1.210208 | Down-regulated |
| CLEC4E | 1.26E-04 | 9.54E-06 | -4.863042 | 3.12458736 | -1.210157 | 1.210157 | Down-regulated |
| TPRKB | 5.27E-03 | 1.65E-03 | -3.30527 | -1.76995899 | -1.209891 | 1.209891 | Down-regulated |
| PGAM4 | 8.95E-06 | 3.62E-07 | -5.757358 | 6.28916217 | -1.209461 | 1.209461 | Down-regulated |
| APOOL | 6.51E-08 | 9.75E-10 | -7.313446 | 12.05131923 | -1.209422 | 1.209422 | Down-regulated |
| REC8 | 5.61E-03 | 1.84E-03 | -3.267938 | -1.87329743 | -1.209205 | 1.209205 | Down-regulated |
| ZNF577 | 1.84E-06 | 5.39E-08 | -6.262851 | 8.13960785 | -1.209115 | 1.209115 | Down-regulated |
| POMT1 | 6.05E-04 | 7.24E-05 | -4.280956 | 1.18032446 | -1.208966 | 1.208966 | Down-regulated |
| KPNB1 | 8.44E-05 | 5.70E-06 | -5.006735 | 3.62056849 | -1.20891 | 1.20891 | Down-regulated |
| FAM13A-AS1 | 2.25E-05 | 1.12E-06 | -5.453251 | 5.19421283 | -1.208347 | 1.208347 | Down-regulated |
| TCP1 | 3.76E-04 | 3.91E-05 | -4.461254 | 1.77032882 | -1.208196 | 1.208196 | Down-regulated |
| TMEM156 | 4.90E-07 | 1.08E-08 | -6.686236 | 9.70938767 | -1.207853 | 1.207853 | Down-regulated |
| ACAD10 | 2.13E-04 | 1.88E-05 | -4.670985 | 2.47088837 | -1.207656 | 1.207656 | Down-regulated |
| CD44 | 1.37E-06 | 3.81E-08 | -6.354447 | 8.47800295 | -1.207531 | 1.207531 | Down-regulated |
| CS | 5.23E-06 | 1.87E-07 | -5.933937 | 6.93181293 | -1.207515 | 1.207515 | Down-regulated |
| SMPDL3A | 1.41E-06 | 3.93E-08 | -6.346292 | 8.44784281 | -1.207204 | 1.207204 | Down-regulated |
| CCP110 | 2.51E-05 | 1.29E-06 | -5.415046 | 5.05785781 | -1.206951 | 1.206951 | Down-regulated |
| RANGAP1 | 2.66E-05 | 1.38E-06 | -5.395858 | 4.98948814 | -1.206781 | 1.206781 | Down-regulated |
| UBL3 | 6.86E-03 | 2.61E-03 | -3.149096 | -2.19689746 | -1.20671 | 1.20671 | Down-regulated |
| ZNF430 | 5.51E-04 | 6.38E-05 | -4.318307 | 1.30156713 | -1.206555 | 1.206555 | Down-regulated |
| NHLRC3 | 8.51E-07 | 2.13E-08 | -6.507021 | 9.04327882 | -1.206267 | 1.206267 | Down-regulated |
| ZFP36L1 | 9.44E-04 | 1.30E-04 | -4.107685 | 0.62498702 | -1.206259 | 1.206259 | Down-regulated |
| TAF15 | 3.45E-03 | 8.25E-04 | -3.532667 | -1.12386612 | -1.206177 | 1.206177 | Down-regulated |
| TSC22D2 | 1.74E-03 | 2.97E-04 | -3.855188 | -0.16183981 | -1.20604 | 1.20604 | Down-regulated |
| KREMEN1 | 2.16E-06 | 6.52E-08 | -6.212798 | 7.9550299 | -1.205822 | 1.205822 | Down-regulated |
| CORO1B | 4.98E-04 | 5.57E-05 | -4.358075 | 1.43122975 | -1.205224 | 1.205224 | Down-regulated |
| GNL3L | 2.73E-04 | 2.57E-05 | -4.582032 | 2.17198094 | -1.205205 | 1.205205 | Down-regulated |
| C22orf39 | 6.13E-06 | 2.25E-07 | -5.88364 | 6.74830182 | -1.205039 | 1.205039 | Down-regulated |
| COMMD10 | 2.91E-04 | 2.79E-05 | -4.557944 | 2.09148255 | -1.204602 | 1.204602 | Down-regulated |
| MARC1 | 5.16E-04 | 5.84E-05 | -4.343951 | 1.38511232 | -1.204247 | 1.204247 | Down-regulated |
| GCNT1 | 6.68E-03 | 2.49E-03 | -3.164871 | -2.15441922 | -1.204179 | 1.204179 | Down-regulated |
| SF3B1 | 1.16E-03 | 1.72E-04 | -4.022894 | 0.3576837 | -1.20406 | 1.20406 | Down-regulated |
| RAB38 | 9.63E-06 | 3.95E-07 | -5.733848 | 6.20395144 | -1.203944 | 1.203944 | Down-regulated |
| ACOT8 | 1.71E-06 | 4.95E-08 | -6.285547 | 8.22338481 | -1.203783 | 1.203783 | Down-regulated |
| SKA1 | 1.50E-04 | 1.19E-05 | -4.799874 | 2.90837556 | -1.203771 | 1.203771 | Down-regulated |
| HIGD2A | 9.16E-04 | 1.24E-04 | -4.120197 | 0.66468396 | -1.203255 | 1.203255 | Down-regulated |
| OARD1 | 3.74E-06 | 1.26E-07 | -6.038912 | 7.31590037 | -1.203152 | 1.203152 | Down-regulated |
| COX7A2 | 9.66E-05 | 6.84E-06 | -4.956006 | 3.44482958 | -1.203032 | 1.203032 | Down-regulated |
| ACTR3 | 3.59E-04 | 3.68E-05 | -4.47841 | 1.82707846 | -1.202341 | 1.202341 | Down-regulated |
| GHRL | 1.15E-03 | 1.70E-04 | -4.026621 | 0.36936734 | -1.201871 | 1.201871 | Down-regulated |
| TLR8 | 2.19E-06 | 6.63E-08 | -6.208179 | 7.93801286 | -1.200581 | 1.200581 | Down-regulated |
| METTL1 | 2.09E-03 | 3.85E-04 | -3.774847 | -0.4062007 | -1.199702 | 1.199702 | Down-regulated |
| SS18 | 1.41E-03 | 2.23E-04 | -3.94393 | 0.11151234 | -1.199546 | 1.199546 | Down-regulated |
| CCDC94 | 1.71E-05 | 7.95E-07 | -5.545737 | 5.52546372 | -1.19947 | 1.19947 | Down-regulated |
| NUDT16P1 | 3.06E-05 | 1.64E-06 | -5.349144 | 4.82334994 | -1.198592 | 1.198592 | Down-regulated |
| HMG20B | 5.88E-05 | 3.64E-06 | -5.130703 | 4.05281257 | -1.198545 | 1.198545 | Down-regulated |
| SLC2A8 | 1.45E-05 | 6.52E-07 | -5.599256 | 5.71787563 | -1.198544 | 1.198544 | Down-regulated |
| GCLM | 3.42E-06 | 1.12E-07 | -6.068686 | 7.42509133 | -1.198442 | 1.198442 | Down-regulated |
| EVI2A | 7.78E-04 | 1.00E-04 | -4.183921 | 0.86785578 | -1.198398 | 1.198398 | Down-regulated |
| MRPS36 | 2.51E-05 | 1.29E-06 | -5.415619 | 5.05990188 | -1.197544 | 1.197544 | Down-regulated |
| ATP5E | 1.43E-03 | 2.27E-04 | -3.937972 | 0.0930491 | -1.197431 | 1.197431 | Down-regulated |
| TTC9C | 5.51E-04 | 6.37E-05 | -4.318764 | 1.30305545 | -1.197403 | 1.197403 | Down-regulated |
| TMEM187 | 5.62E-04 | 6.53E-05 | -4.311474 | 1.27934942 | -1.197096 | 1.197096 | Down-regulated |
| ZNF561 | 1.86E-05 | 8.89E-07 | -5.515658 | 5.4175546 | -1.196865 | 1.196865 | Down-regulated |
| C21orf91 | 1.01E-06 | 2.62E-08 | -6.452464 | 8.84093581 | -1.196765 | 1.196765 | Down-regulated |
| ZNF320 | 2.64E-04 | 2.46E-05 | -4.594286 | 2.21300686 | -1.196655 | 1.196655 | Down-regulated |
| FGD2 | 4.70E-03 | 1.37E-03 | -3.366833 | -1.5978324 | -1.196589 | 1.196589 | Down-regulated |
| LMAN2 | 6.61E-04 | 8.10E-05 | -4.247758 | 1.07300887 | -1.196277 | 1.196277 | Down-regulated |
| ENTPD1 | 7.96E-05 | 5.32E-06 | -5.026003 | 3.68749395 | -1.195992 | 1.195992 | Down-regulated |
| TMEM254 | 4.52E-07 | 9.79E-09 | -6.710979 | 9.8015055 | -1.195879 | 1.195879 | Down-regulated |
| EXOSC4 | 2.88E-04 | 2.75E-05 | -4.562693 | 2.10734002 | -1.195566 | 1.195566 | Down-regulated |
| ASMTL | 1.77E-02 | 1.26E-02 | -2.57576 | -3.63388777 | -1.195372 | 1.195372 | Down-regulated |
| HAUS1 | 5.87E-06 | 2.14E-07 | -5.89712 | 6.79745087 | -1.195328 | 1.195328 | Down-regulated |
| SCAND2P | 9.39E-06 | 3.83E-07 | -5.741969 | 6.23337697 | -1.195314 | 1.195314 | Down-regulated |
| DHX35 | 1.47E-03 | 2.35E-04 | -3.927755 | 0.06142742 | -1.195132 | 1.195132 | Down-regulated |
| LYPLAL1 | 1.02E-03 | 1.44E-04 | -4.07701 | 0.52793451 | -1.19512 | 1.19512 | Down-regulated |
| UBE2G1 | 2.78E-04 | 2.62E-05 | -4.576442 | 2.15328356 | -1.194941 | 1.194941 | Down-regulated |
| GTF2B | 4.63E-06 | 1.62E-07 | -5.972241 | 7.0717978 | -1.194124 | 1.194124 | Down-regulated |
| SDHAP2 | 5.95E-04 | 7.08E-05 | -4.287766 | 1.20239286 | -1.193466 | 1.193466 | Down-regulated |
| CLEC12A | 8.66E-05 | 5.89E-06 | -4.99747 | 3.58841997 | -1.193135 | 1.193135 | Down-regulated |
| ZNF300P1 | 5.31E-08 | 7.61E-10 | -7.37821 | 12.29354158 | -1.192313 | 1.192313 | Down-regulated |
| CRCP | 4.18E-05 | 2.39E-06 | -5.246441 | 4.45972808 | -1.192091 | 1.192091 | Down-regulated |
| VPS25 | 1.52E-03 | 2.47E-04 | -3.912244 | 0.0135031 | -1.192008 | 1.192008 | Down-regulated |
| GTF2F2 | 4.69E-06 | 1.64E-07 | -5.968862 | 7.05944074 | -1.19189 | 1.19189 | Down-regulated |
| PGP | 2.47E-05 | 1.26E-06 | -5.421135 | 5.079573 | -1.191615 | 1.191615 | Down-regulated |
| PRIM1 | 7.35E-07 | 1.77E-08 | -6.555723 | 9.22408633 | -1.191582 | 1.191582 | Down-regulated |
| CGRRF1 | 1.81E-04 | 1.54E-05 | -4.727737 | 2.66289798 | -1.191393 | 1.191393 | Down-regulated |
| HARS2 | 7.53E-06 | 2.91E-07 | -5.815652 | 6.50081315 | -1.191353 | 1.191353 | Down-regulated |
| LMNB1 | 2.68E-04 | 2.51E-05 | -4.588583 | 2.1939073 | -1.191221 | 1.191221 | Down-regulated |
| SLC22A4 | 2.17E-04 | 1.93E-05 | -4.663915 | 2.44703828 | -1.191154 | 1.191154 | Down-regulated |
| PXMP2 | 7.64E-07 | 1.87E-08 | -6.541097 | 9.16976897 | -1.190857 | 1.190857 | Down-regulated |
| STX8 | 6.77E-08 | 1.02E-09 | -7.301417 | 12.00632759 | -1.190151 | 1.190151 | Down-regulated |
| ZDHHC13 | 1.75E-05 | 8.22E-07 | -5.536619 | 5.49273588 | -1.189948 | 1.189948 | Down-regulated |
| MMP9 | 8.94E-04 | 1.20E-04 | -4.130521 | 0.69748985 | -1.189697 | 1.189697 | Down-regulated |
| CSDE1 | 6.08E-06 | 2.23E-07 | -5.886414 | 6.75841409 | -1.189344 | 1.189344 | Down-regulated |
| DNAJB9 | 2.28E-03 | 4.38E-04 | -3.734196 | -0.52867977 | -1.189026 | 1.189026 | Down-regulated |
| GRIP2 | 2.81E-05 | 1.47E-06 | -5.378784 | 4.92871399 | -1.188999 | 1.188999 | Down-regulated |
| PIGQ | 1.98E-08 | 2.31E-10 | -7.689219 | 13.45634052 | -1.188964 | 1.188964 | Down-regulated |
| JAG1 | 9.63E-04 | 1.33E-04 | -4.099068 | 0.59768309 | -1.188576 | 1.188576 | Down-regulated |
| C1QC | 3.34E-04 | 3.36E-05 | -4.504838 | 1.91469606 | -1.188546 | 1.188546 | Down-regulated |
| DNAJC14 | 1.87E-04 | 1.60E-05 | -4.717779 | 2.62913583 | -1.188266 | 1.188266 | Down-regulated |
| GSTZ1 | 9.16E-05 | 6.37E-06 | -4.975825 | 3.51340765 | -1.188262 | 1.188262 | Down-regulated |
| CRABP2 | 1.06E-04 | 7.63E-06 | -4.925399 | 3.33913022 | -1.188056 | 1.188056 | Down-regulated |
| ANO10 | 1.53E-02 | 1.02E-02 | -2.657003 | -3.44356615 | -1.186939 | 1.186939 | Down-regulated |
| SEC24D | 1.57E-06 | 4.50E-08 | -6.310272 | 8.31470559 | -1.186804 | 1.186804 | Down-regulated |
| UQCC3 | 3.27E-04 | 3.25E-05 | -4.514046 | 1.94528169 | -1.186323 | 1.186323 | Down-regulated |
| PPCS | 6.69E-03 | 2.50E-03 | -3.163831 | -2.1572256 | -1.186258 | 1.186258 | Down-regulated |
| HINFP | 2.70E-05 | 1.41E-06 | -5.390366 | 4.9699322 | -1.186251 | 1.186251 | Down-regulated |
| CNOT11 | 1.01E-03 | 1.43E-04 | -4.078986 | 0.53417696 | -1.186212 | 1.186212 | Down-regulated |
| FAM177A1 | 4.94E-07 | 1.09E-08 | -6.683329 | 9.69856502 | -1.186124 | 1.186124 | Down-regulated |
| MRPS6 | 1.37E-04 | 1.07E-05 | -4.831453 | 3.01631905 | -1.186102 | 1.186102 | Down-regulated |
| SLC7A1 | 1.94E-03 | 3.47E-04 | -3.807012 | -0.30873096 | -1.186001 | 1.186001 | Down-regulated |
| SLC9A3R1 | 5.91E-04 | 7.02E-05 | -4.290184 | 1.21023168 | -1.185717 | 1.185717 | Down-regulated |
| SPECC1L | 2.06E-04 | 1.81E-05 | -4.682746 | 2.51059733 | -1.185637 | 1.185637 | Down-regulated |
| RNPC3 | 2.48E-04 | 2.29E-05 | -4.615328 | 2.28356594 | -1.185635 | 1.185635 | Down-regulated |
| NAB1 | 8.68E-05 | 5.91E-06 | -4.996816 | 3.58615176 | -1.184882 | 1.184882 | Down-regulated |
| HK1 | 1.15E-03 | 1.68E-04 | -4.029118 | 0.37720101 | -1.184708 | 1.184708 | Down-regulated |
| SNORD35B | 1.13E-04 | 8.32E-06 | -4.901332 | 3.25619664 | -1.184414 | 1.184414 | Down-regulated |
| TMEM154 | 2.28E-03 | 4.40E-04 | -3.73283 | -0.53278261 | -1.184413 | 1.184413 | Down-regulated |
| EGR1 | 3.54E-05 | 1.95E-06 | -5.302226 | 4.65695433 | -1.184388 | 1.184388 | Down-regulated |
| PPP2R3A | 1.09E-04 | 7.91E-06 | -4.915401 | 3.30465939 | -1.184358 | 1.184358 | Down-regulated |
| CDC42EP4 | 4.45E-08 | 6.09E-10 | -7.436163 | 12.51028827 | -1.183696 | 1.183696 | Down-regulated |
| CNPY3 | 1.32E-03 | 2.03E-04 | -3.97231 | 0.19967399 | -1.183681 | 1.183681 | Down-regulated |
| GFOD2 | 9.38E-04 | 1.29E-04 | -4.110183 | 0.63290837 | -1.183098 | 1.183098 | Down-regulated |
| ASNA1 | 5.87E-03 | 1.99E-03 | -3.242081 | -1.94440398 | -1.182704 | 1.182704 | Down-regulated |
| NUP214 | 4.14E-03 | 1.11E-03 | -3.436437 | -1.40070082 | -1.182537 | 1.182537 | Down-regulated |
| PHACTR2 | 4.65E-05 | 2.72E-06 | -5.211203 | 4.33550923 | -1.182426 | 1.182426 | Down-regulated |
| PANK3 | 1.63E-03 | 2.72E-04 | -3.882606 | -0.07776378 | -1.181266 | 1.181266 | Down-regulated |
| PSMD9 | 3.26E-03 | 7.51E-04 | -3.562852 | -1.03602917 | -1.181051 | 1.181051 | Down-regulated |
| LIAS | 4.91E-06 | 1.73E-07 | -5.954264 | 7.00607652 | -1.181046 | 1.181046 | Down-regulated |
| CCDC167 | 1.45E-04 | 1.14E-05 | -4.812919 | 2.95292947 | -1.181021 | 1.181021 | Down-regulated |
| PXMP4 | 4.76E-07 | 1.04E-08 | -6.695383 | 9.74343669 | -1.180713 | 1.180713 | Down-regulated |
| NQO1 | 4.04E-05 | 2.28E-06 | -5.259107 | 4.50444721 | -1.180689 | 1.180689 | Down-regulated |
| RPS14 | 4.62E-05 | 2.69E-06 | -5.213618 | 4.34401212 | -1.17942 | 1.17942 | Down-regulated |
| EXOC4 | 2.82E-04 | 2.67E-05 | -4.570804 | 2.13443469 | -1.179216 | 1.179216 | Down-regulated |
| MAGOH | 4.00E-05 | 2.26E-06 | -5.262029 | 4.51476852 | -1.179112 | 1.179112 | Down-regulated |
| MB21D1 | 1.80E-04 | 1.52E-05 | -4.731242 | 2.67478826 | -1.179081 | 1.179081 | Down-regulated |
| AOC4P | 1.26E-04 | 9.59E-06 | -4.861455 | 3.11914105 | -1.178776 | 1.178776 | Down-regulated |
| C9orf72 | 1.09E-04 | 7.97E-06 | -4.913326 | 3.29750769 | -1.178458 | 1.178458 | Down-regulated |
| ARFGEF1 | 1.15E-05 | 4.87E-07 | -5.677641 | 6.00059164 | -1.178324 | 1.178324 | Down-regulated |
| WDR44 | 1.59E-05 | 7.28E-07 | -5.569417 | 5.61053379 | -1.178284 | 1.178284 | Down-regulated |
| ECT2L | 5.10E-04 | 5.75E-05 | -4.348797 | 1.40092952 | -1.178197 | 1.178197 | Down-regulated |
| BAD | 1.20E-03 | 1.79E-04 | -4.010139 | 0.31773581 | -1.178161 | 1.178161 | Down-regulated |
| MRPS31 | 1.98E-07 | 3.63E-09 | -6.970063 | 10.76775644 | -1.178051 | 1.178051 | Down-regulated |
| SLC25A10 | 9.76E-06 | 4.03E-07 | -5.728648 | 6.18511722 | -1.177982 | 1.177982 | Down-regulated |
| MAPK6 | 1.70E-04 | 1.41E-05 | -4.753137 | 2.7491532 | -1.177922 | 1.177922 | Down-regulated |
| SCFD1 | 1.26E-02 | 7.49E-03 | -2.773507 | -3.16269798 | -1.177852 | 1.177852 | Down-regulated |
| RPP40 | 1.33E-06 | 3.70E-08 | -6.361979 | 8.50586328 | -1.17776 | 1.17776 | Down-regulated |
| DTWD1 | 6.65E-09 | 6.07E-11 | -8.039086 | 14.76176377 | -1.177687 | 1.177687 | Down-regulated |
| ILKAP | 2.76E-04 | 2.60E-05 | -4.578668 | 2.16072877 | -1.177375 | 1.177375 | Down-regulated |
| BUB3 | 7.58E-06 | 2.94E-07 | -5.812523 | 6.48943942 | -1.177269 | 1.177269 | Down-regulated |
| APOL2 | 9.13E-04 | 1.24E-04 | -4.121572 | 0.66905252 | -1.176789 | 1.176789 | Down-regulated |
| OSGEP | 2.42E-03 | 4.82E-04 | -3.704408 | -0.61792569 | -1.176583 | 1.176583 | Down-regulated |
| EDNRB | 1.67E-04 | 1.38E-05 | -4.758552 | 2.76756664 | -1.176504 | 1.176504 | Down-regulated |
| CLP1 | 3.79E-06 | 1.28E-07 | -6.034046 | 7.29806718 | -1.176402 | 1.176402 | Down-regulated |
| FAM129B | 3.12E-03 | 7.05E-04 | -3.583445 | -0.97583211 | -1.176297 | 1.176297 | Down-regulated |
| METTL26 | 1.07E-02 | 5.70E-03 | -2.87363 | -2.91404522 | -1.176113 | 1.176113 | Down-regulated |
| LEMD3 | 2.26E-05 | 1.13E-06 | -5.450937 | 5.18594449 | -1.17582 | 1.17582 | Down-regulated |
| WDR5B | 4.03E-04 | 4.25E-05 | -4.436943 | 1.69008765 | -1.17525 | 1.17525 | Down-regulated |
| C1QB | 6.94E-07 | 1.64E-08 | -6.575254 | 9.2966444 | -1.175222 | 1.175222 | Down-regulated |
| UBE2C | 4.84E-03 | 1.43E-03 | -3.351472 | -1.64097977 | -1.175212 | 1.175212 | Down-regulated |
| RNF144B | 6.24E-05 | 3.94E-06 | -5.108794 | 3.97614196 | -1.174684 | 1.174684 | Down-regulated |
| PUDP | 3.64E-04 | 3.75E-05 | -4.473102 | 1.80950795 | -1.174514 | 1.174514 | Down-regulated |
| STK38L | 1.47E-04 | 1.16E-05 | -4.808371 | 2.93738928 | -1.17448 | 1.17448 | Down-regulated |
| FAM188A | 1.36E-06 | 3.79E-08 | -6.355658 | 8.48248217 | -1.174332 | 1.174332 | Down-regulated |
| NCK1 | 2.47E-05 | 1.26E-06 | -5.420908 | 5.07876296 | -1.174078 | 1.174078 | Down-regulated |
| CHMP4B | 5.04E-04 | 5.65E-05 | -4.353795 | 1.41724805 | -1.174008 | 1.174008 | Down-regulated |
| SLX1A | 4.37E-04 | 4.73E-05 | -4.405529 | 1.58671456 | -1.173967 | 1.173967 | Down-regulated |
| MAP3K7 | 5.85E-04 | 6.91E-05 | -4.294599 | 1.22454957 | -1.173661 | 1.173661 | Down-regulated |
| ZNF557 | 7.72E-03 | 3.24E-03 | -3.074632 | -2.39538705 | -1.173598 | 1.173598 | Down-regulated |
| PDE1B | 2.55E-05 | 1.31E-06 | -5.410548 | 5.04182573 | -1.173165 | 1.173165 | Down-regulated |
| OSBPL11 | 6.02E-04 | 7.19E-05 | -4.282967 | 1.18683874 | -1.173136 | 1.173136 | Down-regulated |
| CNDP2 | 8.69E-05 | 5.93E-06 | -4.995895 | 3.58295757 | -1.173035 | 1.173035 | Down-regulated |
| APOO | 4.53E-06 | 1.57E-07 | -5.979382 | 7.09791662 | -1.172944 | 1.172944 | Down-regulated |
| KLHL36 | 1.11E-03 | 1.61E-04 | -4.042257 | 0.41845669 | -1.172625 | 1.172625 | Down-regulated |
| BASP1 | 6.13E-05 | 3.85E-06 | -5.115413 | 3.99929476 | -1.172526 | 1.172526 | Down-regulated |
| PKM | 2.35E-03 | 4.60E-04 | -3.719048 | -0.57411966 | -1.172502 | 1.172502 | Down-regulated |
| REM2 | 1.18E-03 | 1.76E-04 | -4.015581 | 0.33477196 | -1.172053 | 1.172053 | Down-regulated |
| ABCB6 | 6.13E-05 | 3.85E-06 | -5.115363 | 3.99911981 | -1.171945 | 1.171945 | Down-regulated |
| INSL3 | 3.61E-08 | 4.79E-10 | -7.498776 | 12.74444326 | -1.171921 | 1.171921 | Down-regulated |
| TCTEX1D2 | 1.67E-04 | 1.38E-05 | -4.758795 | 2.76839515 | -1.171538 | 1.171538 | Down-regulated |
| RSL24D1 | 8.95E-05 | 6.17E-06 | -4.984619 | 3.54386954 | -1.171288 | 1.171288 | Down-regulated |
| LGALS9B | 3.55E-06 | 1.18E-07 | -6.055702 | 7.377462 | -1.171278 | 1.171278 | Down-regulated |
| CSE1L | 1.10E-05 | 4.60E-07 | -5.692725 | 6.05511591 | -1.170972 | 1.170972 | Down-regulated |
| ZNF155 | 1.09E-04 | 7.93E-06 | -4.914531 | 3.30166016 | -1.1707 | 1.1707 | Down-regulated |
| LRRC29 | 2.35E-05 | 1.19E-06 | -5.436993 | 5.13615346 | -1.170593 | 1.170593 | Down-regulated |
| DPY19L1 | 1.64E-04 | 1.34E-05 | -4.766573 | 2.79485859 | -1.170424 | 1.170424 | Down-regulated |
| COMTD1 | 4.64E-04 | 5.09E-05 | -4.384043 | 1.51621365 | -1.170146 | 1.170146 | Down-regulated |
| DNAJC15 | 1.02E-05 | 4.22E-07 | -5.716083 | 6.13962122 | -1.169929 | 1.169929 | Down-regulated |
| F5 | 1.29E-05 | 5.61E-07 | -5.639595 | 5.86323565 | -1.16986 | 1.16986 | Down-regulated |
| PHIP | 1.39E-04 | 1.09E-05 | -4.826431 | 2.99913421 | -1.169671 | 1.169671 | Down-regulated |
| TRIM4 | 6.08E-05 | 3.81E-06 | -5.118432 | 4.0098562 | -1.169608 | 1.169608 | Down-regulated |
| XRCC5 | 3.67E-07 | 7.62E-09 | -6.776361 | 10.045071 | -1.169556 | 1.169556 | Down-regulated |
| CARD16 | 9.63E-06 | 3.94E-07 | -5.73416 | 6.2050803 | -1.16948 | 1.16948 | Down-regulated |
| N4BP2 | 6.84E-04 | 8.51E-05 | -4.233204 | 1.02609538 | -1.169296 | 1.169296 | Down-regulated |
| RORA | 6.66E-04 | 8.17E-05 | -4.24514 | 1.06456484 | -1.169148 | 1.169148 | Down-regulated |
| RGMA | 3.13E-03 | 7.09E-04 | -3.581628 | -0.98115207 | -1.168697 | 1.168697 | Down-regulated |
| S1PR3 | 6.28E-06 | 2.32E-07 | -5.875682 | 6.71929926 | -1.16863 | 1.16863 | Down-regulated |
| SPTY2D1 | 9.63E-06 | 3.95E-07 | -5.733985 | 6.20444923 | -1.167991 | 1.167991 | Down-regulated |
| ALPK1 | 5.00E-06 | 1.77E-07 | -5.948524 | 6.9850989 | -1.167991 | 1.167991 | Down-regulated |
| ALG5 | 7.10E-03 | 2.79E-03 | -3.126748 | -2.25681771 | -1.167707 | 1.167707 | Down-regulated |
| TMA7 | 1.49E-04 | 1.19E-05 | -4.80104 | 2.91235332 | -1.167678 | 1.167678 | Down-regulated |
| FAM153A | 2.12E-05 | 1.04E-06 | -5.473881 | 5.26796077 | -1.167364 | 1.167364 | Down-regulated |
| GRK5 | 1.14E-06 | 3.04E-08 | -6.413482 | 8.69649957 | -1.16732 | 1.16732 | Down-regulated |
| SLC37A3 | 1.21E-05 | 5.19E-07 | -5.660584 | 5.93898176 | -1.167283 | 1.167283 | Down-regulated |
| TBC1D13 | 4.51E-03 | 1.27E-03 | -3.390794 | -1.5302698 | -1.167052 | 1.167052 | Down-regulated |
| EIF4A3 | 1.99E-05 | 9.68E-07 | -5.492626 | 5.33504001 | -1.166449 | 1.166449 | Down-regulated |
| SGMS1 | 1.17E-05 | 4.98E-07 | -5.67171 | 5.97916464 | -1.16633 | 1.16633 | Down-regulated |
| MZB1 | 1.32E-05 | 5.79E-07 | -5.630927 | 5.83197816 | -1.166096 | 1.166096 | Down-regulated |
| ARNT | 6.79E-04 | 8.43E-05 | -4.23616 | 1.03561766 | -1.165963 | 1.165963 | Down-regulated |
| LILRB2 | 1.59E-07 | 2.79E-09 | -7.038911 | 11.02492693 | -1.16572 | 1.16572 | Down-regulated |
| CASC1 | 6.20E-03 | 2.19E-03 | -3.209463 | -2.03355187 | -1.165465 | 1.165465 | Down-regulated |
| SUMF1 | 3.09E-04 | 3.03E-05 | -4.535022 | 2.0150619 | -1.165296 | 1.165296 | Down-regulated |
| MED20 | 6.07E-04 | 7.27E-05 | -4.279847 | 1.17673366 | -1.165277 | 1.165277 | Down-regulated |
| PTPMT1 | 3.57E-04 | 3.66E-05 | -4.480333 | 1.83344579 | -1.165254 | 1.165254 | Down-regulated |
| PCNA | 1.97E-03 | 3.55E-04 | -3.800403 | -0.32879707 | -1.16488 | 1.16488 | Down-regulated |
| SNX13 | 2.36E-03 | 4.62E-04 | -3.717369 | -0.57914843 | -1.164274 | 1.164274 | Down-regulated |
| VSIG4 | 5.50E-03 | 1.78E-03 | -3.279287 | -1.84196591 | -1.164035 | 1.164035 | Down-regulated |
| TOR1B | 6.37E-07 | 1.48E-08 | -6.602826 | 9.39911153 | -1.163884 | 1.163884 | Down-regulated |
| HINT2 | 1.03E-03 | 1.46E-04 | -4.072799 | 0.51464411 | -1.163863 | 1.163863 | Down-regulated |
| UFD1L | 7.48E-04 | 9.55E-05 | -4.199093 | 0.91646804 | -1.163614 | 1.163614 | Down-regulated |
| ECHDC1 | 2.36E-03 | 4.62E-04 | -3.717232 | -0.57955711 | -1.163399 | 1.163399 | Down-regulated |
| SCARNA18 | 1.11E-03 | 1.60E-04 | -4.043525 | 0.42244204 | -1.163274 | 1.163274 | Down-regulated |
| SUN2 | 1.97E-05 | 9.55E-07 | -5.496084 | 5.34742291 | -1.163243 | 1.163243 | Down-regulated |
| C4orf32 | 6.43E-06 | 2.39E-07 | -5.86809 | 6.69163916 | -1.163207 | 1.163207 | Down-regulated |
| FRYL | 5.80E-04 | 6.83E-05 | -4.298141 | 1.23604327 | -1.162656 | 1.162656 | Down-regulated |
| NHLRC2 | 1.13E-04 | 8.38E-06 | -4.899107 | 3.2485373 | -1.162364 | 1.162364 | Down-regulated |
| FBXL6 | 6.63E-07 | 1.55E-08 | -6.589984 | 9.35137847 | -1.161896 | 1.161896 | Down-regulated |
| NFKBID | 1.01E-03 | 1.42E-04 | -4.079426 | 0.53556417 | -1.161797 | 1.161797 | Down-regulated |
| FEN1 | 4.10E-05 | 2.33E-06 | -5.253151 | 4.48341516 | -1.161786 | 1.161786 | Down-regulated |
| SAPCD2 | 9.24E-05 | 6.44E-06 | -4.972802 | 3.50294037 | -1.161759 | 1.161759 | Down-regulated |
| BIRC6 | 1.91E-05 | 9.20E-07 | -5.506243 | 5.38381258 | -1.16103 | 1.16103 | Down-regulated |
| TSPY26P | 6.68E-04 | 8.23E-05 | -4.243239 | 1.05843608 | -1.160561 | 1.160561 | Down-regulated |
| RPS27A | 7.07E-05 | 4.60E-06 | -5.066242 | 3.82757057 | -1.160463 | 1.160463 | Down-regulated |
| RSRC2 | 7.65E-06 | 2.98E-07 | -5.809281 | 6.47765754 | -1.160395 | 1.160395 | Down-regulated |
| ZFAS1 | 7.73E-04 | 9.95E-05 | -4.186878 | 0.87732461 | -1.160312 | 1.160312 | Down-regulated |
| PLIN5 | 4.09E-04 | 4.33E-05 | -4.431353 | 1.67166754 | -1.160202 | 1.160202 | Down-regulated |
| RPL23AP7 | 3.34E-04 | 3.35E-05 | -4.506104 | 1.91890022 | -1.159965 | 1.159965 | Down-regulated |
| SUB1 | 9.94E-07 | 2.58E-08 | -6.456829 | 8.85711603 | -1.159863 | 1.159863 | Down-regulated |
| C2orf69 | 5.53E-05 | 3.38E-06 | -5.151119 | 4.12436291 | -1.159699 | 1.159699 | Down-regulated |
| CHMP1A | 5.36E-05 | 3.26E-06 | -5.161182 | 4.15966586 | -1.159582 | 1.159582 | Down-regulated |
| PTRH2 | 1.56E-03 | 2.56E-04 | -3.900724 | -0.02201668 | -1.159455 | 1.159455 | Down-regulated |
| SPG21 | 1.22E-03 | 1.83E-04 | -4.003897 | 0.29821301 | -1.159366 | 1.159366 | Down-regulated |
| DISC1 | 3.52E-04 | 3.59E-05 | -4.485768 | 1.85144961 | -1.158889 | 1.158889 | Down-regulated |
| DUSP18 | 2.25E-05 | 1.12E-06 | -5.452264 | 5.19068476 | -1.158803 | 1.158803 | Down-regulated |
| APLP2 | 2.80E-03 | 5.97E-04 | -3.636473 | -0.81983504 | -1.158778 | 1.158778 | Down-regulated |
| ARHGEF7 | 6.71E-03 | 2.52E-03 | -3.161288 | -2.16408108 | -1.158742 | 1.158742 | Down-regulated |
| ATP5A1 | 1.16E-05 | 4.96E-07 | -5.672731 | 5.98285124 | -1.158661 | 1.158661 | Down-regulated |
| TCAF2 | 1.18E-06 | 3.19E-08 | -6.400898 | 8.64989886 | -1.158357 | 1.158357 | Down-regulated |
| PIGH | 7.89E-08 | 1.22E-09 | -7.254649 | 11.83142355 | -1.158172 | 1.158172 | Down-regulated |
| RAD51D | 9.49E-04 | 1.31E-04 | -4.105254 | 0.61728166 | -1.158036 | 1.158036 | Down-regulated |
| KCNK12 | 3.79E-03 | 9.63E-04 | -3.482677 | -1.26828996 | -1.157567 | 1.157567 | Down-regulated |
| BBS4 | 2.24E-05 | 1.11E-06 | -5.454938 | 5.20023937 | -1.157157 | 1.157157 | Down-regulated |
| COL17A1 | 2.04E-03 | 3.72E-04 | -3.785664 | -0.37347493 | -1.156774 | 1.156774 | Down-regulated |
| ACOX3 | 3.32E-07 | 6.80E-09 | -6.806426 | 10.15714168 | -1.156117 | 1.156117 | Down-regulated |
| SLC25A34 | 2.82E-06 | 8.87E-08 | -6.13116 | 7.65454168 | -1.155905 | 1.155905 | Down-regulated |
| PPP1R14B | 5.92E-05 | 3.66E-06 | -5.129014 | 4.04689913 | -1.155889 | 1.155889 | Down-regulated |
| METTL21A | 5.50E-03 | 1.78E-03 | -3.279903 | -1.84026561 | -1.155826 | 1.155826 | Down-regulated |
| CCNA2 | 5.07E-04 | 5.71E-05 | -4.350961 | 1.40799472 | -1.155624 | 1.155624 | Down-regulated |
| MKKS | 7.26E-06 | 2.79E-07 | -5.827042 | 6.54222792 | -1.155543 | 1.155543 | Down-regulated |
| CAPN2 | 5.40E-05 | 3.29E-06 | -5.15876 | 4.15116961 | -1.155521 | 1.155521 | Down-regulated |
| ANKRD54 | 2.28E-06 | 6.98E-08 | -6.19467 | 7.88824476 | -1.155492 | 1.155492 | Down-regulated |
| PLK4 | 3.40E-04 | 3.43E-05 | -4.499145 | 1.89580138 | -1.154769 | 1.154769 | Down-regulated |
| DAB2 | 3.72E-03 | 9.32E-04 | -3.493322 | -1.23764877 | -1.154757 | 1.154757 | Down-regulated |
| MAPK14 | 3.40E-06 | 1.12E-07 | -6.070253 | 7.43084223 | -1.154471 | 1.154471 | Down-regulated |
| LSM5 | 4.31E-05 | 2.47E-06 | -5.236917 | 4.4261278 | -1.154286 | 1.154286 | Down-regulated |
| SLC10A3 | 5.08E-06 | 1.80E-07 | -5.944002 | 6.96857804 | -1.153977 | 1.153977 | Down-regulated |
| CAV2 | 9.01E-05 | 6.23E-06 | -4.98193 | 3.53455273 | -1.15397 | 1.15397 | Down-regulated |
| SURF1 | 9.90E-05 | 7.04E-06 | -4.947974 | 3.41706519 | -1.153857 | 1.153857 | Down-regulated |
| GID8 | 1.73E-05 | 8.08E-07 | -5.541254 | 5.50936953 | -1.153636 | 1.153636 | Down-regulated |
| KCTD21 | 2.26E-03 | 4.33E-04 | -3.737835 | -0.51774881 | -1.153027 | 1.153027 | Down-regulated |
| CPQ | 3.25E-03 | 7.51E-04 | -3.56298 | -1.03565454 | -1.15264 | 1.15264 | Down-regulated |
| WDR7 | 2.78E-03 | 5.91E-04 | -3.639645 | -0.81045872 | -1.15247 | 1.15247 | Down-regulated |
| MYB | 2.59E-03 | 5.31E-04 | -3.673678 | -0.70954144 | -1.152327 | 1.152327 | Down-regulated |
| CLEC4D | 6.07E-06 | 2.23E-07 | -5.886976 | 6.76046361 | -1.151809 | 1.151809 | Down-regulated |
| GNGT2 | 1.74E-07 | 3.14E-09 | -7.008193 | 10.91016712 | -1.151564 | 1.151564 | Down-regulated |
| BCKDHB | 2.08E-04 | 1.83E-05 | -4.67862 | 2.4966605 | -1.151446 | 1.151446 | Down-regulated |
| DNAJB14 | 7.30E-03 | 2.93E-03 | -3.109238 | -2.30355922 | -1.151195 | 1.151195 | Down-regulated |
| RPL18 | 6.51E-04 | 7.93E-05 | -4.253938 | 1.09295483 | -1.151084 | 1.151084 | Down-regulated |
| MTMR4 | 7.01E-04 | 8.79E-05 | -4.223543 | 0.99500173 | -1.150985 | 1.150985 | Down-regulated |
| DHX16 | 1.23E-03 | 1.85E-04 | -4.001152 | 0.28963294 | -1.150094 | 1.150094 | Down-regulated |
| ABHD17B | 3.10E-04 | 3.05E-05 | -4.532839 | 2.00779094 | -1.149754 | 1.149754 | Down-regulated |
| HNRNPC | 4.52E-05 | 2.63E-06 | -5.220637 | 4.36873568 | -1.14975 | 1.14975 | Down-regulated |
| DPH5 | 5.76E-06 | 2.10E-07 | -5.903138 | 6.81940004 | -1.149681 | 1.149681 | Down-regulated |
| ARMC2 | 1.88E-03 | 3.33E-04 | -3.820236 | -0.26851903 | -1.149666 | 1.149666 | Down-regulated |
| TIFA | 8.21E-04 | 1.08E-04 | -4.16307 | 0.80119665 | -1.149585 | 1.149585 | Down-regulated |
| NIT2 | 5.80E-03 | 1.95E-03 | -3.249192 | -1.9248877 | -1.149425 | 1.149425 | Down-regulated |
| CDC20 | 8.47E-04 | 1.12E-04 | -4.150665 | 0.76162031 | -1.148789 | 1.148789 | Down-regulated |
| SESN2 | 4.96E-09 | 4.20E-11 | -8.136072 | 15.12281224 | -1.148503 | 1.148503 | Down-regulated |
| LOC646938 | 1.17E-02 | 6.71E-03 | -2.814073 | -3.06275077 | -1.148401 | 1.148401 | Down-regulated |
| IFT20 | 4.62E-04 | 5.07E-05 | -4.385192 | 1.51997847 | -1.148245 | 1.148245 | Down-regulated |
| SLC22A15 | 5.09E-05 | 3.04E-06 | -5.180371 | 4.22705477 | -1.147279 | 1.147279 | Down-regulated |
| DIP2A | 1.82E-04 | 1.55E-05 | -4.725861 | 2.65653626 | -1.147167 | 1.147167 | Down-regulated |
| USP33 | 7.31E-06 | 2.81E-07 | -5.824796 | 6.5340624 | -1.146913 | 1.146913 | Down-regulated |
| CDKN1B | 2.30E-03 | 4.45E-04 | -3.729281 | -0.5434365 | -1.146846 | 1.146846 | Down-regulated |
| CHCHD4 | 5.18E-04 | 5.87E-05 | -4.342395 | 1.38003534 | -1.146464 | 1.146464 | Down-regulated |
| SQRDL | 5.22E-04 | 5.93E-05 | -4.339822 | 1.37164325 | -1.146124 | 1.146124 | Down-regulated |
| ALDH4A1 | 6.94E-04 | 8.67E-05 | -4.227647 | 1.00820774 | -1.145788 | 1.145788 | Down-regulated |
| S100PBP | 7.79E-03 | 3.29E-03 | -3.069079 | -2.4100568 | -1.145667 | 1.145667 | Down-regulated |
| MSRB3 | 2.87E-04 | 2.74E-05 | -4.563856 | 2.11122275 | -1.144925 | 1.144925 | Down-regulated |
| MCM8 | 5.48E-06 | 1.98E-07 | -5.918762 | 6.87640918 | -1.144918 | 1.144918 | Down-regulated |
| PDHA1 | 1.40E-03 | 2.21E-04 | -3.946827 | 0.12049574 | -1.144892 | 1.144892 | Down-regulated |
| BAG6 | 2.10E-05 | 1.03E-06 | -5.476492 | 5.27730125 | -1.144767 | 1.144767 | Down-regulated |
| IL18R1 | 7.57E-06 | 2.94E-07 | -5.813156 | 6.49174168 | -1.144454 | 1.144454 | Down-regulated |
| COA4 | 2.34E-03 | 4.57E-04 | -3.721234 | -0.5675679 | -1.144194 | 1.144194 | Down-regulated |
| SPPL2A | 7.72E-05 | 5.12E-06 | -5.036392 | 3.72362108 | -1.144027 | 1.144027 | Down-regulated |
| ARHGEF10L | 5.88E-05 | 3.64E-06 | -5.130939 | 4.05364038 | -1.143983 | 1.143983 | Down-regulated |
| ING2 | 1.75E-07 | 3.16E-09 | -7.006871 | 10.90523152 | -1.143795 | 1.143795 | Down-regulated |
| SPPL3 | 1.88E-06 | 5.51E-08 | -6.257018 | 8.11808581 | -1.143545 | 1.143545 | Down-regulated |
| KHDC1L | 1.45E-03 | 2.31E-04 | -3.933003 | 0.0776635 | -1.143499 | 1.143499 | Down-regulated |
| MT1X | 3.10E-04 | 3.05E-05 | -4.533105 | 2.00867788 | -1.143436 | 1.143436 | Down-regulated |
| PLGRKT | 3.23E-04 | 3.21E-05 | -4.518425 | 1.95983465 | -1.143115 | 1.143115 | Down-regulated |
| EEF1D | 5.21E-06 | 1.86E-07 | -5.935232 | 6.93654224 | -1.14311 | 1.14311 | Down-regulated |
| HCG27 | 1.60E-04 | 1.30E-05 | -4.776645 | 2.82915711 | -1.143091 | 1.143091 | Down-regulated |
| NR3C1 | 2.24E-03 | 4.26E-04 | -3.742708 | -0.50309874 | -1.143006 | 1.143006 | Down-regulated |
| TOMM5 | 5.17E-05 | 3.11E-06 | -5.174337 | 4.20585293 | -1.142697 | 1.142697 | Down-regulated |
| TJP2 | 6.51E-06 | 2.43E-07 | -5.863091 | 6.67342893 | -1.142673 | 1.142673 | Down-regulated |
| CELF6 | 2.66E-07 | 5.26E-09 | -6.873392 | 10.40690504 | -1.142641 | 1.142641 | Down-regulated |
| LCLAT1 | 8.94E-06 | 3.61E-07 | -5.757947 | 6.29129837 | -1.142312 | 1.142312 | Down-regulated |
| SERPINB2 | 1.74E-05 | 8.16E-07 | -5.538726 | 5.50029677 | -1.14229 | 1.14229 | Down-regulated |
| TMEM222 | 1.54E-04 | 1.24E-05 | -4.788696 | 2.87023536 | -1.141943 | 1.141943 | Down-regulated |
| PPP1R15A | 3.37E-04 | 3.39E-05 | -4.501984 | 1.9052241 | -1.141833 | 1.141833 | Down-regulated |
| STX7 | 4.33E-03 | 1.20E-03 | -3.41096 | -1.47316556 | -1.141813 | 1.141813 | Down-regulated |
| UGDH | 1.32E-05 | 5.75E-07 | -5.633076 | 5.83972748 | -1.141749 | 1.141749 | Down-regulated |
| ZBTB44 | 4.09E-05 | 2.33E-06 | -5.253422 | 4.48437144 | -1.141487 | 1.141487 | Down-regulated |
| RCOR3 | 1.74E-03 | 2.97E-04 | -3.855069 | -0.1622026 | -1.141305 | 1.141305 | Down-regulated |
| C11orf71 | 8.06E-04 | 1.05E-04 | -4.169857 | 0.82287549 | -1.141283 | 1.141283 | Down-regulated |
| VWA3A | 5.98E-06 | 2.19E-07 | -5.891704 | 6.777701 | -1.141004 | 1.141004 | Down-regulated |
| TAP1 | 1.27E-03 | 1.93E-04 | -3.987502 | 0.24701397 | -1.140999 | 1.140999 | Down-regulated |
| ZNF330 | 1.48E-04 | 1.18E-05 | -4.802855 | 2.91855092 | -1.140279 | 1.140279 | Down-regulated |
| KBTBD3 | 5.35E-04 | 6.12E-05 | -4.330189 | 1.34024677 | -1.140273 | 1.140273 | Down-regulated |
| ABI1 | 5.48E-05 | 3.35E-06 | -5.153616 | 4.13311968 | -1.140272 | 1.140272 | Down-regulated |
| SERPINA1 | 2.51E-05 | 1.28E-06 | -5.415786 | 5.06049889 | -1.140167 | 1.140167 | Down-regulated |
| KCNMB3 | 5.76E-03 | 1.92E-03 | -3.253642 | -1.91265886 | -1.140015 | 1.140015 | Down-regulated |
| SPATA5L1 | 1.18E-04 | 8.83E-06 | -4.884532 | 3.19840251 | -1.139978 | 1.139978 | Down-regulated |
| CEP192 | 5.48E-03 | 1.77E-03 | -3.281431 | -1.83604027 | -1.139895 | 1.139895 | Down-regulated |
| DESI1 | 4.45E-04 | 4.85E-05 | -4.398471 | 1.56353679 | -1.139772 | 1.139772 | Down-regulated |
| SIGLEC14 | 2.71E-04 | 2.55E-05 | -4.584493 | 2.18021777 | -1.139733 | 1.139733 | Down-regulated |
| COPS5 | 2.51E-03 | 5.08E-04 | -3.687672 | -0.66787778 | -1.139698 | 1.139698 | Down-regulated |
| IRF2BPL | 5.28E-07 | 1.18E-08 | -6.662437 | 9.62081721 | -1.139669 | 1.139669 | Down-regulated |
| CNIH2 | 8.77E-04 | 1.17E-04 | -4.137932 | 0.72106428 | -1.139666 | 1.139666 | Down-regulated |
| ACSL1 | 4.95E-02 | 4.44E-02 | -2.05583 | -4.73697469 | -1.139644 | 1.139644 | Down-regulated |
| KIF11 | 1.14E-05 | 4.81E-07 | -5.680694 | 6.01162353 | -1.139585 | 1.139585 | Down-regulated |
| LOC100129055 | 3.18E-02 | 2.69E-02 | -2.271437 | -4.30444541 | -1.139552 | 1.139552 | Down-regulated |
| CTU1 | 8.18E-04 | 1.07E-04 | -4.164543 | 0.80590062 | -1.139491 | 1.139491 | Down-regulated |
| ZNF180 | 1.66E-05 | 7.70E-07 | -5.554474 | 5.55684114 | -1.139391 | 1.139391 | Down-regulated |
| PRAF2 | 1.01E-06 | 2.65E-08 | -6.450219 | 8.83261378 | -1.139364 | 1.139364 | Down-regulated |
| LATS2 | 1.72E-03 | 2.92E-04 | -3.860926 | -0.14427361 | -1.139104 | 1.139104 | Down-regulated |
| PPP6R3 | 4.93E-03 | 1.48E-03 | -3.341023 | -1.67025422 | -1.13877 | 1.13877 | Down-regulated |
| OSTC | 8.05E-04 | 1.05E-04 | -4.170671 | 0.82547569 | -1.138289 | 1.138289 | Down-regulated |
| AK6 | 1.78E-03 | 3.06E-04 | -3.845721 | -0.19079001 | -1.138074 | 1.138074 | Down-regulated |
| CHMP4A | 2.49E-04 | 2.30E-05 | -4.613992 | 2.27908209 | -1.137992 | 1.137992 | Down-regulated |
| ISY1 | 4.65E-04 | 5.12E-05 | -4.382611 | 1.51152182 | -1.137864 | 1.137864 | Down-regulated |
| ZNF280D | 1.44E-06 | 4.04E-08 | -6.338755 | 8.41997434 | -1.137648 | 1.137648 | Down-regulated |
| ZDHHC17 | 5.65E-06 | 2.04E-07 | -5.910007 | 6.84446101 | -1.137569 | 1.137569 | Down-regulated |
| RPL9 | 6.73E-06 | 2.53E-07 | -5.852711 | 6.63563234 | -1.137533 | 1.137533 | Down-regulated |
| SDHAF3 | 6.38E-05 | 4.05E-06 | -5.101695 | 3.95132681 | -1.137395 | 1.137395 | Down-regulated |
| MED4 | 7.16E-05 | 4.67E-06 | -5.061704 | 3.81175317 | -1.137263 | 1.137263 | Down-regulated |
| BGLAP | 2.55E-04 | 2.36E-05 | -4.606781 | 2.25489061 | -1.136998 | 1.136998 | Down-regulated |
| TCAF1 | 6.83E-03 | 2.59E-03 | -3.151824 | -2.18956146 | -1.136883 | 1.136883 | Down-regulated |
| SMARCAL1 | 9.14E-05 | 6.35E-06 | -4.976723 | 3.51651462 | -1.136832 | 1.136832 | Down-regulated |
| PRRG2 | 2.61E-04 | 2.43E-05 | -4.598517 | 2.22718274 | -1.136124 | 1.136124 | Down-regulated |
| ASNS | 3.81E-03 | 9.70E-04 | -3.48034 | -1.27501017 | -1.136061 | 1.136061 | Down-regulated |
| NCEH1 | 3.88E-03 | 1.00E-03 | -3.470194 | -1.30415085 | -1.135926 | 1.135926 | Down-regulated |
| TRIM73 | 1.75E-04 | 1.47E-05 | -4.74059 | 2.70652029 | -1.135907 | 1.135907 | Down-regulated |
| GNRH1 | 5.06E-05 | 3.02E-06 | -5.182468 | 4.23442206 | -1.1359 | 1.1359 | Down-regulated |
| UBE2Q1 | 6.79E-05 | 4.38E-06 | -5.079662 | 3.87437782 | -1.135641 | 1.135641 | Down-regulated |
| SLC6A13 | 8.72E-03 | 4.00E-03 | -3.000234 | -2.59032213 | -1.13555 | 1.13555 | Down-regulated |
| CHCHD5 | 5.97E-04 | 7.11E-05 | -4.286148 | 1.19714724 | -1.135464 | 1.135464 | Down-regulated |
| PAFAH1B2 | 7.56E-04 | 9.67E-05 | -4.195383 | 0.90457412 | -1.135417 | 1.135417 | Down-regulated |
| TAB1 | 6.90E-03 | 2.64E-03 | -3.145355 | -2.20694793 | -1.135138 | 1.135138 | Down-regulated |
| ORC1 | 2.11E-04 | 1.86E-05 | -4.67406 | 2.48126591 | -1.135024 | 1.135024 | Down-regulated |
| LIMS1 | 3.01E-07 | 6.11E-09 | -6.834271 | 10.26097207 | -1.134811 | 1.134811 | Down-regulated |
| BOK | 7.54E-09 | 7.08E-11 | -7.998762 | 14.61153038 | -1.133954 | 1.133954 | Down-regulated |
| UBR3 | 7.74E-04 | 9.98E-05 | -4.185994 | 0.8744931 | -1.133925 | 1.133925 | Down-regulated |
| TM9SF1 | 1.05E-05 | 4.35E-07 | -5.707966 | 6.11024596 | -1.133908 | 1.133908 | Down-regulated |
| SHC1 | 4.91E-04 | 5.46E-05 | -4.36373 | 1.44971649 | -1.133804 | 1.133804 | Down-regulated |
| SNX10 | 2.38E-03 | 4.68E-04 | -3.713368 | -0.59112737 | -1.133774 | 1.133774 | Down-regulated |
| IGSF6 | 3.81E-05 | 2.13E-06 | -5.277748 | 4.57032863 | -1.133607 | 1.133607 | Down-regulated |
| ADAP2 | 2.97E-04 | 2.87E-05 | -4.550725 | 2.0673956 | -1.133085 | 1.133085 | Down-regulated |
| ATP6V1G1 | 3.47E-03 | 8.31E-04 | -3.530519 | -1.13010093 | -1.133011 | 1.133011 | Down-regulated |
| ATP13A1 | 3.72E-05 | 2.07E-06 | -5.285265 | 4.5969146 | -1.132908 | 1.132908 | Down-regulated |
| ZNF326 | 5.13E-07 | 1.14E-08 | -6.67201 | 9.65643849 | -1.132646 | 1.132646 | Down-regulated |
| RPP25L | 2.88E-05 | 1.52E-06 | -5.369909 | 4.89714561 | -1.132644 | 1.132644 | Down-regulated |
| CHD4 | 2.94E-05 | 1.56E-06 | -5.362466 | 4.87068334 | -1.132231 | 1.132231 | Down-regulated |
| FSCN1 | 2.44E-05 | 1.24E-06 | -5.42554 | 5.09528477 | -1.132131 | 1.132131 | Down-regulated |
| CR1 | 3.06E-04 | 2.98E-05 | -4.539171 | 2.02888023 | -1.131944 | 1.131944 | Down-regulated |
| TRA2B | 1.18E-03 | 1.76E-04 | -4.015547 | 0.33466562 | -1.13182 | 1.13182 | Down-regulated |
| FABP5 | 1.39E-05 | 6.17E-07 | -5.614119 | 5.77139959 | -1.131732 | 1.131732 | Down-regulated |
| TMEM234 | 3.50E-06 | 1.16E-07 | -6.060564 | 7.39529665 | -1.131179 | 1.131179 | Down-regulated |
| PIGV | 4.15E-03 | 1.11E-03 | -3.435373 | -1.40373458 | -1.131086 | 1.131086 | Down-regulated |
| CRTC3 | 1.59E-03 | 2.64E-04 | -3.891839 | -0.04937377 | -1.131085 | 1.131085 | Down-regulated |
| MFSD1 | 6.70E-04 | 8.28E-05 | -4.241339 | 1.05230993 | -1.130955 | 1.130955 | Down-regulated |
| LONRF3 | 1.00E-06 | 2.61E-08 | -6.45414 | 8.84714826 | -1.130917 | 1.130917 | Down-regulated |
| BAZ2A | 3.33E-04 | 3.34E-05 | -4.506953 | 1.92171913 | -1.130847 | 1.130847 | Down-regulated |
| SIGLEC16 | 2.77E-09 | 2.08E-11 | -8.320572 | 15.80835749 | -1.130798 | 1.130798 | Down-regulated |
| GM2A | 1.25E-05 | 5.39E-07 | -5.650289 | 5.90182009 | -1.130645 | 1.130645 | Down-regulated |
| ATXN7L3B | 4.63E-03 | 1.33E-03 | -3.375617 | -1.57310172 | -1.13062 | 1.13062 | Down-regulated |
| ATP6V1H | 4.69E-03 | 1.36E-03 | -3.36851 | -1.59311391 | -1.130488 | 1.130488 | Down-regulated |
| LOC105377348 | 6.38E-04 | 7.75E-05 | -4.261095 | 1.11607346 | -1.129971 | 1.129971 | Down-regulated |
| NOL6 | 1.51E-06 | 4.28E-08 | -6.323404 | 8.36323069 | -1.12989 | 1.12989 | Down-regulated |
| RNF112 | 2.33E-04 | 2.12E-05 | -4.637186 | 2.35701377 | -1.129646 | 1.129646 | Down-regulated |
| NDUFS2 | 1.41E-03 | 2.22E-04 | -3.944236 | 0.11246334 | -1.129584 | 1.129584 | Down-regulated |
| IRF2BP2 | 8.01E-04 | 1.04E-04 | -4.172949 | 0.83275544 | -1.129485 | 1.129485 | Down-regulated |
| EFNA4 | 4.72E-06 | 1.65E-07 | -5.966242 | 7.04986137 | -1.128763 | 1.128763 | Down-regulated |
| HDHD2 | 4.09E-05 | 2.32E-06 | -5.254086 | 4.48671589 | -1.128716 | 1.128716 | Down-regulated |
| LCMT1 | 4.80E-04 | 5.31E-05 | -4.371854 | 1.47629434 | -1.128379 | 1.128379 | Down-regulated |
| TMSB4X | 1.29E-04 | 9.84E-06 | -4.854135 | 3.09403034 | -1.128278 | 1.128278 | Down-regulated |
| SETD4 | 9.75E-04 | 1.36E-04 | -4.094172 | 0.58218697 | -1.127934 | 1.127934 | Down-regulated |
| FAM168A | 7.72E-04 | 9.94E-05 | -4.187224 | 0.87842983 | -1.12784 | 1.12784 | Down-regulated |
| UEVLD | 1.17E-06 | 3.15E-08 | -6.404422 | 8.66294914 | -1.127545 | 1.127545 | Down-regulated |
| HGSNAT | 1.10E-03 | 1.59E-04 | -4.04604 | 0.43035152 | -1.127439 | 1.127439 | Down-regulated |
| PUSL1 | 1.36E-04 | 1.06E-05 | -4.834042 | 3.02518341 | -1.127419 | 1.127419 | Down-regulated |
| ARMT1 | 8.84E-05 | 6.06E-06 | -4.989592 | 3.56110296 | -1.127383 | 1.127383 | Down-regulated |
| AEBP2 | 2.95E-06 | 9.38E-08 | -6.116525 | 7.60075028 | -1.126946 | 1.126946 | Down-regulated |
| SLC35F6 | 1.51E-05 | 6.86E-07 | -5.585394 | 5.66798866 | -1.126898 | 1.126898 | Down-regulated |
| FAHD1 | 2.01E-06 | 5.99E-08 | -6.235165 | 8.0374803 | -1.126536 | 1.126536 | Down-regulated |
| NRDE2 | 9.09E-04 | 1.23E-04 | -4.123608 | 0.67551921 | -1.126503 | 1.126503 | Down-regulated |
| DPEP3 | 1.95E-05 | 9.42E-07 | -5.499832 | 5.36084662 | -1.126383 | 1.126383 | Down-regulated |
| MTF2 | 9.76E-03 | 4.89E-03 | -2.9285 | -2.77499996 | -1.125751 | 1.125751 | Down-regulated |
| HECTD1 | 1.61E-03 | 2.67E-04 | -3.888003 | -0.0611751 | -1.125732 | 1.125732 | Down-regulated |
| IFT52 | 9.56E-05 | 6.75E-06 | -4.959542 | 3.45705665 | -1.125687 | 1.125687 | Down-regulated |
| PTGES2 | 3.62E-04 | 3.73E-05 | -4.474635 | 1.81458324 | -1.125569 | 1.125569 | Down-regulated |
| GSTTP2 | 1.24E-04 | 9.37E-06 | -4.868097 | 3.14193676 | -1.125336 | 1.125336 | Down-regulated |
| MCFD2 | 8.31E-06 | 3.29E-07 | -5.782534 | 6.38050601 | -1.124989 | 1.124989 | Down-regulated |
| ZNF644 | 3.01E-05 | 1.61E-06 | -5.354301 | 4.84166827 | -1.124815 | 1.124815 | Down-regulated |
| CYB5D2 | 1.23E-03 | 1.86E-04 | -3.99853 | 0.28144091 | -1.124801 | 1.124801 | Down-regulated |
| ZNF366 | 5.67E-07 | 1.28E-08 | -6.639688 | 9.53618183 | -1.124369 | 1.124369 | Down-regulated |
| MRPL14 | 1.77E-04 | 1.49E-05 | -4.737558 | 2.69622658 | -1.123987 | 1.123987 | Down-regulated |
| SBF1 | 1.44E-02 | 9.28E-03 | -2.693487 | -3.35660759 | -1.123745 | 1.123745 | Down-regulated |
| KDM6A | 1.03E-03 | 1.46E-04 | -4.07125 | 0.50975519 | -1.123591 | 1.123591 | Down-regulated |
| SLC2A4RG | 1.81E-06 | 5.28E-08 | -6.268172 | 8.15924518 | -1.123505 | 1.123505 | Down-regulated |
| SERPINB8 | 2.51E-02 | 2.03E-02 | -2.388798 | -4.05395258 | -1.123401 | 1.123401 | Down-regulated |
| ARL16 | 2.96E-03 | 6.49E-04 | -3.609716 | -0.89872657 | -1.123387 | 1.123387 | Down-regulated |
| TXNL1 | 8.69E-05 | 5.93E-06 | -4.995707 | 3.58230602 | -1.123358 | 1.123358 | Down-regulated |
| PATE2 | 2.19E-07 | 4.12E-09 | -6.937117 | 10.64474055 | -1.12328 | 1.12328 | Down-regulated |
| LOC102724002 | 5.86E-04 | 6.92E-05 | -4.294149 | 1.22309074 | -1.123095 | 1.123095 | Down-regulated |
| CLCN3 | 1.77E-04 | 1.49E-05 | -4.736325 | 2.69203976 | -1.123027 | 1.123027 | Down-regulated |
| MOB4 | 1.34E-03 | 2.08E-04 | -3.965178 | 0.17748764 | -1.122765 | 1.122765 | Down-regulated |
| PLIN2 | 2.97E-04 | 2.87E-05 | -4.549956 | 2.06482969 | -1.122631 | 1.122631 | Down-regulated |
| NPY5R | 1.36E-03 | 2.13E-04 | -3.958188 | 0.15576193 | -1.122119 | 1.122119 | Down-regulated |
| TMPO-AS1 | 8.21E-04 | 1.08E-04 | -4.163185 | 0.8015631 | -1.121903 | 1.121903 | Down-regulated |
| SUSD2 | 1.59E-03 | 2.64E-04 | -3.89196 | -0.0490016 | -1.121896 | 1.121896 | Down-regulated |
| LOC101926963 | 5.02E-03 | 1.52E-03 | -3.331854 | -1.69589284 | -1.121809 | 1.121809 | Down-regulated |
| SLC38A2 | 7.61E-03 | 3.16E-03 | -3.082895 | -2.37352888 | -1.121725 | 1.121725 | Down-regulated |
| TTC1 | 1.29E-06 | 3.55E-08 | -6.373193 | 8.54735319 | -1.121718 | 1.121718 | Down-regulated |
| MED9 | 1.04E-03 | 1.48E-04 | -4.067878 | 0.49912004 | -1.121489 | 1.121489 | Down-regulated |
| SSFA2 | 4.39E-05 | 2.53E-06 | -5.230459 | 4.4033549 | -1.121458 | 1.121458 | Down-regulated |
| PRMT9 | 1.29E-04 | 9.83E-06 | -4.854367 | 3.09482426 | -1.121382 | 1.121382 | Down-regulated |
| PPM1B | 5.41E-03 | 1.73E-03 | -3.289818 | -1.81282782 | -1.121331 | 1.121331 | Down-regulated |
| PSMD13 | 4.06E-04 | 4.29E-05 | -4.433838 | 1.67985626 | -1.120934 | 1.120934 | Down-regulated |
| C1RL | 5.72E-04 | 6.70E-05 | -4.303947 | 1.25489211 | -1.1209 | 1.1209 | Down-regulated |
| HIST2H4B | 1.25E-03 | 1.89E-04 | -3.994654 | 0.26933475 | -1.120684 | 1.120684 | Down-regulated |
| MTMR10 | 1.10E-04 | 8.03E-06 | -4.911316 | 3.29058438 | -1.120545 | 1.120545 | Down-regulated |
| TMPRSS15 | 2.91E-05 | 1.54E-06 | -5.366637 | 4.88551314 | -1.120308 | 1.120308 | Down-regulated |
| C1QTNF6 | 1.80E-05 | 8.49E-07 | -5.52811 | 5.4622054 | -1.119961 | 1.119961 | Down-regulated |
| USO1 | 9.52E-06 | 3.89E-07 | -5.737639 | 6.2176869 | -1.119761 | 1.119761 | Down-regulated |
| HAX1 | 1.42E-04 | 1.12E-05 | -4.818779 | 2.97296199 | -1.119479 | 1.119479 | Down-regulated |
| MRPL39 | 5.38E-05 | 3.27E-06 | -5.16011 | 4.15590453 | -1.119047 | 1.119047 | Down-regulated |
| CYB5B | 3.02E-06 | 9.69E-08 | -6.107872 | 7.56895799 | -1.118561 | 1.118561 | Down-regulated |
| MTHFD1L | 5.14E-06 | 1.83E-07 | -5.939396 | 6.95175318 | -1.118187 | 1.118187 | Down-regulated |
| ZAK | 4.10E-05 | 2.34E-06 | -5.252722 | 4.48189954 | -1.118116 | 1.118116 | Down-regulated |
| DOCK7 | 5.82E-04 | 6.86E-05 | -4.296774 | 1.23160679 | -1.118113 | 1.118113 | Down-regulated |
| ORC4 | 1.06E-04 | 7.66E-06 | -4.924447 | 3.3358483 | -1.11776 | 1.11776 | Down-regulated |
| TIMM8B | 7.34E-06 | 2.82E-07 | -5.823637 | 6.52984691 | -1.117232 | 1.117232 | Down-regulated |
| GTF2H1 | 1.13E-05 | 4.77E-07 | -5.683312 | 6.02108821 | -1.11722 | 1.11722 | Down-regulated |
| MAN1B1 | 1.08E-03 | 1.54E-04 | -4.055001 | 0.45854322 | -1.117051 | 1.117051 | Down-regulated |
| STK26 | 6.67E-05 | 4.27E-06 | -5.086998 | 3.89998543 | -1.11696 | 1.11696 | Down-regulated |
| POPDC2 | 1.50E-05 | 6.80E-07 | -5.587926 | 5.67709799 | -1.116888 | 1.116888 | Down-regulated |
| RAB34 | 7.28E-03 | 2.92E-03 | -3.111048 | -2.29873529 | -1.116643 | 1.116643 | Down-regulated |
| EIF3E | 2.44E-03 | 4.88E-04 | -3.700467 | -0.6297001 | -1.1159 | 1.1159 | Down-regulated |
| CELF1 | 1.26E-04 | 9.58E-06 | -4.861629 | 3.11973783 | -1.115641 | 1.115641 | Down-regulated |
| SLC31A1 | 8.30E-04 | 1.09E-04 | -4.158504 | 0.78662045 | -1.115619 | 1.115619 | Down-regulated |
| WLS | 4.83E-05 | 2.85E-06 | -5.19827 | 4.28998723 | -1.115438 | 1.115438 | Down-regulated |
| SNORA59A | 7.03E-04 | 8.82E-05 | -4.222785 | 0.99256254 | -1.115417 | 1.115417 | Down-regulated |
| FAM26F | 5.28E-05 | 3.20E-06 | -5.166711 | 4.17907364 | -1.114831 | 1.114831 | Down-regulated |
| LOC220729 | 2.82E-06 | 8.92E-08 | -6.129812 | 7.64958501 | -1.114805 | 1.114805 | Down-regulated |
| LINC01451 | 1.23E-04 | 9.31E-06 | -4.869839 | 3.14792038 | -1.114563 | 1.114563 | Down-regulated |
| SAMD9 | 1.93E-06 | 5.67E-08 | -6.249424 | 8.09006968 | -1.114433 | 1.114433 | Down-regulated |
| CHML | 9.21E-06 | 3.74E-07 | -5.748079 | 6.2555214 | -1.114411 | 1.114411 | Down-regulated |
| CADPS | 1.12E-04 | 8.22E-06 | -4.904585 | 3.2673979 | -1.114316 | 1.114316 | Down-regulated |
| UHRF1BP1 | 2.68E-04 | 2.51E-05 | -4.58885 | 2.19480322 | -1.114205 | 1.114205 | Down-regulated |
| RACK1 | 6.49E-06 | 2.42E-07 | -5.864709 | 6.67932387 | -1.113725 | 1.113725 | Down-regulated |
| GDAP2 | 5.25E-05 | 3.17E-06 | -5.168534 | 4.18547611 | -1.113571 | 1.113571 | Down-regulated |
| HMGCS1 | 4.21E-05 | 2.42E-06 | -5.243514 | 4.44939917 | -1.113431 | 1.113431 | Down-regulated |
| PGBD3 | 5.11E-04 | 5.77E-05 | -4.347861 | 1.39787121 | -1.113157 | 1.113157 | Down-regulated |
| DDX52 | 2.19E-06 | 6.64E-08 | -6.207981 | 7.93728145 | -1.112679 | 1.112679 | Down-regulated |
| CD53 | 3.50E-05 | 1.92E-06 | -5.306715 | 4.67285206 | -1.112401 | 1.112401 | Down-regulated |
| TMEM191B | 4.51E-03 | 1.28E-03 | -3.390503 | -1.53109214 | -1.112062 | 1.112062 | Down-regulated |
| PSMD5 | 6.94E-03 | 2.66E-03 | -3.142425 | -2.21481375 | -1.111837 | 1.111837 | Down-regulated |
| LYRM4 | 4.12E-03 | 1.10E-03 | -3.4397 | -1.39139543 | -1.111256 | 1.111256 | Down-regulated |
| NDUFB11 | 1.57E-04 | 1.27E-05 | -4.78239 | 2.84873397 | -1.111234 | 1.111234 | Down-regulated |
| XRCC1 | 1.82E-05 | 8.65E-07 | -5.522893 | 5.44349568 | -1.110919 | 1.110919 | Down-regulated |
| F8 | 5.97E-05 | 3.71E-06 | -5.125675 | 4.03520905 | -1.110417 | 1.110417 | Down-regulated |
| TOR1AIP1 | 1.75E-03 | 3.01E-04 | -3.851562 | -0.17293294 | -1.109817 | 1.109817 | Down-regulated |
| TCF20 | 8.93E-07 | 2.26E-08 | -6.491413 | 8.98536842 | -1.109668 | 1.109668 | Down-regulated |
| BMS1P5 | 3.66E-04 | 3.77E-05 | -4.47159 | 1.80450717 | -1.109619 | 1.109619 | Down-regulated |
| TTC26 | 4.71E-03 | 1.37E-03 | -3.366424 | -1.59898518 | -1.109585 | 1.109585 | Down-regulated |
| CLDN9 | 2.61E-03 | 5.38E-04 | -3.669258 | -0.72268062 | -1.109536 | 1.109536 | Down-regulated |
| FAS | 9.32E-05 | 6.51E-06 | -4.969795 | 3.49252844 | -1.109519 | 1.109519 | Down-regulated |
| DLD | 4.42E-04 | 4.81E-05 | -4.400993 | 1.57181816 | -1.109395 | 1.109395 | Down-regulated |
| BTNL10 | 1.46E-05 | 6.55E-07 | -5.597833 | 5.71275233 | -1.109387 | 1.109387 | Down-regulated |
| BMP8B | 1.35E-04 | 1.04E-05 | -4.837474 | 3.03693356 | -1.109206 | 1.109206 | Down-regulated |
| SCO1 | 8.32E-03 | 3.69E-03 | -3.029039 | -2.51525395 | -1.109174 | 1.109174 | Down-regulated |
| LOC100128398 | 5.46E-04 | 6.31E-05 | -4.321599 | 1.31227902 | -1.108968 | 1.108968 | Down-regulated |
| HCK | 2.16E-04 | 1.91E-05 | -4.666606 | 2.45611573 | -1.108833 | 1.108833 | Down-regulated |
| MIEF1 | 1.62E-08 | 1.77E-10 | -7.759239 | 13.71789953 | -1.108818 | 1.108818 | Down-regulated |
| SLC43A3 | 2.43E-04 | 2.23E-05 | -4.622996 | 2.30931457 | -1.108761 | 1.108761 | Down-regulated |
| VTI1B | 1.22E-04 | 9.17E-06 | -4.874059 | 3.16241307 | -1.108754 | 1.108754 | Down-regulated |
| CPNE3 | 2.44E-05 | 1.24E-06 | -5.42582 | 5.09628377 | -1.108655 | 1.108655 | Down-regulated |
| LINC00152 | 1.35E-05 | 5.93E-07 | -5.62456 | 5.80902585 | -1.108358 | 1.108358 | Down-regulated |
| ZFYVE26 | 6.63E-03 | 2.46E-03 | -3.169424 | -2.14213225 | -1.108325 | 1.108325 | Down-regulated |
| TAGLN | 1.04E-02 | 5.45E-03 | -2.889632 | -2.87369616 | -1.108245 | 1.108245 | Down-regulated |
| ARHGAP24 | 1.50E-03 | 2.42E-04 | -3.918557 | 0.03299402 | -1.10747 | 1.10747 | Down-regulated |
| CMTM2 | 9.44E-06 | 3.86E-07 | -5.740259 | 6.22717752 | -1.107269 | 1.107269 | Down-regulated |
| DNAJC21 | 2.70E-06 | 8.41E-08 | -6.145467 | 7.70714836 | -1.107175 | 1.107175 | Down-regulated |
| GTF3C6 | 4.08E-06 | 1.39E-07 | -6.011929 | 7.21704085 | -1.106899 | 1.106899 | Down-regulated |
| METRNL | 9.69E-03 | 4.84E-03 | -2.932608 | -2.76451356 | -1.105994 | 1.105994 | Down-regulated |
| ABHD5 | 5.32E-04 | 6.08E-05 | -4.332473 | 1.34768833 | -1.105778 | 1.105778 | Down-regulated |
| ZNF688 | 1.22E-06 | 3.32E-08 | -6.390599 | 8.61177164 | -1.105715 | 1.105715 | Down-regulated |
| TCF12 | 7.80E-07 | 1.91E-08 | -6.535055 | 9.14733584 | -1.105575 | 1.105575 | Down-regulated |
| UBR2 | 1.78E-03 | 3.08E-04 | -3.843742 | -0.19683716 | -1.105362 | 1.105362 | Down-regulated |
| NCDN | 9.89E-09 | 9.84E-11 | -7.912517 | 14.28998934 | -1.105088 | 1.105088 | Down-regulated |
| GPT2 | 2.53E-07 | 4.92E-09 | -6.890967 | 10.47248218 | -1.104813 | 1.104813 | Down-regulated |
| SENP2 | 8.67E-06 | 3.47E-07 | -5.768239 | 6.32862752 | -1.104661 | 1.104661 | Down-regulated |
| MOB2 | 4.92E-08 | 6.88E-10 | -7.404377 | 12.39141139 | -1.104129 | 1.104129 | Down-regulated |
| CINP | 7.76E-06 | 3.02E-07 | -5.805227 | 6.46292606 | -1.103496 | 1.103496 | Down-regulated |
| ITM2B | 3.74E-03 | 9.37E-04 | -3.491482 | -1.24294846 | -1.103341 | 1.103341 | Down-regulated |
| KIF2C | 1.26E-04 | 9.62E-06 | -4.860641 | 3.11634589 | -1.103067 | 1.103067 | Down-regulated |
| CALU | 1.79E-03 | 3.09E-04 | -3.842792 | -0.19973813 | -1.102728 | 1.102728 | Down-regulated |
| ST3GAL4 | 2.75E-03 | 5.81E-04 | -3.645339 | -0.79361303 | -1.102556 | 1.102556 | Down-regulated |
| RABIF | 1.70E-05 | 7.92E-07 | -5.5468 | 5.52928182 | -1.102275 | 1.102275 | Down-regulated |
| TICAM1 | 2.85E-05 | 1.50E-06 | -5.373297 | 4.90919509 | -1.101836 | 1.101836 | Down-regulated |
| GCLC | 8.73E-05 | 5.97E-06 | -4.993744 | 3.57549991 | -1.101822 | 1.101822 | Down-regulated |
| FAM3A | 1.73E-04 | 1.45E-05 | -4.744971 | 2.72140221 | -1.101784 | 1.101784 | Down-regulated |
| ALDOB | 3.00E-04 | 2.91E-05 | -4.546507 | 2.05332915 | -1.101647 | 1.101647 | Down-regulated |
| FDFT1 | 1.43E-05 | 6.40E-07 | -5.604052 | 5.73514346 | -1.101598 | 1.101598 | Down-regulated |
| TMEM11 | 1.69E-03 | 2.85E-04 | -3.867874 | -0.12298078 | -1.101352 | 1.101352 | Down-regulated |
| ATP2C1 | 1.59E-04 | 1.29E-05 | -4.77889 | 2.83680839 | -1.101244 | 1.101244 | Down-regulated |
| LOC105370613 | 1.47E-03 | 2.36E-04 | -3.926456 | 0.05740987 | -1.101116 | 1.101116 | Down-regulated |
| GREM1 | 1.36E-04 | 1.06E-05 | -4.8342 | 3.02572248 | -1.101053 | 1.101053 | Down-regulated |
| TRIM7 | 6.51E-04 | 7.95E-05 | -4.253223 | 1.09064829 | -1.101003 | 1.101003 | Down-regulated |
| LOC107133515 | 2.01E-03 | 3.67E-04 | -3.789902 | -0.36064025 | -1.100806 | 1.100806 | Down-regulated |
| TAF5 | 7.59E-03 | 3.14E-03 | -3.085015 | -2.36791175 | -1.100236 | 1.100236 | Down-regulated |
| RAP1GDS1 | 1.76E-02 | 1.25E-02 | -2.57895 | -3.62650104 | -1.100207 | 1.100207 | Down-regulated |
| WHSC1L1 | 2.65E-05 | 1.37E-06 | -5.397415 | 4.99503361 | -1.099994 | 1.099994 | Down-regulated |
| TNPO1 | 2.04E-04 | 1.78E-05 | -4.687367 | 2.52620979 | -1.099489 | 1.099489 | Down-regulated |
| DOLPP1 | 7.44E-03 | 3.03E-03 | -3.098108 | -2.33317339 | -1.099291 | 1.099291 | Down-regulated |
| TFDP1 | 1.55E-04 | 1.25E-05 | -4.787095 | 2.86477695 | -1.098942 | 1.098942 | Down-regulated |
| EIF3K | 5.17E-05 | 3.11E-06 | -5.174344 | 4.20587753 | -1.098939 | 1.098939 | Down-regulated |
| B4GALNT4 | 1.17E-03 | 1.72E-04 | -4.021995 | 0.354866 | -1.098546 | 1.098546 | Down-regulated |
| LAMTOR3 | 7.76E-06 | 3.03E-07 | -5.804946 | 6.46190376 | -1.098489 | 1.098489 | Down-regulated |
| STX3 | 3.07E-03 | 6.86E-04 | -3.592005 | -0.95074723 | -1.098302 | 1.098302 | Down-regulated |
| SPDYE1 | 1.25E-02 | 7.41E-03 | -2.777671 | -3.15248756 | -1.098137 | 1.098137 | Down-regulated |
| MED7 | 2.02E-04 | 1.76E-05 | -4.690384 | 2.53640766 | -1.09748 | 1.09748 | Down-regulated |
| EGFEM1P | 7.99E-03 | 3.44E-03 | -3.053413 | -2.4513334 | -1.097376 | 1.097376 | Down-regulated |
| SYAP1 | 2.35E-04 | 2.14E-05 | -4.63465 | 2.34848425 | -1.096938 | 1.096938 | Down-regulated |
| HIST3H3 | 3.48E-03 | 8.35E-04 | -3.528804 | -1.13507285 | -1.096601 | 1.096601 | Down-regulated |
| UBE2K | 1.04E-04 | 7.52E-06 | -4.929342 | 3.35273397 | -1.096225 | 1.096225 | Down-regulated |
| FNBP1 | 5.95E-04 | 7.07E-05 | -4.287946 | 1.2029754 | -1.096069 | 1.096069 | Down-regulated |
| C12orf4 | 5.46E-04 | 6.31E-05 | -4.321416 | 1.31168257 | -1.095613 | 1.095613 | Down-regulated |
| RRAS | 2.86E-05 | 1.51E-06 | -5.372594 | 4.90669549 | -1.095341 | 1.095341 | Down-regulated |
| MBIP | 3.84E-05 | 2.15E-06 | -5.275492 | 4.56234963 | -1.095046 | 1.095046 | Down-regulated |
| WDR18 | 2.19E-04 | 1.96E-05 | -4.659665 | 2.43270931 | -1.094401 | 1.094401 | Down-regulated |
| FKBP4 | 7.58E-06 | 2.94E-07 | -5.812812 | 6.49049213 | -1.094124 | 1.094124 | Down-regulated |
| RHOG | 3.12E-06 | 1.01E-07 | -6.097804 | 7.53197908 | -1.093942 | 1.093942 | Down-regulated |
| MC1R | 2.12E-03 | 3.94E-04 | -3.767733 | -0.4276903 | -1.093642 | 1.093642 | Down-regulated |
| ZNF716 | 4.26E-03 | 1.16E-03 | -3.42057 | -1.44587349 | -1.093631 | 1.093631 | Down-regulated |
| C2 | 2.02E-04 | 1.76E-05 | -4.689635 | 2.53387544 | -1.093547 | 1.093547 | Down-regulated |
| ATAD2B | 8.90E-06 | 3.58E-07 | -5.759811 | 6.29805603 | -1.093424 | 1.093424 | Down-regulated |
| PRPF38A | 2.71E-04 | 2.55E-05 | -4.584494 | 2.18022167 | -1.093206 | 1.093206 | Down-regulated |
| PARP9 | 1.93E-06 | 5.71E-08 | -6.247774 | 8.08398319 | -1.09315 | 1.09315 | Down-regulated |
| PLXNA4 | 3.13E-06 | 1.01E-07 | -6.096839 | 7.52843582 | -1.093136 | 1.093136 | Down-regulated |
| KLHL7 | 5.63E-07 | 1.27E-08 | -6.642447 | 9.54644764 | -1.093064 | 1.093064 | Down-regulated |
| EIF5A2 | 3.11E-03 | 7.01E-04 | -3.585251 | -0.97054393 | -1.093038 | 1.093038 | Down-regulated |
| RAD51C | 5.36E-03 | 1.70E-03 | -3.295514 | -1.79704271 | -1.092993 | 1.092993 | Down-regulated |
| HES6 | 8.32E-05 | 5.61E-06 | -5.011228 | 3.63616439 | -1.092988 | 1.092988 | Down-regulated |
| CCL3 | 8.72E-04 | 1.16E-04 | -4.140304 | 0.72861442 | -1.092982 | 1.092982 | Down-regulated |
| FAM208A | 2.34E-03 | 4.56E-04 | -3.721665 | -0.56627541 | -1.092866 | 1.092866 | Down-regulated |
| CEP135 | 2.70E-04 | 2.53E-05 | -4.586678 | 2.18752904 | -1.09272 | 1.09272 | Down-regulated |
| RAB33B | 3.89E-04 | 4.08E-05 | -4.448517 | 1.72826309 | -1.092359 | 1.092359 | Down-regulated |
| LRP3 | 1.39E-05 | 6.14E-07 | -5.615237 | 5.77542831 | -1.092153 | 1.092153 | Down-regulated |
| LRRC25 | 6.63E-03 | 2.46E-03 | -3.169014 | -2.1432403 | -1.092061 | 1.092061 | Down-regulated |
| NDUFA11 | 8.99E-07 | 2.28E-08 | -6.488753 | 8.97549949 | -1.091966 | 1.091966 | Down-regulated |
| TRIP4 | 2.17E-07 | 4.06E-09 | -6.940818 | 10.65855787 | -1.091551 | 1.091551 | Down-regulated |
| ATP6V1C1 | 3.22E-03 | 7.40E-04 | -3.567942 | -1.02116994 | -1.091406 | 1.091406 | Down-regulated |
| PADI2 | 8.37E-06 | 3.32E-07 | -5.779983 | 6.37124745 | -1.091318 | 1.091318 | Down-regulated |
| HSPA4 | 6.64E-03 | 2.47E-03 | -3.168383 | -2.1449427 | -1.090791 | 1.090791 | Down-regulated |
| ARL2 | 8.06E-06 | 3.17E-07 | -5.792639 | 6.41719768 | -1.090736 | 1.090736 | Down-regulated |
| NBR1 | 2.04E-03 | 3.74E-04 | -3.784072 | -0.37829614 | -1.090555 | 1.090555 | Down-regulated |
| MICB | 6.85E-05 | 4.43E-06 | -5.07646 | 3.86320653 | -1.090165 | 1.090165 | Down-regulated |
| JOSD1 | 1.64E-03 | 2.74E-04 | -3.8803 | -0.08484762 | -1.08937 | 1.08937 | Down-regulated |
| FAM129A | 3.53E-05 | 1.94E-06 | -5.303322 | 4.66083444 | -1.089023 | 1.089023 | Down-regulated |
| GPR35 | 1.21E-07 | 2.02E-09 | -7.122705 | 11.33808388 | -1.088954 | 1.088954 | Down-regulated |
| AP2A1 | 4.94E-05 | 2.93E-06 | -5.190679 | 4.26328749 | -1.08886 | 1.08886 | Down-regulated |
| CDKN2AIPNL | 1.81E-05 | 8.53E-07 | -5.526601 | 5.45679362 | -1.088794 | 1.088794 | Down-regulated |
| POLR2L | 3.46E-04 | 3.51E-05 | -4.491933 | 1.87188031 | -1.088764 | 1.088764 | Down-regulated |
| LINC00173 | 1.77E-04 | 1.49E-05 | -4.737413 | 2.69573356 | -1.088596 | 1.088596 | Down-regulated |
| LMBRD2 | 2.41E-06 | 7.43E-08 | -6.17811 | 7.8272668 | -1.088579 | 1.088579 | Down-regulated |
| TMEM176B | 1.78E-04 | 1.50E-05 | -4.734429 | 2.68560482 | -1.088342 | 1.088342 | Down-regulated |
| RENBP | 2.17E-04 | 1.93E-05 | -4.663339 | 2.44509756 | -1.08833 | 1.08833 | Down-regulated |
| EIF4E3 | 2.13E-04 | 1.88E-05 | -4.67057 | 2.46948869 | -1.088231 | 1.088231 | Down-regulated |
| BCL2L12 | 6.30E-04 | 7.63E-05 | -4.265607 | 1.1306547 | -1.088106 | 1.088106 | Down-regulated |
| TMEM70 | 6.87E-04 | 8.56E-05 | -4.231342 | 1.02010043 | -1.088081 | 1.088081 | Down-regulated |
| BCDIN3D | 3.55E-03 | 8.62E-04 | -3.51846 | -1.16504465 | -1.087883 | 1.087883 | Down-regulated |
| NFAT5 | 3.34E-03 | 7.84E-04 | -3.549129 | -1.07601961 | -1.087656 | 1.087656 | Down-regulated |
| LOC105377443 | 2.84E-04 | 2.70E-05 | -4.567906 | 2.12475093 | -1.087386 | 1.087386 | Down-regulated |
| MTCH2 | 3.15E-03 | 7.13E-04 | -3.579651 | -0.98694071 | -1.086873 | 1.086873 | Down-regulated |
| NLRP8 | 8.32E-06 | 3.30E-07 | -5.782105 | 6.37894897 | -1.086605 | 1.086605 | Down-regulated |
| RHOC | 2.81E-03 | 6.01E-04 | -3.634267 | -0.82635199 | -1.086419 | 1.086419 | Down-regulated |
| PDSS1 | 2.51E-04 | 2.31E-05 | -4.612414 | 2.27378599 | -1.085677 | 1.085677 | Down-regulated |
| SLF2 | 2.20E-03 | 4.15E-04 | -3.751163 | -0.47765587 | -1.085528 | 1.085528 | Down-regulated |
| ZNF765 | 2.20E-04 | 1.97E-05 | -4.658455 | 2.42863217 | -1.085384 | 1.085384 | Down-regulated |
| STAB1 | 2.97E-04 | 2.87E-05 | -4.549746 | 2.064131 | -1.085286 | 1.085286 | Down-regulated |
| CASP9 | 1.60E-04 | 1.31E-05 | -4.774528 | 2.82194736 | -1.084997 | 1.084997 | Down-regulated |
| LAMC1 | 8.08E-06 | 3.18E-07 | -5.79181 | 6.41418655 | -1.084563 | 1.084563 | Down-regulated |
| GPBP1L1 | 1.45E-03 | 2.30E-04 | -3.933575 | 0.07943362 | -1.084525 | 1.084525 | Down-regulated |
| TOPBP1 | 5.06E-04 | 5.68E-05 | -4.352451 | 1.41285725 | -1.084437 | 1.084437 | Down-regulated |
| CTAGE5 | 7.62E-06 | 2.96E-07 | -5.810715 | 6.48287078 | -1.084429 | 1.084429 | Down-regulated |
| GPER1 | 5.23E-05 | 3.16E-06 | -5.169639 | 4.18935385 | -1.084164 | 1.084164 | Down-regulated |
| MEAF6 | 1.65E-06 | 4.74E-08 | -6.29658 | 8.26412581 | -1.083968 | 1.083968 | Down-regulated |
| ATG101 | 2.49E-07 | 4.80E-09 | -6.897494 | 10.49683942 | -1.083965 | 1.083965 | Down-regulated |
| CLN5 | 5.65E-05 | 3.48E-06 | -5.143626 | 4.09809202 | -1.083837 | 1.083837 | Down-regulated |
| FCF1 | 2.18E-03 | 4.12E-04 | -3.753756 | -0.4698441 | -1.08364 | 1.08364 | Down-regulated |
| RXRG | 8.38E-05 | 5.66E-06 | -5.008821 | 3.62781031 | -1.083434 | 1.083434 | Down-regulated |
| MRVI1-AS1 | 2.30E-05 | 1.15E-06 | -5.445689 | 5.16720211 | -1.083214 | 1.083214 | Down-regulated |
| ZFX | 1.71E-03 | 2.91E-04 | -3.861641 | -0.14208234 | -1.083069 | 1.083069 | Down-regulated |
| NOCT | 9.40E-03 | 4.58E-03 | -2.952137 | -2.71450737 | -1.082608 | 1.082608 | Down-regulated |
| HDHD3 | 7.31E-05 | 4.81E-06 | -5.053909 | 3.78459529 | -1.082558 | 1.082558 | Down-regulated |
| SLC36A4 | 5.00E-03 | 1.51E-03 | -3.333459 | -1.69140792 | -1.081968 | 1.081968 | Down-regulated |
| WDR46 | 2.12E-05 | 1.04E-06 | -5.472969 | 5.2646994 | -1.081782 | 1.081782 | Down-regulated |
| TMEM126A | 8.38E-05 | 5.65E-06 | -5.008939 | 3.62821965 | -1.081631 | 1.081631 | Down-regulated |
| FAM96A | 4.53E-04 | 4.95E-05 | -4.392585 | 1.54422197 | -1.081429 | 1.081429 | Down-regulated |
| PITPNB | 2.83E-04 | 2.68E-05 | -4.569921 | 2.13148498 | -1.081067 | 1.081067 | Down-regulated |
| WDR24 | 3.32E-04 | 3.33E-05 | -4.507683 | 1.9241435 | -1.080942 | 1.080942 | Down-regulated |
| DYNLT1 | 1.02E-04 | 7.28E-06 | -4.938587 | 3.38464238 | -1.080708 | 1.080708 | Down-regulated |
| UTP18 | 1.95E-03 | 3.48E-04 | -3.805864 | -0.31221713 | -1.080463 | 1.080463 | Down-regulated |
| KIAA0586 | 3.57E-03 | 8.70E-04 | -3.515651 | -1.1731747 | -1.080433 | 1.080433 | Down-regulated |
| MCTS1 | 3.26E-04 | 3.24E-05 | -4.515291 | 1.94941912 | -1.080078 | 1.080078 | Down-regulated |
| RUNX1 | 5.96E-04 | 7.09E-05 | -4.287292 | 1.20085471 | -1.080001 | 1.080001 | Down-regulated |
| MST1 | 3.32E-05 | 1.80E-06 | -5.323768 | 4.73329591 | -1.079705 | 1.079705 | Down-regulated |
| FBXO5 | 9.81E-05 | 6.97E-06 | -4.950782 | 3.42676864 | -1.07968 | 1.07968 | Down-regulated |
| MYPOP | 1.60E-04 | 1.31E-05 | -4.774599 | 2.82219023 | -1.079622 | 1.079622 | Down-regulated |
| EPRS | 2.42E-03 | 4.82E-04 | -3.704238 | -0.61843472 | -1.079585 | 1.079585 | Down-regulated |
| DCAF11 | 1.05E-06 | 2.75E-08 | -6.439819 | 8.79407111 | -1.079573 | 1.079573 | Down-regulated |
| SMIM12 | 6.19E-05 | 3.90E-06 | -5.111517 | 3.98566744 | -1.07939 | 1.07939 | Down-regulated |
| TCTA | 3.35E-06 | 1.09E-07 | -6.075648 | 7.45063858 | -1.07914 | 1.07914 | Down-regulated |
| TCEANC2 | 1.29E-02 | 7.78E-03 | -2.759193 | -3.19770137 | -1.079082 | 1.079082 | Down-regulated |
| STK36 | 1.46E-06 | 4.11E-08 | -6.334272 | 8.40340349 | -1.078678 | 1.078678 | Down-regulated |
| KNTC1 | 4.09E-04 | 4.35E-05 | -4.430376 | 1.6684497 | -1.078399 | 1.078399 | Down-regulated |
| WDR74 | 1.06E-04 | 7.68E-06 | -4.92381 | 3.33364947 | -1.078353 | 1.078353 | Down-regulated |
| JAK2 | 4.06E-04 | 4.29E-05 | -4.434065 | 1.68060414 | -1.077907 | 1.077907 | Down-regulated |
| TMEM175 | 4.66E-03 | 1.35E-03 | -3.371915 | -1.5835297 | -1.077733 | 1.077733 | Down-regulated |
| ATG4A | 8.61E-05 | 5.85E-06 | -4.99966 | 3.59601746 | -1.077697 | 1.077697 | Down-regulated |
| HUS1 | 5.06E-05 | 3.02E-06 | -5.182401 | 4.23418924 | -1.077406 | 1.077406 | Down-regulated |
| ZKSCAN7 | 1.20E-02 | 6.92E-03 | -2.802821 | -3.0905837 | -1.077079 | 1.077079 | Down-regulated |
| GART | 5.04E-05 | 3.00E-06 | -5.183986 | 4.23975928 | -1.076996 | 1.076996 | Down-regulated |
| ACOX2 | 1.52E-03 | 2.47E-04 | -3.912077 | 0.01298952 | -1.076966 | 1.076966 | Down-regulated |
| VPS39 | 7.23E-05 | 4.73E-06 | -5.058356 | 3.80008735 | -1.076676 | 1.076676 | Down-regulated |
| C11orf57 | 7.42E-04 | 9.45E-05 | -4.202086 | 0.92606859 | -1.076636 | 1.076636 | Down-regulated |
| SNAPC1 | 5.17E-05 | 3.10E-06 | -5.175029 | 4.20828699 | -1.076594 | 1.076594 | Down-regulated |
| C18orf54 | 3.64E-04 | 3.75E-05 | -4.473238 | 1.80995926 | -1.076569 | 1.076569 | Down-regulated |
| POLQ | 1.36E-05 | 6.02E-07 | -5.620781 | 5.79540617 | -1.076308 | 1.076308 | Down-regulated |
| ST8SIA4 | 2.24E-02 | 1.75E-02 | -2.447512 | -3.924774 | -1.07611 | 1.07611 | Down-regulated |
| CDK12 | 4.63E-04 | 5.08E-05 | -4.384599 | 1.5180349 | -1.075987 | 1.075987 | Down-regulated |
| IQGAP2 | 2.16E-03 | 4.05E-04 | -3.759186 | -0.45348124 | -1.075958 | 1.075958 | Down-regulated |
| DSE | 1.45E-03 | 2.30E-04 | -3.933398 | 0.07888792 | -1.075902 | 1.075902 | Down-regulated |
| HSD17B11 | 1.06E-03 | 1.51E-04 | -4.06175 | 0.47980067 | -1.075845 | 1.075845 | Down-regulated |
| MARCO | 1.43E-03 | 2.26E-04 | -3.939053 | 0.09639834 | -1.075821 | 1.075821 | Down-regulated |
| WAC | 3.16E-05 | 1.70E-06 | -5.339037 | 4.78746726 | -1.07564 | 1.07564 | Down-regulated |
| PLA2G15 | 2.90E-05 | 1.53E-06 | -5.367529 | 4.88868297 | -1.075495 | 1.075495 | Down-regulated |
| ALDH9A1 | 2.74E-03 | 5.78E-04 | -3.646659 | -0.7897068 | -1.07538 | 1.07538 | Down-regulated |
| PIK3C3 | 5.71E-03 | 1.90E-03 | -3.258135 | -1.90030008 | -1.075351 | 1.075351 | Down-regulated |
| FICD | 1.01E-02 | 5.16E-03 | -2.909401 | -2.82361816 | -1.075322 | 1.075322 | Down-regulated |
| JUN | 1.25E-05 | 5.42E-07 | -5.64893 | 5.89691722 | -1.074943 | 1.074943 | Down-regulated |
| SELT | 1.57E-06 | 4.50E-08 | -6.31023 | 8.31455036 | -1.074803 | 1.074803 | Down-regulated |
| TMEM185A | 6.51E-04 | 7.96E-05 | -4.253136 | 1.09036721 | -1.0747 | 1.0747 | Down-regulated |
| TCL1B | 4.55E-04 | 4.99E-05 | -4.390365 | 1.53694104 | -1.074568 | 1.074568 | Down-regulated |
| ARIH1 | 1.75E-03 | 2.99E-04 | -3.853392 | -0.16733536 | -1.07449 | 1.07449 | Down-regulated |
| RFXANK | 2.00E-04 | 1.73E-05 | -4.694349 | 2.54981608 | -1.074471 | 1.074471 | Down-regulated |
| SEC23IP | 3.29E-05 | 1.79E-06 | -5.326224 | 4.74200512 | -1.074397 | 1.074397 | Down-regulated |
| FAM104B | 4.12E-05 | 2.35E-06 | -5.250944 | 4.47562236 | -1.073986 | 1.073986 | Down-regulated |
| SIRT5 | 9.10E-04 | 1.23E-04 | -4.122812 | 0.67298936 | -1.073504 | 1.073504 | Down-regulated |
| INTU | 5.69E-06 | 2.06E-07 | -5.907235 | 6.83434766 | -1.072702 | 1.072702 | Down-regulated |
| PAQR6 | 1.87E-04 | 1.60E-05 | -4.717513 | 2.62823253 | -1.072534 | 1.072534 | Down-regulated |
| ATL1 | 3.76E-04 | 3.90E-05 | -4.461769 | 1.77203158 | -1.072424 | 1.072424 | Down-regulated |
| RNASE3 | 3.23E-04 | 3.21E-05 | -4.518208 | 1.95911479 | -1.072323 | 1.072323 | Down-regulated |
| FAM102A | 3.35E-03 | 7.90E-04 | -3.546755 | -1.0829308 | -1.071883 | 1.071883 | Down-regulated |
| ATF6 | 6.09E-03 | 2.12E-03 | -3.220621 | -2.00312563 | -1.071784 | 1.071784 | Down-regulated |
| SPC25 | 3.32E-03 | 7.76E-04 | -3.552546 | -1.06607197 | -1.071666 | 1.071666 | Down-regulated |
| PDCD10 | 9.34E-05 | 6.53E-06 | -4.969062 | 3.48999377 | -1.071231 | 1.071231 | Down-regulated |
| CTBP2 | 2.40E-03 | 4.75E-04 | -3.708769 | -0.6048878 | -1.070942 | 1.070942 | Down-regulated |
| KMT5A | 6.63E-05 | 4.24E-06 | -5.088842 | 3.90642472 | -1.070888 | 1.070888 | Down-regulated |
| POLDIP2 | 2.81E-08 | 3.50E-10 | -7.581242 | 13.05278172 | -1.070676 | 1.070676 | Down-regulated |
| FAM209A | 1.84E-04 | 1.56E-05 | -4.723349 | 2.6480154 | -1.070637 | 1.070637 | Down-regulated |
| HBB | 4.51E-08 | 6.22E-10 | -7.430767 | 12.49011006 | -1.070313 | 1.070313 | Down-regulated |
| RALBP1 | 3.76E-04 | 3.91E-05 | -4.460817 | 1.76888411 | -1.069941 | 1.069941 | Down-regulated |
| SLC16A4 | 2.39E-04 | 2.18E-05 | -4.629321 | 2.33056869 | -1.069893 | 1.069893 | Down-regulated |
| DHX15 | 3.48E-04 | 3.54E-05 | -4.48967 | 1.86438033 | -1.069858 | 1.069858 | Down-regulated |
| LRRC8D | 5.78E-05 | 3.57E-06 | -5.135994 | 4.07134701 | -1.069777 | 1.069777 | Down-regulated |
| GLTSCR1L | 7.09E-04 | 8.92E-05 | -4.219219 | 0.9810944 | -1.069667 | 1.069667 | Down-regulated |
| CUEDC1 | 3.76E-03 | 9.45E-04 | -3.48864 | -1.25113183 | -1.069537 | 1.069537 | Down-regulated |
| DEK | 6.97E-05 | 4.52E-06 | -5.071056 | 3.84435541 | -1.069125 | 1.069125 | Down-regulated |
| YIPF4 | 1.55E-03 | 2.54E-04 | -3.903949 | -0.01207977 | -1.068917 | 1.068917 | Down-regulated |
| C19orf70 | 9.57E-05 | 6.76E-06 | -4.959242 | 3.45602041 | -1.068815 | 1.068815 | Down-regulated |
| CDKN2B | 4.40E-03 | 1.22E-03 | -3.404334 | -1.49195129 | -1.068738 | 1.068738 | Down-regulated |
| C17orf58 | 2.30E-03 | 4.44E-04 | -3.730167 | -0.54077809 | -1.068565 | 1.068565 | Down-regulated |
| RPS18 | 4.29E-03 | 1.18E-03 | -3.416036 | -1.45875555 | -1.06847 | 1.06847 | Down-regulated |
| BRPF1 | 7.80E-06 | 3.04E-07 | -5.803487 | 6.45660361 | -1.068339 | 1.068339 | Down-regulated |
| STAG3 | 1.17E-04 | 8.69E-06 | -4.8891 | 3.21410863 | -1.068141 | 1.068141 | Down-regulated |
| MFSD14A | 1.47E-07 | 2.54E-09 | -7.06389 | 11.11826481 | -1.068116 | 1.068116 | Down-regulated |
| MCTP1 | 2.29E-03 | 4.41E-04 | -3.732361 | -0.53419081 | -1.068104 | 1.068104 | Down-regulated |
| UBC | 3.71E-04 | 3.84E-05 | -4.466186 | 1.78663393 | -1.067811 | 1.067811 | Down-regulated |
| MRPL16 | 1.67E-04 | 1.38E-05 | -4.759362 | 2.77032374 | -1.067671 | 1.067671 | Down-regulated |
| LEAP2 | 4.75E-06 | 1.66E-07 | -5.964314 | 7.04281292 | -1.067625 | 1.067625 | Down-regulated |
| SCARNA3 | 5.68E-06 | 2.06E-07 | -5.908081 | 6.83743461 | -1.067359 | 1.067359 | Down-regulated |
| THUMPD1 | 7.51E-04 | 9.60E-05 | -4.197612 | 0.91172078 | -1.067173 | 1.067173 | Down-regulated |
| C16orf70 | 4.57E-05 | 2.66E-06 | -5.216912 | 4.35561379 | -1.067076 | 1.067076 | Down-regulated |
| EEF1B2 | 4.51E-06 | 1.57E-07 | -5.980699 | 7.10273488 | -1.066746 | 1.066746 | Down-regulated |
| ATP6V0A2 | 4.11E-03 | 1.10E-03 | -3.4402 | -1.38997025 | -1.066464 | 1.066464 | Down-regulated |
| PRKAR2A | 4.79E-03 | 1.41E-03 | -3.357502 | -1.62405864 | -1.066234 | 1.066234 | Down-regulated |
| HCN3 | 7.79E-03 | 3.29E-03 | -3.069511 | -2.40891437 | -1.065965 | 1.065965 | Down-regulated |
| KRAS | 1.35E-03 | 2.11E-04 | -3.960773 | 0.16379234 | -1.065887 | 1.065887 | Down-regulated |
| SPA17 | 8.04E-04 | 1.05E-04 | -4.171167 | 0.8270612 | -1.065459 | 1.065459 | Down-regulated |
| PRPF38B | 2.79E-05 | 1.46E-06 | -5.380349 | 4.93428034 | -1.065416 | 1.065416 | Down-regulated |
| WFS1 | 3.09E-04 | 3.03E-05 | -4.534613 | 2.01369915 | -1.065383 | 1.065383 | Down-regulated |
| COQ2 | 1.12E-02 | 6.14E-03 | -2.84637 | -2.98239692 | -1.065338 | 1.065338 | Down-regulated |
| PEX2 | 6.38E-04 | 7.74E-05 | -4.261317 | 1.11679052 | -1.06512 | 1.06512 | Down-regulated |
| BIK | 9.06E-03 | 4.30E-03 | -2.974837 | -2.65607834 | -1.06507 | 1.06507 | Down-regulated |
| RBP7 | 5.25E-06 | 1.88E-07 | -5.931993 | 6.92471293 | -1.064984 | 1.064984 | Down-regulated |
| ANKS1A | 1.16E-03 | 1.72E-04 | -4.022742 | 0.35720565 | -1.064925 | 1.064925 | Down-regulated |
| GBE1 | 1.14E-03 | 1.66E-04 | -4.032907 | 0.38908943 | -1.064739 | 1.064739 | Down-regulated |
| DYNLRB1 | 2.47E-04 | 2.27E-05 | -4.617832 | 2.29197377 | -1.064664 | 1.064664 | Down-regulated |
| PLAC8 | 5.75E-06 | 2.09E-07 | -5.903811 | 6.82185427 | -1.064322 | 1.064322 | Down-regulated |
| EMG1 | 2.25E-05 | 1.12E-06 | -5.453196 | 5.19401668 | -1.0643 | 1.0643 | Down-regulated |
| CCDC57 | 4.64E-05 | 2.71E-06 | -5.211826 | 4.33770124 | -1.064091 | 1.064091 | Down-regulated |
| COL7A1 | 3.14E-04 | 3.09E-05 | -4.529261 | 1.99588244 | -1.064031 | 1.064031 | Down-regulated |
| GPR1 | 1.09E-05 | 4.57E-07 | -5.694955 | 6.06317972 | -1.064008 | 1.064008 | Down-regulated |
| MYDGF | 1.21E-05 | 5.19E-07 | -5.660597 | 5.93902837 | -1.063635 | 1.063635 | Down-regulated |
| YAF2 | 2.61E-03 | 5.36E-04 | -3.670393 | -0.71930688 | -1.063484 | 1.063484 | Down-regulated |
| SUMO4 | 5.23E-05 | 3.16E-06 | -5.169842 | 4.19006836 | -1.062983 | 1.062983 | Down-regulated |
| PRELID3B | 2.98E-06 | 9.50E-08 | -6.113105 | 7.58818361 | -1.062977 | 1.062977 | Down-regulated |
| NAA25 | 2.30E-05 | 1.15E-06 | -5.445057 | 5.16494497 | -1.062963 | 1.062963 | Down-regulated |
| TRIM11 | 3.30E-06 | 1.08E-07 | -6.079671 | 7.46540435 | -1.062601 | 1.062601 | Down-regulated |
| KRT75 | 6.77E-05 | 4.35E-06 | -5.081394 | 3.88042372 | -1.062241 | 1.062241 | Down-regulated |
| NAPEPLD | 2.85E-03 | 6.13E-04 | -3.627912 | -0.84511409 | -1.062192 | 1.062192 | Down-regulated |
| KLC1 | 6.14E-04 | 7.38E-05 | -4.27539 | 1.1623036 | -1.061961 | 1.061961 | Down-regulated |
| KIAA0391 | 4.05E-02 | 3.56E-02 | -2.152199 | -4.54815789 | -1.061841 | 1.061841 | Down-regulated |
| KCTD10 | 4.04E-05 | 2.29E-06 | -5.257962 | 4.50040304 | -1.061792 | 1.061792 | Down-regulated |
| ZNF28 | 6.79E-05 | 4.38E-06 | -5.079557 | 3.87401324 | -1.061237 | 1.061237 | Down-regulated |
| TTC14 | 2.03E-08 | 2.40E-10 | -7.679323 | 13.41936221 | -1.06098 | 1.06098 | Down-regulated |
| CKAP2 | 2.10E-04 | 1.85E-05 | -4.676226 | 2.48857937 | -1.06018 | 1.06018 | Down-regulated |
| GXYLT1 | 3.41E-05 | 1.86E-06 | -5.314553 | 4.70062622 | -1.059996 | 1.059996 | Down-regulated |
| TLE4 | 3.31E-04 | 3.31E-05 | -4.509289 | 1.92947835 | -1.05998 | 1.05998 | Down-regulated |
| PTPRA | 8.64E-04 | 1.15E-04 | -4.143634 | 0.73921728 | -1.059722 | 1.059722 | Down-regulated |
| CBX4 | 6.82E-03 | 2.58E-03 | -3.152665 | -2.18729985 | -1.059382 | 1.059382 | Down-regulated |
| COX7C | 7.28E-05 | 4.78E-06 | -5.055817 | 3.79124028 | -1.059324 | 1.059324 | Down-regulated |
| CKAP4 | 9.71E-08 | 1.55E-09 | -7.192719 | 11.59983821 | -1.059233 | 1.059233 | Down-regulated |
| PRSS42 | 2.80E-06 | 8.79E-08 | -6.133786 | 7.66419621 | -1.059192 | 1.059192 | Down-regulated |
| POFUT1 | 5.95E-05 | 3.70E-06 | -5.126517 | 4.03815527 | -1.058796 | 1.058796 | Down-regulated |
| TOM1L2 | 2.13E-03 | 3.95E-04 | -3.766344 | -0.43188458 | -1.058783 | 1.058783 | Down-regulated |
| CBX6 | 1.71E-04 | 1.42E-05 | -4.750358 | 2.73970653 | -1.058606 | 1.058606 | Down-regulated |
| BCL2L11 | 1.58E-03 | 2.60E-04 | -3.896034 | -0.03646294 | -1.058564 | 1.058564 | Down-regulated |
| TIGD7 | 1.65E-04 | 1.36E-05 | -4.763062 | 2.78291137 | -1.058331 | 1.058331 | Down-regulated |
| NAT14 | 9.15E-04 | 1.24E-04 | -4.120736 | 0.66639679 | -1.05807 | 1.05807 | Down-regulated |
| SNAPIN | 9.75E-07 | 2.53E-08 | -6.462034 | 8.87641336 | -1.057953 | 1.057953 | Down-regulated |
| UBOX5 | 6.77E-05 | 4.36E-06 | -5.081119 | 3.8794639 | -1.057889 | 1.057889 | Down-regulated |
| GNL2 | 1.81E-05 | 8.59E-07 | -5.524872 | 5.45059104 | -1.056806 | 1.056806 | Down-regulated |
| GET4 | 1.72E-03 | 2.93E-04 | -3.859381 | -0.14900377 | -1.056776 | 1.056776 | Down-regulated |
| APPBP2 | 2.42E-04 | 2.22E-05 | -4.624324 | 2.31377778 | -1.056771 | 1.056771 | Down-regulated |
| DUSP16 | 8.71E-04 | 1.16E-04 | -4.14065 | 0.72971583 | -1.056749 | 1.056749 | Down-regulated |
| DPAGT1 | 5.44E-04 | 6.28E-05 | -4.323022 | 1.31690993 | -1.056649 | 1.056649 | Down-regulated |
| SP110 | 7.12E-04 | 8.98E-05 | -4.21721 | 0.97463751 | -1.056526 | 1.056526 | Down-regulated |
| LINC00672 | 3.45E-03 | 8.23E-04 | -3.53332 | -1.12197136 | -1.056391 | 1.056391 | Down-regulated |
| CABLES2 | 9.81E-05 | 6.96E-06 | -4.95106 | 3.42773044 | -1.056309 | 1.056309 | Down-regulated |
| SLTM | 4.23E-05 | 2.43E-06 | -5.241955 | 4.44389917 | -1.055734 | 1.055734 | Down-regulated |
| VAPA | 9.82E-05 | 6.97E-06 | -4.950563 | 3.4260117 | -1.055495 | 1.055495 | Down-regulated |
| SMARCA5 | 9.97E-04 | 1.40E-04 | -4.085324 | 0.55419988 | -1.055409 | 1.055409 | Down-regulated |
| PSMA3 | 1.42E-03 | 2.23E-04 | -3.942892 | 0.10829526 | -1.055359 | 1.055359 | Down-regulated |
| PLEKHF2 | 5.67E-07 | 1.28E-08 | -6.640353 | 9.53865673 | -1.05532 | 1.05532 | Down-regulated |
| MIR9-3 | 4.90E-04 | 5.46E-05 | -4.363903 | 1.45028357 | -1.054973 | 1.054973 | Down-regulated |
| GSKIP | 4.55E-04 | 4.97E-05 | -4.39101 | 1.5390558 | -1.054929 | 1.054929 | Down-regulated |
| GSTK1 | 1.94E-03 | 3.47E-04 | -3.807213 | -0.30811934 | -1.054379 | 1.054379 | Down-regulated |
| PHF20 | 4.40E-04 | 4.77E-05 | -4.402933 | 1.57818811 | -1.054313 | 1.054313 | Down-regulated |
| HIGD1A | 3.31E-04 | 3.31E-05 | -4.509135 | 1.92896493 | -1.053861 | 1.053861 | Down-regulated |
| CHD8 | 1.46E-03 | 2.33E-04 | -3.929907 | 0.06808208 | -1.05323 | 1.05323 | Down-regulated |
| MANSC1 | 1.87E-03 | 3.29E-04 | -3.823617 | -0.25822213 | -1.05307 | 1.05307 | Down-regulated |
| ZBED8 | 6.34E-03 | 2.27E-03 | -3.19686 | -2.06783061 | -1.052995 | 1.052995 | Down-regulated |
| CEACAM4 | 1.37E-03 | 2.14E-04 | -3.956298 | 0.14989184 | -1.052721 | 1.052721 | Down-regulated |
| ITCH | 9.90E-05 | 7.05E-06 | -4.947699 | 3.41611676 | -1.052693 | 1.052693 | Down-regulated |
| ZNF624 | 6.76E-03 | 2.55E-03 | -3.157164 | -2.17518953 | -1.052371 | 1.052371 | Down-regulated |
| SIGLEC6 | 1.46E-05 | 6.56E-07 | -5.597617 | 5.71197518 | -1.051946 | 1.051946 | Down-regulated |
| SNRPF | 3.14E-04 | 3.09E-05 | -4.529165 | 1.99556014 | -1.051909 | 1.051909 | Down-regulated |
| COL23A1 | 3.51E-04 | 3.58E-05 | -4.48656 | 1.85407198 | -1.051482 | 1.051482 | Down-regulated |
| TRPC4AP | 1.65E-03 | 2.76E-04 | -3.878105 | -0.09158789 | -1.051441 | 1.051441 | Down-regulated |
| PLPP3 | 2.84E-03 | 6.09E-04 | -3.630188 | -0.83839724 | -1.051276 | 1.051276 | Down-regulated |
| SLC26A2 | 1.20E-03 | 1.78E-04 | -4.011451 | 0.32184208 | -1.05127 | 1.05127 | Down-regulated |
| ARHGAP33 | 1.82E-03 | 3.17E-04 | -3.835373 | -0.22238848 | -1.051253 | 1.051253 | Down-regulated |
| HIST2H3D | 3.59E-04 | 3.68E-05 | -4.478624 | 1.82778481 | -1.050911 | 1.050911 | Down-regulated |
| POLR3F | 2.11E-03 | 3.91E-04 | -3.770156 | -0.42037535 | -1.0508 | 1.0508 | Down-regulated |
| SLC35A3 | 2.35E-05 | 1.18E-06 | -5.438601 | 5.14189411 | -1.0507 | 1.0507 | Down-regulated |
| RUBCN | 8.15E-03 | 3.56E-03 | -3.041624 | -2.48229528 | -1.050449 | 1.050449 | Down-regulated |
| SPI1 | 7.86E-03 | 3.34E-03 | -3.063758 | -2.42409328 | -1.050446 | 1.050446 | Down-regulated |
| POM121C | 4.94E-04 | 5.51E-05 | -4.360976 | 1.44071379 | -1.050352 | 1.050352 | Down-regulated |
| SLC50A1 | 3.49E-03 | 8.39E-04 | -3.527421 | -1.13908413 | -1.050068 | 1.050068 | Down-regulated |
| STUM | 7.16E-06 | 2.73E-07 | -5.832149 | 6.5608055 | -1.049784 | 1.049784 | Down-regulated |
| AZI2 | 8.51E-07 | 2.13E-08 | -6.507204 | 9.04395596 | -1.049653 | 1.049653 | Down-regulated |
| IFT27 | 5.83E-06 | 2.12E-07 | -5.899437 | 6.80590016 | -1.049535 | 1.049535 | Down-regulated |
| PRKAG1 | 6.34E-04 | 7.68E-05 | -4.263505 | 1.12385999 | -1.049302 | 1.049302 | Down-regulated |
| NTMT1 | 4.92E-03 | 1.47E-03 | -3.342596 | -1.66585297 | -1.04892 | 1.04892 | Down-regulated |
| ATP2A2 | 4.15E-04 | 4.43E-05 | -4.424642 | 1.64956837 | -1.048904 | 1.048904 | Down-regulated |
| CHPF2 | 5.01E-04 | 5.60E-05 | -4.356335 | 1.42554528 | -1.048616 | 1.048616 | Down-regulated |
| RNF169 | 8.49E-03 | 3.83E-03 | -3.015859 | -2.54966743 | -1.048543 | 1.048543 | Down-regulated |
| SLC35E4 | 4.74E-03 | 1.39E-03 | -3.362867 | -1.60898732 | -1.048446 | 1.048446 | Down-regulated |
| ZNF484 | 1.18E-02 | 6.75E-03 | -2.811712 | -3.068598 | -1.047257 | 1.047257 | Down-regulated |
| IQCF5 | 4.36E-04 | 4.72E-05 | -4.406018 | 1.58832027 | -1.046851 | 1.046851 | Down-regulated |
| RFXAP | 4.41E-03 | 1.23E-03 | -3.402437 | -1.49732618 | -1.046821 | 1.046821 | Down-regulated |
| TMCO6 | 1.18E-03 | 1.75E-04 | -4.016561 | 0.33784162 | -1.046814 | 1.046814 | Down-regulated |
| TIPRL | 8.65E-07 | 2.17E-08 | -6.501986 | 9.02459391 | -1.046536 | 1.046536 | Down-regulated |
| HCST | 1.87E-03 | 3.29E-04 | -3.823364 | -0.25899446 | -1.046402 | 1.046402 | Down-regulated |
| ANXA2P1 | 1.19E-03 | 1.78E-04 | -4.012375 | 0.3247347 | -1.046343 | 1.046343 | Down-regulated |
| LAT2 | 3.89E-07 | 8.20E-09 | -6.757438 | 9.97455472 | -1.045805 | 1.045805 | Down-regulated |
| ZNF674 | 3.02E-03 | 6.72E-04 | -3.598787 | -0.93084571 | -1.045654 | 1.045654 | Down-regulated |
| RHOA | 4.38E-04 | 4.74E-05 | -4.404844 | 1.58446452 | -1.04565 | 1.04565 | Down-regulated |
| MCMDC2 | 1.05E-03 | 1.49E-04 | -4.066142 | 0.49364445 | -1.045537 | 1.045537 | Down-regulated |
| PSMB4 | 3.69E-04 | 3.81E-05 | -4.468279 | 1.7935535 | -1.044647 | 1.044647 | Down-regulated |
| PGGT1B | 2.12E-02 | 1.63E-02 | -2.476909 | -3.85914494 | -1.044455 | 1.044455 | Down-regulated |
| EAF1 | 1.67E-06 | 4.81E-08 | -6.292749 | 8.24997624 | -1.044429 | 1.044429 | Down-regulated |
| C12orf76 | 6.66E-03 | 2.48E-03 | -3.166342 | -2.15045246 | -1.04411 | 1.04411 | Down-regulated |
| SC5D | 8.24E-04 | 1.08E-04 | -4.16158 | 0.7964388 | -1.043881 | 1.043881 | Down-regulated |
| CNIH1 | 7.18E-05 | 4.69E-06 | -5.060787 | 3.80855871 | -1.043625 | 1.043625 | Down-regulated |
| ZBTB24 | 6.98E-04 | 8.73E-05 | -4.225584 | 1.00156729 | -1.043374 | 1.043374 | Down-regulated |
| RPP38 | 8.15E-05 | 5.47E-06 | -5.01804 | 3.6598253 | -1.043071 | 1.043071 | Down-regulated |
| ASGR2 | 1.95E-03 | 3.50E-04 | -3.804478 | -0.31642832 | -1.043028 | 1.043028 | Down-regulated |
| ATG5 | 1.06E-02 | 5.63E-03 | -2.87802 | -2.90299218 | -1.043009 | 1.043009 | Down-regulated |
| SNRK | 2.65E-05 | 1.37E-06 | -5.397541 | 4.99548151 | -1.042971 | 1.042971 | Down-regulated |
| PLEKHO1 | 2.17E-04 | 1.94E-05 | -4.662927 | 2.44370606 | -1.042649 | 1.042649 | Down-regulated |
| HAUS8 | 3.40E-04 | 3.43E-05 | -4.498951 | 1.89515683 | -1.042551 | 1.042551 | Down-regulated |
| ST7 | 3.37E-04 | 3.39E-05 | -4.502561 | 1.90713728 | -1.042507 | 1.042507 | Down-regulated |
| ACACA | 1.08E-04 | 7.89E-06 | -4.916255 | 3.30760357 | -1.042491 | 1.042491 | Down-regulated |
| MED14 | 8.90E-06 | 3.59E-07 | -5.75948 | 6.29685879 | -1.042391 | 1.042391 | Down-regulated |
| PSENEN | 2.75E-03 | 5.80E-04 | -3.645576 | -0.79291271 | -1.042068 | 1.042068 | Down-regulated |
| CES4A | 2.57E-03 | 5.24E-04 | -3.677794 | -0.69729581 | -1.042031 | 1.042031 | Down-regulated |
| TBCE | 9.60E-03 | 4.76E-03 | -2.938575 | -2.74925913 | -1.041985 | 1.041985 | Down-regulated |
| TMEM126B | 5.10E-03 | 1.56E-03 | -3.322947 | -1.72075348 | -1.041748 | 1.041748 | Down-regulated |
| STAT1 | 2.13E-05 | 1.05E-06 | -5.471703 | 5.26016875 | -1.04086 | 1.04086 | Down-regulated |
| TPST1 | 3.76E-03 | 9.48E-04 | -3.487837 | -1.25344568 | -1.040651 | 1.040651 | Down-regulated |
| VRK2 | 1.03E-03 | 1.47E-04 | -4.070518 | 0.50744562 | -1.040575 | 1.040575 | Down-regulated |
| RALGAPA2 | 9.09E-03 | 4.32E-03 | -2.97273 | -2.66151582 | -1.040359 | 1.040359 | Down-regulated |
| MZF1 | 2.66E-05 | 1.39E-06 | -5.395289 | 4.98746309 | -1.040031 | 1.040031 | Down-regulated |
| CEP104 | 1.38E-03 | 2.16E-04 | -3.953315 | 0.14062888 | -1.039892 | 1.039892 | Down-regulated |
| NDUFAF5 | 3.96E-03 | 1.03E-03 | -3.461156 | -1.3300605 | -1.039648 | 1.039648 | Down-regulated |
| CLOCK | 5.00E-04 | 5.59E-05 | -4.356774 | 1.42697907 | -1.039397 | 1.039397 | Down-regulated |
| CD68 | 7.46E-03 | 3.04E-03 | -3.096529 | -2.33736623 | -1.039301 | 1.039301 | Down-regulated |
| MTAP | 9.87E-05 | 7.01E-06 | -4.94895 | 3.42043991 | -1.039203 | 1.039203 | Down-regulated |
| RRM2B | 3.07E-03 | 6.87E-04 | -3.591733 | -0.95154586 | -1.039136 | 1.039136 | Down-regulated |
| SLC22A1 | 2.47E-04 | 2.27E-05 | -4.617983 | 2.29247959 | -1.039086 | 1.039086 | Down-regulated |
| COPG1 | 6.53E-04 | 7.98E-05 | -4.252317 | 1.08772079 | -1.039 | 1.039 | Down-regulated |
| RAD51 | 3.70E-03 | 9.23E-04 | -3.496245 | -1.22922389 | -1.038905 | 1.038905 | Down-regulated |
| ATPAF2 | 6.16E-04 | 7.41E-05 | -4.274338 | 1.15890031 | -1.038709 | 1.038709 | Down-regulated |
| ACRC | 2.91E-05 | 1.54E-06 | -5.366086 | 4.88355343 | -1.038625 | 1.038625 | Down-regulated |
| RPS24 | 9.71E-04 | 1.35E-04 | -4.095961 | 0.58784672 | -1.038297 | 1.038297 | Down-regulated |
| SYNJ1 | 3.16E-03 | 7.17E-04 | -3.577956 | -0.99190027 | -1.038239 | 1.038239 | Down-regulated |
| HIST1H3E | 1.59E-04 | 1.28E-05 | -4.779478 | 2.83881146 | -1.038025 | 1.038025 | Down-regulated |
| PHAX | 8.12E-05 | 5.45E-06 | -5.019357 | 3.66439857 | -1.037851 | 1.037851 | Down-regulated |
| ZEB1-AS1 | 8.37E-06 | 3.33E-07 | -5.779153 | 6.36823502 | -1.037849 | 1.037849 | Down-regulated |
| NSUN4 | 7.85E-03 | 3.33E-03 | -3.064599 | -2.42187419 | -1.037811 | 1.037811 | Down-regulated |
| UCK1 | 7.27E-06 | 2.79E-07 | -5.826561 | 6.54047927 | -1.037806 | 1.037806 | Down-regulated |
| DAD1 | 1.84E-03 | 3.22E-04 | -3.830586 | -0.23698787 | -1.037632 | 1.037632 | Down-regulated |
| TLR4 | 1.77E-04 | 1.49E-05 | -4.737685 | 2.69665796 | -1.037085 | 1.037085 | Down-regulated |
| TSPAN9 | 3.47E-03 | 8.34E-04 | -3.529091 | -1.13424299 | -1.036969 | 1.036969 | Down-regulated |
| TMED7 | 2.51E-08 | 3.07E-10 | -7.61548 | 13.18076498 | -1.036869 | 1.036869 | Down-regulated |
| TMEFF1 | 2.07E-04 | 1.82E-05 | -4.68119 | 2.50534198 | -1.036701 | 1.036701 | Down-regulated |
| COL20A1 | 8.45E-03 | 3.79E-03 | -3.019271 | -2.54076837 | -1.036609 | 1.036609 | Down-regulated |
| SLC7A5 | 1.74E-05 | 8.14E-07 | -5.539164 | 5.50186912 | -1.036566 | 1.036566 | Down-regulated |
| VPS33A | 7.12E-07 | 1.71E-08 | -6.565471 | 9.26029668 | -1.036493 | 1.036493 | Down-regulated |
| FAM63B | 5.73E-05 | 3.53E-06 | -5.139433 | 4.08339505 | -1.036363 | 1.036363 | Down-regulated |
| APTX | 2.24E-05 | 1.11E-06 | -5.455409 | 5.201922 | -1.036291 | 1.036291 | Down-regulated |
| MRTO4 | 4.64E-03 | 1.34E-03 | -3.374329 | -1.57673095 | -1.036252 | 1.036252 | Down-regulated |
| ZKSCAN5 | 1.97E-02 | 1.47E-02 | -2.517321 | -3.76790386 | -1.035678 | 1.035678 | Down-regulated |
| LXN | 7.11E-04 | 8.96E-05 | -4.218103 | 0.97750725 | -1.035391 | 1.035391 | Down-regulated |
| SLC25A19 | 3.61E-04 | 3.71E-05 | -4.476288 | 1.82005301 | -1.034878 | 1.034878 | Down-regulated |
| KITLG | 3.21E-04 | 3.18E-05 | -4.520498 | 1.96672981 | -1.034849 | 1.034849 | Down-regulated |
| BNIPL | 6.95E-07 | 1.65E-08 | -6.574438 | 9.29361027 | -1.034494 | 1.034494 | Down-regulated |
| LMAN1 | 7.47E-03 | 3.05E-03 | -3.095867 | -2.33912469 | -1.034325 | 1.034325 | Down-regulated |
| TTI2 | 6.13E-04 | 7.35E-05 | -4.276392 | 1.16554822 | -1.034137 | 1.034137 | Down-regulated |
| SLC9A8 | 3.07E-04 | 3.00E-05 | -4.537383 | 2.02292475 | -1.034133 | 1.034133 | Down-regulated |
| BUD13 | 1.21E-04 | 9.11E-06 | -4.875772 | 3.16829679 | -1.033699 | 1.033699 | Down-regulated |
| ERAP1 | 9.23E-06 | 3.76E-07 | -5.747174 | 6.2522388 | -1.033662 | 1.033662 | Down-regulated |
| RANBP3 | 7.65E-05 | 5.07E-06 | -5.03934 | 3.73387824 | -1.033577 | 1.033577 | Down-regulated |
| DTWD2 | 2.38E-03 | 4.69E-04 | -3.712955 | -0.59236452 | -1.033349 | 1.033349 | Down-regulated |
| EIF2AK2 | 3.57E-03 | 8.71E-04 | -3.515274 | -1.1742657 | -1.032899 | 1.032899 | Down-regulated |
| RWDD2B | 2.69E-04 | 2.52E-05 | -4.587273 | 2.18952166 | -1.032515 | 1.032515 | Down-regulated |
| MOB3C | 5.22E-05 | 3.15E-06 | -5.170819 | 4.19350036 | -1.032226 | 1.032226 | Down-regulated |
| CACNA1C-AS4 | 9.10E-04 | 1.23E-04 | -4.122933 | 0.67337494 | -1.032056 | 1.032056 | Down-regulated |
| ZNF277 | 1.32E-03 | 2.05E-04 | -3.969768 | 0.19176464 | -1.032034 | 1.032034 | Down-regulated |
| GAR1 | 1.66E-04 | 1.37E-05 | -4.760859 | 2.77541681 | -1.03203 | 1.03203 | Down-regulated |
| CDC40 | 9.30E-04 | 1.27E-04 | -4.113883 | 0.6446431 | -1.031877 | 1.031877 | Down-regulated |
| THG1L | 6.04E-04 | 7.22E-05 | -4.2818 | 1.18306001 | -1.031848 | 1.031848 | Down-regulated |
| TRIOBP | 3.38E-03 | 7.99E-04 | -3.542921 | -1.09408141 | -1.031735 | 1.031735 | Down-regulated |
| ACPT | 5.95E-04 | 7.07E-05 | -4.287846 | 1.20265132 | -1.031559 | 1.031559 | Down-regulated |
| DRICH1 | 2.47E-02 | 1.98E-02 | -2.398401 | -4.0329984 | -1.031191 | 1.031191 | Down-regulated |
| FBXO42 | 8.84E-06 | 3.56E-07 | -5.761641 | 6.30469607 | -1.031163 | 1.031163 | Down-regulated |
| YTHDC1 | 2.30E-03 | 4.43E-04 | -3.730438 | -0.53996415 | -1.031049 | 1.031049 | Down-regulated |
| BCL9 | 2.50E-07 | 4.83E-09 | -6.895758 | 10.49036144 | -1.030839 | 1.030839 | Down-regulated |
| ZNF106 | 4.00E-05 | 2.26E-06 | -5.261539 | 4.51303941 | -1.030733 | 1.030733 | Down-regulated |
| SLAMF7 | 2.39E-03 | 4.73E-04 | -3.710251 | -0.60045538 | -1.030462 | 1.030462 | Down-regulated |
| HN1L | 6.40E-07 | 1.49E-08 | -6.601005 | 9.39234321 | -1.030334 | 1.030334 | Down-regulated |
| ZFAND6 | 7.00E-03 | 2.72E-03 | -3.135164 | -2.23428811 | -1.030303 | 1.030303 | Down-regulated |
| GLB1L | 7.38E-03 | 2.99E-03 | -3.102713 | -2.32092931 | -1.030209 | 1.030209 | Down-regulated |
| PLOD2 | 9.29E-04 | 1.27E-04 | -4.114521 | 0.64666709 | -1.030001 | 1.030001 | Down-regulated |
| RPS19 | 5.60E-03 | 1.83E-03 | -3.270185 | -1.86710038 | -1.029714 | 1.029714 | Down-regulated |
| CUX1 | 1.15E-06 | 3.08E-08 | -6.410524 | 8.68554599 | -1.029666 | 1.029666 | Down-regulated |
| HAT1 | 5.30E-05 | 3.21E-06 | -5.165273 | 4.17402602 | -1.029641 | 1.029641 | Down-regulated |
| VWA5A | 1.30E-03 | 1.99E-04 | -3.977682 | 0.21640241 | -1.029569 | 1.029569 | Down-regulated |
| CXorf21 | 3.47E-03 | 8.34E-04 | -3.529361 | -1.13345791 | -1.029534 | 1.029534 | Down-regulated |
| ZXDC | 2.59E-02 | 2.10E-02 | -2.373392 | -4.08742519 | -1.029101 | 1.029101 | Down-regulated |
| PRR11 | 1.53E-03 | 2.49E-04 | -3.909619 | 0.00540427 | -1.02874 | 1.02874 | Down-regulated |
| MOB1A | 2.97E-03 | 6.53E-04 | -3.608031 | -0.9036817 | -1.028619 | 1.028619 | Down-regulated |
| HRAS | 1.21E-02 | 7.06E-03 | -2.795295 | -3.10915264 | -1.028374 | 1.028374 | Down-regulated |
| HARBI1 | 3.01E-04 | 2.92E-05 | -4.545131 | 2.04874399 | -1.028334 | 1.028334 | Down-regulated |
| MED17 | 5.09E-03 | 1.56E-03 | -3.324425 | -1.7166326 | -1.028326 | 1.028326 | Down-regulated |
| TTLL4 | 3.46E-03 | 8.28E-04 | -3.531351 | -1.127685 | -1.028001 | 1.028001 | Down-regulated |
| IGFBP2 | 7.23E-06 | 2.77E-07 | -5.828752 | 6.54844968 | -1.027981 | 1.027981 | Down-regulated |
| FXR1 | 3.07E-04 | 3.00E-05 | -4.537128 | 2.02207397 | -1.027952 | 1.027952 | Down-regulated |
| METTL25 | 2.29E-02 | 1.80E-02 | -2.435482 | -3.95144885 | -1.027943 | 1.027943 | Down-regulated |
| DMC1 | 6.47E-04 | 7.88E-05 | -4.256126 | 1.10002146 | -1.027888 | 1.027888 | Down-regulated |
| C2orf82 | 3.15E-03 | 7.16E-04 | -3.578364 | -0.99070464 | -1.02708 | 1.02708 | Down-regulated |
| PRDM4 | 2.58E-05 | 1.33E-06 | -5.405964 | 5.02548983 | -1.026941 | 1.026941 | Down-regulated |
| ZNF706 | 1.81E-04 | 1.53E-05 | -4.728993 | 2.66715786 | -1.026858 | 1.026858 | Down-regulated |
| ADCY10P1 | 1.90E-04 | 1.63E-05 | -4.71127 | 2.6070813 | -1.026776 | 1.026776 | Down-regulated |
| FAM20A | 9.60E-04 | 1.33E-04 | -4.100908 | 0.60351201 | -1.026737 | 1.026737 | Down-regulated |
| COMMD6 | 8.44E-03 | 3.79E-03 | -3.019833 | -2.53930181 | -1.026421 | 1.026421 | Down-regulated |
| MPND | 2.17E-03 | 4.08E-04 | -3.756505 | -0.46156063 | -1.026382 | 1.026382 | Down-regulated |
| TMPPE | 4.13E-03 | 1.10E-03 | -3.438399 | -1.39510749 | -1.025878 | 1.025878 | Down-regulated |
| GPATCH8 | 2.24E-05 | 1.12E-06 | -5.45394 | 5.19667503 | -1.025122 | 1.025122 | Down-regulated |
| GIMAP2 | 2.84E-04 | 2.70E-05 | -4.568002 | 2.12507329 | -1.025045 | 1.025045 | Down-regulated |
| PAGR1 | 1.67E-04 | 1.39E-05 | -4.757906 | 2.76537111 | -1.0249 | 1.0249 | Down-regulated |
| LINC00382 | 3.38E-03 | 7.99E-04 | -3.543112 | -1.09352606 | -1.024876 | 1.024876 | Down-regulated |
| ATP5S | 1.26E-03 | 1.91E-04 | -3.990574 | 0.25659795 | -1.024792 | 1.024792 | Down-regulated |
| MRPL46 | 1.32E-05 | 5.76E-07 | -5.632386 | 5.83723769 | -1.024679 | 1.024679 | Down-regulated |
| LINC00938 | 4.04E-04 | 4.26E-05 | -4.435847 | 1.68647697 | -1.024677 | 1.024677 | Down-regulated |
| NCAPD2 | 3.14E-03 | 7.11E-04 | -3.580583 | -0.98421331 | -1.024639 | 1.024639 | Down-regulated |
| ETS2 | 3.15E-06 | 1.02E-07 | -6.094314 | 7.51916108 | -1.02413 | 1.02413 | Down-regulated |
| TRIM65 | 3.12E-06 | 1.01E-07 | -6.098186 | 7.53338183 | -1.023974 | 1.023974 | Down-regulated |
| IL17RC | 1.23E-04 | 9.28E-06 | -4.870619 | 3.15059763 | -1.023847 | 1.023847 | Down-regulated |
| FCHSD1 | 8.79E-04 | 1.18E-04 | -4.136454 | 0.71636049 | -1.023676 | 1.023676 | Down-regulated |
| FBXO6 | 6.35E-03 | 2.28E-03 | -3.195203 | -2.0723304 | -1.023441 | 1.023441 | Down-regulated |
| PSG3 | 4.28E-03 | 1.18E-03 | -3.417145 | -1.45560563 | -1.02344 | 1.02344 | Down-regulated |
| LINC00202-2 | 1.46E-03 | 2.33E-04 | -3.930468 | 0.06981849 | -1.023118 | 1.023118 | Down-regulated |
| CDK2AP1 | 2.93E-04 | 2.82E-05 | -4.555306 | 2.08267864 | -1.02303 | 1.02303 | Down-regulated |
| NICN1 | 6.59E-04 | 8.06E-05 | -4.249175 | 1.07758178 | -1.022764 | 1.022764 | Down-regulated |
| OTOGL | 1.73E-04 | 1.44E-05 | -4.746566 | 2.72681979 | -1.02214 | 1.02214 | Down-regulated |
| FNDC3B | 5.99E-04 | 7.14E-05 | -4.284956 | 1.19328576 | -1.021975 | 1.021975 | Down-regulated |
| KIAA1958 | 1.02E-02 | 5.30E-03 | -2.90001 | -2.847438 | -1.021963 | 1.021963 | Down-regulated |
| PDE12 | 5.09E-04 | 5.73E-05 | -4.349559 | 1.40341492 | -1.021699 | 1.021699 | Down-regulated |
| DGAT2 | 8.99E-03 | 4.23E-03 | -2.980399 | -2.64171143 | -1.021656 | 1.021656 | Down-regulated |
| UBA52 | 6.73E-05 | 4.31E-06 | -5.083896 | 3.88915443 | -1.021451 | 1.021451 | Down-regulated |
| UBXN11 | 1.52E-03 | 2.47E-04 | -3.911813 | 0.0121748 | -1.021281 | 1.021281 | Down-regulated |
| CCNYL1 | 9.36E-05 | 6.55E-06 | -4.967996 | 3.4863043 | -1.021194 | 1.021194 | Down-regulated |
| LPCAT3 | 1.62E-02 | 1.11E-02 | -2.625137 | -3.51876759 | -1.021023 | 1.021023 | Down-regulated |
| NMNAT1 | 1.66E-07 | 2.96E-09 | -7.023898 | 10.96883905 | -1.020793 | 1.020793 | Down-regulated |
| PRDM10 | 1.43E-06 | 4.01E-08 | -6.341112 | 8.4286908 | -1.020397 | 1.020397 | Down-regulated |
| ZSCAN29 | 1.25E-02 | 7.39E-03 | -2.77871 | -3.14994033 | -1.020365 | 1.020365 | Down-regulated |
| NSD1 | 1.37E-05 | 6.06E-07 | -5.618744 | 5.78806434 | -1.020158 | 1.020158 | Down-regulated |
| KYAT3 | 1.30E-03 | 2.01E-04 | -3.975907 | 0.210874 | -1.020032 | 1.020032 | Down-regulated |
| PRC1 | 8.41E-04 | 1.11E-04 | -4.153603 | 0.77098718 | -1.020005 | 1.020005 | Down-regulated |
| SLC6A6 | 1.63E-04 | 1.34E-05 | -4.767952 | 2.7995541 | -1.019948 | 1.019948 | Down-regulated |
| TMEM114 | 2.26E-04 | 2.04E-05 | -4.6485 | 2.39509381 | -1.019881 | 1.019881 | Down-regulated |
| RETNLB | 5.39E-03 | 1.72E-03 | -3.291861 | -1.80716815 | -1.019834 | 1.019834 | Down-regulated |
| MGAT4A | 1.35E-03 | 2.10E-04 | -3.961945 | 0.16743618 | -1.019006 | 1.019006 | Down-regulated |
| CECR5 | 1.10E-03 | 1.59E-04 | -4.046494 | 0.43177621 | -1.018963 | 1.018963 | Down-regulated |
| CEACAM21 | 1.65E-04 | 1.35E-05 | -4.764941 | 2.78930463 | -1.018781 | 1.018781 | Down-regulated |
| PDIK1L | 4.24E-04 | 4.54E-05 | -4.417827 | 1.62714152 | -1.018582 | 1.018582 | Down-regulated |
| EXTL3 | 6.70E-05 | 4.29E-06 | -5.085512 | 3.89479686 | -1.018526 | 1.018526 | Down-regulated |
| RAB21 | 1.95E-04 | 1.68E-05 | -4.703717 | 2.58150813 | -1.018339 | 1.018339 | Down-regulated |
| BEND7 | 1.56E-04 | 1.26E-05 | -4.78553 | 2.85943841 | -1.017885 | 1.017885 | Down-regulated |
| CYB5D1 | 1.86E-05 | 8.84E-07 | -5.516952 | 5.42219509 | -1.01775 | 1.01775 | Down-regulated |
| MYADML | 3.57E-04 | 3.65E-05 | -4.480655 | 1.83451233 | -1.017712 | 1.017712 | Down-regulated |
| GNAT1 | 2.64E-03 | 5.48E-04 | -3.663538 | -0.73966964 | -1.017593 | 1.017593 | Down-regulated |
| TBC1D32 | 6.51E-04 | 7.94E-05 | -4.253671 | 1.09209319 | -1.017348 | 1.017348 | Down-regulated |
| FBXW2 | 1.09E-02 | 5.95E-03 | -2.857924 | -2.95348665 | -1.017032 | 1.017032 | Down-regulated |
| CDK10 | 1.47E-04 | 1.17E-05 | -4.80544 | 2.92738063 | -1.016987 | 1.016987 | Down-regulated |
| DCUN1D5 | 7.12E-05 | 4.64E-06 | -5.063994 | 3.81973389 | -1.016916 | 1.016916 | Down-regulated |
| ZEB2 | 9.24E-05 | 6.44E-06 | -4.972655 | 3.50243176 | -1.016701 | 1.016701 | Down-regulated |
| UNC80 | 9.92E-03 | 5.04E-03 | -2.918198 | -2.80125354 | -1.016678 | 1.016678 | Down-regulated |
| KIAA1683 | 3.16E-04 | 3.12E-05 | -4.526368 | 1.98625537 | -1.016316 | 1.016316 | Down-regulated |
| HNRNPLL | 6.15E-04 | 7.38E-05 | -4.275144 | 1.16150845 | -1.016265 | 1.016265 | Down-regulated |
| CTIF | 8.94E-05 | 6.16E-06 | -4.985135 | 3.54565683 | -1.016157 | 1.016157 | Down-regulated |
| ZDHHC8P1 | 1.86E-02 | 1.36E-02 | -2.547094 | -3.6999311 | -1.016147 | 1.016147 | Down-regulated |
| STT3A | 4.36E-03 | 1.21E-03 | -3.408661 | -1.47968609 | -1.016064 | 1.016064 | Down-regulated |
| ANKRD44 | 1.46E-03 | 2.33E-04 | -3.929586 | 0.06708968 | -1.016016 | 1.016016 | Down-regulated |
| G3BP2 | 1.51E-05 | 6.85E-07 | -5.585713 | 5.66913589 | -1.015927 | 1.015927 | Down-regulated |
| ZHX2 | 4.17E-03 | 1.12E-03 | -3.432215 | -1.41273399 | -1.015791 | 1.015791 | Down-regulated |
| SEPT1 | 4.18E-04 | 4.47E-05 | -4.422139 | 1.64132826 | -1.015619 | 1.015619 | Down-regulated |
| JMJD7-PLA2G4B | 1.32E-05 | 5.77E-07 | -5.631964 | 5.83571602 | -1.015443 | 1.015443 | Down-regulated |
| TOR4A | 1.84E-04 | 1.57E-05 | -4.722282 | 2.64439995 | -1.015256 | 1.015256 | Down-regulated |
| CASP5 | 6.91E-04 | 8.63E-05 | -4.229167 | 1.01309854 | -1.015083 | 1.015083 | Down-regulated |
| C16orf86 | 7.22E-03 | 2.87E-03 | -3.11687 | -2.28320897 | -1.015075 | 1.015075 | Down-regulated |
| ZNF207 | 3.19E-02 | 2.71E-02 | -2.269202 | -4.30911463 | -1.014902 | 1.014902 | Down-regulated |
| TRPM6 | 1.17E-02 | 6.64E-03 | -2.817934 | -3.05317996 | -1.014592 | 1.014592 | Down-regulated |
| FAM86B1 | 6.17E-03 | 2.17E-03 | -3.211895 | -2.02692789 | -1.014444 | 1.014444 | Down-regulated |
| HCP5 | 2.22E-04 | 1.99E-05 | -4.65536 | 2.41820227 | -1.014037 | 1.014037 | Down-regulated |
| SNX5 | 1.17E-02 | 6.70E-03 | -2.814795 | -3.06096292 | -1.014019 | 1.014019 | Down-regulated |
| KIF22 | 1.23E-03 | 1.84E-04 | -4.001449 | 0.29056067 | -1.0137 | 1.0137 | Down-regulated |
| IFRD1 | 6.87E-03 | 2.62E-03 | -3.147561 | -2.20102063 | -1.013213 | 1.013213 | Down-regulated |
| PTP4A1 | 5.64E-04 | 6.57E-05 | -4.309665 | 1.27347036 | -1.013202 | 1.013202 | Down-regulated |
| EBP | 1.96E-04 | 1.69E-05 | -4.701623 | 2.5744207 | -1.013019 | 1.013019 | Down-regulated |
| TMEM106A | 1.78E-04 | 1.50E-05 | -4.735724 | 2.6900014 | -1.012929 | 1.012929 | Down-regulated |
| CCDC50 | 1.72E-03 | 2.92E-04 | -3.861066 | -0.14384292 | -1.012649 | 1.012649 | Down-regulated |
| MAB21L3 | 4.25E-03 | 1.16E-03 | -3.422236 | -1.44113505 | -1.012624 | 1.012624 | Down-regulated |
| EZR-AS1 | 2.61E-03 | 5.37E-04 | -3.670204 | -0.71986733 | -1.012455 | 1.012455 | Down-regulated |
| KCTD5 | 5.72E-03 | 1.90E-03 | -3.257088 | -1.90318167 | -1.012377 | 1.012377 | Down-regulated |
| HTR1E | 3.50E-05 | 1.92E-06 | -5.306755 | 4.67299539 | -1.012348 | 1.012348 | Down-regulated |
| ATXN3 | 6.38E-05 | 4.05E-06 | -5.101622 | 3.95107078 | -1.012277 | 1.012277 | Down-regulated |
| GOLM1 | 6.11E-04 | 7.32E-05 | -4.277602 | 1.16946533 | -1.012205 | 1.012205 | Down-regulated |
| ACOT9 | 1.10E-03 | 1.60E-04 | -4.044935 | 0.42687678 | -1.011648 | 1.011648 | Down-regulated |
| MEIS3 | 1.01E-02 | 5.18E-03 | -2.908359 | -2.8262655 | -1.011412 | 1.011412 | Down-regulated |
| NPHP4 | 7.15E-04 | 9.03E-05 | -4.215592 | 0.96943853 | -1.01106 | 1.01106 | Down-regulated |
| AKAP10 | 1.01E-04 | 7.24E-06 | -4.940104 | 3.38988021 | -1.010965 | 1.010965 | Down-regulated |
| CELA1 | 1.29E-05 | 5.63E-07 | -5.638828 | 5.86046907 | -1.010853 | 1.010853 | Down-regulated |
| MAD2L1BP | 9.81E-03 | 4.94E-03 | -2.925171 | -2.78349244 | -1.010712 | 1.010712 | Down-regulated |
| UGP2 | 4.04E-05 | 2.28E-06 | -5.258812 | 4.50340543 | -1.010335 | 1.010335 | Down-regulated |
| RHBDD1 | 1.41E-02 | 9.02E-03 | -2.704089 | -3.33116618 | -1.0102 | 1.0102 | Down-regulated |
| C17orf80 | 1.56E-04 | 1.26E-05 | -4.785197 | 2.85830513 | -1.009997 | 1.009997 | Down-regulated |
| DDX27 | 1.14E-06 | 3.04E-08 | -6.413611 | 8.69697747 | -1.009654 | 1.009654 | Down-regulated |
| DACH1 | 8.95E-06 | 3.62E-07 | -5.757294 | 6.28893011 | -1.009048 | 1.009048 | Down-regulated |
| GTF3C3 | 5.32E-05 | 3.23E-06 | -5.163548 | 4.16797009 | -1.008989 | 1.008989 | Down-regulated |
| APOM | 1.67E-04 | 1.38E-05 | -4.759006 | 2.76911198 | -1.008606 | 1.008606 | Down-regulated |
| LY6H | 1.05E-04 | 7.58E-06 | -4.927248 | 3.34550787 | -1.00856 | 1.00856 | Down-regulated |
| ADAMTSL4 | 1.72E-03 | 2.92E-04 | -3.860168 | -0.14659458 | -1.008231 | 1.008231 | Down-regulated |
| STX10 | 1.28E-03 | 1.96E-04 | -3.982512 | 0.23145439 | -1.007938 | 1.007938 | Down-regulated |
| CDKN2C | 3.59E-05 | 1.98E-06 | -5.297918 | 4.64169747 | -1.007935 | 1.007935 | Down-regulated |
| TAB2 | 1.90E-03 | 3.37E-04 | -3.816027 | -0.28132536 | -1.007911 | 1.007911 | Down-regulated |
| ARL6IP1 | 7.08E-04 | 8.91E-05 | -4.219637 | 0.9824411 | -1.007653 | 1.007653 | Down-regulated |
| ANP32A | 1.25E-04 | 9.50E-06 | -4.8641 | 3.12821593 | -1.007592 | 1.007592 | Down-regulated |
| RMND1 | 4.90E-05 | 2.90E-06 | -5.193685 | 4.27385891 | -1.007411 | 1.007411 | Down-regulated |
| TSC1 | 4.41E-03 | 1.23E-03 | -3.402031 | -1.4984761 | -1.007049 | 1.007049 | Down-regulated |
| YBEY | 5.47E-05 | 3.34E-06 | -5.154344 | 4.13567543 | -1.006644 | 1.006644 | Down-regulated |
| NOL7 | 2.19E-05 | 1.08E-06 | -5.46186 | 5.22497767 | -1.006264 | 1.006264 | Down-regulated |
| C14orf166 | 2.81E-04 | 2.66E-05 | -4.571762 | 2.13763808 | -1.006218 | 1.006218 | Down-regulated |
| SNORD55 | 1.07E-02 | 5.69E-03 | -2.87445 | -2.91198231 | -1.00603 | 1.00603 | Down-regulated |
| ANKRD37 | 1.25E-04 | 9.52E-06 | -4.863469 | 3.12605285 | -1.006024 | 1.006024 | Down-regulated |
| MAGED4B | 2.13E-04 | 1.88E-05 | -4.671724 | 2.47338133 | -1.005933 | 1.005933 | Down-regulated |
| HIST1H4D | 3.55E-05 | 1.95E-06 | -5.301426 | 4.65411932 | -1.005755 | 1.005755 | Down-regulated |
| CADM4 | 1.43E-03 | 2.27E-04 | -3.938184 | 0.09370699 | -1.005494 | 1.005494 | Down-regulated |
| MON2 | 8.09E-04 | 1.06E-04 | -4.168631 | 0.8189581 | -1.005461 | 1.005461 | Down-regulated |
| MIS18BP1 | 5.60E-04 | 6.51E-05 | -4.312333 | 1.28214063 | -1.005413 | 1.005413 | Down-regulated |
| LOC100419583 | 1.17E-04 | 8.72E-06 | -4.888078 | 3.21059388 | -1.005213 | 1.005213 | Down-regulated |
| LGALS3 | 6.06E-05 | 3.79E-06 | -5.119706 | 4.01431519 | -1.004785 | 1.004785 | Down-regulated |
| NMRK1 | 2.86E-05 | 1.50E-06 | -5.372872 | 4.90768281 | -1.004493 | 1.004493 | Down-regulated |
| P3H1 | 2.55E-04 | 2.36E-05 | -4.606675 | 2.25453482 | -1.004379 | 1.004379 | Down-regulated |
| FUBP3 | 1.64E-05 | 7.59E-07 | -5.558303 | 5.57059551 | -1.004378 | 1.004378 | Down-regulated |
| SCIMP | 1.84E-03 | 3.22E-04 | -3.830098 | -0.23847633 | -1.004243 | 1.004243 | Down-regulated |
| ACSL4 | 9.39E-03 | 4.57E-03 | -2.952786 | -2.71284224 | -1.004214 | 1.004214 | Down-regulated |
| KRTAP10-2 | 3.76E-04 | 3.90E-05 | -4.461569 | 1.7713706 | -1.004087 | 1.004087 | Down-regulated |
| NOC4L | 1.27E-03 | 1.93E-04 | -3.988018 | 0.24862173 | -1.004045 | 1.004045 | Down-regulated |
| HS2ST1 | 1.59E-02 | 1.09E-02 | -2.633757 | -3.49849348 | -1.003814 | 1.003814 | Down-regulated |
| DNAJC13 | 1.38E-05 | 6.09E-07 | -5.617601 | 5.78394485 | -1.003712 | 1.003712 | Down-regulated |
| REPS2 | 4.04E-05 | 2.28E-06 | -5.258965 | 4.50394666 | -1.003686 | 1.003686 | Down-regulated |
| HMMR | 7.79E-04 | 1.01E-04 | -4.183385 | 0.86613941 | -1.003587 | 1.003587 | Down-regulated |
| FBXW11 | 4.25E-03 | 1.16E-03 | -3.422402 | -1.44066351 | -1.003561 | 1.003561 | Down-regulated |
| CAPZB | 2.37E-02 | 1.88E-02 | -2.418975 | -3.9878766 | -1.003538 | 1.003538 | Down-regulated |
| PPT2 | 1.24E-05 | 5.35E-07 | -5.652544 | 5.9099586 | -1.002953 | 1.002953 | Down-regulated |
| CSNK1E | 8.06E-04 | 1.05E-04 | -4.170374 | 0.82452513 | -1.002828 | 1.002828 | Down-regulated |
| ACAD9 | 3.18E-03 | 7.26E-04 | -3.573783 | -1.00410408 | -1.002621 | 1.002621 | Down-regulated |
| CHMP2A | 3.73E-05 | 2.08E-06 | -5.284066 | 4.59267567 | -1.002493 | 1.002493 | Down-regulated |
| RBM23 | 1.76E-03 | 3.03E-04 | -3.849225 | -0.18007921 | -1.002383 | 1.002383 | Down-regulated |
| TMEM251 | 1.05E-02 | 5.53E-03 | -2.884463 | -2.88674788 | -1.002158 | 1.002158 | Down-regulated |
| SPATA2 | 6.54E-03 | 2.41E-03 | -3.177293 | -2.12086823 | -1.002062 | 1.002062 | Down-regulated |
| ATG2A | 6.46E-03 | 2.35E-03 | -3.184709 | -2.10079366 | -1.001712 | 1.001712 | Down-regulated |
| E2F7 | 4.09E-03 | 1.09E-03 | -3.443201 | -1.3814029 | -1.000862 | 1.000862 | Down-regulated |
| PRKDC | 3.63E-04 | 3.74E-05 | -4.473691 | 1.81145833 | -1.000771 | 1.000771 | Down-regulated |
| SIPA1L1 | 1.72E-05 | 8.03E-07 | -5.542993 | 5.51561506 | -1.000731 | 1.000731 | Down-regulated |
| PPARG | 1.45E-05 | 6.48E-07 | -5.600888 | 5.72375053 | -1.00064 | 1.00064 | Down-regulated |
| VPS72 | 1.83E-03 | 3.19E-04 | -3.833124 | -0.22924614 | -1.000188 | 1.000188 | Down-regulated |
| QTRT2 | 2.14E-03 | 3.99E-04 | -3.763683 | -0.43991398 | -1.000006 | 1.000006 | Down-regulated |
| GUK1 | 8.77E-04 | 1.17E-04 | 4.137828 | 0.72073219 | 1.010906 | 1.010906 | Up-regulated |
| PCSK1N | 1.93E-02 | 1.43E-02 | 2.52648 | -3.74706055 | 1.021231 | 1.021231 | Up-regulated |
| SLC8A2 | 1.47E-02 | 9.57E-03 | 2.681765 | -3.38464646 | 1.029587 | 1.029587 | Up-regulated |
| PABPC1 | 1.23E-02 | 7.27E-03 | 2.784454 | -3.13583416 | 1.030514 | 1.030514 | Up-regulated |
| GTPBP3 | 1.79E-04 | 1.51E-05 | 4.732717 | 2.67979399 | 1.03644 | 1.03644 | Up-regulated |
| MAD1L1 | 7.74E-03 | 3.25E-03 | 3.073198 | -2.39917845 | 1.036751 | 1.036751 | Up-regulated |
| GZMK | 4.26E-06 | 1.46E-07 | 5.998358 | 7.16735291 | 1.051077 | 1.051077 | Up-regulated |
| RPLP0 | 7.38E-03 | 2.98E-03 | 3.103033 | -2.32007661 | 1.058189 | 1.058189 | Up-regulated |
| LMF2 | 5.60E-04 | 6.50E-05 | 4.312784 | 1.28360783 | 1.07599 | 1.07599 | Up-regulated |
| CHCHD10 | 1.67E-02 | 1.17E-02 | 2.606346 | -3.56277975 | 1.076841 | 1.076841 | Up-regulated |
| RPS4X | 3.75E-06 | 1.26E-07 | 6.037486 | 7.31067624 | 1.093512 | 1.093512 | Up-regulated |
| GYPB | 8.91E-05 | 6.13E-06 | 4.986277 | 3.54961442 | 1.101552 | 1.101552 | Up-regulated |
| CPVL | 7.80E-04 | 1.01E-04 | 4.182886 | 0.86454148 | 1.101555 | 1.101555 | Up-regulated |
| BMP2K | 1.61E-04 | 1.31E-05 | 4.773203 | 2.81743406 | 1.105264 | 1.105264 | Up-regulated |
| FAM21C | 2.15E-03 | 4.03E-04 | 3.760724 | -0.44884157 | 1.105269 | 1.105269 | Up-regulated |
| SNORD31 | 1.88E-02 | 1.38E-02 | 2.540238 | -3.71563991 | 1.113265 | 1.113265 | Up-regulated |
| CA2 | 8.95E-03 | 4.21E-03 | 2.982425 | -2.63647524 | 1.114626 | 1.114626 | Up-regulated |
| BNIP3L | 2.05E-03 | 3.74E-04 | 3.783432 | -0.38023315 | 1.115449 | 1.115449 | Up-regulated |
| ADD1 | 1.55E-02 | 1.05E-02 | 2.647987 | -3.46491593 | 1.125016 | 1.125016 | Up-regulated |
| XKR8 | 1.90E-05 | 9.12E-07 | 5.508683 | 5.3925569 | 1.16482 | 1.16482 | Up-regulated |
| TAGLN2 | 9.76E-03 | 4.90E-03 | 2.928114 | -2.77598451 | 1.168866 | 1.168866 | Up-regulated |
| CYB561 | 2.90E-02 | 2.42E-02 | 2.315853 | -4.21086812 | 1.199539 | 1.199539 | Up-regulated |
| RPL15 | 7.00E-03 | 2.72E-03 | 3.135575 | -2.23318582 | 1.223871 | 1.223871 | Up-regulated |
| TRIM10 | 4.65E-02 | 4.14E-02 | 2.086683 | -4.67732807 | 1.227776 | 1.227776 | Up-regulated |
| VIPR1 | 3.00E-02 | 2.52E-02 | 2.299843 | -4.24477103 | 1.243998 | 1.243998 | Up-regulated |
| ALDH1A1 | 2.11E-02 | 1.61E-02 | 2.480043 | -3.85211209 | 1.268879 | 1.268879 | Up-regulated |
| EPB41 | 1.34E-02 | 8.31E-03 | 2.734964 | -3.25663733 | 1.291919 | 1.291919 | Up-regulated |
| MPHOSPH10 | 3.33E-03 | 7.81E-04 | 3.550536 | -1.07192546 | 1.300893 | 1.300893 | Up-regulated |
| SMAD3 | 1.48E-03 | 2.39E-04 | 3.922351 | 0.04471847 | 1.334415 | 1.334415 | Up-regulated |
| EIF4B | 1.44E-02 | 9.35E-03 | 2.690839 | -3.36294881 | 1.349184 | 1.349184 | Up-regulated |
| SUN1 | 3.59E-04 | 3.68E-05 | 4.478224 | 1.82646045 | 1.350466 | 1.350466 | Up-regulated |
| TSC22D1 | 6.63E-03 | 2.46E-03 | 3.169072 | -2.14308343 | 1.361543 | 1.361543 | Up-regulated |
| KIAA0355 | 2.13E-02 | 1.63E-02 | 2.47462 | -3.86427771 | 1.474126 | 1.474126 | Up-regulated |
| CEP68 | 2.36E-02 | 1.87E-02 | 2.421927 | -3.98137607 | 1.480974 | 1.480974 | Up-regulated |
| FBRS | 1.29E-02 | 7.82E-03 | 2.757352 | -3.20219329 | 1.481408 | 1.481408 | Up-regulated |
| MAL | 6.04E-04 | 7.22E-05 | 4.281907 | 1.18340616 | 1.485214 | 1.485214 | Up-regulated |
| GPR18 | 1.57E-03 | 2.59E-04 | 3.897327 | -0.03248234 | 1.537295 | 1.537295 | Up-regulated |
| ADARB1 | 4.42E-04 | 4.81E-05 | 4.40085 | 1.57134632 | 1.593547 | 1.593547 | Up-regulated |
| KLHL5 | 5.68E-06 | 2.06E-07 | 5.907876 | 6.8366857 | 1.753687 | 1.753687 | Up-regulated |
| SPATC1L | 7.36E-08 | 1.13E-09 | 7.275696 | 11.91013376 | 1.921494 | 1.921494 | Up-regulated |
| RPL22 | 5.19E-09 | 4.49E-11 | 8.118134 | 15.05606883 | 2.417974 | 2.417974 | Up-regulated |
| MICAL2 | 1.32E-10 | 4.84E-13 | 9.319283 | 19.47835346 | 2.418376 | 2.418376 | Up-regulated |
