# Supplementary material for: Study on the mechanism of Shenmai injection in the treatment of sepsis
Source: J Cell Mol Med. 2024 Nov 25;28(22):e70201. doi: 10.1111/jcmm.70201 (PMC11586680; doi:10.1111/jcmm.70201)
Supplement: Supplementary file 6 — Table S3. [file JCMM-28-e70201-s009.docx]

**Supplementary Table 3 Information of sepsis disease genes**

| Gene Symbol | Relevance score | GeneCards Link |
| --- | --- | --- |
| TNF | 22.42014885 | https://www.genecards.org/cgi-bin/carddisp.pl?gene=TNF |
| IL6 | 21.4563961 | https://www.genecards.org/cgi-bin/carddisp.pl?gene=IL6 |
| IL10 | 18.39486885 | https://www.genecards.org/cgi-bin/carddisp.pl?gene=IL10 |
| CXCL8 | 17.76408958 | https://www.genecards.org/cgi-bin/carddisp.pl?gene=CXCL8 |
| TLR4 | 16.79547119 | https://www.genecards.org/cgi-bin/carddisp.pl?gene=TLR4 |
| CRP | 15.47816658 | https://www.genecards.org/cgi-bin/carddisp.pl?gene=CRP |
| IL1B | 14.00779438 | https://www.genecards.org/cgi-bin/carddisp.pl?gene=IL1B |
| HMGB1 | 13.20816135 | https://www.genecards.org/cgi-bin/carddisp.pl?gene=HMGB1 |
| TLR2 | 12.52583313 | https://www.genecards.org/cgi-bin/carddisp.pl?gene=TLR2 |
| CD14 | 11.0968256 | https://www.genecards.org/cgi-bin/carddisp.pl?gene=CD14 |
| MYD88 | 10.71153927 | https://www.genecards.org/cgi-bin/carddisp.pl?gene=MYD88 |
| TREM1 | 10.63362122 | https://www.genecards.org/cgi-bin/carddisp.pl?gene=TREM1 |
| HOTAIR | 10.25306416 | https://www.genecards.org/cgi-bin/carddisp.pl?gene=HOTAIR |
| CSF3 | 10.10919094 | https://www.genecards.org/cgi-bin/carddisp.pl?gene=CSF3 |
| SERPINC1 | 10.00205708 | https://www.genecards.org/cgi-bin/carddisp.pl?gene=SERPINC1 |
| SERPINE1 | 9.872941971 | https://www.genecards.org/cgi-bin/carddisp.pl?gene=SERPINE1 |
| ALB | 9.756422043 | https://www.genecards.org/cgi-bin/carddisp.pl?gene=ALB |
| IL18 | 9.709262848 | https://www.genecards.org/cgi-bin/carddisp.pl?gene=IL18 |
| LINC02605 | 9.303614616 | https://www.genecards.org/cgi-bin/carddisp.pl?gene=LINC02605 |
| F5 | 9.266923904 | https://www.genecards.org/cgi-bin/carddisp.pl?gene=F5 |
| MYLK | 9.055744171 | https://www.genecards.org/cgi-bin/carddisp.pl?gene=MYLK |
| ELANE | 9.023469925 | https://www.genecards.org/cgi-bin/carddisp.pl?gene=ELANE |
| F3 | 8.94912529 | https://www.genecards.org/cgi-bin/carddisp.pl?gene=F3 |
| IL1A | 8.711558342 | https://www.genecards.org/cgi-bin/carddisp.pl?gene=IL1A |
| CCL2 | 8.515014648 | https://www.genecards.org/cgi-bin/carddisp.pl?gene=CCL2 |
| ITGAM | 8.448890686 | https://www.genecards.org/cgi-bin/carddisp.pl?gene=ITGAM |
| CALCA | 8.446528435 | https://www.genecards.org/cgi-bin/carddisp.pl?gene=CALCA |
| H19 | 8.419380188 | https://www.genecards.org/cgi-bin/carddisp.pl?gene=H19 |
| MIR146B | 8.255517006 | https://www.genecards.org/cgi-bin/carddisp.pl?gene=MIR146B |
| LBP | 8.156266212 | https://www.genecards.org/cgi-bin/carddisp.pl?gene=LBP |
| MIF | 7.720649719 | https://www.genecards.org/cgi-bin/carddisp.pl?gene=MIF |
| GALT | 7.606815815 | https://www.genecards.org/cgi-bin/carddisp.pl?gene=GALT |
| TLR9 | 7.586449623 | https://www.genecards.org/cgi-bin/carddisp.pl?gene=TLR9 |
| TLR5 | 7.528318405 | https://www.genecards.org/cgi-bin/carddisp.pl?gene=TLR5 |
| MBL2 | 7.501828194 | https://www.genecards.org/cgi-bin/carddisp.pl?gene=MBL2 |
| LCN2 | 7.477340221 | https://www.genecards.org/cgi-bin/carddisp.pl?gene=LCN2 |
| IFNG | 7.349286079 | https://www.genecards.org/cgi-bin/carddisp.pl?gene=IFNG |
| CD4 | 7.185584068 | https://www.genecards.org/cgi-bin/carddisp.pl?gene=CD4 |
| IL1RN | 7.112122059 | https://www.genecards.org/cgi-bin/carddisp.pl?gene=IL1RN |
| MYH11 | 6.897387028 | https://www.genecards.org/cgi-bin/carddisp.pl?gene=MYH11 |
| THBD | 6.846528053 | https://www.genecards.org/cgi-bin/carddisp.pl?gene=THBD |
| CSF2 | 6.838051796 | https://www.genecards.org/cgi-bin/carddisp.pl?gene=CSF2 |
| LINC01672 | 6.763669014 | https://www.genecards.org/cgi-bin/carddisp.pl?gene=LINC01672 |
| CCR6 | 6.762800694 | https://www.genecards.org/cgi-bin/carddisp.pl?gene=CCR6 |
| ACTG2 | 6.740917206 | https://www.genecards.org/cgi-bin/carddisp.pl?gene=ACTG2 |
| F2 | 6.656029224 | https://www.genecards.org/cgi-bin/carddisp.pl?gene=F2 |
| LY96 | 6.636755466 | https://www.genecards.org/cgi-bin/carddisp.pl?gene=LY96 |
| ICAM1 | 6.578541279 | https://www.genecards.org/cgi-bin/carddisp.pl?gene=ICAM1 |
| SOD2-OT1 | 6.566740036 | https://www.genecards.org/cgi-bin/carddisp.pl?gene=SOD2-OT1 |
| CERNA3 | 6.51893568 | https://www.genecards.org/cgi-bin/carddisp.pl?gene=CERNA3 |
| LMOD1 | 6.496398926 | https://www.genecards.org/cgi-bin/carddisp.pl?gene=LMOD1 |
| VCAM1 | 6.39460659 | https://www.genecards.org/cgi-bin/carddisp.pl?gene=VCAM1 |
| PROCR | 6.298037529 | https://www.genecards.org/cgi-bin/carddisp.pl?gene=PROCR |
| CASP12 | 6.238653183 | https://www.genecards.org/cgi-bin/carddisp.pl?gene=CASP12 |
| NOS2 | 6.217689991 | https://www.genecards.org/cgi-bin/carddisp.pl?gene=NOS2 |
| LTA | 6.146872997 | https://www.genecards.org/cgi-bin/carddisp.pl?gene=LTA |
| BTK | 6.090130806 | https://www.genecards.org/cgi-bin/carddisp.pl?gene=BTK |
| SPINK5 | 6.016999722 | https://www.genecards.org/cgi-bin/carddisp.pl?gene=SPINK5 |
| GALM | 5.93773222 | https://www.genecards.org/cgi-bin/carddisp.pl?gene=GALM |
| RPSA | 5.929110527 | https://www.genecards.org/cgi-bin/carddisp.pl?gene=RPSA |
| MIR21 | 5.834685802 | https://www.genecards.org/cgi-bin/carddisp.pl?gene=MIR21 |
| ENSG00000275307 | 5.821548462 | https://www.genecards.org/cgi-bin/carddisp.pl?gene=ENSG00000275307 |
| ENSG00000276965 | 5.821548462 | https://www.genecards.org/cgi-bin/carddisp.pl?gene=ENSG00000276965 |
| IL1R1 | 5.8166008 | https://www.genecards.org/cgi-bin/carddisp.pl?gene=IL1R1 |
| NOD2 | 5.759204865 | https://www.genecards.org/cgi-bin/carddisp.pl?gene=NOD2 |
| PWAR1 | 5.669919968 | https://www.genecards.org/cgi-bin/carddisp.pl?gene=PWAR1 |
| IL2 | 5.616464615 | https://www.genecards.org/cgi-bin/carddisp.pl?gene=IL2 |
| FCGR3B | 5.57293272 | https://www.genecards.org/cgi-bin/carddisp.pl?gene=FCGR3B |
| CD40LG | 5.512127399 | https://www.genecards.org/cgi-bin/carddisp.pl?gene=CD40LG |
| RAC2 | 5.490653038 | https://www.genecards.org/cgi-bin/carddisp.pl?gene=RAC2 |
| NFKB1 | 5.468993664 | https://www.genecards.org/cgi-bin/carddisp.pl?gene=NFKB1 |
| MIR155 | 5.461371422 | https://www.genecards.org/cgi-bin/carddisp.pl?gene=MIR155 |
| SELE | 5.442882061 | https://www.genecards.org/cgi-bin/carddisp.pl?gene=SELE |
| PLA2G2A | 5.361628532 | https://www.genecards.org/cgi-bin/carddisp.pl?gene=PLA2G2A |
| ANGPT2 | 5.328507423 | https://www.genecards.org/cgi-bin/carddisp.pl?gene=ANGPT2 |
| IRAK1 | 5.292589188 | https://www.genecards.org/cgi-bin/carddisp.pl?gene=IRAK1 |
| TLR1 | 5.283637047 | https://www.genecards.org/cgi-bin/carddisp.pl?gene=TLR1 |
| CD8A | 5.273484707 | https://www.genecards.org/cgi-bin/carddisp.pl?gene=CD8A |
| IFNB1 | 5.269484043 | https://www.genecards.org/cgi-bin/carddisp.pl?gene=IFNB1 |
| MIR125A | 5.238754272 | https://www.genecards.org/cgi-bin/carddisp.pl?gene=MIR125A |
| MEG3 | 5.235800743 | https://www.genecards.org/cgi-bin/carddisp.pl?gene=MEG3 |
| CCL3 | 5.233223438 | https://www.genecards.org/cgi-bin/carddisp.pl?gene=CCL3 |
| TLR3 | 5.225547314 | https://www.genecards.org/cgi-bin/carddisp.pl?gene=TLR3 |
| TRAF6 | 5.076787949 | https://www.genecards.org/cgi-bin/carddisp.pl?gene=TRAF6 |
| SELL | 5.072111607 | https://www.genecards.org/cgi-bin/carddisp.pl?gene=SELL |
| NKX2-5 | 5.061098099 | https://www.genecards.org/cgi-bin/carddisp.pl?gene=NKX2-5 |
| MMP9 | 5.033742428 | https://www.genecards.org/cgi-bin/carddisp.pl?gene=MMP9 |
| MPO | 4.985769749 | https://www.genecards.org/cgi-bin/carddisp.pl?gene=MPO |
| NFE2L2 | 4.972750664 | https://www.genecards.org/cgi-bin/carddisp.pl?gene=NFE2L2 |
| MAPK14 | 4.969906807 | https://www.genecards.org/cgi-bin/carddisp.pl?gene=MAPK14 |
| IL4 | 4.957773209 | https://www.genecards.org/cgi-bin/carddisp.pl?gene=IL4 |
| NPPB | 4.949479103 | https://www.genecards.org/cgi-bin/carddisp.pl?gene=NPPB |
| PTX3 | 4.938828468 | https://www.genecards.org/cgi-bin/carddisp.pl?gene=PTX3 |
| PROC | 4.932542324 | https://www.genecards.org/cgi-bin/carddisp.pl?gene=PROC |
| MIR122 | 4.890286446 | https://www.genecards.org/cgi-bin/carddisp.pl?gene=MIR122 |
| IL17A | 4.862259388 | https://www.genecards.org/cgi-bin/carddisp.pl?gene=IL17A |
| HMOX1 | 4.825912476 | https://www.genecards.org/cgi-bin/carddisp.pl?gene=HMOX1 |
| SOD1 | 4.823636532 | https://www.genecards.org/cgi-bin/carddisp.pl?gene=SOD1 |
| FCGR1A | 4.764118195 | https://www.genecards.org/cgi-bin/carddisp.pl?gene=FCGR1A |
| FLNA | 4.763964653 | https://www.genecards.org/cgi-bin/carddisp.pl?gene=FLNA |
| IRAK4 | 4.751749516 | https://www.genecards.org/cgi-bin/carddisp.pl?gene=IRAK4 |
| LTF | 4.700494766 | https://www.genecards.org/cgi-bin/carddisp.pl?gene=LTF |
| GALK1 | 4.680478573 | https://www.genecards.org/cgi-bin/carddisp.pl?gene=GALK1 |
| GALE | 4.680478573 | https://www.genecards.org/cgi-bin/carddisp.pl?gene=GALE |
| MIR146A | 4.645521164 | https://www.genecards.org/cgi-bin/carddisp.pl?gene=MIR146A |
| ACE | 4.633337498 | https://www.genecards.org/cgi-bin/carddisp.pl?gene=ACE |
| GAS5 | 4.618186951 | https://www.genecards.org/cgi-bin/carddisp.pl?gene=GAS5 |
| CD274 | 4.582570076 | https://www.genecards.org/cgi-bin/carddisp.pl?gene=CD274 |
| BPI | 4.538882732 | https://www.genecards.org/cgi-bin/carddisp.pl?gene=BPI |
| ABCA12 | 4.523760319 | https://www.genecards.org/cgi-bin/carddisp.pl?gene=ABCA12 |
| VEGFA | 4.518950462 | https://www.genecards.org/cgi-bin/carddisp.pl?gene=VEGFA |
| CXCL2 | 4.518579006 | https://www.genecards.org/cgi-bin/carddisp.pl?gene=CXCL2 |
| ADM | 4.512655735 | https://www.genecards.org/cgi-bin/carddisp.pl?gene=ADM |
| ACE2 | 4.457019329 | https://www.genecards.org/cgi-bin/carddisp.pl?gene=ACE2 |
| IL2RA | 4.438714027 | https://www.genecards.org/cgi-bin/carddisp.pl?gene=IL2RA |
| MYL9 | 4.405590057 | https://www.genecards.org/cgi-bin/carddisp.pl?gene=MYL9 |
| MIR126 | 4.373920441 | https://www.genecards.org/cgi-bin/carddisp.pl?gene=MIR126 |
| SELP | 4.37206459 | https://www.genecards.org/cgi-bin/carddisp.pl?gene=SELP |
| TMX2-CTNND1 | 4.352546692 | https://www.genecards.org/cgi-bin/carddisp.pl?gene=TMX2-CTNND1 |
| ADAMTS13 | 4.347048759 | https://www.genecards.org/cgi-bin/carddisp.pl?gene=ADAMTS13 |
| IL13 | 4.292468548 | https://www.genecards.org/cgi-bin/carddisp.pl?gene=IL13 |
| HBB | 4.284498215 | https://www.genecards.org/cgi-bin/carddisp.pl?gene=HBB |
| PDCD1 | 4.270773888 | https://www.genecards.org/cgi-bin/carddisp.pl?gene=PDCD1 |
| CLMP | 4.268550396 | https://www.genecards.org/cgi-bin/carddisp.pl?gene=CLMP |
| C5AR2 | 4.258349419 | https://www.genecards.org/cgi-bin/carddisp.pl?gene=C5AR2 |
| MIR16-1 | 4.203838348 | https://www.genecards.org/cgi-bin/carddisp.pl?gene=MIR16-1 |
| MAP3K7 | 4.197896004 | https://www.genecards.org/cgi-bin/carddisp.pl?gene=MAP3K7 |
| TIMP1 | 4.178701401 | https://www.genecards.org/cgi-bin/carddisp.pl?gene=TIMP1 |
| GZMB | 4.15747261 | https://www.genecards.org/cgi-bin/carddisp.pl?gene=GZMB |
| MIR16-2 | 4.14447546 | https://www.genecards.org/cgi-bin/carddisp.pl?gene=MIR16-2 |
| NLRP3 | 4.138355255 | https://www.genecards.org/cgi-bin/carddisp.pl?gene=NLRP3 |
| SNHG16 | 4.135272503 | https://www.genecards.org/cgi-bin/carddisp.pl?gene=SNHG16 |
| ICOSLG | 4.126834393 | https://www.genecards.org/cgi-bin/carddisp.pl?gene=ICOSLG |
| VWF | 4.101391792 | https://www.genecards.org/cgi-bin/carddisp.pl?gene=VWF |
| IRAK3 | 4.034486294 | https://www.genecards.org/cgi-bin/carddisp.pl?gene=IRAK3 |
| NFKB2 | 4.02821207 | https://www.genecards.org/cgi-bin/carddisp.pl?gene=NFKB2 |
| HULC | 4.017068863 | https://www.genecards.org/cgi-bin/carddisp.pl?gene=HULC |
| MNX1 | 4.010404587 | https://www.genecards.org/cgi-bin/carddisp.pl?gene=MNX1 |
| MIR493HG | 4.001262665 | https://www.genecards.org/cgi-bin/carddisp.pl?gene=MIR493HG |
| ENSG00000276919 | 4.001262665 | https://www.genecards.org/cgi-bin/carddisp.pl?gene=ENSG00000276919 |
| SERAC1 | 3.999498129 | https://www.genecards.org/cgi-bin/carddisp.pl?gene=SERAC1 |
| CCL4 | 3.998503923 | https://www.genecards.org/cgi-bin/carddisp.pl?gene=CCL4 |
| PLA2G7 | 3.992495298 | https://www.genecards.org/cgi-bin/carddisp.pl?gene=PLA2G7 |
| KRT10 | 3.989461899 | https://www.genecards.org/cgi-bin/carddisp.pl?gene=KRT10 |
| MASP2 | 3.984537601 | https://www.genecards.org/cgi-bin/carddisp.pl?gene=MASP2 |
| CPS1 | 3.964564085 | https://www.genecards.org/cgi-bin/carddisp.pl?gene=CPS1 |
| TF | 3.938699007 | https://www.genecards.org/cgi-bin/carddisp.pl?gene=TF |
| PGR-AS1 | 3.875538349 | https://www.genecards.org/cgi-bin/carddisp.pl?gene=PGR-AS1 |
| ITGB2 | 3.872359276 | https://www.genecards.org/cgi-bin/carddisp.pl?gene=ITGB2 |
| CASP3 | 3.865695953 | https://www.genecards.org/cgi-bin/carddisp.pl?gene=CASP3 |
| THPO | 3.855608702 | https://www.genecards.org/cgi-bin/carddisp.pl?gene=THPO |
| KCNQ1OT1 | 3.841905355 | https://www.genecards.org/cgi-bin/carddisp.pl?gene=KCNQ1OT1 |
| NOS3 | 3.820085526 | https://www.genecards.org/cgi-bin/carddisp.pl?gene=NOS3 |
| BDNF-AS | 3.817754984 | https://www.genecards.org/cgi-bin/carddisp.pl?gene=BDNF-AS |
| IDO1 | 3.796086788 | https://www.genecards.org/cgi-bin/carddisp.pl?gene=IDO1 |
| CP | 3.786608934 | https://www.genecards.org/cgi-bin/carddisp.pl?gene=CP |
| PVT1 | 3.747030735 | https://www.genecards.org/cgi-bin/carddisp.pl?gene=PVT1 |
| MIR150 | 3.720079422 | https://www.genecards.org/cgi-bin/carddisp.pl?gene=MIR150 |
| MIR125B2 | 3.718486309 | https://www.genecards.org/cgi-bin/carddisp.pl?gene=MIR125B2 |
| GSDMD | 3.711807966 | https://www.genecards.org/cgi-bin/carddisp.pl?gene=GSDMD |
| EPO | 3.707824707 | https://www.genecards.org/cgi-bin/carddisp.pl?gene=EPO |
| MAPK8 | 3.685587168 | https://www.genecards.org/cgi-bin/carddisp.pl?gene=MAPK8 |
| PLEC | 3.671973705 | https://www.genecards.org/cgi-bin/carddisp.pl?gene=PLEC |
| PLG | 3.669275761 | https://www.genecards.org/cgi-bin/carddisp.pl?gene=PLG |
| CXCR2 | 3.661672592 | https://www.genecards.org/cgi-bin/carddisp.pl?gene=CXCR2 |
| KRT1 | 3.647407055 | https://www.genecards.org/cgi-bin/carddisp.pl?gene=KRT1 |
| FAS | 3.639813185 | https://www.genecards.org/cgi-bin/carddisp.pl?gene=FAS |
| FCGR2A | 3.636765957 | https://www.genecards.org/cgi-bin/carddisp.pl?gene=FCGR2A |
| TLR7 | 3.615483761 | https://www.genecards.org/cgi-bin/carddisp.pl?gene=TLR7 |
| MIR125B1 | 3.611689568 | https://www.genecards.org/cgi-bin/carddisp.pl?gene=MIR125B1 |
| GPT | 3.599738598 | https://www.genecards.org/cgi-bin/carddisp.pl?gene=GPT |
| FOXP3 | 3.592576981 | https://www.genecards.org/cgi-bin/carddisp.pl?gene=FOXP3 |
| MALAT1 | 3.584370852 | https://www.genecards.org/cgi-bin/carddisp.pl?gene=MALAT1 |
| MIR155HG | 3.583505154 | https://www.genecards.org/cgi-bin/carddisp.pl?gene=MIR155HG |
| XIAP | 3.57881403 | https://www.genecards.org/cgi-bin/carddisp.pl?gene=XIAP |
| LOC112042785 | 3.578736782 | https://www.genecards.org/cgi-bin/carddisp.pl?gene=LOC112042785 |
| C5AR1 | 3.576799393 | https://www.genecards.org/cgi-bin/carddisp.pl?gene=C5AR1 |
| XIST | 3.5717237 | https://www.genecards.org/cgi-bin/carddisp.pl?gene=XIST |
| ZFAS1 | 3.566480637 | https://www.genecards.org/cgi-bin/carddisp.pl?gene=ZFAS1 |
| EDN1 | 3.55684185 | https://www.genecards.org/cgi-bin/carddisp.pl?gene=EDN1 |
| BCL2 | 3.519965172 | https://www.genecards.org/cgi-bin/carddisp.pl?gene=BCL2 |
| CXCL10 | 3.494087934 | https://www.genecards.org/cgi-bin/carddisp.pl?gene=CXCL10 |
| STAT3 | 3.488768339 | https://www.genecards.org/cgi-bin/carddisp.pl?gene=STAT3 |
| FABP2 | 3.46065402 | https://www.genecards.org/cgi-bin/carddisp.pl?gene=FABP2 |
| MIAT | 3.455434799 | https://www.genecards.org/cgi-bin/carddisp.pl?gene=MIAT |
| TAB2 | 3.450863123 | https://www.genecards.org/cgi-bin/carddisp.pl?gene=TAB2 |
| PRTN3 | 3.438145638 | https://www.genecards.org/cgi-bin/carddisp.pl?gene=PRTN3 |
| MIR211 | 3.437768459 | https://www.genecards.org/cgi-bin/carddisp.pl?gene=MIR211 |
| IFNA1 | 3.433571815 | https://www.genecards.org/cgi-bin/carddisp.pl?gene=IFNA1 |
| EDNRB | 3.43356657 | https://www.genecards.org/cgi-bin/carddisp.pl?gene=EDNRB |
| MIR9-1 | 3.432723999 | https://www.genecards.org/cgi-bin/carddisp.pl?gene=MIR9-1 |
| ITGAX | 3.415096998 | https://www.genecards.org/cgi-bin/carddisp.pl?gene=ITGAX |
| CTLA4 | 3.411732197 | https://www.genecards.org/cgi-bin/carddisp.pl?gene=CTLA4 |
| NEAT1 | 3.400866747 | https://www.genecards.org/cgi-bin/carddisp.pl?gene=NEAT1 |
| AGER | 3.400861979 | https://www.genecards.org/cgi-bin/carddisp.pl?gene=AGER |
| IKBKG | 3.399188519 | https://www.genecards.org/cgi-bin/carddisp.pl?gene=IKBKG |
| TRA-TGC7-1 | 3.395115376 | https://www.genecards.org/cgi-bin/carddisp.pl?gene=TRA-TGC7-1 |
| TRA-TGC5-1 | 3.395115376 | https://www.genecards.org/cgi-bin/carddisp.pl?gene=TRA-TGC5-1 |
| LACTB | 3.394562721 | https://www.genecards.org/cgi-bin/carddisp.pl?gene=LACTB |
| COL7A1 | 3.394192219 | https://www.genecards.org/cgi-bin/carddisp.pl?gene=COL7A1 |
| MNX1-AS2 | 3.380908728 | https://www.genecards.org/cgi-bin/carddisp.pl?gene=MNX1-AS2 |
| ENSG00000277577 | 3.37904501 | https://www.genecards.org/cgi-bin/carddisp.pl?gene=ENSG00000277577 |
| PRF1 | 3.376131773 | https://www.genecards.org/cgi-bin/carddisp.pl?gene=PRF1 |
| CASC2 | 3.371594429 | https://www.genecards.org/cgi-bin/carddisp.pl?gene=CASC2 |
| LINC00504 | 3.370393753 | https://www.genecards.org/cgi-bin/carddisp.pl?gene=LINC00504 |
| TTTY10 | 3.370393753 | https://www.genecards.org/cgi-bin/carddisp.pl?gene=TTTY10 |
| ARF4-AS1 | 3.370393753 | https://www.genecards.org/cgi-bin/carddisp.pl?gene=ARF4-AS1 |
| LINC01258 | 3.370393753 | https://www.genecards.org/cgi-bin/carddisp.pl?gene=LINC01258 |
| LINC01725 | 3.370393753 | https://www.genecards.org/cgi-bin/carddisp.pl?gene=LINC01725 |
| LINC02882 | 3.370393753 | https://www.genecards.org/cgi-bin/carddisp.pl?gene=LINC02882 |
| LINC02955 | 3.370393753 | https://www.genecards.org/cgi-bin/carddisp.pl?gene=LINC02955 |
| LINC02895 | 3.370393753 | https://www.genecards.org/cgi-bin/carddisp.pl?gene=LINC02895 |
| ENSG00000230490 | 3.370393753 | https://www.genecards.org/cgi-bin/carddisp.pl?gene=ENSG00000230490 |
| ENSG00000245768 | 3.370393753 | https://www.genecards.org/cgi-bin/carddisp.pl?gene=ENSG00000245768 |
| ENSG00000250519 | 3.370393753 | https://www.genecards.org/cgi-bin/carddisp.pl?gene=ENSG00000250519 |
| ENSG00000258081 | 3.370393753 | https://www.genecards.org/cgi-bin/carddisp.pl?gene=ENSG00000258081 |
| ENSG00000235450 | 3.370393753 | https://www.genecards.org/cgi-bin/carddisp.pl?gene=ENSG00000235450 |
| ENSG00000251216 | 3.370393753 | https://www.genecards.org/cgi-bin/carddisp.pl?gene=ENSG00000251216 |
| LOC101927560 | 3.370393753 | https://www.genecards.org/cgi-bin/carddisp.pl?gene=LOC101927560 |
| ENSG00000253288 | 3.370393753 | https://www.genecards.org/cgi-bin/carddisp.pl?gene=ENSG00000253288 |
| lnc-IQCM-2 | 3.370393753 | https://www.genecards.org/cgi-bin/carddisp.pl?gene=lnc-IQCM-2 |
| lnc-HMGXB4-8 | 3.370393753 | https://www.genecards.org/cgi-bin/carddisp.pl?gene=lnc-HMGXB4-8 |
| MK280073-023 | 3.370393753 | https://www.genecards.org/cgi-bin/carddisp.pl?gene=MK280073-023 |
| MK280073-058 | 3.370393753 | https://www.genecards.org/cgi-bin/carddisp.pl?gene=MK280073-058 |
| MK280073-197 | 3.370393753 | https://www.genecards.org/cgi-bin/carddisp.pl?gene=MK280073-197 |
| MK280073-346 | 3.370393753 | https://www.genecards.org/cgi-bin/carddisp.pl?gene=MK280073-346 |
| MK280073-501 | 3.370393753 | https://www.genecards.org/cgi-bin/carddisp.pl?gene=MK280073-501 |
| MK280073-008 | 3.370393753 | https://www.genecards.org/cgi-bin/carddisp.pl?gene=MK280073-008 |
| MK280073-020 | 3.370393753 | https://www.genecards.org/cgi-bin/carddisp.pl?gene=MK280073-020 |
| MK280073-022 | 3.370393753 | https://www.genecards.org/cgi-bin/carddisp.pl?gene=MK280073-022 |
| MK280073-049 | 3.370393753 | https://www.genecards.org/cgi-bin/carddisp.pl?gene=MK280073-049 |
| MK280073-055 | 3.370393753 | https://www.genecards.org/cgi-bin/carddisp.pl?gene=MK280073-055 |
| MK280073-060 | 3.370393753 | https://www.genecards.org/cgi-bin/carddisp.pl?gene=MK280073-060 |
| MK280073-063 | 3.370393753 | https://www.genecards.org/cgi-bin/carddisp.pl?gene=MK280073-063 |
| MK280073-083 | 3.370393753 | https://www.genecards.org/cgi-bin/carddisp.pl?gene=MK280073-083 |
| MK280073-093 | 3.370393753 | https://www.genecards.org/cgi-bin/carddisp.pl?gene=MK280073-093 |
| MK280073-120 | 3.370393753 | https://www.genecards.org/cgi-bin/carddisp.pl?gene=MK280073-120 |
| MK280073-121 | 3.370393753 | https://www.genecards.org/cgi-bin/carddisp.pl?gene=MK280073-121 |
| MK280073-164 | 3.370393753 | https://www.genecards.org/cgi-bin/carddisp.pl?gene=MK280073-164 |
| MK280073-202 | 3.370393753 | https://www.genecards.org/cgi-bin/carddisp.pl?gene=MK280073-202 |
| MK280073-300 | 3.370393753 | https://www.genecards.org/cgi-bin/carddisp.pl?gene=MK280073-300 |
| MK280073-334 | 3.370393753 | https://www.genecards.org/cgi-bin/carddisp.pl?gene=MK280073-334 |
| MK280073-475 | 3.370393753 | https://www.genecards.org/cgi-bin/carddisp.pl?gene=MK280073-475 |
| MK280073-493 | 3.370393753 | https://www.genecards.org/cgi-bin/carddisp.pl?gene=MK280073-493 |
| MK280073-510 | 3.370393753 | https://www.genecards.org/cgi-bin/carddisp.pl?gene=MK280073-510 |
| MK280073-521 | 3.370393753 | https://www.genecards.org/cgi-bin/carddisp.pl?gene=MK280073-521 |
| MK280073-571 | 3.370393753 | https://www.genecards.org/cgi-bin/carddisp.pl?gene=MK280073-571 |
| MK280073-623 | 3.370393753 | https://www.genecards.org/cgi-bin/carddisp.pl?gene=MK280073-623 |
| MK280073-007 | 3.370393753 | https://www.genecards.org/cgi-bin/carddisp.pl?gene=MK280073-007 |
| MK280073-013 | 3.370393753 | https://www.genecards.org/cgi-bin/carddisp.pl?gene=MK280073-013 |
| MK280073-018 | 3.370393753 | https://www.genecards.org/cgi-bin/carddisp.pl?gene=MK280073-018 |
| MK280073-025 | 3.370393753 | https://www.genecards.org/cgi-bin/carddisp.pl?gene=MK280073-025 |
| MK280073-027 | 3.370393753 | https://www.genecards.org/cgi-bin/carddisp.pl?gene=MK280073-027 |
| MK280073-028 | 3.370393753 | https://www.genecards.org/cgi-bin/carddisp.pl?gene=MK280073-028 |
| MK280073-030 | 3.370393753 | https://www.genecards.org/cgi-bin/carddisp.pl?gene=MK280073-030 |
| MK280073-040 | 3.370393753 | https://www.genecards.org/cgi-bin/carddisp.pl?gene=MK280073-040 |
| MK280073-044 | 3.370393753 | https://www.genecards.org/cgi-bin/carddisp.pl?gene=MK280073-044 |
| MK280073-052 | 3.370393753 | https://www.genecards.org/cgi-bin/carddisp.pl?gene=MK280073-052 |
| MK280073-059 | 3.370393753 | https://www.genecards.org/cgi-bin/carddisp.pl?gene=MK280073-059 |
| MK280073-072 | 3.370393753 | https://www.genecards.org/cgi-bin/carddisp.pl?gene=MK280073-072 |
| MK280073-081 | 3.370393753 | https://www.genecards.org/cgi-bin/carddisp.pl?gene=MK280073-081 |
| MK280073-082 | 3.370393753 | https://www.genecards.org/cgi-bin/carddisp.pl?gene=MK280073-082 |
| MK280073-090 | 3.370393753 | https://www.genecards.org/cgi-bin/carddisp.pl?gene=MK280073-090 |
| MK280073-091 | 3.370393753 | https://www.genecards.org/cgi-bin/carddisp.pl?gene=MK280073-091 |
| MK280073-115 | 3.370393753 | https://www.genecards.org/cgi-bin/carddisp.pl?gene=MK280073-115 |
| MK280073-117 | 3.370393753 | https://www.genecards.org/cgi-bin/carddisp.pl?gene=MK280073-117 |
| MK280073-123 | 3.370393753 | https://www.genecards.org/cgi-bin/carddisp.pl?gene=MK280073-123 |
| MK280073-140 | 3.370393753 | https://www.genecards.org/cgi-bin/carddisp.pl?gene=MK280073-140 |
| MK280073-149 | 3.370393753 | https://www.genecards.org/cgi-bin/carddisp.pl?gene=MK280073-149 |
| MK280073-150 | 3.370393753 | https://www.genecards.org/cgi-bin/carddisp.pl?gene=MK280073-150 |
| MK280073-153 | 3.370393753 | https://www.genecards.org/cgi-bin/carddisp.pl?gene=MK280073-153 |
| MK280073-175 | 3.370393753 | https://www.genecards.org/cgi-bin/carddisp.pl?gene=MK280073-175 |
| MK280073-176 | 3.370393753 | https://www.genecards.org/cgi-bin/carddisp.pl?gene=MK280073-176 |
| MK280073-180 | 3.370393753 | https://www.genecards.org/cgi-bin/carddisp.pl?gene=MK280073-180 |
| MK280073-182 | 3.370393753 | https://www.genecards.org/cgi-bin/carddisp.pl?gene=MK280073-182 |
| MK280073-199 | 3.370393753 | https://www.genecards.org/cgi-bin/carddisp.pl?gene=MK280073-199 |
| MK280073-203 | 3.370393753 | https://www.genecards.org/cgi-bin/carddisp.pl?gene=MK280073-203 |
| MK280073-205 | 3.370393753 | https://www.genecards.org/cgi-bin/carddisp.pl?gene=MK280073-205 |
| MK280073-206 | 3.370393753 | https://www.genecards.org/cgi-bin/carddisp.pl?gene=MK280073-206 |
| MK280073-207 | 3.370393753 | https://www.genecards.org/cgi-bin/carddisp.pl?gene=MK280073-207 |
| MK280073-216 | 3.370393753 | https://www.genecards.org/cgi-bin/carddisp.pl?gene=MK280073-216 |
| MK280073-243 | 3.370393753 | https://www.genecards.org/cgi-bin/carddisp.pl?gene=MK280073-243 |
| MK280073-273 | 3.370393753 | https://www.genecards.org/cgi-bin/carddisp.pl?gene=MK280073-273 |
| MK280073-283 | 3.370393753 | https://www.genecards.org/cgi-bin/carddisp.pl?gene=MK280073-283 |
| MK280073-295 | 3.370393753 | https://www.genecards.org/cgi-bin/carddisp.pl?gene=MK280073-295 |
| MK280073-296 | 3.370393753 | https://www.genecards.org/cgi-bin/carddisp.pl?gene=MK280073-296 |
| MK280073-351 | 3.370393753 | https://www.genecards.org/cgi-bin/carddisp.pl?gene=MK280073-351 |
| MK280073-353 | 3.370393753 | https://www.genecards.org/cgi-bin/carddisp.pl?gene=MK280073-353 |
| MK280073-354 | 3.370393753 | https://www.genecards.org/cgi-bin/carddisp.pl?gene=MK280073-354 |
| MK280073-359 | 3.370393753 | https://www.genecards.org/cgi-bin/carddisp.pl?gene=MK280073-359 |
| MK280073-389 | 3.370393753 | https://www.genecards.org/cgi-bin/carddisp.pl?gene=MK280073-389 |
| MK280073-453 | 3.370393753 | https://www.genecards.org/cgi-bin/carddisp.pl?gene=MK280073-453 |
| MK280073-456 | 3.370393753 | https://www.genecards.org/cgi-bin/carddisp.pl?gene=MK280073-456 |
| MK280073-460 | 3.370393753 | https://www.genecards.org/cgi-bin/carddisp.pl?gene=MK280073-460 |
| MK280073-461 | 3.370393753 | https://www.genecards.org/cgi-bin/carddisp.pl?gene=MK280073-461 |
| MK280073-464 | 3.370393753 | https://www.genecards.org/cgi-bin/carddisp.pl?gene=MK280073-464 |
| MK280073-468 | 3.370393753 | https://www.genecards.org/cgi-bin/carddisp.pl?gene=MK280073-468 |
| MK280073-470 | 3.370393753 | https://www.genecards.org/cgi-bin/carddisp.pl?gene=MK280073-470 |
| MK280073-473 | 3.370393753 | https://www.genecards.org/cgi-bin/carddisp.pl?gene=MK280073-473 |
| MK280073-490 | 3.370393753 | https://www.genecards.org/cgi-bin/carddisp.pl?gene=MK280073-490 |
| MK280073-513 | 3.370393753 | https://www.genecards.org/cgi-bin/carddisp.pl?gene=MK280073-513 |
| MK280073-519 | 3.370393753 | https://www.genecards.org/cgi-bin/carddisp.pl?gene=MK280073-519 |
| MK280073-522 | 3.370393753 | https://www.genecards.org/cgi-bin/carddisp.pl?gene=MK280073-522 |
| MK280073-523 | 3.370393753 | https://www.genecards.org/cgi-bin/carddisp.pl?gene=MK280073-523 |
| MK280073-524 | 3.370393753 | https://www.genecards.org/cgi-bin/carddisp.pl?gene=MK280073-524 |
| MK280073-525 | 3.370393753 | https://www.genecards.org/cgi-bin/carddisp.pl?gene=MK280073-525 |
| MK280073-527 | 3.370393753 | https://www.genecards.org/cgi-bin/carddisp.pl?gene=MK280073-527 |
| MK280073-533 | 3.370393753 | https://www.genecards.org/cgi-bin/carddisp.pl?gene=MK280073-533 |
| MK280073-544 | 3.370393753 | https://www.genecards.org/cgi-bin/carddisp.pl?gene=MK280073-544 |
| MK280073-557 | 3.370393753 | https://www.genecards.org/cgi-bin/carddisp.pl?gene=MK280073-557 |
| MK280073-561 | 3.370393753 | https://www.genecards.org/cgi-bin/carddisp.pl?gene=MK280073-561 |
| MK280073-586 | 3.370393753 | https://www.genecards.org/cgi-bin/carddisp.pl?gene=MK280073-586 |
| MK280073-609 | 3.370393753 | https://www.genecards.org/cgi-bin/carddisp.pl?gene=MK280073-609 |
| MK280073-621 | 3.370393753 | https://www.genecards.org/cgi-bin/carddisp.pl?gene=MK280073-621 |
| MK280073-001 | 3.370393753 | https://www.genecards.org/cgi-bin/carddisp.pl?gene=MK280073-001 |
| MK280073-002 | 3.370393753 | https://www.genecards.org/cgi-bin/carddisp.pl?gene=MK280073-002 |
| MK280073-003 | 3.370393753 | https://www.genecards.org/cgi-bin/carddisp.pl?gene=MK280073-003 |
| MK280073-004 | 3.370393753 | https://www.genecards.org/cgi-bin/carddisp.pl?gene=MK280073-004 |
| MK280073-005 | 3.370393753 | https://www.genecards.org/cgi-bin/carddisp.pl?gene=MK280073-005 |
| MK280073-006 | 3.370393753 | https://www.genecards.org/cgi-bin/carddisp.pl?gene=MK280073-006 |
| MK280073-009 | 3.370393753 | https://www.genecards.org/cgi-bin/carddisp.pl?gene=MK280073-009 |
| MK280073-010 | 3.370393753 | https://www.genecards.org/cgi-bin/carddisp.pl?gene=MK280073-010 |
| MK280073-011 | 3.370393753 | https://www.genecards.org/cgi-bin/carddisp.pl?gene=MK280073-011 |
| MK280073-012 | 3.370393753 | https://www.genecards.org/cgi-bin/carddisp.pl?gene=MK280073-012 |
| MK280073-014 | 3.370393753 | https://www.genecards.org/cgi-bin/carddisp.pl?gene=MK280073-014 |
| MK280073-015 | 3.370393753 | https://www.genecards.org/cgi-bin/carddisp.pl?gene=MK280073-015 |
| MK280073-016 | 3.370393753 | https://www.genecards.org/cgi-bin/carddisp.pl?gene=MK280073-016 |
| MK280073-017 | 3.370393753 | https://www.genecards.org/cgi-bin/carddisp.pl?gene=MK280073-017 |
| MK280073-019 | 3.370393753 | https://www.genecards.org/cgi-bin/carddisp.pl?gene=MK280073-019 |
| MK280073-021 | 3.370393753 | https://www.genecards.org/cgi-bin/carddisp.pl?gene=MK280073-021 |
| MK280073-024 | 3.370393753 | https://www.genecards.org/cgi-bin/carddisp.pl?gene=MK280073-024 |
| MK280073-026 | 3.370393753 | https://www.genecards.org/cgi-bin/carddisp.pl?gene=MK280073-026 |
| MK280073-029 | 3.370393753 | https://www.genecards.org/cgi-bin/carddisp.pl?gene=MK280073-029 |
| MK280073-031 | 3.370393753 | https://www.genecards.org/cgi-bin/carddisp.pl?gene=MK280073-031 |
| MK280073-032 | 3.370393753 | https://www.genecards.org/cgi-bin/carddisp.pl?gene=MK280073-032 |
| MK280073-033 | 3.370393753 | https://www.genecards.org/cgi-bin/carddisp.pl?gene=MK280073-033 |
| MK280073-034 | 3.370393753 | https://www.genecards.org/cgi-bin/carddisp.pl?gene=MK280073-034 |
| MK280073-035 | 3.370393753 | https://www.genecards.org/cgi-bin/carddisp.pl?gene=MK280073-035 |
| MK280073-036 | 3.370393753 | https://www.genecards.org/cgi-bin/carddisp.pl?gene=MK280073-036 |
| MK280073-037 | 3.370393753 | https://www.genecards.org/cgi-bin/carddisp.pl?gene=MK280073-037 |
| MK280073-038 | 3.370393753 | https://www.genecards.org/cgi-bin/carddisp.pl?gene=MK280073-038 |
| MK280073-039 | 3.370393753 | https://www.genecards.org/cgi-bin/carddisp.pl?gene=MK280073-039 |
| MK280073-041 | 3.370393753 | https://www.genecards.org/cgi-bin/carddisp.pl?gene=MK280073-041 |
| MK280073-042 | 3.370393753 | https://www.genecards.org/cgi-bin/carddisp.pl?gene=MK280073-042 |
| MK280073-043 | 3.370393753 | https://www.genecards.org/cgi-bin/carddisp.pl?gene=MK280073-043 |
| MK280073-045 | 3.370393753 | https://www.genecards.org/cgi-bin/carddisp.pl?gene=MK280073-045 |
| MK280073-046 | 3.370393753 | https://www.genecards.org/cgi-bin/carddisp.pl?gene=MK280073-046 |
| MK280073-047 | 3.370393753 | https://www.genecards.org/cgi-bin/carddisp.pl?gene=MK280073-047 |
| MK280073-048 | 3.370393753 | https://www.genecards.org/cgi-bin/carddisp.pl?gene=MK280073-048 |
| MK280073-050 | 3.370393753 | https://www.genecards.org/cgi-bin/carddisp.pl?gene=MK280073-050 |
| MK280073-051 | 3.370393753 | https://www.genecards.org/cgi-bin/carddisp.pl?gene=MK280073-051 |
| MK280073-053 | 3.370393753 | https://www.genecards.org/cgi-bin/carddisp.pl?gene=MK280073-053 |
| MK280073-054 | 3.370393753 | https://www.genecards.org/cgi-bin/carddisp.pl?gene=MK280073-054 |
| MK280073-056 | 3.370393753 | https://www.genecards.org/cgi-bin/carddisp.pl?gene=MK280073-056 |
| MK280073-057 | 3.370393753 | https://www.genecards.org/cgi-bin/carddisp.pl?gene=MK280073-057 |
| MK280073-061 | 3.370393753 | https://www.genecards.org/cgi-bin/carddisp.pl?gene=MK280073-061 |
| MK280073-062 | 3.370393753 | https://www.genecards.org/cgi-bin/carddisp.pl?gene=MK280073-062 |
| MK280073-064 | 3.370393753 | https://www.genecards.org/cgi-bin/carddisp.pl?gene=MK280073-064 |
| MK280073-065 | 3.370393753 | https://www.genecards.org/cgi-bin/carddisp.pl?gene=MK280073-065 |
| MK280073-066 | 3.370393753 | https://www.genecards.org/cgi-bin/carddisp.pl?gene=MK280073-066 |
| MK280073-068 | 3.370393753 | https://www.genecards.org/cgi-bin/carddisp.pl?gene=MK280073-068 |
| MK280073-069 | 3.370393753 | https://www.genecards.org/cgi-bin/carddisp.pl?gene=MK280073-069 |
| MK280073-070 | 3.370393753 | https://www.genecards.org/cgi-bin/carddisp.pl?gene=MK280073-070 |
| MK280073-071 | 3.370393753 | https://www.genecards.org/cgi-bin/carddisp.pl?gene=MK280073-071 |
| MK280073-073 | 3.370393753 | https://www.genecards.org/cgi-bin/carddisp.pl?gene=MK280073-073 |
| MK280073-074 | 3.370393753 | https://www.genecards.org/cgi-bin/carddisp.pl?gene=MK280073-074 |
| MK280073-075 | 3.370393753 | https://www.genecards.org/cgi-bin/carddisp.pl?gene=MK280073-075 |
| MK280073-076 | 3.370393753 | https://www.genecards.org/cgi-bin/carddisp.pl?gene=MK280073-076 |
| MK280073-077 | 3.370393753 | https://www.genecards.org/cgi-bin/carddisp.pl?gene=MK280073-077 |
| MK280073-078 | 3.370393753 | https://www.genecards.org/cgi-bin/carddisp.pl?gene=MK280073-078 |
| MK280073-079 | 3.370393753 | https://www.genecards.org/cgi-bin/carddisp.pl?gene=MK280073-079 |
| MK280073-080 | 3.370393753 | https://www.genecards.org/cgi-bin/carddisp.pl?gene=MK280073-080 |
| MK280073-084 | 3.370393753 | https://www.genecards.org/cgi-bin/carddisp.pl?gene=MK280073-084 |
| MK280073-085 | 3.370393753 | https://www.genecards.org/cgi-bin/carddisp.pl?gene=MK280073-085 |
| MK280073-086 | 3.370393753 | https://www.genecards.org/cgi-bin/carddisp.pl?gene=MK280073-086 |
| MK280073-087 | 3.370393753 | https://www.genecards.org/cgi-bin/carddisp.pl?gene=MK280073-087 |
| MK280073-088 | 3.370393753 | https://www.genecards.org/cgi-bin/carddisp.pl?gene=MK280073-088 |
| MK280073-089 | 3.370393753 | https://www.genecards.org/cgi-bin/carddisp.pl?gene=MK280073-089 |
| MK280073-092 | 3.370393753 | https://www.genecards.org/cgi-bin/carddisp.pl?gene=MK280073-092 |
| MK280073-094 | 3.370393753 | https://www.genecards.org/cgi-bin/carddisp.pl?gene=MK280073-094 |
| MK280073-095 | 3.370393753 | https://www.genecards.org/cgi-bin/carddisp.pl?gene=MK280073-095 |
| MK280073-096 | 3.370393753 | https://www.genecards.org/cgi-bin/carddisp.pl?gene=MK280073-096 |
| MK280073-097 | 3.370393753 | https://www.genecards.org/cgi-bin/carddisp.pl?gene=MK280073-097 |
| MK280073-098 | 3.370393753 | https://www.genecards.org/cgi-bin/carddisp.pl?gene=MK280073-098 |
| MK280073-101 | 3.370393753 | https://www.genecards.org/cgi-bin/carddisp.pl?gene=MK280073-101 |
| MK280073-102 | 3.370393753 | https://www.genecards.org/cgi-bin/carddisp.pl?gene=MK280073-102 |
| MK280073-103 | 3.370393753 | https://www.genecards.org/cgi-bin/carddisp.pl?gene=MK280073-103 |
| MK280073-104 | 3.370393753 | https://www.genecards.org/cgi-bin/carddisp.pl?gene=MK280073-104 |
| MK280073-105 | 3.370393753 | https://www.genecards.org/cgi-bin/carddisp.pl?gene=MK280073-105 |
| MK280073-106 | 3.370393753 | https://www.genecards.org/cgi-bin/carddisp.pl?gene=MK280073-106 |
| MK280073-107 | 3.370393753 | https://www.genecards.org/cgi-bin/carddisp.pl?gene=MK280073-107 |
| MK280073-108 | 3.370393753 | https://www.genecards.org/cgi-bin/carddisp.pl?gene=MK280073-108 |
| MK280073-109 | 3.370393753 | https://www.genecards.org/cgi-bin/carddisp.pl?gene=MK280073-109 |
| MK280073-110 | 3.370393753 | https://www.genecards.org/cgi-bin/carddisp.pl?gene=MK280073-110 |
| MK280073-111 | 3.370393753 | https://www.genecards.org/cgi-bin/carddisp.pl?gene=MK280073-111 |
| MK280073-112 | 3.370393753 | https://www.genecards.org/cgi-bin/carddisp.pl?gene=MK280073-112 |
| MK280073-113 | 3.370393753 | https://www.genecards.org/cgi-bin/carddisp.pl?gene=MK280073-113 |
| MK280073-114 | 3.370393753 | https://www.genecards.org/cgi-bin/carddisp.pl?gene=MK280073-114 |
| MK280073-116 | 3.370393753 | https://www.genecards.org/cgi-bin/carddisp.pl?gene=MK280073-116 |
| MK280073-118 | 3.370393753 | https://www.genecards.org/cgi-bin/carddisp.pl?gene=MK280073-118 |
| MK280073-119 | 3.370393753 | https://www.genecards.org/cgi-bin/carddisp.pl?gene=MK280073-119 |
| MK280073-122 | 3.370393753 | https://www.genecards.org/cgi-bin/carddisp.pl?gene=MK280073-122 |
| MK280073-124 | 3.370393753 | https://www.genecards.org/cgi-bin/carddisp.pl?gene=MK280073-124 |
| MK280073-125 | 3.370393753 | https://www.genecards.org/cgi-bin/carddisp.pl?gene=MK280073-125 |
| MK280073-126 | 3.370393753 | https://www.genecards.org/cgi-bin/carddisp.pl?gene=MK280073-126 |
| MK280073-127 | 3.370393753 | https://www.genecards.org/cgi-bin/carddisp.pl?gene=MK280073-127 |
| MK280073-128 | 3.370393753 | https://www.genecards.org/cgi-bin/carddisp.pl?gene=MK280073-128 |
| MK280073-131 | 3.370393753 | https://www.genecards.org/cgi-bin/carddisp.pl?gene=MK280073-131 |
| MK280073-132 | 3.370393753 | https://www.genecards.org/cgi-bin/carddisp.pl?gene=MK280073-132 |
| MK280073-133 | 3.370393753 | https://www.genecards.org/cgi-bin/carddisp.pl?gene=MK280073-133 |
| MK280073-134 | 3.370393753 | https://www.genecards.org/cgi-bin/carddisp.pl?gene=MK280073-134 |
| MK280073-135 | 3.370393753 | https://www.genecards.org/cgi-bin/carddisp.pl?gene=MK280073-135 |
| MK280073-136 | 3.370393753 | https://www.genecards.org/cgi-bin/carddisp.pl?gene=MK280073-136 |
| MK280073-137 | 3.370393753 | https://www.genecards.org/cgi-bin/carddisp.pl?gene=MK280073-137 |
| MK280073-138 | 3.370393753 | https://www.genecards.org/cgi-bin/carddisp.pl?gene=MK280073-138 |
| MK280073-139 | 3.370393753 | https://www.genecards.org/cgi-bin/carddisp.pl?gene=MK280073-139 |
| MK280073-141 | 3.370393753 | https://www.genecards.org/cgi-bin/carddisp.pl?gene=MK280073-141 |
| MK280073-142 | 3.370393753 | https://www.genecards.org/cgi-bin/carddisp.pl?gene=MK280073-142 |
| MK280073-143 | 3.370393753 | https://www.genecards.org/cgi-bin/carddisp.pl?gene=MK280073-143 |
| MK280073-144 | 3.370393753 | https://www.genecards.org/cgi-bin/carddisp.pl?gene=MK280073-144 |
| MK280073-145 | 3.370393753 | https://www.genecards.org/cgi-bin/carddisp.pl?gene=MK280073-145 |
| MK280073-146 | 3.370393753 | https://www.genecards.org/cgi-bin/carddisp.pl?gene=MK280073-146 |
| MK280073-147 | 3.370393753 | https://www.genecards.org/cgi-bin/carddisp.pl?gene=MK280073-147 |
| MK280073-148 | 3.370393753 | https://www.genecards.org/cgi-bin/carddisp.pl?gene=MK280073-148 |
| MK280073-151 | 3.370393753 | https://www.genecards.org/cgi-bin/carddisp.pl?gene=MK280073-151 |
| MK280073-152 | 3.370393753 | https://www.genecards.org/cgi-bin/carddisp.pl?gene=MK280073-152 |
| MK280073-154 | 3.370393753 | https://www.genecards.org/cgi-bin/carddisp.pl?gene=MK280073-154 |
| MK280073-155 | 3.370393753 | https://www.genecards.org/cgi-bin/carddisp.pl?gene=MK280073-155 |
| MK280073-156 | 3.370393753 | https://www.genecards.org/cgi-bin/carddisp.pl?gene=MK280073-156 |
| MK280073-157 | 3.370393753 | https://www.genecards.org/cgi-bin/carddisp.pl?gene=MK280073-157 |
| MK280073-158 | 3.370393753 | https://www.genecards.org/cgi-bin/carddisp.pl?gene=MK280073-158 |
| MK280073-159 | 3.370393753 | https://www.genecards.org/cgi-bin/carddisp.pl?gene=MK280073-159 |
| MK280073-160 | 3.370393753 | https://www.genecards.org/cgi-bin/carddisp.pl?gene=MK280073-160 |
| MK280073-161 | 3.370393753 | https://www.genecards.org/cgi-bin/carddisp.pl?gene=MK280073-161 |
| MK280073-162 | 3.370393753 | https://www.genecards.org/cgi-bin/carddisp.pl?gene=MK280073-162 |
| MK280073-163 | 3.370393753 | https://www.genecards.org/cgi-bin/carddisp.pl?gene=MK280073-163 |
| MK280073-165 | 3.370393753 | https://www.genecards.org/cgi-bin/carddisp.pl?gene=MK280073-165 |
| MK280073-168 | 3.370393753 | https://www.genecards.org/cgi-bin/carddisp.pl?gene=MK280073-168 |
| MK280073-169 | 3.370393753 | https://www.genecards.org/cgi-bin/carddisp.pl?gene=MK280073-169 |
| MK280073-170 | 3.370393753 | https://www.genecards.org/cgi-bin/carddisp.pl?gene=MK280073-170 |
| MK280073-171 | 3.370393753 | https://www.genecards.org/cgi-bin/carddisp.pl?gene=MK280073-171 |
| MK280073-172 | 3.370393753 | https://www.genecards.org/cgi-bin/carddisp.pl?gene=MK280073-172 |
| MK280073-173 | 3.370393753 | https://www.genecards.org/cgi-bin/carddisp.pl?gene=MK280073-173 |
| MK280073-174 | 3.370393753 | https://www.genecards.org/cgi-bin/carddisp.pl?gene=MK280073-174 |
| MK280073-177 | 3.370393753 | https://www.genecards.org/cgi-bin/carddisp.pl?gene=MK280073-177 |
| MK280073-178 | 3.370393753 | https://www.genecards.org/cgi-bin/carddisp.pl?gene=MK280073-178 |
| MK280073-179 | 3.370393753 | https://www.genecards.org/cgi-bin/carddisp.pl?gene=MK280073-179 |
| MK280073-181 | 3.370393753 | https://www.genecards.org/cgi-bin/carddisp.pl?gene=MK280073-181 |
| MK280073-183 | 3.370393753 | https://www.genecards.org/cgi-bin/carddisp.pl?gene=MK280073-183 |
| MK280073-184 | 3.370393753 | https://www.genecards.org/cgi-bin/carddisp.pl?gene=MK280073-184 |
| MK280073-185 | 3.370393753 | https://www.genecards.org/cgi-bin/carddisp.pl?gene=MK280073-185 |
| MK280073-186 | 3.370393753 | https://www.genecards.org/cgi-bin/carddisp.pl?gene=MK280073-186 |
| MK280073-187 | 3.370393753 | https://www.genecards.org/cgi-bin/carddisp.pl?gene=MK280073-187 |
| MK280073-188 | 3.370393753 | https://www.genecards.org/cgi-bin/carddisp.pl?gene=MK280073-188 |
| MK280073-189 | 3.370393753 | https://www.genecards.org/cgi-bin/carddisp.pl?gene=MK280073-189 |
| MK280073-190 | 3.370393753 | https://www.genecards.org/cgi-bin/carddisp.pl?gene=MK280073-190 |
| MK280073-191 | 3.370393753 | https://www.genecards.org/cgi-bin/carddisp.pl?gene=MK280073-191 |
| MK280073-192 | 3.370393753 | https://www.genecards.org/cgi-bin/carddisp.pl?gene=MK280073-192 |
| MK280073-193 | 3.370393753 | https://www.genecards.org/cgi-bin/carddisp.pl?gene=MK280073-193 |
| MK280073-194 | 3.370393753 | https://www.genecards.org/cgi-bin/carddisp.pl?gene=MK280073-194 |
| MK280073-195 | 3.370393753 | https://www.genecards.org/cgi-bin/carddisp.pl?gene=MK280073-195 |
| MK280073-196 | 3.370393753 | https://www.genecards.org/cgi-bin/carddisp.pl?gene=MK280073-196 |
| MK280073-198 | 3.370393753 | https://www.genecards.org/cgi-bin/carddisp.pl?gene=MK280073-198 |
| MK280073-200 | 3.370393753 | https://www.genecards.org/cgi-bin/carddisp.pl?gene=MK280073-200 |
| MK280073-201 | 3.370393753 | https://www.genecards.org/cgi-bin/carddisp.pl?gene=MK280073-201 |
| MK280073-204 | 3.370393753 | https://www.genecards.org/cgi-bin/carddisp.pl?gene=MK280073-204 |
| MK280073-208 | 3.370393753 | https://www.genecards.org/cgi-bin/carddisp.pl?gene=MK280073-208 |
| MK280073-209 | 3.370393753 | https://www.genecards.org/cgi-bin/carddisp.pl?gene=MK280073-209 |
| MK280073-210 | 3.370393753 | https://www.genecards.org/cgi-bin/carddisp.pl?gene=MK280073-210 |
| MK280073-211 | 3.370393753 | https://www.genecards.org/cgi-bin/carddisp.pl?gene=MK280073-211 |
| MK280073-212 | 3.370393753 | https://www.genecards.org/cgi-bin/carddisp.pl?gene=MK280073-212 |
| MK280073-213 | 3.370393753 | https://www.genecards.org/cgi-bin/carddisp.pl?gene=MK280073-213 |
| MK280073-214 | 3.370393753 | https://www.genecards.org/cgi-bin/carddisp.pl?gene=MK280073-214 |
| MK280073-215 | 3.370393753 | https://www.genecards.org/cgi-bin/carddisp.pl?gene=MK280073-215 |
| MK280073-217 | 3.370393753 | https://www.genecards.org/cgi-bin/carddisp.pl?gene=MK280073-217 |
| MK280073-218 | 3.370393753 | https://www.genecards.org/cgi-bin/carddisp.pl?gene=MK280073-218 |
| MK280073-219 | 3.370393753 | https://www.genecards.org/cgi-bin/carddisp.pl?gene=MK280073-219 |
| MK280073-220 | 3.370393753 | https://www.genecards.org/cgi-bin/carddisp.pl?gene=MK280073-220 |
| MK280073-221 | 3.370393753 | https://www.genecards.org/cgi-bin/carddisp.pl?gene=MK280073-221 |
| MK280073-222 | 3.370393753 | https://www.genecards.org/cgi-bin/carddisp.pl?gene=MK280073-222 |
| MK280073-224 | 3.370393753 | https://www.genecards.org/cgi-bin/carddisp.pl?gene=MK280073-224 |
| MK280073-225 | 3.370393753 | https://www.genecards.org/cgi-bin/carddisp.pl?gene=MK280073-225 |
| MK280073-226 | 3.370393753 | https://www.genecards.org/cgi-bin/carddisp.pl?gene=MK280073-226 |
| MK280073-227 | 3.370393753 | https://www.genecards.org/cgi-bin/carddisp.pl?gene=MK280073-227 |
| MK280073-228 | 3.370393753 | https://www.genecards.org/cgi-bin/carddisp.pl?gene=MK280073-228 |
| MK280073-229 | 3.370393753 | https://www.genecards.org/cgi-bin/carddisp.pl?gene=MK280073-229 |
| MK280073-230 | 3.370393753 | https://www.genecards.org/cgi-bin/carddisp.pl?gene=MK280073-230 |
| MK280073-231 | 3.370393753 | https://www.genecards.org/cgi-bin/carddisp.pl?gene=MK280073-231 |
| MK280073-232 | 3.370393753 | https://www.genecards.org/cgi-bin/carddisp.pl?gene=MK280073-232 |
| MK280073-233 | 3.370393753 | https://www.genecards.org/cgi-bin/carddisp.pl?gene=MK280073-233 |
| MK280073-234 | 3.370393753 | https://www.genecards.org/cgi-bin/carddisp.pl?gene=MK280073-234 |
| MK280073-235 | 3.370393753 | https://www.genecards.org/cgi-bin/carddisp.pl?gene=MK280073-235 |
| MK280073-236 | 3.370393753 | https://www.genecards.org/cgi-bin/carddisp.pl?gene=MK280073-236 |
| MK280073-237 | 3.370393753 | https://www.genecards.org/cgi-bin/carddisp.pl?gene=MK280073-237 |
| MK280073-238 | 3.370393753 | https://www.genecards.org/cgi-bin/carddisp.pl?gene=MK280073-238 |
| MK280073-239 | 3.370393753 | https://www.genecards.org/cgi-bin/carddisp.pl?gene=MK280073-239 |
| MK280073-240 | 3.370393753 | https://www.genecards.org/cgi-bin/carddisp.pl?gene=MK280073-240 |
| MK280073-241 | 3.370393753 | https://www.genecards.org/cgi-bin/carddisp.pl?gene=MK280073-241 |
| MK280073-242 | 3.370393753 | https://www.genecards.org/cgi-bin/carddisp.pl?gene=MK280073-242 |
| MK280073-244 | 3.370393753 | https://www.genecards.org/cgi-bin/carddisp.pl?gene=MK280073-244 |
| MK280073-245 | 3.370393753 | https://www.genecards.org/cgi-bin/carddisp.pl?gene=MK280073-245 |
| MK280073-246 | 3.370393753 | https://www.genecards.org/cgi-bin/carddisp.pl?gene=MK280073-246 |
| MK280073-247 | 3.370393753 | https://www.genecards.org/cgi-bin/carddisp.pl?gene=MK280073-247 |
| MK280073-248 | 3.370393753 | https://www.genecards.org/cgi-bin/carddisp.pl?gene=MK280073-248 |
| MK280073-249 | 3.370393753 | https://www.genecards.org/cgi-bin/carddisp.pl?gene=MK280073-249 |
| MK280073-250 | 3.370393753 | https://www.genecards.org/cgi-bin/carddisp.pl?gene=MK280073-250 |
| MK280073-251 | 3.370393753 | https://www.genecards.org/cgi-bin/carddisp.pl?gene=MK280073-251 |
| MK280073-252 | 3.370393753 | https://www.genecards.org/cgi-bin/carddisp.pl?gene=MK280073-252 |
| MK280073-253 | 3.370393753 | https://www.genecards.org/cgi-bin/carddisp.pl?gene=MK280073-253 |
| MK280073-255 | 3.370393753 | https://www.genecards.org/cgi-bin/carddisp.pl?gene=MK280073-255 |
| MK280073-257 | 3.370393753 | https://www.genecards.org/cgi-bin/carddisp.pl?gene=MK280073-257 |
| MK280073-258 | 3.370393753 | https://www.genecards.org/cgi-bin/carddisp.pl?gene=MK280073-258 |
| MK280073-259 | 3.370393753 | https://www.genecards.org/cgi-bin/carddisp.pl?gene=MK280073-259 |
| MK280073-260 | 3.370393753 | https://www.genecards.org/cgi-bin/carddisp.pl?gene=MK280073-260 |
| MK280073-261 | 3.370393753 | https://www.genecards.org/cgi-bin/carddisp.pl?gene=MK280073-261 |
| MK280073-262 | 3.370393753 | https://www.genecards.org/cgi-bin/carddisp.pl?gene=MK280073-262 |
| MK280073-264 | 3.370393753 | https://www.genecards.org/cgi-bin/carddisp.pl?gene=MK280073-264 |
| MK280073-265 | 3.370393753 | https://www.genecards.org/cgi-bin/carddisp.pl?gene=MK280073-265 |
| MK280073-266 | 3.370393753 | https://www.genecards.org/cgi-bin/carddisp.pl?gene=MK280073-266 |
| MK280073-267 | 3.370393753 | https://www.genecards.org/cgi-bin/carddisp.pl?gene=MK280073-267 |
| MK280073-268 | 3.370393753 | https://www.genecards.org/cgi-bin/carddisp.pl?gene=MK280073-268 |
| MK280073-269 | 3.370393753 | https://www.genecards.org/cgi-bin/carddisp.pl?gene=MK280073-269 |
| MK280073-270 | 3.370393753 | https://www.genecards.org/cgi-bin/carddisp.pl?gene=MK280073-270 |
| MK280073-271 | 3.370393753 | https://www.genecards.org/cgi-bin/carddisp.pl?gene=MK280073-271 |
| MK280073-272 | 3.370393753 | https://www.genecards.org/cgi-bin/carddisp.pl?gene=MK280073-272 |
| MK280073-274 | 3.370393753 | https://www.genecards.org/cgi-bin/carddisp.pl?gene=MK280073-274 |
| MK280073-275 | 3.370393753 | https://www.genecards.org/cgi-bin/carddisp.pl?gene=MK280073-275 |
| MK280073-276 | 3.370393753 | https://www.genecards.org/cgi-bin/carddisp.pl?gene=MK280073-276 |
| MK280073-277 | 3.370393753 | https://www.genecards.org/cgi-bin/carddisp.pl?gene=MK280073-277 |
| MK280073-278 | 3.370393753 | https://www.genecards.org/cgi-bin/carddisp.pl?gene=MK280073-278 |
| MK280073-279 | 3.370393753 | https://www.genecards.org/cgi-bin/carddisp.pl?gene=MK280073-279 |
| MK280073-280 | 3.370393753 | https://www.genecards.org/cgi-bin/carddisp.pl?gene=MK280073-280 |
| MK280073-281 | 3.370393753 | https://www.genecards.org/cgi-bin/carddisp.pl?gene=MK280073-281 |
| MK280073-282 | 3.370393753 | https://www.genecards.org/cgi-bin/carddisp.pl?gene=MK280073-282 |
| MK280073-284 | 3.370393753 | https://www.genecards.org/cgi-bin/carddisp.pl?gene=MK280073-284 |
| MK280073-285 | 3.370393753 | https://www.genecards.org/cgi-bin/carddisp.pl?gene=MK280073-285 |
| MK280073-286 | 3.370393753 | https://www.genecards.org/cgi-bin/carddisp.pl?gene=MK280073-286 |
| MK280073-287 | 3.370393753 | https://www.genecards.org/cgi-bin/carddisp.pl?gene=MK280073-287 |
| MK280073-288 | 3.370393753 | https://www.genecards.org/cgi-bin/carddisp.pl?gene=MK280073-288 |
| MK280073-289 | 3.370393753 | https://www.genecards.org/cgi-bin/carddisp.pl?gene=MK280073-289 |
| MK280073-290 | 3.370393753 | https://www.genecards.org/cgi-bin/carddisp.pl?gene=MK280073-290 |
| MK280073-291 | 3.370393753 | https://www.genecards.org/cgi-bin/carddisp.pl?gene=MK280073-291 |
| MK280073-292 | 3.370393753 | https://www.genecards.org/cgi-bin/carddisp.pl?gene=MK280073-292 |
| MK280073-293 | 3.370393753 | https://www.genecards.org/cgi-bin/carddisp.pl?gene=MK280073-293 |
| MK280073-294 | 3.370393753 | https://www.genecards.org/cgi-bin/carddisp.pl?gene=MK280073-294 |
| MK280073-297 | 3.370393753 | https://www.genecards.org/cgi-bin/carddisp.pl?gene=MK280073-297 |
| MK280073-298 | 3.370393753 | https://www.genecards.org/cgi-bin/carddisp.pl?gene=MK280073-298 |
| MK280073-299 | 3.370393753 | https://www.genecards.org/cgi-bin/carddisp.pl?gene=MK280073-299 |
| MK280073-301 | 3.370393753 | https://www.genecards.org/cgi-bin/carddisp.pl?gene=MK280073-301 |
| MK280073-302 | 3.370393753 | https://www.genecards.org/cgi-bin/carddisp.pl?gene=MK280073-302 |
| MK280073-303 | 3.370393753 | https://www.genecards.org/cgi-bin/carddisp.pl?gene=MK280073-303 |
| MK280073-304 | 3.370393753 | https://www.genecards.org/cgi-bin/carddisp.pl?gene=MK280073-304 |
| MK280073-305 | 3.370393753 | https://www.genecards.org/cgi-bin/carddisp.pl?gene=MK280073-305 |
| MK280073-306 | 3.370393753 | https://www.genecards.org/cgi-bin/carddisp.pl?gene=MK280073-306 |
| MK280073-307 | 3.370393753 | https://www.genecards.org/cgi-bin/carddisp.pl?gene=MK280073-307 |
| MK280073-308 | 3.370393753 | https://www.genecards.org/cgi-bin/carddisp.pl?gene=MK280073-308 |
| MK280073-309 | 3.370393753 | https://www.genecards.org/cgi-bin/carddisp.pl?gene=MK280073-309 |
| MK280073-310 | 3.370393753 | https://www.genecards.org/cgi-bin/carddisp.pl?gene=MK280073-310 |
| MK280073-311 | 3.370393753 | https://www.genecards.org/cgi-bin/carddisp.pl?gene=MK280073-311 |
| MK280073-312 | 3.370393753 | https://www.genecards.org/cgi-bin/carddisp.pl?gene=MK280073-312 |
| MK280073-313 | 3.370393753 | https://www.genecards.org/cgi-bin/carddisp.pl?gene=MK280073-313 |
| MK280073-314 | 3.370393753 | https://www.genecards.org/cgi-bin/carddisp.pl?gene=MK280073-314 |
| MK280073-315 | 3.370393753 | https://www.genecards.org/cgi-bin/carddisp.pl?gene=MK280073-315 |
| MK280073-316 | 3.370393753 | https://www.genecards.org/cgi-bin/carddisp.pl?gene=MK280073-316 |
| MK280073-317 | 3.370393753 | https://www.genecards.org/cgi-bin/carddisp.pl?gene=MK280073-317 |
| MK280073-318 | 3.370393753 | https://www.genecards.org/cgi-bin/carddisp.pl?gene=MK280073-318 |
| MK280073-319 | 3.370393753 | https://www.genecards.org/cgi-bin/carddisp.pl?gene=MK280073-319 |
| MK280073-320 | 3.370393753 | https://www.genecards.org/cgi-bin/carddisp.pl?gene=MK280073-320 |
| MK280073-321 | 3.370393753 | https://www.genecards.org/cgi-bin/carddisp.pl?gene=MK280073-321 |
| MK280073-322 | 3.370393753 | https://www.genecards.org/cgi-bin/carddisp.pl?gene=MK280073-322 |
| MK280073-323 | 3.370393753 | https://www.genecards.org/cgi-bin/carddisp.pl?gene=MK280073-323 |
| MK280073-324 | 3.370393753 | https://www.genecards.org/cgi-bin/carddisp.pl?gene=MK280073-324 |
| MK280073-325 | 3.370393753 | https://www.genecards.org/cgi-bin/carddisp.pl?gene=MK280073-325 |
| MK280073-326 | 3.370393753 | https://www.genecards.org/cgi-bin/carddisp.pl?gene=MK280073-326 |
| MK280073-328 | 3.370393753 | https://www.genecards.org/cgi-bin/carddisp.pl?gene=MK280073-328 |
| MK280073-329 | 3.370393753 | https://www.genecards.org/cgi-bin/carddisp.pl?gene=MK280073-329 |
| MK280073-330 | 3.370393753 | https://www.genecards.org/cgi-bin/carddisp.pl?gene=MK280073-330 |
| MK280073-331 | 3.370393753 | https://www.genecards.org/cgi-bin/carddisp.pl?gene=MK280073-331 |
| MK280073-332 | 3.370393753 | https://www.genecards.org/cgi-bin/carddisp.pl?gene=MK280073-332 |
| MK280073-333 | 3.370393753 | https://www.genecards.org/cgi-bin/carddisp.pl?gene=MK280073-333 |
| MK280073-335 | 3.370393753 | https://www.genecards.org/cgi-bin/carddisp.pl?gene=MK280073-335 |
| MK280073-336 | 3.370393753 | https://www.genecards.org/cgi-bin/carddisp.pl?gene=MK280073-336 |
| MK280073-337 | 3.370393753 | https://www.genecards.org/cgi-bin/carddisp.pl?gene=MK280073-337 |
| MK280073-338 | 3.370393753 | https://www.genecards.org/cgi-bin/carddisp.pl?gene=MK280073-338 |
| MK280073-339 | 3.370393753 | https://www.genecards.org/cgi-bin/carddisp.pl?gene=MK280073-339 |
| MK280073-340 | 3.370393753 | https://www.genecards.org/cgi-bin/carddisp.pl?gene=MK280073-340 |
| MK280073-341 | 3.370393753 | https://www.genecards.org/cgi-bin/carddisp.pl?gene=MK280073-341 |
| MK280073-342 | 3.370393753 | https://www.genecards.org/cgi-bin/carddisp.pl?gene=MK280073-342 |
| MK280073-343 | 3.370393753 | https://www.genecards.org/cgi-bin/carddisp.pl?gene=MK280073-343 |
| MK280073-344 | 3.370393753 | https://www.genecards.org/cgi-bin/carddisp.pl?gene=MK280073-344 |
| MK280073-345 | 3.370393753 | https://www.genecards.org/cgi-bin/carddisp.pl?gene=MK280073-345 |
| MK280073-347 | 3.370393753 | https://www.genecards.org/cgi-bin/carddisp.pl?gene=MK280073-347 |
| MK280073-348 | 3.370393753 | https://www.genecards.org/cgi-bin/carddisp.pl?gene=MK280073-348 |
| MK280073-349 | 3.370393753 | https://www.genecards.org/cgi-bin/carddisp.pl?gene=MK280073-349 |
| MK280073-350 | 3.370393753 | https://www.genecards.org/cgi-bin/carddisp.pl?gene=MK280073-350 |
| MK280073-352 | 3.370393753 | https://www.genecards.org/cgi-bin/carddisp.pl?gene=MK280073-352 |
| MK280073-355 | 3.370393753 | https://www.genecards.org/cgi-bin/carddisp.pl?gene=MK280073-355 |
| MK280073-356 | 3.370393753 | https://www.genecards.org/cgi-bin/carddisp.pl?gene=MK280073-356 |
| MK280073-357 | 3.370393753 | https://www.genecards.org/cgi-bin/carddisp.pl?gene=MK280073-357 |
| MK280073-358 | 3.370393753 | https://www.genecards.org/cgi-bin/carddisp.pl?gene=MK280073-358 |
| MK280073-360 | 3.370393753 | https://www.genecards.org/cgi-bin/carddisp.pl?gene=MK280073-360 |
| MK280073-361 | 3.370393753 | https://www.genecards.org/cgi-bin/carddisp.pl?gene=MK280073-361 |
| MK280073-362 | 3.370393753 | https://www.genecards.org/cgi-bin/carddisp.pl?gene=MK280073-362 |
| MK280073-363 | 3.370393753 | https://www.genecards.org/cgi-bin/carddisp.pl?gene=MK280073-363 |
| MK280073-364 | 3.370393753 | https://www.genecards.org/cgi-bin/carddisp.pl?gene=MK280073-364 |
| MK280073-365 | 3.370393753 | https://www.genecards.org/cgi-bin/carddisp.pl?gene=MK280073-365 |
| MK280073-366 | 3.370393753 | https://www.genecards.org/cgi-bin/carddisp.pl?gene=MK280073-366 |
| MK280073-367 | 3.370393753 | https://www.genecards.org/cgi-bin/carddisp.pl?gene=MK280073-367 |
| MK280073-368 | 3.370393753 | https://www.genecards.org/cgi-bin/carddisp.pl?gene=MK280073-368 |
| MK280073-369 | 3.370393753 | https://www.genecards.org/cgi-bin/carddisp.pl?gene=MK280073-369 |
| MK280073-370 | 3.370393753 | https://www.genecards.org/cgi-bin/carddisp.pl?gene=MK280073-370 |
| MK280073-371 | 3.370393753 | https://www.genecards.org/cgi-bin/carddisp.pl?gene=MK280073-371 |
| MK280073-372 | 3.370393753 | https://www.genecards.org/cgi-bin/carddisp.pl?gene=MK280073-372 |
| MK280073-373 | 3.370393753 | https://www.genecards.org/cgi-bin/carddisp.pl?gene=MK280073-373 |
| MK280073-374 | 3.370393753 | https://www.genecards.org/cgi-bin/carddisp.pl?gene=MK280073-374 |
| MK280073-375 | 3.370393753 | https://www.genecards.org/cgi-bin/carddisp.pl?gene=MK280073-375 |
| MK280073-376 | 3.370393753 | https://www.genecards.org/cgi-bin/carddisp.pl?gene=MK280073-376 |
| MK280073-377 | 3.370393753 | https://www.genecards.org/cgi-bin/carddisp.pl?gene=MK280073-377 |
| MK280073-378 | 3.370393753 | https://www.genecards.org/cgi-bin/carddisp.pl?gene=MK280073-378 |
| MK280073-379 | 3.370393753 | https://www.genecards.org/cgi-bin/carddisp.pl?gene=MK280073-379 |
| MK280073-380 | 3.370393753 | https://www.genecards.org/cgi-bin/carddisp.pl?gene=MK280073-380 |
| MK280073-381 | 3.370393753 | https://www.genecards.org/cgi-bin/carddisp.pl?gene=MK280073-381 |
| MK280073-382 | 3.370393753 | https://www.genecards.org/cgi-bin/carddisp.pl?gene=MK280073-382 |
| MK280073-383 | 3.370393753 | https://www.genecards.org/cgi-bin/carddisp.pl?gene=MK280073-383 |
| MK280073-384 | 3.370393753 | https://www.genecards.org/cgi-bin/carddisp.pl?gene=MK280073-384 |
| MK280073-385 | 3.370393753 | https://www.genecards.org/cgi-bin/carddisp.pl?gene=MK280073-385 |
| MK280073-386 | 3.370393753 | https://www.genecards.org/cgi-bin/carddisp.pl?gene=MK280073-386 |
| MK280073-387 | 3.370393753 | https://www.genecards.org/cgi-bin/carddisp.pl?gene=MK280073-387 |
| MK280073-388 | 3.370393753 | https://www.genecards.org/cgi-bin/carddisp.pl?gene=MK280073-388 |
| MK280073-390 | 3.370393753 | https://www.genecards.org/cgi-bin/carddisp.pl?gene=MK280073-390 |
| MK280073-391 | 3.370393753 | https://www.genecards.org/cgi-bin/carddisp.pl?gene=MK280073-391 |
| MK280073-395 | 3.370393753 | https://www.genecards.org/cgi-bin/carddisp.pl?gene=MK280073-395 |
| MK280073-396 | 3.370393753 | https://www.genecards.org/cgi-bin/carddisp.pl?gene=MK280073-396 |
| MK280073-397 | 3.370393753 | https://www.genecards.org/cgi-bin/carddisp.pl?gene=MK280073-397 |
| MK280073-398 | 3.370393753 | https://www.genecards.org/cgi-bin/carddisp.pl?gene=MK280073-398 |
| MK280073-399 | 3.370393753 | https://www.genecards.org/cgi-bin/carddisp.pl?gene=MK280073-399 |
| MK280073-402 | 3.370393753 | https://www.genecards.org/cgi-bin/carddisp.pl?gene=MK280073-402 |
| MK280073-403 | 3.370393753 | https://www.genecards.org/cgi-bin/carddisp.pl?gene=MK280073-403 |
| MK280073-404 | 3.370393753 | https://www.genecards.org/cgi-bin/carddisp.pl?gene=MK280073-404 |
| MK280073-405 | 3.370393753 | https://www.genecards.org/cgi-bin/carddisp.pl?gene=MK280073-405 |
| MK280073-406 | 3.370393753 | https://www.genecards.org/cgi-bin/carddisp.pl?gene=MK280073-406 |
| MK280073-407 | 3.370393753 | https://www.genecards.org/cgi-bin/carddisp.pl?gene=MK280073-407 |
| MK280073-409 | 3.370393753 | https://www.genecards.org/cgi-bin/carddisp.pl?gene=MK280073-409 |
| MK280073-410 | 3.370393753 | https://www.genecards.org/cgi-bin/carddisp.pl?gene=MK280073-410 |
| MK280073-411 | 3.370393753 | https://www.genecards.org/cgi-bin/carddisp.pl?gene=MK280073-411 |
| MK280073-412 | 3.370393753 | https://www.genecards.org/cgi-bin/carddisp.pl?gene=MK280073-412 |
| MK280073-413 | 3.370393753 | https://www.genecards.org/cgi-bin/carddisp.pl?gene=MK280073-413 |
| MK280073-414 | 3.370393753 | https://www.genecards.org/cgi-bin/carddisp.pl?gene=MK280073-414 |
| MK280073-415 | 3.370393753 | https://www.genecards.org/cgi-bin/carddisp.pl?gene=MK280073-415 |
| MK280073-416 | 3.370393753 | https://www.genecards.org/cgi-bin/carddisp.pl?gene=MK280073-416 |
| MK280073-417 | 3.370393753 | https://www.genecards.org/cgi-bin/carddisp.pl?gene=MK280073-417 |
| MK280073-418 | 3.370393753 | https://www.genecards.org/cgi-bin/carddisp.pl?gene=MK280073-418 |
| MK280073-419 | 3.370393753 | https://www.genecards.org/cgi-bin/carddisp.pl?gene=MK280073-419 |
| MK280073-420 | 3.370393753 | https://www.genecards.org/cgi-bin/carddisp.pl?gene=MK280073-420 |
| MK280073-427 | 3.370393753 | https://www.genecards.org/cgi-bin/carddisp.pl?gene=MK280073-427 |
| MK280073-428 | 3.370393753 | https://www.genecards.org/cgi-bin/carddisp.pl?gene=MK280073-428 |
| MK280073-430 | 3.370393753 | https://www.genecards.org/cgi-bin/carddisp.pl?gene=MK280073-430 |
| MK280073-431 | 3.370393753 | https://www.genecards.org/cgi-bin/carddisp.pl?gene=MK280073-431 |
| MK280073-432 | 3.370393753 | https://www.genecards.org/cgi-bin/carddisp.pl?gene=MK280073-432 |
| MK280073-433 | 3.370393753 | https://www.genecards.org/cgi-bin/carddisp.pl?gene=MK280073-433 |
| MK280073-434 | 3.370393753 | https://www.genecards.org/cgi-bin/carddisp.pl?gene=MK280073-434 |
| MK280073-435 | 3.370393753 | https://www.genecards.org/cgi-bin/carddisp.pl?gene=MK280073-435 |
| MK280073-436 | 3.370393753 | https://www.genecards.org/cgi-bin/carddisp.pl?gene=MK280073-436 |
| MK280073-437 | 3.370393753 | https://www.genecards.org/cgi-bin/carddisp.pl?gene=MK280073-437 |
| MK280073-438 | 3.370393753 | https://www.genecards.org/cgi-bin/carddisp.pl?gene=MK280073-438 |
| MK280073-439 | 3.370393753 | https://www.genecards.org/cgi-bin/carddisp.pl?gene=MK280073-439 |
| MK280073-440 | 3.370393753 | https://www.genecards.org/cgi-bin/carddisp.pl?gene=MK280073-440 |
| MK280073-441 | 3.370393753 | https://www.genecards.org/cgi-bin/carddisp.pl?gene=MK280073-441 |
| MK280073-442 | 3.370393753 | https://www.genecards.org/cgi-bin/carddisp.pl?gene=MK280073-442 |
| MK280073-443 | 3.370393753 | https://www.genecards.org/cgi-bin/carddisp.pl?gene=MK280073-443 |
| MK280073-444 | 3.370393753 | https://www.genecards.org/cgi-bin/carddisp.pl?gene=MK280073-444 |
| MK280073-445 | 3.370393753 | https://www.genecards.org/cgi-bin/carddisp.pl?gene=MK280073-445 |
| MK280073-446 | 3.370393753 | https://www.genecards.org/cgi-bin/carddisp.pl?gene=MK280073-446 |
| MK280073-447 | 3.370393753 | https://www.genecards.org/cgi-bin/carddisp.pl?gene=MK280073-447 |
| MK280073-448 | 3.370393753 | https://www.genecards.org/cgi-bin/carddisp.pl?gene=MK280073-448 |
| MK280073-449 | 3.370393753 | https://www.genecards.org/cgi-bin/carddisp.pl?gene=MK280073-449 |
| MK280073-450 | 3.370393753 | https://www.genecards.org/cgi-bin/carddisp.pl?gene=MK280073-450 |
| MK280073-451 | 3.370393753 | https://www.genecards.org/cgi-bin/carddisp.pl?gene=MK280073-451 |
| MK280073-452 | 3.370393753 | https://www.genecards.org/cgi-bin/carddisp.pl?gene=MK280073-452 |
| MK280073-454 | 3.370393753 | https://www.genecards.org/cgi-bin/carddisp.pl?gene=MK280073-454 |
| MK280073-455 | 3.370393753 | https://www.genecards.org/cgi-bin/carddisp.pl?gene=MK280073-455 |
| MK280073-457 | 3.370393753 | https://www.genecards.org/cgi-bin/carddisp.pl?gene=MK280073-457 |
| MK280073-458 | 3.370393753 | https://www.genecards.org/cgi-bin/carddisp.pl?gene=MK280073-458 |
| MK280073-459 | 3.370393753 | https://www.genecards.org/cgi-bin/carddisp.pl?gene=MK280073-459 |
| MK280073-462 | 3.370393753 | https://www.genecards.org/cgi-bin/carddisp.pl?gene=MK280073-462 |
| MK280073-463 | 3.370393753 | https://www.genecards.org/cgi-bin/carddisp.pl?gene=MK280073-463 |
| MK280073-465 | 3.370393753 | https://www.genecards.org/cgi-bin/carddisp.pl?gene=MK280073-465 |
| MK280073-466 | 3.370393753 | https://www.genecards.org/cgi-bin/carddisp.pl?gene=MK280073-466 |
| MK280073-467 | 3.370393753 | https://www.genecards.org/cgi-bin/carddisp.pl?gene=MK280073-467 |
| MK280073-469 | 3.370393753 | https://www.genecards.org/cgi-bin/carddisp.pl?gene=MK280073-469 |
| MK280073-471 | 3.370393753 | https://www.genecards.org/cgi-bin/carddisp.pl?gene=MK280073-471 |
| MK280073-472 | 3.370393753 | https://www.genecards.org/cgi-bin/carddisp.pl?gene=MK280073-472 |
| MK280073-474 | 3.370393753 | https://www.genecards.org/cgi-bin/carddisp.pl?gene=MK280073-474 |
| MK280073-476 | 3.370393753 | https://www.genecards.org/cgi-bin/carddisp.pl?gene=MK280073-476 |
| MK280073-477 | 3.370393753 | https://www.genecards.org/cgi-bin/carddisp.pl?gene=MK280073-477 |
| MK280073-478 | 3.370393753 | https://www.genecards.org/cgi-bin/carddisp.pl?gene=MK280073-478 |
| MK280073-479 | 3.370393753 | https://www.genecards.org/cgi-bin/carddisp.pl?gene=MK280073-479 |
| MK280073-480 | 3.370393753 | https://www.genecards.org/cgi-bin/carddisp.pl?gene=MK280073-480 |
| MK280073-481 | 3.370393753 | https://www.genecards.org/cgi-bin/carddisp.pl?gene=MK280073-481 |
| MK280073-482 | 3.370393753 | https://www.genecards.org/cgi-bin/carddisp.pl?gene=MK280073-482 |
| MK280073-483 | 3.370393753 | https://www.genecards.org/cgi-bin/carddisp.pl?gene=MK280073-483 |
| MK280073-484 | 3.370393753 | https://www.genecards.org/cgi-bin/carddisp.pl?gene=MK280073-484 |
| MK280073-485 | 3.370393753 | https://www.genecards.org/cgi-bin/carddisp.pl?gene=MK280073-485 |
| MK280073-486 | 3.370393753 | https://www.genecards.org/cgi-bin/carddisp.pl?gene=MK280073-486 |
| MK280073-487 | 3.370393753 | https://www.genecards.org/cgi-bin/carddisp.pl?gene=MK280073-487 |
| MK280073-488 | 3.370393753 | https://www.genecards.org/cgi-bin/carddisp.pl?gene=MK280073-488 |
| MK280073-491 | 3.370393753 | https://www.genecards.org/cgi-bin/carddisp.pl?gene=MK280073-491 |
| MK280073-492 | 3.370393753 | https://www.genecards.org/cgi-bin/carddisp.pl?gene=MK280073-492 |
| MK280073-494 | 3.370393753 | https://www.genecards.org/cgi-bin/carddisp.pl?gene=MK280073-494 |
| MK280073-495 | 3.370393753 | https://www.genecards.org/cgi-bin/carddisp.pl?gene=MK280073-495 |
| MK280073-496 | 3.370393753 | https://www.genecards.org/cgi-bin/carddisp.pl?gene=MK280073-496 |
| MK280073-497 | 3.370393753 | https://www.genecards.org/cgi-bin/carddisp.pl?gene=MK280073-497 |
| MK280073-498 | 3.370393753 | https://www.genecards.org/cgi-bin/carddisp.pl?gene=MK280073-498 |
| MK280073-499 | 3.370393753 | https://www.genecards.org/cgi-bin/carddisp.pl?gene=MK280073-499 |
| MK280073-500 | 3.370393753 | https://www.genecards.org/cgi-bin/carddisp.pl?gene=MK280073-500 |
| MK280073-502 | 3.370393753 | https://www.genecards.org/cgi-bin/carddisp.pl?gene=MK280073-502 |
| MK280073-503 | 3.370393753 | https://www.genecards.org/cgi-bin/carddisp.pl?gene=MK280073-503 |
| MK280073-504 | 3.370393753 | https://www.genecards.org/cgi-bin/carddisp.pl?gene=MK280073-504 |
| MK280073-505 | 3.370393753 | https://www.genecards.org/cgi-bin/carddisp.pl?gene=MK280073-505 |
| MK280073-506 | 3.370393753 | https://www.genecards.org/cgi-bin/carddisp.pl?gene=MK280073-506 |
| MK280073-507 | 3.370393753 | https://www.genecards.org/cgi-bin/carddisp.pl?gene=MK280073-507 |
| MK280073-508 | 3.370393753 | https://www.genecards.org/cgi-bin/carddisp.pl?gene=MK280073-508 |
| MK280073-509 | 3.370393753 | https://www.genecards.org/cgi-bin/carddisp.pl?gene=MK280073-509 |
| MK280073-511 | 3.370393753 | https://www.genecards.org/cgi-bin/carddisp.pl?gene=MK280073-511 |
| MK280073-512 | 3.370393753 | https://www.genecards.org/cgi-bin/carddisp.pl?gene=MK280073-512 |
| MK280073-514 | 3.370393753 | https://www.genecards.org/cgi-bin/carddisp.pl?gene=MK280073-514 |
| MK280073-515 | 3.370393753 | https://www.genecards.org/cgi-bin/carddisp.pl?gene=MK280073-515 |
| MK280073-516 | 3.370393753 | https://www.genecards.org/cgi-bin/carddisp.pl?gene=MK280073-516 |
| MK280073-517 | 3.370393753 | https://www.genecards.org/cgi-bin/carddisp.pl?gene=MK280073-517 |
| MK280073-518 | 3.370393753 | https://www.genecards.org/cgi-bin/carddisp.pl?gene=MK280073-518 |
| MK280073-526 | 3.370393753 | https://www.genecards.org/cgi-bin/carddisp.pl?gene=MK280073-526 |
| MK280073-528 | 3.370393753 | https://www.genecards.org/cgi-bin/carddisp.pl?gene=MK280073-528 |
| MK280073-529 | 3.370393753 | https://www.genecards.org/cgi-bin/carddisp.pl?gene=MK280073-529 |
| MK280073-530 | 3.370393753 | https://www.genecards.org/cgi-bin/carddisp.pl?gene=MK280073-530 |
| MK280073-531 | 3.370393753 | https://www.genecards.org/cgi-bin/carddisp.pl?gene=MK280073-531 |
| MK280073-532 | 3.370393753 | https://www.genecards.org/cgi-bin/carddisp.pl?gene=MK280073-532 |
| MK280073-534 | 3.370393753 | https://www.genecards.org/cgi-bin/carddisp.pl?gene=MK280073-534 |
| MK280073-535 | 3.370393753 | https://www.genecards.org/cgi-bin/carddisp.pl?gene=MK280073-535 |
| MK280073-536 | 3.370393753 | https://www.genecards.org/cgi-bin/carddisp.pl?gene=MK280073-536 |
| MK280073-537 | 3.370393753 | https://www.genecards.org/cgi-bin/carddisp.pl?gene=MK280073-537 |
| MK280073-538 | 3.370393753 | https://www.genecards.org/cgi-bin/carddisp.pl?gene=MK280073-538 |
| MK280073-540 | 3.370393753 | https://www.genecards.org/cgi-bin/carddisp.pl?gene=MK280073-540 |
| MK280073-541 | 3.370393753 | https://www.genecards.org/cgi-bin/carddisp.pl?gene=MK280073-541 |
| MK280073-542 | 3.370393753 | https://www.genecards.org/cgi-bin/carddisp.pl?gene=MK280073-542 |
| MK280073-543 | 3.370393753 | https://www.genecards.org/cgi-bin/carddisp.pl?gene=MK280073-543 |
| MK280073-545 | 3.370393753 | https://www.genecards.org/cgi-bin/carddisp.pl?gene=MK280073-545 |
| MK280073-546 | 3.370393753 | https://www.genecards.org/cgi-bin/carddisp.pl?gene=MK280073-546 |
| MK280073-547 | 3.370393753 | https://www.genecards.org/cgi-bin/carddisp.pl?gene=MK280073-547 |
| MK280073-548 | 3.370393753 | https://www.genecards.org/cgi-bin/carddisp.pl?gene=MK280073-548 |
| MK280073-549 | 3.370393753 | https://www.genecards.org/cgi-bin/carddisp.pl?gene=MK280073-549 |
| MK280073-550 | 3.370393753 | https://www.genecards.org/cgi-bin/carddisp.pl?gene=MK280073-550 |
| MK280073-551 | 3.370393753 | https://www.genecards.org/cgi-bin/carddisp.pl?gene=MK280073-551 |
| MK280073-552 | 3.370393753 | https://www.genecards.org/cgi-bin/carddisp.pl?gene=MK280073-552 |
| MK280073-553 | 3.370393753 | https://www.genecards.org/cgi-bin/carddisp.pl?gene=MK280073-553 |
| MK280073-554 | 3.370393753 | https://www.genecards.org/cgi-bin/carddisp.pl?gene=MK280073-554 |
| MK280073-555 | 3.370393753 | https://www.genecards.org/cgi-bin/carddisp.pl?gene=MK280073-555 |
| MK280073-556 | 3.370393753 | https://www.genecards.org/cgi-bin/carddisp.pl?gene=MK280073-556 |
| MK280073-558 | 3.370393753 | https://www.genecards.org/cgi-bin/carddisp.pl?gene=MK280073-558 |
| MK280073-559 | 3.370393753 | https://www.genecards.org/cgi-bin/carddisp.pl?gene=MK280073-559 |
| MK280073-560 | 3.370393753 | https://www.genecards.org/cgi-bin/carddisp.pl?gene=MK280073-560 |
| MK280073-562 | 3.370393753 | https://www.genecards.org/cgi-bin/carddisp.pl?gene=MK280073-562 |
| MK280073-563 | 3.370393753 | https://www.genecards.org/cgi-bin/carddisp.pl?gene=MK280073-563 |
| MK280073-564 | 3.370393753 | https://www.genecards.org/cgi-bin/carddisp.pl?gene=MK280073-564 |
| MK280073-565 | 3.370393753 | https://www.genecards.org/cgi-bin/carddisp.pl?gene=MK280073-565 |
| MK280073-566 | 3.370393753 | https://www.genecards.org/cgi-bin/carddisp.pl?gene=MK280073-566 |
| MK280073-567 | 3.370393753 | https://www.genecards.org/cgi-bin/carddisp.pl?gene=MK280073-567 |
| MK280073-568 | 3.370393753 | https://www.genecards.org/cgi-bin/carddisp.pl?gene=MK280073-568 |
| MK280073-569 | 3.370393753 | https://www.genecards.org/cgi-bin/carddisp.pl?gene=MK280073-569 |
| MK280073-570 | 3.370393753 | https://www.genecards.org/cgi-bin/carddisp.pl?gene=MK280073-570 |
| MK280073-572 | 3.370393753 | https://www.genecards.org/cgi-bin/carddisp.pl?gene=MK280073-572 |
| MK280073-573 | 3.370393753 | https://www.genecards.org/cgi-bin/carddisp.pl?gene=MK280073-573 |
| MK280073-574 | 3.370393753 | https://www.genecards.org/cgi-bin/carddisp.pl?gene=MK280073-574 |
| MK280073-575 | 3.370393753 | https://www.genecards.org/cgi-bin/carddisp.pl?gene=MK280073-575 |
| MK280073-577 | 3.370393753 | https://www.genecards.org/cgi-bin/carddisp.pl?gene=MK280073-577 |
| MK280073-578 | 3.370393753 | https://www.genecards.org/cgi-bin/carddisp.pl?gene=MK280073-578 |
| MK280073-579 | 3.370393753 | https://www.genecards.org/cgi-bin/carddisp.pl?gene=MK280073-579 |
| MK280073-580 | 3.370393753 | https://www.genecards.org/cgi-bin/carddisp.pl?gene=MK280073-580 |
| MK280073-581 | 3.370393753 | https://www.genecards.org/cgi-bin/carddisp.pl?gene=MK280073-581 |
| MK280073-582 | 3.370393753 | https://www.genecards.org/cgi-bin/carddisp.pl?gene=MK280073-582 |
| MK280073-583 | 3.370393753 | https://www.genecards.org/cgi-bin/carddisp.pl?gene=MK280073-583 |
| MK280073-584 | 3.370393753 | https://www.genecards.org/cgi-bin/carddisp.pl?gene=MK280073-584 |
| MK280073-585 | 3.370393753 | https://www.genecards.org/cgi-bin/carddisp.pl?gene=MK280073-585 |
| MK280073-588 | 3.370393753 | https://www.genecards.org/cgi-bin/carddisp.pl?gene=MK280073-588 |
| MK280073-589 | 3.370393753 | https://www.genecards.org/cgi-bin/carddisp.pl?gene=MK280073-589 |
| MK280073-590 | 3.370393753 | https://www.genecards.org/cgi-bin/carddisp.pl?gene=MK280073-590 |
| MK280073-591 | 3.370393753 | https://www.genecards.org/cgi-bin/carddisp.pl?gene=MK280073-591 |
| MK280073-592 | 3.370393753 | https://www.genecards.org/cgi-bin/carddisp.pl?gene=MK280073-592 |
| MK280073-593 | 3.370393753 | https://www.genecards.org/cgi-bin/carddisp.pl?gene=MK280073-593 |
| MK280073-594 | 3.370393753 | https://www.genecards.org/cgi-bin/carddisp.pl?gene=MK280073-594 |
| MK280073-595 | 3.370393753 | https://www.genecards.org/cgi-bin/carddisp.pl?gene=MK280073-595 |
| MK280073-596 | 3.370393753 | https://www.genecards.org/cgi-bin/carddisp.pl?gene=MK280073-596 |
| MK280073-597 | 3.370393753 | https://www.genecards.org/cgi-bin/carddisp.pl?gene=MK280073-597 |
| MK280073-598 | 3.370393753 | https://www.genecards.org/cgi-bin/carddisp.pl?gene=MK280073-598 |
| MK280073-599 | 3.370393753 | https://www.genecards.org/cgi-bin/carddisp.pl?gene=MK280073-599 |
| MK280073-600 | 3.370393753 | https://www.genecards.org/cgi-bin/carddisp.pl?gene=MK280073-600 |
| MK280073-606 | 3.370393753 | https://www.genecards.org/cgi-bin/carddisp.pl?gene=MK280073-606 |
| MK280073-615 | 3.370393753 | https://www.genecards.org/cgi-bin/carddisp.pl?gene=MK280073-615 |
| MK280073-618 | 3.370393753 | https://www.genecards.org/cgi-bin/carddisp.pl?gene=MK280073-618 |
| MK280073-624 | 3.370393753 | https://www.genecards.org/cgi-bin/carddisp.pl?gene=MK280073-624 |
| MK280073-625 | 3.370393753 | https://www.genecards.org/cgi-bin/carddisp.pl?gene=MK280073-625 |
| MK280073-629 | 3.370393753 | https://www.genecards.org/cgi-bin/carddisp.pl?gene=MK280073-629 |
| MK280073-654 | 3.370393753 | https://www.genecards.org/cgi-bin/carddisp.pl?gene=MK280073-654 |
| MK280073-655 | 3.370393753 | https://www.genecards.org/cgi-bin/carddisp.pl?gene=MK280073-655 |
| MK280073-656 | 3.370393753 | https://www.genecards.org/cgi-bin/carddisp.pl?gene=MK280073-656 |
| MK280073-659 | 3.370393753 | https://www.genecards.org/cgi-bin/carddisp.pl?gene=MK280073-659 |
| MK280073-670 | 3.370393753 | https://www.genecards.org/cgi-bin/carddisp.pl?gene=MK280073-670 |
| MK280073-674 | 3.370393753 | https://www.genecards.org/cgi-bin/carddisp.pl?gene=MK280073-674 |
| MK280073-676 | 3.370393753 | https://www.genecards.org/cgi-bin/carddisp.pl?gene=MK280073-676 |
| MK280073-678 | 3.370393753 | https://www.genecards.org/cgi-bin/carddisp.pl?gene=MK280073-678 |
| MK280073-683 | 3.370393753 | https://www.genecards.org/cgi-bin/carddisp.pl?gene=MK280073-683 |
| MK280073-686 | 3.370393753 | https://www.genecards.org/cgi-bin/carddisp.pl?gene=MK280073-686 |
| MK280073-688 | 3.370393753 | https://www.genecards.org/cgi-bin/carddisp.pl?gene=MK280073-688 |
| MK280073-689 | 3.370393753 | https://www.genecards.org/cgi-bin/carddisp.pl?gene=MK280073-689 |
| MK280073-691 | 3.370393753 | https://www.genecards.org/cgi-bin/carddisp.pl?gene=MK280073-691 |
| lnc-LRP5L-15 | 3.370393753 | https://www.genecards.org/cgi-bin/carddisp.pl?gene=lnc-LRP5L-15 |
| MK280073-067 | 3.370393753 | https://www.genecards.org/cgi-bin/carddisp.pl?gene=MK280073-067 |
| MK280073-099 | 3.370393753 | https://www.genecards.org/cgi-bin/carddisp.pl?gene=MK280073-099 |
| MK280073-100 | 3.370393753 | https://www.genecards.org/cgi-bin/carddisp.pl?gene=MK280073-100 |
| MK280073-129 | 3.370393753 | https://www.genecards.org/cgi-bin/carddisp.pl?gene=MK280073-129 |
| MK280073-130 | 3.370393753 | https://www.genecards.org/cgi-bin/carddisp.pl?gene=MK280073-130 |
| MK280073-166 | 3.370393753 | https://www.genecards.org/cgi-bin/carddisp.pl?gene=MK280073-166 |
| MK280073-167 | 3.370393753 | https://www.genecards.org/cgi-bin/carddisp.pl?gene=MK280073-167 |
| MK280073-223 | 3.370393753 | https://www.genecards.org/cgi-bin/carddisp.pl?gene=MK280073-223 |
| MK280073-254 | 3.370393753 | https://www.genecards.org/cgi-bin/carddisp.pl?gene=MK280073-254 |
| MK280073-256 | 3.370393753 | https://www.genecards.org/cgi-bin/carddisp.pl?gene=MK280073-256 |
| MK280073-263 | 3.370393753 | https://www.genecards.org/cgi-bin/carddisp.pl?gene=MK280073-263 |
| MK280073-327 | 3.370393753 | https://www.genecards.org/cgi-bin/carddisp.pl?gene=MK280073-327 |
| MK280073-392 | 3.370393753 | https://www.genecards.org/cgi-bin/carddisp.pl?gene=MK280073-392 |
| MK280073-393 | 3.370393753 | https://www.genecards.org/cgi-bin/carddisp.pl?gene=MK280073-393 |
| MK280073-394 | 3.370393753 | https://www.genecards.org/cgi-bin/carddisp.pl?gene=MK280073-394 |
| MK280073-400 | 3.370393753 | https://www.genecards.org/cgi-bin/carddisp.pl?gene=MK280073-400 |
| MK280073-401 | 3.370393753 | https://www.genecards.org/cgi-bin/carddisp.pl?gene=MK280073-401 |
| MK280073-408 | 3.370393753 | https://www.genecards.org/cgi-bin/carddisp.pl?gene=MK280073-408 |
| MK280073-421 | 3.370393753 | https://www.genecards.org/cgi-bin/carddisp.pl?gene=MK280073-421 |
| MK280073-422 | 3.370393753 | https://www.genecards.org/cgi-bin/carddisp.pl?gene=MK280073-422 |
| MK280073-423 | 3.370393753 | https://www.genecards.org/cgi-bin/carddisp.pl?gene=MK280073-423 |
| MK280073-424 | 3.370393753 | https://www.genecards.org/cgi-bin/carddisp.pl?gene=MK280073-424 |
| MK280073-425 | 3.370393753 | https://www.genecards.org/cgi-bin/carddisp.pl?gene=MK280073-425 |
| MK280073-426 | 3.370393753 | https://www.genecards.org/cgi-bin/carddisp.pl?gene=MK280073-426 |
| MK280073-429 | 3.370393753 | https://www.genecards.org/cgi-bin/carddisp.pl?gene=MK280073-429 |
| MK280073-489 | 3.370393753 | https://www.genecards.org/cgi-bin/carddisp.pl?gene=MK280073-489 |
| MK280073-520 | 3.370393753 | https://www.genecards.org/cgi-bin/carddisp.pl?gene=MK280073-520 |
| MK280073-539 | 3.370393753 | https://www.genecards.org/cgi-bin/carddisp.pl?gene=MK280073-539 |
| MK280073-576 | 3.370393753 | https://www.genecards.org/cgi-bin/carddisp.pl?gene=MK280073-576 |
| MK280073-587 | 3.370393753 | https://www.genecards.org/cgi-bin/carddisp.pl?gene=MK280073-587 |
| MK280073-601 | 3.370393753 | https://www.genecards.org/cgi-bin/carddisp.pl?gene=MK280073-601 |
| MK280073-602 | 3.370393753 | https://www.genecards.org/cgi-bin/carddisp.pl?gene=MK280073-602 |
| MK280073-603 | 3.370393753 | https://www.genecards.org/cgi-bin/carddisp.pl?gene=MK280073-603 |
| MK280073-604 | 3.370393753 | https://www.genecards.org/cgi-bin/carddisp.pl?gene=MK280073-604 |
| MK280073-605 | 3.370393753 | https://www.genecards.org/cgi-bin/carddisp.pl?gene=MK280073-605 |
| MK280073-607 | 3.370393753 | https://www.genecards.org/cgi-bin/carddisp.pl?gene=MK280073-607 |
| MK280073-608 | 3.370393753 | https://www.genecards.org/cgi-bin/carddisp.pl?gene=MK280073-608 |
| MK280073-610 | 3.370393753 | https://www.genecards.org/cgi-bin/carddisp.pl?gene=MK280073-610 |
| MK280073-611 | 3.370393753 | https://www.genecards.org/cgi-bin/carddisp.pl?gene=MK280073-611 |
| MK280073-612 | 3.370393753 | https://www.genecards.org/cgi-bin/carddisp.pl?gene=MK280073-612 |
| MK280073-613 | 3.370393753 | https://www.genecards.org/cgi-bin/carddisp.pl?gene=MK280073-613 |
| MK280073-614 | 3.370393753 | https://www.genecards.org/cgi-bin/carddisp.pl?gene=MK280073-614 |
| MK280073-616 | 3.370393753 | https://www.genecards.org/cgi-bin/carddisp.pl?gene=MK280073-616 |
| MK280073-617 | 3.370393753 | https://www.genecards.org/cgi-bin/carddisp.pl?gene=MK280073-617 |
| MK280073-619 | 3.370393753 | https://www.genecards.org/cgi-bin/carddisp.pl?gene=MK280073-619 |
| MK280073-620 | 3.370393753 | https://www.genecards.org/cgi-bin/carddisp.pl?gene=MK280073-620 |
| MK280073-622 | 3.370393753 | https://www.genecards.org/cgi-bin/carddisp.pl?gene=MK280073-622 |
| MK280073-626 | 3.370393753 | https://www.genecards.org/cgi-bin/carddisp.pl?gene=MK280073-626 |
| MK280073-627 | 3.370393753 | https://www.genecards.org/cgi-bin/carddisp.pl?gene=MK280073-627 |
| MK280073-628 | 3.370393753 | https://www.genecards.org/cgi-bin/carddisp.pl?gene=MK280073-628 |
| MK280073-630 | 3.370393753 | https://www.genecards.org/cgi-bin/carddisp.pl?gene=MK280073-630 |
| MK280073-631 | 3.370393753 | https://www.genecards.org/cgi-bin/carddisp.pl?gene=MK280073-631 |
| MK280073-632 | 3.370393753 | https://www.genecards.org/cgi-bin/carddisp.pl?gene=MK280073-632 |
| MK280073-633 | 3.370393753 | https://www.genecards.org/cgi-bin/carddisp.pl?gene=MK280073-633 |
| MK280073-634 | 3.370393753 | https://www.genecards.org/cgi-bin/carddisp.pl?gene=MK280073-634 |
| MK280073-635 | 3.370393753 | https://www.genecards.org/cgi-bin/carddisp.pl?gene=MK280073-635 |
| MK280073-636 | 3.370393753 | https://www.genecards.org/cgi-bin/carddisp.pl?gene=MK280073-636 |
| MK280073-637 | 3.370393753 | https://www.genecards.org/cgi-bin/carddisp.pl?gene=MK280073-637 |
| MK280073-638 | 3.370393753 | https://www.genecards.org/cgi-bin/carddisp.pl?gene=MK280073-638 |
| MK280073-639 | 3.370393753 | https://www.genecards.org/cgi-bin/carddisp.pl?gene=MK280073-639 |
| MK280073-640 | 3.370393753 | https://www.genecards.org/cgi-bin/carddisp.pl?gene=MK280073-640 |
| MK280073-641 | 3.370393753 | https://www.genecards.org/cgi-bin/carddisp.pl?gene=MK280073-641 |
| MK280073-642 | 3.370393753 | https://www.genecards.org/cgi-bin/carddisp.pl?gene=MK280073-642 |
| MK280073-643 | 3.370393753 | https://www.genecards.org/cgi-bin/carddisp.pl?gene=MK280073-643 |
| MK280073-644 | 3.370393753 | https://www.genecards.org/cgi-bin/carddisp.pl?gene=MK280073-644 |
| MK280073-645 | 3.370393753 | https://www.genecards.org/cgi-bin/carddisp.pl?gene=MK280073-645 |
| MK280073-646 | 3.370393753 | https://www.genecards.org/cgi-bin/carddisp.pl?gene=MK280073-646 |
| MK280073-647 | 3.370393753 | https://www.genecards.org/cgi-bin/carddisp.pl?gene=MK280073-647 |
| MK280073-648 | 3.370393753 | https://www.genecards.org/cgi-bin/carddisp.pl?gene=MK280073-648 |
| MK280073-649 | 3.370393753 | https://www.genecards.org/cgi-bin/carddisp.pl?gene=MK280073-649 |
| MK280073-650 | 3.370393753 | https://www.genecards.org/cgi-bin/carddisp.pl?gene=MK280073-650 |
| MK280073-651 | 3.370393753 | https://www.genecards.org/cgi-bin/carddisp.pl?gene=MK280073-651 |
| MK280073-652 | 3.370393753 | https://www.genecards.org/cgi-bin/carddisp.pl?gene=MK280073-652 |
| MK280073-653 | 3.370393753 | https://www.genecards.org/cgi-bin/carddisp.pl?gene=MK280073-653 |
| MK280073-657 | 3.370393753 | https://www.genecards.org/cgi-bin/carddisp.pl?gene=MK280073-657 |
| MK280073-658 | 3.370393753 | https://www.genecards.org/cgi-bin/carddisp.pl?gene=MK280073-658 |
| MK280073-660 | 3.370393753 | https://www.genecards.org/cgi-bin/carddisp.pl?gene=MK280073-660 |
| MK280073-661 | 3.370393753 | https://www.genecards.org/cgi-bin/carddisp.pl?gene=MK280073-661 |
| MK280073-662 | 3.370393753 | https://www.genecards.org/cgi-bin/carddisp.pl?gene=MK280073-662 |
| MK280073-663 | 3.370393753 | https://www.genecards.org/cgi-bin/carddisp.pl?gene=MK280073-663 |
| MK280073-664 | 3.370393753 | https://www.genecards.org/cgi-bin/carddisp.pl?gene=MK280073-664 |
| MK280073-665 | 3.370393753 | https://www.genecards.org/cgi-bin/carddisp.pl?gene=MK280073-665 |
| MK280073-666 | 3.370393753 | https://www.genecards.org/cgi-bin/carddisp.pl?gene=MK280073-666 |
| MK280073-667 | 3.370393753 | https://www.genecards.org/cgi-bin/carddisp.pl?gene=MK280073-667 |
| MK280073-668 | 3.370393753 | https://www.genecards.org/cgi-bin/carddisp.pl?gene=MK280073-668 |
| MK280073-669 | 3.370393753 | https://www.genecards.org/cgi-bin/carddisp.pl?gene=MK280073-669 |
| MK280073-671 | 3.370393753 | https://www.genecards.org/cgi-bin/carddisp.pl?gene=MK280073-671 |
| MK280073-672 | 3.370393753 | https://www.genecards.org/cgi-bin/carddisp.pl?gene=MK280073-672 |
| MK280073-673 | 3.370393753 | https://www.genecards.org/cgi-bin/carddisp.pl?gene=MK280073-673 |
| MK280073-675 | 3.370393753 | https://www.genecards.org/cgi-bin/carddisp.pl?gene=MK280073-675 |
| MK280073-677 | 3.370393753 | https://www.genecards.org/cgi-bin/carddisp.pl?gene=MK280073-677 |
| MK280073-679 | 3.370393753 | https://www.genecards.org/cgi-bin/carddisp.pl?gene=MK280073-679 |
| MK280073-680 | 3.370393753 | https://www.genecards.org/cgi-bin/carddisp.pl?gene=MK280073-680 |
| MK280073-681 | 3.370393753 | https://www.genecards.org/cgi-bin/carddisp.pl?gene=MK280073-681 |
| MK280073-682 | 3.370393753 | https://www.genecards.org/cgi-bin/carddisp.pl?gene=MK280073-682 |
| MK280073-684 | 3.370393753 | https://www.genecards.org/cgi-bin/carddisp.pl?gene=MK280073-684 |
| MK280073-685 | 3.370393753 | https://www.genecards.org/cgi-bin/carddisp.pl?gene=MK280073-685 |
| MK280073-687 | 3.370393753 | https://www.genecards.org/cgi-bin/carddisp.pl?gene=MK280073-687 |
| MK280073-690 | 3.370393753 | https://www.genecards.org/cgi-bin/carddisp.pl?gene=MK280073-690 |
| MK280073-692 | 3.370393753 | https://www.genecards.org/cgi-bin/carddisp.pl?gene=MK280073-692 |
| MK280073-693 | 3.370393753 | https://www.genecards.org/cgi-bin/carddisp.pl?gene=MK280073-693 |
| MK280073-694 | 3.370393753 | https://www.genecards.org/cgi-bin/carddisp.pl?gene=MK280073-694 |
| MK280073-695 | 3.370393753 | https://www.genecards.org/cgi-bin/carddisp.pl?gene=MK280073-695 |
| MK280073-696 | 3.370393753 | https://www.genecards.org/cgi-bin/carddisp.pl?gene=MK280073-696 |
| MK280073-697 | 3.370393753 | https://www.genecards.org/cgi-bin/carddisp.pl?gene=MK280073-697 |
| MK280073-698 | 3.370393753 | https://www.genecards.org/cgi-bin/carddisp.pl?gene=MK280073-698 |
| MK280073-699 | 3.370393753 | https://www.genecards.org/cgi-bin/carddisp.pl?gene=MK280073-699 |
| MK280073-700 | 3.370393753 | https://www.genecards.org/cgi-bin/carddisp.pl?gene=MK280073-700 |
| MK280073-701 | 3.370393753 | https://www.genecards.org/cgi-bin/carddisp.pl?gene=MK280073-701 |
| MK280073-702 | 3.370393753 | https://www.genecards.org/cgi-bin/carddisp.pl?gene=MK280073-702 |
| MK280073-703 | 3.370393753 | https://www.genecards.org/cgi-bin/carddisp.pl?gene=MK280073-703 |
| MK280073-704 | 3.370393753 | https://www.genecards.org/cgi-bin/carddisp.pl?gene=MK280073-704 |
| MK280073-705 | 3.370393753 | https://www.genecards.org/cgi-bin/carddisp.pl?gene=MK280073-705 |
| MK280073-706 | 3.370393753 | https://www.genecards.org/cgi-bin/carddisp.pl?gene=MK280073-706 |
| MK280073-707 | 3.370393753 | https://www.genecards.org/cgi-bin/carddisp.pl?gene=MK280073-707 |
| MK280073-708 | 3.370393753 | https://www.genecards.org/cgi-bin/carddisp.pl?gene=MK280073-708 |
| MK280073-709 | 3.370393753 | https://www.genecards.org/cgi-bin/carddisp.pl?gene=MK280073-709 |
| MK280073-710 | 3.370393753 | https://www.genecards.org/cgi-bin/carddisp.pl?gene=MK280073-710 |
| MK280073-711 | 3.370393753 | https://www.genecards.org/cgi-bin/carddisp.pl?gene=MK280073-711 |
| MK280073-712 | 3.370393753 | https://www.genecards.org/cgi-bin/carddisp.pl?gene=MK280073-712 |
| MK280073-713 | 3.370393753 | https://www.genecards.org/cgi-bin/carddisp.pl?gene=MK280073-713 |
| MK280073-714 | 3.370393753 | https://www.genecards.org/cgi-bin/carddisp.pl?gene=MK280073-714 |
| MK280073-715 | 3.370393753 | https://www.genecards.org/cgi-bin/carddisp.pl?gene=MK280073-715 |
| MK280073-716 | 3.370393753 | https://www.genecards.org/cgi-bin/carddisp.pl?gene=MK280073-716 |
| MK280073-717 | 3.370393753 | https://www.genecards.org/cgi-bin/carddisp.pl?gene=MK280073-717 |
| MK280073-718 | 3.370393753 | https://www.genecards.org/cgi-bin/carddisp.pl?gene=MK280073-718 |
| MK280073-719 | 3.370393753 | https://www.genecards.org/cgi-bin/carddisp.pl?gene=MK280073-719 |
| MK280073-720 | 3.370393753 | https://www.genecards.org/cgi-bin/carddisp.pl?gene=MK280073-720 |
| MK280073-721 | 3.370393753 | https://www.genecards.org/cgi-bin/carddisp.pl?gene=MK280073-721 |
| MK280073-722 | 3.370393753 | https://www.genecards.org/cgi-bin/carddisp.pl?gene=MK280073-722 |
| TIMP2 | 3.369617224 | https://www.genecards.org/cgi-bin/carddisp.pl?gene=TIMP2 |
| MIR19A | 3.367638588 | https://www.genecards.org/cgi-bin/carddisp.pl?gene=MIR19A |
| PON1 | 3.353440046 | https://www.genecards.org/cgi-bin/carddisp.pl?gene=PON1 |
| CFH | 3.348329306 | https://www.genecards.org/cgi-bin/carddisp.pl?gene=CFH |
| CAMP | 3.318599224 | https://www.genecards.org/cgi-bin/carddisp.pl?gene=CAMP |
| PARK7 | 3.316042423 | https://www.genecards.org/cgi-bin/carddisp.pl?gene=PARK7 |
| MFHAS1 | 3.316042423 | https://www.genecards.org/cgi-bin/carddisp.pl?gene=MFHAS1 |
| PPARG | 3.307022095 | https://www.genecards.org/cgi-bin/carddisp.pl?gene=PPARG |
| SMAD5-AS1 | 3.303615093 | https://www.genecards.org/cgi-bin/carddisp.pl?gene=SMAD5-AS1 |
| PIK3C2A | 3.291813135 | https://www.genecards.org/cgi-bin/carddisp.pl?gene=PIK3C2A |
| TGFB1 | 3.253546238 | https://www.genecards.org/cgi-bin/carddisp.pl?gene=TGFB1 |
| IL7 | 3.244293928 | https://www.genecards.org/cgi-bin/carddisp.pl?gene=IL7 |
| ENSG00000274760 | 3.240248203 | https://www.genecards.org/cgi-bin/carddisp.pl?gene=ENSG00000274760 |
| ENSG00000277967 | 3.240248203 | https://www.genecards.org/cgi-bin/carddisp.pl?gene=ENSG00000277967 |
| WAS | 3.21854353 | https://www.genecards.org/cgi-bin/carddisp.pl?gene=WAS |
| TLR8 | 3.206163168 | https://www.genecards.org/cgi-bin/carddisp.pl?gene=TLR8 |
| PLAUR | 3.20477128 | https://www.genecards.org/cgi-bin/carddisp.pl?gene=PLAUR |
| SNHG31 | 3.202709675 | https://www.genecards.org/cgi-bin/carddisp.pl?gene=SNHG31 |
| PCSK9 | 3.189791679 | https://www.genecards.org/cgi-bin/carddisp.pl?gene=PCSK9 |
| IL7R | 3.188418388 | https://www.genecards.org/cgi-bin/carddisp.pl?gene=IL7R |
| CD40 | 3.188410759 | https://www.genecards.org/cgi-bin/carddisp.pl?gene=CD40 |
| HIF1A | 3.187903166 | https://www.genecards.org/cgi-bin/carddisp.pl?gene=HIF1A |
| POMC | 3.175040722 | https://www.genecards.org/cgi-bin/carddisp.pl?gene=POMC |
| CD86 | 3.167502165 | https://www.genecards.org/cgi-bin/carddisp.pl?gene=CD86 |
| INS | 3.161165953 | https://www.genecards.org/cgi-bin/carddisp.pl?gene=INS |
| G6PD | 3.151050568 | https://www.genecards.org/cgi-bin/carddisp.pl?gene=G6PD |
| FASLG | 3.149103642 | https://www.genecards.org/cgi-bin/carddisp.pl?gene=FASLG |
| CCL5 | 3.145208597 | https://www.genecards.org/cgi-bin/carddisp.pl?gene=CCL5 |
| CXCL1 | 3.123566628 | https://www.genecards.org/cgi-bin/carddisp.pl?gene=CXCL1 |
| CFI | 3.115394831 | https://www.genecards.org/cgi-bin/carddisp.pl?gene=CFI |
| IL2RB | 3.111529112 | https://www.genecards.org/cgi-bin/carddisp.pl?gene=IL2RB |
| SFTPB | 3.108539581 | https://www.genecards.org/cgi-bin/carddisp.pl?gene=SFTPB |
| MIR223 | 3.101330757 | https://www.genecards.org/cgi-bin/carddisp.pl?gene=MIR223 |
| IL1RAPL2 | 3.096954823 | https://www.genecards.org/cgi-bin/carddisp.pl?gene=IL1RAPL2 |
| ACOD1 | 3.089308023 | https://www.genecards.org/cgi-bin/carddisp.pl?gene=ACOD1 |
| TFRC | 3.065500736 | https://www.genecards.org/cgi-bin/carddisp.pl?gene=TFRC |
| CHD7 | 3.038943529 | https://www.genecards.org/cgi-bin/carddisp.pl?gene=CHD7 |
| MIR145 | 3.03691721 | https://www.genecards.org/cgi-bin/carddisp.pl?gene=MIR145 |
| MIR758 | 3.03691721 | https://www.genecards.org/cgi-bin/carddisp.pl?gene=MIR758 |
| CTSG | 3.03217721 | https://www.genecards.org/cgi-bin/carddisp.pl?gene=CTSG |
| ANGPT1 | 3.030390501 | https://www.genecards.org/cgi-bin/carddisp.pl?gene=ANGPT1 |
| RELA | 2.985467911 | https://www.genecards.org/cgi-bin/carddisp.pl?gene=RELA |
| MIR106B | 2.985294819 | https://www.genecards.org/cgi-bin/carddisp.pl?gene=MIR106B |
| MMP2 | 2.967144966 | https://www.genecards.org/cgi-bin/carddisp.pl?gene=MMP2 |
| MIR199A2 | 2.955548763 | https://www.genecards.org/cgi-bin/carddisp.pl?gene=MIR199A2 |
| MIR199A1 | 2.955548763 | https://www.genecards.org/cgi-bin/carddisp.pl?gene=MIR199A1 |
| LRBA | 2.954989433 | https://www.genecards.org/cgi-bin/carddisp.pl?gene=LRBA |
| HSPA4 | 2.951766491 | https://www.genecards.org/cgi-bin/carddisp.pl?gene=HSPA4 |
| F2R | 2.948045492 | https://www.genecards.org/cgi-bin/carddisp.pl?gene=F2R |
| SIRT1 | 2.920924187 | https://www.genecards.org/cgi-bin/carddisp.pl?gene=SIRT1 |
| RMRP | 2.92081666 | https://www.genecards.org/cgi-bin/carddisp.pl?gene=RMRP |
| IL3 | 2.889513254 | https://www.genecards.org/cgi-bin/carddisp.pl?gene=IL3 |
| IL5 | 2.871613741 | https://www.genecards.org/cgi-bin/carddisp.pl?gene=IL5 |
| IL1R2 | 2.871186256 | https://www.genecards.org/cgi-bin/carddisp.pl?gene=IL1R2 |
| ADA | 2.862682104 | https://www.genecards.org/cgi-bin/carddisp.pl?gene=ADA |
| IL27 | 2.857913494 | https://www.genecards.org/cgi-bin/carddisp.pl?gene=IL27 |
| AZU1 | 2.84878397 | https://www.genecards.org/cgi-bin/carddisp.pl?gene=AZU1 |
| TIRAP | 2.836951733 | https://www.genecards.org/cgi-bin/carddisp.pl?gene=TIRAP |
| ACTA1 | 2.83099556 | https://www.genecards.org/cgi-bin/carddisp.pl?gene=ACTA1 |
| SRC | 2.824073315 | https://www.genecards.org/cgi-bin/carddisp.pl?gene=SRC |
| CD79A | 2.817914963 | https://www.genecards.org/cgi-bin/carddisp.pl?gene=CD79A |
| SAMD9 | 2.812965393 | https://www.genecards.org/cgi-bin/carddisp.pl?gene=SAMD9 |
| TNFRSF1A | 2.80102396 | https://www.genecards.org/cgi-bin/carddisp.pl?gene=TNFRSF1A |
| FLT1 | 2.756695986 | https://www.genecards.org/cgi-bin/carddisp.pl?gene=FLT1 |
| SOD2 | 2.743993044 | https://www.genecards.org/cgi-bin/carddisp.pl?gene=SOD2 |
| AVP | 2.741137505 | https://www.genecards.org/cgi-bin/carddisp.pl?gene=AVP |
| DANCR | 2.737910986 | https://www.genecards.org/cgi-bin/carddisp.pl?gene=DANCR |
| SNHG14 | 2.735752583 | https://www.genecards.org/cgi-bin/carddisp.pl?gene=SNHG14 |
| CXCR4 | 2.729949713 | https://www.genecards.org/cgi-bin/carddisp.pl?gene=CXCR4 |
| TGM1 | 2.723816156 | https://www.genecards.org/cgi-bin/carddisp.pl?gene=TGM1 |
| CAT | 2.721418858 | https://www.genecards.org/cgi-bin/carddisp.pl?gene=CAT |
| BMP6 | 2.717900991 | https://www.genecards.org/cgi-bin/carddisp.pl?gene=BMP6 |
| CTCF | 2.712126255 | https://www.genecards.org/cgi-bin/carddisp.pl?gene=CTCF |
| MIR23B | 2.706572294 | https://www.genecards.org/cgi-bin/carddisp.pl?gene=MIR23B |
| S100A8 | 2.705786705 | https://www.genecards.org/cgi-bin/carddisp.pl?gene=S100A8 |
| CD163 | 2.702189684 | https://www.genecards.org/cgi-bin/carddisp.pl?gene=CD163 |
| G6PC3 | 2.694770098 | https://www.genecards.org/cgi-bin/carddisp.pl?gene=G6PC3 |
| PLAT | 2.690485239 | https://www.genecards.org/cgi-bin/carddisp.pl?gene=PLAT |
| S100B | 2.689227581 | https://www.genecards.org/cgi-bin/carddisp.pl?gene=S100B |
| IL10RA | 2.685225248 | https://www.genecards.org/cgi-bin/carddisp.pl?gene=IL10RA |
| C3 | 2.68169117 | https://www.genecards.org/cgi-bin/carddisp.pl?gene=C3 |
| ESM1 | 2.679022312 | https://www.genecards.org/cgi-bin/carddisp.pl?gene=ESM1 |
| CXCL5 | 2.665035248 | https://www.genecards.org/cgi-bin/carddisp.pl?gene=CXCL5 |
| AKT1 | 2.659132004 | https://www.genecards.org/cgi-bin/carddisp.pl?gene=AKT1 |
| ITGB4 | 2.656554699 | https://www.genecards.org/cgi-bin/carddisp.pl?gene=ITGB4 |
| GHR | 2.648556709 | https://www.genecards.org/cgi-bin/carddisp.pl?gene=GHR |
| HOTAIRM1 | 2.648333788 | https://www.genecards.org/cgi-bin/carddisp.pl?gene=HOTAIRM1 |
| RELB | 2.638748884 | https://www.genecards.org/cgi-bin/carddisp.pl?gene=RELB |
| IRF1 | 2.622414351 | https://www.genecards.org/cgi-bin/carddisp.pl?gene=IRF1 |
| CRNDE | 2.610496759 | https://www.genecards.org/cgi-bin/carddisp.pl?gene=CRNDE |
| SERPINF2 | 2.600691319 | https://www.genecards.org/cgi-bin/carddisp.pl?gene=SERPINF2 |
| RAG1 | 2.594076633 | https://www.genecards.org/cgi-bin/carddisp.pl?gene=RAG1 |
| RETN | 2.590711117 | https://www.genecards.org/cgi-bin/carddisp.pl?gene=RETN |
| CFP | 2.590107679 | https://www.genecards.org/cgi-bin/carddisp.pl?gene=CFP |
| HP | 2.578532219 | https://www.genecards.org/cgi-bin/carddisp.pl?gene=HP |
| S100A12 | 2.566099644 | https://www.genecards.org/cgi-bin/carddisp.pl?gene=S100A12 |
| SIGLEC5 | 2.565942287 | https://www.genecards.org/cgi-bin/carddisp.pl?gene=SIGLEC5 |
| CYBB | 2.565490484 | https://www.genecards.org/cgi-bin/carddisp.pl?gene=CYBB |
| C1S | 2.550442696 | https://www.genecards.org/cgi-bin/carddisp.pl?gene=C1S |
| C4A | 2.543600798 | https://www.genecards.org/cgi-bin/carddisp.pl?gene=C4A |
| IKBKB | 2.542489052 | https://www.genecards.org/cgi-bin/carddisp.pl?gene=IKBKB |
| VDR | 2.534542561 | https://www.genecards.org/cgi-bin/carddisp.pl?gene=VDR |
| C2 | 2.530549049 | https://www.genecards.org/cgi-bin/carddisp.pl?gene=C2 |
| KRT10-AS1 | 2.530549049 | https://www.genecards.org/cgi-bin/carddisp.pl?gene=KRT10-AS1 |
| IFNG-AS1 | 2.530549049 | https://www.genecards.org/cgi-bin/carddisp.pl?gene=IFNG-AS1 |
| FOXD2-AS1 | 2.530549049 | https://www.genecards.org/cgi-bin/carddisp.pl?gene=FOXD2-AS1 |
| DLGAP4-AS1 | 2.530549049 | https://www.genecards.org/cgi-bin/carddisp.pl?gene=DLGAP4-AS1 |
| LOC126862559 | 2.530549049 | https://www.genecards.org/cgi-bin/carddisp.pl?gene=LOC126862559 |
| TNFRSF13B | 2.527512074 | https://www.genecards.org/cgi-bin/carddisp.pl?gene=TNFRSF13B |
| IGF1 | 2.525712013 | https://www.genecards.org/cgi-bin/carddisp.pl?gene=IGF1 |
| MIR23A | 2.520082474 | https://www.genecards.org/cgi-bin/carddisp.pl?gene=MIR23A |
| TTR | 2.517982006 | https://www.genecards.org/cgi-bin/carddisp.pl?gene=TTR |
| PTAFR | 2.512717485 | https://www.genecards.org/cgi-bin/carddisp.pl?gene=PTAFR |
| TFF3 | 2.508358955 | https://www.genecards.org/cgi-bin/carddisp.pl?gene=TFF3 |
| REL | 2.500374556 | https://www.genecards.org/cgi-bin/carddisp.pl?gene=REL |
| TNNI3 | 2.497000217 | https://www.genecards.org/cgi-bin/carddisp.pl?gene=TNNI3 |
| BAX | 2.490682602 | https://www.genecards.org/cgi-bin/carddisp.pl?gene=BAX |
| MIR147B | 2.482020378 | https://www.genecards.org/cgi-bin/carddisp.pl?gene=MIR147B |
| TAB1 | 2.466662645 | https://www.genecards.org/cgi-bin/carddisp.pl?gene=TAB1 |
| ITIH4 | 2.465892315 | https://www.genecards.org/cgi-bin/carddisp.pl?gene=ITIH4 |
| IL12B | 2.455356121 | https://www.genecards.org/cgi-bin/carddisp.pl?gene=IL12B |
| ANXA5 | 2.443319559 | https://www.genecards.org/cgi-bin/carddisp.pl?gene=ANXA5 |
| MIR494 | 2.42269063 | https://www.genecards.org/cgi-bin/carddisp.pl?gene=MIR494 |
| REG1A | 2.416448116 | https://www.genecards.org/cgi-bin/carddisp.pl?gene=REG1A |
| IRF3 | 2.416081429 | https://www.genecards.org/cgi-bin/carddisp.pl?gene=IRF3 |
| APOA1 | 2.415377617 | https://www.genecards.org/cgi-bin/carddisp.pl?gene=APOA1 |
| MMP8 | 2.410068274 | https://www.genecards.org/cgi-bin/carddisp.pl?gene=MMP8 |
| LEP | 2.400057793 | https://www.genecards.org/cgi-bin/carddisp.pl?gene=LEP |
| THBS1 | 2.393795013 | https://www.genecards.org/cgi-bin/carddisp.pl?gene=THBS1 |
| CYTOR | 2.392745495 | https://www.genecards.org/cgi-bin/carddisp.pl?gene=CYTOR |
| APOE | 2.388457775 | https://www.genecards.org/cgi-bin/carddisp.pl?gene=APOE |
| DEFB4A | 2.386326313 | https://www.genecards.org/cgi-bin/carddisp.pl?gene=DEFB4A |
| AMH | 2.384821177 | https://www.genecards.org/cgi-bin/carddisp.pl?gene=AMH |
| MIR451A | 2.38245368 | https://www.genecards.org/cgi-bin/carddisp.pl?gene=MIR451A |
| ENO2 | 2.375984192 | https://www.genecards.org/cgi-bin/carddisp.pl?gene=ENO2 |
| TRAF3 | 2.374819279 | https://www.genecards.org/cgi-bin/carddisp.pl?gene=TRAF3 |
| MIR203A | 2.374819279 | https://www.genecards.org/cgi-bin/carddisp.pl?gene=MIR203A |
| IRF5 | 2.374368906 | https://www.genecards.org/cgi-bin/carddisp.pl?gene=IRF5 |
| NEU1 | 2.372017622 | https://www.genecards.org/cgi-bin/carddisp.pl?gene=NEU1 |
| MIRLET7B | 2.363327503 | https://www.genecards.org/cgi-bin/carddisp.pl?gene=MIRLET7B |
| SYK | 2.361505747 | https://www.genecards.org/cgi-bin/carddisp.pl?gene=SYK |
| PF4 | 2.355759382 | https://www.genecards.org/cgi-bin/carddisp.pl?gene=PF4 |
| MGAT3-AS1 | 2.352899075 | https://www.genecards.org/cgi-bin/carddisp.pl?gene=MGAT3-AS1 |
| TNFRSF1B | 2.352401733 | https://www.genecards.org/cgi-bin/carddisp.pl?gene=TNFRSF1B |
| S100A9 | 2.352401733 | https://www.genecards.org/cgi-bin/carddisp.pl?gene=S100A9 |
| CETP | 2.346389532 | https://www.genecards.org/cgi-bin/carddisp.pl?gene=CETP |
| FCGR3A | 2.345181704 | https://www.genecards.org/cgi-bin/carddisp.pl?gene=FCGR3A |
| APC | 2.34337306 | https://www.genecards.org/cgi-bin/carddisp.pl?gene=APC |
| HBEGF | 2.340058088 | https://www.genecards.org/cgi-bin/carddisp.pl?gene=HBEGF |
| TJP1 | 2.339832306 | https://www.genecards.org/cgi-bin/carddisp.pl?gene=TJP1 |
| PLAU | 2.336714506 | https://www.genecards.org/cgi-bin/carddisp.pl?gene=PLAU |
| IL4R | 2.332644701 | https://www.genecards.org/cgi-bin/carddisp.pl?gene=IL4R |
| FN1 | 2.327742577 | https://www.genecards.org/cgi-bin/carddisp.pl?gene=FN1 |
| MIR98 | 2.322987318 | https://www.genecards.org/cgi-bin/carddisp.pl?gene=MIR98 |
| NCF1 | 2.322925091 | https://www.genecards.org/cgi-bin/carddisp.pl?gene=NCF1 |
| ITGA4 | 2.311447382 | https://www.genecards.org/cgi-bin/carddisp.pl?gene=ITGA4 |
| MIR143 | 2.310972691 | https://www.genecards.org/cgi-bin/carddisp.pl?gene=MIR143 |
| AHSG | 2.302978992 | https://www.genecards.org/cgi-bin/carddisp.pl?gene=AHSG |
| MIR574 | 2.298434734 | https://www.genecards.org/cgi-bin/carddisp.pl?gene=MIR574 |
| ADAM17 | 2.298070431 | https://www.genecards.org/cgi-bin/carddisp.pl?gene=ADAM17 |
| MAPK1 | 2.296574116 | https://www.genecards.org/cgi-bin/carddisp.pl?gene=MAPK1 |
| MIR92A1 | 2.287026405 | https://www.genecards.org/cgi-bin/carddisp.pl?gene=MIR92A1 |
| MIR92A2 | 2.287026405 | https://www.genecards.org/cgi-bin/carddisp.pl?gene=MIR92A2 |
| NAMPT | 2.286256313 | https://www.genecards.org/cgi-bin/carddisp.pl?gene=NAMPT |
| SNHG5 | 2.27890873 | https://www.genecards.org/cgi-bin/carddisp.pl?gene=SNHG5 |
| NR3C1 | 2.27613306 | https://www.genecards.org/cgi-bin/carddisp.pl?gene=NR3C1 |
| TEK | 2.265286446 | https://www.genecards.org/cgi-bin/carddisp.pl?gene=TEK |
| GHRL | 2.264907122 | https://www.genecards.org/cgi-bin/carddisp.pl?gene=GHRL |
| KRT14 | 2.263333797 | https://www.genecards.org/cgi-bin/carddisp.pl?gene=KRT14 |
| MIR495 | 2.260582924 | https://www.genecards.org/cgi-bin/carddisp.pl?gene=MIR495 |
| AQP5 | 2.259142399 | https://www.genecards.org/cgi-bin/carddisp.pl?gene=AQP5 |
| HLA-DRA | 2.251609564 | https://www.genecards.org/cgi-bin/carddisp.pl?gene=HLA-DRA |
| IL2RG | 2.249597549 | https://www.genecards.org/cgi-bin/carddisp.pl?gene=IL2RG |
| HBG2 | 2.24360466 | https://www.genecards.org/cgi-bin/carddisp.pl?gene=HBG2 |
| OCLN | 2.23567152 | https://www.genecards.org/cgi-bin/carddisp.pl?gene=OCLN |
| CARMN | 2.23567152 | https://www.genecards.org/cgi-bin/carddisp.pl?gene=CARMN |
| NOS1 | 2.234544277 | https://www.genecards.org/cgi-bin/carddisp.pl?gene=NOS1 |
| CST3 | 2.229989529 | https://www.genecards.org/cgi-bin/carddisp.pl?gene=CST3 |
| GPR182 | 2.226893425 | https://www.genecards.org/cgi-bin/carddisp.pl?gene=GPR182 |
| TLR6 | 2.219742537 | https://www.genecards.org/cgi-bin/carddisp.pl?gene=TLR6 |
| PGF | 2.214164257 | https://www.genecards.org/cgi-bin/carddisp.pl?gene=PGF |
| IL11 | 2.214164257 | https://www.genecards.org/cgi-bin/carddisp.pl?gene=IL11 |
| MIR106A | 2.213754654 | https://www.genecards.org/cgi-bin/carddisp.pl?gene=MIR106A |
| DEFB130A | 2.207589626 | https://www.genecards.org/cgi-bin/carddisp.pl?gene=DEFB130A |
| SDC1 | 2.2054739 | https://www.genecards.org/cgi-bin/carddisp.pl?gene=SDC1 |
| C5 | 2.201166868 | https://www.genecards.org/cgi-bin/carddisp.pl?gene=C5 |
| CARD11 | 2.199988604 | https://www.genecards.org/cgi-bin/carddisp.pl?gene=CARD11 |
| SLX1A-SULT1A3 | 2.199988604 | https://www.genecards.org/cgi-bin/carddisp.pl?gene=SLX1A-SULT1A3 |
| XDH | 2.19624424 | https://www.genecards.org/cgi-bin/carddisp.pl?gene=XDH |
| LINC00472 | 2.187883854 | https://www.genecards.org/cgi-bin/carddisp.pl?gene=LINC00472 |
| DEFB1 | 2.185339451 | https://www.genecards.org/cgi-bin/carddisp.pl?gene=DEFB1 |
| HLA-DRB1 | 2.183532715 | https://www.genecards.org/cgi-bin/carddisp.pl?gene=HLA-DRB1 |
| WNT5A | 2.180636406 | https://www.genecards.org/cgi-bin/carddisp.pl?gene=WNT5A |
| NFKBIL1 | 2.170496941 | https://www.genecards.org/cgi-bin/carddisp.pl?gene=NFKBIL1 |
| GP1BA | 2.166361094 | https://www.genecards.org/cgi-bin/carddisp.pl?gene=GP1BA |
| CYP4F22 | 2.165741444 | https://www.genecards.org/cgi-bin/carddisp.pl?gene=CYP4F22 |
| CDH5 | 2.160250664 | https://www.genecards.org/cgi-bin/carddisp.pl?gene=CDH5 |
| CHUK | 2.157116413 | https://www.genecards.org/cgi-bin/carddisp.pl?gene=CHUK |
| MIR187 | 2.156570911 | https://www.genecards.org/cgi-bin/carddisp.pl?gene=MIR187 |
| HAVCR2 | 2.146111012 | https://www.genecards.org/cgi-bin/carddisp.pl?gene=HAVCR2 |
| PPARA | 2.146111012 | https://www.genecards.org/cgi-bin/carddisp.pl?gene=PPARA |
| ABCA3 | 2.137327909 | https://www.genecards.org/cgi-bin/carddisp.pl?gene=ABCA3 |
| BLNK | 2.137327909 | https://www.genecards.org/cgi-bin/carddisp.pl?gene=BLNK |
| CD79B | 2.137327909 | https://www.genecards.org/cgi-bin/carddisp.pl?gene=CD79B |
| IGHM | 2.137327909 | https://www.genecards.org/cgi-bin/carddisp.pl?gene=IGHM |
| HLA-A | 2.134694338 | https://www.genecards.org/cgi-bin/carddisp.pl?gene=HLA-A |
| LYZ | 2.132809639 | https://www.genecards.org/cgi-bin/carddisp.pl?gene=LYZ |
| KNG1 | 2.128120899 | https://www.genecards.org/cgi-bin/carddisp.pl?gene=KNG1 |
| GC | 2.126968145 | https://www.genecards.org/cgi-bin/carddisp.pl?gene=GC |
| MIR149 | 2.123353243 | https://www.genecards.org/cgi-bin/carddisp.pl?gene=MIR149 |
| MIRLET7I | 2.123353243 | https://www.genecards.org/cgi-bin/carddisp.pl?gene=MIRLET7I |
| PRL | 2.122212648 | https://www.genecards.org/cgi-bin/carddisp.pl?gene=PRL |
| STAT1 | 2.118620872 | https://www.genecards.org/cgi-bin/carddisp.pl?gene=STAT1 |
| IL33 | 2.116399527 | https://www.genecards.org/cgi-bin/carddisp.pl?gene=IL33 |
| PPIG | 2.105062485 | https://www.genecards.org/cgi-bin/carddisp.pl?gene=PPIG |
| FGA | 2.105050564 | https://www.genecards.org/cgi-bin/carddisp.pl?gene=FGA |
| ALG12 | 2.102361441 | https://www.genecards.org/cgi-bin/carddisp.pl?gene=ALG12 |
| ADORA2A | 2.100687981 | https://www.genecards.org/cgi-bin/carddisp.pl?gene=ADORA2A |
| HSP90AA1 | 2.092811108 | https://www.genecards.org/cgi-bin/carddisp.pl?gene=HSP90AA1 |
| DEFA1 | 2.087239027 | https://www.genecards.org/cgi-bin/carddisp.pl?gene=DEFA1 |
| HSPA1B | 2.084882736 | https://www.genecards.org/cgi-bin/carddisp.pl?gene=HSPA1B |
| FER | 2.079489946 | https://www.genecards.org/cgi-bin/carddisp.pl?gene=FER |
| TRL-TAG1-1 | 2.078730822 | https://www.genecards.org/cgi-bin/carddisp.pl?gene=TRL-TAG1-1 |
| PTPRC | 2.073437452 | https://www.genecards.org/cgi-bin/carddisp.pl?gene=PTPRC |
| TFPI | 2.073183298 | https://www.genecards.org/cgi-bin/carddisp.pl?gene=TFPI |
| MIR511 | 2.066858768 | https://www.genecards.org/cgi-bin/carddisp.pl?gene=MIR511 |
| CHI3L1 | 2.064081669 | https://www.genecards.org/cgi-bin/carddisp.pl?gene=CHI3L1 |
| MIR545 | 2.053861618 | https://www.genecards.org/cgi-bin/carddisp.pl?gene=MIR545 |
| CPB2 | 2.053759098 | https://www.genecards.org/cgi-bin/carddisp.pl?gene=CPB2 |
| CXCR3 | 2.049019575 | https://www.genecards.org/cgi-bin/carddisp.pl?gene=CXCR3 |
| IPO9-AS1 | 2.048371792 | https://www.genecards.org/cgi-bin/carddisp.pl?gene=IPO9-AS1 |
| H2AC18 | 2.043632746 | https://www.genecards.org/cgi-bin/carddisp.pl?gene=H2AC18 |
| S1PR3 | 2.041324854 | https://www.genecards.org/cgi-bin/carddisp.pl?gene=S1PR3 |
| CHIT1 | 2.040578604 | https://www.genecards.org/cgi-bin/carddisp.pl?gene=CHIT1 |
| IL18R1 | 2.039317369 | https://www.genecards.org/cgi-bin/carddisp.pl?gene=IL18R1 |
| FURIN | 2.036771297 | https://www.genecards.org/cgi-bin/carddisp.pl?gene=FURIN |
| MIR335 | 2.013820171 | https://www.genecards.org/cgi-bin/carddisp.pl?gene=MIR335 |
| MAPK3 | 2.012953997 | https://www.genecards.org/cgi-bin/carddisp.pl?gene=MAPK3 |
| SPI1 | 2.011250496 | https://www.genecards.org/cgi-bin/carddisp.pl?gene=SPI1 |
| MMP1 | 1.999551773 | https://www.genecards.org/cgi-bin/carddisp.pl?gene=MMP1 |
| CHKB-CPT1B | 1.999551773 | https://www.genecards.org/cgi-bin/carddisp.pl?gene=CHKB-CPT1B |
| TP53 | 1.996628523 | https://www.genecards.org/cgi-bin/carddisp.pl?gene=TP53 |
| MT-ND1 | 1.995329738 | https://www.genecards.org/cgi-bin/carddisp.pl?gene=MT-ND1 |
| ADAM10 | 1.993161917 | https://www.genecards.org/cgi-bin/carddisp.pl?gene=ADAM10 |
| MIR320A | 1.987227678 | https://www.genecards.org/cgi-bin/carddisp.pl?gene=MIR320A |
| VIP | 1.983078361 | https://www.genecards.org/cgi-bin/carddisp.pl?gene=VIP |
| MSN | 1.977902412 | https://www.genecards.org/cgi-bin/carddisp.pl?gene=MSN |
| HSPA12B | 1.973944664 | https://www.genecards.org/cgi-bin/carddisp.pl?gene=HSPA12B |
| TUG1 | 1.969840527 | https://www.genecards.org/cgi-bin/carddisp.pl?gene=TUG1 |
| TLR10 | 1.96748364 | https://www.genecards.org/cgi-bin/carddisp.pl?gene=TLR10 |
| CFD | 1.96739316 | https://www.genecards.org/cgi-bin/carddisp.pl?gene=CFD |
| MRTFA | 1.96628499 | https://www.genecards.org/cgi-bin/carddisp.pl?gene=MRTFA |
| SLC17A5 | 1.945505857 | https://www.genecards.org/cgi-bin/carddisp.pl?gene=SLC17A5 |
| CIRBP | 1.932171702 | https://www.genecards.org/cgi-bin/carddisp.pl?gene=CIRBP |
| FPR1 | 1.928204417 | https://www.genecards.org/cgi-bin/carddisp.pl?gene=FPR1 |
| CXCL12 | 1.928204417 | https://www.genecards.org/cgi-bin/carddisp.pl?gene=CXCL12 |
| CR1 | 1.92263031 | https://www.genecards.org/cgi-bin/carddisp.pl?gene=CR1 |
| HSPA1A | 1.921775818 | https://www.genecards.org/cgi-bin/carddisp.pl?gene=HSPA1A |
| PTMA | 1.921775818 | https://www.genecards.org/cgi-bin/carddisp.pl?gene=PTMA |
| LIF | 1.921029568 | https://www.genecards.org/cgi-bin/carddisp.pl?gene=LIF |
| GJB2 | 1.919530153 | https://www.genecards.org/cgi-bin/carddisp.pl?gene=GJB2 |
| JUN | 1.918964863 | https://www.genecards.org/cgi-bin/carddisp.pl?gene=JUN |
| GAS6 | 1.91752243 | https://www.genecards.org/cgi-bin/carddisp.pl?gene=GAS6 |
| MIR26B | 1.91752243 | https://www.genecards.org/cgi-bin/carddisp.pl?gene=MIR26B |
| CSF1 | 1.912199497 | https://www.genecards.org/cgi-bin/carddisp.pl?gene=CSF1 |
| CASP8 | 1.90902555 | https://www.genecards.org/cgi-bin/carddisp.pl?gene=CASP8 |
| SNORD15A | 1.90902555 | https://www.genecards.org/cgi-bin/carddisp.pl?gene=SNORD15A |
| IRF7 | 1.905104995 | https://www.genecards.org/cgi-bin/carddisp.pl?gene=IRF7 |
| MIR200C | 1.905104995 | https://www.genecards.org/cgi-bin/carddisp.pl?gene=MIR200C |
| MIR200B | 1.905104995 | https://www.genecards.org/cgi-bin/carddisp.pl?gene=MIR200B |
| MIR203B | 1.905104995 | https://www.genecards.org/cgi-bin/carddisp.pl?gene=MIR203B |
| MIR223HG | 1.905104995 | https://www.genecards.org/cgi-bin/carddisp.pl?gene=MIR223HG |
| UCP2 | 1.904625654 | https://www.genecards.org/cgi-bin/carddisp.pl?gene=UCP2 |
| C9 | 1.893445253 | https://www.genecards.org/cgi-bin/carddisp.pl?gene=C9 |
| KRT5 | 1.892809868 | https://www.genecards.org/cgi-bin/carddisp.pl?gene=KRT5 |
| SFTPD | 1.88857317 | https://www.genecards.org/cgi-bin/carddisp.pl?gene=SFTPD |
| P2RX7 | 1.886948347 | https://www.genecards.org/cgi-bin/carddisp.pl?gene=P2RX7 |
| GRK5 | 1.886948347 | https://www.genecards.org/cgi-bin/carddisp.pl?gene=GRK5 |
| TXN | 1.886802197 | https://www.genecards.org/cgi-bin/carddisp.pl?gene=TXN |
| RAG2 | 1.884113193 | https://www.genecards.org/cgi-bin/carddisp.pl?gene=RAG2 |
| BID | 1.880430937 | https://www.genecards.org/cgi-bin/carddisp.pl?gene=BID |
| CFB | 1.880175114 | https://www.genecards.org/cgi-bin/carddisp.pl?gene=CFB |
| STAT5A | 1.878501654 | https://www.genecards.org/cgi-bin/carddisp.pl?gene=STAT5A |
| ITGAL | 1.873342514 | https://www.genecards.org/cgi-bin/carddisp.pl?gene=ITGAL |
| MEFV | 1.870943546 | https://www.genecards.org/cgi-bin/carddisp.pl?gene=MEFV |
| MIR210 | 1.870943546 | https://www.genecards.org/cgi-bin/carddisp.pl?gene=MIR210 |
| MIR499A | 1.870943546 | https://www.genecards.org/cgi-bin/carddisp.pl?gene=MIR499A |
| CEACAM1 | 1.867473006 | https://www.genecards.org/cgi-bin/carddisp.pl?gene=CEACAM1 |
| CYBA | 1.858500361 | https://www.genecards.org/cgi-bin/carddisp.pl?gene=CYBA |
| DENND3 | 1.857388496 | https://www.genecards.org/cgi-bin/carddisp.pl?gene=DENND3 |
| NCLN | 1.857388496 | https://www.genecards.org/cgi-bin/carddisp.pl?gene=NCLN |
| TBATA | 1.857388496 | https://www.genecards.org/cgi-bin/carddisp.pl?gene=TBATA |
| PLK1 | 1.854395747 | https://www.genecards.org/cgi-bin/carddisp.pl?gene=PLK1 |
| HABP2 | 1.846468449 | https://www.genecards.org/cgi-bin/carddisp.pl?gene=HABP2 |
| SBDS | 1.84626317 | https://www.genecards.org/cgi-bin/carddisp.pl?gene=SBDS |
| CCR5 | 1.844719648 | https://www.genecards.org/cgi-bin/carddisp.pl?gene=CCR5 |
| IL22 | 1.843327045 | https://www.genecards.org/cgi-bin/carddisp.pl?gene=IL22 |
| F2RL1 | 1.842095137 | https://www.genecards.org/cgi-bin/carddisp.pl?gene=F2RL1 |
| MIR346 | 1.837597132 | https://www.genecards.org/cgi-bin/carddisp.pl?gene=MIR346 |
| SERPINA3 | 1.835675478 | https://www.genecards.org/cgi-bin/carddisp.pl?gene=SERPINA3 |
| CASP1 | 1.834529638 | https://www.genecards.org/cgi-bin/carddisp.pl?gene=CASP1 |
| FABP1 | 1.829689384 | https://www.genecards.org/cgi-bin/carddisp.pl?gene=FABP1 |
| HOTTIP | 1.826991081 | https://www.genecards.org/cgi-bin/carddisp.pl?gene=HOTTIP |
| SFTA3 | 1.822535038 | https://www.genecards.org/cgi-bin/carddisp.pl?gene=SFTA3 |
| LINC01618 | 1.822535038 | https://www.genecards.org/cgi-bin/carddisp.pl?gene=LINC01618 |
| MIR4772 | 1.822535038 | https://www.genecards.org/cgi-bin/carddisp.pl?gene=MIR4772 |
| EZH2 | 1.819422126 | https://www.genecards.org/cgi-bin/carddisp.pl?gene=EZH2 |
| MIR34B | 1.819422126 | https://www.genecards.org/cgi-bin/carddisp.pl?gene=MIR34B |
| DCLRE1C | 1.819128871 | https://www.genecards.org/cgi-bin/carddisp.pl?gene=DCLRE1C |
| EDNRA | 1.81569159 | https://www.genecards.org/cgi-bin/carddisp.pl?gene=EDNRA |
| AQP1 | 1.811056256 | https://www.genecards.org/cgi-bin/carddisp.pl?gene=AQP1 |
| BCL2L11 | 1.811056256 | https://www.genecards.org/cgi-bin/carddisp.pl?gene=BCL2L11 |
| ALOX12B | 1.810114861 | https://www.genecards.org/cgi-bin/carddisp.pl?gene=ALOX12B |
| ALOXE3 | 1.810114861 | https://www.genecards.org/cgi-bin/carddisp.pl?gene=ALOXE3 |
| NIPAL4 | 1.810114861 | https://www.genecards.org/cgi-bin/carddisp.pl?gene=NIPAL4 |
| KITLG | 1.806782126 | https://www.genecards.org/cgi-bin/carddisp.pl?gene=KITLG |
| SERPINA6 | 1.800098419 | https://www.genecards.org/cgi-bin/carddisp.pl?gene=SERPINA6 |
| SOCS3 | 1.795994163 | https://www.genecards.org/cgi-bin/carddisp.pl?gene=SOCS3 |
| PIK3R1 | 1.794069886 | https://www.genecards.org/cgi-bin/carddisp.pl?gene=PIK3R1 |
| CTNNB1 | 1.793938518 | https://www.genecards.org/cgi-bin/carddisp.pl?gene=CTNNB1 |
| IL15 | 1.791747212 | https://www.genecards.org/cgi-bin/carddisp.pl?gene=IL15 |
| IL6R | 1.787955761 | https://www.genecards.org/cgi-bin/carddisp.pl?gene=IL6R |
| HRG | 1.786509991 | https://www.genecards.org/cgi-bin/carddisp.pl?gene=HRG |
| WIPF1 | 1.785288453 | https://www.genecards.org/cgi-bin/carddisp.pl?gene=WIPF1 |
| MB | 1.780776262 | https://www.genecards.org/cgi-bin/carddisp.pl?gene=MB |
| TRC-GCA24-1 | 1.780482173 | https://www.genecards.org/cgi-bin/carddisp.pl?gene=TRC-GCA24-1 |
| EGF | 1.778748512 | https://www.genecards.org/cgi-bin/carddisp.pl?gene=EGF |
| CD69 | 1.772593856 | https://www.genecards.org/cgi-bin/carddisp.pl?gene=CD69 |
| CASC9 | 1.772593856 | https://www.genecards.org/cgi-bin/carddisp.pl?gene=CASC9 |
| BTLA | 1.764477253 | https://www.genecards.org/cgi-bin/carddisp.pl?gene=BTLA |
| RIGI | 1.761576891 | https://www.genecards.org/cgi-bin/carddisp.pl?gene=RIGI |
| TRAF1 | 1.760942698 | https://www.genecards.org/cgi-bin/carddisp.pl?gene=TRAF1 |
| IL21 | 1.757561326 | https://www.genecards.org/cgi-bin/carddisp.pl?gene=IL21 |
| CD80 | 1.757561326 | https://www.genecards.org/cgi-bin/carddisp.pl?gene=CD80 |
| MIR378A | 1.757320285 | https://www.genecards.org/cgi-bin/carddisp.pl?gene=MIR378A |
| MIR7-3HG | 1.756517529 | https://www.genecards.org/cgi-bin/carddisp.pl?gene=MIR7-3HG |
| CPT1A | 1.753736019 | https://www.genecards.org/cgi-bin/carddisp.pl?gene=CPT1A |
| BMAL1 | 1.750391841 | https://www.genecards.org/cgi-bin/carddisp.pl?gene=BMAL1 |
| PROZ | 1.748085737 | https://www.genecards.org/cgi-bin/carddisp.pl?gene=PROZ |
| DUSP1 | 1.747929573 | https://www.genecards.org/cgi-bin/carddisp.pl?gene=DUSP1 |
| RPS27A | 1.747929573 | https://www.genecards.org/cgi-bin/carddisp.pl?gene=RPS27A |
| CHRNA7 | 1.746879816 | https://www.genecards.org/cgi-bin/carddisp.pl?gene=CHRNA7 |
| TOLLIP | 1.743676186 | https://www.genecards.org/cgi-bin/carddisp.pl?gene=TOLLIP |
| GDF15 | 1.743676186 | https://www.genecards.org/cgi-bin/carddisp.pl?gene=GDF15 |
| XK | 1.74225831 | https://www.genecards.org/cgi-bin/carddisp.pl?gene=XK |
| SFTPC | 1.741745234 | https://www.genecards.org/cgi-bin/carddisp.pl?gene=SFTPC |
| MIR483 | 1.737934589 | https://www.genecards.org/cgi-bin/carddisp.pl?gene=MIR483 |
| PARP1 | 1.730779409 | https://www.genecards.org/cgi-bin/carddisp.pl?gene=PARP1 |
| GP6 | 1.728614092 | https://www.genecards.org/cgi-bin/carddisp.pl?gene=GP6 |
| SUCLG2 | 1.723972559 | https://www.genecards.org/cgi-bin/carddisp.pl?gene=SUCLG2 |
| ACR | 1.723972559 | https://www.genecards.org/cgi-bin/carddisp.pl?gene=ACR |
| MORN2 | 1.723972559 | https://www.genecards.org/cgi-bin/carddisp.pl?gene=MORN2 |
| MAP2K3 | 1.718485951 | https://www.genecards.org/cgi-bin/carddisp.pl?gene=MAP2K3 |
| ANO1 | 1.718485951 | https://www.genecards.org/cgi-bin/carddisp.pl?gene=ANO1 |
| FUT2 | 1.718451142 | https://www.genecards.org/cgi-bin/carddisp.pl?gene=FUT2 |
| CCL11 | 1.718374491 | https://www.genecards.org/cgi-bin/carddisp.pl?gene=CCL11 |
| UGT1A1 | 1.713102102 | https://www.genecards.org/cgi-bin/carddisp.pl?gene=UGT1A1 |
| CISH | 1.713102102 | https://www.genecards.org/cgi-bin/carddisp.pl?gene=CISH |
| NPPA | 1.713102102 | https://www.genecards.org/cgi-bin/carddisp.pl?gene=NPPA |
| FGL2 | 1.713102102 | https://www.genecards.org/cgi-bin/carddisp.pl?gene=FGL2 |
| FGB | 1.712955952 | https://www.genecards.org/cgi-bin/carddisp.pl?gene=FGB |
| SLC2A2 | 1.711848974 | https://www.genecards.org/cgi-bin/carddisp.pl?gene=SLC2A2 |
| AKR1B1 | 1.711848974 | https://www.genecards.org/cgi-bin/carddisp.pl?gene=AKR1B1 |
| SERPINA1 | 1.70745945 | https://www.genecards.org/cgi-bin/carddisp.pl?gene=SERPINA1 |
| IFNAR1 | 1.706816196 | https://www.genecards.org/cgi-bin/carddisp.pl?gene=IFNAR1 |
| CXCL9 | 1.702877045 | https://www.genecards.org/cgi-bin/carddisp.pl?gene=CXCL9 |
| DKC1 | 1.698038101 | https://www.genecards.org/cgi-bin/carddisp.pl?gene=DKC1 |
| ACP1 | 1.698038101 | https://www.genecards.org/cgi-bin/carddisp.pl?gene=ACP1 |
| FOXO3 | 1.697097182 | https://www.genecards.org/cgi-bin/carddisp.pl?gene=FOXO3 |
| SIRT3 | 1.697097182 | https://www.genecards.org/cgi-bin/carddisp.pl?gene=SIRT3 |
| PTK2B | 1.694372892 | https://www.genecards.org/cgi-bin/carddisp.pl?gene=PTK2B |
| TSPOAP1-AS1 | 1.692668676 | https://www.genecards.org/cgi-bin/carddisp.pl?gene=TSPOAP1-AS1 |
| CYP1A2 | 1.680549383 | https://www.genecards.org/cgi-bin/carddisp.pl?gene=CYP1A2 |
| EMSLR | 1.679738164 | https://www.genecards.org/cgi-bin/carddisp.pl?gene=EMSLR |
| TMPRSS2 | 1.676432014 | https://www.genecards.org/cgi-bin/carddisp.pl?gene=TMPRSS2 |
| OTC | 1.676432014 | https://www.genecards.org/cgi-bin/carddisp.pl?gene=OTC |
| C4B | 1.676432014 | https://www.genecards.org/cgi-bin/carddisp.pl?gene=C4B |
| PDGFB | 1.6764189 | https://www.genecards.org/cgi-bin/carddisp.pl?gene=PDGFB |
| NRG1 | 1.6764189 | https://www.genecards.org/cgi-bin/carddisp.pl?gene=NRG1 |
| ADAR | 1.6764189 | https://www.genecards.org/cgi-bin/carddisp.pl?gene=ADAR |
| GFAP | 1.676192999 | https://www.genecards.org/cgi-bin/carddisp.pl?gene=GFAP |
| FGF23 | 1.676192999 | https://www.genecards.org/cgi-bin/carddisp.pl?gene=FGF23 |
| SLIT2 | 1.676192999 | https://www.genecards.org/cgi-bin/carddisp.pl?gene=SLIT2 |
| GZMA | 1.676192999 | https://www.genecards.org/cgi-bin/carddisp.pl?gene=GZMA |
| CRYAA | 1.676165104 | https://www.genecards.org/cgi-bin/carddisp.pl?gene=CRYAA |
| AK2 | 1.668064117 | https://www.genecards.org/cgi-bin/carddisp.pl?gene=AK2 |
| ALG1 | 1.668064117 | https://www.genecards.org/cgi-bin/carddisp.pl?gene=ALG1 |
| PGM3 | 1.668064117 | https://www.genecards.org/cgi-bin/carddisp.pl?gene=PGM3 |
| PKP1 | 1.668064117 | https://www.genecards.org/cgi-bin/carddisp.pl?gene=PKP1 |
| MIR181D | 1.664682865 | https://www.genecards.org/cgi-bin/carddisp.pl?gene=MIR181D |
| SMPD1 | 1.66339922 | https://www.genecards.org/cgi-bin/carddisp.pl?gene=SMPD1 |
| MMP10 | 1.66339922 | https://www.genecards.org/cgi-bin/carddisp.pl?gene=MMP10 |
| SLC2A1 | 1.65340066 | https://www.genecards.org/cgi-bin/carddisp.pl?gene=SLC2A1 |
| HSPD1 | 1.65340066 | https://www.genecards.org/cgi-bin/carddisp.pl?gene=HSPD1 |
| HMOX2 | 1.65340066 | https://www.genecards.org/cgi-bin/carddisp.pl?gene=HMOX2 |
| MDM2 | 1.650742292 | https://www.genecards.org/cgi-bin/carddisp.pl?gene=MDM2 |
| FOXO1 | 1.648688793 | https://www.genecards.org/cgi-bin/carddisp.pl?gene=FOXO1 |
| UCA1 | 1.648688793 | https://www.genecards.org/cgi-bin/carddisp.pl?gene=UCA1 |
| FGF2 | 1.645575762 | https://www.genecards.org/cgi-bin/carddisp.pl?gene=FGF2 |
| CXCR1 | 1.645575762 | https://www.genecards.org/cgi-bin/carddisp.pl?gene=CXCR1 |
| DSG1 | 1.637492657 | https://www.genecards.org/cgi-bin/carddisp.pl?gene=DSG1 |
| KLK7 | 1.637492657 | https://www.genecards.org/cgi-bin/carddisp.pl?gene=KLK7 |
| FAH | 1.636379957 | https://www.genecards.org/cgi-bin/carddisp.pl?gene=FAH |
| GBA1 | 1.635831952 | https://www.genecards.org/cgi-bin/carddisp.pl?gene=GBA1 |
| MTOR | 1.633176804 | https://www.genecards.org/cgi-bin/carddisp.pl?gene=MTOR |
| TREM2 | 1.633176804 | https://www.genecards.org/cgi-bin/carddisp.pl?gene=TREM2 |
| MIR508 | 1.633176804 | https://www.genecards.org/cgi-bin/carddisp.pl?gene=MIR508 |
| CYLD-AS1 | 1.633176804 | https://www.genecards.org/cgi-bin/carddisp.pl?gene=CYLD-AS1 |
| MIR1246 | 1.629295468 | https://www.genecards.org/cgi-bin/carddisp.pl?gene=MIR1246 |
| NPY | 1.628670573 | https://www.genecards.org/cgi-bin/carddisp.pl?gene=NPY |
| TLX1NB | 1.628670573 | https://www.genecards.org/cgi-bin/carddisp.pl?gene=TLX1NB |
| HGF | 1.626992822 | https://www.genecards.org/cgi-bin/carddisp.pl?gene=HGF |
| SERPING1 | 1.626992822 | https://www.genecards.org/cgi-bin/carddisp.pl?gene=SERPING1 |
| MIR17 | 1.626992822 | https://www.genecards.org/cgi-bin/carddisp.pl?gene=MIR17 |
| SMAD3 | 1.624094248 | https://www.genecards.org/cgi-bin/carddisp.pl?gene=SMAD3 |
| CD36 | 1.617172003 | https://www.genecards.org/cgi-bin/carddisp.pl?gene=CD36 |
| GSN | 1.617172003 | https://www.genecards.org/cgi-bin/carddisp.pl?gene=GSN |
| HSPA5 | 1.617172003 | https://www.genecards.org/cgi-bin/carddisp.pl?gene=HSPA5 |
| PINK1 | 1.617172003 | https://www.genecards.org/cgi-bin/carddisp.pl?gene=PINK1 |
| IGF2-AS | 1.617172003 | https://www.genecards.org/cgi-bin/carddisp.pl?gene=IGF2-AS |
| MIR199B | 1.617172003 | https://www.genecards.org/cgi-bin/carddisp.pl?gene=MIR199B |
| GUSB | 1.616134644 | https://www.genecards.org/cgi-bin/carddisp.pl?gene=GUSB |
| SULT2B1 | 1.615739465 | https://www.genecards.org/cgi-bin/carddisp.pl?gene=SULT2B1 |
| LIPN | 1.615739465 | https://www.genecards.org/cgi-bin/carddisp.pl?gene=LIPN |
| SDR9C7 | 1.615739465 | https://www.genecards.org/cgi-bin/carddisp.pl?gene=SDR9C7 |
| APOM | 1.607544184 | https://www.genecards.org/cgi-bin/carddisp.pl?gene=APOM |
| CYP3A5 | 1.601778269 | https://www.genecards.org/cgi-bin/carddisp.pl?gene=CYP3A5 |
| PNPLA2 | 1.601778269 | https://www.genecards.org/cgi-bin/carddisp.pl?gene=PNPLA2 |
| TNFRSF18 | 1.601778269 | https://www.genecards.org/cgi-bin/carddisp.pl?gene=TNFRSF18 |
| SPN | 1.601778269 | https://www.genecards.org/cgi-bin/carddisp.pl?gene=SPN |
| MIR615 | 1.601778269 | https://www.genecards.org/cgi-bin/carddisp.pl?gene=MIR615 |
| GJA1 | 1.600624084 | https://www.genecards.org/cgi-bin/carddisp.pl?gene=GJA1 |
| CYCS | 1.600624084 | https://www.genecards.org/cgi-bin/carddisp.pl?gene=CYCS |
| APLN | 1.600624084 | https://www.genecards.org/cgi-bin/carddisp.pl?gene=APLN |
| IFNL2 | 1.600624084 | https://www.genecards.org/cgi-bin/carddisp.pl?gene=IFNL2 |
| MIR22 | 1.600624084 | https://www.genecards.org/cgi-bin/carddisp.pl?gene=MIR22 |
| GSEC | 1.600624084 | https://www.genecards.org/cgi-bin/carddisp.pl?gene=GSEC |
| CD27 | 1.600180507 | https://www.genecards.org/cgi-bin/carddisp.pl?gene=CD27 |
| TRE-TTC3-1 | 1.597304583 | https://www.genecards.org/cgi-bin/carddisp.pl?gene=TRE-TTC3-1 |
| MIR7-1 | 1.58850801 | https://www.genecards.org/cgi-bin/carddisp.pl?gene=MIR7-1 |
| GRK2 | 1.587096334 | https://www.genecards.org/cgi-bin/carddisp.pl?gene=GRK2 |
| ADAMTSL1 | 1.587096334 | https://www.genecards.org/cgi-bin/carddisp.pl?gene=ADAMTSL1 |
| ACADVL | 1.586121917 | https://www.genecards.org/cgi-bin/carddisp.pl?gene=ACADVL |
| CALCRL | 1.583473921 | https://www.genecards.org/cgi-bin/carddisp.pl?gene=CALCRL |
| APOL1 | 1.583473921 | https://www.genecards.org/cgi-bin/carddisp.pl?gene=APOL1 |
| CX3CR1 | 1.583473921 | https://www.genecards.org/cgi-bin/carddisp.pl?gene=CX3CR1 |
| RAMP2 | 1.583473921 | https://www.genecards.org/cgi-bin/carddisp.pl?gene=RAMP2 |
| IGHE | 1.57947576 | https://www.genecards.org/cgi-bin/carddisp.pl?gene=IGHE |
| FLG | 1.577340484 | https://www.genecards.org/cgi-bin/carddisp.pl?gene=FLG |
| SLPI | 1.576345921 | https://www.genecards.org/cgi-bin/carddisp.pl?gene=SLPI |
| IRGM | 1.576345921 | https://www.genecards.org/cgi-bin/carddisp.pl?gene=IRGM |
| TPMT | 1.575698137 | https://www.genecards.org/cgi-bin/carddisp.pl?gene=TPMT |
| S1PR1 | 1.573537469 | https://www.genecards.org/cgi-bin/carddisp.pl?gene=S1PR1 |
| JAK1 | 1.572032332 | https://www.genecards.org/cgi-bin/carddisp.pl?gene=JAK1 |
| CTSB | 1.572032332 | https://www.genecards.org/cgi-bin/carddisp.pl?gene=CTSB |
| TSLP | 1.572032332 | https://www.genecards.org/cgi-bin/carddisp.pl?gene=TSLP |
| GALK2 | 1.570894241 | https://www.genecards.org/cgi-bin/carddisp.pl?gene=GALK2 |
| AIRE | 1.567057014 | https://www.genecards.org/cgi-bin/carddisp.pl?gene=AIRE |
| CAV1 | 1.565650463 | https://www.genecards.org/cgi-bin/carddisp.pl?gene=CAV1 |
| CD44 | 1.565650463 | https://www.genecards.org/cgi-bin/carddisp.pl?gene=CD44 |
| MTHFR | 1.565650463 | https://www.genecards.org/cgi-bin/carddisp.pl?gene=MTHFR |
| DEFA3 | 1.565650463 | https://www.genecards.org/cgi-bin/carddisp.pl?gene=DEFA3 |
| ACSM6 | 1.565650463 | https://www.genecards.org/cgi-bin/carddisp.pl?gene=ACSM6 |
| RAB4B-EGLN2 | 1.565650463 | https://www.genecards.org/cgi-bin/carddisp.pl?gene=RAB4B-EGLN2 |
| ENSG00000269966 | 1.565650463 | https://www.genecards.org/cgi-bin/carddisp.pl?gene=ENSG00000269966 |
| HPSE2 | 1.562699556 | https://www.genecards.org/cgi-bin/carddisp.pl?gene=HPSE2 |
| SPP1 | 1.55959022 | https://www.genecards.org/cgi-bin/carddisp.pl?gene=SPP1 |
| PMM2 | 1.550426245 | https://www.genecards.org/cgi-bin/carddisp.pl?gene=PMM2 |
| POLG | 1.550426245 | https://www.genecards.org/cgi-bin/carddisp.pl?gene=POLG |
| ALG2 | 1.550426245 | https://www.genecards.org/cgi-bin/carddisp.pl?gene=ALG2 |
| CD244 | 1.547067642 | https://www.genecards.org/cgi-bin/carddisp.pl?gene=CD244 |
| HLA-G | 1.547067642 | https://www.genecards.org/cgi-bin/carddisp.pl?gene=HLA-G |
| SELENOP | 1.547067642 | https://www.genecards.org/cgi-bin/carddisp.pl?gene=SELENOP |
| MIR93 | 1.547067642 | https://www.genecards.org/cgi-bin/carddisp.pl?gene=MIR93 |
| MIR193A | 1.547067642 | https://www.genecards.org/cgi-bin/carddisp.pl?gene=MIR193A |
| CBS | 1.544528127 | https://www.genecards.org/cgi-bin/carddisp.pl?gene=CBS |
| TNFRSF11B | 1.544528127 | https://www.genecards.org/cgi-bin/carddisp.pl?gene=TNFRSF11B |
| DPP4 | 1.542459011 | https://www.genecards.org/cgi-bin/carddisp.pl?gene=DPP4 |
| TAFAZZIN | 1.540281177 | https://www.genecards.org/cgi-bin/carddisp.pl?gene=TAFAZZIN |
| CTSL | 1.538269162 | https://www.genecards.org/cgi-bin/carddisp.pl?gene=CTSL |
| PADI4 | 1.538269162 | https://www.genecards.org/cgi-bin/carddisp.pl?gene=PADI4 |
| RAP1A | 1.538269162 | https://www.genecards.org/cgi-bin/carddisp.pl?gene=RAP1A |
| LCP2 | 1.538269162 | https://www.genecards.org/cgi-bin/carddisp.pl?gene=LCP2 |
| RBMS1 | 1.538269162 | https://www.genecards.org/cgi-bin/carddisp.pl?gene=RBMS1 |
| OLFM4 | 1.531764507 | https://www.genecards.org/cgi-bin/carddisp.pl?gene=OLFM4 |
| MIR142 | 1.531764507 | https://www.genecards.org/cgi-bin/carddisp.pl?gene=MIR142 |
| DOCK8 | 1.529637098 | https://www.genecards.org/cgi-bin/carddisp.pl?gene=DOCK8 |
| ATG7 | 1.527619004 | https://www.genecards.org/cgi-bin/carddisp.pl?gene=ATG7 |
| HSPA1L | 1.527619004 | https://www.genecards.org/cgi-bin/carddisp.pl?gene=HSPA1L |
| IL1RL1 | 1.527619004 | https://www.genecards.org/cgi-bin/carddisp.pl?gene=IL1RL1 |
| MIR128-1 | 1.527619004 | https://www.genecards.org/cgi-bin/carddisp.pl?gene=MIR128-1 |
| MIR642A | 1.527619004 | https://www.genecards.org/cgi-bin/carddisp.pl?gene=MIR642A |
| IL12RB1 | 1.522326946 | https://www.genecards.org/cgi-bin/carddisp.pl?gene=IL12RB1 |
| TICAM1 | 1.521368623 | https://www.genecards.org/cgi-bin/carddisp.pl?gene=TICAM1 |
| ITGA2B | 1.51970768 | https://www.genecards.org/cgi-bin/carddisp.pl?gene=ITGA2B |
| TTC7A | 1.51716876 | https://www.genecards.org/cgi-bin/carddisp.pl?gene=TTC7A |
| LIG4 | 1.515294433 | https://www.genecards.org/cgi-bin/carddisp.pl?gene=LIG4 |
| PNPLA1 | 1.513998985 | https://www.genecards.org/cgi-bin/carddisp.pl?gene=PNPLA1 |
| ATF3 | 1.513011336 | https://www.genecards.org/cgi-bin/carddisp.pl?gene=ATF3 |
| IFIH1 | 1.512440801 | https://www.genecards.org/cgi-bin/carddisp.pl?gene=IFIH1 |
| PTEN | 1.507171035 | https://www.genecards.org/cgi-bin/carddisp.pl?gene=PTEN |
| PRKCA | 1.507171035 | https://www.genecards.org/cgi-bin/carddisp.pl?gene=PRKCA |
| KEAP1 | 1.507171035 | https://www.genecards.org/cgi-bin/carddisp.pl?gene=KEAP1 |
| PKM | 1.507171035 | https://www.genecards.org/cgi-bin/carddisp.pl?gene=PKM |
| HAMP | 1.507171035 | https://www.genecards.org/cgi-bin/carddisp.pl?gene=HAMP |
| BCL2A1 | 1.507171035 | https://www.genecards.org/cgi-bin/carddisp.pl?gene=BCL2A1 |
| GPX3 | 1.507171035 | https://www.genecards.org/cgi-bin/carddisp.pl?gene=GPX3 |
| MIR497 | 1.507171035 | https://www.genecards.org/cgi-bin/carddisp.pl?gene=MIR497 |
| MIR376B | 1.507171035 | https://www.genecards.org/cgi-bin/carddisp.pl?gene=MIR376B |
| ATG16L1 | 1.494256973 | https://www.genecards.org/cgi-bin/carddisp.pl?gene=ATG16L1 |
| MIR27A | 1.490836501 | https://www.genecards.org/cgi-bin/carddisp.pl?gene=MIR27A |
| CLDN2 | 1.48594451 | https://www.genecards.org/cgi-bin/carddisp.pl?gene=CLDN2 |
| SCGB1A1 | 1.48594451 | https://www.genecards.org/cgi-bin/carddisp.pl?gene=SCGB1A1 |
| APOB | 1.485551834 | https://www.genecards.org/cgi-bin/carddisp.pl?gene=APOB |
| IL32 | 1.485551834 | https://www.genecards.org/cgi-bin/carddisp.pl?gene=IL32 |
| MIR370 | 1.485551834 | https://www.genecards.org/cgi-bin/carddisp.pl?gene=MIR370 |
| SNHG1 | 1.485551834 | https://www.genecards.org/cgi-bin/carddisp.pl?gene=SNHG1 |
| FERMT3 | 1.471192837 | https://www.genecards.org/cgi-bin/carddisp.pl?gene=FERMT3 |
| LEPQTL1 | 1.467812061 | https://www.genecards.org/cgi-bin/carddisp.pl?gene=LEPQTL1 |
| PI3 | 1.463364959 | https://www.genecards.org/cgi-bin/carddisp.pl?gene=PI3 |
| MCL1 | 1.462533712 | https://www.genecards.org/cgi-bin/carddisp.pl?gene=MCL1 |
| MIR96 | 1.462533712 | https://www.genecards.org/cgi-bin/carddisp.pl?gene=MIR96 |
| RNU6-1 | 1.462533712 | https://www.genecards.org/cgi-bin/carddisp.pl?gene=RNU6-1 |
| RNU6-2 | 1.462533712 | https://www.genecards.org/cgi-bin/carddisp.pl?gene=RNU6-2 |
| RNU6-7 | 1.462533712 | https://www.genecards.org/cgi-bin/carddisp.pl?gene=RNU6-7 |
| RNU6-8 | 1.462533712 | https://www.genecards.org/cgi-bin/carddisp.pl?gene=RNU6-8 |
| RNU6-9 | 1.462533712 | https://www.genecards.org/cgi-bin/carddisp.pl?gene=RNU6-9 |
| RNU6-1-001 | 1.462533712 | https://www.genecards.org/cgi-bin/carddisp.pl?gene=RNU6-1-001 |
| RNU6-1-002 | 1.462533712 | https://www.genecards.org/cgi-bin/carddisp.pl?gene=RNU6-1-002 |
| RNU6-1-003 | 1.462533712 | https://www.genecards.org/cgi-bin/carddisp.pl?gene=RNU6-1-003 |
| RNU6-1-004 | 1.462533712 | https://www.genecards.org/cgi-bin/carddisp.pl?gene=RNU6-1-004 |
| TMPRSS15 | 1.461489916 | https://www.genecards.org/cgi-bin/carddisp.pl?gene=TMPRSS15 |
| SESN2 | 1.461489916 | https://www.genecards.org/cgi-bin/carddisp.pl?gene=SESN2 |
| MT-CO1 | 1.461090565 | https://www.genecards.org/cgi-bin/carddisp.pl?gene=MT-CO1 |
| LYN | 1.46056664 | https://www.genecards.org/cgi-bin/carddisp.pl?gene=LYN |
| UGDH | 1.460116148 | https://www.genecards.org/cgi-bin/carddisp.pl?gene=UGDH |
| UAP1 | 1.460116148 | https://www.genecards.org/cgi-bin/carddisp.pl?gene=UAP1 |
| UGP2 | 1.460116148 | https://www.genecards.org/cgi-bin/carddisp.pl?gene=UGP2 |
| IGFBP1 | 1.453988552 | https://www.genecards.org/cgi-bin/carddisp.pl?gene=IGFBP1 |
| SERPINA4 | 1.453988552 | https://www.genecards.org/cgi-bin/carddisp.pl?gene=SERPINA4 |
| MIRLET7C | 1.453988552 | https://www.genecards.org/cgi-bin/carddisp.pl?gene=MIRLET7C |
| KLK5 | 1.451601624 | https://www.genecards.org/cgi-bin/carddisp.pl?gene=KLK5 |
| SAA1 | 1.448363423 | https://www.genecards.org/cgi-bin/carddisp.pl?gene=SAA1 |
| CASC15 | 1.446026564 | https://www.genecards.org/cgi-bin/carddisp.pl?gene=CASC15 |
| PPP3CA | 1.445895195 | https://www.genecards.org/cgi-bin/carddisp.pl?gene=PPP3CA |
| NKX2-6 | 1.437803626 | https://www.genecards.org/cgi-bin/carddisp.pl?gene=NKX2-6 |
| STARD3NL | 1.437803626 | https://www.genecards.org/cgi-bin/carddisp.pl?gene=STARD3NL |
| ADM2 | 1.437803626 | https://www.genecards.org/cgi-bin/carddisp.pl?gene=ADM2 |
| KRT18P32 | 1.437803626 | https://www.genecards.org/cgi-bin/carddisp.pl?gene=KRT18P32 |
| IL37 | 1.437190056 | https://www.genecards.org/cgi-bin/carddisp.pl?gene=IL37 |
| MIR218-1 | 1.437190056 | https://www.genecards.org/cgi-bin/carddisp.pl?gene=MIR218-1 |
| FBXO32 | 1.436838388 | https://www.genecards.org/cgi-bin/carddisp.pl?gene=FBXO32 |
| AIM2 | 1.436693788 | https://www.genecards.org/cgi-bin/carddisp.pl?gene=AIM2 |
| ATP7A | 1.424893737 | https://www.genecards.org/cgi-bin/carddisp.pl?gene=ATP7A |
| HYOU1 | 1.423546076 | https://www.genecards.org/cgi-bin/carddisp.pl?gene=HYOU1 |
| GLS | 1.423458338 | https://www.genecards.org/cgi-bin/carddisp.pl?gene=GLS |
| RIPK3 | 1.423458338 | https://www.genecards.org/cgi-bin/carddisp.pl?gene=RIPK3 |
| MIR381 | 1.423458338 | https://www.genecards.org/cgi-bin/carddisp.pl?gene=MIR381 |
| PLCG2 | 1.420517325 | https://www.genecards.org/cgi-bin/carddisp.pl?gene=PLCG2 |
| TNFSF13B | 1.419262528 | https://www.genecards.org/cgi-bin/carddisp.pl?gene=TNFSF13B |
| IL12A | 1.419014931 | https://www.genecards.org/cgi-bin/carddisp.pl?gene=IL12A |
| PWAR4 | 1.419014931 | https://www.genecards.org/cgi-bin/carddisp.pl?gene=PWAR4 |
| GATA2 | 1.40689528 | https://www.genecards.org/cgi-bin/carddisp.pl?gene=GATA2 |
| MYC | 1.406615973 | https://www.genecards.org/cgi-bin/carddisp.pl?gene=MYC |
| MIR214 | 1.406296372 | https://www.genecards.org/cgi-bin/carddisp.pl?gene=MIR214 |
| DNAJC19 | 1.401276827 | https://www.genecards.org/cgi-bin/carddisp.pl?gene=DNAJC19 |
| GGT1 | 1.400431991 | https://www.genecards.org/cgi-bin/carddisp.pl?gene=GGT1 |
| ELAVL1 | 1.400431991 | https://www.genecards.org/cgi-bin/carddisp.pl?gene=ELAVL1 |
| MIR221 | 1.390611053 | https://www.genecards.org/cgi-bin/carddisp.pl?gene=MIR221 |
| MMP3 | 1.386663795 | https://www.genecards.org/cgi-bin/carddisp.pl?gene=MMP3 |
| KRT4 | 1.385100365 | https://www.genecards.org/cgi-bin/carddisp.pl?gene=KRT4 |
| AK8 | 1.384732485 | https://www.genecards.org/cgi-bin/carddisp.pl?gene=AK8 |
| MED27 | 1.384732485 | https://www.genecards.org/cgi-bin/carddisp.pl?gene=MED27 |
| ARPP21 | 1.384732485 | https://www.genecards.org/cgi-bin/carddisp.pl?gene=ARPP21 |
| PDE7A | 1.381391287 | https://www.genecards.org/cgi-bin/carddisp.pl?gene=PDE7A |
| CYP1A1 | 1.381165266 | https://www.genecards.org/cgi-bin/carddisp.pl?gene=CYP1A1 |
| IGFBP7 | 1.381165266 | https://www.genecards.org/cgi-bin/carddisp.pl?gene=IGFBP7 |
| MMP16 | 1.381165266 | https://www.genecards.org/cgi-bin/carddisp.pl?gene=MMP16 |
| FABP3 | 1.381165266 | https://www.genecards.org/cgi-bin/carddisp.pl?gene=FABP3 |
| PICK1 | 1.381165266 | https://www.genecards.org/cgi-bin/carddisp.pl?gene=PICK1 |
| SERPINA7 | 1.381165266 | https://www.genecards.org/cgi-bin/carddisp.pl?gene=SERPINA7 |
| MIR181A1 | 1.381165266 | https://www.genecards.org/cgi-bin/carddisp.pl?gene=MIR181A1 |
| CDKN3 | 1.380983353 | https://www.genecards.org/cgi-bin/carddisp.pl?gene=CDKN3 |
| LINC01629 | 1.380983353 | https://www.genecards.org/cgi-bin/carddisp.pl?gene=LINC01629 |
| ADIPOQ | 1.374063253 | https://www.genecards.org/cgi-bin/carddisp.pl?gene=ADIPOQ |
| IL17F | 1.374063253 | https://www.genecards.org/cgi-bin/carddisp.pl?gene=IL17F |
| NORAD | 1.374063253 | https://www.genecards.org/cgi-bin/carddisp.pl?gene=NORAD |
| ERVW-1 | 1.372681618 | https://www.genecards.org/cgi-bin/carddisp.pl?gene=ERVW-1 |
| LAMC2 | 1.364723921 | https://www.genecards.org/cgi-bin/carddisp.pl?gene=LAMC2 |
| TNFRSF6B | 1.360535383 | https://www.genecards.org/cgi-bin/carddisp.pl?gene=TNFRSF6B |
| MIR423 | 1.35691309 | https://www.genecards.org/cgi-bin/carddisp.pl?gene=MIR423 |
| MIR4270 | 1.35691309 | https://www.genecards.org/cgi-bin/carddisp.pl?gene=MIR4270 |
| HCCAT5 | 1.350958109 | https://www.genecards.org/cgi-bin/carddisp.pl?gene=HCCAT5 |
| EGR1 | 1.347678542 | https://www.genecards.org/cgi-bin/carddisp.pl?gene=EGR1 |
| FBXW7 | 1.347402215 | https://www.genecards.org/cgi-bin/carddisp.pl?gene=FBXW7 |
| LST1 | 1.347402215 | https://www.genecards.org/cgi-bin/carddisp.pl?gene=LST1 |
| COG4 | 1.342943311 | https://www.genecards.org/cgi-bin/carddisp.pl?gene=COG4 |
| HSPA8 | 1.339089632 | https://www.genecards.org/cgi-bin/carddisp.pl?gene=HSPA8 |
| LGALS3 | 1.339089632 | https://www.genecards.org/cgi-bin/carddisp.pl?gene=LGALS3 |
| PAH | 1.334377289 | https://www.genecards.org/cgi-bin/carddisp.pl?gene=PAH |
| PGM1 | 1.334377289 | https://www.genecards.org/cgi-bin/carddisp.pl?gene=PGM1 |
| HADH | 1.334377289 | https://www.genecards.org/cgi-bin/carddisp.pl?gene=HADH |
| ACADM | 1.334377289 | https://www.genecards.org/cgi-bin/carddisp.pl?gene=ACADM |
| BTD | 1.334377289 | https://www.genecards.org/cgi-bin/carddisp.pl?gene=BTD |
| SLC25A13 | 1.334377289 | https://www.genecards.org/cgi-bin/carddisp.pl?gene=SLC25A13 |
| ACADS | 1.334377289 | https://www.genecards.org/cgi-bin/carddisp.pl?gene=ACADS |
| LCT | 1.334377289 | https://www.genecards.org/cgi-bin/carddisp.pl?gene=LCT |
| BMP15 | 1.334377289 | https://www.genecards.org/cgi-bin/carddisp.pl?gene=BMP15 |
| GDF9 | 1.334377289 | https://www.genecards.org/cgi-bin/carddisp.pl?gene=GDF9 |
| LGALS7 | 1.334377289 | https://www.genecards.org/cgi-bin/carddisp.pl?gene=LGALS7 |
| SOHLH1 | 1.334377289 | https://www.genecards.org/cgi-bin/carddisp.pl?gene=SOHLH1 |
| LGALS7B | 1.334377289 | https://www.genecards.org/cgi-bin/carddisp.pl?gene=LGALS7B |
| LAMB3 | 1.324674606 | https://www.genecards.org/cgi-bin/carddisp.pl?gene=LAMB3 |
| HSPB1 | 1.320506692 | https://www.genecards.org/cgi-bin/carddisp.pl?gene=HSPB1 |
| PAFAH1B1 | 1.320506692 | https://www.genecards.org/cgi-bin/carddisp.pl?gene=PAFAH1B1 |
| HPSE | 1.320506692 | https://www.genecards.org/cgi-bin/carddisp.pl?gene=HPSE |
| MIR124-1 | 1.315898061 | https://www.genecards.org/cgi-bin/carddisp.pl?gene=MIR124-1 |
| MIR15A | 1.315898061 | https://www.genecards.org/cgi-bin/carddisp.pl?gene=MIR15A |
| TYMP | 1.311233521 | https://www.genecards.org/cgi-bin/carddisp.pl?gene=TYMP |
| CSN1S1 | 1.311233521 | https://www.genecards.org/cgi-bin/carddisp.pl?gene=CSN1S1 |
| NOTCH4 | 1.307352901 | https://www.genecards.org/cgi-bin/carddisp.pl?gene=NOTCH4 |
| TNFSF14 | 1.307352901 | https://www.genecards.org/cgi-bin/carddisp.pl?gene=TNFSF14 |
| BAG6 | 1.307352901 | https://www.genecards.org/cgi-bin/carddisp.pl?gene=BAG6 |
| CCHCR1 | 1.307352901 | https://www.genecards.org/cgi-bin/carddisp.pl?gene=CCHCR1 |
| HDAC6 | 1.302455306 | https://www.genecards.org/cgi-bin/carddisp.pl?gene=HDAC6 |
| KLK4 | 1.302452087 | https://www.genecards.org/cgi-bin/carddisp.pl?gene=KLK4 |
| KLK6 | 1.302452087 | https://www.genecards.org/cgi-bin/carddisp.pl?gene=KLK6 |
| TGM3 | 1.302452087 | https://www.genecards.org/cgi-bin/carddisp.pl?gene=TGM3 |
| CDSN | 1.302452087 | https://www.genecards.org/cgi-bin/carddisp.pl?gene=CDSN |
| KLK13 | 1.302452087 | https://www.genecards.org/cgi-bin/carddisp.pl?gene=KLK13 |
| KLK12 | 1.302452087 | https://www.genecards.org/cgi-bin/carddisp.pl?gene=KLK12 |
| KLK14 | 1.302452087 | https://www.genecards.org/cgi-bin/carddisp.pl?gene=KLK14 |
| ACLY | 1.301058054 | https://www.genecards.org/cgi-bin/carddisp.pl?gene=ACLY |
| DDIT4 | 1.301058054 | https://www.genecards.org/cgi-bin/carddisp.pl?gene=DDIT4 |
| PNOC | 1.301058054 | https://www.genecards.org/cgi-bin/carddisp.pl?gene=PNOC |
| SULT1A3 | 1.301058054 | https://www.genecards.org/cgi-bin/carddisp.pl?gene=SULT1A3 |
| MIR34A | 1.301058054 | https://www.genecards.org/cgi-bin/carddisp.pl?gene=MIR34A |
| MT-TP | 1.301058054 | https://www.genecards.org/cgi-bin/carddisp.pl?gene=MT-TP |
| CD19 | 1.293967605 | https://www.genecards.org/cgi-bin/carddisp.pl?gene=CD19 |
| ITK | 1.293967605 | https://www.genecards.org/cgi-bin/carddisp.pl?gene=ITK |
| PLEK | 1.293967605 | https://www.genecards.org/cgi-bin/carddisp.pl?gene=PLEK |
| STK4 | 1.29325676 | https://www.genecards.org/cgi-bin/carddisp.pl?gene=STK4 |
| LINC01554 | 1.286803126 | https://www.genecards.org/cgi-bin/carddisp.pl?gene=LINC01554 |
| GRN | 1.286450386 | https://www.genecards.org/cgi-bin/carddisp.pl?gene=GRN |
| PYCARD | 1.286450386 | https://www.genecards.org/cgi-bin/carddisp.pl?gene=PYCARD |
| VOPP1 | 1.286450386 | https://www.genecards.org/cgi-bin/carddisp.pl?gene=VOPP1 |
| TRN-GTT2-5 | 1.286450386 | https://www.genecards.org/cgi-bin/carddisp.pl?gene=TRN-GTT2-5 |
| TRN-GTT2-6 | 1.286450386 | https://www.genecards.org/cgi-bin/carddisp.pl?gene=TRN-GTT2-6 |
| TRN-GTT2-1 | 1.286450386 | https://www.genecards.org/cgi-bin/carddisp.pl?gene=TRN-GTT2-1 |
| TRN-GTT2-3 | 1.286450386 | https://www.genecards.org/cgi-bin/carddisp.pl?gene=TRN-GTT2-3 |
| TRN-GTT2-2 | 1.286450386 | https://www.genecards.org/cgi-bin/carddisp.pl?gene=TRN-GTT2-2 |
| TRN-GTT2-4 | 1.286450386 | https://www.genecards.org/cgi-bin/carddisp.pl?gene=TRN-GTT2-4 |
| TRN-GTT2-7 | 1.286450386 | https://www.genecards.org/cgi-bin/carddisp.pl?gene=TRN-GTT2-7 |
| TRN-GTT2-8 | 1.286450386 | https://www.genecards.org/cgi-bin/carddisp.pl?gene=TRN-GTT2-8 |
| INSR | 1.280610204 | https://www.genecards.org/cgi-bin/carddisp.pl?gene=INSR |
| BRD4 | 1.280610204 | https://www.genecards.org/cgi-bin/carddisp.pl?gene=BRD4 |
| PDE4A | 1.280610204 | https://www.genecards.org/cgi-bin/carddisp.pl?gene=PDE4A |
| CSRP1 | 1.280610204 | https://www.genecards.org/cgi-bin/carddisp.pl?gene=CSRP1 |
| ERBB2 | 1.272481441 | https://www.genecards.org/cgi-bin/carddisp.pl?gene=ERBB2 |
| HLA-B | 1.272481441 | https://www.genecards.org/cgi-bin/carddisp.pl?gene=HLA-B |
| MMUT | 1.272481441 | https://www.genecards.org/cgi-bin/carddisp.pl?gene=MMUT |
| RHOA | 1.27086091 | https://www.genecards.org/cgi-bin/carddisp.pl?gene=RHOA |
| ITGB1 | 1.269902587 | https://www.genecards.org/cgi-bin/carddisp.pl?gene=ITGB1 |
| APOC1 | 1.258991003 | https://www.genecards.org/cgi-bin/carddisp.pl?gene=APOC1 |
| ZNF644 | 1.258991003 | https://www.genecards.org/cgi-bin/carddisp.pl?gene=ZNF644 |
| MIR29A | 1.258991003 | https://www.genecards.org/cgi-bin/carddisp.pl?gene=MIR29A |
| SNHG7 | 1.258991003 | https://www.genecards.org/cgi-bin/carddisp.pl?gene=SNHG7 |
| MIR138-1 | 1.258991003 | https://www.genecards.org/cgi-bin/carddisp.pl?gene=MIR138-1 |
| MIR138-2 | 1.258991003 | https://www.genecards.org/cgi-bin/carddisp.pl?gene=MIR138-2 |
| MIR195 | 1.258991003 | https://www.genecards.org/cgi-bin/carddisp.pl?gene=MIR195 |
| LINC-ROR | 1.258991003 | https://www.genecards.org/cgi-bin/carddisp.pl?gene=LINC-ROR |
| CORT | 1.252752423 | https://www.genecards.org/cgi-bin/carddisp.pl?gene=CORT |
| MIR326 | 1.252752423 | https://www.genecards.org/cgi-bin/carddisp.pl?gene=MIR326 |
| CLOCK | 1.243241549 | https://www.genecards.org/cgi-bin/carddisp.pl?gene=CLOCK |
| DMD | 1.242318392 | https://www.genecards.org/cgi-bin/carddisp.pl?gene=DMD |
| STIM1 | 1.235972762 | https://www.genecards.org/cgi-bin/carddisp.pl?gene=STIM1 |
| PLTP | 1.235972762 | https://www.genecards.org/cgi-bin/carddisp.pl?gene=PLTP |
| CXCL6 | 1.235972762 | https://www.genecards.org/cgi-bin/carddisp.pl?gene=CXCL6 |
| IGFBP3 | 1.234928966 | https://www.genecards.org/cgi-bin/carddisp.pl?gene=IGFBP3 |
| BCKDK | 1.234928966 | https://www.genecards.org/cgi-bin/carddisp.pl?gene=BCKDK |
| IL3RA | 1.234928966 | https://www.genecards.org/cgi-bin/carddisp.pl?gene=IL3RA |
| USF2 | 1.234928966 | https://www.genecards.org/cgi-bin/carddisp.pl?gene=USF2 |
| MIR7-3 | 1.227847338 | https://www.genecards.org/cgi-bin/carddisp.pl?gene=MIR7-3 |
| MIR7-2 | 1.227847338 | https://www.genecards.org/cgi-bin/carddisp.pl?gene=MIR7-2 |
| LEPR | 1.216346025 | https://www.genecards.org/cgi-bin/carddisp.pl?gene=LEPR |
| ROCK1 | 1.216346025 | https://www.genecards.org/cgi-bin/carddisp.pl?gene=ROCK1 |
| BCL2L1 | 1.216346025 | https://www.genecards.org/cgi-bin/carddisp.pl?gene=BCL2L1 |
| ENG | 1.216346025 | https://www.genecards.org/cgi-bin/carddisp.pl?gene=ENG |
| A2M | 1.216346025 | https://www.genecards.org/cgi-bin/carddisp.pl?gene=A2M |
| BIRC5 | 1.216346025 | https://www.genecards.org/cgi-bin/carddisp.pl?gene=BIRC5 |
| CS | 1.216346025 | https://www.genecards.org/cgi-bin/carddisp.pl?gene=CS |
| FABP4 | 1.216346025 | https://www.genecards.org/cgi-bin/carddisp.pl?gene=FABP4 |
| DUSP22 | 1.216346025 | https://www.genecards.org/cgi-bin/carddisp.pl?gene=DUSP22 |
| STAP2 | 1.216346025 | https://www.genecards.org/cgi-bin/carddisp.pl?gene=STAP2 |
| TRIM7 | 1.216346025 | https://www.genecards.org/cgi-bin/carddisp.pl?gene=TRIM7 |
| CDKN2B-AS1 | 1.216346025 | https://www.genecards.org/cgi-bin/carddisp.pl?gene=CDKN2B-AS1 |
| MIR191 | 1.216346025 | https://www.genecards.org/cgi-bin/carddisp.pl?gene=MIR191 |
| MIR452 | 1.216346025 | https://www.genecards.org/cgi-bin/carddisp.pl?gene=MIR452 |
| OVCH1-AS1 | 1.216346025 | https://www.genecards.org/cgi-bin/carddisp.pl?gene=OVCH1-AS1 |
| ATP2B1-AS1 | 1.213726044 | https://www.genecards.org/cgi-bin/carddisp.pl?gene=ATP2B1-AS1 |
| SLC2A4 | 1.211242676 | https://www.genecards.org/cgi-bin/carddisp.pl?gene=SLC2A4 |
| NTN1 | 1.211242676 | https://www.genecards.org/cgi-bin/carddisp.pl?gene=NTN1 |
| SIRT6 | 1.211242676 | https://www.genecards.org/cgi-bin/carddisp.pl?gene=SIRT6 |
| AGO2 | 1.211242676 | https://www.genecards.org/cgi-bin/carddisp.pl?gene=AGO2 |
| CD63 | 1.211242676 | https://www.genecards.org/cgi-bin/carddisp.pl?gene=CD63 |
| TMSB4X | 1.211242676 | https://www.genecards.org/cgi-bin/carddisp.pl?gene=TMSB4X |
| C1D | 1.211242676 | https://www.genecards.org/cgi-bin/carddisp.pl?gene=C1D |
| MIR141 | 1.211242676 | https://www.genecards.org/cgi-bin/carddisp.pl?gene=MIR141 |
| MIR449C | 1.211242676 | https://www.genecards.org/cgi-bin/carddisp.pl?gene=MIR449C |
| LMAN1 | 1.21002388 | https://www.genecards.org/cgi-bin/carddisp.pl?gene=LMAN1 |
| ADRB2 | 1.209551215 | https://www.genecards.org/cgi-bin/carddisp.pl?gene=ADRB2 |
| SUCLA2 | 1.206901312 | https://www.genecards.org/cgi-bin/carddisp.pl?gene=SUCLA2 |
| SUCLG1 | 1.206901312 | https://www.genecards.org/cgi-bin/carddisp.pl?gene=SUCLG1 |
| TEC | 1.206901312 | https://www.genecards.org/cgi-bin/carddisp.pl?gene=TEC |
| CHKB | 1.206901312 | https://www.genecards.org/cgi-bin/carddisp.pl?gene=CHKB |
| KRT16 | 1.206901312 | https://www.genecards.org/cgi-bin/carddisp.pl?gene=KRT16 |
| IFITM3 | 1.206901312 | https://www.genecards.org/cgi-bin/carddisp.pl?gene=IFITM3 |
| KRT6B | 1.206901312 | https://www.genecards.org/cgi-bin/carddisp.pl?gene=KRT6B |
| TGM5 | 1.206901312 | https://www.genecards.org/cgi-bin/carddisp.pl?gene=TGM5 |
| ALG3 | 1.206901312 | https://www.genecards.org/cgi-bin/carddisp.pl?gene=ALG3 |
| CPSF4 | 1.206901312 | https://www.genecards.org/cgi-bin/carddisp.pl?gene=CPSF4 |
| MAVS | 1.206901312 | https://www.genecards.org/cgi-bin/carddisp.pl?gene=MAVS |
| PEX19 | 1.20161581 | https://www.genecards.org/cgi-bin/carddisp.pl?gene=PEX19 |
| SMARCD2 | 1.20161581 | https://www.genecards.org/cgi-bin/carddisp.pl?gene=SMARCD2 |
| JAK2 | 1.196897507 | https://www.genecards.org/cgi-bin/carddisp.pl?gene=JAK2 |
| CTH | 1.196897507 | https://www.genecards.org/cgi-bin/carddisp.pl?gene=CTH |
| SRPK1 | 1.196897507 | https://www.genecards.org/cgi-bin/carddisp.pl?gene=SRPK1 |
| INPP5D | 1.196897507 | https://www.genecards.org/cgi-bin/carddisp.pl?gene=INPP5D |
| NPC2 | 1.196897507 | https://www.genecards.org/cgi-bin/carddisp.pl?gene=NPC2 |
| MFGE8 | 1.196897507 | https://www.genecards.org/cgi-bin/carddisp.pl?gene=MFGE8 |
| OSM | 1.196897507 | https://www.genecards.org/cgi-bin/carddisp.pl?gene=OSM |
| GPR65 | 1.196897507 | https://www.genecards.org/cgi-bin/carddisp.pl?gene=GPR65 |
| MIR140 | 1.196897507 | https://www.genecards.org/cgi-bin/carddisp.pl?gene=MIR140 |
| MIR3165 | 1.196897507 | https://www.genecards.org/cgi-bin/carddisp.pl?gene=MIR3165 |
| MIR4321 | 1.196897507 | https://www.genecards.org/cgi-bin/carddisp.pl?gene=MIR4321 |
| MIR5094 | 1.196897507 | https://www.genecards.org/cgi-bin/carddisp.pl?gene=MIR5094 |
| MIR4456 | 1.196897507 | https://www.genecards.org/cgi-bin/carddisp.pl?gene=MIR4456 |
| MIR4456-001 | 1.196897507 | https://www.genecards.org/cgi-bin/carddisp.pl?gene=MIR4456-001 |
| AXIN2 | 1.193749189 | https://www.genecards.org/cgi-bin/carddisp.pl?gene=AXIN2 |
| MAN2B1 | 1.193749189 | https://www.genecards.org/cgi-bin/carddisp.pl?gene=MAN2B1 |
| FOXN1 | 1.193749189 | https://www.genecards.org/cgi-bin/carddisp.pl?gene=FOXN1 |
| NOD1 | 1.190968275 | https://www.genecards.org/cgi-bin/carddisp.pl?gene=NOD1 |
| CDH1 | 1.185227752 | https://www.genecards.org/cgi-bin/carddisp.pl?gene=CDH1 |
| ACTG1 | 1.185227752 | https://www.genecards.org/cgi-bin/carddisp.pl?gene=ACTG1 |
| CHRM3 | 1.185227752 | https://www.genecards.org/cgi-bin/carddisp.pl?gene=CHRM3 |
| ACTA2 | 1.185227752 | https://www.genecards.org/cgi-bin/carddisp.pl?gene=ACTA2 |
| EPHX2 | 1.185227752 | https://www.genecards.org/cgi-bin/carddisp.pl?gene=EPHX2 |
| GOT2 | 1.185227752 | https://www.genecards.org/cgi-bin/carddisp.pl?gene=GOT2 |
| ABHD5 | 1.185227752 | https://www.genecards.org/cgi-bin/carddisp.pl?gene=ABHD5 |
| CHRNA3 | 1.185227752 | https://www.genecards.org/cgi-bin/carddisp.pl?gene=CHRNA3 |
| TBX21 | 1.185227752 | https://www.genecards.org/cgi-bin/carddisp.pl?gene=TBX21 |
| CHRNB4 | 1.185227752 | https://www.genecards.org/cgi-bin/carddisp.pl?gene=CHRNB4 |
| LGR5 | 1.185227752 | https://www.genecards.org/cgi-bin/carddisp.pl?gene=LGR5 |
| MYOCD | 1.185227752 | https://www.genecards.org/cgi-bin/carddisp.pl?gene=MYOCD |
| NDUFA9 | 1.185227752 | https://www.genecards.org/cgi-bin/carddisp.pl?gene=NDUFA9 |
| TST | 1.185227752 | https://www.genecards.org/cgi-bin/carddisp.pl?gene=TST |
| LRIG2 | 1.185227752 | https://www.genecards.org/cgi-bin/carddisp.pl?gene=LRIG2 |
| SGO1 | 1.185227752 | https://www.genecards.org/cgi-bin/carddisp.pl?gene=SGO1 |
| CFHR2 | 1.185227752 | https://www.genecards.org/cgi-bin/carddisp.pl?gene=CFHR2 |
| CLDN3 | 1.185227752 | https://www.genecards.org/cgi-bin/carddisp.pl?gene=CLDN3 |
| KLRG1 | 1.185227752 | https://www.genecards.org/cgi-bin/carddisp.pl?gene=KLRG1 |
| LMOD3 | 1.185227752 | https://www.genecards.org/cgi-bin/carddisp.pl?gene=LMOD3 |
| SIGIRR | 1.185227752 | https://www.genecards.org/cgi-bin/carddisp.pl?gene=SIGIRR |
| TMOD1 | 1.185227752 | https://www.genecards.org/cgi-bin/carddisp.pl?gene=TMOD1 |
| DDX59 | 1.185227752 | https://www.genecards.org/cgi-bin/carddisp.pl?gene=DDX59 |
| IVL | 1.185227752 | https://www.genecards.org/cgi-bin/carddisp.pl?gene=IVL |
| LGI2 | 1.185227752 | https://www.genecards.org/cgi-bin/carddisp.pl?gene=LGI2 |
| LALBA | 1.185227752 | https://www.genecards.org/cgi-bin/carddisp.pl?gene=LALBA |
| SMTN | 1.185227752 | https://www.genecards.org/cgi-bin/carddisp.pl?gene=SMTN |
| PDCL3 | 1.185227752 | https://www.genecards.org/cgi-bin/carddisp.pl?gene=PDCL3 |
| BAHD1 | 1.185227752 | https://www.genecards.org/cgi-bin/carddisp.pl?gene=BAHD1 |
| LMOD2 | 1.185227752 | https://www.genecards.org/cgi-bin/carddisp.pl?gene=LMOD2 |
| LORICRIN | 1.185227752 | https://www.genecards.org/cgi-bin/carddisp.pl?gene=LORICRIN |
| RBPMS2 | 1.185227752 | https://www.genecards.org/cgi-bin/carddisp.pl?gene=RBPMS2 |
| PCDHA7 | 1.185227752 | https://www.genecards.org/cgi-bin/carddisp.pl?gene=PCDHA7 |
| CCDC97 | 1.185227752 | https://www.genecards.org/cgi-bin/carddisp.pl?gene=CCDC97 |
| PYDC1 | 1.185227752 | https://www.genecards.org/cgi-bin/carddisp.pl?gene=PYDC1 |
| ALPL | 1.184350371 | https://www.genecards.org/cgi-bin/carddisp.pl?gene=ALPL |
| MMP14 | 1.184350371 | https://www.genecards.org/cgi-bin/carddisp.pl?gene=MMP14 |
| GLUL | 1.184350371 | https://www.genecards.org/cgi-bin/carddisp.pl?gene=GLUL |
| CD59 | 1.184350371 | https://www.genecards.org/cgi-bin/carddisp.pl?gene=CD59 |
| CYP2E1 | 1.184350371 | https://www.genecards.org/cgi-bin/carddisp.pl?gene=CYP2E1 |
| PDE4B | 1.184350371 | https://www.genecards.org/cgi-bin/carddisp.pl?gene=PDE4B |
| FCN2 | 1.184350371 | https://www.genecards.org/cgi-bin/carddisp.pl?gene=FCN2 |
| AGPAT1 | 1.184350371 | https://www.genecards.org/cgi-bin/carddisp.pl?gene=AGPAT1 |
| TREML1 | 1.184350371 | https://www.genecards.org/cgi-bin/carddisp.pl?gene=TREML1 |
| DSC1 | 1.181607485 | https://www.genecards.org/cgi-bin/carddisp.pl?gene=DSC1 |
| TGFBR2 | 1.176449537 | https://www.genecards.org/cgi-bin/carddisp.pl?gene=TGFBR2 |
| HDAC5 | 1.176449537 | https://www.genecards.org/cgi-bin/carddisp.pl?gene=HDAC5 |
| MALT1 | 1.176449537 | https://www.genecards.org/cgi-bin/carddisp.pl?gene=MALT1 |
| PRKCI | 1.176449537 | https://www.genecards.org/cgi-bin/carddisp.pl?gene=PRKCI |
| MC1R | 1.176449537 | https://www.genecards.org/cgi-bin/carddisp.pl?gene=MC1R |
| P2RY12 | 1.176449537 | https://www.genecards.org/cgi-bin/carddisp.pl?gene=P2RY12 |
| TMPO | 1.176449537 | https://www.genecards.org/cgi-bin/carddisp.pl?gene=TMPO |
| KDM6B | 1.176449537 | https://www.genecards.org/cgi-bin/carddisp.pl?gene=KDM6B |
| BAK1 | 1.176449537 | https://www.genecards.org/cgi-bin/carddisp.pl?gene=BAK1 |
| NONO | 1.176449537 | https://www.genecards.org/cgi-bin/carddisp.pl?gene=NONO |
| CFLAR | 1.176449537 | https://www.genecards.org/cgi-bin/carddisp.pl?gene=CFLAR |
| CCL20 | 1.176449537 | https://www.genecards.org/cgi-bin/carddisp.pl?gene=CCL20 |
| GRPR | 1.176449537 | https://www.genecards.org/cgi-bin/carddisp.pl?gene=GRPR |
| PDCD1LG2 | 1.176449537 | https://www.genecards.org/cgi-bin/carddisp.pl?gene=PDCD1LG2 |
| TDRG1 | 1.176449537 | https://www.genecards.org/cgi-bin/carddisp.pl?gene=TDRG1 |
| MIR185 | 1.176449537 | https://www.genecards.org/cgi-bin/carddisp.pl?gene=MIR185 |
| EGOT | 1.176449537 | https://www.genecards.org/cgi-bin/carddisp.pl?gene=EGOT |
| ST7-OT3 | 1.176449537 | https://www.genecards.org/cgi-bin/carddisp.pl?gene=ST7-OT3 |
| VTRNA2-1 | 1.176449537 | https://www.genecards.org/cgi-bin/carddisp.pl?gene=VTRNA2-1 |
| P2RX5-TAX1BP3 | 1.176449537 | https://www.genecards.org/cgi-bin/carddisp.pl?gene=P2RX5-TAX1BP3 |
| LOC124906209 | 1.176449537 | https://www.genecards.org/cgi-bin/carddisp.pl?gene=LOC124906209 |
| B2M | 1.171519637 | https://www.genecards.org/cgi-bin/carddisp.pl?gene=B2M |
| RAC1 | 1.155551195 | https://www.genecards.org/cgi-bin/carddisp.pl?gene=RAC1 |
| HAX1 | 1.155429244 | https://www.genecards.org/cgi-bin/carddisp.pl?gene=HAX1 |
| PTPN11 | 1.154830337 | https://www.genecards.org/cgi-bin/carddisp.pl?gene=PTPN11 |
| CREB1 | 1.154830337 | https://www.genecards.org/cgi-bin/carddisp.pl?gene=CREB1 |
| PROS1 | 1.154830337 | https://www.genecards.org/cgi-bin/carddisp.pl?gene=PROS1 |
| KDM6A | 1.154830337 | https://www.genecards.org/cgi-bin/carddisp.pl?gene=KDM6A |
| ODC1 | 1.154830337 | https://www.genecards.org/cgi-bin/carddisp.pl?gene=ODC1 |
| GP9 | 1.154830337 | https://www.genecards.org/cgi-bin/carddisp.pl?gene=GP9 |
| AVPR2 | 1.154830337 | https://www.genecards.org/cgi-bin/carddisp.pl?gene=AVPR2 |
| FEN1 | 1.154830337 | https://www.genecards.org/cgi-bin/carddisp.pl?gene=FEN1 |
| PAX5 | 1.154830337 | https://www.genecards.org/cgi-bin/carddisp.pl?gene=PAX5 |
| PHB1 | 1.154830337 | https://www.genecards.org/cgi-bin/carddisp.pl?gene=PHB1 |
| GDF2 | 1.154830337 | https://www.genecards.org/cgi-bin/carddisp.pl?gene=GDF2 |
| AZGP1 | 1.154830337 | https://www.genecards.org/cgi-bin/carddisp.pl?gene=AZGP1 |
| BACH2 | 1.154830337 | https://www.genecards.org/cgi-bin/carddisp.pl?gene=BACH2 |
| BCL11B | 1.154830337 | https://www.genecards.org/cgi-bin/carddisp.pl?gene=BCL11B |
| SLC10A1 | 1.154830337 | https://www.genecards.org/cgi-bin/carddisp.pl?gene=SLC10A1 |
| AOC1 | 1.154830337 | https://www.genecards.org/cgi-bin/carddisp.pl?gene=AOC1 |
| TNFSF4 | 1.154830337 | https://www.genecards.org/cgi-bin/carddisp.pl?gene=TNFSF4 |
| CXCL13 | 1.154830337 | https://www.genecards.org/cgi-bin/carddisp.pl?gene=CXCL13 |
| BGLAP | 1.154830337 | https://www.genecards.org/cgi-bin/carddisp.pl?gene=BGLAP |
| EDIL3 | 1.154830337 | https://www.genecards.org/cgi-bin/carddisp.pl?gene=EDIL3 |
| H3C1 | 1.154830337 | https://www.genecards.org/cgi-bin/carddisp.pl?gene=H3C1 |
| IL31 | 1.154830337 | https://www.genecards.org/cgi-bin/carddisp.pl?gene=IL31 |
| IFNL3 | 1.154830337 | https://www.genecards.org/cgi-bin/carddisp.pl?gene=IFNL3 |
| TP53TG1 | 1.154830337 | https://www.genecards.org/cgi-bin/carddisp.pl?gene=TP53TG1 |
| PRECSIT | 1.154830337 | https://www.genecards.org/cgi-bin/carddisp.pl?gene=PRECSIT |
| MIR375 | 1.154830337 | https://www.genecards.org/cgi-bin/carddisp.pl?gene=MIR375 |
| OIP5-AS1 | 1.154830337 | https://www.genecards.org/cgi-bin/carddisp.pl?gene=OIP5-AS1 |
| CBR3-AS1 | 1.154830337 | https://www.genecards.org/cgi-bin/carddisp.pl?gene=CBR3-AS1 |
| ITGB2-AS1 | 1.154830337 | https://www.genecards.org/cgi-bin/carddisp.pl?gene=ITGB2-AS1 |
| LINC00963 | 1.154830337 | https://www.genecards.org/cgi-bin/carddisp.pl?gene=LINC00963 |
| THRIL | 1.154830337 | https://www.genecards.org/cgi-bin/carddisp.pl?gene=THRIL |
| LOC100131496 | 1.154830337 | https://www.genecards.org/cgi-bin/carddisp.pl?gene=LOC100131496 |
| XLOC_007697 | 1.154830337 | https://www.genecards.org/cgi-bin/carddisp.pl?gene=XLOC_007697 |
| HNP1 | 1.154830337 | https://www.genecards.org/cgi-bin/carddisp.pl?gene=HNP1 |
| AQP2 | 1.154604435 | https://www.genecards.org/cgi-bin/carddisp.pl?gene=AQP2 |
| KLKB1 | 1.154604435 | https://www.genecards.org/cgi-bin/carddisp.pl?gene=KLKB1 |
| SERPINB2 | 1.151544452 | https://www.genecards.org/cgi-bin/carddisp.pl?gene=SERPINB2 |
| TCN2 | 1.15099895 | https://www.genecards.org/cgi-bin/carddisp.pl?gene=TCN2 |
| ERBB3 | 1.146475673 | https://www.genecards.org/cgi-bin/carddisp.pl?gene=ERBB3 |
| RET | 1.146475673 | https://www.genecards.org/cgi-bin/carddisp.pl?gene=RET |
| IDH1 | 1.146475673 | https://www.genecards.org/cgi-bin/carddisp.pl?gene=IDH1 |
| SMO | 1.146475673 | https://www.genecards.org/cgi-bin/carddisp.pl?gene=SMO |
| GDNF | 1.146475673 | https://www.genecards.org/cgi-bin/carddisp.pl?gene=GDNF |
| ECE1 | 1.146475673 | https://www.genecards.org/cgi-bin/carddisp.pl?gene=ECE1 |
| IKZF1 | 1.146475673 | https://www.genecards.org/cgi-bin/carddisp.pl?gene=IKZF1 |
| NCF4 | 1.146475673 | https://www.genecards.org/cgi-bin/carddisp.pl?gene=NCF4 |
| SREBF1 | 1.146475673 | https://www.genecards.org/cgi-bin/carddisp.pl?gene=SREBF1 |
| NCF2 | 1.146475673 | https://www.genecards.org/cgi-bin/carddisp.pl?gene=NCF2 |
| ABCD1 | 1.146475673 | https://www.genecards.org/cgi-bin/carddisp.pl?gene=ABCD1 |
| EDN3 | 1.146475673 | https://www.genecards.org/cgi-bin/carddisp.pl?gene=EDN3 |
| TCF3 | 1.146475673 | https://www.genecards.org/cgi-bin/carddisp.pl?gene=TCF3 |
| LAMA3 | 1.146475673 | https://www.genecards.org/cgi-bin/carddisp.pl?gene=LAMA3 |
| SEMA3C | 1.146475673 | https://www.genecards.org/cgi-bin/carddisp.pl?gene=SEMA3C |
| IGLL1 | 1.146475673 | https://www.genecards.org/cgi-bin/carddisp.pl?gene=IGLL1 |
| SRP54 | 1.146475673 | https://www.genecards.org/cgi-bin/carddisp.pl?gene=SRP54 |
| GJB6 | 1.146475673 | https://www.genecards.org/cgi-bin/carddisp.pl?gene=GJB6 |
| RNF13 | 1.146475673 | https://www.genecards.org/cgi-bin/carddisp.pl?gene=RNF13 |
| TREX1 | 1.146475673 | https://www.genecards.org/cgi-bin/carddisp.pl?gene=TREX1 |
| VPS33A | 1.146475673 | https://www.genecards.org/cgi-bin/carddisp.pl?gene=VPS33A |
| LRRC8A | 1.146475673 | https://www.genecards.org/cgi-bin/carddisp.pl?gene=LRRC8A |
| NRTN | 1.146475673 | https://www.genecards.org/cgi-bin/carddisp.pl?gene=NRTN |
| SEMA3D | 1.146475673 | https://www.genecards.org/cgi-bin/carddisp.pl?gene=SEMA3D |
| EFL1 | 1.146475673 | https://www.genecards.org/cgi-bin/carddisp.pl?gene=EFL1 |
| ASPRV1 | 1.146475673 | https://www.genecards.org/cgi-bin/carddisp.pl?gene=ASPRV1 |
| DNAJC21 | 1.146475673 | https://www.genecards.org/cgi-bin/carddisp.pl?gene=DNAJC21 |
| CYBC1 | 1.146475673 | https://www.genecards.org/cgi-bin/carddisp.pl?gene=CYBC1 |
| IGKC | 1.146475673 | https://www.genecards.org/cgi-bin/carddisp.pl?gene=IGKC |
| IGHG2 | 1.146475673 | https://www.genecards.org/cgi-bin/carddisp.pl?gene=IGHG2 |
| SLC27A4 | 1.141424537 | https://www.genecards.org/cgi-bin/carddisp.pl?gene=SLC27A4 |
| CYP21A2 | 1.141424537 | https://www.genecards.org/cgi-bin/carddisp.pl?gene=CYP21A2 |
| CERS3 | 1.141424537 | https://www.genecards.org/cgi-bin/carddisp.pl?gene=CERS3 |
| F10 | 1.135498285 | https://www.genecards.org/cgi-bin/carddisp.pl?gene=F10 |
| AGTR1 | 1.131812096 | https://www.genecards.org/cgi-bin/carddisp.pl?gene=AGTR1 |
| RPS6KB1 | 1.131812096 | https://www.genecards.org/cgi-bin/carddisp.pl?gene=RPS6KB1 |
| CASP4 | 1.131812096 | https://www.genecards.org/cgi-bin/carddisp.pl?gene=CASP4 |
| DIABLO | 1.131812096 | https://www.genecards.org/cgi-bin/carddisp.pl?gene=DIABLO |
| LPIN1 | 1.131812096 | https://www.genecards.org/cgi-bin/carddisp.pl?gene=LPIN1 |
| PDP1 | 1.131812096 | https://www.genecards.org/cgi-bin/carddisp.pl?gene=PDP1 |
| SERPINF1 | 1.131812096 | https://www.genecards.org/cgi-bin/carddisp.pl?gene=SERPINF1 |
| SOCS1 | 1.131812096 | https://www.genecards.org/cgi-bin/carddisp.pl?gene=SOCS1 |
| APOC3 | 1.131812096 | https://www.genecards.org/cgi-bin/carddisp.pl?gene=APOC3 |
| ATG5 | 1.131812096 | https://www.genecards.org/cgi-bin/carddisp.pl?gene=ATG5 |
| MKNK1 | 1.131812096 | https://www.genecards.org/cgi-bin/carddisp.pl?gene=MKNK1 |
| HSPA2 | 1.131812096 | https://www.genecards.org/cgi-bin/carddisp.pl?gene=HSPA2 |
| PRSS1 | 1.131812096 | https://www.genecards.org/cgi-bin/carddisp.pl?gene=PRSS1 |
| TFAM | 1.131812096 | https://www.genecards.org/cgi-bin/carddisp.pl?gene=TFAM |
| TRPM7 | 1.131812096 | https://www.genecards.org/cgi-bin/carddisp.pl?gene=TRPM7 |
| CHGA | 1.131812096 | https://www.genecards.org/cgi-bin/carddisp.pl?gene=CHGA |
| RAMP3 | 1.131812096 | https://www.genecards.org/cgi-bin/carddisp.pl?gene=RAMP3 |
| SOCS6 | 1.131812096 | https://www.genecards.org/cgi-bin/carddisp.pl?gene=SOCS6 |
| SORBS2 | 1.131812096 | https://www.genecards.org/cgi-bin/carddisp.pl?gene=SORBS2 |
| SPOCK1 | 1.131812096 | https://www.genecards.org/cgi-bin/carddisp.pl?gene=SPOCK1 |
| SDF4 | 1.131812096 | https://www.genecards.org/cgi-bin/carddisp.pl?gene=SDF4 |
| STEAP1 | 1.131812096 | https://www.genecards.org/cgi-bin/carddisp.pl?gene=STEAP1 |
| CXCL14 | 1.131812096 | https://www.genecards.org/cgi-bin/carddisp.pl?gene=CXCL14 |
| GPR174 | 1.131812096 | https://www.genecards.org/cgi-bin/carddisp.pl?gene=GPR174 |
| ZBTB7C | 1.131812096 | https://www.genecards.org/cgi-bin/carddisp.pl?gene=ZBTB7C |
| AKIRIN1 | 1.131812096 | https://www.genecards.org/cgi-bin/carddisp.pl?gene=AKIRIN1 |
| SERP1 | 1.131812096 | https://www.genecards.org/cgi-bin/carddisp.pl?gene=SERP1 |
| TRB | 1.131812096 | https://www.genecards.org/cgi-bin/carddisp.pl?gene=TRB |
| LINC00261 | 1.131812096 | https://www.genecards.org/cgi-bin/carddisp.pl?gene=LINC00261 |
| MIR133B | 1.131812096 | https://www.genecards.org/cgi-bin/carddisp.pl?gene=MIR133B |
| MIR205 | 1.131812096 | https://www.genecards.org/cgi-bin/carddisp.pl?gene=MIR205 |
| MIR217 | 1.131812096 | https://www.genecards.org/cgi-bin/carddisp.pl?gene=MIR217 |
| MIR373 | 1.131812096 | https://www.genecards.org/cgi-bin/carddisp.pl?gene=MIR373 |
| MIR1262 | 1.131812096 | https://www.genecards.org/cgi-bin/carddisp.pl?gene=MIR1262 |
| MIR1298 | 1.131812096 | https://www.genecards.org/cgi-bin/carddisp.pl?gene=MIR1298 |
| ENSG00000229278 | 1.131812096 | https://www.genecards.org/cgi-bin/carddisp.pl?gene=ENSG00000229278 |
| ST2 | 1.131812096 | https://www.genecards.org/cgi-bin/carddisp.pl?gene=ST2 |
| DEFA1A3 | 1.131812096 | https://www.genecards.org/cgi-bin/carddisp.pl?gene=DEFA1A3 |
| lnc-HJURP-12 | 1.131812096 | https://www.genecards.org/cgi-bin/carddisp.pl?gene=lnc-HJURP-12 |
| RF03470-004 | 1.131812096 | https://www.genecards.org/cgi-bin/carddisp.pl?gene=RF03470-004 |
| NAXE | 1.130745173 | https://www.genecards.org/cgi-bin/carddisp.pl?gene=NAXE |
| TAC1 | 1.129452586 | https://www.genecards.org/cgi-bin/carddisp.pl?gene=TAC1 |
| TRP-AGG2-5 | 1.129226685 | https://www.genecards.org/cgi-bin/carddisp.pl?gene=TRP-AGG2-5 |
| TRP-AGG2-6 | 1.129226685 | https://www.genecards.org/cgi-bin/carddisp.pl?gene=TRP-AGG2-6 |
| TRP-AGG2-1 | 1.129226685 | https://www.genecards.org/cgi-bin/carddisp.pl?gene=TRP-AGG2-1 |
| TRP-AGG2-2 | 1.129226685 | https://www.genecards.org/cgi-bin/carddisp.pl?gene=TRP-AGG2-2 |
| TRP-AGG2-3 | 1.129226685 | https://www.genecards.org/cgi-bin/carddisp.pl?gene=TRP-AGG2-3 |
| TRP-AGG2-4 | 1.129226685 | https://www.genecards.org/cgi-bin/carddisp.pl?gene=TRP-AGG2-4 |
| TRP-AGG2-7 | 1.129226685 | https://www.genecards.org/cgi-bin/carddisp.pl?gene=TRP-AGG2-7 |
| TRP-AGG2-8 | 1.129226685 | https://www.genecards.org/cgi-bin/carddisp.pl?gene=TRP-AGG2-8 |
| CRBN | 1.120841265 | https://www.genecards.org/cgi-bin/carddisp.pl?gene=CRBN |
| FNDC5 | 1.120841265 | https://www.genecards.org/cgi-bin/carddisp.pl?gene=FNDC5 |
| MIR192 | 1.120841265 | https://www.genecards.org/cgi-bin/carddisp.pl?gene=MIR192 |
| HSPG2 | 1.116858006 | https://www.genecards.org/cgi-bin/carddisp.pl?gene=HSPG2 |
| C1R | 1.116858006 | https://www.genecards.org/cgi-bin/carddisp.pl?gene=C1R |
| BAMBI | 1.116858006 | https://www.genecards.org/cgi-bin/carddisp.pl?gene=BAMBI |
| DSG3 | 1.116858006 | https://www.genecards.org/cgi-bin/carddisp.pl?gene=DSG3 |
| AGK | 1.116858006 | https://www.genecards.org/cgi-bin/carddisp.pl?gene=AGK |
| ANPEP | 1.107082129 | https://www.genecards.org/cgi-bin/carddisp.pl?gene=ANPEP |
| BDNF | 1.107082129 | https://www.genecards.org/cgi-bin/carddisp.pl?gene=BDNF |
| CAMK2D | 1.107082129 | https://www.genecards.org/cgi-bin/carddisp.pl?gene=CAMK2D |
| TNC | 1.107082129 | https://www.genecards.org/cgi-bin/carddisp.pl?gene=TNC |
| ABCC2 | 1.107082129 | https://www.genecards.org/cgi-bin/carddisp.pl?gene=ABCC2 |
| PTGER2 | 1.107082129 | https://www.genecards.org/cgi-bin/carddisp.pl?gene=PTGER2 |
| YY1 | 1.107082129 | https://www.genecards.org/cgi-bin/carddisp.pl?gene=YY1 |
| EIF4EBP1 | 1.107082129 | https://www.genecards.org/cgi-bin/carddisp.pl?gene=EIF4EBP1 |
| SPHK1 | 1.107082129 | https://www.genecards.org/cgi-bin/carddisp.pl?gene=SPHK1 |
| HLA-DPB1 | 1.107082129 | https://www.genecards.org/cgi-bin/carddisp.pl?gene=HLA-DPB1 |
| CIITA | 1.107082129 | https://www.genecards.org/cgi-bin/carddisp.pl?gene=CIITA |
| ATN1 | 1.107082129 | https://www.genecards.org/cgi-bin/carddisp.pl?gene=ATN1 |
| IGFBP2 | 1.107082129 | https://www.genecards.org/cgi-bin/carddisp.pl?gene=IGFBP2 |
| SOCS2 | 1.107082129 | https://www.genecards.org/cgi-bin/carddisp.pl?gene=SOCS2 |
| TFDP1 | 1.107082129 | https://www.genecards.org/cgi-bin/carddisp.pl?gene=TFDP1 |
| ADGRE2 | 1.107082129 | https://www.genecards.org/cgi-bin/carddisp.pl?gene=ADGRE2 |
| CD84 | 1.107082129 | https://www.genecards.org/cgi-bin/carddisp.pl?gene=CD84 |
| CCN1 | 1.107082129 | https://www.genecards.org/cgi-bin/carddisp.pl?gene=CCN1 |
| FCN1 | 1.107082129 | https://www.genecards.org/cgi-bin/carddisp.pl?gene=FCN1 |
| PTP4A2 | 1.107082129 | https://www.genecards.org/cgi-bin/carddisp.pl?gene=PTP4A2 |
| CD177 | 1.107082129 | https://www.genecards.org/cgi-bin/carddisp.pl?gene=CD177 |
| TRIM8 | 1.107082129 | https://www.genecards.org/cgi-bin/carddisp.pl?gene=TRIM8 |
| EPG5 | 1.107082129 | https://www.genecards.org/cgi-bin/carddisp.pl?gene=EPG5 |
| SFRP5 | 1.107082129 | https://www.genecards.org/cgi-bin/carddisp.pl?gene=SFRP5 |
| SPIB | 1.107082129 | https://www.genecards.org/cgi-bin/carddisp.pl?gene=SPIB |
| SELENOS | 1.107082129 | https://www.genecards.org/cgi-bin/carddisp.pl?gene=SELENOS |
| GADD45B | 1.107082129 | https://www.genecards.org/cgi-bin/carddisp.pl?gene=GADD45B |
| ZNFX1 | 1.107082129 | https://www.genecards.org/cgi-bin/carddisp.pl?gene=ZNFX1 |
| LCN10 | 1.107082129 | https://www.genecards.org/cgi-bin/carddisp.pl?gene=LCN10 |
| MAPKAPK5-AS1 | 1.107082129 | https://www.genecards.org/cgi-bin/carddisp.pl?gene=MAPKAPK5-AS1 |
| MIR433 | 1.107082129 | https://www.genecards.org/cgi-bin/carddisp.pl?gene=MIR433 |
| MCM3AP-AS1 | 1.107082129 | https://www.genecards.org/cgi-bin/carddisp.pl?gene=MCM3AP-AS1 |
| SNHG6 | 1.107082129 | https://www.genecards.org/cgi-bin/carddisp.pl?gene=SNHG6 |
| MIR30C2 | 1.107082129 | https://www.genecards.org/cgi-bin/carddisp.pl?gene=MIR30C2 |
| MIR345 | 1.107082129 | https://www.genecards.org/cgi-bin/carddisp.pl?gene=MIR345 |
| MIR9-2 | 1.107082129 | https://www.genecards.org/cgi-bin/carddisp.pl?gene=MIR9-2 |
| MIR9-3 | 1.107082129 | https://www.genecards.org/cgi-bin/carddisp.pl?gene=MIR9-3 |
| MIRLET7G | 1.107082129 | https://www.genecards.org/cgi-bin/carddisp.pl?gene=MIRLET7G |
| PITX1-AS1 | 1.107082129 | https://www.genecards.org/cgi-bin/carddisp.pl?gene=PITX1-AS1 |
| MIR338 | 1.107082129 | https://www.genecards.org/cgi-bin/carddisp.pl?gene=MIR338 |
| MIR367 | 1.107082129 | https://www.genecards.org/cgi-bin/carddisp.pl?gene=MIR367 |
| MIR526B | 1.107082129 | https://www.genecards.org/cgi-bin/carddisp.pl?gene=MIR526B |
| MIR874 | 1.107082129 | https://www.genecards.org/cgi-bin/carddisp.pl?gene=MIR874 |
| STARD7-AS1 | 1.107082129 | https://www.genecards.org/cgi-bin/carddisp.pl?gene=STARD7-AS1 |
| MIR371A | 1.107082129 | https://www.genecards.org/cgi-bin/carddisp.pl?gene=MIR371A |
| MIR378D2 | 1.107082129 | https://www.genecards.org/cgi-bin/carddisp.pl?gene=MIR378D2 |
| MIR4476 | 1.107082129 | https://www.genecards.org/cgi-bin/carddisp.pl?gene=MIR4476 |
| MIR378D1 | 1.107082129 | https://www.genecards.org/cgi-bin/carddisp.pl?gene=MIR378D1 |
| JAK3 | 1.10645628 | https://www.genecards.org/cgi-bin/carddisp.pl?gene=JAK3 |
| NT5E | 1.106434345 | https://www.genecards.org/cgi-bin/carddisp.pl?gene=NT5E |
| TNNT2 | 1.098747015 | https://www.genecards.org/cgi-bin/carddisp.pl?gene=TNNT2 |
| NLRC4 | 1.083146095 | https://www.genecards.org/cgi-bin/carddisp.pl?gene=NLRC4 |
| EGFR | 1.080189705 | https://www.genecards.org/cgi-bin/carddisp.pl?gene=EGFR |
| PRKCG | 1.080189705 | https://www.genecards.org/cgi-bin/carddisp.pl?gene=PRKCG |
| PRKN | 1.080189705 | https://www.genecards.org/cgi-bin/carddisp.pl?gene=PRKN |
| GLP1R | 1.080189705 | https://www.genecards.org/cgi-bin/carddisp.pl?gene=GLP1R |
| GPX4 | 1.080189705 | https://www.genecards.org/cgi-bin/carddisp.pl?gene=GPX4 |
| HSF1 | 1.080189705 | https://www.genecards.org/cgi-bin/carddisp.pl?gene=HSF1 |
| IL17RA | 1.080189705 | https://www.genecards.org/cgi-bin/carddisp.pl?gene=IL17RA |
| SLC6A8 | 1.080189705 | https://www.genecards.org/cgi-bin/carddisp.pl?gene=SLC6A8 |
| ATF4 | 1.080189705 | https://www.genecards.org/cgi-bin/carddisp.pl?gene=ATF4 |
| SDC2 | 1.080189705 | https://www.genecards.org/cgi-bin/carddisp.pl?gene=SDC2 |
| CCR1 | 1.080189705 | https://www.genecards.org/cgi-bin/carddisp.pl?gene=CCR1 |
| FGF19 | 1.080189705 | https://www.genecards.org/cgi-bin/carddisp.pl?gene=FGF19 |
| PDK4 | 1.080189705 | https://www.genecards.org/cgi-bin/carddisp.pl?gene=PDK4 |
| JMJD6 | 1.080189705 | https://www.genecards.org/cgi-bin/carddisp.pl?gene=JMJD6 |
| PRDX4 | 1.080189705 | https://www.genecards.org/cgi-bin/carddisp.pl?gene=PRDX4 |
| RHOQ | 1.080189705 | https://www.genecards.org/cgi-bin/carddisp.pl?gene=RHOQ |
| SPINT2 | 1.080189705 | https://www.genecards.org/cgi-bin/carddisp.pl?gene=SPINT2 |
| MAP1LC3A | 1.080189705 | https://www.genecards.org/cgi-bin/carddisp.pl?gene=MAP1LC3A |
| AFM | 1.080189705 | https://www.genecards.org/cgi-bin/carddisp.pl?gene=AFM |
| CD248 | 1.080189705 | https://www.genecards.org/cgi-bin/carddisp.pl?gene=CD248 |
| CLEC12A | 1.080189705 | https://www.genecards.org/cgi-bin/carddisp.pl?gene=CLEC12A |
| NLRX1 | 1.080189705 | https://www.genecards.org/cgi-bin/carddisp.pl?gene=NLRX1 |
| UTS2 | 1.080189705 | https://www.genecards.org/cgi-bin/carddisp.pl?gene=UTS2 |
| GPR18 | 1.080189705 | https://www.genecards.org/cgi-bin/carddisp.pl?gene=GPR18 |
| CCL8 | 1.080189705 | https://www.genecards.org/cgi-bin/carddisp.pl?gene=CCL8 |
| LILRB3 | 1.080189705 | https://www.genecards.org/cgi-bin/carddisp.pl?gene=LILRB3 |
| POLDIP2 | 1.080189705 | https://www.genecards.org/cgi-bin/carddisp.pl?gene=POLDIP2 |
| TIFA | 1.080189705 | https://www.genecards.org/cgi-bin/carddisp.pl?gene=TIFA |
| CD24 | 1.080189705 | https://www.genecards.org/cgi-bin/carddisp.pl?gene=CD24 |
| TMT1B | 1.080189705 | https://www.genecards.org/cgi-bin/carddisp.pl?gene=TMT1B |
| NPAS4 | 1.080189705 | https://www.genecards.org/cgi-bin/carddisp.pl?gene=NPAS4 |
| USP50 | 1.080189705 | https://www.genecards.org/cgi-bin/carddisp.pl?gene=USP50 |
| MIR132 | 1.080189705 | https://www.genecards.org/cgi-bin/carddisp.pl?gene=MIR132 |
| MIR152 | 1.080189705 | https://www.genecards.org/cgi-bin/carddisp.pl?gene=MIR152 |
| MIR206 | 1.080189705 | https://www.genecards.org/cgi-bin/carddisp.pl?gene=MIR206 |
| HOXA-AS2 | 1.080189705 | https://www.genecards.org/cgi-bin/carddisp.pl?gene=HOXA-AS2 |
| MIR124-2 | 1.080189705 | https://www.genecards.org/cgi-bin/carddisp.pl?gene=MIR124-2 |
| MIR124-3 | 1.080189705 | https://www.genecards.org/cgi-bin/carddisp.pl?gene=MIR124-3 |
| LINC00426 | 1.080189705 | https://www.genecards.org/cgi-bin/carddisp.pl?gene=LINC00426 |
| MIR361 | 1.080189705 | https://www.genecards.org/cgi-bin/carddisp.pl?gene=MIR361 |
| EDNRB-AS1 | 1.080189705 | https://www.genecards.org/cgi-bin/carddisp.pl?gene=EDNRB-AS1 |
| MIR498 | 1.080189705 | https://www.genecards.org/cgi-bin/carddisp.pl?gene=MIR498 |
| SNORD47 | 1.080189705 | https://www.genecards.org/cgi-bin/carddisp.pl?gene=SNORD47 |
| MIR4634 | 1.080189705 | https://www.genecards.org/cgi-bin/carddisp.pl?gene=MIR4634 |
| MIR8056 | 1.080189705 | https://www.genecards.org/cgi-bin/carddisp.pl?gene=MIR8056 |
| MIR8063 | 1.080189705 | https://www.genecards.org/cgi-bin/carddisp.pl?gene=MIR8063 |
| MIR8085 | 1.080189705 | https://www.genecards.org/cgi-bin/carddisp.pl?gene=MIR8085 |
| MIR7847 | 1.080189705 | https://www.genecards.org/cgi-bin/carddisp.pl?gene=MIR7847 |
| MIR8059 | 1.080189705 | https://www.genecards.org/cgi-bin/carddisp.pl?gene=MIR8059 |
| MIR8061 | 1.080189705 | https://www.genecards.org/cgi-bin/carddisp.pl?gene=MIR8061 |
| MIR8066 | 1.080189705 | https://www.genecards.org/cgi-bin/carddisp.pl?gene=MIR8066 |
| MIR8072 | 1.080189705 | https://www.genecards.org/cgi-bin/carddisp.pl?gene=MIR8072 |
| MIR8073 | 1.080189705 | https://www.genecards.org/cgi-bin/carddisp.pl?gene=MIR8073 |
| MIR8083 | 1.080189705 | https://www.genecards.org/cgi-bin/carddisp.pl?gene=MIR8083 |
| MIR8052 | 1.080189705 | https://www.genecards.org/cgi-bin/carddisp.pl?gene=MIR8052 |
| MIR8055 | 1.080189705 | https://www.genecards.org/cgi-bin/carddisp.pl?gene=MIR8055 |
| MIR8058 | 1.080189705 | https://www.genecards.org/cgi-bin/carddisp.pl?gene=MIR8058 |
| MIR8064 | 1.080189705 | https://www.genecards.org/cgi-bin/carddisp.pl?gene=MIR8064 |
| MIR8076 | 1.080189705 | https://www.genecards.org/cgi-bin/carddisp.pl?gene=MIR8076 |
| MIR8077 | 1.080189705 | https://www.genecards.org/cgi-bin/carddisp.pl?gene=MIR8077 |
| MIR8078 | 1.080189705 | https://www.genecards.org/cgi-bin/carddisp.pl?gene=MIR8078 |
| MIR8079 | 1.080189705 | https://www.genecards.org/cgi-bin/carddisp.pl?gene=MIR8079 |
| MIR8080 | 1.080189705 | https://www.genecards.org/cgi-bin/carddisp.pl?gene=MIR8080 |
| MIR8082 | 1.080189705 | https://www.genecards.org/cgi-bin/carddisp.pl?gene=MIR8082 |
| MIR8086 | 1.080189705 | https://www.genecards.org/cgi-bin/carddisp.pl?gene=MIR8086 |
| MIR8089 | 1.080189705 | https://www.genecards.org/cgi-bin/carddisp.pl?gene=MIR8089 |
| MIR8053 | 1.080189705 | https://www.genecards.org/cgi-bin/carddisp.pl?gene=MIR8053 |
| MIR8054 | 1.080189705 | https://www.genecards.org/cgi-bin/carddisp.pl?gene=MIR8054 |
| MIR8057 | 1.080189705 | https://www.genecards.org/cgi-bin/carddisp.pl?gene=MIR8057 |
| MIR8060 | 1.080189705 | https://www.genecards.org/cgi-bin/carddisp.pl?gene=MIR8060 |
| MIR8062 | 1.080189705 | https://www.genecards.org/cgi-bin/carddisp.pl?gene=MIR8062 |
| MIR8065 | 1.080189705 | https://www.genecards.org/cgi-bin/carddisp.pl?gene=MIR8065 |
| MIR8067 | 1.080189705 | https://www.genecards.org/cgi-bin/carddisp.pl?gene=MIR8067 |
| MIR8068 | 1.080189705 | https://www.genecards.org/cgi-bin/carddisp.pl?gene=MIR8068 |
| MIR8070 | 1.080189705 | https://www.genecards.org/cgi-bin/carddisp.pl?gene=MIR8070 |
| MIR8071-1 | 1.080189705 | https://www.genecards.org/cgi-bin/carddisp.pl?gene=MIR8071-1 |
| MIR8074 | 1.080189705 | https://www.genecards.org/cgi-bin/carddisp.pl?gene=MIR8074 |
| MIR8075 | 1.080189705 | https://www.genecards.org/cgi-bin/carddisp.pl?gene=MIR8075 |
| MIR8081 | 1.080189705 | https://www.genecards.org/cgi-bin/carddisp.pl?gene=MIR8081 |
| MIR8084 | 1.080189705 | https://www.genecards.org/cgi-bin/carddisp.pl?gene=MIR8084 |
| MIR8088 | 1.080189705 | https://www.genecards.org/cgi-bin/carddisp.pl?gene=MIR8088 |
| MIR8069 | 1.080189705 | https://www.genecards.org/cgi-bin/carddisp.pl?gene=MIR8069 |
| MIR8071-2 | 1.080189705 | https://www.genecards.org/cgi-bin/carddisp.pl?gene=MIR8071-2 |
| MIR8087 | 1.080189705 | https://www.genecards.org/cgi-bin/carddisp.pl?gene=MIR8087 |
| MIR8069-1 | 1.080189705 | https://www.genecards.org/cgi-bin/carddisp.pl?gene=MIR8069-1 |
| AMPD1 | 1.078412652 | https://www.genecards.org/cgi-bin/carddisp.pl?gene=AMPD1 |
| PLA2G6 | 1.077127934 | https://www.genecards.org/cgi-bin/carddisp.pl?gene=PLA2G6 |
| RPS19 | 1.0554142 | https://www.genecards.org/cgi-bin/carddisp.pl?gene=RPS19 |
| PKD1 | 1.0554142 | https://www.genecards.org/cgi-bin/carddisp.pl?gene=PKD1 |
| SMAD2 | 1.050443769 | https://www.genecards.org/cgi-bin/carddisp.pl?gene=SMAD2 |
| APP | 1.050443769 | https://www.genecards.org/cgi-bin/carddisp.pl?gene=APP |
| EGLN1 | 1.050443769 | https://www.genecards.org/cgi-bin/carddisp.pl?gene=EGLN1 |
| LDHA | 1.050443769 | https://www.genecards.org/cgi-bin/carddisp.pl?gene=LDHA |
| CD46 | 1.050443769 | https://www.genecards.org/cgi-bin/carddisp.pl?gene=CD46 |
| NRP1 | 1.050443769 | https://www.genecards.org/cgi-bin/carddisp.pl?gene=NRP1 |
| CD22 | 1.050443769 | https://www.genecards.org/cgi-bin/carddisp.pl?gene=CD22 |
| LCAT | 1.050443769 | https://www.genecards.org/cgi-bin/carddisp.pl?gene=LCAT |
| PLD2 | 1.050443769 | https://www.genecards.org/cgi-bin/carddisp.pl?gene=PLD2 |
| BECN1 | 1.050443769 | https://www.genecards.org/cgi-bin/carddisp.pl?gene=BECN1 |
| CDC20 | 1.050443769 | https://www.genecards.org/cgi-bin/carddisp.pl?gene=CDC20 |
| PFKFB3 | 1.050443769 | https://www.genecards.org/cgi-bin/carddisp.pl?gene=PFKFB3 |
| CCR7 | 1.050443769 | https://www.genecards.org/cgi-bin/carddisp.pl?gene=CCR7 |
| FGF5 | 1.050443769 | https://www.genecards.org/cgi-bin/carddisp.pl?gene=FGF5 |
| USP14 | 1.050443769 | https://www.genecards.org/cgi-bin/carddisp.pl?gene=USP14 |
| IGFBP4 | 1.050443769 | https://www.genecards.org/cgi-bin/carddisp.pl?gene=IGFBP4 |
| SOX6 | 1.050443769 | https://www.genecards.org/cgi-bin/carddisp.pl?gene=SOX6 |
| UCP1 | 1.050443769 | https://www.genecards.org/cgi-bin/carddisp.pl?gene=UCP1 |
| FFAR2 | 1.050443769 | https://www.genecards.org/cgi-bin/carddisp.pl?gene=FFAR2 |
| GSTA1 | 1.050443769 | https://www.genecards.org/cgi-bin/carddisp.pl?gene=GSTA1 |
| TFF1 | 1.050443769 | https://www.genecards.org/cgi-bin/carddisp.pl?gene=TFF1 |
| TFF2 | 1.050443769 | https://www.genecards.org/cgi-bin/carddisp.pl?gene=TFF2 |
| PFN2 | 1.050443769 | https://www.genecards.org/cgi-bin/carddisp.pl?gene=PFN2 |
| SERPINA12 | 1.050443769 | https://www.genecards.org/cgi-bin/carddisp.pl?gene=SERPINA12 |
| EBI3 | 1.050443769 | https://www.genecards.org/cgi-bin/carddisp.pl?gene=EBI3 |
| N4BP1 | 1.050443769 | https://www.genecards.org/cgi-bin/carddisp.pl?gene=N4BP1 |
| XCL1 | 1.050443769 | https://www.genecards.org/cgi-bin/carddisp.pl?gene=XCL1 |
| EGFL6 | 1.050443769 | https://www.genecards.org/cgi-bin/carddisp.pl?gene=EGFL6 |
| ACBD6 | 1.050443769 | https://www.genecards.org/cgi-bin/carddisp.pl?gene=ACBD6 |
| UBL4A | 1.050443769 | https://www.genecards.org/cgi-bin/carddisp.pl?gene=UBL4A |
| CRMA | 1.050443769 | https://www.genecards.org/cgi-bin/carddisp.pl?gene=CRMA |
| MIR1-1 | 1.050443769 | https://www.genecards.org/cgi-bin/carddisp.pl?gene=MIR1-1 |
| MIR128-2 | 1.050443769 | https://www.genecards.org/cgi-bin/carddisp.pl?gene=MIR128-2 |
| MIR31 | 1.050443769 | https://www.genecards.org/cgi-bin/carddisp.pl?gene=MIR31 |
| RN7SL1 | 1.050443769 | https://www.genecards.org/cgi-bin/carddisp.pl?gene=RN7SL1 |
| MIR1224 | 1.050443769 | https://www.genecards.org/cgi-bin/carddisp.pl?gene=MIR1224 |
| LUCAT1 | 1.050443769 | https://www.genecards.org/cgi-bin/carddisp.pl?gene=LUCAT1 |
| MIR1-2 | 1.050443769 | https://www.genecards.org/cgi-bin/carddisp.pl?gene=MIR1-2 |
| LUADT1 | 1.050443769 | https://www.genecards.org/cgi-bin/carddisp.pl?gene=LUADT1 |
| CD34 | 1.043177009 | https://www.genecards.org/cgi-bin/carddisp.pl?gene=CD34 |
| U2AF1 | 1.043177009 | https://www.genecards.org/cgi-bin/carddisp.pl?gene=U2AF1 |
| DDAH2 | 1.03498435 | https://www.genecards.org/cgi-bin/carddisp.pl?gene=DDAH2 |
| KLK1 | 1.032458067 | https://www.genecards.org/cgi-bin/carddisp.pl?gene=KLK1 |
| SLC26A2 | 1.032458067 | https://www.genecards.org/cgi-bin/carddisp.pl?gene=SLC26A2 |
| CELA2A | 1.032458067 | https://www.genecards.org/cgi-bin/carddisp.pl?gene=CELA2A |
| CTRL | 1.032458067 | https://www.genecards.org/cgi-bin/carddisp.pl?gene=CTRL |
| CSF3R | 1.029423475 | https://www.genecards.org/cgi-bin/carddisp.pl?gene=CSF3R |
| PDGFRB | 1.025066018 | https://www.genecards.org/cgi-bin/carddisp.pl?gene=PDGFRB |
| BCHE | 1.025066018 | https://www.genecards.org/cgi-bin/carddisp.pl?gene=BCHE |
| CDKN1A | 1.025066018 | https://www.genecards.org/cgi-bin/carddisp.pl?gene=CDKN1A |
| FYN | 1.025066018 | https://www.genecards.org/cgi-bin/carddisp.pl?gene=FYN |
| IGF2R | 1.025066018 | https://www.genecards.org/cgi-bin/carddisp.pl?gene=IGF2R |
| MASP1 | 1.022466898 | https://www.genecards.org/cgi-bin/carddisp.pl?gene=MASP1 |
| CFTR | 1.016680717 | https://www.genecards.org/cgi-bin/carddisp.pl?gene=CFTR |
| DPYD | 1.016680717 | https://www.genecards.org/cgi-bin/carddisp.pl?gene=DPYD |
| CAPN1 | 1.016680717 | https://www.genecards.org/cgi-bin/carddisp.pl?gene=CAPN1 |
| GSTP1 | 1.016680717 | https://www.genecards.org/cgi-bin/carddisp.pl?gene=GSTP1 |
| ADORA2B | 1.016680717 | https://www.genecards.org/cgi-bin/carddisp.pl?gene=ADORA2B |
| MFN2 | 1.016680717 | https://www.genecards.org/cgi-bin/carddisp.pl?gene=MFN2 |
| MPL | 1.016680717 | https://www.genecards.org/cgi-bin/carddisp.pl?gene=MPL |
| CARM1 | 1.016680717 | https://www.genecards.org/cgi-bin/carddisp.pl?gene=CARM1 |
| FOLH1 | 1.016680717 | https://www.genecards.org/cgi-bin/carddisp.pl?gene=FOLH1 |
| HPGD | 1.016680717 | https://www.genecards.org/cgi-bin/carddisp.pl?gene=HPGD |
| MSX2 | 1.016680717 | https://www.genecards.org/cgi-bin/carddisp.pl?gene=MSX2 |
| PCK2 | 1.016680717 | https://www.genecards.org/cgi-bin/carddisp.pl?gene=PCK2 |
| PTGDR | 1.016680717 | https://www.genecards.org/cgi-bin/carddisp.pl?gene=PTGDR |
| SH2B3 | 1.016680717 | https://www.genecards.org/cgi-bin/carddisp.pl?gene=SH2B3 |
| ATP2B1 | 1.016680717 | https://www.genecards.org/cgi-bin/carddisp.pl?gene=ATP2B1 |
| DRD1 | 1.016680717 | https://www.genecards.org/cgi-bin/carddisp.pl?gene=DRD1 |
| ADRB3 | 1.016680717 | https://www.genecards.org/cgi-bin/carddisp.pl?gene=ADRB3 |
| F11R | 1.016680717 | https://www.genecards.org/cgi-bin/carddisp.pl?gene=F11R |
| TANK | 1.016680717 | https://www.genecards.org/cgi-bin/carddisp.pl?gene=TANK |
| GJA4 | 1.016680717 | https://www.genecards.org/cgi-bin/carddisp.pl?gene=GJA4 |
| HSPE1 | 1.016680717 | https://www.genecards.org/cgi-bin/carddisp.pl?gene=HSPE1 |
| PAPPA | 1.016680717 | https://www.genecards.org/cgi-bin/carddisp.pl?gene=PAPPA |
| TLK1 | 1.016680717 | https://www.genecards.org/cgi-bin/carddisp.pl?gene=TLK1 |
| IL18BP | 1.016680717 | https://www.genecards.org/cgi-bin/carddisp.pl?gene=IL18BP |
| BSND | 1.016680717 | https://www.genecards.org/cgi-bin/carddisp.pl?gene=BSND |
| DIO2 | 1.016680717 | https://www.genecards.org/cgi-bin/carddisp.pl?gene=DIO2 |
| TRIM31 | 1.016680717 | https://www.genecards.org/cgi-bin/carddisp.pl?gene=TRIM31 |
| NUDT16 | 1.016680717 | https://www.genecards.org/cgi-bin/carddisp.pl?gene=NUDT16 |
| OFCC1 | 1.016680717 | https://www.genecards.org/cgi-bin/carddisp.pl?gene=OFCC1 |
| MIR626 | 1.016680717 | https://www.genecards.org/cgi-bin/carddisp.pl?gene=MIR626 |
| SNORD26 | 1.016680717 | https://www.genecards.org/cgi-bin/carddisp.pl?gene=SNORD26 |
| LINC01150 | 1.016680717 | https://www.genecards.org/cgi-bin/carddisp.pl?gene=LINC01150 |
| NKILA | 1.016680717 | https://www.genecards.org/cgi-bin/carddisp.pl?gene=NKILA |
| SNORD44 | 1.016680717 | https://www.genecards.org/cgi-bin/carddisp.pl?gene=SNORD44 |
| TTC4P1 | 1.016680717 | https://www.genecards.org/cgi-bin/carddisp.pl?gene=TTC4P1 |
| RPL4P6 | 1.016680717 | https://www.genecards.org/cgi-bin/carddisp.pl?gene=RPL4P6 |
| RPSAP56 | 1.016680717 | https://www.genecards.org/cgi-bin/carddisp.pl?gene=RPSAP56 |
| LOC101929258 | 1.016680717 | https://www.genecards.org/cgi-bin/carddisp.pl?gene=LOC101929258 |
| ALOX5 | 1.002487183 | https://www.genecards.org/cgi-bin/carddisp.pl?gene=ALOX5 |
| FYCO1 | 1.001286507 | https://www.genecards.org/cgi-bin/carddisp.pl?gene=FYCO1 |
| SNORD118 | 1.001286507 | https://www.genecards.org/cgi-bin/carddisp.pl?gene=SNORD118 |
| C3AR1 | 0.991775513 | https://www.genecards.org/cgi-bin/carddisp.pl?gene=C3AR1 |
| LINC01230 | 0.991775513 | https://www.genecards.org/cgi-bin/carddisp.pl?gene=LINC01230 |
| CYP2B6 | 0.991302848 | https://www.genecards.org/cgi-bin/carddisp.pl?gene=CYP2B6 |
| CTTN | 0.991302848 | https://www.genecards.org/cgi-bin/carddisp.pl?gene=CTTN |
| RAB1A | 0.991302848 | https://www.genecards.org/cgi-bin/carddisp.pl?gene=RAB1A |
| BCR | 0.990852356 | https://www.genecards.org/cgi-bin/carddisp.pl?gene=BCR |
| DSP | 0.990852356 | https://www.genecards.org/cgi-bin/carddisp.pl?gene=DSP |
| STAT6 | 0.990852356 | https://www.genecards.org/cgi-bin/carddisp.pl?gene=STAT6 |
| MSH2 | 0.990852356 | https://www.genecards.org/cgi-bin/carddisp.pl?gene=MSH2 |
| MSH6 | 0.990852356 | https://www.genecards.org/cgi-bin/carddisp.pl?gene=MSH6 |
| ATP7B | 0.990852356 | https://www.genecards.org/cgi-bin/carddisp.pl?gene=ATP7B |
| ALDH5A1 | 0.990852356 | https://www.genecards.org/cgi-bin/carddisp.pl?gene=ALDH5A1 |
| AP2M1 | 0.990852356 | https://www.genecards.org/cgi-bin/carddisp.pl?gene=AP2M1 |
| ATP2B3 | 0.990852356 | https://www.genecards.org/cgi-bin/carddisp.pl?gene=ATP2B3 |
| BMX | 0.990852356 | https://www.genecards.org/cgi-bin/carddisp.pl?gene=BMX |
| CASP14 | 0.990852356 | https://www.genecards.org/cgi-bin/carddisp.pl?gene=CASP14 |
| DAG1 | 0.990852356 | https://www.genecards.org/cgi-bin/carddisp.pl?gene=DAG1 |
| STS | 0.990852356 | https://www.genecards.org/cgi-bin/carddisp.pl?gene=STS |
| CLCN2 | 0.990852356 | https://www.genecards.org/cgi-bin/carddisp.pl?gene=CLCN2 |
| DVL3 | 0.990852356 | https://www.genecards.org/cgi-bin/carddisp.pl?gene=DVL3 |
| DGUOK | 0.990852356 | https://www.genecards.org/cgi-bin/carddisp.pl?gene=DGUOK |
| DUOX2 | 0.990852356 | https://www.genecards.org/cgi-bin/carddisp.pl?gene=DUOX2 |
| KRT6A | 0.990852356 | https://www.genecards.org/cgi-bin/carddisp.pl?gene=KRT6A |
| SCO2 | 0.990852356 | https://www.genecards.org/cgi-bin/carddisp.pl?gene=SCO2 |
| TRIM25 | 0.990852356 | https://www.genecards.org/cgi-bin/carddisp.pl?gene=TRIM25 |
| TXK | 0.990852356 | https://www.genecards.org/cgi-bin/carddisp.pl?gene=TXK |
| ABCC5 | 0.990852356 | https://www.genecards.org/cgi-bin/carddisp.pl?gene=ABCC5 |
| ANP32A | 0.990852356 | https://www.genecards.org/cgi-bin/carddisp.pl?gene=ANP32A |
| AUH | 0.990852356 | https://www.genecards.org/cgi-bin/carddisp.pl?gene=AUH |
| C6 | 0.990852356 | https://www.genecards.org/cgi-bin/carddisp.pl?gene=C6 |
| C7 | 0.990852356 | https://www.genecards.org/cgi-bin/carddisp.pl?gene=C7 |
| DHX9 | 0.990852356 | https://www.genecards.org/cgi-bin/carddisp.pl?gene=DHX9 |
| EHHADH | 0.990852356 | https://www.genecards.org/cgi-bin/carddisp.pl?gene=EHHADH |
| ELN | 0.990852356 | https://www.genecards.org/cgi-bin/carddisp.pl?gene=ELN |
| PAX7 | 0.990852356 | https://www.genecards.org/cgi-bin/carddisp.pl?gene=PAX7 |
| ABCA2 | 0.990852356 | https://www.genecards.org/cgi-bin/carddisp.pl?gene=ABCA2 |
| ATP11B | 0.990852356 | https://www.genecards.org/cgi-bin/carddisp.pl?gene=ATP11B |
| CDH17 | 0.990852356 | https://www.genecards.org/cgi-bin/carddisp.pl?gene=CDH17 |
| HIBCH | 0.990852356 | https://www.genecards.org/cgi-bin/carddisp.pl?gene=HIBCH |
| KDSR | 0.990852356 | https://www.genecards.org/cgi-bin/carddisp.pl?gene=KDSR |
| KRT2 | 0.990852356 | https://www.genecards.org/cgi-bin/carddisp.pl?gene=KRT2 |
| MPV17 | 0.990852356 | https://www.genecards.org/cgi-bin/carddisp.pl?gene=MPV17 |
| ACTL6A | 0.990852356 | https://www.genecards.org/cgi-bin/carddisp.pl?gene=ACTL6A |
| B3GNT5 | 0.990852356 | https://www.genecards.org/cgi-bin/carddisp.pl?gene=B3GNT5 |
| CHRD | 0.990852356 | https://www.genecards.org/cgi-bin/carddisp.pl?gene=CHRD |
| CST6 | 0.990852356 | https://www.genecards.org/cgi-bin/carddisp.pl?gene=CST6 |
| DSG4 | 0.990852356 | https://www.genecards.org/cgi-bin/carddisp.pl?gene=DSG4 |
| EIF2B5 | 0.990852356 | https://www.genecards.org/cgi-bin/carddisp.pl?gene=EIF2B5 |
| MX1 | 0.990852356 | https://www.genecards.org/cgi-bin/carddisp.pl?gene=MX1 |
| SETBP1 | 0.990852356 | https://www.genecards.org/cgi-bin/carddisp.pl?gene=SETBP1 |
| COG2 | 0.990852356 | https://www.genecards.org/cgi-bin/carddisp.pl?gene=COG2 |
| DDHD2 | 0.990852356 | https://www.genecards.org/cgi-bin/carddisp.pl?gene=DDHD2 |
| NBR1 | 0.990852356 | https://www.genecards.org/cgi-bin/carddisp.pl?gene=NBR1 |
| TIMM50 | 0.990852356 | https://www.genecards.org/cgi-bin/carddisp.pl?gene=TIMM50 |
| TMEM70 | 0.990852356 | https://www.genecards.org/cgi-bin/carddisp.pl?gene=TMEM70 |
| TMPRSS11D | 0.990852356 | https://www.genecards.org/cgi-bin/carddisp.pl?gene=TMPRSS11D |
| COG1 | 0.990852356 | https://www.genecards.org/cgi-bin/carddisp.pl?gene=COG1 |
| DCUN1D1 | 0.990852356 | https://www.genecards.org/cgi-bin/carddisp.pl?gene=DCUN1D1 |
| DDHD1 | 0.990852356 | https://www.genecards.org/cgi-bin/carddisp.pl?gene=DDHD1 |
| KPNA6 | 0.990852356 | https://www.genecards.org/cgi-bin/carddisp.pl?gene=KPNA6 |
| SH3BP5 | 0.990852356 | https://www.genecards.org/cgi-bin/carddisp.pl?gene=SH3BP5 |
| ECE2 | 0.990852356 | https://www.genecards.org/cgi-bin/carddisp.pl?gene=ECE2 |
| OPA3 | 0.990852356 | https://www.genecards.org/cgi-bin/carddisp.pl?gene=OPA3 |
| TCHH | 0.990852356 | https://www.genecards.org/cgi-bin/carddisp.pl?gene=TCHH |
| CCDC39 | 0.990852356 | https://www.genecards.org/cgi-bin/carddisp.pl?gene=CCDC39 |
| DENND2B | 0.990852356 | https://www.genecards.org/cgi-bin/carddisp.pl?gene=DENND2B |
| ABCF3 | 0.990852356 | https://www.genecards.org/cgi-bin/carddisp.pl?gene=ABCF3 |
| MAPK1IP1L | 0.990852356 | https://www.genecards.org/cgi-bin/carddisp.pl?gene=MAPK1IP1L |
| CAMK2N2 | 0.990852356 | https://www.genecards.org/cgi-bin/carddisp.pl?gene=CAMK2N2 |
| IFIT5 | 0.990852356 | https://www.genecards.org/cgi-bin/carddisp.pl?gene=IFIT5 |
| C3orf70 | 0.990852356 | https://www.genecards.org/cgi-bin/carddisp.pl?gene=C3orf70 |
| EEF1AKMT4-ECE2 | 0.990852356 | https://www.genecards.org/cgi-bin/carddisp.pl?gene=EEF1AKMT4-ECE2 |
| EEF1AKMT4 | 0.990852356 | https://www.genecards.org/cgi-bin/carddisp.pl?gene=EEF1AKMT4 |
| ABCC5-AS1 | 0.990852356 | https://www.genecards.org/cgi-bin/carddisp.pl?gene=ABCC5-AS1 |
| EHHADH-AS1 | 0.990852356 | https://www.genecards.org/cgi-bin/carddisp.pl?gene=EHHADH-AS1 |
| ATP11B-DT | 0.990852356 | https://www.genecards.org/cgi-bin/carddisp.pl?gene=ATP11B-DT |
| EIF2B5-DT | 0.990852356 | https://www.genecards.org/cgi-bin/carddisp.pl?gene=EIF2B5-DT |
| AGMX2 | 0.990852356 | https://www.genecards.org/cgi-bin/carddisp.pl?gene=AGMX2 |
| GZMK | 0.984798193 | https://www.genecards.org/cgi-bin/carddisp.pl?gene=GZMK |
| SOX9-AS1 | 0.983462989 | https://www.genecards.org/cgi-bin/carddisp.pl?gene=SOX9-AS1 |
| ROCR | 0.983462989 | https://www.genecards.org/cgi-bin/carddisp.pl?gene=ROCR |
| ENSG00000288605 | 0.983462989 | https://www.genecards.org/cgi-bin/carddisp.pl?gene=ENSG00000288605 |
| LOC102723517 | 0.983462989 | https://www.genecards.org/cgi-bin/carddisp.pl?gene=LOC102723517 |
| F2RL3 | 0.976631343 | https://www.genecards.org/cgi-bin/carddisp.pl?gene=F2RL3 |
| KCNA2 | 0.976631343 | https://www.genecards.org/cgi-bin/carddisp.pl?gene=KCNA2 |
| CBLB | 0.976631343 | https://www.genecards.org/cgi-bin/carddisp.pl?gene=CBLB |
| MSR1 | 0.976631343 | https://www.genecards.org/cgi-bin/carddisp.pl?gene=MSR1 |
| COX5A | 0.976631343 | https://www.genecards.org/cgi-bin/carddisp.pl?gene=COX5A |
| TNFSF10 | 0.976631343 | https://www.genecards.org/cgi-bin/carddisp.pl?gene=TNFSF10 |
| HNRNPD | 0.976631343 | https://www.genecards.org/cgi-bin/carddisp.pl?gene=HNRNPD |
| ROBO4 | 0.976631343 | https://www.genecards.org/cgi-bin/carddisp.pl?gene=ROBO4 |
| CD276 | 0.976631343 | https://www.genecards.org/cgi-bin/carddisp.pl?gene=CD276 |
| MLYCD | 0.976631343 | https://www.genecards.org/cgi-bin/carddisp.pl?gene=MLYCD |
| LUM | 0.976631343 | https://www.genecards.org/cgi-bin/carddisp.pl?gene=LUM |
| PBX3 | 0.976631343 | https://www.genecards.org/cgi-bin/carddisp.pl?gene=PBX3 |
| SHANK3 | 0.976631343 | https://www.genecards.org/cgi-bin/carddisp.pl?gene=SHANK3 |
| HSP90AA2P | 0.976631343 | https://www.genecards.org/cgi-bin/carddisp.pl?gene=HSP90AA2P |
| MIRLET7A1 | 0.976631343 | https://www.genecards.org/cgi-bin/carddisp.pl?gene=MIRLET7A1 |
| MIR99A | 0.976631343 | https://www.genecards.org/cgi-bin/carddisp.pl?gene=MIR99A |
| MIR99B | 0.976631343 | https://www.genecards.org/cgi-bin/carddisp.pl?gene=MIR99B |
| MIR30A | 0.976631343 | https://www.genecards.org/cgi-bin/carddisp.pl?gene=MIR30A |
| MIR1306 | 0.976631343 | https://www.genecards.org/cgi-bin/carddisp.pl?gene=MIR1306 |
| IL19 | 0.976456881 | https://www.genecards.org/cgi-bin/carddisp.pl?gene=IL19 |
| F7 | 0.974402308 | https://www.genecards.org/cgi-bin/carddisp.pl?gene=F7 |
| IL10RB | 0.972741306 | https://www.genecards.org/cgi-bin/carddisp.pl?gene=IL10RB |
| ADCY10 | 0.964880049 | https://www.genecards.org/cgi-bin/carddisp.pl?gene=ADCY10 |
| LINC00861 | 0.964880049 | https://www.genecards.org/cgi-bin/carddisp.pl?gene=LINC00861 |
| GH1 | 0.957252145 | https://www.genecards.org/cgi-bin/carddisp.pl?gene=GH1 |
| DOCK2 | 0.957140446 | https://www.genecards.org/cgi-bin/carddisp.pl?gene=DOCK2 |
| CD28 | 0.955885887 | https://www.genecards.org/cgi-bin/carddisp.pl?gene=CD28 |
| CYP2D6 | 0.955885887 | https://www.genecards.org/cgi-bin/carddisp.pl?gene=CYP2D6 |
| YARS1 | 0.949604332 | https://www.genecards.org/cgi-bin/carddisp.pl?gene=YARS1 |
| DEF6 | 0.949604332 | https://www.genecards.org/cgi-bin/carddisp.pl?gene=DEF6 |
| COX8A | 0.949604332 | https://www.genecards.org/cgi-bin/carddisp.pl?gene=COX8A |
| SASH3 | 0.949604332 | https://www.genecards.org/cgi-bin/carddisp.pl?gene=SASH3 |
| FOCAD | 0.949604332 | https://www.genecards.org/cgi-bin/carddisp.pl?gene=FOCAD |
| F13B | 0.945431471 | https://www.genecards.org/cgi-bin/carddisp.pl?gene=F13B |
| MIR365A | 0.945431471 | https://www.genecards.org/cgi-bin/carddisp.pl?gene=MIR365A |
| LINC01159 | 0.945431471 | https://www.genecards.org/cgi-bin/carddisp.pl?gene=LINC01159 |
| MIR365B | 0.945431471 | https://www.genecards.org/cgi-bin/carddisp.pl?gene=MIR365B |
| F13A1 | 0.939428687 | https://www.genecards.org/cgi-bin/carddisp.pl?gene=F13A1 |
| TNFRSF13C | 0.928934515 | https://www.genecards.org/cgi-bin/carddisp.pl?gene=TNFRSF13C |
| KRAS | 0.924438059 | https://www.genecards.org/cgi-bin/carddisp.pl?gene=KRAS |
| DNM1L | 0.924438059 | https://www.genecards.org/cgi-bin/carddisp.pl?gene=DNM1L |
| TPO | 0.924438059 | https://www.genecards.org/cgi-bin/carddisp.pl?gene=TPO |
| AQP4 | 0.924438059 | https://www.genecards.org/cgi-bin/carddisp.pl?gene=AQP4 |
| PYGM | 0.924438059 | https://www.genecards.org/cgi-bin/carddisp.pl?gene=PYGM |
| MST1 | 0.924438059 | https://www.genecards.org/cgi-bin/carddisp.pl?gene=MST1 |
| NR4A2 | 0.924438059 | https://www.genecards.org/cgi-bin/carddisp.pl?gene=NR4A2 |
| ADORA3 | 0.924438059 | https://www.genecards.org/cgi-bin/carddisp.pl?gene=ADORA3 |
| CLPP | 0.924438059 | https://www.genecards.org/cgi-bin/carddisp.pl?gene=CLPP |
| G6PC1 | 0.924438059 | https://www.genecards.org/cgi-bin/carddisp.pl?gene=G6PC1 |
| MAPKAP1 | 0.924438059 | https://www.genecards.org/cgi-bin/carddisp.pl?gene=MAPKAP1 |
| BAAT | 0.924438059 | https://www.genecards.org/cgi-bin/carddisp.pl?gene=BAAT |
| IL16 | 0.924438059 | https://www.genecards.org/cgi-bin/carddisp.pl?gene=IL16 |
| AQP8 | 0.924438059 | https://www.genecards.org/cgi-bin/carddisp.pl?gene=AQP8 |
| ADPRH | 0.924438059 | https://www.genecards.org/cgi-bin/carddisp.pl?gene=ADPRH |
| UCN2 | 0.924438059 | https://www.genecards.org/cgi-bin/carddisp.pl?gene=UCN2 |
| MIR181B1 | 0.924438059 | https://www.genecards.org/cgi-bin/carddisp.pl?gene=MIR181B1 |
| MIR181C | 0.924438059 | https://www.genecards.org/cgi-bin/carddisp.pl?gene=MIR181C |
| MIR103A1 | 0.924438059 | https://www.genecards.org/cgi-bin/carddisp.pl?gene=MIR103A1 |
| MIR129-1 | 0.924438059 | https://www.genecards.org/cgi-bin/carddisp.pl?gene=MIR129-1 |
| ZFP91-CNTF | 0.924438059 | https://www.genecards.org/cgi-bin/carddisp.pl?gene=ZFP91-CNTF |
| LINC01191 | 0.924438059 | https://www.genecards.org/cgi-bin/carddisp.pl?gene=LINC01191 |
| THORLNC | 0.924438059 | https://www.genecards.org/cgi-bin/carddisp.pl?gene=THORLNC |
| STAR | 0.924172223 | https://www.genecards.org/cgi-bin/carddisp.pl?gene=STAR |
| PKHD1 | 0.924172223 | https://www.genecards.org/cgi-bin/carddisp.pl?gene=PKHD1 |
| NUP98 | 0.923626781 | https://www.genecards.org/cgi-bin/carddisp.pl?gene=NUP98 |
| MECOM | 0.915836573 | https://www.genecards.org/cgi-bin/carddisp.pl?gene=MECOM |
| PSMA7 | 0.903417766 | https://www.genecards.org/cgi-bin/carddisp.pl?gene=PSMA7 |
| MIR151A | 0.90336436 | https://www.genecards.org/cgi-bin/carddisp.pl?gene=MIR151A |
| MIR186 | 0.90336436 | https://www.genecards.org/cgi-bin/carddisp.pl?gene=MIR186 |
| MIR25 | 0.90336436 | https://www.genecards.org/cgi-bin/carddisp.pl?gene=MIR25 |
| MIR28 | 0.90336436 | https://www.genecards.org/cgi-bin/carddisp.pl?gene=MIR28 |
| MIR340 | 0.90336436 | https://www.genecards.org/cgi-bin/carddisp.pl?gene=MIR340 |
| MIR618 | 0.90336436 | https://www.genecards.org/cgi-bin/carddisp.pl?gene=MIR618 |
| KRT18 | 0.901397109 | https://www.genecards.org/cgi-bin/carddisp.pl?gene=KRT18 |
| TBK1 | 0.899060249 | https://www.genecards.org/cgi-bin/carddisp.pl?gene=TBK1 |
| SMAD4 | 0.898928881 | https://www.genecards.org/cgi-bin/carddisp.pl?gene=SMAD4 |
| GAST | 0.880346179 | https://www.genecards.org/cgi-bin/carddisp.pl?gene=GAST |
| MIR885 | 0.880346179 | https://www.genecards.org/cgi-bin/carddisp.pl?gene=MIR885 |
| KIT | 0.863643289 | https://www.genecards.org/cgi-bin/carddisp.pl?gene=KIT |
| STAT5B | 0.863643289 | https://www.genecards.org/cgi-bin/carddisp.pl?gene=STAT5B |
| PNP | 0.863643289 | https://www.genecards.org/cgi-bin/carddisp.pl?gene=PNP |
| DES | 0.863643289 | https://www.genecards.org/cgi-bin/carddisp.pl?gene=DES |
| CLEC7A | 0.863643289 | https://www.genecards.org/cgi-bin/carddisp.pl?gene=CLEC7A |
| ADCYAP1 | 0.863643289 | https://www.genecards.org/cgi-bin/carddisp.pl?gene=ADCYAP1 |
| TMEM165 | 0.863643289 | https://www.genecards.org/cgi-bin/carddisp.pl?gene=TMEM165 |
| TSC1 | 0.863143981 | https://www.genecards.org/cgi-bin/carddisp.pl?gene=TSC1 |
| CHRM2 | 0.863143981 | https://www.genecards.org/cgi-bin/carddisp.pl?gene=CHRM2 |
| CEL | 0.863143981 | https://www.genecards.org/cgi-bin/carddisp.pl?gene=CEL |
| MAD1L1 | 0.863143981 | https://www.genecards.org/cgi-bin/carddisp.pl?gene=MAD1L1 |
| SLC11A1 | 0.863143981 | https://www.genecards.org/cgi-bin/carddisp.pl?gene=SLC11A1 |
| FOXC2 | 0.863143981 | https://www.genecards.org/cgi-bin/carddisp.pl?gene=FOXC2 |
| PLCD4 | 0.863143981 | https://www.genecards.org/cgi-bin/carddisp.pl?gene=PLCD4 |
| SETX | 0.863143981 | https://www.genecards.org/cgi-bin/carddisp.pl?gene=SETX |
| GPC5 | 0.863143981 | https://www.genecards.org/cgi-bin/carddisp.pl?gene=GPC5 |
| RALGDS | 0.863143981 | https://www.genecards.org/cgi-bin/carddisp.pl?gene=RALGDS |
| RPL7A | 0.863143981 | https://www.genecards.org/cgi-bin/carddisp.pl?gene=RPL7A |
| VIL1 | 0.863143981 | https://www.genecards.org/cgi-bin/carddisp.pl?gene=VIL1 |
| GPM6A | 0.863143981 | https://www.genecards.org/cgi-bin/carddisp.pl?gene=GPM6A |
| USP37 | 0.863143981 | https://www.genecards.org/cgi-bin/carddisp.pl?gene=USP37 |
| REXO4 | 0.863143981 | https://www.genecards.org/cgi-bin/carddisp.pl?gene=REXO4 |
| GTF3C4 | 0.863143981 | https://www.genecards.org/cgi-bin/carddisp.pl?gene=GTF3C4 |
| KIF15 | 0.863143981 | https://www.genecards.org/cgi-bin/carddisp.pl?gene=KIF15 |
| MED22 | 0.863143981 | https://www.genecards.org/cgi-bin/carddisp.pl?gene=MED22 |
| RIN3 | 0.863143981 | https://www.genecards.org/cgi-bin/carddisp.pl?gene=RIN3 |
| ZC3HC1 | 0.863143981 | https://www.genecards.org/cgi-bin/carddisp.pl?gene=ZC3HC1 |
| ZFP57 | 0.863143981 | https://www.genecards.org/cgi-bin/carddisp.pl?gene=ZFP57 |
| CNOT9 | 0.863143981 | https://www.genecards.org/cgi-bin/carddisp.pl?gene=CNOT9 |
| DDX31 | 0.863143981 | https://www.genecards.org/cgi-bin/carddisp.pl?gene=DDX31 |
| GTF3C5 | 0.863143981 | https://www.genecards.org/cgi-bin/carddisp.pl?gene=GTF3C5 |
| NAV1 | 0.863143981 | https://www.genecards.org/cgi-bin/carddisp.pl?gene=NAV1 |
| TTF1 | 0.863143981 | https://www.genecards.org/cgi-bin/carddisp.pl?gene=TTF1 |
| FOXL1 | 0.863143981 | https://www.genecards.org/cgi-bin/carddisp.pl?gene=FOXL1 |
| SLC5A12 | 0.863143981 | https://www.genecards.org/cgi-bin/carddisp.pl?gene=SLC5A12 |
| SURF6 | 0.863143981 | https://www.genecards.org/cgi-bin/carddisp.pl?gene=SURF6 |
| FIBIN | 0.863143981 | https://www.genecards.org/cgi-bin/carddisp.pl?gene=FIBIN |
| SPACA9 | 0.863143981 | https://www.genecards.org/cgi-bin/carddisp.pl?gene=SPACA9 |
| STKLD1 | 0.863143981 | https://www.genecards.org/cgi-bin/carddisp.pl?gene=STKLD1 |
| SLC35F4 | 0.863143981 | https://www.genecards.org/cgi-bin/carddisp.pl?gene=SLC35F4 |
| EEF1A1P5 | 0.863143981 | https://www.genecards.org/cgi-bin/carddisp.pl?gene=EEF1A1P5 |
| BRD3OS | 0.863143981 | https://www.genecards.org/cgi-bin/carddisp.pl?gene=BRD3OS |
| WAKMAR2 | 0.863143981 | https://www.genecards.org/cgi-bin/carddisp.pl?gene=WAKMAR2 |
| LINC01623 | 0.863143981 | https://www.genecards.org/cgi-bin/carddisp.pl?gene=LINC01623 |
| LINC00378 | 0.863143981 | https://www.genecards.org/cgi-bin/carddisp.pl?gene=LINC00378 |
| LINC00363 | 0.863143981 | https://www.genecards.org/cgi-bin/carddisp.pl?gene=LINC00363 |
| ENSG00000251391 | 0.863143981 | https://www.genecards.org/cgi-bin/carddisp.pl?gene=ENSG00000251391 |
| LOC105376306 | 0.863143981 | https://www.genecards.org/cgi-bin/carddisp.pl?gene=LOC105376306 |
| NOP56P1 | 0.863143981 | https://www.genecards.org/cgi-bin/carddisp.pl?gene=NOP56P1 |
| PSMC1P3 | 0.863143981 | https://www.genecards.org/cgi-bin/carddisp.pl?gene=PSMC1P3 |
| RBBP4P6 | 0.863143981 | https://www.genecards.org/cgi-bin/carddisp.pl?gene=RBBP4P6 |
| RN7SL366P | 0.863143981 | https://www.genecards.org/cgi-bin/carddisp.pl?gene=RN7SL366P |
| PPIAP79 | 0.863143981 | https://www.genecards.org/cgi-bin/carddisp.pl?gene=PPIAP79 |
| RFC3P1 | 0.863143981 | https://www.genecards.org/cgi-bin/carddisp.pl?gene=RFC3P1 |
| RF00017-8145 | 0.863143981 | https://www.genecards.org/cgi-bin/carddisp.pl?gene=RF00017-8145 |
| RF00017-8147 | 0.863143981 | https://www.genecards.org/cgi-bin/carddisp.pl?gene=RF00017-8147 |
| lnc-ZNF142-4-001 | 0.863143981 | https://www.genecards.org/cgi-bin/carddisp.pl?gene=lnc-ZNF142-4-001 |
| LOC107133510 | 0.859330058 | https://www.genecards.org/cgi-bin/carddisp.pl?gene=LOC107133510 |
| AGRN | 0.855616093 | https://www.genecards.org/cgi-bin/carddisp.pl?gene=AGRN |
| DLL1 | 0.855616093 | https://www.genecards.org/cgi-bin/carddisp.pl?gene=DLL1 |
| CYSLTR1 | 0.855616093 | https://www.genecards.org/cgi-bin/carddisp.pl?gene=CYSLTR1 |
| AHSP | 0.855616093 | https://www.genecards.org/cgi-bin/carddisp.pl?gene=AHSP |
| MIR3175 | 0.855616093 | https://www.genecards.org/cgi-bin/carddisp.pl?gene=MIR3175 |
| MYOD1 | 0.846735597 | https://www.genecards.org/cgi-bin/carddisp.pl?gene=MYOD1 |
| F9 | 0.845982909 | https://www.genecards.org/cgi-bin/carddisp.pl?gene=F9 |
| SST | 0.834017038 | https://www.genecards.org/cgi-bin/carddisp.pl?gene=SST |
| MT-RNR1 | 0.830238223 | https://www.genecards.org/cgi-bin/carddisp.pl?gene=MT-RNR1 |
| SUFU | 0.828723669 | https://www.genecards.org/cgi-bin/carddisp.pl?gene=SUFU |
| PYY | 0.828723669 | https://www.genecards.org/cgi-bin/carddisp.pl?gene=PYY |
| GRP | 0.828723669 | https://www.genecards.org/cgi-bin/carddisp.pl?gene=GRP |
| SCT | 0.828723669 | https://www.genecards.org/cgi-bin/carddisp.pl?gene=SCT |
| MYCNOS | 0.828723669 | https://www.genecards.org/cgi-bin/carddisp.pl?gene=MYCNOS |
| C8B | 0.824890494 | https://www.genecards.org/cgi-bin/carddisp.pl?gene=C8B |
| ZAP70 | 0.824703813 | https://www.genecards.org/cgi-bin/carddisp.pl?gene=ZAP70 |
| CORO1A | 0.824703813 | https://www.genecards.org/cgi-bin/carddisp.pl?gene=CORO1A |
| AICDA | 0.824703813 | https://www.genecards.org/cgi-bin/carddisp.pl?gene=AICDA |
| FERMT1 | 0.824703813 | https://www.genecards.org/cgi-bin/carddisp.pl?gene=FERMT1 |
| NHEJ1 | 0.824703813 | https://www.genecards.org/cgi-bin/carddisp.pl?gene=NHEJ1 |
| SLC35C1 | 0.824703813 | https://www.genecards.org/cgi-bin/carddisp.pl?gene=SLC35C1 |
| PRKDC | 0.817011833 | https://www.genecards.org/cgi-bin/carddisp.pl?gene=PRKDC |
| PIK3CG | 0.813569129 | https://www.genecards.org/cgi-bin/carddisp.pl?gene=PIK3CG |
| CKB | 0.812788785 | https://www.genecards.org/cgi-bin/carddisp.pl?gene=CKB |
| CYP3A4 | 0.803345919 | https://www.genecards.org/cgi-bin/carddisp.pl?gene=CYP3A4 |
| PAPPA-AS1 | 0.803345919 | https://www.genecards.org/cgi-bin/carddisp.pl?gene=PAPPA-AS1 |
| SQSTM1 | 0.798977792 | https://www.genecards.org/cgi-bin/carddisp.pl?gene=SQSTM1 |
| SRF | 0.798977792 | https://www.genecards.org/cgi-bin/carddisp.pl?gene=SRF |
| NBR2 | 0.798977792 | https://www.genecards.org/cgi-bin/carddisp.pl?gene=NBR2 |
| MIR182 | 0.798977792 | https://www.genecards.org/cgi-bin/carddisp.pl?gene=MIR182 |
| MIR26A1 | 0.798977792 | https://www.genecards.org/cgi-bin/carddisp.pl?gene=MIR26A1 |
| MIR26A2 | 0.798977792 | https://www.genecards.org/cgi-bin/carddisp.pl?gene=MIR26A2 |
| HAGLROS | 0.798977792 | https://www.genecards.org/cgi-bin/carddisp.pl?gene=HAGLROS |
| GCK | 0.79843235 | https://www.genecards.org/cgi-bin/carddisp.pl?gene=GCK |
| AVPR1A | 0.79843235 | https://www.genecards.org/cgi-bin/carddisp.pl?gene=AVPR1A |
| CD33 | 0.79843235 | https://www.genecards.org/cgi-bin/carddisp.pl?gene=CD33 |
| HLCS | 0.79843235 | https://www.genecards.org/cgi-bin/carddisp.pl?gene=HLCS |
| TFB1M | 0.79843235 | https://www.genecards.org/cgi-bin/carddisp.pl?gene=TFB1M |
| GYPB | 0.79843235 | https://www.genecards.org/cgi-bin/carddisp.pl?gene=GYPB |
| EIF2AK3 | 0.798166513 | https://www.genecards.org/cgi-bin/carddisp.pl?gene=EIF2AK3 |
| DDX3X | 0.792123318 | https://www.genecards.org/cgi-bin/carddisp.pl?gene=DDX3X |
| GMDS-DT | 0.792123318 | https://www.genecards.org/cgi-bin/carddisp.pl?gene=GMDS-DT |
| TPH1 | 0.789644957 | https://www.genecards.org/cgi-bin/carddisp.pl?gene=TPH1 |
| ABCA1 | 0.773599982 | https://www.genecards.org/cgi-bin/carddisp.pl?gene=ABCA1 |
| SAA4 | 0.773540378 | https://www.genecards.org/cgi-bin/carddisp.pl?gene=SAA4 |
| UCHL1 | 0.773054481 | https://www.genecards.org/cgi-bin/carddisp.pl?gene=UCHL1 |
| NKX2-1 | 0.773054481 | https://www.genecards.org/cgi-bin/carddisp.pl?gene=NKX2-1 |
| ABCB4 | 0.773054481 | https://www.genecards.org/cgi-bin/carddisp.pl?gene=ABCB4 |
| SELPLG | 0.773054481 | https://www.genecards.org/cgi-bin/carddisp.pl?gene=SELPLG |
| DNASE1L3 | 0.773054481 | https://www.genecards.org/cgi-bin/carddisp.pl?gene=DNASE1L3 |
| CGB7 | 0.773054481 | https://www.genecards.org/cgi-bin/carddisp.pl?gene=CGB7 |
| FAAH | 0.765214682 | https://www.genecards.org/cgi-bin/carddisp.pl?gene=FAAH |
| HLA-DRB4 | 0.765214682 | https://www.genecards.org/cgi-bin/carddisp.pl?gene=HLA-DRB4 |
| GABPB1-AS1 | 0.765214682 | https://www.genecards.org/cgi-bin/carddisp.pl?gene=GABPB1-AS1 |
| MIR4668 | 0.765214682 | https://www.genecards.org/cgi-bin/carddisp.pl?gene=MIR4668 |
| FERMT2 | 0.764818549 | https://www.genecards.org/cgi-bin/carddisp.pl?gene=FERMT2 |
| NR1I2 | 0.75409174 | https://www.genecards.org/cgi-bin/carddisp.pl?gene=NR1I2 |
| RDX | 0.746290505 | https://www.genecards.org/cgi-bin/carddisp.pl?gene=RDX |
| EZR | 0.746290505 | https://www.genecards.org/cgi-bin/carddisp.pl?gene=EZR |
| TNFSF9 | 0.746290505 | https://www.genecards.org/cgi-bin/carddisp.pl?gene=TNFSF9 |
| PTPN6 | 0.74420166 | https://www.genecards.org/cgi-bin/carddisp.pl?gene=PTPN6 |
| ESR1 | 0.739836812 | https://www.genecards.org/cgi-bin/carddisp.pl?gene=ESR1 |
| IFNGR1 | 0.739836812 | https://www.genecards.org/cgi-bin/carddisp.pl?gene=IFNGR1 |
| JUP | 0.739836812 | https://www.genecards.org/cgi-bin/carddisp.pl?gene=JUP |
| VCL | 0.739836812 | https://www.genecards.org/cgi-bin/carddisp.pl?gene=VCL |
| NR4A1 | 0.739836812 | https://www.genecards.org/cgi-bin/carddisp.pl?gene=NR4A1 |
| CYP2C9 | 0.739836812 | https://www.genecards.org/cgi-bin/carddisp.pl?gene=CYP2C9 |
| ACTC1 | 0.739836812 | https://www.genecards.org/cgi-bin/carddisp.pl?gene=ACTC1 |
| CYP2C19 | 0.739836812 | https://www.genecards.org/cgi-bin/carddisp.pl?gene=CYP2C19 |
| PLA2G10 | 0.739836812 | https://www.genecards.org/cgi-bin/carddisp.pl?gene=PLA2G10 |
| ALG6 | 0.739836812 | https://www.genecards.org/cgi-bin/carddisp.pl?gene=ALG6 |
| VTRNA1-1 | 0.739836812 | https://www.genecards.org/cgi-bin/carddisp.pl?gene=VTRNA1-1 |
| DBH | 0.73763752 | https://www.genecards.org/cgi-bin/carddisp.pl?gene=DBH |
| NOTCH1 | 0.73763752 | https://www.genecards.org/cgi-bin/carddisp.pl?gene=NOTCH1 |
| NPC1 | 0.73763752 | https://www.genecards.org/cgi-bin/carddisp.pl?gene=NPC1 |
| LOX | 0.73763752 | https://www.genecards.org/cgi-bin/carddisp.pl?gene=LOX |
| TYR | 0.73763752 | https://www.genecards.org/cgi-bin/carddisp.pl?gene=TYR |
| CD3D | 0.73763752 | https://www.genecards.org/cgi-bin/carddisp.pl?gene=CD3D |
| CTSA | 0.73763752 | https://www.genecards.org/cgi-bin/carddisp.pl?gene=CTSA |
| ISG15 | 0.73763752 | https://www.genecards.org/cgi-bin/carddisp.pl?gene=ISG15 |
| PRDM16 | 0.73763752 | https://www.genecards.org/cgi-bin/carddisp.pl?gene=PRDM16 |
| INVS | 0.73763752 | https://www.genecards.org/cgi-bin/carddisp.pl?gene=INVS |
| VASP | 0.73763752 | https://www.genecards.org/cgi-bin/carddisp.pl?gene=VASP |
| LAMP1 | 0.73763752 | https://www.genecards.org/cgi-bin/carddisp.pl?gene=LAMP1 |
| PAX1 | 0.73763752 | https://www.genecards.org/cgi-bin/carddisp.pl?gene=PAX1 |
| ALG9 | 0.73763752 | https://www.genecards.org/cgi-bin/carddisp.pl?gene=ALG9 |
| GFI1 | 0.73763752 | https://www.genecards.org/cgi-bin/carddisp.pl?gene=GFI1 |
| MBTPS2 | 0.73763752 | https://www.genecards.org/cgi-bin/carddisp.pl?gene=MBTPS2 |
| SUMF1 | 0.73763752 | https://www.genecards.org/cgi-bin/carddisp.pl?gene=SUMF1 |
| TCOF1 | 0.73763752 | https://www.genecards.org/cgi-bin/carddisp.pl?gene=TCOF1 |
| WASL | 0.73763752 | https://www.genecards.org/cgi-bin/carddisp.pl?gene=WASL |
| LMO2 | 0.73763752 | https://www.genecards.org/cgi-bin/carddisp.pl?gene=LMO2 |
| MKKS | 0.73763752 | https://www.genecards.org/cgi-bin/carddisp.pl?gene=MKKS |
| PIGO | 0.73763752 | https://www.genecards.org/cgi-bin/carddisp.pl?gene=PIGO |
| GTPBP1 | 0.73763752 | https://www.genecards.org/cgi-bin/carddisp.pl?gene=GTPBP1 |
| BST2 | 0.73763752 | https://www.genecards.org/cgi-bin/carddisp.pl?gene=BST2 |
| GP2 | 0.73763752 | https://www.genecards.org/cgi-bin/carddisp.pl?gene=GP2 |
| BLOC1S1 | 0.73763752 | https://www.genecards.org/cgi-bin/carddisp.pl?gene=BLOC1S1 |
| NHS | 0.73763752 | https://www.genecards.org/cgi-bin/carddisp.pl?gene=NHS |
| JAGN1 | 0.73763752 | https://www.genecards.org/cgi-bin/carddisp.pl?gene=JAGN1 |
| PPP1R12C | 0.73763752 | https://www.genecards.org/cgi-bin/carddisp.pl?gene=PPP1R12C |
| NFKBIA | 0.733643889 | https://www.genecards.org/cgi-bin/carddisp.pl?gene=NFKBIA |
| TAGAP | 0.725165367 | https://www.genecards.org/cgi-bin/carddisp.pl?gene=TAGAP |
| CEBPB | 0.722936273 | https://www.genecards.org/cgi-bin/carddisp.pl?gene=CEBPB |
| HPX | 0.717309296 | https://www.genecards.org/cgi-bin/carddisp.pl?gene=HPX |
| RBP4 | 0.705786109 | https://www.genecards.org/cgi-bin/carddisp.pl?gene=RBP4 |
| IFNA2 | 0.705786109 | https://www.genecards.org/cgi-bin/carddisp.pl?gene=IFNA2 |
| GLA | 0.699787498 | https://www.genecards.org/cgi-bin/carddisp.pl?gene=GLA |
| PRKD1 | 0.699787498 | https://www.genecards.org/cgi-bin/carddisp.pl?gene=PRKD1 |
| GALNS | 0.699787498 | https://www.genecards.org/cgi-bin/carddisp.pl?gene=GALNS |
| RYR1 | 0.699787498 | https://www.genecards.org/cgi-bin/carddisp.pl?gene=RYR1 |
| SLCO1B1 | 0.699787498 | https://www.genecards.org/cgi-bin/carddisp.pl?gene=SLCO1B1 |
| CHKA | 0.699787498 | https://www.genecards.org/cgi-bin/carddisp.pl?gene=CHKA |
| ICAM2 | 0.699787498 | https://www.genecards.org/cgi-bin/carddisp.pl?gene=ICAM2 |
| RAPGEF3 | 0.699787498 | https://www.genecards.org/cgi-bin/carddisp.pl?gene=RAPGEF3 |
| IL9 | 0.699787498 | https://www.genecards.org/cgi-bin/carddisp.pl?gene=IL9 |
| SLC37A4 | 0.699787498 | https://www.genecards.org/cgi-bin/carddisp.pl?gene=SLC37A4 |
| STING1 | 0.699787498 | https://www.genecards.org/cgi-bin/carddisp.pl?gene=STING1 |
| MEG8 | 0.699787498 | https://www.genecards.org/cgi-bin/carddisp.pl?gene=MEG8 |
| TGFBI | 0.689006448 | https://www.genecards.org/cgi-bin/carddisp.pl?gene=TGFBI |
| NINJ1 | 0.689006448 | https://www.genecards.org/cgi-bin/carddisp.pl?gene=NINJ1 |
| FGF7 | 0.687512159 | https://www.genecards.org/cgi-bin/carddisp.pl?gene=FGF7 |
| DUSP3 | 0.672972083 | https://www.genecards.org/cgi-bin/carddisp.pl?gene=DUSP3 |
| GRIN2A | 0.672160745 | https://www.genecards.org/cgi-bin/carddisp.pl?gene=GRIN2A |
| GLI3 | 0.672160745 | https://www.genecards.org/cgi-bin/carddisp.pl?gene=GLI3 |
| GFRA1 | 0.672160745 | https://www.genecards.org/cgi-bin/carddisp.pl?gene=GFRA1 |
| IHH | 0.672160745 | https://www.genecards.org/cgi-bin/carddisp.pl?gene=IHH |
| SOX10 | 0.672160745 | https://www.genecards.org/cgi-bin/carddisp.pl?gene=SOX10 |
| UGCG | 0.672160745 | https://www.genecards.org/cgi-bin/carddisp.pl?gene=UGCG |
| ATRIP | 0.672160745 | https://www.genecards.org/cgi-bin/carddisp.pl?gene=ATRIP |
| MMACHC | 0.672160745 | https://www.genecards.org/cgi-bin/carddisp.pl?gene=MMACHC |
| NRG3 | 0.672160745 | https://www.genecards.org/cgi-bin/carddisp.pl?gene=NRG3 |
| PHOX2B | 0.672160745 | https://www.genecards.org/cgi-bin/carddisp.pl?gene=PHOX2B |
| SLC39A7 | 0.672160745 | https://www.genecards.org/cgi-bin/carddisp.pl?gene=SLC39A7 |
| TBX1 | 0.672160745 | https://www.genecards.org/cgi-bin/carddisp.pl?gene=TBX1 |
| DSCAM | 0.672160745 | https://www.genecards.org/cgi-bin/carddisp.pl?gene=DSCAM |
| POLR2F | 0.672160745 | https://www.genecards.org/cgi-bin/carddisp.pl?gene=POLR2F |
| RGS6 | 0.672160745 | https://www.genecards.org/cgi-bin/carddisp.pl?gene=RGS6 |
| SRP19 | 0.672160745 | https://www.genecards.org/cgi-bin/carddisp.pl?gene=SRP19 |
| IFT56 | 0.672160745 | https://www.genecards.org/cgi-bin/carddisp.pl?gene=IFT56 |
| PIEZO2 | 0.672160745 | https://www.genecards.org/cgi-bin/carddisp.pl?gene=PIEZO2 |
| PROKR1 | 0.672160745 | https://www.genecards.org/cgi-bin/carddisp.pl?gene=PROKR1 |
| SRPRA | 0.672160745 | https://www.genecards.org/cgi-bin/carddisp.pl?gene=SRPRA |
| AP4E1 | 0.672160745 | https://www.genecards.org/cgi-bin/carddisp.pl?gene=AP4E1 |
| DOP1A | 0.672160745 | https://www.genecards.org/cgi-bin/carddisp.pl?gene=DOP1A |
| EEF2KMT | 0.672160745 | https://www.genecards.org/cgi-bin/carddisp.pl?gene=EEF2KMT |
| MIR31HG | 0.672160745 | https://www.genecards.org/cgi-bin/carddisp.pl?gene=MIR31HG |
| IGK | 0.672160745 | https://www.genecards.org/cgi-bin/carddisp.pl?gene=IGK |
| LINC01844 | 0.672160745 | https://www.genecards.org/cgi-bin/carddisp.pl?gene=LINC01844 |
| NCF4-AS1 | 0.672160745 | https://www.genecards.org/cgi-bin/carddisp.pl?gene=NCF4-AS1 |
| MIR4260 | 0.672160745 | https://www.genecards.org/cgi-bin/carddisp.pl?gene=MIR4260 |
| LOC101928371 | 0.672160745 | https://www.genecards.org/cgi-bin/carddisp.pl?gene=LOC101928371 |
| MTRNR2L12 | 0.672160745 | https://www.genecards.org/cgi-bin/carddisp.pl?gene=MTRNR2L12 |
| ATRIP-TREX1 | 0.672160745 | https://www.genecards.org/cgi-bin/carddisp.pl?gene=ATRIP-TREX1 |
| LOC106029312 | 0.672160745 | https://www.genecards.org/cgi-bin/carddisp.pl?gene=LOC106029312 |
| LOC106780800 | 0.672160745 | https://www.genecards.org/cgi-bin/carddisp.pl?gene=LOC106780800 |
| LOC110121502 | 0.672160745 | https://www.genecards.org/cgi-bin/carddisp.pl?gene=LOC110121502 |
| MCS+9.7 | 0.672160745 | https://www.genecards.org/cgi-bin/carddisp.pl?gene=MCS%2b9.7 |
| LOC126805948 | 0.672160745 | https://www.genecards.org/cgi-bin/carddisp.pl?gene=LOC126805948 |
| LOC126859690 | 0.672160745 | https://www.genecards.org/cgi-bin/carddisp.pl?gene=LOC126859690 |
| LOC126861525 | 0.672160745 | https://www.genecards.org/cgi-bin/carddisp.pl?gene=LOC126861525 |
| LOC126861526 | 0.672160745 | https://www.genecards.org/cgi-bin/carddisp.pl?gene=LOC126861526 |
| LOC126862707 | 0.672160745 | https://www.genecards.org/cgi-bin/carddisp.pl?gene=LOC126862707 |
| LOC126863274 | 0.672160745 | https://www.genecards.org/cgi-bin/carddisp.pl?gene=LOC126863274 |
| GSTM1 | 0.664276361 | https://www.genecards.org/cgi-bin/carddisp.pl?gene=GSTM1 |
| LILRB2 | 0.664276361 | https://www.genecards.org/cgi-bin/carddisp.pl?gene=LILRB2 |
| ABO | 0.664276361 | https://www.genecards.org/cgi-bin/carddisp.pl?gene=ABO |
| LOC106099062 | 0.664276361 | https://www.genecards.org/cgi-bin/carddisp.pl?gene=LOC106099062 |
| PTPRN | 0.663639247 | https://www.genecards.org/cgi-bin/carddisp.pl?gene=PTPRN |
| CD27-AS1 | 0.653488457 | https://www.genecards.org/cgi-bin/carddisp.pl?gene=CD27-AS1 |
| CDC42 | 0.647594213 | https://www.genecards.org/cgi-bin/carddisp.pl?gene=CDC42 |
| ENTPD1 | 0.647594213 | https://www.genecards.org/cgi-bin/carddisp.pl?gene=ENTPD1 |
| TH | 0.647594213 | https://www.genecards.org/cgi-bin/carddisp.pl?gene=TH |
| HNF4A | 0.647594213 | https://www.genecards.org/cgi-bin/carddisp.pl?gene=HNF4A |
| NTRK1 | 0.647594213 | https://www.genecards.org/cgi-bin/carddisp.pl?gene=NTRK1 |
| HCK | 0.647594213 | https://www.genecards.org/cgi-bin/carddisp.pl?gene=HCK |
| NCAM1 | 0.647594213 | https://www.genecards.org/cgi-bin/carddisp.pl?gene=NCAM1 |
| VKORC1 | 0.647594213 | https://www.genecards.org/cgi-bin/carddisp.pl?gene=VKORC1 |
| MECP2 | 0.647594213 | https://www.genecards.org/cgi-bin/carddisp.pl?gene=MECP2 |
| ACHE | 0.647594213 | https://www.genecards.org/cgi-bin/carddisp.pl?gene=ACHE |
| HTR3A | 0.647594213 | https://www.genecards.org/cgi-bin/carddisp.pl?gene=HTR3A |
| PXN | 0.647594213 | https://www.genecards.org/cgi-bin/carddisp.pl?gene=PXN |
| SOX9 | 0.647594213 | https://www.genecards.org/cgi-bin/carddisp.pl?gene=SOX9 |
| VIPR1 | 0.647594213 | https://www.genecards.org/cgi-bin/carddisp.pl?gene=VIPR1 |
| HTRA1 | 0.647594213 | https://www.genecards.org/cgi-bin/carddisp.pl?gene=HTRA1 |
| ELP1 | 0.647594213 | https://www.genecards.org/cgi-bin/carddisp.pl?gene=ELP1 |
| SUOX | 0.647594213 | https://www.genecards.org/cgi-bin/carddisp.pl?gene=SUOX |
| NOX1 | 0.647594213 | https://www.genecards.org/cgi-bin/carddisp.pl?gene=NOX1 |
| CD52 | 0.647594213 | https://www.genecards.org/cgi-bin/carddisp.pl?gene=CD52 |
| RHOD | 0.647594213 | https://www.genecards.org/cgi-bin/carddisp.pl?gene=RHOD |
| PGAM4 | 0.647594213 | https://www.genecards.org/cgi-bin/carddisp.pl?gene=PGAM4 |
| TERC | 0.647594213 | https://www.genecards.org/cgi-bin/carddisp.pl?gene=TERC |
| PPBP | 0.647462845 | https://www.genecards.org/cgi-bin/carddisp.pl?gene=PPBP |
| PLA2G2D | 0.647462845 | https://www.genecards.org/cgi-bin/carddisp.pl?gene=PLA2G2D |
| MYOG | 0.647462845 | https://www.genecards.org/cgi-bin/carddisp.pl?gene=MYOG |
| AP3B1 | 0.63881284 | https://www.genecards.org/cgi-bin/carddisp.pl?gene=AP3B1 |
| RASGRP2 | 0.63881284 | https://www.genecards.org/cgi-bin/carddisp.pl?gene=RASGRP2 |
| TLN1 | 0.63881284 | https://www.genecards.org/cgi-bin/carddisp.pl?gene=TLN1 |
| XRCC4 | 0.63881284 | https://www.genecards.org/cgi-bin/carddisp.pl?gene=XRCC4 |
| MAGT1 | 0.63881284 | https://www.genecards.org/cgi-bin/carddisp.pl?gene=MAGT1 |
| APBB1IP | 0.63881284 | https://www.genecards.org/cgi-bin/carddisp.pl?gene=APBB1IP |
| ITGAD | 0.63881284 | https://www.genecards.org/cgi-bin/carddisp.pl?gene=ITGAD |
| IGHV4-38-2 | 0.63881284 | https://www.genecards.org/cgi-bin/carddisp.pl?gene=IGHV4-38-2 |
| RIPK1 | 0.637384057 | https://www.genecards.org/cgi-bin/carddisp.pl?gene=RIPK1 |
| TNFAIP3 | 0.637384057 | https://www.genecards.org/cgi-bin/carddisp.pl?gene=TNFAIP3 |
| KLF6 | 0.637384057 | https://www.genecards.org/cgi-bin/carddisp.pl?gene=KLF6 |
| PPIC | 0.637384057 | https://www.genecards.org/cgi-bin/carddisp.pl?gene=PPIC |
| FUT4 | 0.637384057 | https://www.genecards.org/cgi-bin/carddisp.pl?gene=FUT4 |
| CALR | 0.629483223 | https://www.genecards.org/cgi-bin/carddisp.pl?gene=CALR |
| F11 | 0.629483223 | https://www.genecards.org/cgi-bin/carddisp.pl?gene=F11 |
| TGIF1 | 0.629483223 | https://www.genecards.org/cgi-bin/carddisp.pl?gene=TGIF1 |
| ALPP | 0.629483223 | https://www.genecards.org/cgi-bin/carddisp.pl?gene=ALPP |
| BAD | 0.607864022 | https://www.genecards.org/cgi-bin/carddisp.pl?gene=BAD |
| IL23A | 0.607864022 | https://www.genecards.org/cgi-bin/carddisp.pl?gene=IL23A |
| RBM3 | 0.607864022 | https://www.genecards.org/cgi-bin/carddisp.pl?gene=RBM3 |
| NQO1 | 0.607638121 | https://www.genecards.org/cgi-bin/carddisp.pl?gene=NQO1 |
| MVK | 0.607638121 | https://www.genecards.org/cgi-bin/carddisp.pl?gene=MVK |
| APOH | 0.607638121 | https://www.genecards.org/cgi-bin/carddisp.pl?gene=APOH |
| EPRS1 | 0.607638121 | https://www.genecards.org/cgi-bin/carddisp.pl?gene=EPRS1 |
| RBM4 | 0.607638121 | https://www.genecards.org/cgi-bin/carddisp.pl?gene=RBM4 |
| DYNC2I1 | 0.607638121 | https://www.genecards.org/cgi-bin/carddisp.pl?gene=DYNC2I1 |
| MIR24-1 | 0.607638121 | https://www.genecards.org/cgi-bin/carddisp.pl?gene=MIR24-1 |
| LOC110006319 | 0.607638121 | https://www.genecards.org/cgi-bin/carddisp.pl?gene=LOC110006319 |
| GATA3 | 0.595269561 | https://www.genecards.org/cgi-bin/carddisp.pl?gene=GATA3 |
| HMGCR | 0.595269561 | https://www.genecards.org/cgi-bin/carddisp.pl?gene=HMGCR |
| APEH | 0.595269561 | https://www.genecards.org/cgi-bin/carddisp.pl?gene=APEH |
| MAPK10 | 0.584845841 | https://www.genecards.org/cgi-bin/carddisp.pl?gene=MAPK10 |
| H6PD | 0.584845841 | https://www.genecards.org/cgi-bin/carddisp.pl?gene=H6PD |
| P2RY11 | 0.584845841 | https://www.genecards.org/cgi-bin/carddisp.pl?gene=P2RY11 |
| CD226 | 0.584845841 | https://www.genecards.org/cgi-bin/carddisp.pl?gene=CD226 |
| C4BPA | 0.584845841 | https://www.genecards.org/cgi-bin/carddisp.pl?gene=C4BPA |
| MARCO | 0.584845841 | https://www.genecards.org/cgi-bin/carddisp.pl?gene=MARCO |
| BOK | 0.584845841 | https://www.genecards.org/cgi-bin/carddisp.pl?gene=BOK |
| TIGIT | 0.584845841 | https://www.genecards.org/cgi-bin/carddisp.pl?gene=TIGIT |
| CRISPLD2 | 0.584845841 | https://www.genecards.org/cgi-bin/carddisp.pl?gene=CRISPLD2 |
| F12 | 0.57387495 | https://www.genecards.org/cgi-bin/carddisp.pl?gene=F12 |
| BIRC3 | 0.57387495 | https://www.genecards.org/cgi-bin/carddisp.pl?gene=BIRC3 |
| NPHS1 | 0.57387495 | https://www.genecards.org/cgi-bin/carddisp.pl?gene=NPHS1 |
| TAT | 0.57387495 | https://www.genecards.org/cgi-bin/carddisp.pl?gene=TAT |
| HAVCR1 | 0.57387495 | https://www.genecards.org/cgi-bin/carddisp.pl?gene=HAVCR1 |
| HBA1 | 0.57387495 | https://www.genecards.org/cgi-bin/carddisp.pl?gene=HBA1 |
| DDT | 0.57387495 | https://www.genecards.org/cgi-bin/carddisp.pl?gene=DDT |
| SCARNA5 | 0.57387495 | https://www.genecards.org/cgi-bin/carddisp.pl?gene=SCARNA5 |
| FST | 0.561557651 | https://www.genecards.org/cgi-bin/carddisp.pl?gene=FST |
| ACVR1B | 0.560115755 | https://www.genecards.org/cgi-bin/carddisp.pl?gene=ACVR1B |
| GPX1 | 0.560115755 | https://www.genecards.org/cgi-bin/carddisp.pl?gene=GPX1 |
| HRH4 | 0.560115755 | https://www.genecards.org/cgi-bin/carddisp.pl?gene=HRH4 |
| NPPC | 0.560115755 | https://www.genecards.org/cgi-bin/carddisp.pl?gene=NPPC |
| TNFSF12 | 0.560115755 | https://www.genecards.org/cgi-bin/carddisp.pl?gene=TNFSF12 |
| SH2D1A | 0.547017813 | https://www.genecards.org/cgi-bin/carddisp.pl?gene=SH2D1A |
| PSME3 | 0.546966314 | https://www.genecards.org/cgi-bin/carddisp.pl?gene=PSME3 |
| ZNF22-AS1 | 0.546966314 | https://www.genecards.org/cgi-bin/carddisp.pl?gene=ZNF22-AS1 |
| SPEN-AS1 | 0.546966314 | https://www.genecards.org/cgi-bin/carddisp.pl?gene=SPEN-AS1 |
| TTTY12 | 0.546966314 | https://www.genecards.org/cgi-bin/carddisp.pl?gene=TTTY12 |
| GSR | 0.533825636 | https://www.genecards.org/cgi-bin/carddisp.pl?gene=GSR |
| PPM1D | 0.533825636 | https://www.genecards.org/cgi-bin/carddisp.pl?gene=PPM1D |
| BCS1L | 0.533825636 | https://www.genecards.org/cgi-bin/carddisp.pl?gene=BCS1L |
| PON3 | 0.533825636 | https://www.genecards.org/cgi-bin/carddisp.pl?gene=PON3 |
| VTN | 0.533825636 | https://www.genecards.org/cgi-bin/carddisp.pl?gene=VTN |
| PON2 | 0.533825636 | https://www.genecards.org/cgi-bin/carddisp.pl?gene=PON2 |
| CALCB | 0.533825636 | https://www.genecards.org/cgi-bin/carddisp.pl?gene=CALCB |
| TNIP3 | 0.533825636 | https://www.genecards.org/cgi-bin/carddisp.pl?gene=TNIP3 |
| MIR210HG | 0.533825636 | https://www.genecards.org/cgi-bin/carddisp.pl?gene=MIR210HG |
| LINC00887 | 0.533825636 | https://www.genecards.org/cgi-bin/carddisp.pl?gene=LINC00887 |
| LDLR | 0.533223391 | https://www.genecards.org/cgi-bin/carddisp.pl?gene=LDLR |
| CASP9 | 0.533223391 | https://www.genecards.org/cgi-bin/carddisp.pl?gene=CASP9 |
| IRS1 | 0.533223391 | https://www.genecards.org/cgi-bin/carddisp.pl?gene=IRS1 |
| PDE2A | 0.533223391 | https://www.genecards.org/cgi-bin/carddisp.pl?gene=PDE2A |
| NR1I3 | 0.533223391 | https://www.genecards.org/cgi-bin/carddisp.pl?gene=NR1I3 |
| HIPK1 | 0.533223391 | https://www.genecards.org/cgi-bin/carddisp.pl?gene=HIPK1 |
| HOXA5 | 0.533223391 | https://www.genecards.org/cgi-bin/carddisp.pl?gene=HOXA5 |
| ARFGEF1 | 0.533223391 | https://www.genecards.org/cgi-bin/carddisp.pl?gene=ARFGEF1 |
| AKT3 | 0.521588504 | https://www.genecards.org/cgi-bin/carddisp.pl?gene=AKT3 |
| CCND1 | 0.521588504 | https://www.genecards.org/cgi-bin/carddisp.pl?gene=CCND1 |
| CTSD | 0.521588504 | https://www.genecards.org/cgi-bin/carddisp.pl?gene=CTSD |
| NTRK3 | 0.521588504 | https://www.genecards.org/cgi-bin/carddisp.pl?gene=NTRK3 |
| PDGFRA | 0.521588504 | https://www.genecards.org/cgi-bin/carddisp.pl?gene=PDGFRA |
| LCK | 0.521588504 | https://www.genecards.org/cgi-bin/carddisp.pl?gene=LCK |
| CASR | 0.521588504 | https://www.genecards.org/cgi-bin/carddisp.pl?gene=CASR |
| CBL | 0.521588504 | https://www.genecards.org/cgi-bin/carddisp.pl?gene=CBL |
| CDH2 | 0.521588504 | https://www.genecards.org/cgi-bin/carddisp.pl?gene=CDH2 |
| NGF | 0.521588504 | https://www.genecards.org/cgi-bin/carddisp.pl?gene=NGF |
| NOTCH3 | 0.521588504 | https://www.genecards.org/cgi-bin/carddisp.pl?gene=NOTCH3 |
| PTCH1 | 0.521588504 | https://www.genecards.org/cgi-bin/carddisp.pl?gene=PTCH1 |
| TERT | 0.521588504 | https://www.genecards.org/cgi-bin/carddisp.pl?gene=TERT |
| ALK | 0.521588504 | https://www.genecards.org/cgi-bin/carddisp.pl?gene=ALK |
| FANCA | 0.521588504 | https://www.genecards.org/cgi-bin/carddisp.pl?gene=FANCA |
| GAPDH | 0.521588504 | https://www.genecards.org/cgi-bin/carddisp.pl?gene=GAPDH |
| SHH | 0.521588504 | https://www.genecards.org/cgi-bin/carddisp.pl?gene=SHH |
| ALDH1A2 | 0.521588504 | https://www.genecards.org/cgi-bin/carddisp.pl?gene=ALDH1A2 |
| BMP4 | 0.521588504 | https://www.genecards.org/cgi-bin/carddisp.pl?gene=BMP4 |
| CSNK1A1 | 0.521588504 | https://www.genecards.org/cgi-bin/carddisp.pl?gene=CSNK1A1 |
| DRD2 | 0.521588504 | https://www.genecards.org/cgi-bin/carddisp.pl?gene=DRD2 |
| GLB1 | 0.521588504 | https://www.genecards.org/cgi-bin/carddisp.pl?gene=GLB1 |
| PGK1 | 0.521588504 | https://www.genecards.org/cgi-bin/carddisp.pl?gene=PGK1 |
| PMS2 | 0.521588504 | https://www.genecards.org/cgi-bin/carddisp.pl?gene=PMS2 |
| PRDX1 | 0.521588504 | https://www.genecards.org/cgi-bin/carddisp.pl?gene=PRDX1 |
| RAB7A | 0.521588504 | https://www.genecards.org/cgi-bin/carddisp.pl?gene=RAB7A |
| ATP1A2 | 0.521588504 | https://www.genecards.org/cgi-bin/carddisp.pl?gene=ATP1A2 |
| CCND2 | 0.521588504 | https://www.genecards.org/cgi-bin/carddisp.pl?gene=CCND2 |
| CHAT | 0.521588504 | https://www.genecards.org/cgi-bin/carddisp.pl?gene=CHAT |
| COL4A1 | 0.521588504 | https://www.genecards.org/cgi-bin/carddisp.pl?gene=COL4A1 |
| HEXB | 0.521588504 | https://www.genecards.org/cgi-bin/carddisp.pl?gene=HEXB |
| IDS | 0.521588504 | https://www.genecards.org/cgi-bin/carddisp.pl?gene=IDS |
| L1CAM | 0.521588504 | https://www.genecards.org/cgi-bin/carddisp.pl?gene=L1CAM |
| MITF | 0.521588504 | https://www.genecards.org/cgi-bin/carddisp.pl?gene=MITF |
| PAX6 | 0.521588504 | https://www.genecards.org/cgi-bin/carddisp.pl?gene=PAX6 |
| PKD2 | 0.521588504 | https://www.genecards.org/cgi-bin/carddisp.pl?gene=PKD2 |
| PLCG1 | 0.521588504 | https://www.genecards.org/cgi-bin/carddisp.pl?gene=PLCG1 |
| RPL11 | 0.521588504 | https://www.genecards.org/cgi-bin/carddisp.pl?gene=RPL11 |
| RPL5 | 0.521588504 | https://www.genecards.org/cgi-bin/carddisp.pl?gene=RPL5 |
| SLC11A2 | 0.521588504 | https://www.genecards.org/cgi-bin/carddisp.pl?gene=SLC11A2 |
| ST14 | 0.521588504 | https://www.genecards.org/cgi-bin/carddisp.pl?gene=ST14 |
| STX1A | 0.521588504 | https://www.genecards.org/cgi-bin/carddisp.pl?gene=STX1A |
| TFAP2A | 0.521588504 | https://www.genecards.org/cgi-bin/carddisp.pl?gene=TFAP2A |
| VRK1 | 0.521588504 | https://www.genecards.org/cgi-bin/carddisp.pl?gene=VRK1 |
| ABCC6 | 0.521588504 | https://www.genecards.org/cgi-bin/carddisp.pl?gene=ABCC6 |
| APRT | 0.521588504 | https://www.genecards.org/cgi-bin/carddisp.pl?gene=APRT |
| ARSA | 0.521588504 | https://www.genecards.org/cgi-bin/carddisp.pl?gene=ARSA |
| ARSB | 0.521588504 | https://www.genecards.org/cgi-bin/carddisp.pl?gene=ARSB |
| AXIN1 | 0.521588504 | https://www.genecards.org/cgi-bin/carddisp.pl?gene=AXIN1 |
| CD3G | 0.521588504 | https://www.genecards.org/cgi-bin/carddisp.pl?gene=CD3G |
| COL4A2 | 0.521588504 | https://www.genecards.org/cgi-bin/carddisp.pl?gene=COL4A2 |
| CTNNA1 | 0.521588504 | https://www.genecards.org/cgi-bin/carddisp.pl?gene=CTNNA1 |
| CTSC | 0.521588504 | https://www.genecards.org/cgi-bin/carddisp.pl?gene=CTSC |
| GATA1 | 0.521588504 | https://www.genecards.org/cgi-bin/carddisp.pl?gene=GATA1 |
| HEXA | 0.521588504 | https://www.genecards.org/cgi-bin/carddisp.pl?gene=HEXA |
| HTR2A | 0.521588504 | https://www.genecards.org/cgi-bin/carddisp.pl?gene=HTR2A |
| HYAL1 | 0.521588504 | https://www.genecards.org/cgi-bin/carddisp.pl?gene=HYAL1 |
| NEDD4 | 0.521588504 | https://www.genecards.org/cgi-bin/carddisp.pl?gene=NEDD4 |
| NGFR | 0.521588504 | https://www.genecards.org/cgi-bin/carddisp.pl?gene=NGFR |
| NOG | 0.521588504 | https://www.genecards.org/cgi-bin/carddisp.pl?gene=NOG |
| PPP1CA | 0.521588504 | https://www.genecards.org/cgi-bin/carddisp.pl?gene=PPP1CA |
| TPI1 | 0.521588504 | https://www.genecards.org/cgi-bin/carddisp.pl?gene=TPI1 |
| VCAN | 0.521588504 | https://www.genecards.org/cgi-bin/carddisp.pl?gene=VCAN |
| WNT1 | 0.521588504 | https://www.genecards.org/cgi-bin/carddisp.pl?gene=WNT1 |
| ZEB2 | 0.521588504 | https://www.genecards.org/cgi-bin/carddisp.pl?gene=ZEB2 |
| AK1 | 0.521588504 | https://www.genecards.org/cgi-bin/carddisp.pl?gene=AK1 |
| ALDH3A2 | 0.521588504 | https://www.genecards.org/cgi-bin/carddisp.pl?gene=ALDH3A2 |
| ANTXR1 | 0.521588504 | https://www.genecards.org/cgi-bin/carddisp.pl?gene=ANTXR1 |
| ANTXR2 | 0.521588504 | https://www.genecards.org/cgi-bin/carddisp.pl?gene=ANTXR2 |
| ARF1 | 0.521588504 | https://www.genecards.org/cgi-bin/carddisp.pl?gene=ARF1 |
| BMP2 | 0.521588504 | https://www.genecards.org/cgi-bin/carddisp.pl?gene=BMP2 |
| CANX | 0.521588504 | https://www.genecards.org/cgi-bin/carddisp.pl?gene=CANX |
| CEBPA | 0.521588504 | https://www.genecards.org/cgi-bin/carddisp.pl?gene=CEBPA |
| CRKL | 0.521588504 | https://www.genecards.org/cgi-bin/carddisp.pl?gene=CRKL |
| ERCC6 | 0.521588504 | https://www.genecards.org/cgi-bin/carddisp.pl?gene=ERCC6 |
| GRB2 | 0.521588504 | https://www.genecards.org/cgi-bin/carddisp.pl?gene=GRB2 |
| GRK6 | 0.521588504 | https://www.genecards.org/cgi-bin/carddisp.pl?gene=GRK6 |
| HTR1A | 0.521588504 | https://www.genecards.org/cgi-bin/carddisp.pl?gene=HTR1A |
| IDUA | 0.521588504 | https://www.genecards.org/cgi-bin/carddisp.pl?gene=IDUA |
| LAMA4 | 0.521588504 | https://www.genecards.org/cgi-bin/carddisp.pl?gene=LAMA4 |
| METAP2 | 0.521588504 | https://www.genecards.org/cgi-bin/carddisp.pl?gene=METAP2 |
| MPZ | 0.521588504 | https://www.genecards.org/cgi-bin/carddisp.pl?gene=MPZ |
| PAX3 | 0.521588504 | https://www.genecards.org/cgi-bin/carddisp.pl?gene=PAX3 |
| PTGER3 | 0.521588504 | https://www.genecards.org/cgi-bin/carddisp.pl?gene=PTGER3 |
| PTS | 0.521588504 | https://www.genecards.org/cgi-bin/carddisp.pl?gene=PTS |
| SCO1 | 0.521588504 | https://www.genecards.org/cgi-bin/carddisp.pl?gene=SCO1 |
| SEMA3A | 0.521588504 | https://www.genecards.org/cgi-bin/carddisp.pl?gene=SEMA3A |
| SGSH | 0.521588504 | https://www.genecards.org/cgi-bin/carddisp.pl?gene=SGSH |
| SOX2 | 0.521588504 | https://www.genecards.org/cgi-bin/carddisp.pl?gene=SOX2 |
| SYP | 0.521588504 | https://www.genecards.org/cgi-bin/carddisp.pl?gene=SYP |
| TBX2 | 0.521588504 | https://www.genecards.org/cgi-bin/carddisp.pl?gene=TBX2 |
| ALOX12 | 0.521588504 | https://www.genecards.org/cgi-bin/carddisp.pl?gene=ALOX12 |
| ARRB2 | 0.521588504 | https://www.genecards.org/cgi-bin/carddisp.pl?gene=ARRB2 |
| DCT | 0.521588504 | https://www.genecards.org/cgi-bin/carddisp.pl?gene=DCT |
| ELOVL4 | 0.521588504 | https://www.genecards.org/cgi-bin/carddisp.pl?gene=ELOVL4 |
| EPHX1 | 0.521588504 | https://www.genecards.org/cgi-bin/carddisp.pl?gene=EPHX1 |
| GALC | 0.521588504 | https://www.genecards.org/cgi-bin/carddisp.pl?gene=GALC |
| GRK3 | 0.521588504 | https://www.genecards.org/cgi-bin/carddisp.pl?gene=GRK3 |
| MUTYH | 0.521588504 | https://www.genecards.org/cgi-bin/carddisp.pl?gene=MUTYH |
| NRG2 | 0.521588504 | https://www.genecards.org/cgi-bin/carddisp.pl?gene=NRG2 |
| PEX1 | 0.521588504 | https://www.genecards.org/cgi-bin/carddisp.pl?gene=PEX1 |
| PRKCSH | 0.521588504 | https://www.genecards.org/cgi-bin/carddisp.pl?gene=PRKCSH |
| PRSS8 | 0.521588504 | https://www.genecards.org/cgi-bin/carddisp.pl?gene=PRSS8 |
| PTH | 0.521588504 | https://www.genecards.org/cgi-bin/carddisp.pl?gene=PTH |
| SAG | 0.521588504 | https://www.genecards.org/cgi-bin/carddisp.pl?gene=SAG |
| TNFRSF10A | 0.521588504 | https://www.genecards.org/cgi-bin/carddisp.pl?gene=TNFRSF10A |
| TPP1 | 0.521588504 | https://www.genecards.org/cgi-bin/carddisp.pl?gene=TPP1 |
| VAV1 | 0.521588504 | https://www.genecards.org/cgi-bin/carddisp.pl?gene=VAV1 |
| ABCA4 | 0.521588504 | https://www.genecards.org/cgi-bin/carddisp.pl?gene=ABCA4 |
| ABCA7 | 0.521588504 | https://www.genecards.org/cgi-bin/carddisp.pl?gene=ABCA7 |
| ARRB1 | 0.521588504 | https://www.genecards.org/cgi-bin/carddisp.pl?gene=ARRB1 |
| CLN3 | 0.521588504 | https://www.genecards.org/cgi-bin/carddisp.pl?gene=CLN3 |
| COL17A1 | 0.521588504 | https://www.genecards.org/cgi-bin/carddisp.pl?gene=COL17A1 |
| EEF1A1 | 0.521588504 | https://www.genecards.org/cgi-bin/carddisp.pl?gene=EEF1A1 |
| EFNA5 | 0.521588504 | https://www.genecards.org/cgi-bin/carddisp.pl?gene=EFNA5 |
| EGR2 | 0.521588504 | https://www.genecards.org/cgi-bin/carddisp.pl?gene=EGR2 |
| EPS15 | 0.521588504 | https://www.genecards.org/cgi-bin/carddisp.pl?gene=EPS15 |
| EXTL3 | 0.521588504 | https://www.genecards.org/cgi-bin/carddisp.pl?gene=EXTL3 |
| FANCG | 0.521588504 | https://www.genecards.org/cgi-bin/carddisp.pl?gene=FANCG |
| FOXP1 | 0.521588504 | https://www.genecards.org/cgi-bin/carddisp.pl?gene=FOXP1 |
| FOXP2 | 0.521588504 | https://www.genecards.org/cgi-bin/carddisp.pl?gene=FOXP2 |
| GAL | 0.521588504 | https://www.genecards.org/cgi-bin/carddisp.pl?gene=GAL |
| GFRA2 | 0.521588504 | https://www.genecards.org/cgi-bin/carddisp.pl?gene=GFRA2 |
| GNS | 0.521588504 | https://www.genecards.org/cgi-bin/carddisp.pl?gene=GNS |
| HLA-C | 0.521588504 | https://www.genecards.org/cgi-bin/carddisp.pl?gene=HLA-C |
| IFNGR2 | 0.521588504 | https://www.genecards.org/cgi-bin/carddisp.pl?gene=IFNGR2 |
| MBTPS1 | 0.521588504 | https://www.genecards.org/cgi-bin/carddisp.pl?gene=MBTPS1 |
| MKI67 | 0.521588504 | https://www.genecards.org/cgi-bin/carddisp.pl?gene=MKI67 |
| MPI | 0.521588504 | https://www.genecards.org/cgi-bin/carddisp.pl?gene=MPI |
| MSX1 | 0.521588504 | https://www.genecards.org/cgi-bin/carddisp.pl?gene=MSX1 |
| NHP2 | 0.521588504 | https://www.genecards.org/cgi-bin/carddisp.pl?gene=NHP2 |
| NTF3 | 0.521588504 | https://www.genecards.org/cgi-bin/carddisp.pl?gene=NTF3 |
| OCRL | 0.521588504 | https://www.genecards.org/cgi-bin/carddisp.pl?gene=OCRL |
| PEX2 | 0.521588504 | https://www.genecards.org/cgi-bin/carddisp.pl?gene=PEX2 |
| PGAM1 | 0.521588504 | https://www.genecards.org/cgi-bin/carddisp.pl?gene=PGAM1 |
| PSTPIP1 | 0.521588504 | https://www.genecards.org/cgi-bin/carddisp.pl?gene=PSTPIP1 |
| RAB5A | 0.521588504 | https://www.genecards.org/cgi-bin/carddisp.pl?gene=RAB5A |
| RNASEH2A | 0.521588504 | https://www.genecards.org/cgi-bin/carddisp.pl?gene=RNASEH2A |
| RPL15 | 0.521588504 | https://www.genecards.org/cgi-bin/carddisp.pl?gene=RPL15 |
| RPS10 | 0.521588504 | https://www.genecards.org/cgi-bin/carddisp.pl?gene=RPS10 |
| SEC63 | 0.521588504 | https://www.genecards.org/cgi-bin/carddisp.pl?gene=SEC63 |
| SFTPA1 | 0.521588504 | https://www.genecards.org/cgi-bin/carddisp.pl?gene=SFTPA1 |
| SHC1 | 0.521588504 | https://www.genecards.org/cgi-bin/carddisp.pl?gene=SHC1 |
| SLC31A1 | 0.521588504 | https://www.genecards.org/cgi-bin/carddisp.pl?gene=SLC31A1 |
| TFE3 | 0.521588504 | https://www.genecards.org/cgi-bin/carddisp.pl?gene=TFE3 |
| TSPO | 0.521588504 | https://www.genecards.org/cgi-bin/carddisp.pl?gene=TSPO |
| WASF1 | 0.521588504 | https://www.genecards.org/cgi-bin/carddisp.pl?gene=WASF1 |
| AAK1 | 0.521588504 | https://www.genecards.org/cgi-bin/carddisp.pl?gene=AAK1 |
| ACKR3 | 0.521588504 | https://www.genecards.org/cgi-bin/carddisp.pl?gene=ACKR3 |
| ACTR2 | 0.521588504 | https://www.genecards.org/cgi-bin/carddisp.pl?gene=ACTR2 |
| ARPC2 | 0.521588504 | https://www.genecards.org/cgi-bin/carddisp.pl?gene=ARPC2 |
| ARPC3 | 0.521588504 | https://www.genecards.org/cgi-bin/carddisp.pl?gene=ARPC3 |
| ASXL1 | 0.521588504 | https://www.genecards.org/cgi-bin/carddisp.pl?gene=ASXL1 |
| B3GAT1 | 0.521588504 | https://www.genecards.org/cgi-bin/carddisp.pl?gene=B3GAT1 |
| CALD1 | 0.521588504 | https://www.genecards.org/cgi-bin/carddisp.pl?gene=CALD1 |
| CD209 | 0.521588504 | https://www.genecards.org/cgi-bin/carddisp.pl?gene=CD209 |
| CDC27 | 0.521588504 | https://www.genecards.org/cgi-bin/carddisp.pl?gene=CDC27 |
| CDC42BPB | 0.521588504 | https://www.genecards.org/cgi-bin/carddisp.pl?gene=CDC42BPB |
| CHL1 | 0.521588504 | https://www.genecards.org/cgi-bin/carddisp.pl?gene=CHL1 |
| CYP4F2 | 0.521588504 | https://www.genecards.org/cgi-bin/carddisp.pl?gene=CYP4F2 |
| DDX41 | 0.521588504 | https://www.genecards.org/cgi-bin/carddisp.pl?gene=DDX41 |
| DNM3 | 0.521588504 | https://www.genecards.org/cgi-bin/carddisp.pl?gene=DNM3 |
| EXO1 | 0.521588504 | https://www.genecards.org/cgi-bin/carddisp.pl?gene=EXO1 |
| FABP7 | 0.521588504 | https://www.genecards.org/cgi-bin/carddisp.pl?gene=FABP7 |
| FZD3 | 0.521588504 | https://www.genecards.org/cgi-bin/carddisp.pl?gene=FZD3 |
| GAP43 | 0.521588504 | https://www.genecards.org/cgi-bin/carddisp.pl?gene=GAP43 |
| GFRA3 | 0.521588504 | https://www.genecards.org/cgi-bin/carddisp.pl?gene=GFRA3 |
| GP1BB | 0.521588504 | https://www.genecards.org/cgi-bin/carddisp.pl?gene=GP1BB |
| GYPC | 0.521588504 | https://www.genecards.org/cgi-bin/carddisp.pl?gene=GYPC |
| HAND2 | 0.521588504 | https://www.genecards.org/cgi-bin/carddisp.pl?gene=HAND2 |
| HNF1B | 0.521588504 | https://www.genecards.org/cgi-bin/carddisp.pl?gene=HNF1B |
| ICAM3 | 0.521588504 | https://www.genecards.org/cgi-bin/carddisp.pl?gene=ICAM3 |
| KCNN3 | 0.521588504 | https://www.genecards.org/cgi-bin/carddisp.pl?gene=KCNN3 |
| KRT13 | 0.521588504 | https://www.genecards.org/cgi-bin/carddisp.pl?gene=KRT13 |
| METAP1 | 0.521588504 | https://www.genecards.org/cgi-bin/carddisp.pl?gene=METAP1 |
| MGP | 0.521588504 | https://www.genecards.org/cgi-bin/carddisp.pl?gene=MGP |
| MOGS | 0.521588504 | https://www.genecards.org/cgi-bin/carddisp.pl?gene=MOGS |
| NAGLU | 0.521588504 | https://www.genecards.org/cgi-bin/carddisp.pl?gene=NAGLU |
| NANS | 0.521588504 | https://www.genecards.org/cgi-bin/carddisp.pl?gene=NANS |
| NCK1 | 0.521588504 | https://www.genecards.org/cgi-bin/carddisp.pl?gene=NCK1 |
| NOX3 | 0.521588504 | https://www.genecards.org/cgi-bin/carddisp.pl?gene=NOX3 |
| NOX4 | 0.521588504 | https://www.genecards.org/cgi-bin/carddisp.pl?gene=NOX4 |
| NTHL1 | 0.521588504 | https://www.genecards.org/cgi-bin/carddisp.pl?gene=NTHL1 |
| PADI2 | 0.521588504 | https://www.genecards.org/cgi-bin/carddisp.pl?gene=PADI2 |
| PAM | 0.521588504 | https://www.genecards.org/cgi-bin/carddisp.pl?gene=PAM |
| PHOX2A | 0.521588504 | https://www.genecards.org/cgi-bin/carddisp.pl?gene=PHOX2A |
| POLR1D | 0.521588504 | https://www.genecards.org/cgi-bin/carddisp.pl?gene=POLR1D |
| RAPGEF4 | 0.521588504 | https://www.genecards.org/cgi-bin/carddisp.pl?gene=RAPGEF4 |
| RPL26 | 0.521588504 | https://www.genecards.org/cgi-bin/carddisp.pl?gene=RPL26 |
| RPS20 | 0.521588504 | https://www.genecards.org/cgi-bin/carddisp.pl?gene=RPS20 |
| RPS24 | 0.521588504 | https://www.genecards.org/cgi-bin/carddisp.pl?gene=RPS24 |
| RPS26 | 0.521588504 | https://www.genecards.org/cgi-bin/carddisp.pl?gene=RPS26 |
| RPS27 | 0.521588504 | https://www.genecards.org/cgi-bin/carddisp.pl?gene=RPS27 |
| SAMHD1 | 0.521588504 | https://www.genecards.org/cgi-bin/carddisp.pl?gene=SAMHD1 |
| SLC33A1 | 0.521588504 | https://www.genecards.org/cgi-bin/carddisp.pl?gene=SLC33A1 |
| SNAI2 | 0.521588504 | https://www.genecards.org/cgi-bin/carddisp.pl?gene=SNAI2 |
| SPG7 | 0.521588504 | https://www.genecards.org/cgi-bin/carddisp.pl?gene=SPG7 |
| TMEM38B | 0.521588504 | https://www.genecards.org/cgi-bin/carddisp.pl?gene=TMEM38B |
| UGT2B7 | 0.521588504 | https://www.genecards.org/cgi-bin/carddisp.pl?gene=UGT2B7 |
| USH1C | 0.521588504 | https://www.genecards.org/cgi-bin/carddisp.pl?gene=USH1C |
| ZIC1 | 0.521588504 | https://www.genecards.org/cgi-bin/carddisp.pl?gene=ZIC1 |
| ABCA5 | 0.521588504 | https://www.genecards.org/cgi-bin/carddisp.pl?gene=ABCA5 |
| ACTR3 | 0.521588504 | https://www.genecards.org/cgi-bin/carddisp.pl?gene=ACTR3 |
| AK3 | 0.521588504 | https://www.genecards.org/cgi-bin/carddisp.pl?gene=AK3 |
| AP3D1 | 0.521588504 | https://www.genecards.org/cgi-bin/carddisp.pl?gene=AP3D1 |
| ASCL1 | 0.521588504 | https://www.genecards.org/cgi-bin/carddisp.pl?gene=ASCL1 |
| ATP12A | 0.521588504 | https://www.genecards.org/cgi-bin/carddisp.pl?gene=ATP12A |
| BACE2 | 0.521588504 | https://www.genecards.org/cgi-bin/carddisp.pl?gene=BACE2 |
| BAIAP2 | 0.521588504 | https://www.genecards.org/cgi-bin/carddisp.pl?gene=BAIAP2 |
| CCK | 0.521588504 | https://www.genecards.org/cgi-bin/carddisp.pl?gene=CCK |
| CCS | 0.521588504 | https://www.genecards.org/cgi-bin/carddisp.pl?gene=CCS |
| COMMD1 | 0.521588504 | https://www.genecards.org/cgi-bin/carddisp.pl?gene=COMMD1 |
| CYP4A11 | 0.521588504 | https://www.genecards.org/cgi-bin/carddisp.pl?gene=CYP4A11 |
| DUOX1 | 0.521588504 | https://www.genecards.org/cgi-bin/carddisp.pl?gene=DUOX1 |
| EIF6 | 0.521588504 | https://www.genecards.org/cgi-bin/carddisp.pl?gene=EIF6 |
| GNPTAB | 0.521588504 | https://www.genecards.org/cgi-bin/carddisp.pl?gene=GNPTAB |
| HIRA | 0.521588504 | https://www.genecards.org/cgi-bin/carddisp.pl?gene=HIRA |
| HLA-DQB1 | 0.521588504 | https://www.genecards.org/cgi-bin/carddisp.pl?gene=HLA-DQB1 |
| KLK8 | 0.521588504 | https://www.genecards.org/cgi-bin/carddisp.pl?gene=KLK8 |
| M6PR | 0.521588504 | https://www.genecards.org/cgi-bin/carddisp.pl?gene=M6PR |
| MT2A | 0.521588504 | https://www.genecards.org/cgi-bin/carddisp.pl?gene=MT2A |
| PMP2 | 0.521588504 | https://www.genecards.org/cgi-bin/carddisp.pl?gene=PMP2 |
| PROKR2 | 0.521588504 | https://www.genecards.org/cgi-bin/carddisp.pl?gene=PROKR2 |
| PTPRU | 0.521588504 | https://www.genecards.org/cgi-bin/carddisp.pl?gene=PTPRU |
| RAPGEF2 | 0.521588504 | https://www.genecards.org/cgi-bin/carddisp.pl?gene=RAPGEF2 |
| RPL3 | 0.521588504 | https://www.genecards.org/cgi-bin/carddisp.pl?gene=RPL3 |
| RPL35A | 0.521588504 | https://www.genecards.org/cgi-bin/carddisp.pl?gene=RPL35A |
| RPN2 | 0.521588504 | https://www.genecards.org/cgi-bin/carddisp.pl?gene=RPN2 |
| RPS14 | 0.521588504 | https://www.genecards.org/cgi-bin/carddisp.pl?gene=RPS14 |
| RPS17 | 0.521588504 | https://www.genecards.org/cgi-bin/carddisp.pl?gene=RPS17 |
| RPS29 | 0.521588504 | https://www.genecards.org/cgi-bin/carddisp.pl?gene=RPS29 |
| SLC25A6 | 0.521588504 | https://www.genecards.org/cgi-bin/carddisp.pl?gene=SLC25A6 |
| SLIT3 | 0.521588504 | https://www.genecards.org/cgi-bin/carddisp.pl?gene=SLIT3 |
| ST8SIA2 | 0.521588504 | https://www.genecards.org/cgi-bin/carddisp.pl?gene=ST8SIA2 |
| TFEB | 0.521588504 | https://www.genecards.org/cgi-bin/carddisp.pl?gene=TFEB |
| TMC6 | 0.521588504 | https://www.genecards.org/cgi-bin/carddisp.pl?gene=TMC6 |
| WASF2 | 0.521588504 | https://www.genecards.org/cgi-bin/carddisp.pl?gene=WASF2 |
| ALOX15B | 0.521588504 | https://www.genecards.org/cgi-bin/carddisp.pl?gene=ALOX15B |
| ANK2 | 0.521588504 | https://www.genecards.org/cgi-bin/carddisp.pl?gene=ANK2 |
| ANKRD26 | 0.521588504 | https://www.genecards.org/cgi-bin/carddisp.pl?gene=ANKRD26 |
| ARHGEF9 | 0.521588504 | https://www.genecards.org/cgi-bin/carddisp.pl?gene=ARHGEF9 |
| ARTN | 0.521588504 | https://www.genecards.org/cgi-bin/carddisp.pl?gene=ARTN |
| ATOX1 | 0.521588504 | https://www.genecards.org/cgi-bin/carddisp.pl?gene=ATOX1 |
| ATP4A | 0.521588504 | https://www.genecards.org/cgi-bin/carddisp.pl?gene=ATP4A |
| BBS10 | 0.521588504 | https://www.genecards.org/cgi-bin/carddisp.pl?gene=BBS10 |
| CD83 | 0.521588504 | https://www.genecards.org/cgi-bin/carddisp.pl?gene=CD83 |
| DNASE2 | 0.521588504 | https://www.genecards.org/cgi-bin/carddisp.pl?gene=DNASE2 |
| ELOVL1 | 0.521588504 | https://www.genecards.org/cgi-bin/carddisp.pl?gene=ELOVL1 |
| ERF | 0.521588504 | https://www.genecards.org/cgi-bin/carddisp.pl?gene=ERF |
| KEL | 0.521588504 | https://www.genecards.org/cgi-bin/carddisp.pl?gene=KEL |
| LRSAM1 | 0.521588504 | https://www.genecards.org/cgi-bin/carddisp.pl?gene=LRSAM1 |
| MKS1 | 0.521588504 | https://www.genecards.org/cgi-bin/carddisp.pl?gene=MKS1 |
| MTSS1 | 0.521588504 | https://www.genecards.org/cgi-bin/carddisp.pl?gene=MTSS1 |
| MXI1 | 0.521588504 | https://www.genecards.org/cgi-bin/carddisp.pl?gene=MXI1 |
| NES | 0.521588504 | https://www.genecards.org/cgi-bin/carddisp.pl?gene=NES |
| NOP10 | 0.521588504 | https://www.genecards.org/cgi-bin/carddisp.pl?gene=NOP10 |
| ORC2 | 0.521588504 | https://www.genecards.org/cgi-bin/carddisp.pl?gene=ORC2 |
| RAB33B | 0.521588504 | https://www.genecards.org/cgi-bin/carddisp.pl?gene=RAB33B |
| RAB6A | 0.521588504 | https://www.genecards.org/cgi-bin/carddisp.pl?gene=RAB6A |
| RFXANK | 0.521588504 | https://www.genecards.org/cgi-bin/carddisp.pl?gene=RFXANK |
| RPGR | 0.521588504 | https://www.genecards.org/cgi-bin/carddisp.pl?gene=RPGR |
| RPL22 | 0.521588504 | https://www.genecards.org/cgi-bin/carddisp.pl?gene=RPL22 |
| RYR3 | 0.521588504 | https://www.genecards.org/cgi-bin/carddisp.pl?gene=RYR3 |
| SLC30A2 | 0.521588504 | https://www.genecards.org/cgi-bin/carddisp.pl?gene=SLC30A2 |
| SLC35A3 | 0.521588504 | https://www.genecards.org/cgi-bin/carddisp.pl?gene=SLC35A3 |
| SNX9 | 0.521588504 | https://www.genecards.org/cgi-bin/carddisp.pl?gene=SNX9 |
| SPAM1 | 0.521588504 | https://www.genecards.org/cgi-bin/carddisp.pl?gene=SPAM1 |
| SRP72 | 0.521588504 | https://www.genecards.org/cgi-bin/carddisp.pl?gene=SRP72 |
| TINF2 | 0.521588504 | https://www.genecards.org/cgi-bin/carddisp.pl?gene=TINF2 |
| TMC8 | 0.521588504 | https://www.genecards.org/cgi-bin/carddisp.pl?gene=TMC8 |
| TRIP10 | 0.521588504 | https://www.genecards.org/cgi-bin/carddisp.pl?gene=TRIP10 |
| USP18 | 0.521588504 | https://www.genecards.org/cgi-bin/carddisp.pl?gene=USP18 |
| ADAMTS17 | 0.521588504 | https://www.genecards.org/cgi-bin/carddisp.pl?gene=ADAMTS17 |
| AK4 | 0.521588504 | https://www.genecards.org/cgi-bin/carddisp.pl?gene=AK4 |
| AK5 | 0.521588504 | https://www.genecards.org/cgi-bin/carddisp.pl?gene=AK5 |
| ALG11 | 0.521588504 | https://www.genecards.org/cgi-bin/carddisp.pl?gene=ALG11 |
| ALG13 | 0.521588504 | https://www.genecards.org/cgi-bin/carddisp.pl?gene=ALG13 |
| AP1S1 | 0.521588504 | https://www.genecards.org/cgi-bin/carddisp.pl?gene=AP1S1 |
| AP3S1 | 0.521588504 | https://www.genecards.org/cgi-bin/carddisp.pl?gene=AP3S1 |
| BBS1 | 0.521588504 | https://www.genecards.org/cgi-bin/carddisp.pl?gene=BBS1 |
| BLVRB | 0.521588504 | https://www.genecards.org/cgi-bin/carddisp.pl?gene=BLVRB |
| CALB2 | 0.521588504 | https://www.genecards.org/cgi-bin/carddisp.pl?gene=CALB2 |
| CLEC4M | 0.521588504 | https://www.genecards.org/cgi-bin/carddisp.pl?gene=CLEC4M |
| COG5 | 0.521588504 | https://www.genecards.org/cgi-bin/carddisp.pl?gene=COG5 |
| COG8 | 0.521588504 | https://www.genecards.org/cgi-bin/carddisp.pl?gene=COG8 |
| COPZ1 | 0.521588504 | https://www.genecards.org/cgi-bin/carddisp.pl?gene=COPZ1 |
| CTCFL | 0.521588504 | https://www.genecards.org/cgi-bin/carddisp.pl?gene=CTCFL |
| EDN2 | 0.521588504 | https://www.genecards.org/cgi-bin/carddisp.pl?gene=EDN2 |
| ELAVL4 | 0.521588504 | https://www.genecards.org/cgi-bin/carddisp.pl?gene=ELAVL4 |
| EMG1 | 0.521588504 | https://www.genecards.org/cgi-bin/carddisp.pl?gene=EMG1 |
| FOXD3 | 0.521588504 | https://www.genecards.org/cgi-bin/carddisp.pl?gene=FOXD3 |
| GJB4 | 0.521588504 | https://www.genecards.org/cgi-bin/carddisp.pl?gene=GJB4 |
| HOXA3 | 0.521588504 | https://www.genecards.org/cgi-bin/carddisp.pl?gene=HOXA3 |
| IFI30 | 0.521588504 | https://www.genecards.org/cgi-bin/carddisp.pl?gene=IFI30 |
| KRT12 | 0.521588504 | https://www.genecards.org/cgi-bin/carddisp.pl?gene=KRT12 |
| KRT3 | 0.521588504 | https://www.genecards.org/cgi-bin/carddisp.pl?gene=KRT3 |
| NHLRC1 | 0.521588504 | https://www.genecards.org/cgi-bin/carddisp.pl?gene=NHLRC1 |
| NPHP3 | 0.521588504 | https://www.genecards.org/cgi-bin/carddisp.pl?gene=NPHP3 |
| PIGM | 0.521588504 | https://www.genecards.org/cgi-bin/carddisp.pl?gene=PIGM |
| PIGV | 0.521588504 | https://www.genecards.org/cgi-bin/carddisp.pl?gene=PIGV |
| PPL | 0.521588504 | https://www.genecards.org/cgi-bin/carddisp.pl?gene=PPL |
| RAB1B | 0.521588504 | https://www.genecards.org/cgi-bin/carddisp.pl?gene=RAB1B |
| RAB35 | 0.521588504 | https://www.genecards.org/cgi-bin/carddisp.pl?gene=RAB35 |
| RAB8A | 0.521588504 | https://www.genecards.org/cgi-bin/carddisp.pl?gene=RAB8A |
| RPL29 | 0.521588504 | https://www.genecards.org/cgi-bin/carddisp.pl?gene=RPL29 |
| RPL6 | 0.521588504 | https://www.genecards.org/cgi-bin/carddisp.pl?gene=RPL6 |
| RPS15 | 0.521588504 | https://www.genecards.org/cgi-bin/carddisp.pl?gene=RPS15 |
| RPS28 | 0.521588504 | https://www.genecards.org/cgi-bin/carddisp.pl?gene=RPS28 |
| SEMA3B | 0.521588504 | https://www.genecards.org/cgi-bin/carddisp.pl?gene=SEMA3B |
| SEMA6A | 0.521588504 | https://www.genecards.org/cgi-bin/carddisp.pl?gene=SEMA6A |
| SLC30A1 | 0.521588504 | https://www.genecards.org/cgi-bin/carddisp.pl?gene=SLC30A1 |
| SLC35D1 | 0.521588504 | https://www.genecards.org/cgi-bin/carddisp.pl?gene=SLC35D1 |
| STMN2 | 0.521588504 | https://www.genecards.org/cgi-bin/carddisp.pl?gene=STMN2 |
| VPS29 | 0.521588504 | https://www.genecards.org/cgi-bin/carddisp.pl?gene=VPS29 |
| ABCA8 | 0.521588504 | https://www.genecards.org/cgi-bin/carddisp.pl?gene=ABCA8 |
| AFAP1 | 0.521588504 | https://www.genecards.org/cgi-bin/carddisp.pl?gene=AFAP1 |
| AK7 | 0.521588504 | https://www.genecards.org/cgi-bin/carddisp.pl?gene=AK7 |
| CEBPZ | 0.521588504 | https://www.genecards.org/cgi-bin/carddisp.pl?gene=CEBPZ |
| COG6 | 0.521588504 | https://www.genecards.org/cgi-bin/carddisp.pl?gene=COG6 |
| COG7 | 0.521588504 | https://www.genecards.org/cgi-bin/carddisp.pl?gene=COG7 |
| ESCO2 | 0.521588504 | https://www.genecards.org/cgi-bin/carddisp.pl?gene=ESCO2 |
| FMN1 | 0.521588504 | https://www.genecards.org/cgi-bin/carddisp.pl?gene=FMN1 |
| HOXC4 | 0.521588504 | https://www.genecards.org/cgi-bin/carddisp.pl?gene=HOXC4 |
| IFIT1 | 0.521588504 | https://www.genecards.org/cgi-bin/carddisp.pl?gene=IFIT1 |
| MMAA | 0.521588504 | https://www.genecards.org/cgi-bin/carddisp.pl?gene=MMAA |
| MT3 | 0.521588504 | https://www.genecards.org/cgi-bin/carddisp.pl?gene=MT3 |
| MUC5AC | 0.521588504 | https://www.genecards.org/cgi-bin/carddisp.pl?gene=MUC5AC |
| MYO18A | 0.521588504 | https://www.genecards.org/cgi-bin/carddisp.pl?gene=MYO18A |
| RASSF5 | 0.521588504 | https://www.genecards.org/cgi-bin/carddisp.pl?gene=RASSF5 |
| SNX27 | 0.521588504 | https://www.genecards.org/cgi-bin/carddisp.pl?gene=SNX27 |
| UBR4 | 0.521588504 | https://www.genecards.org/cgi-bin/carddisp.pl?gene=UBR4 |
| WASF3 | 0.521588504 | https://www.genecards.org/cgi-bin/carddisp.pl?gene=WASF3 |
| WDR19 | 0.521588504 | https://www.genecards.org/cgi-bin/carddisp.pl?gene=WDR19 |
| ABCA6 | 0.521588504 | https://www.genecards.org/cgi-bin/carddisp.pl?gene=ABCA6 |
| AEBP2 | 0.521588504 | https://www.genecards.org/cgi-bin/carddisp.pl?gene=AEBP2 |
| ARSD | 0.521588504 | https://www.genecards.org/cgi-bin/carddisp.pl?gene=ARSD |
| ARVCF | 0.521588504 | https://www.genecards.org/cgi-bin/carddisp.pl?gene=ARVCF |
| BLZF1 | 0.521588504 | https://www.genecards.org/cgi-bin/carddisp.pl?gene=BLZF1 |
| BYSL | 0.521588504 | https://www.genecards.org/cgi-bin/carddisp.pl?gene=BYSL |
| CELSR3 | 0.521588504 | https://www.genecards.org/cgi-bin/carddisp.pl?gene=CELSR3 |
| CLEC11A | 0.521588504 | https://www.genecards.org/cgi-bin/carddisp.pl?gene=CLEC11A |
| CLEC6A | 0.521588504 | https://www.genecards.org/cgi-bin/carddisp.pl?gene=CLEC6A |
| COX11 | 0.521588504 | https://www.genecards.org/cgi-bin/carddisp.pl?gene=COX11 |
| CTNNAL1 | 0.521588504 | https://www.genecards.org/cgi-bin/carddisp.pl?gene=CTNNAL1 |
| DOK6 | 0.521588504 | https://www.genecards.org/cgi-bin/carddisp.pl?gene=DOK6 |
| ERCC6L2 | 0.521588504 | https://www.genecards.org/cgi-bin/carddisp.pl?gene=ERCC6L2 |
| FNBP1 | 0.521588504 | https://www.genecards.org/cgi-bin/carddisp.pl?gene=FNBP1 |
| GNLY | 0.521588504 | https://www.genecards.org/cgi-bin/carddisp.pl?gene=GNLY |
| GRHL3 | 0.521588504 | https://www.genecards.org/cgi-bin/carddisp.pl?gene=GRHL3 |
| KIFBP | 0.521588504 | https://www.genecards.org/cgi-bin/carddisp.pl?gene=KIFBP |
| KRT71 | 0.521588504 | https://www.genecards.org/cgi-bin/carddisp.pl?gene=KRT71 |
| MAPRE3 | 0.521588504 | https://www.genecards.org/cgi-bin/carddisp.pl?gene=MAPRE3 |
| MT1A | 0.521588504 | https://www.genecards.org/cgi-bin/carddisp.pl?gene=MT1A |
| NCKIPSD | 0.521588504 | https://www.genecards.org/cgi-bin/carddisp.pl?gene=NCKIPSD |
| NIPA1 | 0.521588504 | https://www.genecards.org/cgi-bin/carddisp.pl?gene=NIPA1 |
| RNASEH2C | 0.521588504 | https://www.genecards.org/cgi-bin/carddisp.pl?gene=RNASEH2C |
| RPH3AL | 0.521588504 | https://www.genecards.org/cgi-bin/carddisp.pl?gene=RPH3AL |
| RPL38 | 0.521588504 | https://www.genecards.org/cgi-bin/carddisp.pl?gene=RPL38 |
| RSL24D1 | 0.521588504 | https://www.genecards.org/cgi-bin/carddisp.pl?gene=RSL24D1 |
| SETD5 | 0.521588504 | https://www.genecards.org/cgi-bin/carddisp.pl?gene=SETD5 |
| SH3PXD2B | 0.521588504 | https://www.genecards.org/cgi-bin/carddisp.pl?gene=SH3PXD2B |
| SOX8 | 0.521588504 | https://www.genecards.org/cgi-bin/carddisp.pl?gene=SOX8 |
| STX2 | 0.521588504 | https://www.genecards.org/cgi-bin/carddisp.pl?gene=STX2 |
| SUPT5H | 0.521588504 | https://www.genecards.org/cgi-bin/carddisp.pl?gene=SUPT5H |
| TGM6 | 0.521588504 | https://www.genecards.org/cgi-bin/carddisp.pl?gene=TGM6 |
| TGOLN2 | 0.521588504 | https://www.genecards.org/cgi-bin/carddisp.pl?gene=TGOLN2 |
| TPCN2 | 0.521588504 | https://www.genecards.org/cgi-bin/carddisp.pl?gene=TPCN2 |
| TSPAN2 | 0.521588504 | https://www.genecards.org/cgi-bin/carddisp.pl?gene=TSPAN2 |
| ZDHHC8 | 0.521588504 | https://www.genecards.org/cgi-bin/carddisp.pl?gene=ZDHHC8 |
| ABCA9 | 0.521588504 | https://www.genecards.org/cgi-bin/carddisp.pl?gene=ABCA9 |
| ANKK1 | 0.521588504 | https://www.genecards.org/cgi-bin/carddisp.pl?gene=ANKK1 |
| ARFRP1 | 0.521588504 | https://www.genecards.org/cgi-bin/carddisp.pl?gene=ARFRP1 |
| COG3 | 0.521588504 | https://www.genecards.org/cgi-bin/carddisp.pl?gene=COG3 |
| DEFA5 | 0.521588504 | https://www.genecards.org/cgi-bin/carddisp.pl?gene=DEFA5 |
| DNAH8 | 0.521588504 | https://www.genecards.org/cgi-bin/carddisp.pl?gene=DNAH8 |
| ELAVL3 | 0.521588504 | https://www.genecards.org/cgi-bin/carddisp.pl?gene=ELAVL3 |
| EXPH5 | 0.521588504 | https://www.genecards.org/cgi-bin/carddisp.pl?gene=EXPH5 |
| GNPTG | 0.521588504 | https://www.genecards.org/cgi-bin/carddisp.pl?gene=GNPTG |
| HGSNAT | 0.521588504 | https://www.genecards.org/cgi-bin/carddisp.pl?gene=HGSNAT |
| HOXB5 | 0.521588504 | https://www.genecards.org/cgi-bin/carddisp.pl?gene=HOXB5 |
| KRT33B | 0.521588504 | https://www.genecards.org/cgi-bin/carddisp.pl?gene=KRT33B |
| KRT74 | 0.521588504 | https://www.genecards.org/cgi-bin/carddisp.pl?gene=KRT74 |
| LUC7L2 | 0.521588504 | https://www.genecards.org/cgi-bin/carddisp.pl?gene=LUC7L2 |
| MYOM2 | 0.521588504 | https://www.genecards.org/cgi-bin/carddisp.pl?gene=MYOM2 |
| NIP7 | 0.521588504 | https://www.genecards.org/cgi-bin/carddisp.pl?gene=NIP7 |
| NIPA2 | 0.521588504 | https://www.genecards.org/cgi-bin/carddisp.pl?gene=NIPA2 |
| NMD3 | 0.521588504 | https://www.genecards.org/cgi-bin/carddisp.pl?gene=NMD3 |
| NOX5 | 0.521588504 | https://www.genecards.org/cgi-bin/carddisp.pl?gene=NOX5 |
| NOXO1 | 0.521588504 | https://www.genecards.org/cgi-bin/carddisp.pl?gene=NOXO1 |
| PCDHA12 | 0.521588504 | https://www.genecards.org/cgi-bin/carddisp.pl?gene=PCDHA12 |
| PCDHGC4 | 0.521588504 | https://www.genecards.org/cgi-bin/carddisp.pl?gene=PCDHGC4 |
| PRSS16 | 0.521588504 | https://www.genecards.org/cgi-bin/carddisp.pl?gene=PRSS16 |
| PSPN | 0.521588504 | https://www.genecards.org/cgi-bin/carddisp.pl?gene=PSPN |
| RASGEF1A | 0.521588504 | https://www.genecards.org/cgi-bin/carddisp.pl?gene=RASGEF1A |
| RNASEH2B | 0.521588504 | https://www.genecards.org/cgi-bin/carddisp.pl?gene=RNASEH2B |
| RNF150 | 0.521588504 | https://www.genecards.org/cgi-bin/carddisp.pl?gene=RNF150 |
| RNF167 | 0.521588504 | https://www.genecards.org/cgi-bin/carddisp.pl?gene=RNF167 |
| RP9 | 0.521588504 | https://www.genecards.org/cgi-bin/carddisp.pl?gene=RP9 |
| SAMD9L | 0.521588504 | https://www.genecards.org/cgi-bin/carddisp.pl?gene=SAMD9L |
| SH2D3C | 0.521588504 | https://www.genecards.org/cgi-bin/carddisp.pl?gene=SH2D3C |
| TBC1D10C | 0.521588504 | https://www.genecards.org/cgi-bin/carddisp.pl?gene=TBC1D10C |
| TBC1D5 | 0.521588504 | https://www.genecards.org/cgi-bin/carddisp.pl?gene=TBC1D5 |
| TSR2 | 0.521588504 | https://www.genecards.org/cgi-bin/carddisp.pl?gene=TSR2 |
| VAMP5 | 0.521588504 | https://www.genecards.org/cgi-bin/carddisp.pl?gene=VAMP5 |
| VPS16 | 0.521588504 | https://www.genecards.org/cgi-bin/carddisp.pl?gene=VPS16 |
| APLF | 0.521588504 | https://www.genecards.org/cgi-bin/carddisp.pl?gene=APLF |
| CAPN12 | 0.521588504 | https://www.genecards.org/cgi-bin/carddisp.pl?gene=CAPN12 |
| CDC42BPG | 0.521588504 | https://www.genecards.org/cgi-bin/carddisp.pl?gene=CDC42BPG |
| CGAS | 0.521588504 | https://www.genecards.org/cgi-bin/carddisp.pl?gene=CGAS |
| COX17 | 0.521588504 | https://www.genecards.org/cgi-bin/carddisp.pl?gene=COX17 |
| GTDC1 | 0.521588504 | https://www.genecards.org/cgi-bin/carddisp.pl?gene=GTDC1 |
| LSG1 | 0.521588504 | https://www.genecards.org/cgi-bin/carddisp.pl?gene=LSG1 |
| MBD6 | 0.521588504 | https://www.genecards.org/cgi-bin/carddisp.pl?gene=MBD6 |
| NDUFAF5 | 0.521588504 | https://www.genecards.org/cgi-bin/carddisp.pl?gene=NDUFAF5 |
| NOXA1 | 0.521588504 | https://www.genecards.org/cgi-bin/carddisp.pl?gene=NOXA1 |
| PCDHGB2 | 0.521588504 | https://www.genecards.org/cgi-bin/carddisp.pl?gene=PCDHGB2 |
| PLB1 | 0.521588504 | https://www.genecards.org/cgi-bin/carddisp.pl?gene=PLB1 |
| PROK1 | 0.521588504 | https://www.genecards.org/cgi-bin/carddisp.pl?gene=PROK1 |
| RPF2 | 0.521588504 | https://www.genecards.org/cgi-bin/carddisp.pl?gene=RPF2 |
| RPTN | 0.521588504 | https://www.genecards.org/cgi-bin/carddisp.pl?gene=RPTN |
| RXYLT1 | 0.521588504 | https://www.genecards.org/cgi-bin/carddisp.pl?gene=RXYLT1 |
| SLC31A2 | 0.521588504 | https://www.genecards.org/cgi-bin/carddisp.pl?gene=SLC31A2 |
| SPRR1A | 0.521588504 | https://www.genecards.org/cgi-bin/carddisp.pl?gene=SPRR1A |
| TLX2 | 0.521588504 | https://www.genecards.org/cgi-bin/carddisp.pl?gene=TLX2 |
| TTC4 | 0.521588504 | https://www.genecards.org/cgi-bin/carddisp.pl?gene=TTC4 |
| USB1 | 0.521588504 | https://www.genecards.org/cgi-bin/carddisp.pl?gene=USB1 |
| UTP4 | 0.521588504 | https://www.genecards.org/cgi-bin/carddisp.pl?gene=UTP4 |
| AK9 | 0.521588504 | https://www.genecards.org/cgi-bin/carddisp.pl?gene=AK9 |
| DEDD | 0.521588504 | https://www.genecards.org/cgi-bin/carddisp.pl?gene=DEDD |
| DPF3 | 0.521588504 | https://www.genecards.org/cgi-bin/carddisp.pl?gene=DPF3 |
| GFRA4 | 0.521588504 | https://www.genecards.org/cgi-bin/carddisp.pl?gene=GFRA4 |
| HRNR | 0.521588504 | https://www.genecards.org/cgi-bin/carddisp.pl?gene=HRNR |
| KIF26A | 0.521588504 | https://www.genecards.org/cgi-bin/carddisp.pl?gene=KIF26A |
| KLHL24 | 0.521588504 | https://www.genecards.org/cgi-bin/carddisp.pl?gene=KLHL24 |
| MEIS3 | 0.521588504 | https://www.genecards.org/cgi-bin/carddisp.pl?gene=MEIS3 |
| MRTO4 | 0.521588504 | https://www.genecards.org/cgi-bin/carddisp.pl?gene=MRTO4 |
| MTUS1 | 0.521588504 | https://www.genecards.org/cgi-bin/carddisp.pl?gene=MTUS1 |
| MTUS2 | 0.521588504 | https://www.genecards.org/cgi-bin/carddisp.pl?gene=MTUS2 |
| NRSN1 | 0.521588504 | https://www.genecards.org/cgi-bin/carddisp.pl?gene=NRSN1 |
| PCDHGA3 | 0.521588504 | https://www.genecards.org/cgi-bin/carddisp.pl?gene=PCDHGA3 |
| PGAP2 | 0.521588504 | https://www.genecards.org/cgi-bin/carddisp.pl?gene=PGAP2 |
| RNF133 | 0.521588504 | https://www.genecards.org/cgi-bin/carddisp.pl?gene=RNF133 |
| SEC22B | 0.521588504 | https://www.genecards.org/cgi-bin/carddisp.pl?gene=SEC22B |
| SH2D3A | 0.521588504 | https://www.genecards.org/cgi-bin/carddisp.pl?gene=SH2D3A |
| TMEM79 | 0.521588504 | https://www.genecards.org/cgi-bin/carddisp.pl?gene=TMEM79 |
| UMODL1 | 0.521588504 | https://www.genecards.org/cgi-bin/carddisp.pl?gene=UMODL1 |
| WDR76 | 0.521588504 | https://www.genecards.org/cgi-bin/carddisp.pl?gene=WDR76 |
| ANKLE1 | 0.521588504 | https://www.genecards.org/cgi-bin/carddisp.pl?gene=ANKLE1 |
| ARHGEF37 | 0.521588504 | https://www.genecards.org/cgi-bin/carddisp.pl?gene=ARHGEF37 |
| COX19 | 0.521588504 | https://www.genecards.org/cgi-bin/carddisp.pl?gene=COX19 |
| CYP4A22 | 0.521588504 | https://www.genecards.org/cgi-bin/carddisp.pl?gene=CYP4A22 |
| LYSMD4 | 0.521588504 | https://www.genecards.org/cgi-bin/carddisp.pl?gene=LYSMD4 |
| MLN | 0.521588504 | https://www.genecards.org/cgi-bin/carddisp.pl?gene=MLN |
| NIPAL2 | 0.521588504 | https://www.genecards.org/cgi-bin/carddisp.pl?gene=NIPAL2 |
| PHACTR4 | 0.521588504 | https://www.genecards.org/cgi-bin/carddisp.pl?gene=PHACTR4 |
| PIGY | 0.521588504 | https://www.genecards.org/cgi-bin/carddisp.pl?gene=PIGY |
| POGLUT2 | 0.521588504 | https://www.genecards.org/cgi-bin/carddisp.pl?gene=POGLUT2 |
| PSMB11 | 0.521588504 | https://www.genecards.org/cgi-bin/carddisp.pl?gene=PSMB11 |
| ZCCHC14 | 0.521588504 | https://www.genecards.org/cgi-bin/carddisp.pl?gene=ZCCHC14 |
| ZNF2 | 0.521588504 | https://www.genecards.org/cgi-bin/carddisp.pl?gene=ZNF2 |
| CCDC66 | 0.521588504 | https://www.genecards.org/cgi-bin/carddisp.pl?gene=CCDC66 |
| PTRHD1 | 0.521588504 | https://www.genecards.org/cgi-bin/carddisp.pl?gene=PTRHD1 |
| TMBIM4 | 0.521588504 | https://www.genecards.org/cgi-bin/carddisp.pl?gene=TMBIM4 |
| ZNF609 | 0.521588504 | https://www.genecards.org/cgi-bin/carddisp.pl?gene=ZNF609 |
| ARSH | 0.521588504 | https://www.genecards.org/cgi-bin/carddisp.pl?gene=ARSH |
| KLHDC7A | 0.521588504 | https://www.genecards.org/cgi-bin/carddisp.pl?gene=KLHDC7A |
| KLK9 | 0.521588504 | https://www.genecards.org/cgi-bin/carddisp.pl?gene=KLK9 |
| MT-CYB | 0.521588504 | https://www.genecards.org/cgi-bin/carddisp.pl?gene=MT-CYB |
| WFDC12 | 0.521588504 | https://www.genecards.org/cgi-bin/carddisp.pl?gene=WFDC12 |
| ZNF621 | 0.521588504 | https://www.genecards.org/cgi-bin/carddisp.pl?gene=ZNF621 |
| FAM162B | 0.521588504 | https://www.genecards.org/cgi-bin/carddisp.pl?gene=FAM162B |
| GARIN5A | 0.521588504 | https://www.genecards.org/cgi-bin/carddisp.pl?gene=GARIN5A |
| RAB7B | 0.521588504 | https://www.genecards.org/cgi-bin/carddisp.pl?gene=RAB7B |
| RNF148 | 0.521588504 | https://www.genecards.org/cgi-bin/carddisp.pl?gene=RNF148 |
| UTF1 | 0.521588504 | https://www.genecards.org/cgi-bin/carddisp.pl?gene=UTF1 |
| ZNRF4 | 0.521588504 | https://www.genecards.org/cgi-bin/carddisp.pl?gene=ZNRF4 |
| CFAP47 | 0.521588504 | https://www.genecards.org/cgi-bin/carddisp.pl?gene=CFAP47 |
| KPRP | 0.521588504 | https://www.genecards.org/cgi-bin/carddisp.pl?gene=KPRP |
| LCE3D | 0.521588504 | https://www.genecards.org/cgi-bin/carddisp.pl?gene=LCE3D |
| FOXI3 | 0.521588504 | https://www.genecards.org/cgi-bin/carddisp.pl?gene=FOXI3 |
| MEIG1 | 0.521588504 | https://www.genecards.org/cgi-bin/carddisp.pl?gene=MEIG1 |
| PSORS1C1 | 0.521588504 | https://www.genecards.org/cgi-bin/carddisp.pl?gene=PSORS1C1 |
| SPRR2B | 0.521588504 | https://www.genecards.org/cgi-bin/carddisp.pl?gene=SPRR2B |
| ANKRD60 | 0.521588504 | https://www.genecards.org/cgi-bin/carddisp.pl?gene=ANKRD60 |
| SPATA46 | 0.521588504 | https://www.genecards.org/cgi-bin/carddisp.pl?gene=SPATA46 |
| TMPRSS11F | 0.521588504 | https://www.genecards.org/cgi-bin/carddisp.pl?gene=TMPRSS11F |
| HMSD | 0.521588504 | https://www.genecards.org/cgi-bin/carddisp.pl?gene=HMSD |
| IQCF3 | 0.521588504 | https://www.genecards.org/cgi-bin/carddisp.pl?gene=IQCF3 |
| VNN3P | 0.521588504 | https://www.genecards.org/cgi-bin/carddisp.pl?gene=VNN3P |
| HSFX1 | 0.521588504 | https://www.genecards.org/cgi-bin/carddisp.pl?gene=HSFX1 |
| HSFX2 | 0.521588504 | https://www.genecards.org/cgi-bin/carddisp.pl?gene=HSFX2 |
| MIR369 | 0.521588504 | https://www.genecards.org/cgi-bin/carddisp.pl?gene=MIR369 |
| SPATA41 | 0.521588504 | https://www.genecards.org/cgi-bin/carddisp.pl?gene=SPATA41 |
| MTRNR2L3 | 0.521588504 | https://www.genecards.org/cgi-bin/carddisp.pl?gene=MTRNR2L3 |
| MIR488 | 0.521588504 | https://www.genecards.org/cgi-bin/carddisp.pl?gene=MIR488 |
| KC6 | 0.521588504 | https://www.genecards.org/cgi-bin/carddisp.pl?gene=KC6 |
| LINC01518 | 0.521588504 | https://www.genecards.org/cgi-bin/carddisp.pl?gene=LINC01518 |
| MIR518D | 0.521588504 | https://www.genecards.org/cgi-bin/carddisp.pl?gene=MIR518D |
| MT-TD | 0.521588504 | https://www.genecards.org/cgi-bin/carddisp.pl?gene=MT-TD |
| MIR1324 | 0.521588504 | https://www.genecards.org/cgi-bin/carddisp.pl?gene=MIR1324 |
| MIR3975 | 0.521588504 | https://www.genecards.org/cgi-bin/carddisp.pl?gene=MIR3975 |
| MIR4273 | 0.521588504 | https://www.genecards.org/cgi-bin/carddisp.pl?gene=MIR4273 |
| MIR4325 | 0.521588504 | https://www.genecards.org/cgi-bin/carddisp.pl?gene=MIR4325 |
| MIR6082 | 0.521588504 | https://www.genecards.org/cgi-bin/carddisp.pl?gene=MIR6082 |
| LOC106694316 | 0.521588504 | https://www.genecards.org/cgi-bin/carddisp.pl?gene=LOC106694316 |
| LOC107303343 | 0.521588504 | https://www.genecards.org/cgi-bin/carddisp.pl?gene=LOC107303343 |
| HSCR5 | 0.521588504 | https://www.genecards.org/cgi-bin/carddisp.pl?gene=HSCR5 |
| ARCI7 | 0.521588504 | https://www.genecards.org/cgi-bin/carddisp.pl?gene=ARCI7 |
| HSCR6 | 0.521588504 | https://www.genecards.org/cgi-bin/carddisp.pl?gene=HSCR6 |
| HSCR7 | 0.521588504 | https://www.genecards.org/cgi-bin/carddisp.pl?gene=HSCR7 |
| HSCR8 | 0.521588504 | https://www.genecards.org/cgi-bin/carddisp.pl?gene=HSCR8 |
| HSCR9 | 0.521588504 | https://www.genecards.org/cgi-bin/carddisp.pl?gene=HSCR9 |
| PRKCD | 0.503477454 | https://www.genecards.org/cgi-bin/carddisp.pl?gene=PRKCD |
| GSK3B | 0.503477454 | https://www.genecards.org/cgi-bin/carddisp.pl?gene=GSK3B |
| MAPK9 | 0.503477454 | https://www.genecards.org/cgi-bin/carddisp.pl?gene=MAPK9 |
| YAP1 | 0.503477454 | https://www.genecards.org/cgi-bin/carddisp.pl?gene=YAP1 |
| KCNJ5 | 0.503477454 | https://www.genecards.org/cgi-bin/carddisp.pl?gene=KCNJ5 |
| SCARB1 | 0.503477454 | https://www.genecards.org/cgi-bin/carddisp.pl?gene=SCARB1 |
| SCARB2 | 0.503477454 | https://www.genecards.org/cgi-bin/carddisp.pl?gene=SCARB2 |
| CX3CL1 | 0.503477454 | https://www.genecards.org/cgi-bin/carddisp.pl?gene=CX3CL1 |
| TNFRSF14 | 0.503477454 | https://www.genecards.org/cgi-bin/carddisp.pl?gene=TNFRSF14 |
| ME1 | 0.503477454 | https://www.genecards.org/cgi-bin/carddisp.pl?gene=ME1 |
| PDSS1 | 0.503477454 | https://www.genecards.org/cgi-bin/carddisp.pl?gene=PDSS1 |
| SMC4 | 0.503477454 | https://www.genecards.org/cgi-bin/carddisp.pl?gene=SMC4 |
| EIF2A | 0.503477454 | https://www.genecards.org/cgi-bin/carddisp.pl?gene=EIF2A |
| GSTA4 | 0.503477454 | https://www.genecards.org/cgi-bin/carddisp.pl?gene=GSTA4 |
| PECAM1 | 0.503477454 | https://www.genecards.org/cgi-bin/carddisp.pl?gene=PECAM1 |
| CYB561 | 0.503477454 | https://www.genecards.org/cgi-bin/carddisp.pl?gene=CYB561 |
| MRGPRX1 | 0.503477454 | https://www.genecards.org/cgi-bin/carddisp.pl?gene=MRGPRX1 |
| TENT5C | 0.503477454 | https://www.genecards.org/cgi-bin/carddisp.pl?gene=TENT5C |
| MIR29B1 | 0.503477454 | https://www.genecards.org/cgi-bin/carddisp.pl?gene=MIR29B1 |
| MIR1290 | 0.503477454 | https://www.genecards.org/cgi-bin/carddisp.pl?gene=MIR1290 |
| MIR6503 | 0.503477454 | https://www.genecards.org/cgi-bin/carddisp.pl?gene=MIR6503 |
| STXBP2 | 0.494824499 | https://www.genecards.org/cgi-bin/carddisp.pl?gene=STXBP2 |
| UNC13D | 0.494824499 | https://www.genecards.org/cgi-bin/carddisp.pl?gene=UNC13D |
| PIK3CD | 0.481632382 | https://www.genecards.org/cgi-bin/carddisp.pl?gene=PIK3CD |
| LPL | 0.481632382 | https://www.genecards.org/cgi-bin/carddisp.pl?gene=LPL |
| F8 | 0.481632382 | https://www.genecards.org/cgi-bin/carddisp.pl?gene=F8 |
| TAB3 | 0.481632382 | https://www.genecards.org/cgi-bin/carddisp.pl?gene=TAB3 |
| TBX5 | 0.475289434 | https://www.genecards.org/cgi-bin/carddisp.pl?gene=TBX5 |
| ARPC1B | 0.475289434 | https://www.genecards.org/cgi-bin/carddisp.pl?gene=ARPC1B |
| PRIM1 | 0.475289434 | https://www.genecards.org/cgi-bin/carddisp.pl?gene=PRIM1 |
| IL36RN | 0.475289434 | https://www.genecards.org/cgi-bin/carddisp.pl?gene=IL36RN |
| AP1S3 | 0.475289434 | https://www.genecards.org/cgi-bin/carddisp.pl?gene=AP1S3 |
| MAL | 0.475289434 | https://www.genecards.org/cgi-bin/carddisp.pl?gene=MAL |
| C2orf69 | 0.475289434 | https://www.genecards.org/cgi-bin/carddisp.pl?gene=C2orf69 |
| LOC124629375 | 0.475289434 | https://www.genecards.org/cgi-bin/carddisp.pl?gene=LOC124629375 |
| LOC126805688 | 0.475289434 | https://www.genecards.org/cgi-bin/carddisp.pl?gene=LOC126805688 |
| ENDOVESL | 0.475289434 | https://www.genecards.org/cgi-bin/carddisp.pl?gene=ENDOVESL |
| P4HB | 0.469714344 | https://www.genecards.org/cgi-bin/carddisp.pl?gene=P4HB |
| UBA1 | 0.469714344 | https://www.genecards.org/cgi-bin/carddisp.pl?gene=UBA1 |
| CD81 | 0.469714344 | https://www.genecards.org/cgi-bin/carddisp.pl?gene=CD81 |
| FZD5 | 0.469714344 | https://www.genecards.org/cgi-bin/carddisp.pl?gene=FZD5 |
| ROCK2 | 0.469714344 | https://www.genecards.org/cgi-bin/carddisp.pl?gene=ROCK2 |
| ATF1 | 0.469714344 | https://www.genecards.org/cgi-bin/carddisp.pl?gene=ATF1 |
| LTA4H | 0.469714344 | https://www.genecards.org/cgi-bin/carddisp.pl?gene=LTA4H |
| PTHLH | 0.469714344 | https://www.genecards.org/cgi-bin/carddisp.pl?gene=PTHLH |
| PLA2G1B | 0.469714344 | https://www.genecards.org/cgi-bin/carddisp.pl?gene=PLA2G1B |
| NUMA1 | 0.469714344 | https://www.genecards.org/cgi-bin/carddisp.pl?gene=NUMA1 |
| DFFB | 0.469714344 | https://www.genecards.org/cgi-bin/carddisp.pl?gene=DFFB |
| IQGAP1 | 0.469714344 | https://www.genecards.org/cgi-bin/carddisp.pl?gene=IQGAP1 |
| P2RX1 | 0.469714344 | https://www.genecards.org/cgi-bin/carddisp.pl?gene=P2RX1 |
| PDE3B | 0.469714344 | https://www.genecards.org/cgi-bin/carddisp.pl?gene=PDE3B |
| ABCF1 | 0.469714344 | https://www.genecards.org/cgi-bin/carddisp.pl?gene=ABCF1 |
| SIRT4 | 0.469714344 | https://www.genecards.org/cgi-bin/carddisp.pl?gene=SIRT4 |
| CXCL3 | 0.469714344 | https://www.genecards.org/cgi-bin/carddisp.pl?gene=CXCL3 |
| CHAC1 | 0.469714344 | https://www.genecards.org/cgi-bin/carddisp.pl?gene=CHAC1 |
| MIR301A | 0.469714344 | https://www.genecards.org/cgi-bin/carddisp.pl?gene=MIR301A |
| MIR133A2 | 0.469714344 | https://www.genecards.org/cgi-bin/carddisp.pl?gene=MIR133A2 |
| MIR486-1 | 0.469714344 | https://www.genecards.org/cgi-bin/carddisp.pl?gene=MIR486-1 |
| MIR489 | 0.469714344 | https://www.genecards.org/cgi-bin/carddisp.pl?gene=MIR489 |
| MIR133A1 | 0.469714344 | https://www.genecards.org/cgi-bin/carddisp.pl?gene=MIR133A1 |
| MIR320B1 | 0.469714344 | https://www.genecards.org/cgi-bin/carddisp.pl?gene=MIR320B1 |
| MIR579 | 0.469714344 | https://www.genecards.org/cgi-bin/carddisp.pl?gene=MIR579 |
| AQP5-AS1 | 0.469714344 | https://www.genecards.org/cgi-bin/carddisp.pl?gene=AQP5-AS1 |
| MIR320B2 | 0.469714344 | https://www.genecards.org/cgi-bin/carddisp.pl?gene=MIR320B2 |
| MIR486-2 | 0.469714344 | https://www.genecards.org/cgi-bin/carddisp.pl?gene=MIR486-2 |
| ABCB1 | 0.469263822 | https://www.genecards.org/cgi-bin/carddisp.pl?gene=ABCB1 |
| MLH1 | 0.469263822 | https://www.genecards.org/cgi-bin/carddisp.pl?gene=MLH1 |
| CPE | 0.469263822 | https://www.genecards.org/cgi-bin/carddisp.pl?gene=CPE |
| HADHA | 0.469263822 | https://www.genecards.org/cgi-bin/carddisp.pl?gene=HADHA |
| SERPINH1 | 0.469263822 | https://www.genecards.org/cgi-bin/carddisp.pl?gene=SERPINH1 |
| SLC22A5 | 0.469263822 | https://www.genecards.org/cgi-bin/carddisp.pl?gene=SLC22A5 |
| DSC2 | 0.469263822 | https://www.genecards.org/cgi-bin/carddisp.pl?gene=DSC2 |
| DSG2 | 0.469263822 | https://www.genecards.org/cgi-bin/carddisp.pl?gene=DSG2 |
| CCR3 | 0.469263822 | https://www.genecards.org/cgi-bin/carddisp.pl?gene=CCR3 |
| IL23R | 0.469263822 | https://www.genecards.org/cgi-bin/carddisp.pl?gene=IL23R |
| KRT17 | 0.469263822 | https://www.genecards.org/cgi-bin/carddisp.pl?gene=KRT17 |
| MYO9B | 0.469263822 | https://www.genecards.org/cgi-bin/carddisp.pl?gene=MYO9B |
| SMAD7 | 0.469263822 | https://www.genecards.org/cgi-bin/carddisp.pl?gene=SMAD7 |
| TNFSF15 | 0.469263822 | https://www.genecards.org/cgi-bin/carddisp.pl?gene=TNFSF15 |
| DSC3 | 0.469263822 | https://www.genecards.org/cgi-bin/carddisp.pl?gene=DSC3 |
| GCNT2 | 0.469263822 | https://www.genecards.org/cgi-bin/carddisp.pl?gene=GCNT2 |
| MTM1 | 0.469263822 | https://www.genecards.org/cgi-bin/carddisp.pl?gene=MTM1 |
| TNNT1 | 0.469263822 | https://www.genecards.org/cgi-bin/carddisp.pl?gene=TNNT1 |
| CPB1 | 0.469263822 | https://www.genecards.org/cgi-bin/carddisp.pl?gene=CPB1 |
| CRYBA4 | 0.469263822 | https://www.genecards.org/cgi-bin/carddisp.pl?gene=CRYBA4 |
| CRYBB3 | 0.469263822 | https://www.genecards.org/cgi-bin/carddisp.pl?gene=CRYBB3 |
| GUK1 | 0.469263822 | https://www.genecards.org/cgi-bin/carddisp.pl?gene=GUK1 |
| KRT9 | 0.469263822 | https://www.genecards.org/cgi-bin/carddisp.pl?gene=KRT9 |
| ST6GALNAC1 | 0.469263822 | https://www.genecards.org/cgi-bin/carddisp.pl?gene=ST6GALNAC1 |
| BFSP2 | 0.469263822 | https://www.genecards.org/cgi-bin/carddisp.pl?gene=BFSP2 |
| CLC | 0.469263822 | https://www.genecards.org/cgi-bin/carddisp.pl?gene=CLC |
| CRYGS | 0.469263822 | https://www.genecards.org/cgi-bin/carddisp.pl?gene=CRYGS |
| PROP1 | 0.469263822 | https://www.genecards.org/cgi-bin/carddisp.pl?gene=PROP1 |
| REG4 | 0.469263822 | https://www.genecards.org/cgi-bin/carddisp.pl?gene=REG4 |
| UTRN | 0.469263822 | https://www.genecards.org/cgi-bin/carddisp.pl?gene=UTRN |
| BFSP1 | 0.469263822 | https://www.genecards.org/cgi-bin/carddisp.pl?gene=BFSP1 |
| IL4I1 | 0.469263822 | https://www.genecards.org/cgi-bin/carddisp.pl?gene=IL4I1 |
| IMPG2 | 0.469263822 | https://www.genecards.org/cgi-bin/carddisp.pl?gene=IMPG2 |
| BTNL2 | 0.469263822 | https://www.genecards.org/cgi-bin/carddisp.pl?gene=BTNL2 |
| CATSPER1 | 0.469263822 | https://www.genecards.org/cgi-bin/carddisp.pl?gene=CATSPER1 |
| CNMD | 0.469263822 | https://www.genecards.org/cgi-bin/carddisp.pl?gene=CNMD |
| DUSP19 | 0.469263822 | https://www.genecards.org/cgi-bin/carddisp.pl?gene=DUSP19 |
| KRT15 | 0.469263822 | https://www.genecards.org/cgi-bin/carddisp.pl?gene=KRT15 |
| MUC2 | 0.469263822 | https://www.genecards.org/cgi-bin/carddisp.pl?gene=MUC2 |
| OTOA | 0.469263822 | https://www.genecards.org/cgi-bin/carddisp.pl?gene=OTOA |
| SCIN | 0.469263822 | https://www.genecards.org/cgi-bin/carddisp.pl?gene=SCIN |
| SRA1 | 0.469263822 | https://www.genecards.org/cgi-bin/carddisp.pl?gene=SRA1 |
| ZNF23 | 0.469263822 | https://www.genecards.org/cgi-bin/carddisp.pl?gene=ZNF23 |
| LIM2 | 0.469263822 | https://www.genecards.org/cgi-bin/carddisp.pl?gene=LIM2 |
| LACC1 | 0.469263822 | https://www.genecards.org/cgi-bin/carddisp.pl?gene=LACC1 |
| SPRR1B | 0.469263822 | https://www.genecards.org/cgi-bin/carddisp.pl?gene=SPRR1B |
| UCN | 0.469263822 | https://www.genecards.org/cgi-bin/carddisp.pl?gene=UCN |
| ZBTB11 | 0.469263822 | https://www.genecards.org/cgi-bin/carddisp.pl?gene=ZBTB11 |
| INAVA | 0.469263822 | https://www.genecards.org/cgi-bin/carddisp.pl?gene=INAVA |
| SLC16A12 | 0.469263822 | https://www.genecards.org/cgi-bin/carddisp.pl?gene=SLC16A12 |
| EYS | 0.469263822 | https://www.genecards.org/cgi-bin/carddisp.pl?gene=EYS |
| ZFP90 | 0.469263822 | https://www.genecards.org/cgi-bin/carddisp.pl?gene=ZFP90 |
| MUC12 | 0.469263822 | https://www.genecards.org/cgi-bin/carddisp.pl?gene=MUC12 |
| ARMS2 | 0.469263822 | https://www.genecards.org/cgi-bin/carddisp.pl?gene=ARMS2 |
| SELENOO | 0.469263822 | https://www.genecards.org/cgi-bin/carddisp.pl?gene=SELENOO |
| TMEM114 | 0.469263822 | https://www.genecards.org/cgi-bin/carddisp.pl?gene=TMEM114 |
| LIM2-AS1 | 0.469263822 | https://www.genecards.org/cgi-bin/carddisp.pl?gene=LIM2-AS1 |
| CAMK2G | 0.429665029 | https://www.genecards.org/cgi-bin/carddisp.pl?gene=CAMK2G |
| PLA2G4A | 0.429665029 | https://www.genecards.org/cgi-bin/carddisp.pl?gene=PLA2G4A |
| PRKCE | 0.429665029 | https://www.genecards.org/cgi-bin/carddisp.pl?gene=PRKCE |
| SLC9A3 | 0.429665029 | https://www.genecards.org/cgi-bin/carddisp.pl?gene=SLC9A3 |
| PRKAA1 | 0.429665029 | https://www.genecards.org/cgi-bin/carddisp.pl?gene=PRKAA1 |
| RIPK2 | 0.429665029 | https://www.genecards.org/cgi-bin/carddisp.pl?gene=RIPK2 |
| STAT4 | 0.429665029 | https://www.genecards.org/cgi-bin/carddisp.pl?gene=STAT4 |
| TGM2 | 0.429665029 | https://www.genecards.org/cgi-bin/carddisp.pl?gene=TGM2 |
| CASP2 | 0.429665029 | https://www.genecards.org/cgi-bin/carddisp.pl?gene=CASP2 |
| PRDX2 | 0.429665029 | https://www.genecards.org/cgi-bin/carddisp.pl?gene=PRDX2 |
| TIE1 | 0.429665029 | https://www.genecards.org/cgi-bin/carddisp.pl?gene=TIE1 |
| CD74 | 0.429665029 | https://www.genecards.org/cgi-bin/carddisp.pl?gene=CD74 |
| TPP2 | 0.429665029 | https://www.genecards.org/cgi-bin/carddisp.pl?gene=TPP2 |
| JAM3 | 0.429665029 | https://www.genecards.org/cgi-bin/carddisp.pl?gene=JAM3 |
| NR4A3 | 0.429665029 | https://www.genecards.org/cgi-bin/carddisp.pl?gene=NR4A3 |
| PTPA | 0.429665029 | https://www.genecards.org/cgi-bin/carddisp.pl?gene=PTPA |
| IRAK2 | 0.429665029 | https://www.genecards.org/cgi-bin/carddisp.pl?gene=IRAK2 |
| LPCAT2 | 0.429665029 | https://www.genecards.org/cgi-bin/carddisp.pl?gene=LPCAT2 |
| MLKL | 0.429665029 | https://www.genecards.org/cgi-bin/carddisp.pl?gene=MLKL |
| RHOT1 | 0.429665029 | https://www.genecards.org/cgi-bin/carddisp.pl?gene=RHOT1 |
| IL22RA2 | 0.429665029 | https://www.genecards.org/cgi-bin/carddisp.pl?gene=IL22RA2 |
| RNF31 | 0.429665029 | https://www.genecards.org/cgi-bin/carddisp.pl?gene=RNF31 |
| ATG12 | 0.429665029 | https://www.genecards.org/cgi-bin/carddisp.pl?gene=ATG12 |
| FFAR4 | 0.429665029 | https://www.genecards.org/cgi-bin/carddisp.pl?gene=FFAR4 |
| NCR3 | 0.429665029 | https://www.genecards.org/cgi-bin/carddisp.pl?gene=NCR3 |
| TRIM22 | 0.429665029 | https://www.genecards.org/cgi-bin/carddisp.pl?gene=TRIM22 |
| SHARPIN | 0.429665029 | https://www.genecards.org/cgi-bin/carddisp.pl?gene=SHARPIN |
| TNNI1 | 0.429665029 | https://www.genecards.org/cgi-bin/carddisp.pl?gene=TNNI1 |
| CHCHD2 | 0.429665029 | https://www.genecards.org/cgi-bin/carddisp.pl?gene=CHCHD2 |
| MIOX | 0.429665029 | https://www.genecards.org/cgi-bin/carddisp.pl?gene=MIOX |
| PLA2G12B | 0.429665029 | https://www.genecards.org/cgi-bin/carddisp.pl?gene=PLA2G12B |
| REG3A | 0.429665029 | https://www.genecards.org/cgi-bin/carddisp.pl?gene=REG3A |
| ISM1 | 0.429665029 | https://www.genecards.org/cgi-bin/carddisp.pl?gene=ISM1 |
| NFKBIZ | 0.429665029 | https://www.genecards.org/cgi-bin/carddisp.pl?gene=NFKBIZ |
| PPARGC1B | 0.429665029 | https://www.genecards.org/cgi-bin/carddisp.pl?gene=PPARGC1B |
| NCR3LG1 | 0.429665029 | https://www.genecards.org/cgi-bin/carddisp.pl?gene=NCR3LG1 |
| IRX2-DT | 0.429665029 | https://www.genecards.org/cgi-bin/carddisp.pl?gene=IRX2-DT |
| MIR18A | 0.429665029 | https://www.genecards.org/cgi-bin/carddisp.pl?gene=MIR18A |
| MIR424 | 0.429665029 | https://www.genecards.org/cgi-bin/carddisp.pl?gene=MIR424 |
| MIR29B2 | 0.429665029 | https://www.genecards.org/cgi-bin/carddisp.pl?gene=MIR29B2 |
| MIR501 | 0.429665029 | https://www.genecards.org/cgi-bin/carddisp.pl?gene=MIR501 |
| MIR942 | 0.429665029 | https://www.genecards.org/cgi-bin/carddisp.pl?gene=MIR942 |
| SREBF2-AS1 | 0.429665029 | https://www.genecards.org/cgi-bin/carddisp.pl?gene=SREBF2-AS1 |
| TP53COR1 | 0.429665029 | https://www.genecards.org/cgi-bin/carddisp.pl?gene=TP53COR1 |
| TGFB2 | 0.377471745 | https://www.genecards.org/cgi-bin/carddisp.pl?gene=TGFB2 |
| ATP1A1 | 0.377471745 | https://www.genecards.org/cgi-bin/carddisp.pl?gene=ATP1A1 |
| DAPK1 | 0.377471745 | https://www.genecards.org/cgi-bin/carddisp.pl?gene=DAPK1 |
| REN | 0.377471745 | https://www.genecards.org/cgi-bin/carddisp.pl?gene=REN |
| SCNN1A | 0.377471745 | https://www.genecards.org/cgi-bin/carddisp.pl?gene=SCNN1A |
| CYLD | 0.377471745 | https://www.genecards.org/cgi-bin/carddisp.pl?gene=CYLD |
| ESRRB | 0.377471745 | https://www.genecards.org/cgi-bin/carddisp.pl?gene=ESRRB |
| ALPI | 0.377471745 | https://www.genecards.org/cgi-bin/carddisp.pl?gene=ALPI |
| LIPE | 0.377471745 | https://www.genecards.org/cgi-bin/carddisp.pl?gene=LIPE |
| PC | 0.377471745 | https://www.genecards.org/cgi-bin/carddisp.pl?gene=PC |
| PIN1 | 0.377471745 | https://www.genecards.org/cgi-bin/carddisp.pl?gene=PIN1 |
| WARS1 | 0.377471745 | https://www.genecards.org/cgi-bin/carddisp.pl?gene=WARS1 |
| MAP3K8 | 0.377471745 | https://www.genecards.org/cgi-bin/carddisp.pl?gene=MAP3K8 |
| RAB11A | 0.377471745 | https://www.genecards.org/cgi-bin/carddisp.pl?gene=RAB11A |
| APOA2 | 0.377471745 | https://www.genecards.org/cgi-bin/carddisp.pl?gene=APOA2 |
| AQP3 | 0.377471745 | https://www.genecards.org/cgi-bin/carddisp.pl?gene=AQP3 |
| BTRC | 0.377471745 | https://www.genecards.org/cgi-bin/carddisp.pl?gene=BTRC |
| CD2 | 0.377471745 | https://www.genecards.org/cgi-bin/carddisp.pl?gene=CD2 |
| AKR1B10 | 0.377471745 | https://www.genecards.org/cgi-bin/carddisp.pl?gene=AKR1B10 |
| RBX1 | 0.377471745 | https://www.genecards.org/cgi-bin/carddisp.pl?gene=RBX1 |
| TYROBP | 0.377471745 | https://www.genecards.org/cgi-bin/carddisp.pl?gene=TYROBP |
| UBC | 0.377471745 | https://www.genecards.org/cgi-bin/carddisp.pl?gene=UBC |
| AASS | 0.377471745 | https://www.genecards.org/cgi-bin/carddisp.pl?gene=AASS |
| TRIM63 | 0.377471745 | https://www.genecards.org/cgi-bin/carddisp.pl?gene=TRIM63 |
| UBE2G1 | 0.377471745 | https://www.genecards.org/cgi-bin/carddisp.pl?gene=UBE2G1 |
| NR0B2 | 0.377471745 | https://www.genecards.org/cgi-bin/carddisp.pl?gene=NR0B2 |
| PIEZO1 | 0.377471745 | https://www.genecards.org/cgi-bin/carddisp.pl?gene=PIEZO1 |
| SLC5A4 | 0.377471745 | https://www.genecards.org/cgi-bin/carddisp.pl?gene=SLC5A4 |
| UBE2G2 | 0.377471745 | https://www.genecards.org/cgi-bin/carddisp.pl?gene=UBE2G2 |
| YBX1 | 0.377471745 | https://www.genecards.org/cgi-bin/carddisp.pl?gene=YBX1 |
| KCNH4 | 0.377471745 | https://www.genecards.org/cgi-bin/carddisp.pl?gene=KCNH4 |
| NAPRT | 0.377471745 | https://www.genecards.org/cgi-bin/carddisp.pl?gene=NAPRT |
| ITLN1 | 0.377471745 | https://www.genecards.org/cgi-bin/carddisp.pl?gene=ITLN1 |
| NAIP | 0.377471745 | https://www.genecards.org/cgi-bin/carddisp.pl?gene=NAIP |
| CPQ | 0.377471745 | https://www.genecards.org/cgi-bin/carddisp.pl?gene=CPQ |
| CST7 | 0.377471745 | https://www.genecards.org/cgi-bin/carddisp.pl?gene=CST7 |
| FBXO3 | 0.377471745 | https://www.genecards.org/cgi-bin/carddisp.pl?gene=FBXO3 |
| PPIL3 | 0.377471745 | https://www.genecards.org/cgi-bin/carddisp.pl?gene=PPIL3 |
| CD300LB | 0.377471745 | https://www.genecards.org/cgi-bin/carddisp.pl?gene=CD300LB |
| LPCAT1 | 0.377471745 | https://www.genecards.org/cgi-bin/carddisp.pl?gene=LPCAT1 |
| MIR107 | 0.377471745 | https://www.genecards.org/cgi-bin/carddisp.pl?gene=MIR107 |
| MIR24-2 | 0.377471745 | https://www.genecards.org/cgi-bin/carddisp.pl?gene=MIR24-2 |
| MIR130B | 0.377471745 | https://www.genecards.org/cgi-bin/carddisp.pl?gene=MIR130B |
| SNORD50A | 0.377471745 | https://www.genecards.org/cgi-bin/carddisp.pl?gene=SNORD50A |
| MIR1260A | 0.377471745 | https://www.genecards.org/cgi-bin/carddisp.pl?gene=MIR1260A |
| MIR1285-1 | 0.377471745 | https://www.genecards.org/cgi-bin/carddisp.pl?gene=MIR1285-1 |
| MIR4454 | 0.377471745 | https://www.genecards.org/cgi-bin/carddisp.pl?gene=MIR4454 |
| MIR7975 | 0.377471745 | https://www.genecards.org/cgi-bin/carddisp.pl?gene=MIR7975 |
| ATM | 0.36881876 | https://www.genecards.org/cgi-bin/carddisp.pl?gene=ATM |
| HPRT1 | 0.36881876 | https://www.genecards.org/cgi-bin/carddisp.pl?gene=HPRT1 |
| CTPS1 | 0.36881876 | https://www.genecards.org/cgi-bin/carddisp.pl?gene=CTPS1 |
| PCYT1A | 0.36881876 | https://www.genecards.org/cgi-bin/carddisp.pl?gene=PCYT1A |
| RASGRP1 | 0.36881876 | https://www.genecards.org/cgi-bin/carddisp.pl?gene=RASGRP1 |
| TNK2 | 0.36881876 | https://www.genecards.org/cgi-bin/carddisp.pl?gene=TNK2 |
| UNG | 0.36881876 | https://www.genecards.org/cgi-bin/carddisp.pl?gene=UNG |
| XRCC6 | 0.36881876 | https://www.genecards.org/cgi-bin/carddisp.pl?gene=XRCC6 |
| TNFRSF4 | 0.36881876 | https://www.genecards.org/cgi-bin/carddisp.pl?gene=TNFRSF4 |
| TNFRSF9 | 0.36881876 | https://www.genecards.org/cgi-bin/carddisp.pl?gene=TNFRSF9 |
| XRCC5 | 0.36881876 | https://www.genecards.org/cgi-bin/carddisp.pl?gene=XRCC5 |
| CD70 | 0.36881876 | https://www.genecards.org/cgi-bin/carddisp.pl?gene=CD70 |
| DLG3 | 0.36881876 | https://www.genecards.org/cgi-bin/carddisp.pl?gene=DLG3 |
| IL1RL2 | 0.36881876 | https://www.genecards.org/cgi-bin/carddisp.pl?gene=IL1RL2 |
| MAP3K9 | 0.36881876 | https://www.genecards.org/cgi-bin/carddisp.pl?gene=MAP3K9 |
| RNF168 | 0.36881876 | https://www.genecards.org/cgi-bin/carddisp.pl?gene=RNF168 |
| CARD14 | 0.36881876 | https://www.genecards.org/cgi-bin/carddisp.pl?gene=CARD14 |
| SLAMF1 | 0.36881876 | https://www.genecards.org/cgi-bin/carddisp.pl?gene=SLAMF1 |
| STX11 | 0.36881876 | https://www.genecards.org/cgi-bin/carddisp.pl?gene=STX11 |
| WDR1 | 0.36881876 | https://www.genecards.org/cgi-bin/carddisp.pl?gene=WDR1 |
| EN1 | 0.36881876 | https://www.genecards.org/cgi-bin/carddisp.pl?gene=EN1 |
| NCKAP1L | 0.36881876 | https://www.genecards.org/cgi-bin/carddisp.pl?gene=NCKAP1L |
| ZFYVE16 | 0.36881876 | https://www.genecards.org/cgi-bin/carddisp.pl?gene=ZFYVE16 |
| SLAMF6 | 0.36881876 | https://www.genecards.org/cgi-bin/carddisp.pl?gene=SLAMF6 |
| TNIP1 | 0.36881876 | https://www.genecards.org/cgi-bin/carddisp.pl?gene=TNIP1 |
| APOBEC1 | 0.36881876 | https://www.genecards.org/cgi-bin/carddisp.pl?gene=APOBEC1 |
| IL1F10 | 0.36881876 | https://www.genecards.org/cgi-bin/carddisp.pl?gene=IL1F10 |
| IL36G | 0.36881876 | https://www.genecards.org/cgi-bin/carddisp.pl?gene=IL36G |
| SLC51A | 0.36881876 | https://www.genecards.org/cgi-bin/carddisp.pl?gene=SLC51A |
| FBLIM1 | 0.36881876 | https://www.genecards.org/cgi-bin/carddisp.pl?gene=FBLIM1 |
| IL36A | 0.36881876 | https://www.genecards.org/cgi-bin/carddisp.pl?gene=IL36A |
| MPP3 | 0.36881876 | https://www.genecards.org/cgi-bin/carddisp.pl?gene=MPP3 |
| DYNLT2B | 0.36881876 | https://www.genecards.org/cgi-bin/carddisp.pl?gene=DYNLT2B |
| NRROS | 0.36881876 | https://www.genecards.org/cgi-bin/carddisp.pl?gene=NRROS |
| ZDHHC19 | 0.36881876 | https://www.genecards.org/cgi-bin/carddisp.pl?gene=ZDHHC19 |
| CARMIL2 | 0.36881876 | https://www.genecards.org/cgi-bin/carddisp.pl?gene=CARMIL2 |
| CEP19 | 0.36881876 | https://www.genecards.org/cgi-bin/carddisp.pl?gene=CEP19 |
| IL36B | 0.36881876 | https://www.genecards.org/cgi-bin/carddisp.pl?gene=IL36B |
| FBXO45 | 0.36881876 | https://www.genecards.org/cgi-bin/carddisp.pl?gene=FBXO45 |
| UBXN7 | 0.36881876 | https://www.genecards.org/cgi-bin/carddisp.pl?gene=UBXN7 |
| WDR53 | 0.36881876 | https://www.genecards.org/cgi-bin/carddisp.pl?gene=WDR53 |
| PIH1D2 | 0.36881876 | https://www.genecards.org/cgi-bin/carddisp.pl?gene=PIH1D2 |
| TM4SF19 | 0.36881876 | https://www.genecards.org/cgi-bin/carddisp.pl?gene=TM4SF19 |
| PAXX | 0.36881876 | https://www.genecards.org/cgi-bin/carddisp.pl?gene=PAXX |
| SMCO1 | 0.36881876 | https://www.genecards.org/cgi-bin/carddisp.pl?gene=SMCO1 |
| LCE3C | 0.36881876 | https://www.genecards.org/cgi-bin/carddisp.pl?gene=LCE3C |
| C1orf141 | 0.36881876 | https://www.genecards.org/cgi-bin/carddisp.pl?gene=C1orf141 |
| LCE3B | 0.36881876 | https://www.genecards.org/cgi-bin/carddisp.pl?gene=LCE3B |
| RASGRF2-AS1 | 0.356398046 | https://www.genecards.org/cgi-bin/carddisp.pl?gene=RASGRF2-AS1 |
| DEFB114 | 0.355626643 | https://www.genecards.org/cgi-bin/carddisp.pl?gene=DEFB114 |
| PTGS2 | 0.333379835 | https://www.genecards.org/cgi-bin/carddisp.pl?gene=PTGS2 |
| MAP3K5 | 0.333379835 | https://www.genecards.org/cgi-bin/carddisp.pl?gene=MAP3K5 |
| ITGB3 | 0.308649749 | https://www.genecards.org/cgi-bin/carddisp.pl?gene=ITGB3 |
| ABCC8 | 0.308649749 | https://www.genecards.org/cgi-bin/carddisp.pl?gene=ABCC8 |
| PSMA3 | 0.308649749 | https://www.genecards.org/cgi-bin/carddisp.pl?gene=PSMA3 |
| NLRP1 | 0.308649749 | https://www.genecards.org/cgi-bin/carddisp.pl?gene=NLRP1 |
| NLRP12 | 0.308649749 | https://www.genecards.org/cgi-bin/carddisp.pl?gene=NLRP12 |
| ZDHHC6 | 0.308649749 | https://www.genecards.org/cgi-bin/carddisp.pl?gene=ZDHHC6 |
| LOC106627981 | 0.308649749 | https://www.genecards.org/cgi-bin/carddisp.pl?gene=LOC106627981 |
| PIK3CA | 0.281757385 | https://www.genecards.org/cgi-bin/carddisp.pl?gene=PIK3CA |
| ATF2 | 0.281757385 | https://www.genecards.org/cgi-bin/carddisp.pl?gene=ATF2 |
| BRCA2 | 0.281757385 | https://www.genecards.org/cgi-bin/carddisp.pl?gene=BRCA2 |
| PTGER4 | 0.281757385 | https://www.genecards.org/cgi-bin/carddisp.pl?gene=PTGER4 |
| ABCB7 | 0.281757385 | https://www.genecards.org/cgi-bin/carddisp.pl?gene=ABCB7 |
| ELK1 | 0.281757385 | https://www.genecards.org/cgi-bin/carddisp.pl?gene=ELK1 |
| KCNJ8 | 0.281757385 | https://www.genecards.org/cgi-bin/carddisp.pl?gene=KCNJ8 |
| ANKRD36 | 0.281757385 | https://www.genecards.org/cgi-bin/carddisp.pl?gene=ANKRD36 |
| CREBBP | 0.252011478 | https://www.genecards.org/cgi-bin/carddisp.pl?gene=CREBBP |
| TNFSF11 | 0.252011478 | https://www.genecards.org/cgi-bin/carddisp.pl?gene=TNFSF11 |
| ITGAV | 0.252011478 | https://www.genecards.org/cgi-bin/carddisp.pl?gene=ITGAV |
| TAF1 | 0.252011478 | https://www.genecards.org/cgi-bin/carddisp.pl?gene=TAF1 |
| LBR | 0.252011478 | https://www.genecards.org/cgi-bin/carddisp.pl?gene=LBR |
| ASL | 0.252011478 | https://www.genecards.org/cgi-bin/carddisp.pl?gene=ASL |
| CTNND1 | 0.252011478 | https://www.genecards.org/cgi-bin/carddisp.pl?gene=CTNND1 |
| FOSL1 | 0.252011478 | https://www.genecards.org/cgi-bin/carddisp.pl?gene=FOSL1 |
| BCL10 | 0.252011478 | https://www.genecards.org/cgi-bin/carddisp.pl?gene=BCL10 |
| SLC39A8 | 0.252011478 | https://www.genecards.org/cgi-bin/carddisp.pl?gene=SLC39A8 |
| DDAH1 | 0.252011478 | https://www.genecards.org/cgi-bin/carddisp.pl?gene=DDAH1 |
| SHBG | 0.252011478 | https://www.genecards.org/cgi-bin/carddisp.pl?gene=SHBG |
| MRC1 | 0.252011478 | https://www.genecards.org/cgi-bin/carddisp.pl?gene=MRC1 |
| ZPR1 | 0.252011478 | https://www.genecards.org/cgi-bin/carddisp.pl?gene=ZPR1 |
| HSPB6 | 0.252011478 | https://www.genecards.org/cgi-bin/carddisp.pl?gene=HSPB6 |
| OXT | 0.252011478 | https://www.genecards.org/cgi-bin/carddisp.pl?gene=OXT |
| PJA2 | 0.252011478 | https://www.genecards.org/cgi-bin/carddisp.pl?gene=PJA2 |
| RMST | 0.252011478 | https://www.genecards.org/cgi-bin/carddisp.pl?gene=RMST |
| IFNAR2-IL10RB | 0.252011478 | https://www.genecards.org/cgi-bin/carddisp.pl?gene=IFNAR2-IL10RB |
| CACNA1C | 0.251466006 | https://www.genecards.org/cgi-bin/carddisp.pl?gene=CACNA1C |
| FZD4 | 0.251466006 | https://www.genecards.org/cgi-bin/carddisp.pl?gene=FZD4 |
| KDM1A | 0.251466006 | https://www.genecards.org/cgi-bin/carddisp.pl?gene=KDM1A |
| BIRC2 | 0.251466006 | https://www.genecards.org/cgi-bin/carddisp.pl?gene=BIRC2 |
| CLU | 0.251466006 | https://www.genecards.org/cgi-bin/carddisp.pl?gene=CLU |
| PNLIP | 0.251466006 | https://www.genecards.org/cgi-bin/carddisp.pl?gene=PNLIP |
| SGPL1 | 0.251466006 | https://www.genecards.org/cgi-bin/carddisp.pl?gene=SGPL1 |
| CKM | 0.251466006 | https://www.genecards.org/cgi-bin/carddisp.pl?gene=CKM |
| SLC1A4 | 0.251466006 | https://www.genecards.org/cgi-bin/carddisp.pl?gene=SLC1A4 |
| AARS1 | 0.251466006 | https://www.genecards.org/cgi-bin/carddisp.pl?gene=AARS1 |
| DNASE1 | 0.251466006 | https://www.genecards.org/cgi-bin/carddisp.pl?gene=DNASE1 |
| VDAC3 | 0.251466006 | https://www.genecards.org/cgi-bin/carddisp.pl?gene=VDAC3 |
| RHOH | 0.251466006 | https://www.genecards.org/cgi-bin/carddisp.pl?gene=RHOH |
| STK26 | 0.251466006 | https://www.genecards.org/cgi-bin/carddisp.pl?gene=STK26 |
| PELI1 | 0.251466006 | https://www.genecards.org/cgi-bin/carddisp.pl?gene=PELI1 |
| BMPER | 0.251466006 | https://www.genecards.org/cgi-bin/carddisp.pl?gene=BMPER |
| CFHR3 | 0.251466006 | https://www.genecards.org/cgi-bin/carddisp.pl?gene=CFHR3 |
| SLC39A10 | 0.251466006 | https://www.genecards.org/cgi-bin/carddisp.pl?gene=SLC39A10 |
| H1-0 | 0.251466006 | https://www.genecards.org/cgi-bin/carddisp.pl?gene=H1-0 |
| H1-5 | 0.251466006 | https://www.genecards.org/cgi-bin/carddisp.pl?gene=H1-5 |
| MICA | 0.251466006 | https://www.genecards.org/cgi-bin/carddisp.pl?gene=MICA |
| RNF41 | 0.251466006 | https://www.genecards.org/cgi-bin/carddisp.pl?gene=RNF41 |
| USP19 | 0.251466006 | https://www.genecards.org/cgi-bin/carddisp.pl?gene=USP19 |
| COX7C | 0.251466006 | https://www.genecards.org/cgi-bin/carddisp.pl?gene=COX7C |
| TIMMDC1 | 0.251466006 | https://www.genecards.org/cgi-bin/carddisp.pl?gene=TIMMDC1 |
| CARD16 | 0.251466006 | https://www.genecards.org/cgi-bin/carddisp.pl?gene=CARD16 |
| NDUFB5 | 0.251466006 | https://www.genecards.org/cgi-bin/carddisp.pl?gene=NDUFB5 |
| PDF | 0.251466006 | https://www.genecards.org/cgi-bin/carddisp.pl?gene=PDF |
| CALML6 | 0.251466006 | https://www.genecards.org/cgi-bin/carddisp.pl?gene=CALML6 |
| BCL2L15 | 0.251466006 | https://www.genecards.org/cgi-bin/carddisp.pl?gene=BCL2L15 |
| ST20 | 0.251466006 | https://www.genecards.org/cgi-bin/carddisp.pl?gene=ST20 |
| RNY3 | 0.251466006 | https://www.genecards.org/cgi-bin/carddisp.pl?gene=RNY3 |
| LOC107980440 | 0.251466006 | https://www.genecards.org/cgi-bin/carddisp.pl?gene=LOC107980440 |
| LOC107966121 | 0.251466006 | https://www.genecards.org/cgi-bin/carddisp.pl?gene=LOC107966121 |
| ABL1 | 0.218248338 | https://www.genecards.org/cgi-bin/carddisp.pl?gene=ABL1 |
| FOS | 0.218248338 | https://www.genecards.org/cgi-bin/carddisp.pl?gene=FOS |
| VIM | 0.218248338 | https://www.genecards.org/cgi-bin/carddisp.pl?gene=VIM |
| ASS1 | 0.218248338 | https://www.genecards.org/cgi-bin/carddisp.pl?gene=ASS1 |
| FBP1 | 0.218248338 | https://www.genecards.org/cgi-bin/carddisp.pl?gene=FBP1 |
| TOP1 | 0.218248338 | https://www.genecards.org/cgi-bin/carddisp.pl?gene=TOP1 |
| ADRB1 | 0.218248338 | https://www.genecards.org/cgi-bin/carddisp.pl?gene=ADRB1 |
| ERCC2 | 0.218248338 | https://www.genecards.org/cgi-bin/carddisp.pl?gene=ERCC2 |
| GCH1 | 0.218248338 | https://www.genecards.org/cgi-bin/carddisp.pl?gene=GCH1 |
| FGR | 0.218248338 | https://www.genecards.org/cgi-bin/carddisp.pl?gene=FGR |
| PTGS1 | 0.218248338 | https://www.genecards.org/cgi-bin/carddisp.pl?gene=PTGS1 |
| TNFRSF11A | 0.218248338 | https://www.genecards.org/cgi-bin/carddisp.pl?gene=TNFRSF11A |
| ARHGEF2 | 0.218248338 | https://www.genecards.org/cgi-bin/carddisp.pl?gene=ARHGEF2 |
| ITPR3 | 0.218248338 | https://www.genecards.org/cgi-bin/carddisp.pl?gene=ITPR3 |
| MYLK2 | 0.218248338 | https://www.genecards.org/cgi-bin/carddisp.pl?gene=MYLK2 |
| TKT | 0.218248338 | https://www.genecards.org/cgi-bin/carddisp.pl?gene=TKT |
| GCLC | 0.218248338 | https://www.genecards.org/cgi-bin/carddisp.pl?gene=GCLC |
| TRAF2 | 0.218248338 | https://www.genecards.org/cgi-bin/carddisp.pl?gene=TRAF2 |
| ADD1 | 0.218248338 | https://www.genecards.org/cgi-bin/carddisp.pl?gene=ADD1 |
| ALOX5AP | 0.218248338 | https://www.genecards.org/cgi-bin/carddisp.pl?gene=ALOX5AP |
| DPAGT1 | 0.218248338 | https://www.genecards.org/cgi-bin/carddisp.pl?gene=DPAGT1 |
| SERPINA5 | 0.218248338 | https://www.genecards.org/cgi-bin/carddisp.pl?gene=SERPINA5 |
| XRCC1 | 0.218248338 | https://www.genecards.org/cgi-bin/carddisp.pl?gene=XRCC1 |
| ADAMTS2 | 0.218248338 | https://www.genecards.org/cgi-bin/carddisp.pl?gene=ADAMTS2 |
| ARF6 | 0.218248338 | https://www.genecards.org/cgi-bin/carddisp.pl?gene=ARF6 |
| GGH | 0.218248338 | https://www.genecards.org/cgi-bin/carddisp.pl?gene=GGH |
| GCG | 0.218248338 | https://www.genecards.org/cgi-bin/carddisp.pl?gene=GCG |
| GCLM | 0.218248338 | https://www.genecards.org/cgi-bin/carddisp.pl?gene=GCLM |
| OLR1 | 0.218248338 | https://www.genecards.org/cgi-bin/carddisp.pl?gene=OLR1 |
| XRCC3 | 0.218248338 | https://www.genecards.org/cgi-bin/carddisp.pl?gene=XRCC3 |
| BIRC6 | 0.218248338 | https://www.genecards.org/cgi-bin/carddisp.pl?gene=BIRC6 |
| FLT3LG | 0.218248338 | https://www.genecards.org/cgi-bin/carddisp.pl?gene=FLT3LG |
| PGLYRP1 | 0.218248338 | https://www.genecards.org/cgi-bin/carddisp.pl?gene=PGLYRP1 |
| TRIM27 | 0.218248338 | https://www.genecards.org/cgi-bin/carddisp.pl?gene=TRIM27 |
| OTUD5 | 0.218248338 | https://www.genecards.org/cgi-bin/carddisp.pl?gene=OTUD5 |
| IFI35 | 0.218248338 | https://www.genecards.org/cgi-bin/carddisp.pl?gene=IFI35 |
| TIMD4 | 0.218248338 | https://www.genecards.org/cgi-bin/carddisp.pl?gene=TIMD4 |
| TJP3 | 0.218248338 | https://www.genecards.org/cgi-bin/carddisp.pl?gene=TJP3 |
| INTS11 | 0.218248338 | https://www.genecards.org/cgi-bin/carddisp.pl?gene=INTS11 |
| NKX3-2 | 0.218248338 | https://www.genecards.org/cgi-bin/carddisp.pl?gene=NKX3-2 |
| SLC25A38 | 0.218248338 | https://www.genecards.org/cgi-bin/carddisp.pl?gene=SLC25A38 |
| MRAP | 0.218248338 | https://www.genecards.org/cgi-bin/carddisp.pl?gene=MRAP |
| ZNF318 | 0.218248338 | https://www.genecards.org/cgi-bin/carddisp.pl?gene=ZNF318 |
| DEFB103B | 0.218248338 | https://www.genecards.org/cgi-bin/carddisp.pl?gene=DEFB103B |
| C3orf36 | 0.218248338 | https://www.genecards.org/cgi-bin/carddisp.pl?gene=C3orf36 |
| ARRDC1-AS1 | 0.218248338 | https://www.genecards.org/cgi-bin/carddisp.pl?gene=ARRDC1-AS1 |
| LINC02915 | 0.218248338 | https://www.genecards.org/cgi-bin/carddisp.pl?gene=LINC02915 |
| DLX6-AS1 | 0.218248338 | https://www.genecards.org/cgi-bin/carddisp.pl?gene=DLX6-AS1 |
| MIR208B | 0.218248338 | https://www.genecards.org/cgi-bin/carddisp.pl?gene=MIR208B |
| LINC01556 | 0.218248338 | https://www.genecards.org/cgi-bin/carddisp.pl?gene=LINC01556 |
| MIR429 | 0.218248338 | https://www.genecards.org/cgi-bin/carddisp.pl?gene=MIR429 |
| MIR101-1 | 0.218248338 | https://www.genecards.org/cgi-bin/carddisp.pl?gene=MIR101-1 |
| LINC02897 | 0.218248338 | https://www.genecards.org/cgi-bin/carddisp.pl?gene=LINC02897 |
| MIR202 | 0.218248338 | https://www.genecards.org/cgi-bin/carddisp.pl?gene=MIR202 |
| MIR101-2 | 0.218248338 | https://www.genecards.org/cgi-bin/carddisp.pl?gene=MIR101-2 |
| MIR320C1 | 0.218248338 | https://www.genecards.org/cgi-bin/carddisp.pl?gene=MIR320C1 |
| MIR887 | 0.218248338 | https://www.genecards.org/cgi-bin/carddisp.pl?gene=MIR887 |
| MIR320C2 | 0.218248338 | https://www.genecards.org/cgi-bin/carddisp.pl?gene=MIR320C2 |
| CCL15-CCL14 | 0.218248338 | https://www.genecards.org/cgi-bin/carddisp.pl?gene=CCL15-CCL14 |
| ENSG00000228741 | 0.218248338 | https://www.genecards.org/cgi-bin/carddisp.pl?gene=ENSG00000228741 |
| RAF1 | 0.178199023 | https://www.genecards.org/cgi-bin/carddisp.pl?gene=RAF1 |
| CSF1R | 0.178199023 | https://www.genecards.org/cgi-bin/carddisp.pl?gene=CSF1R |
| EP300 | 0.178199023 | https://www.genecards.org/cgi-bin/carddisp.pl?gene=EP300 |
| PRKACA | 0.178199023 | https://www.genecards.org/cgi-bin/carddisp.pl?gene=PRKACA |
| SNCA | 0.178199023 | https://www.genecards.org/cgi-bin/carddisp.pl?gene=SNCA |
| ACVRL1 | 0.178199023 | https://www.genecards.org/cgi-bin/carddisp.pl?gene=ACVRL1 |
| IL6ST | 0.178199023 | https://www.genecards.org/cgi-bin/carddisp.pl?gene=IL6ST |
| JAG1 | 0.178199023 | https://www.genecards.org/cgi-bin/carddisp.pl?gene=JAG1 |
| PAK1 | 0.178199023 | https://www.genecards.org/cgi-bin/carddisp.pl?gene=PAK1 |
| TBXA2R | 0.178199023 | https://www.genecards.org/cgi-bin/carddisp.pl?gene=TBXA2R |
| TYMS | 0.178199023 | https://www.genecards.org/cgi-bin/carddisp.pl?gene=TYMS |
| AGT | 0.178199023 | https://www.genecards.org/cgi-bin/carddisp.pl?gene=AGT |
| ARG1 | 0.178199023 | https://www.genecards.org/cgi-bin/carddisp.pl?gene=ARG1 |
| CASP7 | 0.178199023 | https://www.genecards.org/cgi-bin/carddisp.pl?gene=CASP7 |
| DNM2 | 0.178199023 | https://www.genecards.org/cgi-bin/carddisp.pl?gene=DNM2 |
| GAA | 0.178199023 | https://www.genecards.org/cgi-bin/carddisp.pl?gene=GAA |
| PRKCQ | 0.178199023 | https://www.genecards.org/cgi-bin/carddisp.pl?gene=PRKCQ |
| RUNX1 | 0.178199023 | https://www.genecards.org/cgi-bin/carddisp.pl?gene=RUNX1 |
| TBP | 0.178199023 | https://www.genecards.org/cgi-bin/carddisp.pl?gene=TBP |
| ADCY1 | 0.178199023 | https://www.genecards.org/cgi-bin/carddisp.pl?gene=ADCY1 |
| ALDH7A1 | 0.178199023 | https://www.genecards.org/cgi-bin/carddisp.pl?gene=ALDH7A1 |
| ALDOA | 0.178199023 | https://www.genecards.org/cgi-bin/carddisp.pl?gene=ALDOA |
| CASP10 | 0.178199023 | https://www.genecards.org/cgi-bin/carddisp.pl?gene=CASP10 |
| DUSP6 | 0.178199023 | https://www.genecards.org/cgi-bin/carddisp.pl?gene=DUSP6 |
| NBN | 0.178199023 | https://www.genecards.org/cgi-bin/carddisp.pl?gene=NBN |
| NR1H4 | 0.178199023 | https://www.genecards.org/cgi-bin/carddisp.pl?gene=NR1H4 |
| PDE3A | 0.178199023 | https://www.genecards.org/cgi-bin/carddisp.pl?gene=PDE3A |
| PTK2 | 0.178199023 | https://www.genecards.org/cgi-bin/carddisp.pl?gene=PTK2 |
| RAB27A | 0.178199023 | https://www.genecards.org/cgi-bin/carddisp.pl?gene=RAB27A |
| DHCR7 | 0.178199023 | https://www.genecards.org/cgi-bin/carddisp.pl?gene=DHCR7 |
| DLL4 | 0.178199023 | https://www.genecards.org/cgi-bin/carddisp.pl?gene=DLL4 |
| GLDC | 0.178199023 | https://www.genecards.org/cgi-bin/carddisp.pl?gene=GLDC |
| KCNQ2 | 0.178199023 | https://www.genecards.org/cgi-bin/carddisp.pl?gene=KCNQ2 |
| KLK3 | 0.178199023 | https://www.genecards.org/cgi-bin/carddisp.pl?gene=KLK3 |
| MYH7 | 0.178199023 | https://www.genecards.org/cgi-bin/carddisp.pl?gene=MYH7 |
| FADD | 0.178199023 | https://www.genecards.org/cgi-bin/carddisp.pl?gene=FADD |
| FPR2 | 0.178199023 | https://www.genecards.org/cgi-bin/carddisp.pl?gene=FPR2 |
| GCDH | 0.178199023 | https://www.genecards.org/cgi-bin/carddisp.pl?gene=GCDH |
| KCNJ11 | 0.178199023 | https://www.genecards.org/cgi-bin/carddisp.pl?gene=KCNJ11 |
| KLF5 | 0.178199023 | https://www.genecards.org/cgi-bin/carddisp.pl?gene=KLF5 |
| NR1H2 | 0.178199023 | https://www.genecards.org/cgi-bin/carddisp.pl?gene=NR1H2 |
| OPRL1 | 0.178199023 | https://www.genecards.org/cgi-bin/carddisp.pl?gene=OPRL1 |
| VDAC1 | 0.178199023 | https://www.genecards.org/cgi-bin/carddisp.pl?gene=VDAC1 |
| ABL2 | 0.178199023 | https://www.genecards.org/cgi-bin/carddisp.pl?gene=ABL2 |
| CLCN3 | 0.178199023 | https://www.genecards.org/cgi-bin/carddisp.pl?gene=CLCN3 |
| LAMP2 | 0.178199023 | https://www.genecards.org/cgi-bin/carddisp.pl?gene=LAMP2 |
| AMBP | 0.178199023 | https://www.genecards.org/cgi-bin/carddisp.pl?gene=AMBP |
| CPN1 | 0.178199023 | https://www.genecards.org/cgi-bin/carddisp.pl?gene=CPN1 |
| IKBKE | 0.178199023 | https://www.genecards.org/cgi-bin/carddisp.pl?gene=IKBKE |
| IVD | 0.178199023 | https://www.genecards.org/cgi-bin/carddisp.pl?gene=IVD |
| PIGA | 0.178199023 | https://www.genecards.org/cgi-bin/carddisp.pl?gene=PIGA |
| RHOB | 0.178199023 | https://www.genecards.org/cgi-bin/carddisp.pl?gene=RHOB |
| SDC4 | 0.178199023 | https://www.genecards.org/cgi-bin/carddisp.pl?gene=SDC4 |
| SI | 0.178199023 | https://www.genecards.org/cgi-bin/carddisp.pl?gene=SI |
| UGT8 | 0.178199023 | https://www.genecards.org/cgi-bin/carddisp.pl?gene=UGT8 |
| ADIPOR1 | 0.178199023 | https://www.genecards.org/cgi-bin/carddisp.pl?gene=ADIPOR1 |
| ARHGEF7 | 0.178199023 | https://www.genecards.org/cgi-bin/carddisp.pl?gene=ARHGEF7 |
| CBX5 | 0.178199023 | https://www.genecards.org/cgi-bin/carddisp.pl?gene=CBX5 |
| CEBPE | 0.178199023 | https://www.genecards.org/cgi-bin/carddisp.pl?gene=CEBPE |
| CRH | 0.178199023 | https://www.genecards.org/cgi-bin/carddisp.pl?gene=CRH |
| HJV | 0.178199023 | https://www.genecards.org/cgi-bin/carddisp.pl?gene=HJV |
| NID1 | 0.178199023 | https://www.genecards.org/cgi-bin/carddisp.pl?gene=NID1 |
| KIRREL2 | 0.178199023 | https://www.genecards.org/cgi-bin/carddisp.pl?gene=KIRREL2 |
| KRT7 | 0.178199023 | https://www.genecards.org/cgi-bin/carddisp.pl?gene=KRT7 |
| LASP1 | 0.178199023 | https://www.genecards.org/cgi-bin/carddisp.pl?gene=LASP1 |
| PFKFB1 | 0.178199023 | https://www.genecards.org/cgi-bin/carddisp.pl?gene=PFKFB1 |
| SGCA | 0.178199023 | https://www.genecards.org/cgi-bin/carddisp.pl?gene=SGCA |
| TIA1 | 0.178199023 | https://www.genecards.org/cgi-bin/carddisp.pl?gene=TIA1 |
| ANKRD1 | 0.178199023 | https://www.genecards.org/cgi-bin/carddisp.pl?gene=ANKRD1 |
| CD48 | 0.178199023 | https://www.genecards.org/cgi-bin/carddisp.pl?gene=CD48 |
| EGR3 | 0.178199023 | https://www.genecards.org/cgi-bin/carddisp.pl?gene=EGR3 |
| F2RL2 | 0.178199023 | https://www.genecards.org/cgi-bin/carddisp.pl?gene=F2RL2 |
| LIPT1 | 0.178199023 | https://www.genecards.org/cgi-bin/carddisp.pl?gene=LIPT1 |
| OSTM1 | 0.178199023 | https://www.genecards.org/cgi-bin/carddisp.pl?gene=OSTM1 |
| PIGR | 0.178199023 | https://www.genecards.org/cgi-bin/carddisp.pl?gene=PIGR |
| TBCE | 0.178199023 | https://www.genecards.org/cgi-bin/carddisp.pl?gene=TBCE |
| TNFAIP6 | 0.178199023 | https://www.genecards.org/cgi-bin/carddisp.pl?gene=TNFAIP6 |
| ZNF148 | 0.178199023 | https://www.genecards.org/cgi-bin/carddisp.pl?gene=ZNF148 |
| AZIN2 | 0.178199023 | https://www.genecards.org/cgi-bin/carddisp.pl?gene=AZIN2 |
| CD207 | 0.178199023 | https://www.genecards.org/cgi-bin/carddisp.pl?gene=CD207 |
| DIO3 | 0.178199023 | https://www.genecards.org/cgi-bin/carddisp.pl?gene=DIO3 |
| DMBT1 | 0.178199023 | https://www.genecards.org/cgi-bin/carddisp.pl?gene=DMBT1 |
| LPA | 0.178199023 | https://www.genecards.org/cgi-bin/carddisp.pl?gene=LPA |
| NMI | 0.178199023 | https://www.genecards.org/cgi-bin/carddisp.pl?gene=NMI |
| SFRP2 | 0.178199023 | https://www.genecards.org/cgi-bin/carddisp.pl?gene=SFRP2 |
| ILF2 | 0.178199023 | https://www.genecards.org/cgi-bin/carddisp.pl?gene=ILF2 |
| JCHAIN | 0.178199023 | https://www.genecards.org/cgi-bin/carddisp.pl?gene=JCHAIN |
| AREL1 | 0.178199023 | https://www.genecards.org/cgi-bin/carddisp.pl?gene=AREL1 |
| DYNC2I2 | 0.178199023 | https://www.genecards.org/cgi-bin/carddisp.pl?gene=DYNC2I2 |
| ERGIC2 | 0.178199023 | https://www.genecards.org/cgi-bin/carddisp.pl?gene=ERGIC2 |
| CFHR4 | 0.178199023 | https://www.genecards.org/cgi-bin/carddisp.pl?gene=CFHR4 |
| H4C16 | 0.178199023 | https://www.genecards.org/cgi-bin/carddisp.pl?gene=H4C16 |
| OPN1LW | 0.178199023 | https://www.genecards.org/cgi-bin/carddisp.pl?gene=OPN1LW |
| FBXL19 | 0.178199023 | https://www.genecards.org/cgi-bin/carddisp.pl?gene=FBXL19 |
| IAH1 | 0.178199023 | https://www.genecards.org/cgi-bin/carddisp.pl?gene=IAH1 |
| MITD1 | 0.178199023 | https://www.genecards.org/cgi-bin/carddisp.pl?gene=MITD1 |
| TRIM65 | 0.178199023 | https://www.genecards.org/cgi-bin/carddisp.pl?gene=TRIM65 |
| RNFT2 | 0.178199023 | https://www.genecards.org/cgi-bin/carddisp.pl?gene=RNFT2 |
| OTUD1 | 0.178199023 | https://www.genecards.org/cgi-bin/carddisp.pl?gene=OTUD1 |
| MARCHF3 | 0.178199023 | https://www.genecards.org/cgi-bin/carddisp.pl?gene=MARCHF3 |
| DOCK8-AS1 | 0.178199023 | https://www.genecards.org/cgi-bin/carddisp.pl?gene=DOCK8-AS1 |
| PRKY | 0.178199023 | https://www.genecards.org/cgi-bin/carddisp.pl?gene=PRKY |
| SNHG29 | 0.178199023 | https://www.genecards.org/cgi-bin/carddisp.pl?gene=SNHG29 |
| HNF1A-AS1 | 0.178199023 | https://www.genecards.org/cgi-bin/carddisp.pl?gene=HNF1A-AS1 |
| APOC4-APOC2 | 0.178199023 | https://www.genecards.org/cgi-bin/carddisp.pl?gene=APOC4-APOC2 |
| CACNA1G-AS1 | 0.178199023 | https://www.genecards.org/cgi-bin/carddisp.pl?gene=CACNA1G-AS1 |
| FAM157C | 0.178199023 | https://www.genecards.org/cgi-bin/carddisp.pl?gene=FAM157C |
| MIR608 | 0.178199023 | https://www.genecards.org/cgi-bin/carddisp.pl?gene=MIR608 |
| LINC00243 | 0.178199023 | https://www.genecards.org/cgi-bin/carddisp.pl?gene=LINC00243 |
| MIR29B2CHG | 0.178199023 | https://www.genecards.org/cgi-bin/carddisp.pl?gene=MIR29B2CHG |
| MIR379 | 0.178199023 | https://www.genecards.org/cgi-bin/carddisp.pl?gene=MIR379 |
| GAPLINC | 0.178199023 | https://www.genecards.org/cgi-bin/carddisp.pl?gene=GAPLINC |
| HIF1A-AS1 | 0.178199023 | https://www.genecards.org/cgi-bin/carddisp.pl?gene=HIF1A-AS1 |
| ZFPM2-AS1 | 0.178199023 | https://www.genecards.org/cgi-bin/carddisp.pl?gene=ZFPM2-AS1 |
| VTRNA1-2 | 0.178199023 | https://www.genecards.org/cgi-bin/carddisp.pl?gene=VTRNA1-2 |
| MIR297 | 0.178199023 | https://www.genecards.org/cgi-bin/carddisp.pl?gene=MIR297 |
| ENSG00000225450 | 0.178199023 | https://www.genecards.org/cgi-bin/carddisp.pl?gene=ENSG00000225450 |
| TRV-AAC1-1 | 0.178199023 | https://www.genecards.org/cgi-bin/carddisp.pl?gene=TRV-AAC1-1 |
| TRV-AAC1-3 | 0.178199023 | https://www.genecards.org/cgi-bin/carddisp.pl?gene=TRV-AAC1-3 |
| TRV-AAC1-4 | 0.178199023 | https://www.genecards.org/cgi-bin/carddisp.pl?gene=TRV-AAC1-4 |
| TRV-AAC1-2 | 0.178199023 | https://www.genecards.org/cgi-bin/carddisp.pl?gene=TRV-AAC1-2 |
| TRV-AAC1-5 | 0.178199023 | https://www.genecards.org/cgi-bin/carddisp.pl?gene=TRV-AAC1-5 |
| IGES | 0.178199023 | https://www.genecards.org/cgi-bin/carddisp.pl?gene=IGES |
| LOC126860552 | 0.178199023 | https://www.genecards.org/cgi-bin/carddisp.pl?gene=LOC126860552 |
| AKT2 | 0.126005739 | https://www.genecards.org/cgi-bin/carddisp.pl?gene=AKT2 |
| DNMT1 | 0.126005739 | https://www.genecards.org/cgi-bin/carddisp.pl?gene=DNMT1 |
| EPHB2 | 0.126005739 | https://www.genecards.org/cgi-bin/carddisp.pl?gene=EPHB2 |
| HRAS | 0.126005739 | https://www.genecards.org/cgi-bin/carddisp.pl?gene=HRAS |
| DNMT3A | 0.126005739 | https://www.genecards.org/cgi-bin/carddisp.pl?gene=DNMT3A |
| NOTCH2 | 0.126005739 | https://www.genecards.org/cgi-bin/carddisp.pl?gene=NOTCH2 |
| ADK | 0.126005739 | https://www.genecards.org/cgi-bin/carddisp.pl?gene=ADK |
| AIFM1 | 0.126005739 | https://www.genecards.org/cgi-bin/carddisp.pl?gene=AIFM1 |
| BRCA1 | 0.126005739 | https://www.genecards.org/cgi-bin/carddisp.pl?gene=BRCA1 |
| BUB1B | 0.126005739 | https://www.genecards.org/cgi-bin/carddisp.pl?gene=BUB1B |
| DNMT3B | 0.126005739 | https://www.genecards.org/cgi-bin/carddisp.pl?gene=DNMT3B |
| ITGA6 | 0.126005739 | https://www.genecards.org/cgi-bin/carddisp.pl?gene=ITGA6 |
| MME | 0.126005739 | https://www.genecards.org/cgi-bin/carddisp.pl?gene=MME |
| PPIB | 0.126005739 | https://www.genecards.org/cgi-bin/carddisp.pl?gene=PPIB |
| PTPN1 | 0.126005739 | https://www.genecards.org/cgi-bin/carddisp.pl?gene=PTPN1 |
| RAD51 | 0.126005739 | https://www.genecards.org/cgi-bin/carddisp.pl?gene=RAD51 |
| RPS6KA1 | 0.126005739 | https://www.genecards.org/cgi-bin/carddisp.pl?gene=RPS6KA1 |
| SPR | 0.126005739 | https://www.genecards.org/cgi-bin/carddisp.pl?gene=SPR |
| TSC2 | 0.126005739 | https://www.genecards.org/cgi-bin/carddisp.pl?gene=TSC2 |
| ABCG2 | 0.126005739 | https://www.genecards.org/cgi-bin/carddisp.pl?gene=ABCG2 |
| CD247 | 0.126005739 | https://www.genecards.org/cgi-bin/carddisp.pl?gene=CD247 |
| CD55 | 0.126005739 | https://www.genecards.org/cgi-bin/carddisp.pl?gene=CD55 |
| COL1A1 | 0.126005739 | https://www.genecards.org/cgi-bin/carddisp.pl?gene=COL1A1 |
| EIF4E | 0.126005739 | https://www.genecards.org/cgi-bin/carddisp.pl?gene=EIF4E |
| HDAC3 | 0.126005739 | https://www.genecards.org/cgi-bin/carddisp.pl?gene=HDAC3 |
| HSP90AB1 | 0.126005739 | https://www.genecards.org/cgi-bin/carddisp.pl?gene=HSP90AB1 |
| MAP3K1 | 0.126005739 | https://www.genecards.org/cgi-bin/carddisp.pl?gene=MAP3K1 |
| PLD1 | 0.126005739 | https://www.genecards.org/cgi-bin/carddisp.pl?gene=PLD1 |
| PRKCH | 0.126005739 | https://www.genecards.org/cgi-bin/carddisp.pl?gene=PRKCH |
| COL1A2 | 0.126005739 | https://www.genecards.org/cgi-bin/carddisp.pl?gene=COL1A2 |
| CUL3 | 0.126005739 | https://www.genecards.org/cgi-bin/carddisp.pl?gene=CUL3 |
| FCGR2B | 0.126005739 | https://www.genecards.org/cgi-bin/carddisp.pl?gene=FCGR2B |
| GGCX | 0.126005739 | https://www.genecards.org/cgi-bin/carddisp.pl?gene=GGCX |
| HDAC9 | 0.126005739 | https://www.genecards.org/cgi-bin/carddisp.pl?gene=HDAC9 |
| ITGA5 | 0.126005739 | https://www.genecards.org/cgi-bin/carddisp.pl?gene=ITGA5 |
| KCNJ2 | 0.126005739 | https://www.genecards.org/cgi-bin/carddisp.pl?gene=KCNJ2 |
| KL | 0.126005739 | https://www.genecards.org/cgi-bin/carddisp.pl?gene=KL |
| KMT2A | 0.126005739 | https://www.genecards.org/cgi-bin/carddisp.pl?gene=KMT2A |
| LRP2 | 0.126005739 | https://www.genecards.org/cgi-bin/carddisp.pl?gene=LRP2 |
| MUC1 | 0.126005739 | https://www.genecards.org/cgi-bin/carddisp.pl?gene=MUC1 |
| MYH9 | 0.126005739 | https://www.genecards.org/cgi-bin/carddisp.pl?gene=MYH9 |
| NFATC1 | 0.126005739 | https://www.genecards.org/cgi-bin/carddisp.pl?gene=NFATC1 |
| OPRM1 | 0.126005739 | https://www.genecards.org/cgi-bin/carddisp.pl?gene=OPRM1 |
| PI4KA | 0.126005739 | https://www.genecards.org/cgi-bin/carddisp.pl?gene=PI4KA |
| PIK3CB | 0.126005739 | https://www.genecards.org/cgi-bin/carddisp.pl?gene=PIK3CB |
| POR | 0.126005739 | https://www.genecards.org/cgi-bin/carddisp.pl?gene=POR |
| PRKCZ | 0.126005739 | https://www.genecards.org/cgi-bin/carddisp.pl?gene=PRKCZ |
| SCN1A | 0.126005739 | https://www.genecards.org/cgi-bin/carddisp.pl?gene=SCN1A |
| TRPV4 | 0.126005739 | https://www.genecards.org/cgi-bin/carddisp.pl?gene=TRPV4 |
| TTN | 0.126005739 | https://www.genecards.org/cgi-bin/carddisp.pl?gene=TTN |
| CAPN2 | 0.126005739 | https://www.genecards.org/cgi-bin/carddisp.pl?gene=CAPN2 |
| CNR1 | 0.126005739 | https://www.genecards.org/cgi-bin/carddisp.pl?gene=CNR1 |
| COMP | 0.126005739 | https://www.genecards.org/cgi-bin/carddisp.pl?gene=COMP |
| CR2 | 0.126005739 | https://www.genecards.org/cgi-bin/carddisp.pl?gene=CR2 |
| EHMT2 | 0.126005739 | https://www.genecards.org/cgi-bin/carddisp.pl?gene=EHMT2 |
| EIF4G1 | 0.126005739 | https://www.genecards.org/cgi-bin/carddisp.pl?gene=EIF4G1 |
| EPHA3 | 0.126005739 | https://www.genecards.org/cgi-bin/carddisp.pl?gene=EPHA3 |
| ERN1 | 0.126005739 | https://www.genecards.org/cgi-bin/carddisp.pl?gene=ERN1 |
| FANCD2 | 0.126005739 | https://www.genecards.org/cgi-bin/carddisp.pl?gene=FANCD2 |
| FGF1 | 0.126005739 | https://www.genecards.org/cgi-bin/carddisp.pl?gene=FGF1 |
| FLNB | 0.126005739 | https://www.genecards.org/cgi-bin/carddisp.pl?gene=FLNB |
| FTO | 0.126005739 | https://www.genecards.org/cgi-bin/carddisp.pl?gene=FTO |
| GPC3 | 0.126005739 | https://www.genecards.org/cgi-bin/carddisp.pl?gene=GPC3 |
| HSP90B1 | 0.126005739 | https://www.genecards.org/cgi-bin/carddisp.pl?gene=HSP90B1 |
| ITCH | 0.126005739 | https://www.genecards.org/cgi-bin/carddisp.pl?gene=ITCH |
| KRT8 | 0.126005739 | https://www.genecards.org/cgi-bin/carddisp.pl?gene=KRT8 |
| PKLR | 0.126005739 | https://www.genecards.org/cgi-bin/carddisp.pl?gene=PKLR |
| PPARGC1A | 0.126005739 | https://www.genecards.org/cgi-bin/carddisp.pl?gene=PPARGC1A |
| PTPN22 | 0.126005739 | https://www.genecards.org/cgi-bin/carddisp.pl?gene=PTPN22 |
| SETD2 | 0.126005739 | https://www.genecards.org/cgi-bin/carddisp.pl?gene=SETD2 |
| ARG2 | 0.126005739 | https://www.genecards.org/cgi-bin/carddisp.pl?gene=ARG2 |
| ATP5F1A | 0.126005739 | https://www.genecards.org/cgi-bin/carddisp.pl?gene=ATP5F1A |
| BCL6 | 0.126005739 | https://www.genecards.org/cgi-bin/carddisp.pl?gene=BCL6 |
| CLTC | 0.126005739 | https://www.genecards.org/cgi-bin/carddisp.pl?gene=CLTC |
| COX4I1 | 0.126005739 | https://www.genecards.org/cgi-bin/carddisp.pl?gene=COX4I1 |
| ETFDH | 0.126005739 | https://www.genecards.org/cgi-bin/carddisp.pl?gene=ETFDH |
| FTL | 0.126005739 | https://www.genecards.org/cgi-bin/carddisp.pl?gene=FTL |
| GDF5 | 0.126005739 | https://www.genecards.org/cgi-bin/carddisp.pl?gene=GDF5 |
| GHSR | 0.126005739 | https://www.genecards.org/cgi-bin/carddisp.pl?gene=GHSR |
| GOT1 | 0.126005739 | https://www.genecards.org/cgi-bin/carddisp.pl?gene=GOT1 |
| GSS | 0.126005739 | https://www.genecards.org/cgi-bin/carddisp.pl?gene=GSS |
| HRH1 | 0.126005739 | https://www.genecards.org/cgi-bin/carddisp.pl?gene=HRH1 |
| IRF8 | 0.126005739 | https://www.genecards.org/cgi-bin/carddisp.pl?gene=IRF8 |
| MAP3K2 | 0.126005739 | https://www.genecards.org/cgi-bin/carddisp.pl?gene=MAP3K2 |
| MAPK13 | 0.126005739 | https://www.genecards.org/cgi-bin/carddisp.pl?gene=MAPK13 |
| MC2R | 0.126005739 | https://www.genecards.org/cgi-bin/carddisp.pl?gene=MC2R |
| MEF2A | 0.126005739 | https://www.genecards.org/cgi-bin/carddisp.pl?gene=MEF2A |
| MYH6 | 0.126005739 | https://www.genecards.org/cgi-bin/carddisp.pl?gene=MYH6 |
| NR0B1 | 0.126005739 | https://www.genecards.org/cgi-bin/carddisp.pl?gene=NR0B1 |
| NR1H3 | 0.126005739 | https://www.genecards.org/cgi-bin/carddisp.pl?gene=NR1H3 |
| OGT | 0.126005739 | https://www.genecards.org/cgi-bin/carddisp.pl?gene=OGT |
| PLCB2 | 0.126005739 | https://www.genecards.org/cgi-bin/carddisp.pl?gene=PLCB2 |
| PPIA | 0.126005739 | https://www.genecards.org/cgi-bin/carddisp.pl?gene=PPIA |
| PRKD2 | 0.126005739 | https://www.genecards.org/cgi-bin/carddisp.pl?gene=PRKD2 |
| SP1 | 0.126005739 | https://www.genecards.org/cgi-bin/carddisp.pl?gene=SP1 |
| TGFA | 0.126005739 | https://www.genecards.org/cgi-bin/carddisp.pl?gene=TGFA |
| TUBB1 | 0.126005739 | https://www.genecards.org/cgi-bin/carddisp.pl?gene=TUBB1 |
| ANG | 0.126005739 | https://www.genecards.org/cgi-bin/carddisp.pl?gene=ANG |
| APEX1 | 0.126005739 | https://www.genecards.org/cgi-bin/carddisp.pl?gene=APEX1 |
| CANT1 | 0.126005739 | https://www.genecards.org/cgi-bin/carddisp.pl?gene=CANT1 |
| CARD9 | 0.126005739 | https://www.genecards.org/cgi-bin/carddisp.pl?gene=CARD9 |
| CUBN | 0.126005739 | https://www.genecards.org/cgi-bin/carddisp.pl?gene=CUBN |
| ICOS | 0.126005739 | https://www.genecards.org/cgi-bin/carddisp.pl?gene=ICOS |
| KAT6A | 0.126005739 | https://www.genecards.org/cgi-bin/carddisp.pl?gene=KAT6A |
| PANX1 | 0.126005739 | https://www.genecards.org/cgi-bin/carddisp.pl?gene=PANX1 |
| RAD23B | 0.126005739 | https://www.genecards.org/cgi-bin/carddisp.pl?gene=RAD23B |
| SLC19A2 | 0.126005739 | https://www.genecards.org/cgi-bin/carddisp.pl?gene=SLC19A2 |
| TIAM1 | 0.126005739 | https://www.genecards.org/cgi-bin/carddisp.pl?gene=TIAM1 |
| UBE2L3 | 0.126005739 | https://www.genecards.org/cgi-bin/carddisp.pl?gene=UBE2L3 |
| ADAM15 | 0.126005739 | https://www.genecards.org/cgi-bin/carddisp.pl?gene=ADAM15 |
| ADGRE5 | 0.126005739 | https://www.genecards.org/cgi-bin/carddisp.pl?gene=ADGRE5 |
| AMPD3 | 0.126005739 | https://www.genecards.org/cgi-bin/carddisp.pl?gene=AMPD3 |
| APOC2 | 0.126005739 | https://www.genecards.org/cgi-bin/carddisp.pl?gene=APOC2 |
| ARID1B | 0.126005739 | https://www.genecards.org/cgi-bin/carddisp.pl?gene=ARID1B |
| CD9 | 0.126005739 | https://www.genecards.org/cgi-bin/carddisp.pl?gene=CD9 |
| CTSZ | 0.126005739 | https://www.genecards.org/cgi-bin/carddisp.pl?gene=CTSZ |
| FLNC | 0.126005739 | https://www.genecards.org/cgi-bin/carddisp.pl?gene=FLNC |
| HDC | 0.126005739 | https://www.genecards.org/cgi-bin/carddisp.pl?gene=HDC |
| HSPA6 | 0.126005739 | https://www.genecards.org/cgi-bin/carddisp.pl?gene=HSPA6 |
| ITGA1 | 0.126005739 | https://www.genecards.org/cgi-bin/carddisp.pl?gene=ITGA1 |
| MAP3K14 | 0.126005739 | https://www.genecards.org/cgi-bin/carddisp.pl?gene=MAP3K14 |
| NRIP1 | 0.126005739 | https://www.genecards.org/cgi-bin/carddisp.pl?gene=NRIP1 |
| ORAI1 | 0.126005739 | https://www.genecards.org/cgi-bin/carddisp.pl?gene=ORAI1 |
| PDIA3 | 0.126005739 | https://www.genecards.org/cgi-bin/carddisp.pl?gene=PDIA3 |
| PITX1 | 0.126005739 | https://www.genecards.org/cgi-bin/carddisp.pl?gene=PITX1 |
| PPP1R12A | 0.126005739 | https://www.genecards.org/cgi-bin/carddisp.pl?gene=PPP1R12A |
| SLCO2A1 | 0.126005739 | https://www.genecards.org/cgi-bin/carddisp.pl?gene=SLCO2A1 |
| SPHK2 | 0.126005739 | https://www.genecards.org/cgi-bin/carddisp.pl?gene=SPHK2 |
| STAMBP | 0.126005739 | https://www.genecards.org/cgi-bin/carddisp.pl?gene=STAMBP |
| ADSS2 | 0.126005739 | https://www.genecards.org/cgi-bin/carddisp.pl?gene=ADSS2 |
| ARHGAP1 | 0.126005739 | https://www.genecards.org/cgi-bin/carddisp.pl?gene=ARHGAP1 |
| BCAR1 | 0.126005739 | https://www.genecards.org/cgi-bin/carddisp.pl?gene=BCAR1 |
| BRCC3 | 0.126005739 | https://www.genecards.org/cgi-bin/carddisp.pl?gene=BRCC3 |
| CALU | 0.126005739 | https://www.genecards.org/cgi-bin/carddisp.pl?gene=CALU |
| CDKL5 | 0.126005739 | https://www.genecards.org/cgi-bin/carddisp.pl?gene=CDKL5 |
| CEACAM5 | 0.126005739 | https://www.genecards.org/cgi-bin/carddisp.pl?gene=CEACAM5 |
| CITED2 | 0.126005739 | https://www.genecards.org/cgi-bin/carddisp.pl?gene=CITED2 |
| CYP2R1 | 0.126005739 | https://www.genecards.org/cgi-bin/carddisp.pl?gene=CYP2R1 |
| EIF3F | 0.126005739 | https://www.genecards.org/cgi-bin/carddisp.pl?gene=EIF3F |
| FLII | 0.126005739 | https://www.genecards.org/cgi-bin/carddisp.pl?gene=FLII |
| LGALS1 | 0.126005739 | https://www.genecards.org/cgi-bin/carddisp.pl?gene=LGALS1 |
| MGAM | 0.126005739 | https://www.genecards.org/cgi-bin/carddisp.pl?gene=MGAM |
| MTO1 | 0.126005739 | https://www.genecards.org/cgi-bin/carddisp.pl?gene=MTO1 |
| OPLAH | 0.126005739 | https://www.genecards.org/cgi-bin/carddisp.pl?gene=OPLAH |
| RIPK4 | 0.126005739 | https://www.genecards.org/cgi-bin/carddisp.pl?gene=RIPK4 |
| RYK | 0.126005739 | https://www.genecards.org/cgi-bin/carddisp.pl?gene=RYK |
| SARDH | 0.126005739 | https://www.genecards.org/cgi-bin/carddisp.pl?gene=SARDH |
| SEC23A | 0.126005739 | https://www.genecards.org/cgi-bin/carddisp.pl?gene=SEC23A |
| SEMA7A | 0.126005739 | https://www.genecards.org/cgi-bin/carddisp.pl?gene=SEMA7A |
| ST3GAL4 | 0.126005739 | https://www.genecards.org/cgi-bin/carddisp.pl?gene=ST3GAL4 |
| TRADD | 0.126005739 | https://www.genecards.org/cgi-bin/carddisp.pl?gene=TRADD |
| TRIM21 | 0.126005739 | https://www.genecards.org/cgi-bin/carddisp.pl?gene=TRIM21 |
| ABCG8 | 0.126005739 | https://www.genecards.org/cgi-bin/carddisp.pl?gene=ABCG8 |
| BDKRB1 | 0.126005739 | https://www.genecards.org/cgi-bin/carddisp.pl?gene=BDKRB1 |
| CD5 | 0.126005739 | https://www.genecards.org/cgi-bin/carddisp.pl?gene=CD5 |
| CEACAM3 | 0.126005739 | https://www.genecards.org/cgi-bin/carddisp.pl?gene=CEACAM3 |
| COPS5 | 0.126005739 | https://www.genecards.org/cgi-bin/carddisp.pl?gene=COPS5 |
| CPA3 | 0.126005739 | https://www.genecards.org/cgi-bin/carddisp.pl?gene=CPA3 |
| DPP3 | 0.126005739 | https://www.genecards.org/cgi-bin/carddisp.pl?gene=DPP3 |
| DYNLL1 | 0.126005739 | https://www.genecards.org/cgi-bin/carddisp.pl?gene=DYNLL1 |
| EDAR | 0.126005739 | https://www.genecards.org/cgi-bin/carddisp.pl?gene=EDAR |
| FSTL1 | 0.126005739 | https://www.genecards.org/cgi-bin/carddisp.pl?gene=FSTL1 |
| H3-3A | 0.126005739 | https://www.genecards.org/cgi-bin/carddisp.pl?gene=H3-3A |
| HAS2 | 0.126005739 | https://www.genecards.org/cgi-bin/carddisp.pl?gene=HAS2 |
| KPNA1 | 0.126005739 | https://www.genecards.org/cgi-bin/carddisp.pl?gene=KPNA1 |
| PLSCR1 | 0.126005739 | https://www.genecards.org/cgi-bin/carddisp.pl?gene=PLSCR1 |
| RHBDF2 | 0.126005739 | https://www.genecards.org/cgi-bin/carddisp.pl?gene=RHBDF2 |
| RNF216 | 0.126005739 | https://www.genecards.org/cgi-bin/carddisp.pl?gene=RNF216 |
| RUNX1T1 | 0.126005739 | https://www.genecards.org/cgi-bin/carddisp.pl?gene=RUNX1T1 |
| TPBG | 0.126005739 | https://www.genecards.org/cgi-bin/carddisp.pl?gene=TPBG |
| VAV2 | 0.126005739 | https://www.genecards.org/cgi-bin/carddisp.pl?gene=VAV2 |
| ZBTB20 | 0.126005739 | https://www.genecards.org/cgi-bin/carddisp.pl?gene=ZBTB20 |
| ADAMTS12 | 0.126005739 | https://www.genecards.org/cgi-bin/carddisp.pl?gene=ADAMTS12 |
| ARHGEF4 | 0.126005739 | https://www.genecards.org/cgi-bin/carddisp.pl?gene=ARHGEF4 |
| ASGR1 | 0.126005739 | https://www.genecards.org/cgi-bin/carddisp.pl?gene=ASGR1 |
| ASGR2 | 0.126005739 | https://www.genecards.org/cgi-bin/carddisp.pl?gene=ASGR2 |
| CD180 | 0.126005739 | https://www.genecards.org/cgi-bin/carddisp.pl?gene=CD180 |
| CNPY3 | 0.126005739 | https://www.genecards.org/cgi-bin/carddisp.pl?gene=CNPY3 |
| DNAJA3 | 0.126005739 | https://www.genecards.org/cgi-bin/carddisp.pl?gene=DNAJA3 |
| FBXO11 | 0.126005739 | https://www.genecards.org/cgi-bin/carddisp.pl?gene=FBXO11 |
| FCAR | 0.126005739 | https://www.genecards.org/cgi-bin/carddisp.pl?gene=FCAR |
| GNRH1 | 0.126005739 | https://www.genecards.org/cgi-bin/carddisp.pl?gene=GNRH1 |
| GPER1 | 0.126005739 | https://www.genecards.org/cgi-bin/carddisp.pl?gene=GPER1 |
| H2AZ1 | 0.126005739 | https://www.genecards.org/cgi-bin/carddisp.pl?gene=H2AZ1 |
| HBA2 | 0.126005739 | https://www.genecards.org/cgi-bin/carddisp.pl?gene=HBA2 |
| IMMT | 0.126005739 | https://www.genecards.org/cgi-bin/carddisp.pl?gene=IMMT |
| JUND | 0.126005739 | https://www.genecards.org/cgi-bin/carddisp.pl?gene=JUND |
| NUCB2 | 0.126005739 | https://www.genecards.org/cgi-bin/carddisp.pl?gene=NUCB2 |
| SEPTIN9 | 0.126005739 | https://www.genecards.org/cgi-bin/carddisp.pl?gene=SEPTIN9 |
| SERPINB1 | 0.126005739 | https://www.genecards.org/cgi-bin/carddisp.pl?gene=SERPINB1 |
| SPTB | 0.126005739 | https://www.genecards.org/cgi-bin/carddisp.pl?gene=SPTB |
| ZMPSTE24 | 0.126005739 | https://www.genecards.org/cgi-bin/carddisp.pl?gene=ZMPSTE24 |
| ARHGAP35 | 0.126005739 | https://www.genecards.org/cgi-bin/carddisp.pl?gene=ARHGAP35 |
| CCL17 | 0.126005739 | https://www.genecards.org/cgi-bin/carddisp.pl?gene=CCL17 |
| CD5L | 0.126005739 | https://www.genecards.org/cgi-bin/carddisp.pl?gene=CD5L |
| CD93 | 0.126005739 | https://www.genecards.org/cgi-bin/carddisp.pl?gene=CD93 |
| CLDN5 | 0.126005739 | https://www.genecards.org/cgi-bin/carddisp.pl?gene=CLDN5 |
| FPR3 | 0.126005739 | https://www.genecards.org/cgi-bin/carddisp.pl?gene=FPR3 |
| H2BC21 | 0.126005739 | https://www.genecards.org/cgi-bin/carddisp.pl?gene=H2BC21 |
| H3-3B | 0.126005739 | https://www.genecards.org/cgi-bin/carddisp.pl?gene=H3-3B |
| HBG1 | 0.126005739 | https://www.genecards.org/cgi-bin/carddisp.pl?gene=HBG1 |
| LCN1 | 0.126005739 | https://www.genecards.org/cgi-bin/carddisp.pl?gene=LCN1 |
| MNDA | 0.126005739 | https://www.genecards.org/cgi-bin/carddisp.pl?gene=MNDA |
| PDLIM5 | 0.126005739 | https://www.genecards.org/cgi-bin/carddisp.pl?gene=PDLIM5 |
| PSMC6 | 0.126005739 | https://www.genecards.org/cgi-bin/carddisp.pl?gene=PSMC6 |
| SCRIB | 0.126005739 | https://www.genecards.org/cgi-bin/carddisp.pl?gene=SCRIB |
| SGCB | 0.126005739 | https://www.genecards.org/cgi-bin/carddisp.pl?gene=SGCB |
| SIGLEC1 | 0.126005739 | https://www.genecards.org/cgi-bin/carddisp.pl?gene=SIGLEC1 |
| SKAP2 | 0.126005739 | https://www.genecards.org/cgi-bin/carddisp.pl?gene=SKAP2 |
| SKIC3 | 0.126005739 | https://www.genecards.org/cgi-bin/carddisp.pl?gene=SKIC3 |
| TONSL | 0.126005739 | https://www.genecards.org/cgi-bin/carddisp.pl?gene=TONSL |
| CLEC4A | 0.126005739 | https://www.genecards.org/cgi-bin/carddisp.pl?gene=CLEC4A |
| EEFSEC | 0.126005739 | https://www.genecards.org/cgi-bin/carddisp.pl?gene=EEFSEC |
| GZMM | 0.126005739 | https://www.genecards.org/cgi-bin/carddisp.pl?gene=GZMM |
| HBD | 0.126005739 | https://www.genecards.org/cgi-bin/carddisp.pl?gene=HBD |
| INTS8 | 0.126005739 | https://www.genecards.org/cgi-bin/carddisp.pl?gene=INTS8 |
| PAG1 | 0.126005739 | https://www.genecards.org/cgi-bin/carddisp.pl?gene=PAG1 |
| PRSS2 | 0.126005739 | https://www.genecards.org/cgi-bin/carddisp.pl?gene=PRSS2 |
| RHBDF1 | 0.126005739 | https://www.genecards.org/cgi-bin/carddisp.pl?gene=RHBDF1 |
| SPDEF | 0.126005739 | https://www.genecards.org/cgi-bin/carddisp.pl?gene=SPDEF |
| ZC3H12A | 0.126005739 | https://www.genecards.org/cgi-bin/carddisp.pl?gene=ZC3H12A |
| C1QTNF5 | 0.126005739 | https://www.genecards.org/cgi-bin/carddisp.pl?gene=C1QTNF5 |
| CPEB4 | 0.126005739 | https://www.genecards.org/cgi-bin/carddisp.pl?gene=CPEB4 |
| ENTPD7 | 0.126005739 | https://www.genecards.org/cgi-bin/carddisp.pl?gene=ENTPD7 |
| FIS1 | 0.126005739 | https://www.genecards.org/cgi-bin/carddisp.pl?gene=FIS1 |
| H4C3 | 0.126005739 | https://www.genecards.org/cgi-bin/carddisp.pl?gene=H4C3 |
| HBE1 | 0.126005739 | https://www.genecards.org/cgi-bin/carddisp.pl?gene=HBE1 |
| HBZ | 0.126005739 | https://www.genecards.org/cgi-bin/carddisp.pl?gene=HBZ |
| IDO2 | 0.126005739 | https://www.genecards.org/cgi-bin/carddisp.pl?gene=IDO2 |
| LPCAT3 | 0.126005739 | https://www.genecards.org/cgi-bin/carddisp.pl?gene=LPCAT3 |
| LY86 | 0.126005739 | https://www.genecards.org/cgi-bin/carddisp.pl?gene=LY86 |
| SIGLEC9 | 0.126005739 | https://www.genecards.org/cgi-bin/carddisp.pl?gene=SIGLEC9 |
| CCL22 | 0.126005739 | https://www.genecards.org/cgi-bin/carddisp.pl?gene=CCL22 |
| FTMT | 0.126005739 | https://www.genecards.org/cgi-bin/carddisp.pl?gene=FTMT |
| GCHFR | 0.126005739 | https://www.genecards.org/cgi-bin/carddisp.pl?gene=GCHFR |
| IPMK | 0.126005739 | https://www.genecards.org/cgi-bin/carddisp.pl?gene=IPMK |
| SEPHS2 | 0.126005739 | https://www.genecards.org/cgi-bin/carddisp.pl?gene=SEPHS2 |
| VASH1 | 0.126005739 | https://www.genecards.org/cgi-bin/carddisp.pl?gene=VASH1 |
| CHCHD4 | 0.126005739 | https://www.genecards.org/cgi-bin/carddisp.pl?gene=CHCHD4 |
| FXYD3 | 0.126005739 | https://www.genecards.org/cgi-bin/carddisp.pl?gene=FXYD3 |
| GTF3A | 0.126005739 | https://www.genecards.org/cgi-bin/carddisp.pl?gene=GTF3A |
| H2BC11 | 0.126005739 | https://www.genecards.org/cgi-bin/carddisp.pl?gene=H2BC11 |
| H2BC9 | 0.126005739 | https://www.genecards.org/cgi-bin/carddisp.pl?gene=H2BC9 |
| H3C2 | 0.126005739 | https://www.genecards.org/cgi-bin/carddisp.pl?gene=H3C2 |
| H4C11 | 0.126005739 | https://www.genecards.org/cgi-bin/carddisp.pl?gene=H4C11 |
| H4C9 | 0.126005739 | https://www.genecards.org/cgi-bin/carddisp.pl?gene=H4C9 |
| HCAR3 | 0.126005739 | https://www.genecards.org/cgi-bin/carddisp.pl?gene=HCAR3 |
| MYDGF | 0.126005739 | https://www.genecards.org/cgi-bin/carddisp.pl?gene=MYDGF |
| PELI3 | 0.126005739 | https://www.genecards.org/cgi-bin/carddisp.pl?gene=PELI3 |
| PGAM5 | 0.126005739 | https://www.genecards.org/cgi-bin/carddisp.pl?gene=PGAM5 |
| SLCO6A1 | 0.126005739 | https://www.genecards.org/cgi-bin/carddisp.pl?gene=SLCO6A1 |
| TMED7 | 0.126005739 | https://www.genecards.org/cgi-bin/carddisp.pl?gene=TMED7 |
| FBXO33 | 0.126005739 | https://www.genecards.org/cgi-bin/carddisp.pl?gene=FBXO33 |
| H2BC14 | 0.126005739 | https://www.genecards.org/cgi-bin/carddisp.pl?gene=H2BC14 |
| H2BC3 | 0.126005739 | https://www.genecards.org/cgi-bin/carddisp.pl?gene=H2BC3 |
| H2BC4 | 0.126005739 | https://www.genecards.org/cgi-bin/carddisp.pl?gene=H2BC4 |
| H3C4 | 0.126005739 | https://www.genecards.org/cgi-bin/carddisp.pl?gene=H3C4 |
| H4C1 | 0.126005739 | https://www.genecards.org/cgi-bin/carddisp.pl?gene=H4C1 |
| H4C12 | 0.126005739 | https://www.genecards.org/cgi-bin/carddisp.pl?gene=H4C12 |
| H4C2 | 0.126005739 | https://www.genecards.org/cgi-bin/carddisp.pl?gene=H4C2 |
| H4C5 | 0.126005739 | https://www.genecards.org/cgi-bin/carddisp.pl?gene=H4C5 |
| H4C8 | 0.126005739 | https://www.genecards.org/cgi-bin/carddisp.pl?gene=H4C8 |
| PLEKHO1 | 0.126005739 | https://www.genecards.org/cgi-bin/carddisp.pl?gene=PLEKHO1 |
| ABRAXAS2 | 0.126005739 | https://www.genecards.org/cgi-bin/carddisp.pl?gene=ABRAXAS2 |
| CGB3 | 0.126005739 | https://www.genecards.org/cgi-bin/carddisp.pl?gene=CGB3 |
| DMRT3 | 0.126005739 | https://www.genecards.org/cgi-bin/carddisp.pl?gene=DMRT3 |
| ETV7 | 0.126005739 | https://www.genecards.org/cgi-bin/carddisp.pl?gene=ETV7 |
| H2BC1 | 0.126005739 | https://www.genecards.org/cgi-bin/carddisp.pl?gene=H2BC1 |
| H2BC13 | 0.126005739 | https://www.genecards.org/cgi-bin/carddisp.pl?gene=H2BC13 |
| H2BC15 | 0.126005739 | https://www.genecards.org/cgi-bin/carddisp.pl?gene=H2BC15 |
| H2BC5 | 0.126005739 | https://www.genecards.org/cgi-bin/carddisp.pl?gene=H2BC5 |
| H3C11 | 0.126005739 | https://www.genecards.org/cgi-bin/carddisp.pl?gene=H3C11 |
| H3C12 | 0.126005739 | https://www.genecards.org/cgi-bin/carddisp.pl?gene=H3C12 |
| H3C14 | 0.126005739 | https://www.genecards.org/cgi-bin/carddisp.pl?gene=H3C14 |
| H3C8 | 0.126005739 | https://www.genecards.org/cgi-bin/carddisp.pl?gene=H3C8 |
| H4C4 | 0.126005739 | https://www.genecards.org/cgi-bin/carddisp.pl?gene=H4C4 |
| H4C6 | 0.126005739 | https://www.genecards.org/cgi-bin/carddisp.pl?gene=H4C6 |
| H2BC10 | 0.126005739 | https://www.genecards.org/cgi-bin/carddisp.pl?gene=H2BC10 |
| H2BC6 | 0.126005739 | https://www.genecards.org/cgi-bin/carddisp.pl?gene=H2BC6 |
| H3C10 | 0.126005739 | https://www.genecards.org/cgi-bin/carddisp.pl?gene=H3C10 |
| H3C3 | 0.126005739 | https://www.genecards.org/cgi-bin/carddisp.pl?gene=H3C3 |
| H3C6 | 0.126005739 | https://www.genecards.org/cgi-bin/carddisp.pl?gene=H3C6 |
| RETNLB | 0.126005739 | https://www.genecards.org/cgi-bin/carddisp.pl?gene=RETNLB |
| H2BC12 | 0.126005739 | https://www.genecards.org/cgi-bin/carddisp.pl?gene=H2BC12 |
| H2BC17 | 0.126005739 | https://www.genecards.org/cgi-bin/carddisp.pl?gene=H2BC17 |
| H2BC18 | 0.126005739 | https://www.genecards.org/cgi-bin/carddisp.pl?gene=H2BC18 |
| H2BC26 | 0.126005739 | https://www.genecards.org/cgi-bin/carddisp.pl?gene=H2BC26 |
| H2BC8 | 0.126005739 | https://www.genecards.org/cgi-bin/carddisp.pl?gene=H2BC8 |
| H3C7 | 0.126005739 | https://www.genecards.org/cgi-bin/carddisp.pl?gene=H3C7 |
| H4C13 | 0.126005739 | https://www.genecards.org/cgi-bin/carddisp.pl?gene=H4C13 |
| H4C14 | 0.126005739 | https://www.genecards.org/cgi-bin/carddisp.pl?gene=H4C14 |
| ENHO | 0.126005739 | https://www.genecards.org/cgi-bin/carddisp.pl?gene=ENHO |
| H2BC7 | 0.126005739 | https://www.genecards.org/cgi-bin/carddisp.pl?gene=H2BC7 |
| H3C13 | 0.126005739 | https://www.genecards.org/cgi-bin/carddisp.pl?gene=H3C13 |
| H3C15 | 0.126005739 | https://www.genecards.org/cgi-bin/carddisp.pl?gene=H3C15 |
| H4C15 | 0.126005739 | https://www.genecards.org/cgi-bin/carddisp.pl?gene=H4C15 |
| H2BC12L | 0.126005739 | https://www.genecards.org/cgi-bin/carddisp.pl?gene=H2BC12L |
| DELEC1 | 0.126005739 | https://www.genecards.org/cgi-bin/carddisp.pl?gene=DELEC1 |
| TNXA | 0.126005739 | https://www.genecards.org/cgi-bin/carddisp.pl?gene=TNXA |
| MIR204 | 0.126005739 | https://www.genecards.org/cgi-bin/carddisp.pl?gene=MIR204 |
| DHRS4-AS1 | 0.126005739 | https://www.genecards.org/cgi-bin/carddisp.pl?gene=DHRS4-AS1 |
| JPX | 0.126005739 | https://www.genecards.org/cgi-bin/carddisp.pl?gene=JPX |
| MIR27B | 0.126005739 | https://www.genecards.org/cgi-bin/carddisp.pl?gene=MIR27B |
| MIR34C | 0.126005739 | https://www.genecards.org/cgi-bin/carddisp.pl?gene=MIR34C |
| SNHG8 | 0.126005739 | https://www.genecards.org/cgi-bin/carddisp.pl?gene=SNHG8 |
| SNORA73B | 0.126005739 | https://www.genecards.org/cgi-bin/carddisp.pl?gene=SNORA73B |
| ADAMTS9-AS2 | 0.126005739 | https://www.genecards.org/cgi-bin/carddisp.pl?gene=ADAMTS9-AS2 |
| BCYRN1 | 0.126005739 | https://www.genecards.org/cgi-bin/carddisp.pl?gene=BCYRN1 |
| EPB41L4A-AS1 | 0.126005739 | https://www.genecards.org/cgi-bin/carddisp.pl?gene=EPB41L4A-AS1 |
| MIR215 | 0.126005739 | https://www.genecards.org/cgi-bin/carddisp.pl?gene=MIR215 |
| TMEM256-PLSCR3 | 0.126005739 | https://www.genecards.org/cgi-bin/carddisp.pl?gene=TMEM256-PLSCR3 |
| ZNF561-AS1 | 0.126005739 | https://www.genecards.org/cgi-bin/carddisp.pl?gene=ZNF561-AS1 |
| MIR144 | 0.126005739 | https://www.genecards.org/cgi-bin/carddisp.pl?gene=MIR144 |
| MIR551A | 0.126005739 | https://www.genecards.org/cgi-bin/carddisp.pl?gene=MIR551A |
| ZNF503-AS1 | 0.126005739 | https://www.genecards.org/cgi-bin/carddisp.pl?gene=ZNF503-AS1 |
| CECR7 | 0.126005739 | https://www.genecards.org/cgi-bin/carddisp.pl?gene=CECR7 |
| IGKV2D-29 | 0.126005739 | https://www.genecards.org/cgi-bin/carddisp.pl?gene=IGKV2D-29 |
| LINC01082 | 0.126005739 | https://www.genecards.org/cgi-bin/carddisp.pl?gene=LINC01082 |
| MIR543 | 0.126005739 | https://www.genecards.org/cgi-bin/carddisp.pl?gene=MIR543 |
| MIR551B | 0.126005739 | https://www.genecards.org/cgi-bin/carddisp.pl?gene=MIR551B |
| MIR889 | 0.126005739 | https://www.genecards.org/cgi-bin/carddisp.pl?gene=MIR889 |
| HEIH | 0.126005739 | https://www.genecards.org/cgi-bin/carddisp.pl?gene=HEIH |
| MIR648 | 0.126005739 | https://www.genecards.org/cgi-bin/carddisp.pl?gene=MIR648 |
| NALT1 | 0.126005739 | https://www.genecards.org/cgi-bin/carddisp.pl?gene=NALT1 |
| FAS-AS1 | 0.126005739 | https://www.genecards.org/cgi-bin/carddisp.pl?gene=FAS-AS1 |
| KDM7A-DT | 0.126005739 | https://www.genecards.org/cgi-bin/carddisp.pl?gene=KDM7A-DT |
| SLC12A2-DT | 0.126005739 | https://www.genecards.org/cgi-bin/carddisp.pl?gene=SLC12A2-DT |
| THAP9-AS1 | 0.126005739 | https://www.genecards.org/cgi-bin/carddisp.pl?gene=THAP9-AS1 |
| TRG | 0.126005739 | https://www.genecards.org/cgi-bin/carddisp.pl?gene=TRG |
| LINC01215 | 0.126005739 | https://www.genecards.org/cgi-bin/carddisp.pl?gene=LINC01215 |
| LYPLAL1-DT | 0.126005739 | https://www.genecards.org/cgi-bin/carddisp.pl?gene=LYPLAL1-DT |
| MIR4707 | 0.126005739 | https://www.genecards.org/cgi-bin/carddisp.pl?gene=MIR4707 |
| MIR513B | 0.126005739 | https://www.genecards.org/cgi-bin/carddisp.pl?gene=MIR513B |
| PAXIP1-DT | 0.126005739 | https://www.genecards.org/cgi-bin/carddisp.pl?gene=PAXIP1-DT |
| MIR3682 | 0.126005739 | https://www.genecards.org/cgi-bin/carddisp.pl?gene=MIR3682 |
| MIR5100 | 0.126005739 | https://www.genecards.org/cgi-bin/carddisp.pl?gene=MIR5100 |
| RNY5 | 0.126005739 | https://www.genecards.org/cgi-bin/carddisp.pl?gene=RNY5 |
| TRD | 0.126005739 | https://www.genecards.org/cgi-bin/carddisp.pl?gene=TRD |
| TRU-TCA1-1 | 0.126005739 | https://www.genecards.org/cgi-bin/carddisp.pl?gene=TRU-TCA1-1 |
| DINOL | 0.126005739 | https://www.genecards.org/cgi-bin/carddisp.pl?gene=DINOL |
| IRF2-DT | 0.126005739 | https://www.genecards.org/cgi-bin/carddisp.pl?gene=IRF2-DT |
| ENSG00000276609 | 0.126005739 | https://www.genecards.org/cgi-bin/carddisp.pl?gene=ENSG00000276609 |
| ENSG00000274430 | 0.126005739 | https://www.genecards.org/cgi-bin/carddisp.pl?gene=ENSG00000274430 |
| ENSG00000276784 | 0.126005739 | https://www.genecards.org/cgi-bin/carddisp.pl?gene=ENSG00000276784 |
| ENSG00000277469 | 0.126005739 | https://www.genecards.org/cgi-bin/carddisp.pl?gene=ENSG00000277469 |
| ENSG00000277553 | 0.126005739 | https://www.genecards.org/cgi-bin/carddisp.pl?gene=ENSG00000277553 |
| ENSG00000278708 | 0.126005739 | https://www.genecards.org/cgi-bin/carddisp.pl?gene=ENSG00000278708 |
| H19-ICR | 0.126005739 | https://www.genecards.org/cgi-bin/carddisp.pl?gene=H19-ICR |
| AB196722-001 | 0.126005739 | https://www.genecards.org/cgi-bin/carddisp.pl?gene=AB196722-001 |
| AF420032-001 | 0.126005739 | https://www.genecards.org/cgi-bin/carddisp.pl?gene=AF420032-001 |
| MSBP1 | 0.126005739 | https://www.genecards.org/cgi-bin/carddisp.pl?gene=MSBP1 |
| lnc-IRAK3-3 | 0.126005739 | https://www.genecards.org/cgi-bin/carddisp.pl?gene=lnc-IRAK3-3 |
| LOC111365141 | 0.126005739 | https://www.genecards.org/cgi-bin/carddisp.pl?gene=LOC111365141 |
| RF00873 | 0.126005739 | https://www.genecards.org/cgi-bin/carddisp.pl?gene=RF00873 |
