# Supplementary material for: Study on the mechanism of Shenmai injection in the treatment of sepsis
Source: J Cell Mol Med. 2024 Nov 25;28(22):e70201. doi: 10.1111/jcmm.70201 (PMC11586680; doi:10.1111/jcmm.70201)
Supplement: Supplementary file 7 — Table S4. [file JCMM-28-e70201-s004.docx]

**Supplementary Table 4 Information of 122 SMI-sepsis potential targets**

| Gene Symbol | Uniprot ID | Relevance score | GeneCards Link |
| --- | --- | --- | --- |
| TNF | P01375 | 22.42014885 | https://www.genecards.org/cgi-bin/carddisp.pl?gene=TNF |
| MYLK | Q15746 | 9.055744171 | https://www.genecards.org/cgi-bin/carddisp.pl?gene=MYLK |
| NOS2 | P35228 | 6.217689991 | https://www.genecards.org/cgi-bin/carddisp.pl?gene=NOS2 |
| NOD2 | Q9HC29 | 5.759204865 | https://www.genecards.org/cgi-bin/carddisp.pl?gene=NOD2 |
| IL2 | P60568 | 5.616464615 | https://www.genecards.org/cgi-bin/carddisp.pl?gene=IL2 |
| MMP9 | P14780 | 5.033742428 | https://www.genecards.org/cgi-bin/carddisp.pl?gene=MMP9 |
| MAPK14 | Q16539 | 4.969906807 | https://www.genecards.org/cgi-bin/carddisp.pl?gene=MAPK14 |
| VEGFA | P15692 | 4.518950462 | https://www.genecards.org/cgi-bin/carddisp.pl?gene=VEGFA |
| CASP3 | P42574 | 3.865695953 | https://www.genecards.org/cgi-bin/carddisp.pl?gene=CASP3 |
| BCL2 | P10415 | 3.519965172 | https://www.genecards.org/cgi-bin/carddisp.pl?gene=BCL2 |
| STAT3 | P40763 | 3.488768339 | https://www.genecards.org/cgi-bin/carddisp.pl?gene=STAT3 |
| G6PD | P11413 | 3.151050568 | https://www.genecards.org/cgi-bin/carddisp.pl?gene=G6PD |
| MMP2 | P08253 | 2.967144966 | https://www.genecards.org/cgi-bin/carddisp.pl?gene=MMP2 |
| F2R | P25116 | 2.948045492 | https://www.genecards.org/cgi-bin/carddisp.pl?gene=F2R |
| FLT1 | P17948 | 2.756695986 | https://www.genecards.org/cgi-bin/carddisp.pl?gene=FLT1 |
| VDR | P11473 | 2.534542561 | https://www.genecards.org/cgi-bin/carddisp.pl?gene=VDR |
| PTAFR | P25105 | 2.512717485 | https://www.genecards.org/cgi-bin/carddisp.pl?gene=PTAFR |
| MMP8 | P22894 | 2.410068274 | https://www.genecards.org/cgi-bin/carddisp.pl?gene=MMP8 |
| SYK | P43405 | 2.361505747 | https://www.genecards.org/cgi-bin/carddisp.pl?gene=SYK |
| ADAM17 | P78536 | 2.298070431 | https://www.genecards.org/cgi-bin/carddisp.pl?gene=ADAM17 |
| NR3C1 | P04150 | 2.27613306 | https://www.genecards.org/cgi-bin/carddisp.pl?gene=NR3C1 |
| PPARA | Q07869 | 2.146111012 | https://www.genecards.org/cgi-bin/carddisp.pl?gene=PPARA |
| ADORA2A | P29274 | 2.100687981 | https://www.genecards.org/cgi-bin/carddisp.pl?gene=ADORA2A |
| HSP90AA1 | P07900 | 2.092811108 | https://www.genecards.org/cgi-bin/carddisp.pl?gene=HSP90AA1 |
| MMP1 | P03956 | 1.999551773 | https://www.genecards.org/cgi-bin/carddisp.pl?gene=MMP1 |
| CFD | P00746 | 1.96739316 | https://www.genecards.org/cgi-bin/carddisp.pl?gene=CFD |
| CASP1 | P29466 | 1.834529638 | https://www.genecards.org/cgi-bin/carddisp.pl?gene=CASP1 |
| SERPINA6 | P08185 | 1.800098419 | https://www.genecards.org/cgi-bin/carddisp.pl?gene=SERPINA6 |
| FGF2 | P09038 | 1.645575762 | https://www.genecards.org/cgi-bin/carddisp.pl?gene=FGF2 |
| MTOR | P42345 | 1.633176804 | https://www.genecards.org/cgi-bin/carddisp.pl?gene=MTOR |
| GRK2 | P25098 | 1.587096334 | https://www.genecards.org/cgi-bin/carddisp.pl?gene=GRK2 |
| S1PR1 | P21453 | 1.573537469 | https://www.genecards.org/cgi-bin/carddisp.pl?gene=S1PR1 |
| CTSB | P07858 | 1.572032332 | https://www.genecards.org/cgi-bin/carddisp.pl?gene=CTSB |
| CTSL | P07711 | 1.538269162 | https://www.genecards.org/cgi-bin/carddisp.pl?gene=CTSL |
| RAP1A | P62834 | 1.538269162 | https://www.genecards.org/cgi-bin/carddisp.pl?gene=RAP1A |
| PRKCA | P17252 | 1.507171035 | https://www.genecards.org/cgi-bin/carddisp.pl?gene=PRKCA |
| MCL1 | Q07820 | 1.462533712 | https://www.genecards.org/cgi-bin/carddisp.pl?gene=MCL1 |
| MMP3 | P08254 | 1.386663795 | https://www.genecards.org/cgi-bin/carddisp.pl?gene=MMP3 |
| LGALS3 | P17931 | 1.339089632 | https://www.genecards.org/cgi-bin/carddisp.pl?gene=LGALS3 |
| HPSE | Q9Y251 | 1.320506692 | https://www.genecards.org/cgi-bin/carddisp.pl?gene=HPSE |
| INSR | P06213 | 1.280610204 | https://www.genecards.org/cgi-bin/carddisp.pl?gene=INSR |
| BRD4 | O60885 | 1.280610204 | https://www.genecards.org/cgi-bin/carddisp.pl?gene=BRD4 |
| PDE4A | P27815 | 1.280610204 | https://www.genecards.org/cgi-bin/carddisp.pl?gene=PDE4A |
| BCL2L1 | Q07817 | 1.216346025 | https://www.genecards.org/cgi-bin/carddisp.pl?gene=BCL2L1 |
| MMP14 | P50281 | 1.184350371 | https://www.genecards.org/cgi-bin/carddisp.pl?gene=MMP14 |
| PDE4B | Q07343 | 1.184350371 | https://www.genecards.org/cgi-bin/carddisp.pl?gene=PDE4B |
| SMO | Q99835 | 1.146475673 | https://www.genecards.org/cgi-bin/carddisp.pl?gene=SMO |
| AGTR1 | P30556 | 1.131812096 | https://www.genecards.org/cgi-bin/carddisp.pl?gene=AGTR1 |
| RPS6KB1 | P23443 | 1.131812096 | https://www.genecards.org/cgi-bin/carddisp.pl?gene=RPS6KB1 |
| ANPEP | P15144 | 1.107082129 | https://www.genecards.org/cgi-bin/carddisp.pl?gene=ANPEP |
| PTGER2 | P43116 | 1.107082129 | https://www.genecards.org/cgi-bin/carddisp.pl?gene=PTGER2 |
| EGFR | P00533 | 1.080189705 | https://www.genecards.org/cgi-bin/carddisp.pl?gene=EGFR |
| PDGFRB | P09619 | 1.025066018 | https://www.genecards.org/cgi-bin/carddisp.pl?gene=PDGFRB |
| BCHE | P06276 | 1.025066018 | https://www.genecards.org/cgi-bin/carddisp.pl?gene=BCHE |
| ALOX5 | P09917 | 1.002487183 | https://www.genecards.org/cgi-bin/carddisp.pl?gene=ALOX5 |
| C3AR1 | Q16581 | 0.991775513 | https://www.genecards.org/cgi-bin/carddisp.pl?gene=C3AR1 |
| KRAS | P01116 | 0.924438059 | https://www.genecards.org/cgi-bin/carddisp.pl?gene=KRAS |
| KIT | P10721 | 0.863643289 | https://www.genecards.org/cgi-bin/carddisp.pl?gene=KIT |
| CHRM2 | P08172 | 0.863143981 | https://www.genecards.org/cgi-bin/carddisp.pl?gene=CHRM2 |
| PRKDC | P78527 | 0.817011833 | https://www.genecards.org/cgi-bin/carddisp.pl?gene=PRKDC |
| AVPR1A | P37288 | 0.79843235 | https://www.genecards.org/cgi-bin/carddisp.pl?gene=AVPR1A |
| FAAH | O00519 | 0.765214682 | https://www.genecards.org/cgi-bin/carddisp.pl?gene=FAAH |
| PTPN6 | P29350 | 0.74420166 | https://www.genecards.org/cgi-bin/carddisp.pl?gene=PTPN6 |
| ESR1 | P03372 | 0.739836812 | https://www.genecards.org/cgi-bin/carddisp.pl?gene=ESR1 |
| CYP2C9 | P11712 | 0.739836812 | https://www.genecards.org/cgi-bin/carddisp.pl?gene=CYP2C9 |
| CYP2C19 | P33261 | 0.739836812 | https://www.genecards.org/cgi-bin/carddisp.pl?gene=CYP2C19 |
| TYR | P14679 | 0.73763752 | https://www.genecards.org/cgi-bin/carddisp.pl?gene=TYR |
| DUSP3 | P51452 | 0.672972083 | https://www.genecards.org/cgi-bin/carddisp.pl?gene=DUSP3 |
| ACHE | P22303 | 0.647594213 | https://www.genecards.org/cgi-bin/carddisp.pl?gene=ACHE |
| HTR3A | P46098 | 0.647594213 | https://www.genecards.org/cgi-bin/carddisp.pl?gene=HTR3A |
| HMGCR | P04035 | 0.595269561 | https://www.genecards.org/cgi-bin/carddisp.pl?gene=HMGCR |
| PDE2A | O00408 | 0.533223391 | https://www.genecards.org/cgi-bin/carddisp.pl?gene=PDE2A |
| NR1I3 | Q14994 | 0.533223391 | https://www.genecards.org/cgi-bin/carddisp.pl?gene=NR1I3 |
| LCK | P06239 | 0.521588504 | https://www.genecards.org/cgi-bin/carddisp.pl?gene=LCK |
| ALK | Q9UM73 | 0.521588504 | https://www.genecards.org/cgi-bin/carddisp.pl?gene=ALK |
| SHH | Q15465 | 0.521588504 | https://www.genecards.org/cgi-bin/carddisp.pl?gene=SHH |
| DRD2 | P14416 | 0.521588504 | https://www.genecards.org/cgi-bin/carddisp.pl?gene=DRD2 |
| TSPO | B1AH88 | 0.521588504 | https://www.genecards.org/cgi-bin/carddisp.pl?gene=TSPO |
| UGT2B7 | P16662 | 0.521588504 | https://www.genecards.org/cgi-bin/carddisp.pl?gene=UGT2B7 |
| ATP4A | P20648 | 0.521588504 | https://www.genecards.org/cgi-bin/carddisp.pl?gene=ATP4A |
| PRKCD | Q05655 | 0.503477454 | https://www.genecards.org/cgi-bin/carddisp.pl?gene=PRKCD |
| ROCK2 | O75116 | 0.469714344 | https://www.genecards.org/cgi-bin/carddisp.pl?gene=ROCK2 |
| ATP1A1 | P05023 | 0.377471745 | https://www.genecards.org/cgi-bin/carddisp.pl?gene=ATP1A1 |
| ESRRB | O95718 | 0.377471745 | https://www.genecards.org/cgi-bin/carddisp.pl?gene=ESRRB |
| PTGS2 | P35354 | 0.333379835 | https://www.genecards.org/cgi-bin/carddisp.pl?gene=PTGS2 |
| SHBG | P04278 | 0.252011478 | https://www.genecards.org/cgi-bin/carddisp.pl?gene=SHBG |
| ABL1 | P00519 | 0.218248338 | https://www.genecards.org/cgi-bin/carddisp.pl?gene=ABL1 |
| EP300 | Q09472 | 0.178199023 | https://www.genecards.org/cgi-bin/carddisp.pl?gene=EP300 |
| TBXA2R | P21731 | 0.178199023 | https://www.genecards.org/cgi-bin/carddisp.pl?gene=TBXA2R |
| CASP7 | P55210 | 0.178199023 | https://www.genecards.org/cgi-bin/carddisp.pl?gene=CASP7 |
| DHCR7 | Q9UBM7 | 0.178199023 | https://www.genecards.org/cgi-bin/carddisp.pl?gene=DHCR7 |
| NR1H2 | P55055 | 0.178199023 | https://www.genecards.org/cgi-bin/carddisp.pl?gene=NR1H2 |
| AKT2 | P31751 | 0.126005739 | https://www.genecards.org/cgi-bin/carddisp.pl?gene=AKT2 |
| EPHB2 | P29323 | 0.126005739 | https://www.genecards.org/cgi-bin/carddisp.pl?gene=EPHB2 |
| PTPN1 | P18031 | 0.126005739 | https://www.genecards.org/cgi-bin/carddisp.pl?gene=PTPN1 |
| HSP90AB1 | P08238 | 0.126005739 | https://www.genecards.org/cgi-bin/carddisp.pl?gene=HSP90AB1 |
| PRKCZ | Q05513 | 0.126005739 | https://www.genecards.org/cgi-bin/carddisp.pl?gene=PRKCZ |
| EPHA3 | P29320 | 0.126005739 | https://www.genecards.org/cgi-bin/carddisp.pl?gene=EPHA3 |
| FGF1 | P05230 | 0.126005739 | https://www.genecards.org/cgi-bin/carddisp.pl?gene=FGF1 |
| HSP90B1 | P14625 | 0.126005739 | https://www.genecards.org/cgi-bin/carddisp.pl?gene=HSP90B1 |
| NR1H3 | Q13133 | 0.126005739 | https://www.genecards.org/cgi-bin/carddisp.pl?gene=NR1H3 |
| SPHK2 | Q9NRA0 | 0.126005739 | https://www.genecards.org/cgi-bin/carddisp.pl?gene=SPHK2 |
| BRD9 | Q9H8M2 | 1.571253538 | https://www.genecards.org/cgi-bin/carddisp.pl?gene=BRD9 |
| CDK4 | P11802 | 13.97164536 | https://www.genecards.org/cgi-bin/carddisp.pl?gene=CDK4 |
| CHEK2 | O96017 | 13.87888527 | https://www.genecards.org/cgi-bin/carddisp.pl?gene=CHEK2 |
| CRHR1 | P34998 | 3.222166777 | https://www.genecards.org/cgi-bin/carddisp.pl?gene=CRHR1 |
| CTSS | P25774 | 1.07932806 | https://www.genecards.org/cgi-bin/carddisp.pl?gene=CTSS |
| DNAJA1 | P31689 | 1.260759234 | https://www.genecards.org/cgi-bin/carddisp.pl?gene=DNAJA1 |
| FDFT1 | P37268 | 0.617117703 | https://www.genecards.org/cgi-bin/carddisp.pl?gene=FDFT1 |
| FKBP1A | P62942 | 1.11703968 | https://www.genecards.org/cgi-bin/carddisp.pl?gene=FKBP1A |
| HDAC1 | Q13547 | 4.609915733 | https://www.genecards.org/cgi-bin/carddisp.pl?gene=HDAC1 |
| NCSTN | Q92542 | 1.632872343 | https://www.genecards.org/cgi-bin/carddisp.pl?gene=NCSTN |
| POLB | P06746 | 1.21531105 | https://www.genecards.org/cgi-bin/carddisp.pl?gene=POLB |
| PSENEN | Q9NZ42 | 2.342224836 | https://www.genecards.org/cgi-bin/carddisp.pl?gene=PSENEN |
| RORA | P35398 | 1.93742919 | https://www.genecards.org/cgi-bin/carddisp.pl?gene=RORA |
| VCP | P55072 | 9.107070923 | https://www.genecards.org/cgi-bin/carddisp.pl?gene=VCP |
| APH1A | Q96BI3 | 38.84 | https://www.genecards.org/cgi-bin/carddisp.pl?gene=APH1A |
| APH1B | Q8WW43 | 8.22 | https://www.genecards.org/cgi-bin/carddisp.pl?gene=APH1B |
| LGALS8 | O00214 | 34.38 | https://www.genecards.org/cgi-bin/carddisp.pl?gene=LGALS8 |
| PDE1B | Q01064 | 37.05 | https://www.genecards.org/cgi-bin/carddisp.pl?gene=PDE1B |
| PDE4C | Q08493 | 34.85 | https://www.genecards.org/cgi-bin/carddisp.pl?gene=PDE4C |
| PGGT1B | P53609 | 37.41 | https://www.genecards.org/cgi-bin/carddisp.pl?gene=PGGT1B |
