# Supplementary material for: Study on the mechanism of Shenmai injection in the treatment of sepsis
Source: J Cell Mol Med. 2024 Nov 25;28(22):e70201. doi: 10.1111/jcmm.70201 (PMC11586680; doi:10.1111/jcmm.70201)
Supplement: Supplementary file 8 — Table S5. [file JCMM-28-e70201-s007.docx]

**Supplementary Table 5 Information of potential targets-organs location**

| Organs | Targets symbol |
| --- | --- |
| AdrenalCortex | INSR |
| AdrenalCortex | PDE2A |
| Amygdala | HMGCR |
| Amygdala | KIT |
| Amygdala | KRAS |
| BDCA4+_DentriticCells | LCK |
| BronchialEpithelialCells | MMP14 |
| CardiacMyocytes | BCL2 |
| CardiacMyocytes | G6PD |
| CardiacMyocytes | MMP14 |
| CardiacMyocytes | MMP2 |
| CardiacMyocytes | TSPO |
| CD14+_Monocytes | MCL1 |
| CD19+_BCells | SYK |
| CD33+_Myeloid | FKBP1A |
| CD33+_Myeloid | MAPK14 |
| CD33+_Myeloid | MCL1 |
| CD34+ | PDE4B |
| CD4+_Tcells | CASP1 |
| CD56+_NKCells | MAPK14 |
| CD56+_NKCells | SYK |
| CD8+_Tcells | MCL1 |
| CingulateCortex | PDE2A |
| CingulateCortex | PRKCA |
| Colon | CFD |
| Colorectaladenocarcinoma | PRKDC |
| Colorectaladenocarcinoma | SYK |
| Fetallung | ADAM17 |
| Heart | LCK |
| Lung | ALOX5 |
| Lung | CTSS |
| Lymphoma_burkitts | CTSS |
| Ovary | MCL1 |
| Ovary | STAT3 |
| Pancreas | ROCK2 |
| PancreaticIslet | ANPEP |
| PancreaticIslet | HSP90B1 |
| PancreaticIslet | INSR |
| PancreaticIslet | MMP1 |
| PancreaticIslet | PTGS2 |
| PancreaticIslet | PTGS2 |
| PancreaticIslet | STAT3 |
| Pineal | PRKCA |
| Pineal | RORA |
| Pineal | PDE4A |
| PrefrontalCortex | EGFR |
| PrefrontalCortex | FKBP1A |
| PrefrontalCortex | HMGCR |
| PrefrontalCortex | NR3C1 |
| PrefrontalCortex | PDE4B |
| Prostate | ABL1 |
| Prostate | LCK |
| Retina | PDGFRB |
| Small_intestine | CASP1 |
| SmoothMuscle | F2R |
| SubthalamicNucleus | PDE4B |
| SuperiorCervicalGanglion | CYP2C9 |
| Testis | PSENEN |
| TestisGermCell | APH1B |
| TestisGermCell | CFD |
| TestisGermCell | DNAJA1 |
| TestisGermCell | LGALS8 |
| TestisGermCell | MMP2 |
| TestisGermCell | MYLK |
| TestisGermCell | POLB |
| TestisGermCell | PSENEN |
| TestisGermCell | STAT3 |
| TestisIntersitial | PSENEN |
| TestisLeydigCell | LGALS8 |
| TestisLeydigCell | POLB |
| TestisSeminiferousTubule | ACHE |
| Tonsil | SYK |
| TrigeminalGanglion | FGF1 |
| TrigeminalGanglion | PDE4B |
| Uterus | ABL1 |
| WholeBlood | ALOX5 |
| WholeBlood | GRK2 |
| WholeBlood | MAPK14 |
| WholeBlood | MCL1 |
| WholeBlood | PTGS2 |
| WholeBlood | PTGS2 |
| WholeBlood | STAT3 |
| Wholebrain | FGF1 |
