# Supplementary material for: Study on the mechanism of Shenmai injection in the treatment of sepsis
Source: J Cell Mol Med. 2024 Nov 25;28(22):e70201. doi: 10.1111/jcmm.70201 (PMC11586680; doi:10.1111/jcmm.70201)
Supplement: Supplementary file 9 — Table S6. [file JCMM-28-e70201-s008.docx]

**Supplementary Table 6 Results of the biological process category terms from GO enrichment analysis**

| Term | Count | % | PValue | FDR |
| --- | --- | --- | --- | --- |
| GO:0045944~positive regulation of transcription from RNA polymerase II promoter | 27 | 22.13114754 | 2.55E-08 | 2.91E-06 |
| GO:0010628~positive regulation of gene expression | 17 | 13.93442623 | 1.26E-07 | 1.06E-05 |
| GO:0045893~positive regulation of transcription, DNA-templated | 17 | 13.93442623 | 1.04E-05 | 4.28E-04 |
| GO:0006468~protein phosphorylation | 16 | 13.1147541 | 9.96E-08 | 8.72E-06 |
| GO:0006508~proteolysis | 16 | 13.1147541 | 1.49E-07 | 1.16E-05 |
| GO:0000122~negative regulation of transcription from RNA polymerase II promoter | 15 | 12.29508197 | 0.003962522 | 0.048145787 |
| GO:0051897~positive regulation of protein kinase B signaling | 14 | 11.47540984 | 1.23E-10 | 3.51E-08 |
| GO:0018105~peptidyl-serine phosphorylation | 13 | 10.6557377 | 1.55E-09 | 2.96E-07 |
| GO:0043410~positive regulation of MAPK cascade | 12 | 9.836065574 | 9.45E-09 | 1.32E-06 |
| GO:0010629~negative regulation of gene expression | 12 | 9.836065574 | 4.60E-06 | 2.20E-04 |
| GO:0018108~peptidyl-tyrosine phosphorylation | 10 | 8.196721311 | 7.88E-11 | 3.31E-08 |
| GO:0043406~positive regulation of MAP kinase activity | 10 | 8.196721311 | 1.08E-09 | 2.53E-07 |
| GO:0007169~transmembrane receptor protein tyrosine kinase signaling pathway | 10 | 8.196721311 | 1.77E-07 | 1.28E-05 |
| GO:0046777~protein autophosphorylation | 10 | 8.196721311 | 1.93E-06 | 9.90E-05 |
| GO:0030574~collagen catabolic process | 9 | 7.37704918 | 1.33E-10 | 3.51E-08 |
| GO:0014068~positive regulation of phosphatidylinositol 3-kinase signaling | 9 | 7.37704918 | 3.00E-08 | 3.15E-06 |
| GO:0016485~protein processing | 9 | 7.37704918 | 5.88E-08 | 5.89E-06 |
| GO:0008630~intrinsic apoptotic signaling pathway in response to DNA damage | 8 | 6.557377049 | 2.63E-08 | 2.91E-06 |
| GO:0033674~positive regulation of kinase activity | 8 | 6.557377049 | 3.01E-07 | 2.04E-05 |
| GO:0022617~extracellular matrix disassembly | 7 | 5.737704918 | 3.72E-07 | 2.37E-05 |
| GO:0045429~positive regulation of nitric oxide biosynthetic process | 7 | 5.737704918 | 4.24E-07 | 2.55E-05 |
| GO:0033138~positive regulation of peptidyl-serine phosphorylation | 7 | 5.737704918 | 2.39E-05 | 8.98E-04 |
| GO:0032869~cellular response to insulin stimulus | 7 | 5.737704918 | 5.01E-05 | 0.001473945 |
| GO:0007275~multicellular organism development | 7 | 5.737704918 | 2.71E-04 | 0.006545812 |
| GO:0030198~extracellular matrix organization | 7 | 5.737704918 | 0.001166564 | 0.020243897 |
| GO:0010863~positive regulation of phospholipase C activity | 6 | 4.918032787 | 2.02E-09 | 3.54E-07 |
| GO:0030522~intracellular receptor signaling pathway | 6 | 4.918032787 | 1.98E-06 | 9.91E-05 |
| GO:0010507~negative regulation of autophagy | 6 | 4.918032787 | 4.34E-05 | 0.001341158 |
| GO:0032757~positive regulation of interleukin-8 production | 6 | 4.918032787 | 5.05E-05 | 0.001473945 |
| GO:0010595~positive regulation of endothelial cell migration | 6 | 4.918032787 | 6.28E-05 | 0.001759635 |
| GO:0050679~positive regulation of epithelial cell proliferation | 6 | 4.918032787 | 8.26E-05 | 0.002198661 |
| GO:0030324~lung development | 6 | 4.918032787 | 1.62E-04 | 0.004065658 |
| GO:0007200~phospholipase C-activating G-protein coupled receptor signaling pathway | 6 | 4.918032787 | 3.06E-04 | 0.007140334 |
| GO:0007219~Notch signaling pathway | 6 | 4.918032787 | 8.57E-04 | 0.016437377 |
| GO:0051092~positive regulation of NF-kappaB transcription factor activity | 6 | 4.918032787 | 0.0035045 | 0.045193 |
| GO:0007220~Notch receptor processing | 5 | 4.098360656 | 1.69E-07 | 1.27E-05 |
| GO:0042987~amyloid precursor protein catabolic process | 5 | 4.098360656 | 1.31E-06 | 7.27E-05 |
| GO:0038083~peptidyl-tyrosine autophosphorylation | 5 | 4.098360656 | 9.24E-06 | 3.89E-04 |
| GO:2001243~negative regulation of intrinsic apoptotic signaling pathway | 5 | 4.098360656 | 2.51E-05 | 9.27E-04 |
| GO:0043085~positive regulation of catalytic activity | 5 | 4.098360656 | 2.90E-05 | 0.001052059 |
| GO:0043552~positive regulation of phosphatidylinositol 3-kinase activity | 5 | 4.098360656 | 4.33E-05 | 0.001341158 |
| GO:0051482~positive regulation of cytosolic calcium ion concentration involved in phospholipase C-activating G-protein coupled signaling pathway | 5 | 4.098360656 | 4.33E-05 | 0.001341158 |
| GO:0002052~positive regulation of neuroblast proliferation | 5 | 4.098360656 | 7.77E-05 | 0.002094066 |
| GO:0042327~positive regulation of phosphorylation | 5 | 4.098360656 | 8.64E-05 | 0.002271086 |
| GO:0071466~cellular response to xenobiotic stimulus | 5 | 4.098360656 | 9.73E-04 | 0.017478748 |
| GO:0034205~beta-amyloid formation | 4 | 3.278688525 | 1.87E-05 | 7.29E-04 |
| GO:0071492~cellular response to UV-A | 4 | 3.278688525 | 3.64E-05 | 0.001276401 |
| GO:0010950~positive regulation of endopeptidase activity | 4 | 3.278688525 | 3.64E-05 | 0.001276401 |
| GO:0042982~amyloid precursor protein metabolic process | 4 | 3.278688525 | 4.84E-05 | 0.001452176 |
| GO:0031293~membrane protein intracellular domain proteolysis | 4 | 3.278688525 | 6.26E-05 | 0.001759635 |
| GO:0097067~cellular response to thyroid hormone stimulus | 4 | 3.278688525 | 9.87E-05 | 0.00256062 |
| GO:2000573~positive regulation of DNA biosynthetic process | 4 | 3.278688525 | 2.42E-04 | 0.005977535 |
| GO:0006509~membrane protein ectodomain proteolysis | 4 | 3.278688525 | 3.70E-04 | 0.008426546 |
| GO:0035987~endodermal cell differentiation | 4 | 3.278688525 | 9.07E-04 | 0.016437377 |
| GO:0032148~activation of protein kinase B activity | 4 | 3.278688525 | 9.07E-04 | 0.016437377 |
| GO:0097192~extrinsic apoptotic signaling pathway in absence of ligand | 4 | 3.278688525 | 0.001297312 | 0.021472047 |
| GO:2001240~negative regulation of extrinsic apoptotic signaling pathway in absence of ligand | 4 | 3.278688525 | 0.001526379 | 0.024306429 |
| GO:0051781~positive regulation of cell division | 4 | 3.278688525 | 0.003430126 | 0.04450694 |
| GO:0043536~positive regulation of blood vessel endothelial cell migration | 4 | 3.278688525 | 0.003430126 | 0.04450694 |
| GO:0042789~mRNA transcription from RNA polymerase II promoter | 4 | 3.278688525 | 0.003632811 | 0.045725559 |
| GO:0006198~cAMP catabolic process | 3 | 2.459016393 | 0.001320618 | 0.021687026 |
| GO:0031401~positive regulation of protein modification process | 3 | 2.459016393 | 0.001644118 | 0.02568804 |
| GO:0009651~response to salt stress | 3 | 2.459016393 | 0.002001382 | 0.029836199 |
| GO:0010887~negative regulation of cholesterol storage | 3 | 2.459016393 | 0.002001382 | 0.029836199 |
| GO:0030518~intracellular steroid hormone receptor signaling pathway | 3 | 2.459016393 | 0.002815513 | 0.039193439 |
| GO:0010745~negative regulation of macrophage derived foam cell differentiation | 3 | 2.459016393 | 0.00327155 | 0.0432503 |
| GO:1904645~response to beta-amyloid | 3 | 2.459016393 | 0.003759685 | 0.04621554 |
| GO:0080135~regulation of cellular response to stress | 3 | 2.459016393 | 0.003759685 | 0.04621554 |
