# Supplementary material for: Study on the mechanism of Shenmai injection in the treatment of sepsis
Source: J Cell Mol Med. 2024 Nov 25;28(22):e70201. doi: 10.1111/jcmm.70201 (PMC11586680; doi:10.1111/jcmm.70201)
Supplement: Supplementary file 10 — Table S7. [file JCMM-28-e70201-s006.docx]

**Supplementary Table 7 Results of the cellular component category terms from GO enrichment analysis**

| Term | Count | % | PValue | FDR |
| --- | --- | --- | --- | --- |
| GO:0000785~chromatin | 16 | 13.1147541 | 0.002161824 | 0.017654899 |
| GO:0043235~receptor complex | 10 | 8.196721311 | 7.25E-06 | 1.27E-04 |
| GO:0031012~extracellular matrix | 9 | 7.37704918 | 1.46E-04 | 0.001626649 |
| GO:0098978~glutamatergic synapse | 9 | 7.37704918 | 0.003645104 | 0.025515729 |
| GO:0042734~presynaptic membrane | 8 | 6.557377049 | 1.22E-05 | 1.99E-04 |
| GO:0005741~mitochondrial outer membrane | 8 | 6.557377049 | 2.87E-04 | 0.003053552 |
| GO:0010008~endosome membrane | 7 | 5.737704918 | 0.005479901 | 0.034425018 |
| GO:0099056~integral component of presynaptic membrane | 6 | 4.918032787 | 2.27E-05 | 3.28E-04 |
| GO:0045211~postsynaptic membrane | 6 | 4.918032787 | 0.007071563 | 0.042256899 |
| GO:0070765~gamma-secretase complex | 4 | 3.278688525 | 6.75E-06 | 1.27E-04 |
| GO:0099055~integral component of postsynaptic membrane | 4 | 3.278688525 | 0.002637999 | 0.020310416 |
| GO:0036021~endolysosome lumen | 3 | 2.459016393 | 3.37E-04 | 0.003437277 |
| GO:0008303~caspase complex | 3 | 2.459016393 | 9.32E-04 | 0.008154897 |
