# Supplementary material for: Study on the mechanism of Shenmai injection in the treatment of sepsis
Source: J Cell Mol Med. 2024 Nov 25;28(22):e70201. doi: 10.1111/jcmm.70201 (PMC11586680; doi:10.1111/jcmm.70201)
Supplement: Supplementary file 11 — Table S8. [file JCMM-28-e70201-s003.docx]

**Supplementary Table 8 Results of the molecular function category terms from GO enrichment analysis**

| Term | Count | % | PValue | FDR |
| --- | --- | --- | --- | --- |
| GO:0005524~ATP binding | 34 | 27.86885246 | 3.18E-10 | 3.57E-08 |
| GO:0004712~protein serine/threonine/tyrosine kinase activity | 22 | 18.03278689 | 7.04E-13 | 1.58E-10 |
| GO:0008270~zinc ion binding | 22 | 18.03278689 | 1.89E-07 | 9.41E-06 |
| GO:0004672~protein kinase activity | 16 | 13.1147541 | 2.15E-08 | 1.93E-06 |
| GO:0004674~protein serine/threonine kinase activity | 12 | 9.836065574 | 5.12E-05 | 0.001150226 |
| GO:0004879~RNA polymerase II transcription factor activity, ligand-activated sequence-specific DNA binding | 10 | 8.196721311 | 5.41E-11 | 8.10E-09 |
| GO:0004713~protein tyrosine kinase activity | 10 | 8.196721311 | 4.79E-08 | 2.80E-06 |
| GO:0016301~kinase activity | 10 | 8.196721311 | 1.81E-05 | 4.51E-04 |
| GO:0043565~sequence-specific DNA binding | 10 | 8.196721311 | 3.64E-04 | 0.006535719 |
| GO:0004175~endopeptidase activity | 9 | 7.37704918 | 3.88E-08 | 2.80E-06 |
| GO:0008233~peptidase activity | 9 | 7.37704918 | 3.61E-07 | 1.56E-05 |
| GO:0004714~transmembrane receptor protein tyrosine kinase activity | 8 | 6.557377049 | 4.99E-08 | 2.80E-06 |
| GO:0004252~serine-type endopeptidase activity | 8 | 6.557377049 | 1.98E-04 | 0.003873372 |
| GO:0004222~metalloendopeptidase activity | 7 | 5.737704918 | 8.21E-05 | 0.001754522 |
| GO:0004197~cysteine-type endopeptidase activity | 6 | 4.918032787 | 4.31E-04 | 0.007446099 |
| GO:0004115~3',5'-cyclic-AMP phosphodiesterase activity | 5 | 4.098360656 | 2.70E-06 | 9.34E-05 |
| GO:0004114~3',5'-cyclic-nucleotide phosphodiesterase activity | 5 | 4.098360656 | 8.67E-06 | 2.43E-04 |
| GO:0005496~steroid binding | 5 | 4.098360656 | 3.80E-05 | 8.98E-04 |
| GO:0042169~SH2 domain binding | 5 | 4.098360656 | 1.33E-04 | 0.002713748 |
| GO:0005518~collagen binding | 5 | 4.098360656 | 9.37E-04 | 0.013565962 |
| GO:0008237~metallopeptidase activity | 5 | 4.098360656 | 0.001101196 | 0.014982933 |
| GO:0030235~nitric-oxide synthase regulator activity | 4 | 3.278688525 | 1.39E-05 | 3.66E-04 |
| GO:0008234~cysteine-type peptidase activity | 4 | 3.278688525 | 0.001815323 | 0.02397294 |
| GO:0004715~non-membrane spanning protein tyrosine kinase activity | 4 | 3.278688525 | 0.003147831 | 0.03447259 |
| GO:0001784~phosphotyrosine binding | 4 | 3.278688525 | 0.003553626 | 0.03626314 |
| GO:0001223~transcription coactivator binding | 4 | 3.278688525 | 0.003553626 | 0.03626314 |
| GO:0051434~BH3 domain binding | 3 | 2.459016393 | 5.95E-04 | 0.009528381 |
| GO:0061133~endopeptidase activator activity | 3 | 2.459016393 | 0.002137973 | 0.02666527 |
| GO:0034056~estrogen response element binding | 3 | 2.459016393 | 0.002137973 | 0.02666527 |
| GO:0043394~proteoglycan binding | 3 | 2.459016393 | 0.003493381 | 0.03626314 |
