# Supplementary material for: Study on the mechanism of Shenmai injection in the treatment of sepsis
Source: J Cell Mol Med. 2024 Nov 25;28(22):e70201. doi: 10.1111/jcmm.70201 (PMC11586680; doi:10.1111/jcmm.70201)
Supplement: Supplementary file 12 — Table S9. [file JCMM-28-e70201-s010.docx]

**Supplementary Table 9 Information of the pathways based on KEGG enrichment analysis**

| Term | Count | % | PValue | FDR |
| --- | --- | --- | --- | --- |
| hsa04151:PI3K-Akt signaling pathway | 24 | 19.67213115 | 1.30E-10 | 4.03E-09 |
| hsa05417:Lipid and atherosclerosis | 20 | 16.39344262 | 2.72E-11 | 1.13E-09 |
| hsa04015:Rap1 signaling pathway | 17 | 13.93442623 | 1.01E-08 | 1.57E-07 |
| hsa04010:MAPK signaling pathway | 16 | 13.1147541 | 7.04E-06 | 3.79E-05 |
| hsa05163:Human cytomegalovirus infection | 15 | 12.29508197 | 1.10E-06 | 8.00E-06 |
| hsa04933:AGE-RAGE signaling pathway in diabetic complications | 14 | 11.47540984 | 3.97E-10 | 9.84E-09 |
| hsa04014:Ras signaling pathway | 14 | 11.47540984 | 1.04E-05 | 4.78E-05 |
| hsa05165:Human papillomavirus infection | 14 | 11.47540984 | 3.33E-04 | 8.80E-04 |
| hsa04072:Phospholipase D signaling pathway | 13 | 10.6557377 | 4.13E-07 | 3.93E-06 |
| hsa01521:EGFR tyrosine kinase inhibitor resistance | 12 | 9.836065574 | 4.54E-09 | 8.05E-08 |
| hsa04066:HIF-1 signaling pathway | 12 | 9.836065574 | 1.39E-07 | 1.44E-06 |
| hsa05418:Fluid shear stress and atherosclerosis | 12 | 9.836065574 | 1.64E-06 | 1.13E-05 |
| hsa05152:Tuberculosis | 12 | 9.836065574 | 1.99E-05 | 8.22E-05 |
| hsa05167:Kaposi sarcoma-associated herpesvirus infection | 12 | 9.836065574 | 3.99E-05 | 1.54E-04 |
| hsa05022:Pathways of neurodegeneration - multiple diseases | 12 | 9.836065574 | 0.043911047 | 0.044268047 |
| hsa01522:Endocrine resistance | 11 | 9.016393443 | 4.80E-07 | 4.25E-06 |
| hsa04926:Relaxin signaling pathway | 11 | 9.016393443 | 6.03E-06 | 3.40E-05 |
| hsa04210:Apoptosis | 11 | 9.016393443 | 9.66E-06 | 4.78E-05 |
| hsa05131:Shigellosis | 11 | 9.016393443 | 0.001328141 | 0.002405411 |
| hsa05132:Salmonella infection | 11 | 9.016393443 | 0.001411109 | 0.002499679 |
| hsa05235:PD-L1 expression and PD-1 checkpoint pathway in cancer | 10 | 8.196721311 | 1.99E-06 | 1.30E-05 |
| hsa04657:IL-17 signaling pathway | 10 | 8.196721311 | 3.16E-06 | 1.96E-05 |
| hsa04071:Sphingolipid signaling pathway | 10 | 8.196721311 | 2.51E-05 | 1.00E-04 |
| hsa05224:Breast cancer | 10 | 8.196721311 | 1.15E-04 | 3.67E-04 |
| hsa05161:Hepatitis B | 10 | 8.196721311 | 2.41E-04 | 6.96E-04 |
| hsa04621:NOD-like receptor signaling pathway | 10 | 8.196721311 | 6.66E-04 | 0.001583215 |
| hsa04510:Focal adhesion | 10 | 8.196721311 | 0.001238971 | 0.002373747 |
| hsa05170:Human immunodeficiency virus 1 infection | 10 | 8.196721311 | 0.001675114 | 0.002845398 |
| hsa05212:Pancreatic cancer | 9 | 7.37704918 | 5.60E-06 | 3.31E-05 |
| hsa04625:C-type lectin receptor signaling pathway | 9 | 7.37704918 | 5.67E-05 | 2.01E-04 |
| hsa05145:Toxoplasmosis | 9 | 7.37704918 | 9.01E-05 | 3.02E-04 |
| hsa05226:Gastric cancer | 9 | 7.37704918 | 6.77E-04 | 0.001583215 |
| hsa04140:Autophagy - animal | 9 | 7.37704918 | 0.001314573 | 0.002405411 |
| hsa05130:Pathogenic Escherichia coli infection | 9 | 7.37704918 | 0.004094837 | 0.006117587 |
| hsa05169:Epstein-Barr virus infection | 9 | 7.37704918 | 0.004618551 | 0.006659307 |
| hsa04919:Thyroid hormone signaling pathway | 8 | 6.557377049 | 9.53E-04 | 0.002001881 |
| hsa04660:T cell receptor signaling pathway | 8 | 6.557377049 | 9.53E-04 | 0.002001881 |
| hsa04068:FoxO signaling pathway | 8 | 6.557377049 | 0.001514606 | 0.002645228 |
| hsa04371:Apelin signaling pathway | 8 | 6.557377049 | 0.002127855 | 0.003518053 |
| hsa05225:Hepatocellular carcinoma | 8 | 6.557377049 | 0.006066589 | 0.008452326 |
| hsa05203:Viral carcinogenesis | 8 | 6.557377049 | 0.016559241 | 0.019067608 |
| hsa05219:Bladder cancer | 7 | 5.737704918 | 1.29E-05 | 5.69E-05 |
| hsa04370:VEGF signaling pathway | 7 | 5.737704918 | 1.07E-04 | 3.48E-04 |
| hsa04664:Fc epsilon RI signaling pathway | 7 | 5.737704918 | 2.36E-04 | 6.96E-04 |
| hsa05230:Central carbon metabolism in cancer | 7 | 5.737704918 | 2.76E-04 | 7.79E-04 |
| hsa05218:Melanoma | 7 | 5.737704918 | 3.23E-04 | 8.70E-04 |
| hsa05223:Non-small cell lung cancer | 7 | 5.737704918 | 3.23E-04 | 8.70E-04 |
| hsa05214:Glioma | 7 | 5.737704918 | 4.03E-04 | 0.00104078 |
| hsa04012:ErbB signaling pathway | 7 | 5.737704918 | 7.88E-04 | 0.001776863 |
| hsa05210:Colorectal cancer | 7 | 5.737704918 | 8.38E-04 | 0.001856507 |
| hsa05222:Small cell lung cancer | 7 | 5.737704918 | 0.001194339 | 0.002350763 |
| hsa05231:Choline metabolism in cancer | 7 | 5.737704918 | 0.001656039 | 0.002845398 |
| hsa04935:Growth hormone synthesis, secretion and action | 7 | 5.737704918 | 0.004570358 | 0.006659307 |
| hsa04650:Natural killer cell mediated cytotoxicity | 7 | 5.737704918 | 0.006014605 | 0.008452326 |
| hsa04910:Insulin signaling pathway | 7 | 5.737704918 | 0.008620802 | 0.011252415 |
| hsa05135:Yersinia infection | 7 | 5.737704918 | 0.008620802 | 0.011252415 |
| hsa05162:Measles | 7 | 5.737704918 | 0.008919831 | 0.011521449 |
| hsa04150:mTOR signaling pathway | 7 | 5.737704918 | 0.01565484 | 0.018313209 |
| hsa04613:Neutrophil extracellular trap formation | 7 | 5.737704918 | 0.037480982 | 0.039386795 |
| hsa04213:Longevity regulating pathway - multiple species | 6 | 4.918032787 | 0.001115133 | 0.002256337 |
| hsa05221:Acute myeloid leukemia | 6 | 4.918032787 | 0.001702396 | 0.002852664 |
| hsa05133:Pertussis | 6 | 4.918032787 | 0.002970449 | 0.004604196 |
| hsa05220:Chronic myeloid leukemia | 6 | 4.918032787 | 0.002970449 | 0.004604196 |
| hsa04936:Alcoholic liver disease | 6 | 4.918032787 | 0.036957347 | 0.039168471 |
| hsa04550:Signaling pathways regulating pluripotency of stem cells | 6 | 4.918032787 | 0.037914949 | 0.039508014 |
| hsa05134:Legionellosis | 5 | 4.098360656 | 0.005845735 | 0.008331852 |
| hsa04917:Prolactin signaling pathway | 5 | 4.098360656 | 0.012715297 | 0.015307737 |
| hsa04115:p53 signaling pathway | 5 | 4.098360656 | 0.015343036 | 0.01829362 |
| hsa05140:Leishmaniasis | 5 | 4.098360656 | 0.017523041 | 0.019934469 |
| hsa04540:Gap junction | 5 | 4.098360656 | 0.027121078 | 0.029761183 |
| hsa04211:Longevity regulating pathway | 5 | 4.098360656 | 0.028122274 | 0.030589141 |
| hsa04914:Progesterone-mediated oocyte maturation | 5 | 4.098360656 | 0.043144859 | 0.043852152 |
| hsa05142:Chagas disease | 5 | 4.098360656 | 0.043144859 | 0.043852152 |
| hsa04215:Apoptosis - multiple species | 4 | 3.278688525 | 0.007991768 | 0.010771513 |
